# Sanqi oral solution alleviates podocyte apoptosis in experimental membranous nephropathy by mediating EMT through the ERK/CK2-α/β-catenin pathway

**DOI:** 10.3389/fphar.2025.1503961

**Published:** 2025-05-09

**Authors:** Xiaowan Wang, Juanjuan Wang, Bidan Zheng, Ruimin Tian, Lihua Huang, Wei Mao, Yi Feng, Bo Liu, Peng Xu

**Affiliations:** ^1^ State Key Laboratory of Dampness Syndrome of Chinese Medicine, The Second Affiliated Hospital of Guangzhou University of Chinese Medicine, Guangzhou, China; ^2^ Department of Nephrology, Guangdong Provincial Hospital of Chinese Medicine, Guangzhou, China; ^3^ Guangdong Provincial Academy of Chinese Medical Sciences, Guangzhou, China; ^4^ Guangdong Provincial Key Laboratory of Chinese Medicine for Prevention and Treatment of Refractory Chronic Diseases, Guangzhou, China

**Keywords:** passive heymann nephropathy, sanqi oral solution, apoptosis, epithelialmesenchymal transition, the ERK/CK2-α/β-catenin pathway

## Abstract

**Introduction:**

Sanqi oral solution (SQ) is a Chinese medicine that has been used well to treat idiopathic membranous nephropathy (IMN). It has been demonstrated to mitigate IMN proteinuria by inhibiting podocyte apoptosis. however, the precise mechanism has not been fully elucidated.

**Methods:**

A passive Heymann nephropathy (PHN) rat model was used to mimic the *in vivo* disease characteristics of IMN. The PHN rats were intragastrically administered SQ (12.6/6.3 mL/kg) or tacrolimus (0.315 mg/kg) for 21 days. SQ was applied to ADR-induced podocytes *in vitro*. The effects of SQ on IMN and its underlying mechanisms were determined by measuring biochemical indices, pathomorphological characteristics, membrane attack complex (MAC), cell morphology, and protein levels.

**Results:**

The SQ ingredients found in rat serum underscored their successful absorption in rats. In PHN rats, SQ induced a significant reduction in proteinuria, MAC, C5b-9, and glomerular basement membrane thickness, along with a drop in apoptotic podocytes. Similarly, SQ exerted a protective effect against ADR-induced podocyte injury by inhibiting apoptosis. Furthermore, inhibition of the ERK/CK2-α/β-catenin pathway-mediated epithelial-to-mesenchymal transition (EMT) was found to be involved in the anti-apoptotic effect of SQ in PHN rats and podocytes, marked by the reduction in vimentin and α-SMA and the induction of Synaptopodin and Podocin protein levels.

**Conclusion:**

Inhibition of EMT via the ERK/CK2-α/β-catenin pathway may be the main mechanism by which SQ suppresses podocyte apoptosis in IMN.

## Highlights

1. The main active ingredients of SQ could be absorbed by rats, which underlay the effectiveness of SQ.
2. The ERK/CK2-α/β-catenin pathway was required for EMT in apoptosis podocytes.
3. SQ could reduce apoptotic podocytes in IMN by inhibiting EMT via the ERK/CK2-α/β-catenin pathway.

## 1 Introduction

Idiopathic membranous nephropathy (IMN) is a pathomorphologically defined proteinuric glomerular disease and a common cause of adult nephrotic syndrome (Morelle et al., 2025). IMN is typically characterized by a distinct thickening of the glomerular capillary walls resulting from the subepithelial embedding of immune deposits in the glomerular basement membrane (GBM), together with podocyte foot process detachment (Kistler and Salant, 2024). Immune deposits activate the complement cascade to produce assembled C5b-9 in the course of IMN, and podocytes are a major target in this process (Liu Z. et al., 2025). Podocytes are important barriers to maintain glomerular filtration function and are vulnerable to injury in IMN (Zhang et al., 2024). Injured podocytes detach from the GBM, inducing proteinuria (Cai et al., 2024). As specialized, highly differentiated terminal cells, podocytes will die after being injured by C5b-9 (Jiang et al., 2023). Apoptosis is the main type of podocyte death and directly associated with the degree of proteinuria and renal hypofunction (Liu et al., 2023; Wang et al., 2023). Therefore, preventing podocyte injury is of importance to relieve proteinuria and inhibit IMN progression.

How nociceptive stimuli lead to podocyte injury and apoptosis in the IMN remains debatable. In recent years, research on podocyte injury has focused on the epithelial-to-mesenchymal transition (EMT) process (Li et al., 2022; Dan Hu et al., 2023; Yue et al., 2024). During EMT, podocytes undergo a phenotypic transformation from epithelial cells to mesenchymal cells following stimulation, characterized by loss of epithelioid marker proteins such as Nephrin, Podocin, and Synaptopodin, while acquiring mesenchymal characteristics such as increased vimentin and α-SMA expression (Zheng T. et al., 2025). EMT will cause podocyte motion and affects adhesion to the GBM, resulting in the loss of podocyte integrity, fusion of podocytes, disappearance of the hiatal septum, and podocyte apoptosis (Huang et al., 2025). Therefore, podocyte EMT plays a vital role in the destruction of the glomerular filtration barrier.

Numerous studies have shown that traditional Chinese medicine has distinctive benefits in treating glomerular disease (Miao et al., 2022; Wang et al., 2024; Li et al., 2025; Wang et al., 2025). Sanqi oral solution (SQ) is a classic traditional Chinese medicine developed by the famous doctor Nizhi Yang from Guangdong Province, China. It is composed of Panax notoginseng (Burkill) F.H. Chen (Sanqi), and Astragalus mongholicus Bunge (Huangqi). SQ has been used to treat chronic kidney disease (CKD) for several years and good therapeutic efficiency has been chieved (Dai et al., 2013). Research has demonstrated that SQ or Sanqi-Huangqi herb-pair exerted renal protective effect in CKD via inhibiting macrophage inflammatory response (Wei et al., 2013; Tan et al., 2020). It was also confirmed that SQ reduces renal injury in diabetic nephropathy by inhibiting the inflammatory response of infiltrated macrophages and upregulating autophagy (Wen et al., 2020; Lin et al., 2022). According to traditional Chinese medicine, SQ has the function of promoting blood circulation and invigorating Qi in CKD, including diabetic nephropathy. Our previous study has shown that SQ could alleviate fatigue in rats with Qi deficiency and blood stasis induced by exhaustive swimming (Xu et al., 2019). As a type of CKD, IMN can also be attributed to Qi deficiency and blood stasis; hence, Chinese physicians have recommended to treat IMN with herbs that invigorate Qi and promote blood circulation (Lang et al., 2020). SQ has been used to treat IMN for many years, and SQ could effectively mitigate proteinuria and slow down IMN progression. We also found that SQ inhibited podocyte apoptosis in rats with passive Heymann nephropathy (PHN) and inhibited IMN progression (Wang et al., 2021). However, the underlying mechanism has not yet been fully elucidated.

In the present study, ERK was identified as a key protein that regulates the EMT. The influence of SQ on podocyte apoptosis via ERK-mediated EMT was also expored in IMN *in vivo* and *in vitro*.

## 2 Materials and methods

### 2.1 Analysis of SQ ingredients in rat serum using UPLC-QQQ-MS/MS

SQ ingredients in rat serum were detected using UPLC-QQQ-MS/MS with high sensitivity and specificity. Reference standard for caylcosin-7-O-β-D-glucopyranoside, notoginsenoside R1, puerarin, and saikosaponin a were purchased from the China National Institute for the Control of Pharmaceutical and Biological Products (purity 98%; Beijing, China). The reference standards for ginsenosides Rb1, Rd, Re, Rg1, calycosin, and ononin were obtained from PUSH BIO-TECHNOLOGY (Chengdu, China). UPLC-QQQ-MS/MS was performed using Shimadzu LC-30A (Shimadzu Corporation, Kyoto, Japan) and SCIEX Triple QuadTM 6500 (AB Sciex Pte. Ltd, California, United States) coupled with a mass spectroscopy system equipped with an Acquity UPLC BEH C18 column (1.7 µm, 100 × 2.1 mm). The mobile phase consisted of 5 mM ammonium acetate aqueous solution (A) and acetonitrile (B) in the gradient mode. The injection volume for each sample was 3 μL. The preparation method for the SQ-treated rat serum samples and detailed protocols are provided in the Supplementary Material.

### 2.2 Preparation of SQ

SQ (Batch No. 210102; Cantonese Medicine Ratification No. Z20071155), extracted from Astragalus mongholicus Bunge (0.333 g/mL) and Panax notoginseng (Burkill) F.H. Chen (0.056 g/mL), was provided and authenticated by Guangdong Provincial Hospital of Chinese Medicine (Guangzhou, China). The botanical entities of Astragalus mongholicus Bunge and Panax notoginseng (Burkill) F.H. Chen can be identified at https://mpns.science.kew.org/mpns-portal/. SQ was obtained via water extraction and alcohol precipitation. The main components of SQ were analyzed using high-performance liquid chromatography coupled with ultraviolet spectroscopy, as described in our previously published study (Wang et al., 2021). The lyophilized powder of Sanqi oral solution (SQL) was prepared as described in our previously published study (Wang et al., 2021). Briefly, SQ solutions were frozen in 100 mm culture dishes at −80°C for 48 h and dried under vacuum conditions at −60°C to −70°C to obtain SQL. SQL was stored at −80°C until use. The components and characteristic chromatograms of the SQ are shown in Supplementary Table S1 and Supplementary Figure S1.

### 2.3 Animals and ethics statement

Specific pathogen-free (SPF) male Sprague-Dawley (SD) rats were used in this study. Rats were purchased from the Medical Experimental Animal Center of Guangdong Province (Production Certification No. SCXK 2019-0035, Guangzhou), and maintained at the Experimental Animal Center of Guangdong Provincial Hospital of Chinese Medicine (Use Certification No. SYXK 2018-0094, Guangzhou). All rats were acclimatized for 3 days before the experiment and housed under standard conditions at a constant temperature (20°C ± 2°C), humidity (50% ± 10% humidity), and artificial lighting from 07:00 to 19:00. The rats had free access to standard laboratory food and water. All animal experiments were conducted in accordance with the guidelines of the International Association for Assessment and Accreditation of Laboratory Animal Care and were approved by the Animal Care and Use Committee of Guangdong Provincial Hospital of Chinese Medicine (Ethics approval No. 2021079).

### 2.4 PHN establishment and experimental design

The PHN model was established in rats weighing 180–220 g using a single caudal vein injection of anti-Fx1A antiserum (#PTX-002S; PROBETEX, San Antonio, CA, USA) in accordance with reagent instruction and published studies (Wang et al., 2021; Zhao et al., 2024). Thirty rats were randomly divided into five groups (n = 6 rats per group): (1) control group (CON), (2) PHN group (PHN), (3) SQ low-dose group (SQ-L), (4) SQ high-dose group (SQ-H), (5) the tacrolimus group (TAC, Batch No. 22009252, Cantonese medicine ratification No. H20083039). For animal experiment, we used equivalent doses that were one and two times the clinical dose of SQ, 6.3 mL/kg and 12.6 mL/kg, respectively. TAC was employed as the positive control (Fernández-Juárez et al., 2021), and its dosage was determined based on clinical dosage using the rat-human conversion coefficient (6.3). Rats in the PHN, SQ-L, SQ-H and TAC groups were given anti-Fx1A antiserum (0.5 mL/100g) to induce proteinuria. Rats of the SQ-L, SQ-H and TAC groups were administrated with SQ (6.3 or 12.6 mL/kg) or TAC (0.315 mg/kg) by gavage once a day in the morning for 21 days, beginning on the day of modeling. During model establishment or treatment, the rats in the CON and PHN groups received water. Throughout the experiment, rats were weighed weekly and the doses of SQ and TAC were adjusted accordingly. At the end of the experiment, the rats were euthanized by intraperitoneal injection of pentobarbital sodium (100 mg/kg) and blood and renal tissues were collected.

### 2.5 Biochemical parameters

To determine the protein levels in the urine, 24 h urine of rats was collected and centrifuged. Blood samples were obtained and centrifuged to obtain serum, which was stored at −80°C until analysis. Albuminuria and serum albumin (ALB), total cholesterol (TG), triglyceride (TC), and low-density lipoprotein cholesterol (LDL-c) levels were measured using an automatic biochemical analyzer in the clinical laboratory of Guangdong Provincial Hospital of Chinese Medicine (Guangzhou, China).

### 2.6 Histological analysis

The abdominal aorta was perfused with PBS to clear blood, and the kidneys of the rats were harvested. Renal specimens were fixed in 4% (w/v) paraformaldehyde, embedded in paraffin, and cut into 3-µm sections, which were stained with hematoxylin and eosin (H&E). General morphology was examined using a light microscope (Olympus BX53, Japan).

### 2.7 Transmission electron microscopy

The electron density in the kidneys was examined by transmission electron microscopy, as described in our previously published article (Wang et al., 2021). In brief, cubes of the renal cortex (1 mm^3^) were fixed in 5% glutaraldehyde for 2 h and immersed in 1% osmic acid for 1.five to two h. After dehydration with gradient alcohol and immersion in the embedding solution overnight, cubes were dried and cut into 50–70 nm slices, which were stained with uranyl acetate and lead citrate, and then scanned using a transmission electron microscope (JEM1400 PLUS, Japan).

### 2.8 Periodic acid-silver methenamine staining

A periodic acid-silver methenamine (PASM) staining kit (Solarbio, G1790) was used to visualize the basement membrane thickness of GBM. Briefly, paraffin sections were dewaxed in xylene and rehydrated in alcohol, followed by incubation in oxidant for 15 min, staining with Ammonium Ferric Sulfate Solution for 10 min, and staining with Methenamine Silver Working Solution at 60°C for 20–30 min until the sections turned black. The sections were soaked in Hypo Solution for 1 min, stained with Gold Chloride Solution for 1 min, and re-dyed with a Light Green Solution for 1 min. Finally, the stained sections were dehydrated using an alcohol gradient, transparentized in xylene, and sealed with neutral gum. All the sections were viewed under a light microscope (Olympus BX53, Japan). The mean density of black metal gold was measured in five randomly captured fields of six sections using Image Pro Plus (Bio-Rad Laboratories, Hercules, CA, United States).

### 2.9 Hoechst 33342 staining

Apoptotic cells in the renal cryosections from each group were visualized using Hoechst 33342 staining (Boster, AR0039) at room temperature for 5 min. The stained renal cryosections were washed with TBST solution and examined under a fluorescence microscope (Nikon Eclipse E800).

### 2.10 Cell culture

The conditionally immortalized temperature-sensitive mouse podocyte cell line was a gift from Professor Peter Mundel (Medical College, Harvard University, Boston, MA, United States). Podocytes were induced to proliferate in RPMI 1640 culture medium containing 10% fetal bovine serum and 10 U/mL recombinant IFN-γ (Sigma, St. Louis, MO, United States) at 33°C. Podocytes were induced to differentiate at 37°C in the absence of interferon-γ for 10–14 days. SQL was dissolved in PBS and stored at −20°C. Differentiated podocytes were cultured overnight at 37°C and treated with 400 ng/mL ADR, with or without 600 μg/mL SQL, for 24 h. ADR and SQL concentrations were chosen based on our previous study (Wang et al., 2021).

### 2.11 Immunofluorescence and cytoskeleton staining

OCT compound-embedded renal tissues were used for the immunofluorescence analysis of C5b-9 and Nephrin. Frozen sections (5 µm) were fixed with acetone, blocked with 5% bovine serum albumin (BSA), and stained with anti-C5b-9, anti-synaptopodin, and anti-vimentin antibodies overnight at 4°C, followed by incubation with Goat Anti-Mouse IgG H&L (Alexa Fluor^®^ 488) and Goat Anti-Rabbit IgG H&L (Alexa Fluor^®^ 594). The sections were observed under a fluorescence microscope (Olympus BX50). Fluorescence intensity was semi-quantitatively analyzed in five randomly selected fields of six slices using ImageJ software (Bio-Rad Laboratories, Hercules, CA, United States).

Cellular localization and expression of vimentin and α-SMA were examined in podocytes fixed with paraformaldehyde (w/v). After incubation with 5% bovine serum albumin (BSA) at 37°C for 30 min in the dark, podocytes were incubated with anti-vimentin or anti-α-SMA at 4°C overnight. After washing, podocytes were incubated with Goat Anti-Rabbit IgG H&L (Alexa Fluor^®^ 594) for 1 h and stained with DAPI for 5 min. To perform TUNEL staining, the ADR-induced podocyte treated with or without SQL were fixed with 4% paraformaldehyde (w/v) and incubated for 5 min at room temperature in PBS containing 0.3% Triton X-100. The One-step TUNEL Cell Apoptosis Detection Kit (Beyotime, C1089) was employed to evaluate apoptosis. For cytoskeletal staining, podocytes were fixed with 4% (w/v) paraformaldehyde for 5 min, incubated with 0.2% Triton X-100 for 10 min, and stained with 5 μg/mL phalloidin-iFluor 488 reagent (Cytoskeleton, Abcam) for 20 min in the dark. After washing, podocytes were stained with DAPI for 5 min. Finally, stained podocytes were washed with PBS and examined under a fluorescence microscope (Olympus BX50). Two to Five fields from three independent experiments were randomly selected for analysis, and the fluorescence intensity was measured using ImageJ software (Bio-Rad Laboratories, Hercules, CA, United States). Details of the antibodies are presented in Table 1.

**TABLE 1:** Ingredient sources and retention times.

No.	Ingredient	Source	MW	MRM ion pair	Retention time (min)
1	caylcosin-7-O-β-D-glucopyranoside	Radix Astragali	446.41	m/z 447.3→285.1*	3.13
2	notoginsenoside R1	Radix Notoginseng	933.14	m/z 931.6→637.5#	3.44
3	ginsenoside Re	Radix Notoginseng	947.15	m/z 945.3→637.4#	3.52
4	ginsenoside Rg1	Radix Notoginseng	801.01	m/z 799.5→637.5#	3.54
5	ononin	Radix Astragali	430.40	m/z 431.0→269.1*	3.73
6	calycosin	Radix Astragali	284.26	m/z 283.0→268.1#	3.92
7	ginsenoside Rb1	Radix Notoginseng	1109.3	m/z 1107.6→945.6#	3.95
8	ginsenoside Rd	Radix Notoginseng	947.15	m/z 945.3 →621.5#	4.15

*Positive; # Negative.

### 2.12 Western blot

Total protein was extracted from renal tissues and podocytes using a tissue Protein Extraction buffer (Thermo Fisher Scientific, Rockford, IL, United States) or RIPA Lysis buffer (Beyotime, Shanghai, China) with proteinase and phosphatase inhibitor tablets (Roche, Mannheim, Germany). The total protein concentration was determined using a BCA assay kit. Next, 5 × Loading Buffer (CWBIO, Beijing, China) was added to the lysates, which were then incubated for 10 min at 100°C. Proteins (Loading volume: 5 μL) were separated by 10%–12.5% sodium dodecyl sulfate-polyacrylamide gel electrophoresis (SDS-PAGE) and transferred onto polyvinylidene difluoride (PVDF) membranes, which were blocked and incubated overnight with the following primary antibodies: anti-Caspase-3, anti-Bax, anti-Bcl-2, anti-vimentin, anti-α-SMA, anti-ERK, anti-p-ERK, anti-CK2-α, anti-β-catenin, and anti-GAPDH. The membranes were then washed, incubated with anti-rabbit or anti-mouse IgG, and washed again. Protein bands were visualized using an enhanced chemiluminescence detection system (Bio-Rad, Laboratories, Hercules, CA, United States) and signal intensities were quantified by Image Lab software 5.2.1 (Bio-Rad, Laboratories, Hercules, CA, United States).

### 2.13 Statistical analysis

Statistical comparisons were conducted using SPSS software version 19.0. Multiple sets of independent quantitative data that follow a normal distribution are compared for mean using one-way ANOVA. If the one-way ANOVA is statistically significant, pairwise comparisons of mean values between groups are conducted. When the variances are equal, the least significant difference t-test (LSD-t test) is used to compare and analyze the differences in mean values between groups; when the variance is uneven, statistical analysis is performed using Dunnett’s T3. Results are expressed as the mean ± standard deviation (SD). Differences were considered statistically significant at *p* < 0.05, and differences were considered statistically significant at *p* < 0.01.

## 3 Results

### 3.1 The main active ingredients in SQ are absorbed by rats

**FIGURE 1:**
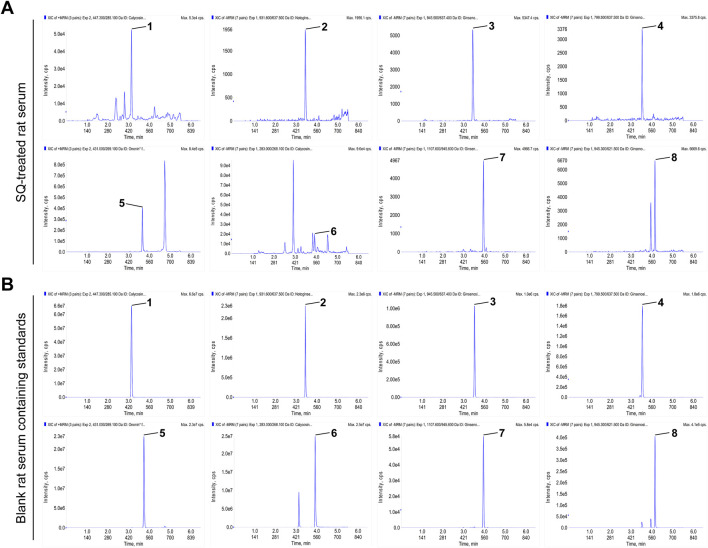
The main active ingredients of SQ were absorbed by rats. **(A, B)** MRM chromatograms of the main active ingredients of SQ in SQ-treated rat serum and blank rat serum-containing standards. (1) caylcosin-7-O-β-D-glucopyranoside, (2) notoginsenoside R1, (3) ginsenoside Re, (4) ginsenoside Rg1, (5) ononin, (6) calycosin, (7) ginsenoside Rb1, and (8) ginsenoside Rd.

In our previously published study, the main active ingredients in commercialized SQ prepared by the water extraction alcohol precipitation method according to the Chinese Pharmacopoeia were determined (Tian et al., 2020), and these ingredients were set as the standard for commercialized SQ quality control. In this study, the main absorbed ingredients of SQ in rat serum were identified using UPLC-QQQ-MS/MS with high sensitivity and specificity. Positive and negative ion switching - multi-reaction monitoring (MRM) chromatograms were obtained using two mobile phase systems (5 mM ammonium acetate aqueous solution and acetonitrile) with gradient elution. All the ingredients were separated within 5 min under optimized chromatography and mass spectrometry conditions. The representative MRM chromatogram of SQ-treated rat serum is shown in Figure 1A, and the MRM chromatogram of blank rat serum-containing standards is shown in Figure 1B. Eight ingredients (caylcosin-7-O-β-D-glucopyranoside, notoginsenoside R1, ginsenoside Re, ginsenoside Rg1, ginsenoside Rb1, calycosin, ginsenoside Rd, and ononin) of SQ, assigned to Radix Astragali and Radix Notoginseng, were identified based on their retention times and MRM ion pairs (Table 1). These data indicated that the main active ingredients of SQ could be absorbed into rat blood and were a potential pharmacological basis for SQ efficacy.

### 3.2 SQ relieved albuminuria and hyperlipidemia in PHN rats

**FIGURE 2:**
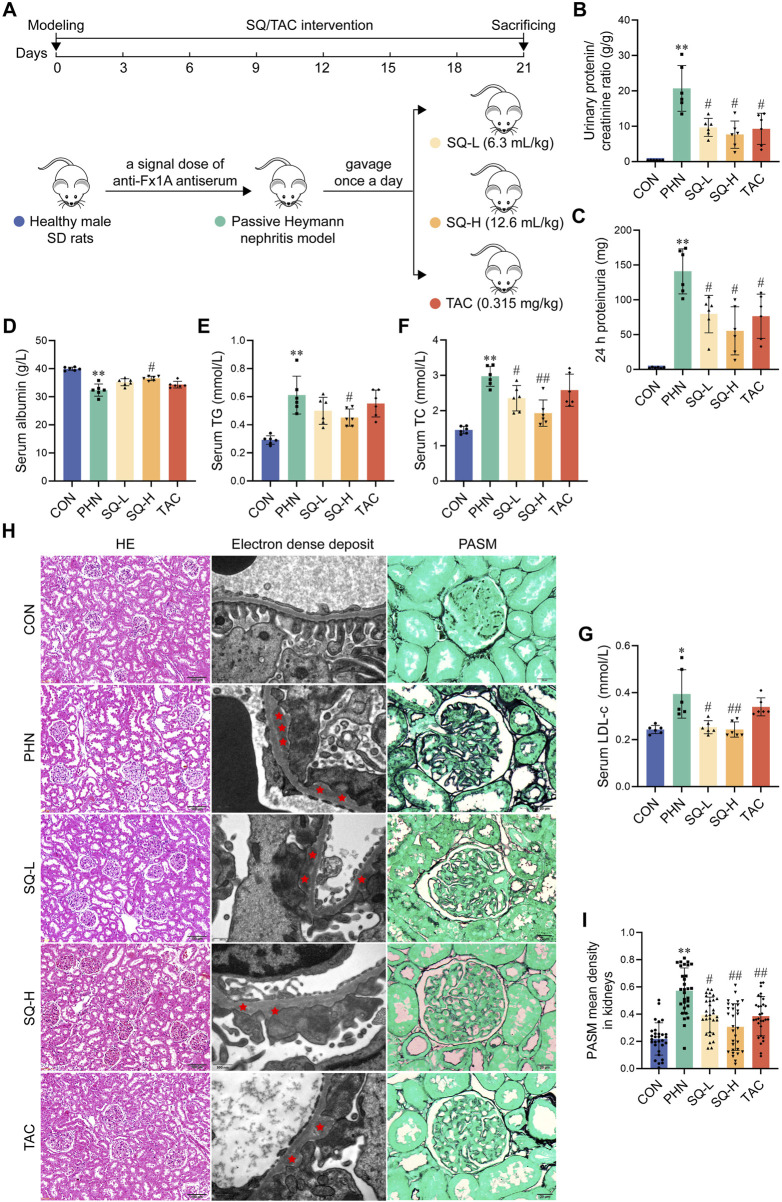
SQ relieved albuminuria, hyperlipidemia, and glomerular histopathological injury in PHN rats. **(A)** Diagram of the animal experiments. **(B, C)** Proteinuria was detected in rats from all groups, and the urinary protein/creatinine ratio and 24 h proteinuria were calculated (n = 6). **(D–G)** Serum albumin, TG, TC, and LDL-c levels were measured in the peripheral blood of rats in all the groups (n = 6). **(H)** HE staining (magnification ×200) and Transmission Electron Microscopy (magnification ×30000) showed overall histomorphological changes and microscopic electron dense deposits in the kidneys of all groups, and PASM staining (magnification ×1000) indicated the thickness of GBM in the glomeruli of all groups (n = 6). **(I)** The thickness of the GBM was semi-quantified (n = 6). Data are presented as the mean ± SD from independent groups. *p < 0.05 vs. the CON group. **p < 0.01 vs. the CON group. #*p* < 0.05 vs. the PHN group. ##*p* < 0.01 vs. the PHN group.

Figure 2A shows the procedure for performing animal experiments. Albuminuria and hyperlipidemia, which are typical manifestations of IMN, were examined in all rats. The urinary protein/creatinine ratio and 24-h proteinuria in PHN rats markedly increased (Figures 2B, C). However, treatment with SQ-L, SQ-H, and TAC inhibited the elevation of these indicators. The anti-proteinuria effect of the SQ-H was superior to that of the SQ-L. Consistent with proteinuria, serum albumin levels were lower in PHN rats than in healthy rats, and higher in SQ-H rats than in PHN rats (Figure 2D). Similarly, PHN rats had high serum TG, TC, and LDL-c levels, and the therapeutic effect of SQ-H on the serum levels of TG, TC, and LDL-c was significant (Figures 2E–G). SQ-L only exerted therapeutic effects on serum TC and LDL-c levels, whereas TAC had no significant effect on lipid metabolism biomarkers. Thus, SQ could efficiently relieve albuminuria and hyperlipidemia in PHN rats in a dose-dependent manner.

### 3.3 SQ reduced glomerular histopathological injury in PHN rats

The typical pathological characteristics of IMN are obvious at the subcellular level, including subepithelial glomerular immune complex (electron dense) deposition and thickening of the GBM, whereas it is difficult to detect histological glomerular changes at the cellular level (H&E staining). As shown in Figure 2E, H&E staining did not reveal any obvious differences in the glomerular morphology. Transmission electron microscopy images showed large amounts of subepithelial glomerular electron-dense deposits in PHN rats compared to healthy rats, and treatment with SQ and TAC significantly alleviated the pathology of PHN rats (Figure 2H). Similarly, PASM staining indicated that PHN rats had a thicker GBM than healthy rats, whereas a thinner GMB was observed in SQ- and TAC-treated PHN rats (Figures 2H, I). The mitigating effect of the SQ-H was better than that of the SQ-L. These results indicated that SQ significantly reduced glomerular histopathological injury in PHN rats in a dose-dependent manner.

### 3.4 SQ decreased glomerular apoptotic podocytes in PHN rats

**FIGURE 3:**
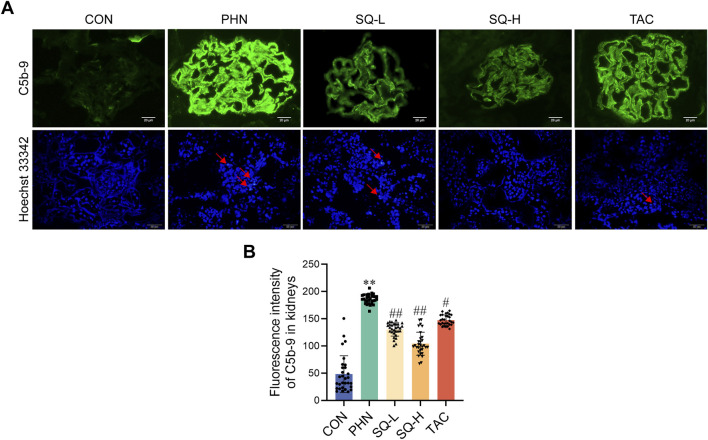
SQ reduced glomerular apoptotic podocytes in PHN rats. **(A, B)** C5b-9 assembly was visualized by immunofluorescence staining (magnification ×1000) and semi-quantified using the ImageJ software. A Hoechst 33342 staining (magnification ×400) revealed apoptotic podocytes. Data are presented as the mean ± SD from independent groups. **p < 0.01 vs. the CON group. #*p* < 0.05 vs. the PHN group. ##*p* < 0.01 vs. the PHN group.

The immune complex activates the local complement system in the glomeruli to produce C5b-9, which targets podocytes to exert cellular destructive effects and is a classic type of cell death in IMN. As shown in Figures 3A, B, deposition of C5b-9 deposition was visible in the glomeruli of PHN rats after immunofluorescence staining, and SQ decreased the expression of C5b-9 in PHN rats in a dose-dependent manner. Moreover, an increase in apoptotic podocytes was observed in PHN rats, SQ-H effectively reduced apoptosis in PHN rats (Figure 3A). However, the antiapoptotic effects of SQL were not evident. Therefore, the SQ-H was selected to study the underlying mechanism.

### 3.5 SQ reduced podocyte apoptosis by inhibiting EMT in PHN rats

**FIGURE 4:**
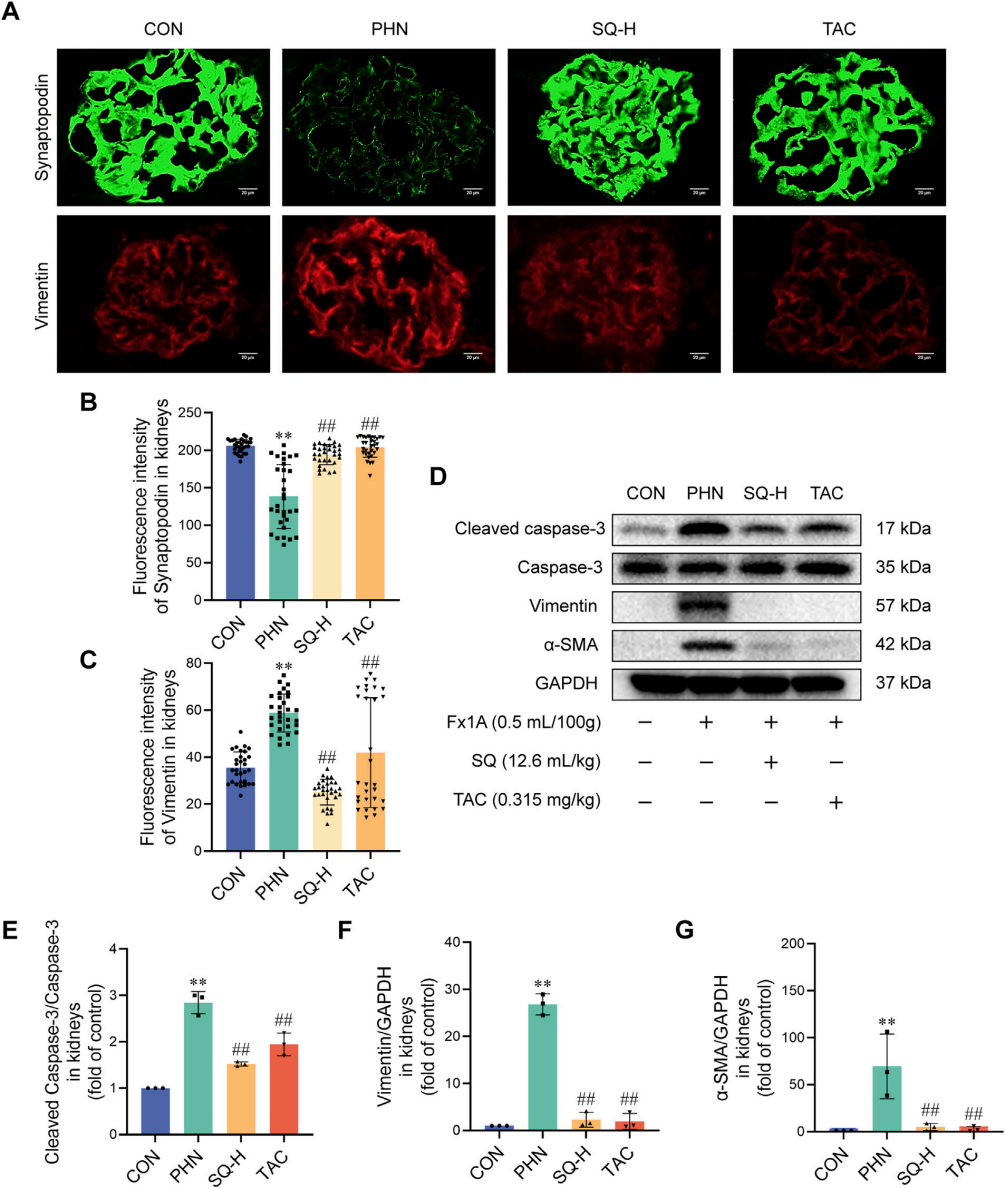
SQ reduced apoptotic podocytes by inhibiting EMT in PHN rats. **(A–C)** Synaptopodin (magnification ×1000) and Vimentin (magnification ×1000) were visualized by immunofluorescence staining and semi-quantified using the ImageJ software. **(D–G)** Protein levels of cleaved caspase-3, Caspase-3, vimentin, and α-SMA in the kidneys of all groups were detected by Western blotting. Data are presented as the mean ± SD from independent groups. **p < 0.01 vs. the CON group. ##*p* < 0.01 vs. the PHN group.

EMT can cause highly differentiated podocytes to lose their epithelial phenotype and irreversible injury. Podocyte apoptosis is most likely the type of injury induced by cellular EMT. Increased Cleaved caspase-3, vimentin, and α-SMA expression, and decreased synaptopodin expression were observed in PHN rats. SQ-H and TAC significantly inhibited the upregulation of vimentin, α-SMA, and Cleaved caspase-3, and restored the downregulation of Synaptopodin in PHN rats (Figures 4A–G). These data revealed that SQ might inhibit podocyte apoptosis by inhibiting EMT in rats with PHN. It should be noted that increasing β-catenin expression in diabetes nephropathy can trigger mitochondrial-mediated apoptosis in podocytes by Bax/Bcl-2/caspase-3 pathway (Wang et al., 2018). Besides, we have previously demonstrated that SQ alleviates mitochondrial-mediated podocyte apoptosis in IMN via Bax/Bcl-2/caspase-3 pathway (Wang et al., 2021). Thus, we believe that SQ alleviated mitochondrial-mediated apoptosis in podocyte of IMN. Bax and Bcl-2 will not be checked repeatedly in this study.

### 3.6 The ERK/CK2-α/β-catenin pathway mediated the effects of SQ on EMT in PHN rats

**FIGURE 5:**
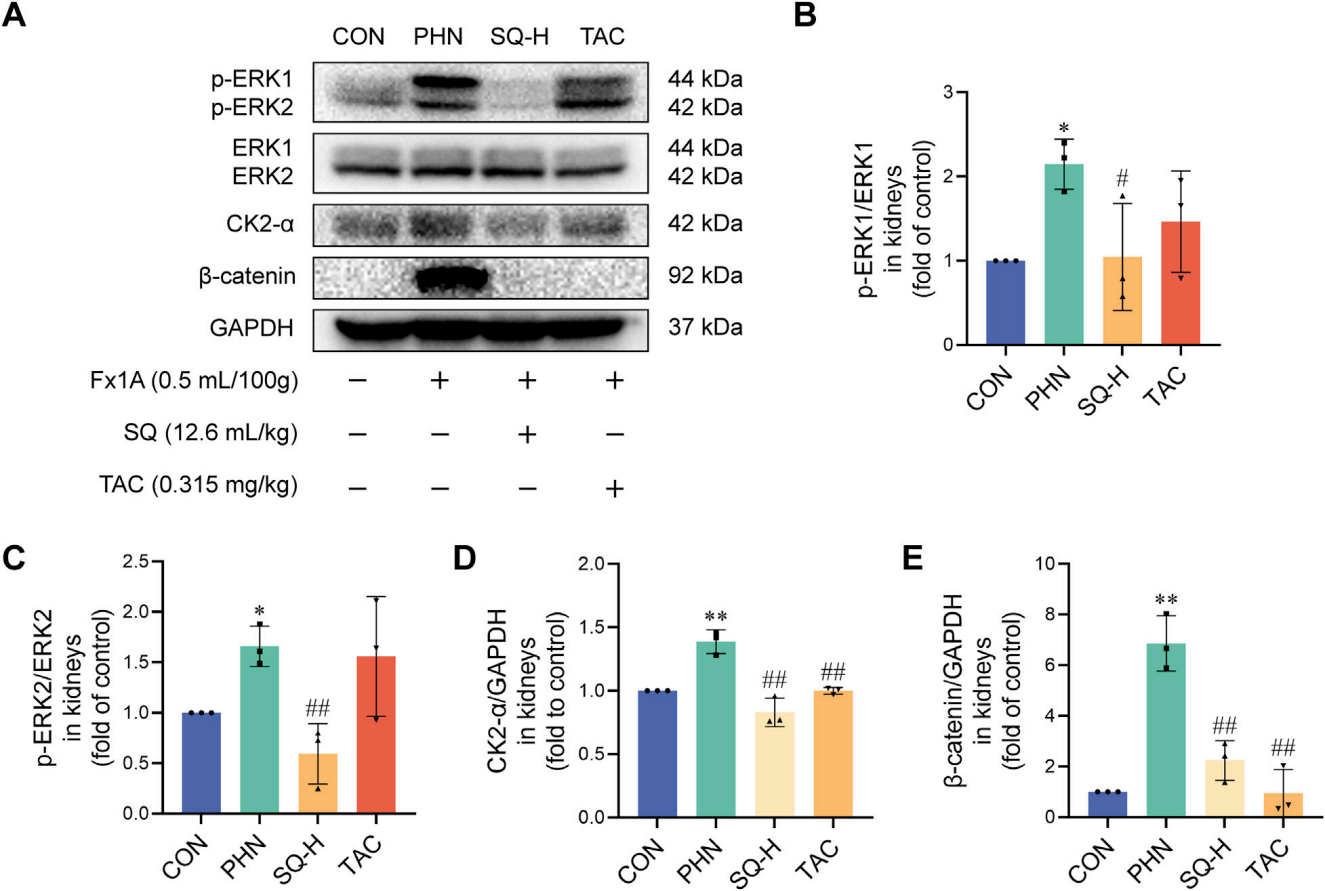
The ERK/CK2-α/β-catenin pathway mediated the effects of SQ on EMT in PHN rats. **(A–E)** Protein expression of p-ERK1, ERK1, p-ERK2, ERK2, CK2-α, and β-catenin was evaluated in the kidneys of all groups by Western blotting. Data are presented as the mean ± SD from independent groups. *p < 0.05 vs. the CON group. **p < 0.01 vs. the CON group. #*p* < 0.05 vs. the PHN group. ##*p* < 0.01 vs. the PHN group.

Next, the protein expression levels of ERK, CK2-α, and β-catenin were determined. As shown in Figures 5A–E, ERK1/2, CK2-α, and β-catenin were implicated in the EMT process in PHN rats. The levels of p-ERK1/2, CK2-α, and β-catenin were strongly increased in PHN rats, whereas SQ-H treatment significantly reduced p-ERK1/2, CK2-α, and β-catenin protein levels. These data indicate that SQ may inhibit EMT in podocytes of PHN rats via the ERK/CK2-α/β-catenin signaling pathway *in vivo*.

### 3.7 SQ alleviated ADR-induced podocyte injury

**FIGURE 6:**
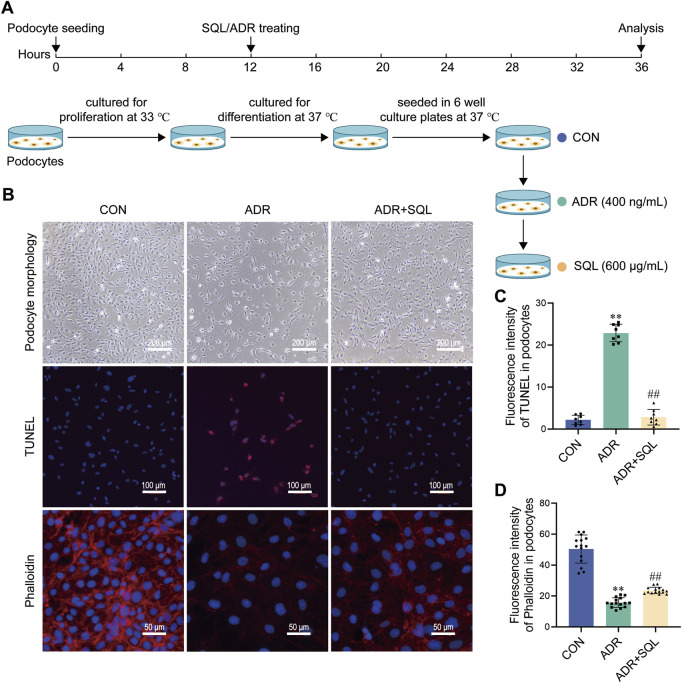
SQ alleviated ADR-induced podocyte injury. **(A)** Diagram of the cell experiments. **(B–D)** Morphological changes (magnification ×100) in podocytes were recorded using a light microscope, the nuclear DNA fragmentation of the podocyte was visualized by TUNEL staining (magnification ×200), and the podocyte cytoskeleton (magnification ×400) was visualized by immunofluorescence staining. Semi-quantified using the ImageJ software. Data are presented as the mean ± SD from independent groups. **p < 0.01 vs. the CON group. ##*p* < 0.01 vs. the PHN group.

We used an ADR-induced podocyte model to validate the protective effects of SQ against cell injury *in vitro* (Figure 6A). Podocytes were treated with or without ADR/SQL, and cellular morphology, nucleus and cytoskeleton were examined (Figures 6B–D). ADR significantly reduced podocyte size and led to pseudopodia formation and podocyte detachment; SQL could migrate these changes. Moreover, ADR caused nuclear DNA fragmentation and a dramatic loss of cytoplasmic actin stress fibers in the cytoplasm of podocytes, which was partially recovered by SQL.

### 3.8 SQ reduced apoptosis in ADR-injured podocytes by suppressing EMT

**FIGURE 7:**
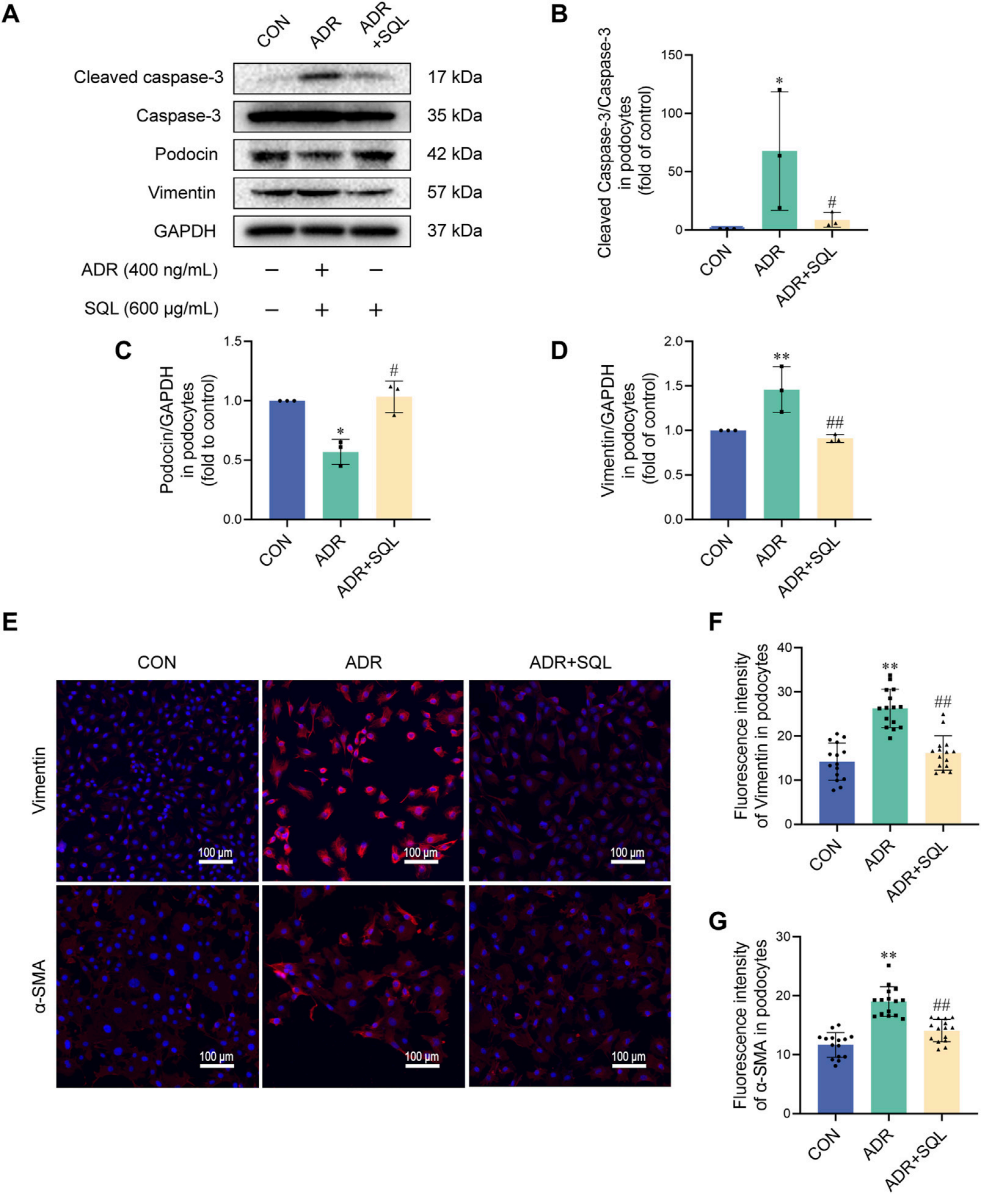
SQ reduced apoptosis in ADR-injured podocytes by suppressing EMT. **(A–D)** Cleaved caspase-3, Caspase-3, podocin, and vimentin protein levels in podocytes were measured by Western blotting. **(E–G)** Vimentin (magnification ×200) and α-SMA (magnification ×200) in podocytes were visualized **(E)** by immunofluorescence staining and semi-quantified using ImageJ software. Data are presented as the mean ± SD from independent groups. *p < 0.05 vs. the CON group. **p < 0.01 vs. the CON group. #*p* < 0.05 vs. the PHN group. ##*p* < 0.01 vs. the PHN group.

In parallel with the animal experiments, ADR increased the levels of cleaved caspase-3, the mesenchymal proteins vimentin and α-SMA, and the epithelioid marker protein podocin in podocytes *in vitro* (Figures 7A–G). SQL reduced the protein changes (Figures 7A–G).

### 3.9 SQ inhibited ERK/CK2-α/β-catenin pathway in ADR-injured podocytes

**FIGURE 8:**
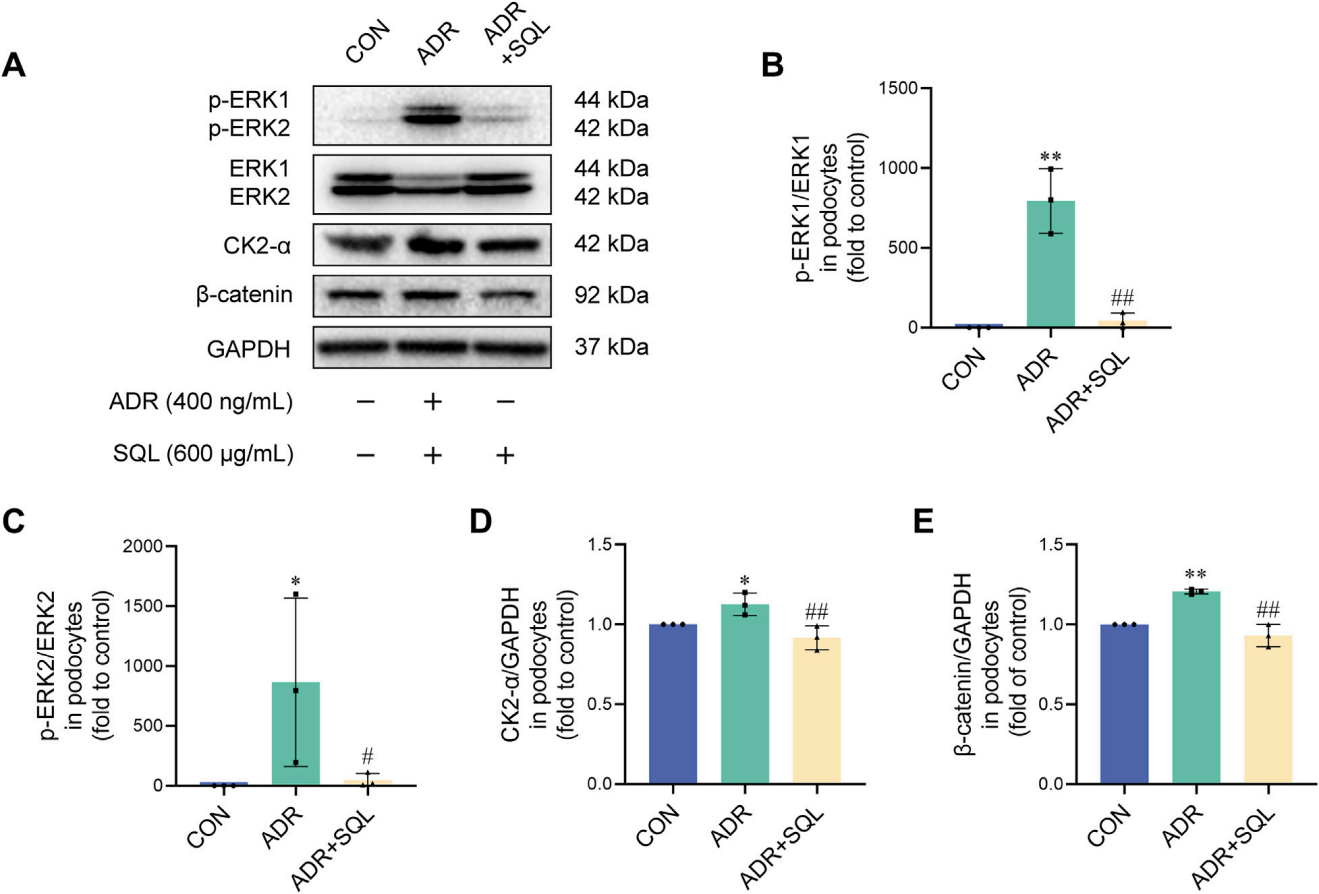
SQ inhibited the ERK/CK2-α/β-catenin pathway in ADR-injured podocytes. **(A–E)** Protein expression of p-ERK1, ERK1, p-ERK2, ERK2, CK2-α, and β-catenin in podocytes was measured by Western blotting. Data are presented as the mean ± SD from independent groups. *p < 0.05 vs. the CON group. **p < 0.01 vs. the CON group. #*p* < 0.05 vs the PHN group. ##*p* < 0.01 vs. the PHN group.

The EMT-pathway proteins ERK, CK2-α, and β-catenin were also measured in podocytes, with and without ADR/SQL intervention (Figures 8A–E). ADR significantly affected the protein expression levels of ERK, CK2-α, and β-catenin; however, SQL blocked these increases.

## 4 Discussion

In the present study, SQ was shown to be absorbed by rats, reduced albuminuria, and relieved hyperlipidemia and podocyte loss in anti-Fx1A antiserum-challenged PHN rats in a dose-dependent manner. Apoptosis was induced in PHN rat kidneys and cultured podocytes and was alleviated by SQ treatment. Surprisingly, EMT is activated, resulting in glomerular injury, which is significantly reduced by SQ through the ERK/CK2-α/β-catenin signaling pathway. These data suggest that SQ exerts protective effects against podocyte apoptosis and renal pathological injury by inhibiting EMT through the ERK/CK2-α/β-catenin signaling pathway.

Apoptosis is considered a primary mode of cell death in diverse cell types (Tian et al., 2020; Newton et al., 2024; Moyer et al., 2025), and it is also the major cause of podocyte loss and albuminuria in IMN(Yin et al., 2021). It was previously demonstrated that oxidative stress (Gomaraschi et al., 2023), inflammation (Oh et al., 2023), and mitochondrial dysfunction (Yang et al., 2024), could induce apoptosis. However, the therapeutic strategies for oxidative stress, inflammation, and mitochondrial disorders do not completely inhibit apoptosis in podocytes. Therefore, there may be another mechanism that mediates podocyte apoptosis. EMT is a newly discovered type of podocyte injury (Li et al., 2023). EMT causes podocytes to transform from an epithelial phenotype to an interstitial phenotype, leading to irreversible cell injury (Li et al., 2022). Highly differentiated podocytes lose the support of key proteins during EMT, resulting in insufficient adhesion, apoptosis, and albuminuria. In the present study, SQ almost completely inhibited the occurrence of EMT in podocytes while mitigating apoptosis. These data indicate the widespread occurrence of EMT in the kidneys of PHN rats, and suggest that EMT inhibition may be a major factor underlying the pharmacological effects of SQ.

β-catenin is a multifunctional transcriptional regulatory protein (Dai et al., 2009). While β-catenin and α-catenin at the podocyte foot processes form a complex to maintain the tight adhesion between podocytes, β-catenin in the cytoplasm of podocytes is quickly degraded by GSK-3β-mediated phosphorylation. Elevated levels of β-catenin correlate with increased foot process fusion and the loss of foot process marker Nephrin in podocyte of human proteinuria nephropathy, and β-catenin knockout led to a significant reduction of podocyte damage and proteinuria (Dai et al., 2009; Chen et al., 2023). Besides, increasing β-catenin expression in diabetes nephropathy can trigger mitochondrial-mediated apoptosis in podocytes by Bax/Bcl-2/caspase-3 pathway (Wang et al., 2018). Thus, β-catenin is closely related to the occurrence of apoptosis in podocyte and proteinuria. Furthermore, it’s confirmed that β-catenin is a key molecule that initiates podocyte EMT (Xiong et al., 2023). Inhibition of β-catenin, which is a highly conserved and ubiquitous serine and threonine protein kinase, effectively reduces EMT in apoptotic podocytes, and delays CKD (Kang et al., 2020). The increase of β-catenin in podocytes may be controlled by CK2-α. CK2-α is a highly pleiotropic protein kinase and closely related to various glomerular diseases (Li et al., 2024). CK2 is highly expressed in glomerulonephritis and renal biopsy specimens of patients with lupus nephritis, as well as in rat models of IgA nephropathy; and application of CK2 inhibitors can effectively control proteinuria and reduce podocyte apoptosis in rats with glomerulonephritis (Yamada et al., 2005). It has been confirmed that CK2-α can elevate β-catenin by inhibiting the phosphorylation of GSK-3β, but also release β-catenin by binding to α-catenin, resulting in an increase in free β-catenin in the cytoplasm (Seldin et al., 2005; Silva-Pavez and Tapia, 2020). More importantly, the activation of CK2-α is partially dependent on ERK. ERK is a serine and threonine protein kinase that belongs to the MAPK family (Liu J. et al., 2025). Activated ERK leads to acute and chronic renal disease (Jung et al., 2020; Livingston et al., 2024). An aberrant effect of ERK was also confirmed in proteinuric glomerular diseases, such as diabetic nephropathy, glomerulosclerosis, and nephritic syndrome (Das et al., 2019; Pan et al., 2025; Zhang et al., 2025). Moreover, it has been demonstrated that ERK has a key role in regulating EMT in renal tubular epithelial cells (Juan et al., 2023). Researcher has demonstrated that activated ERK can mutate S360/S362A residues to enhance CK2-α activity (Ji et al., 2009). The present study indicated that ERK/CK2-α/β-catenin signaling pathway is involved in podocyte EMT, and SQ alleviates EMT-mediated podocyte apoptosis via the ERK/CK2-α/β-catenin pathway.

Actually,the major components of SQ have also been reported to target EMT-related molecules. Calycosin can target ERK-mediated apoptosis in H9c2 cells to reduce heat shock, but also directly regulate cell EMT to treat colorectal cancer (Wang et al., 2019; Lai et al., 2024). Moreover, caylcosin-7-O-β-D-glucopyranoside promotes osteoblast differentiation by modulating β-catenin (Jian et al., 2015). Notoginsenoside R1 can reduce heart damage caused by high altitude hypoxia in rats by activating ERK1/2, in addition to increase Lgr5^+^ stem cell and epithelial healing in colitis mice by stimulating Wnt/β-Catenin signaling (Zhao et al., 2023; Yu et al., 2024). Ginsenoside Re inhibits melanoma development by downregulating ERK and reduces EMT in non-small cell lung cancer cells by interfering with M2-like macrophage polarization (Hwang et al., 2023; Tang et al., 2024). Ginsenoside Rg1 regulates cell apoptosis and improves Alzheimer’s disease via the Wnt/GSK-3β/β-catenin signaling pathway (Yang et al., 2022). Ginsenoside Rb1 inhibits porcine epidemic diarrhea virus replication by suppressing the MAPK/ERK pathway and reducing cell apoptosis, and improves Bavachin induced renal fibrosis by inhibiting EMT (Ni et al., 2022; Zheng X. et al., 2025). Ginsenoside Rd promotes Differentiation of Myeloid Leukemia Cells via modulating the ERK/GSK-3β signaling pathway (Jiang et al., 2024). Ononin prevents angiogenesis by inhibiting the MEK/Erk signaling pathway (Gong et al., 2021). The research outcomes presented above support our findings.

This study elucidated EMT activation in apoptotic podocytes of IMN and the role of SQ in EMT activation, as well as performed a correlation analysis on the signaling pathways that mediate EMT activation utilizing previously published studies. However, the mechanisms underlying EMT and the role of SQ in it have not been thoroughly investigated. Specially, β-catenin is a crucial molecule for EMT activation in podocytes and also a vital molecule for tight adhesion of podocyte processes. The likely mechanism of higher β-catenin protein levels in podocytes in IMN is still unknown. All of these subjects should be investigated further in subsequent research.

## 5 Conclusion

In the present study, we identified SQ as a potential candidate for renoprotective effects in PHN rats by inhibiting podocyte apoptosis by reducing podocyte EMT. Moreover, SQ had an eliminative effect on podocyte EMT via the ERK/CK2-α/β-catenin signaling pathway. These data confirm that SQ is a promising therapeutic traditional Chinese medicine for IMN treatment.

## Data Availability

The original contributions presented in the study are included in the article/Supplementary Material, further inquiries can be directed to the corresponding authors.
