# Supplementary figures and images for: Sanqi oral solution alleviates podocyte apoptosis in experimental membranous nephropathy by mediating EMT through the ERK/CK2-α/β-catenin pathway (part 2 of 4)
Source: Front Pharmacol. 2025 May 9;16:1503961. doi: 10.3389/fphar.2025.1503961 (PMC12098599; doi:10.3389/fphar.2025.1503961)

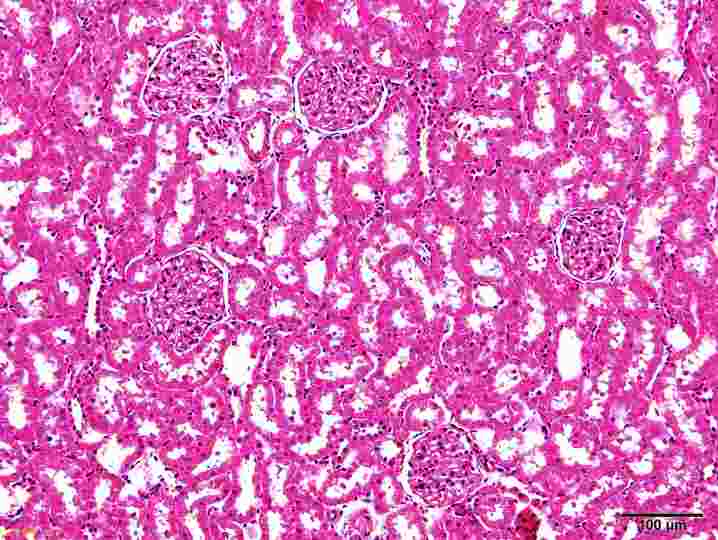

Supplement: Supplementary file 1 [file DataSheet1.zip › Original images and results for Figure 2/Fig. 2H/Fig. 2H-HE/HE-SQ-H/6-1.jpg]

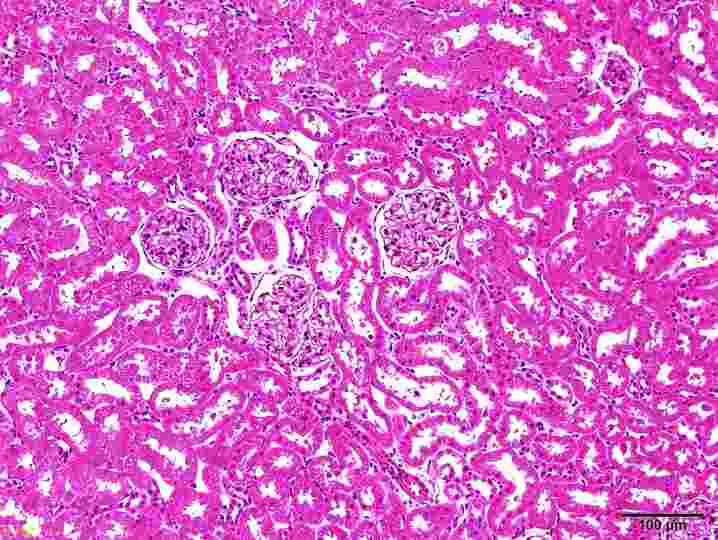

Supplement: Supplementary file 1 [file DataSheet1.zip › Original images and results for Figure 2/Fig. 2H/Fig. 2H-HE/HE-SQ-H/6-3.jpg]

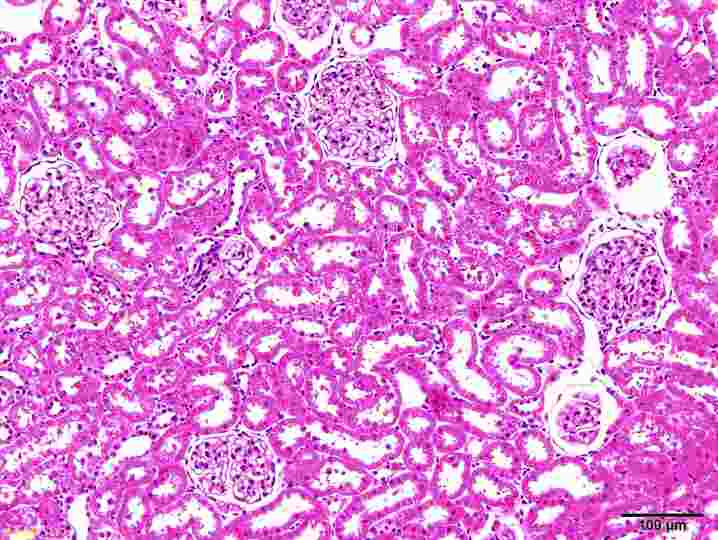

Supplement: Supplementary file 1 [file DataSheet1.zip › Original images and results for Figure 2/Fig. 2H/Fig. 2H-HE/HE-SQ-H/6-4.jpg]

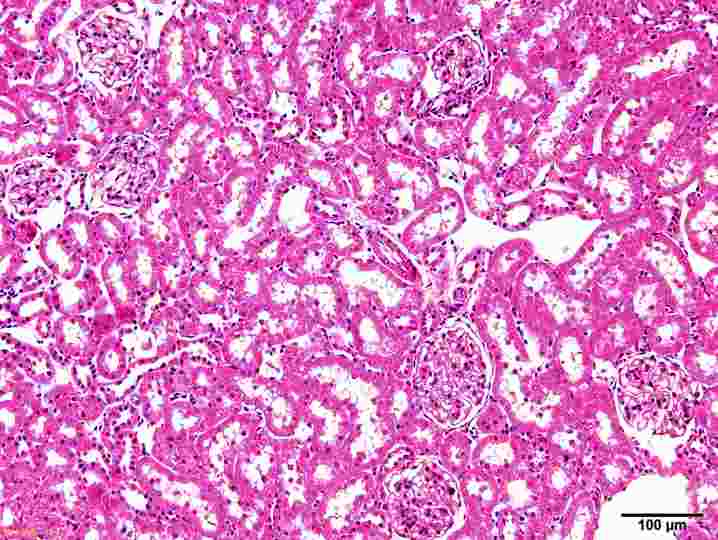

Supplement: Supplementary file 1 [file DataSheet1.zip › Original images and results for Figure 2/Fig. 2H/Fig. 2H-HE/HE-SQ-L/1-1.jpg]

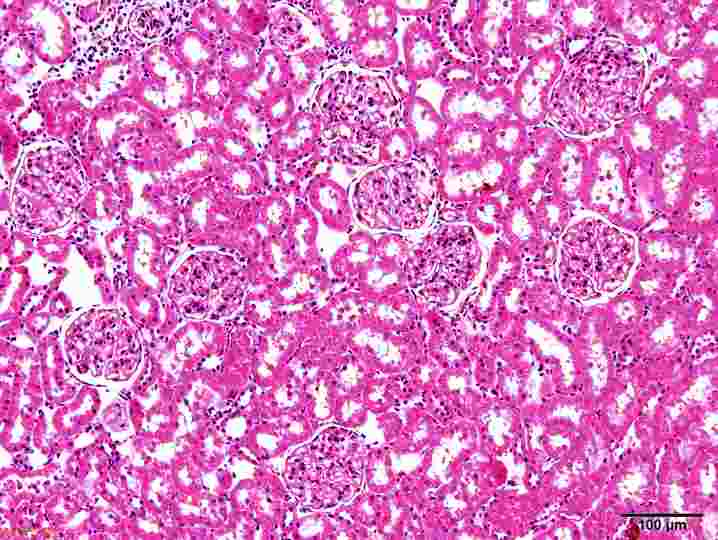

Supplement: Supplementary file 1 [file DataSheet1.zip › Original images and results for Figure 2/Fig. 2H/Fig. 2H-HE/HE-SQ-L/1-2.jpg]

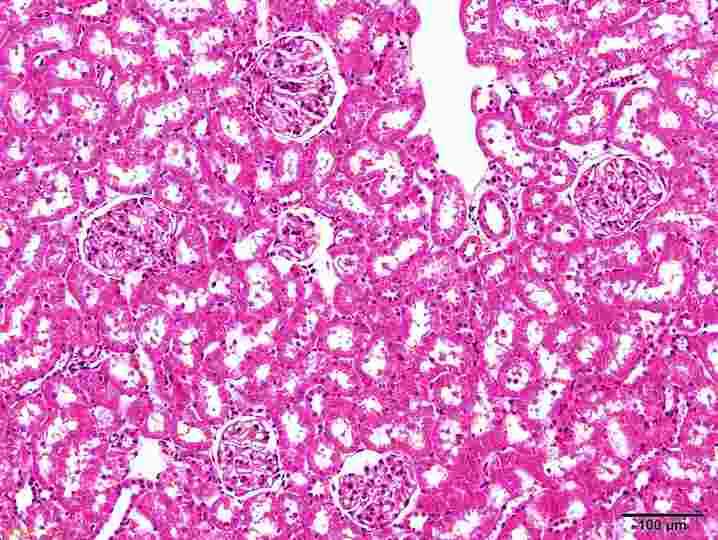

Supplement: Supplementary file 1 [file DataSheet1.zip › Original images and results for Figure 2/Fig. 2H/Fig. 2H-HE/HE-SQ-L/1-3.jpg]

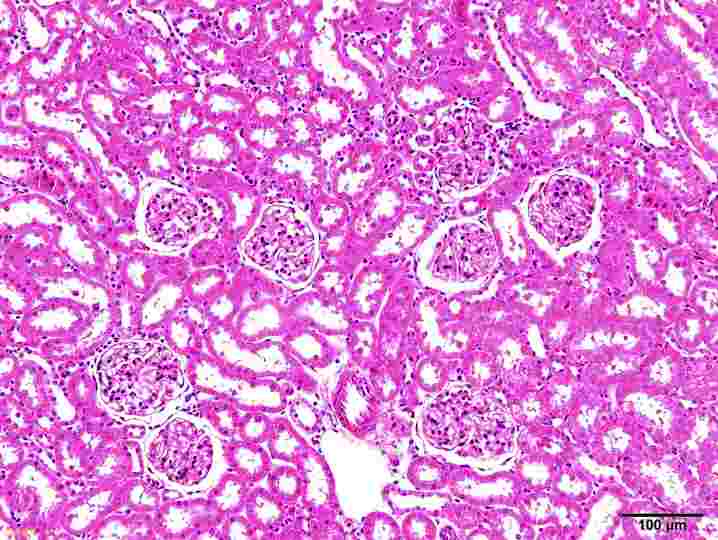

Supplement: Supplementary file 1 [file DataSheet1.zip › Original images and results for Figure 2/Fig. 2H/Fig. 2H-HE/HE-SQ-L/1-5.jpg]

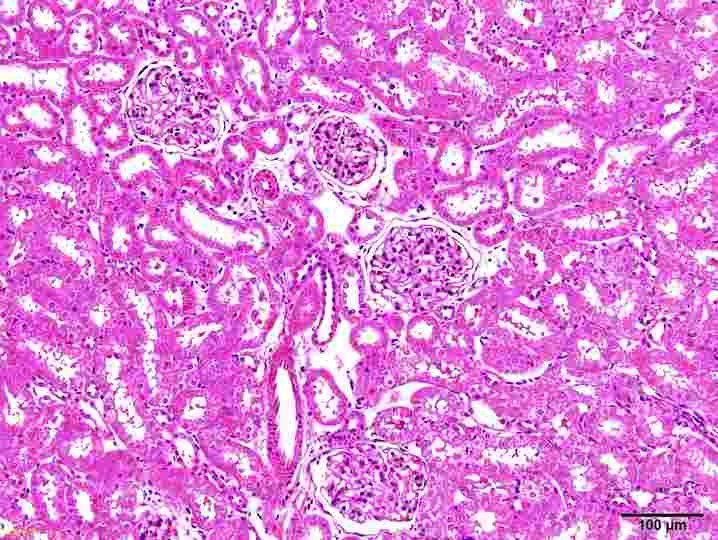

Supplement: Supplementary file 1 [file DataSheet1.zip › Original images and results for Figure 2/Fig. 2H/Fig. 2H-HE/HE-SQ-L/2-1.jpg]

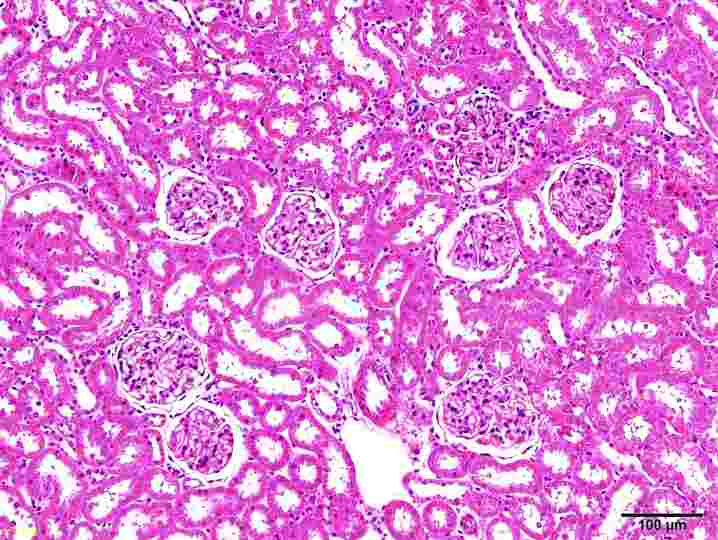

Supplement: Supplementary file 1 [file DataSheet1.zip › Original images and results for Figure 2/Fig. 2H/Fig. 2H-HE/HE-SQ-L/2-2 image in Fig. 1H-HE.jpg]

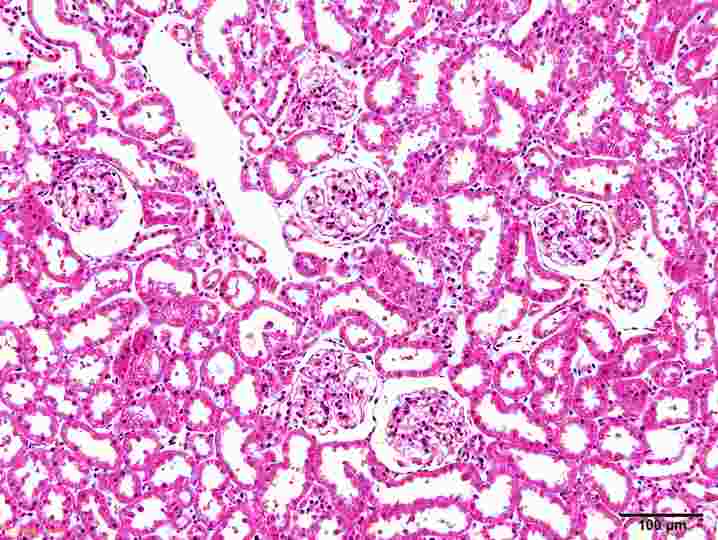

Supplement: Supplementary file 1 [file DataSheet1.zip › Original images and results for Figure 2/Fig. 2H/Fig. 2H-HE/HE-SQ-L/2-5.jpg]

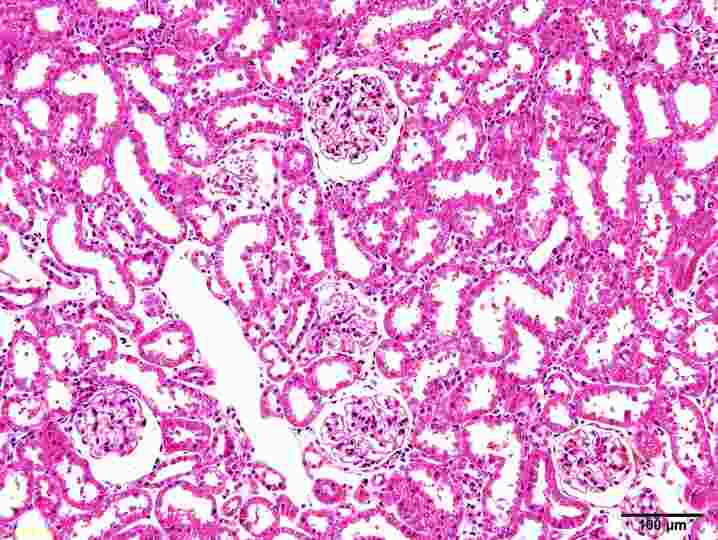

Supplement: Supplementary file 1 [file DataSheet1.zip › Original images and results for Figure 2/Fig. 2H/Fig. 2H-HE/HE-SQ-L/3-1.jpg]

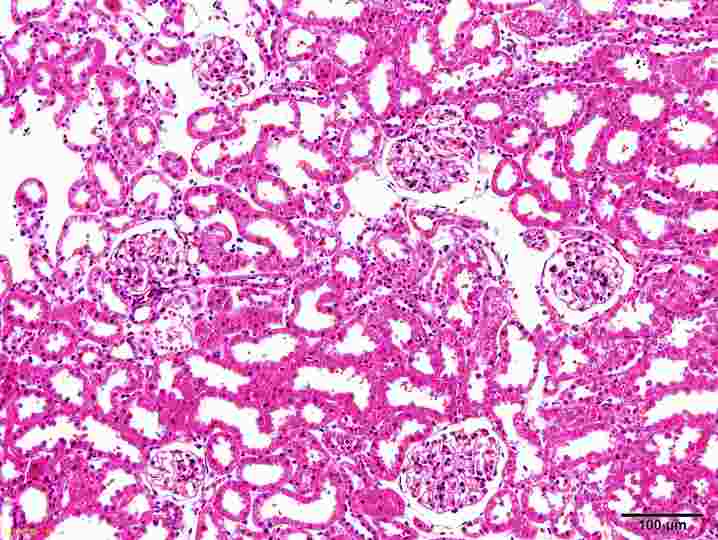

Supplement: Supplementary file 1 [file DataSheet1.zip › Original images and results for Figure 2/Fig. 2H/Fig. 2H-HE/HE-SQ-L/3-2.jpg]

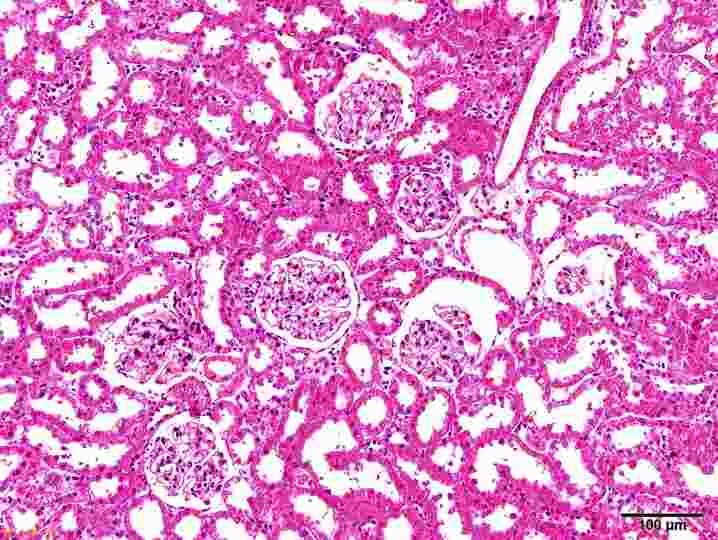

Supplement: Supplementary file 1 [file DataSheet1.zip › Original images and results for Figure 2/Fig. 2H/Fig. 2H-HE/HE-SQ-L/3-3.jpg]

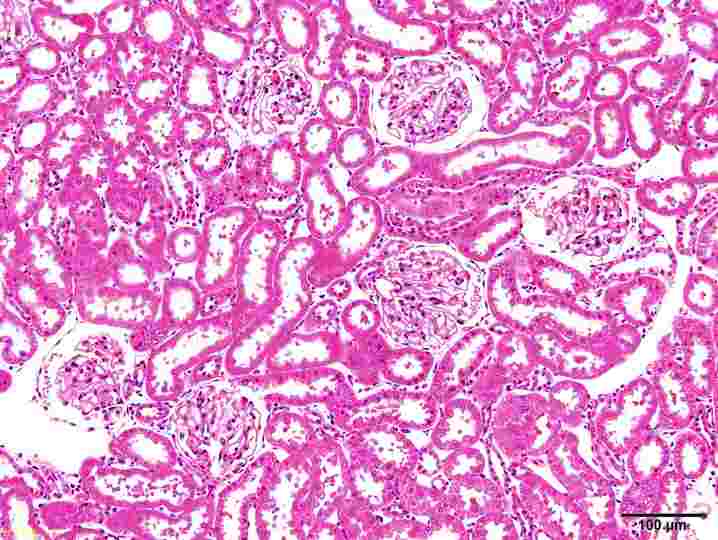

Supplement: Supplementary file 1 [file DataSheet1.zip › Original images and results for Figure 2/Fig. 2H/Fig. 2H-HE/HE-SQ-L/3-4.jpg]

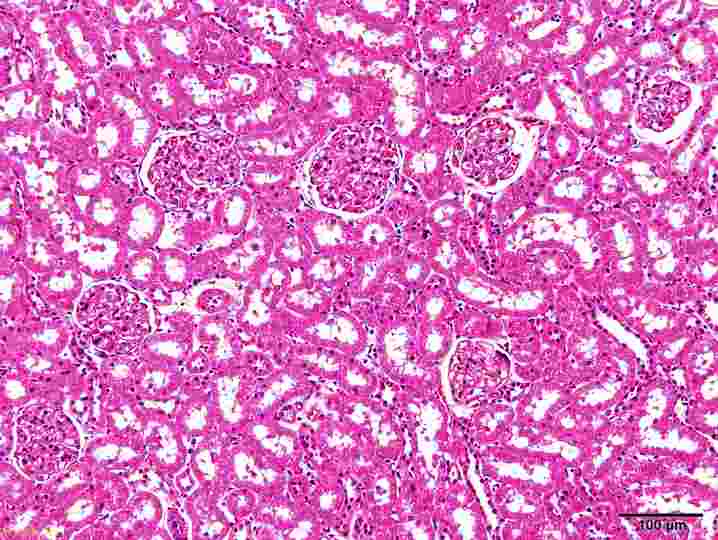

Supplement: Supplementary file 1 [file DataSheet1.zip › Original images and results for Figure 2/Fig. 2H/Fig. 2H-HE/HE-SQ-L/3-5.jpg]

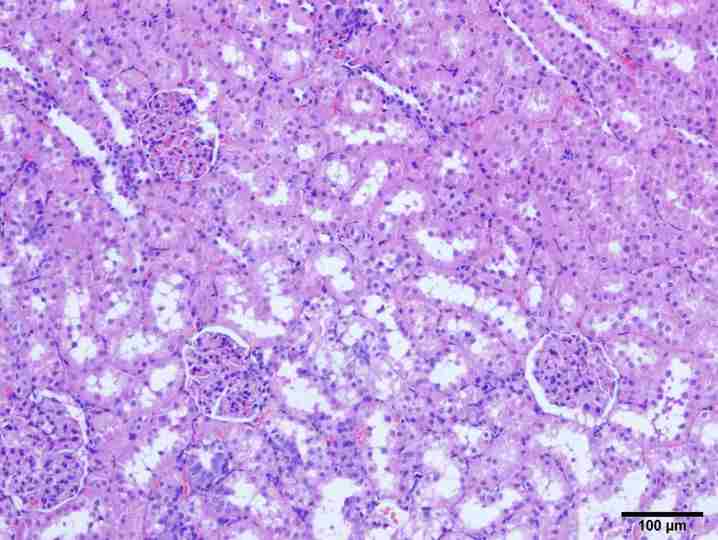

Supplement: Supplementary file 1 [file DataSheet1.zip › Original images and results for Figure 2/Fig. 2H/Fig. 2H-HE/HE-SQ-L/4-1.jpg]

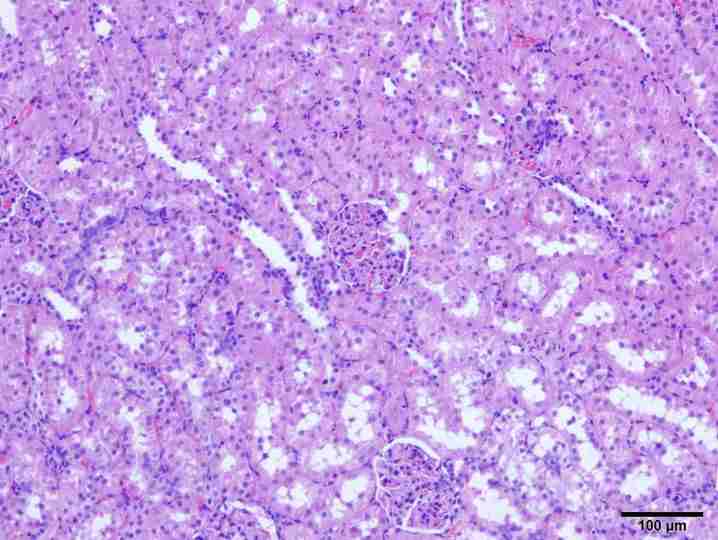

Supplement: Supplementary file 1 [file DataSheet1.zip › Original images and results for Figure 2/Fig. 2H/Fig. 2H-HE/HE-SQ-L/4-2.jpg]

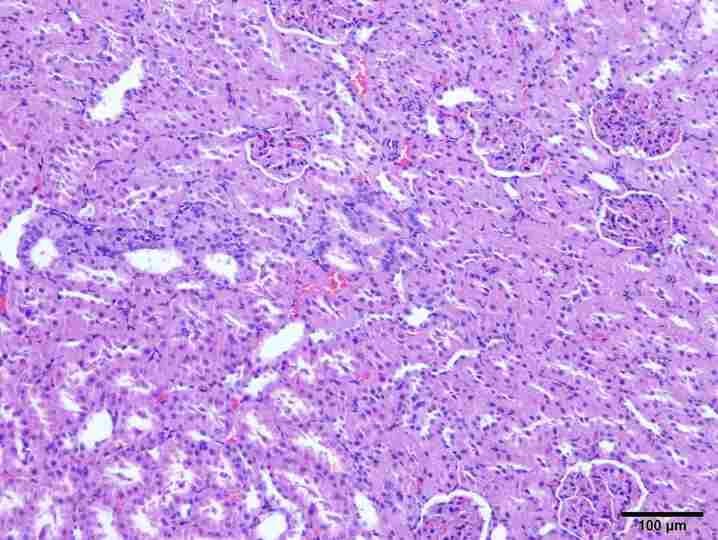

Supplement: Supplementary file 1 [file DataSheet1.zip › Original images and results for Figure 2/Fig. 2H/Fig. 2H-HE/HE-SQ-L/4-3.jpg]

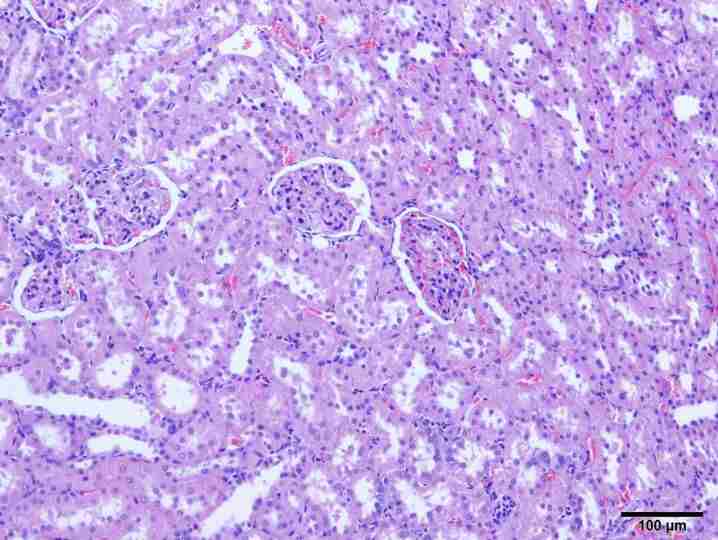

Supplement: Supplementary file 1 [file DataSheet1.zip › Original images and results for Figure 2/Fig. 2H/Fig. 2H-HE/HE-SQ-L/4-4.jpg]

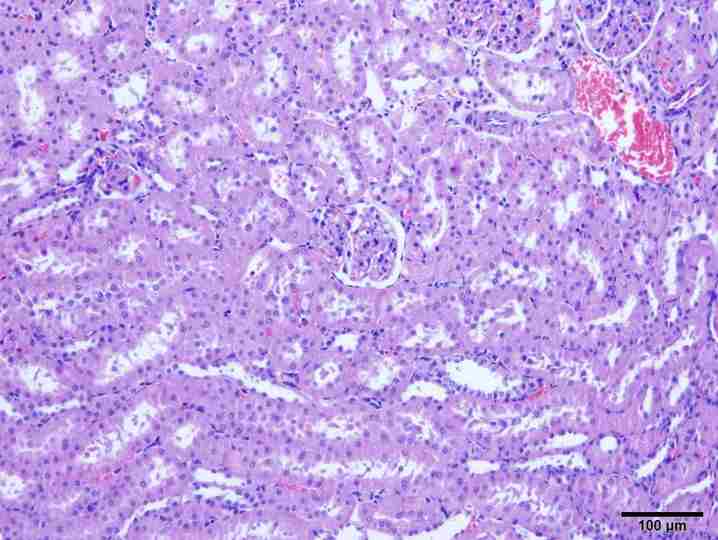

Supplement: Supplementary file 1 [file DataSheet1.zip › Original images and results for Figure 2/Fig. 2H/Fig. 2H-HE/HE-SQ-L/4-5.jpg]

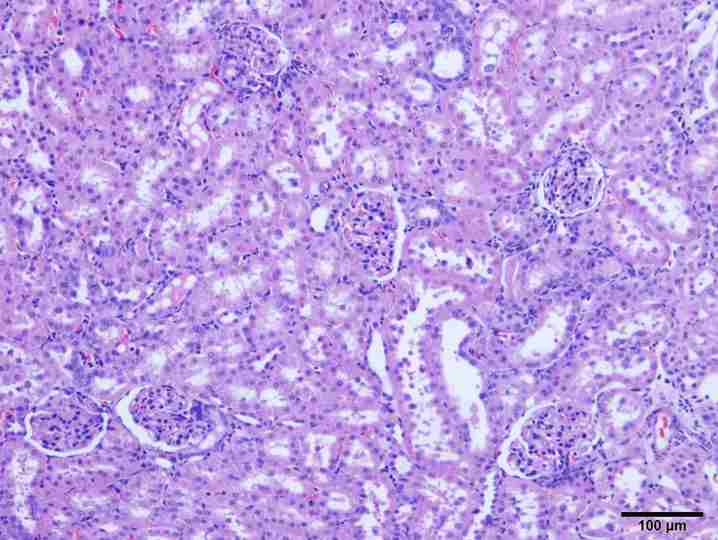

Supplement: Supplementary file 1 [file DataSheet1.zip › Original images and results for Figure 2/Fig. 2H/Fig. 2H-HE/HE-SQ-L/5-1.jpg]

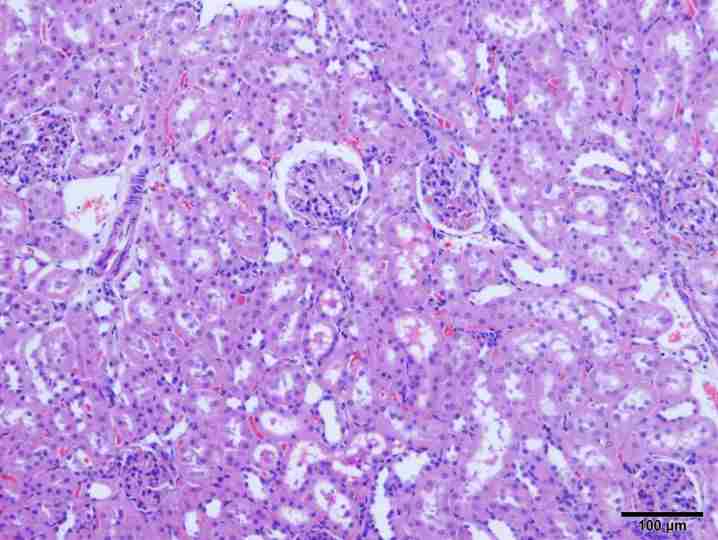

Supplement: Supplementary file 1 [file DataSheet1.zip › Original images and results for Figure 2/Fig. 2H/Fig. 2H-HE/HE-SQ-L/5-2.jpg]

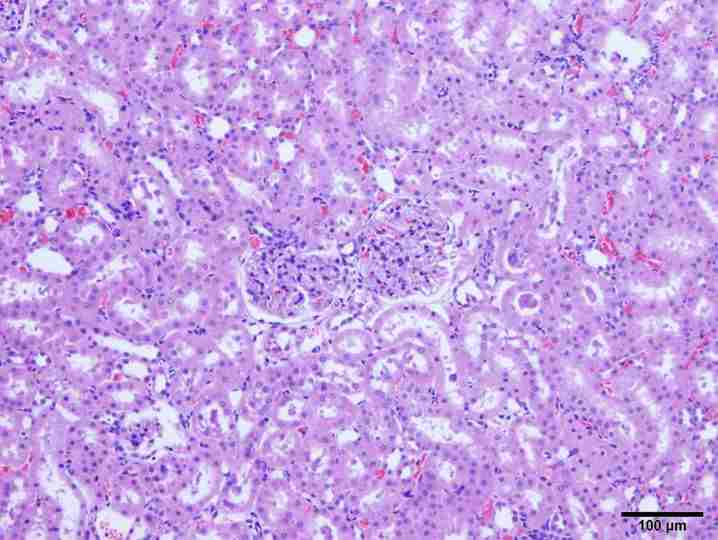

Supplement: Supplementary file 1 [file DataSheet1.zip › Original images and results for Figure 2/Fig. 2H/Fig. 2H-HE/HE-SQ-L/5-3.jpg]

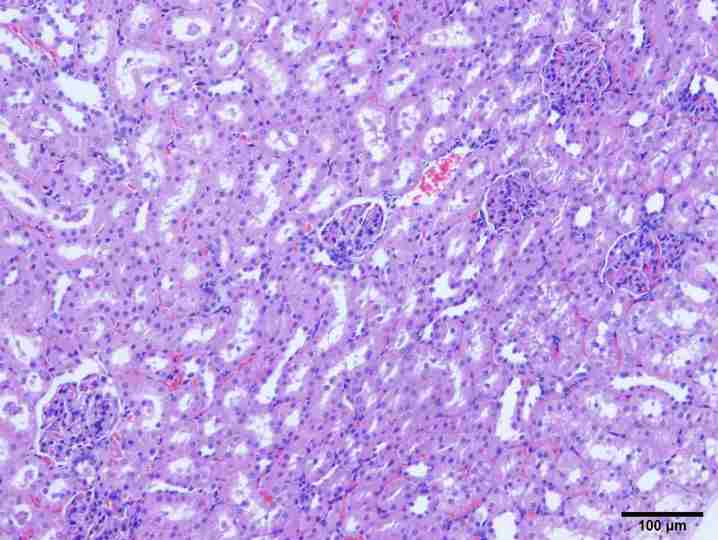

Supplement: Supplementary file 1 [file DataSheet1.zip › Original images and results for Figure 2/Fig. 2H/Fig. 2H-HE/HE-SQ-L/5-4.jpg]

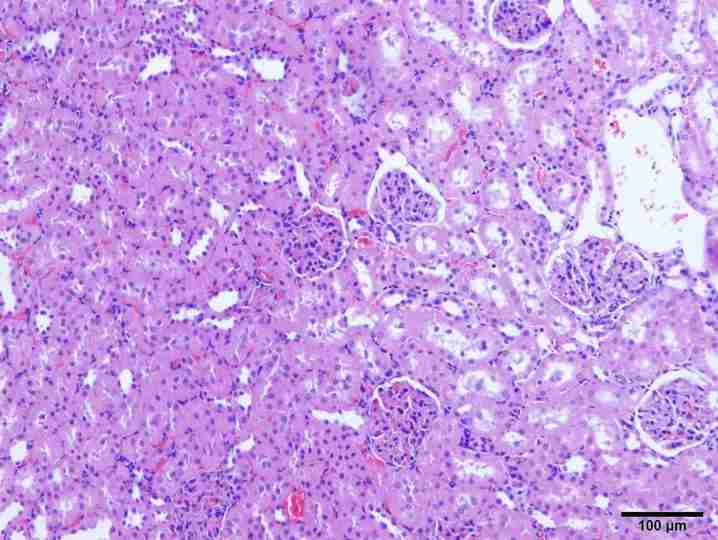

Supplement: Supplementary file 1 [file DataSheet1.zip › Original images and results for Figure 2/Fig. 2H/Fig. 2H-HE/HE-SQ-L/5-5.jpg]

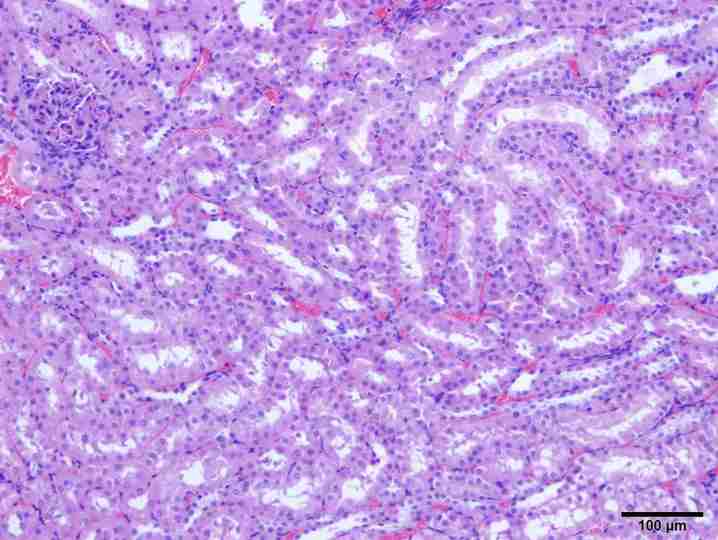

Supplement: Supplementary file 1 [file DataSheet1.zip › Original images and results for Figure 2/Fig. 2H/Fig. 2H-HE/HE-SQ-L/6-1.jpg]

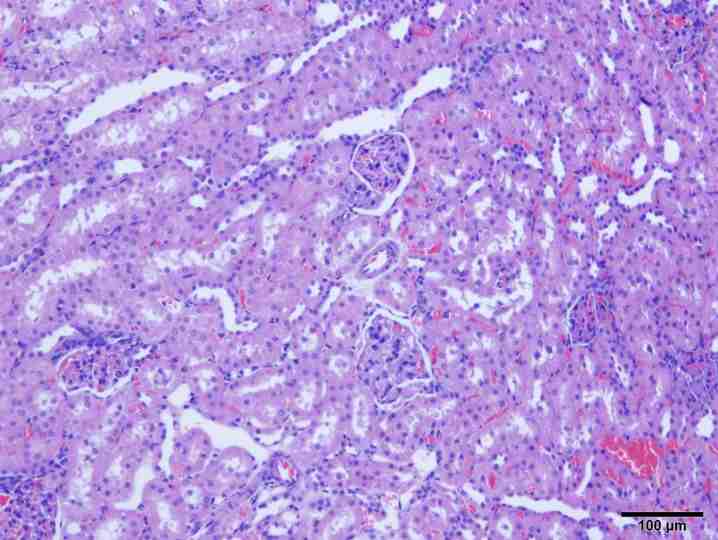

Supplement: Supplementary file 1 [file DataSheet1.zip › Original images and results for Figure 2/Fig. 2H/Fig. 2H-HE/HE-SQ-L/6-2.jpg]

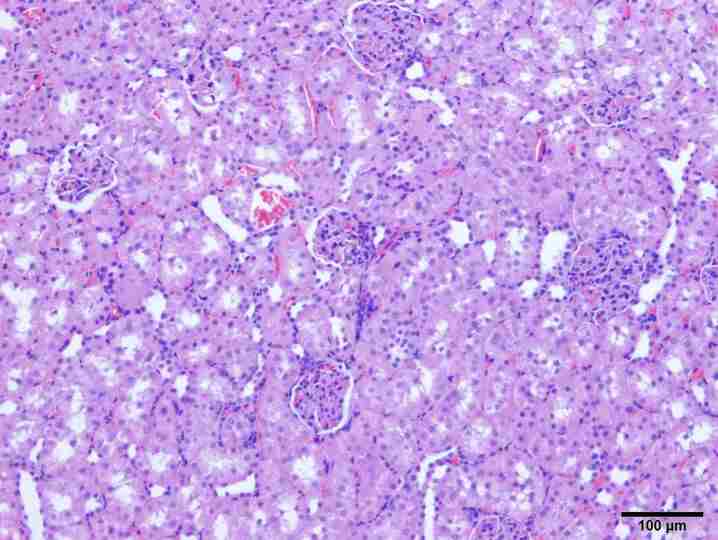

Supplement: Supplementary file 1 [file DataSheet1.zip › Original images and results for Figure 2/Fig. 2H/Fig. 2H-HE/HE-SQ-L/6-3.jpg]

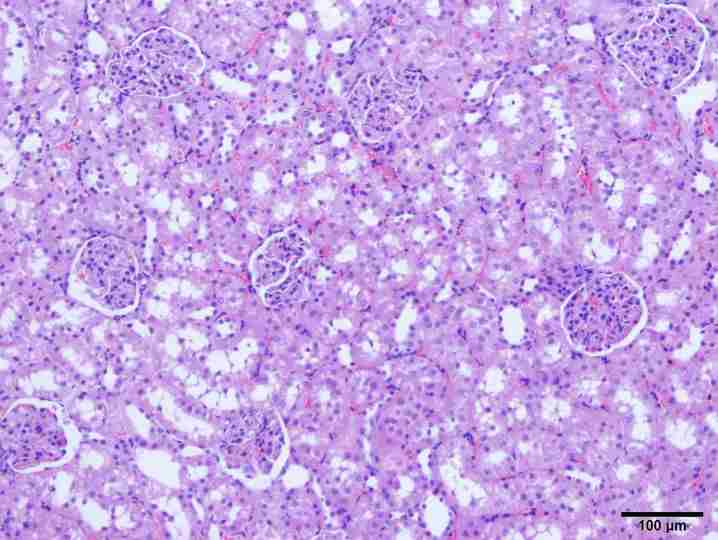

Supplement: Supplementary file 1 [file DataSheet1.zip › Original images and results for Figure 2/Fig. 2H/Fig. 2H-HE/HE-SQ-L/6-4.jpg]

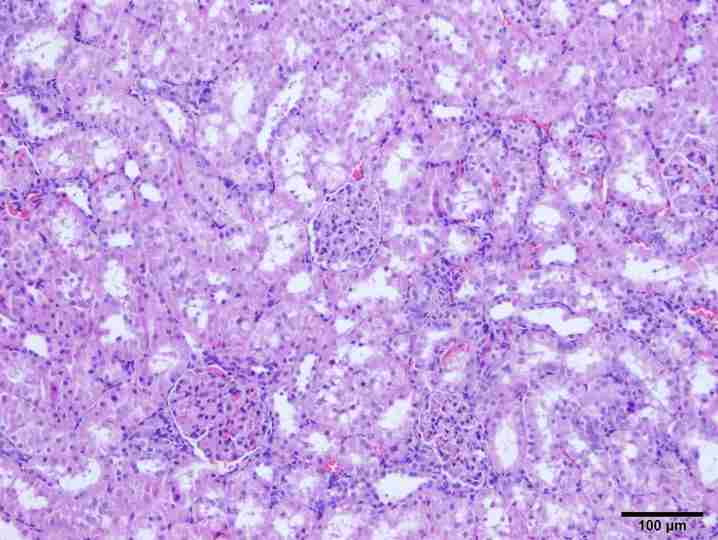

Supplement: Supplementary file 1 [file DataSheet1.zip › Original images and results for Figure 2/Fig. 2H/Fig. 2H-HE/HE-SQ-L/6-5.jpg]

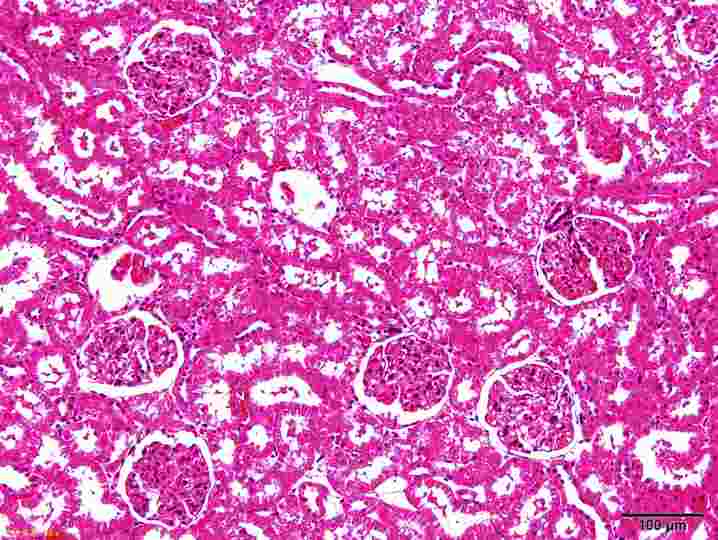

Supplement: Supplementary file 1 [file DataSheet1.zip › Original images and results for Figure 2/Fig. 2H/Fig. 2H-HE/HE-TAC/1-1.jpg]

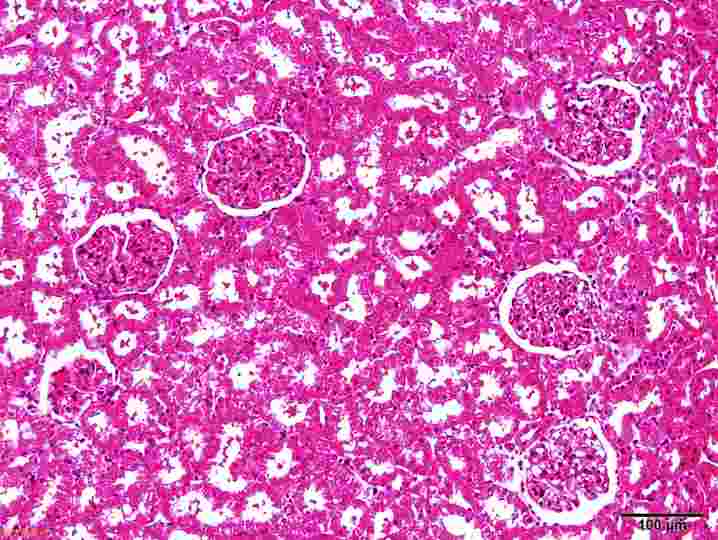

Supplement: Supplementary file 1 [file DataSheet1.zip › Original images and results for Figure 2/Fig. 2H/Fig. 2H-HE/HE-TAC/1-2.jpg]

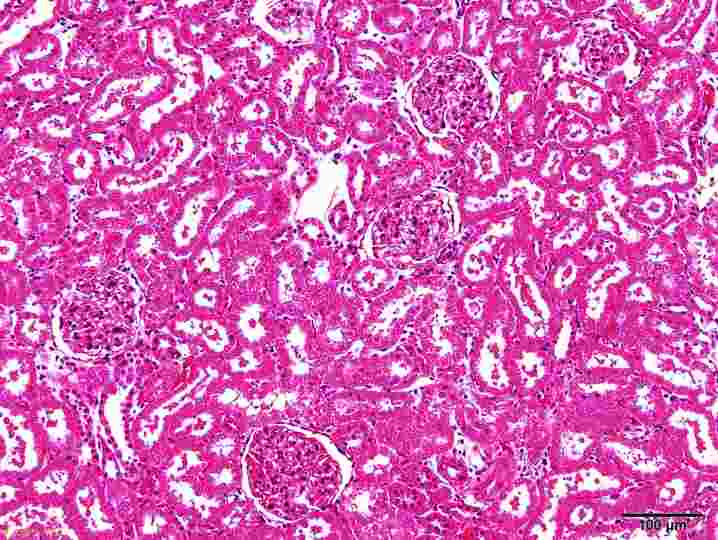

Supplement: Supplementary file 1 [file DataSheet1.zip › Original images and results for Figure 2/Fig. 2H/Fig. 2H-HE/HE-TAC/1-3.jpg]

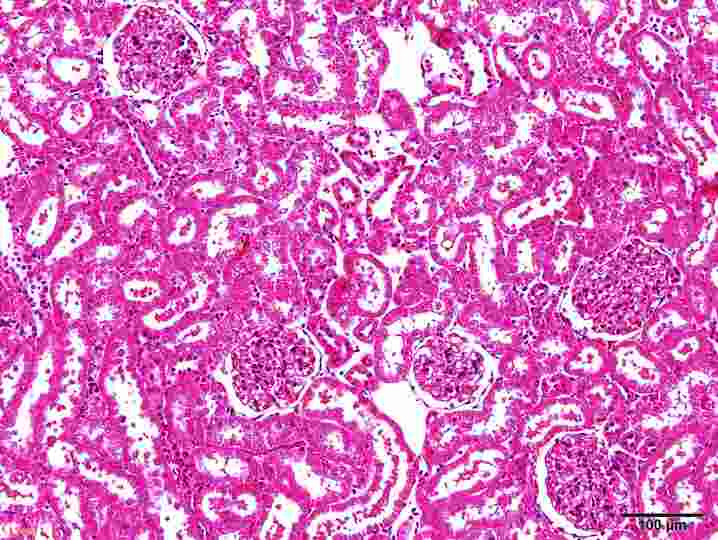

Supplement: Supplementary file 1 [file DataSheet1.zip › Original images and results for Figure 2/Fig. 2H/Fig. 2H-HE/HE-TAC/1-4.jpg]

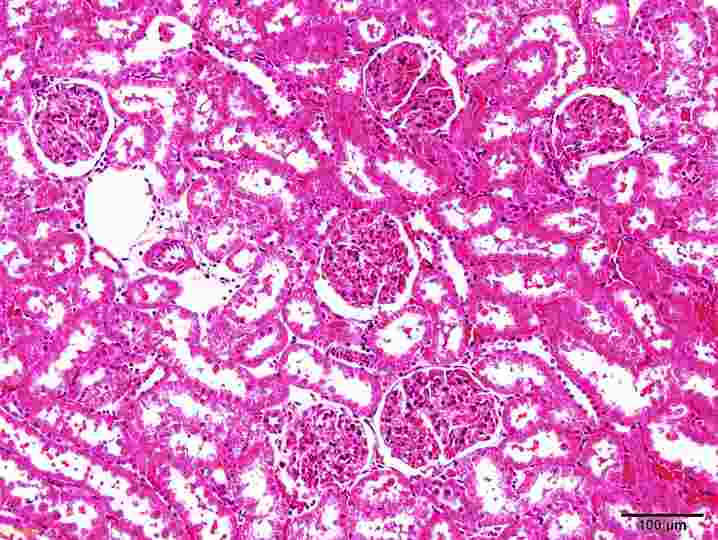

Supplement: Supplementary file 1 [file DataSheet1.zip › Original images and results for Figure 2/Fig. 2H/Fig. 2H-HE/HE-TAC/1-5.jpg]

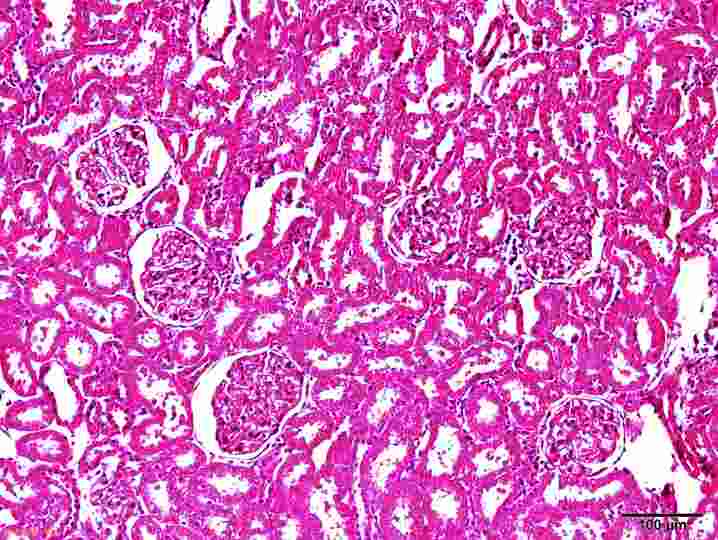

Supplement: Supplementary file 1 [file DataSheet1.zip › Original images and results for Figure 2/Fig. 2H/Fig. 2H-HE/HE-TAC/2-1.jpg]

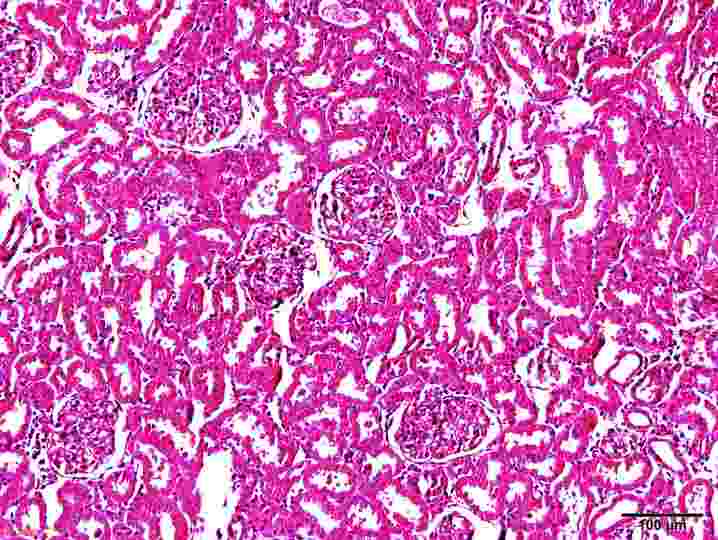

Supplement: Supplementary file 1 [file DataSheet1.zip › Original images and results for Figure 2/Fig. 2H/Fig. 2H-HE/HE-TAC/2-2.jpg]

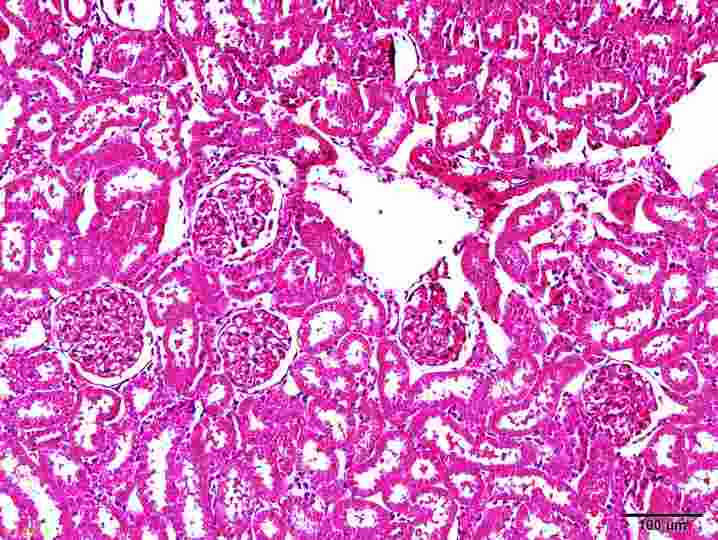

Supplement: Supplementary file 1 [file DataSheet1.zip › Original images and results for Figure 2/Fig. 2H/Fig. 2H-HE/HE-TAC/2-3.jpg]

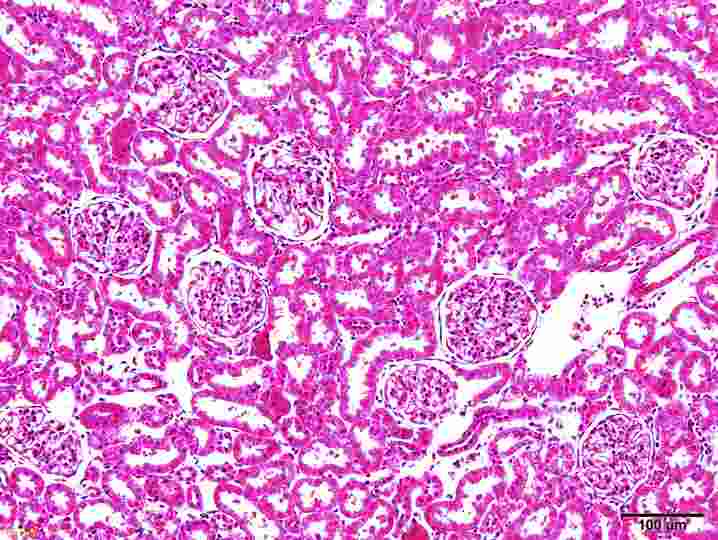

Supplement: Supplementary file 1 [file DataSheet1.zip › Original images and results for Figure 2/Fig. 2H/Fig. 2H-HE/HE-TAC/2-4.jpg]

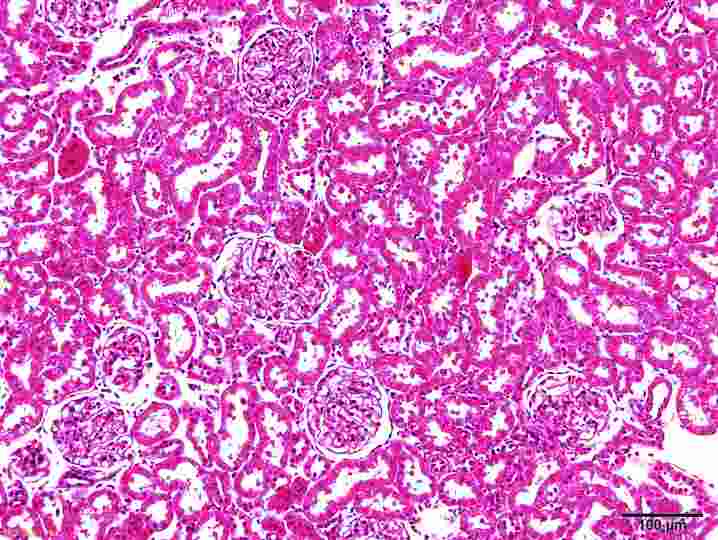

Supplement: Supplementary file 1 [file DataSheet1.zip › Original images and results for Figure 2/Fig. 2H/Fig. 2H-HE/HE-TAC/2-5.jpg]

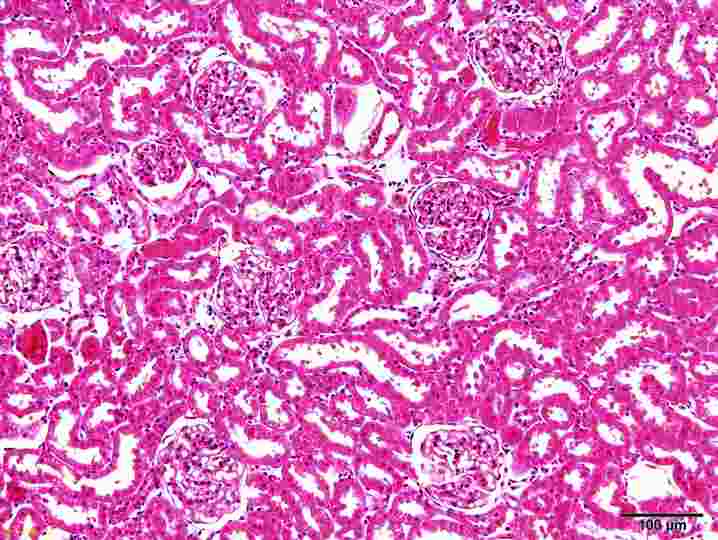

Supplement: Supplementary file 1 [file DataSheet1.zip › Original images and results for Figure 2/Fig. 2H/Fig. 2H-HE/HE-TAC/3-1.jpg]

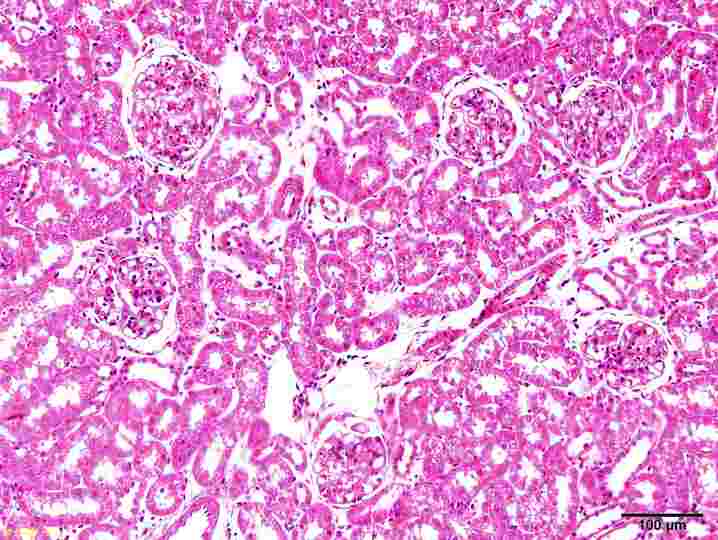

Supplement: Supplementary file 1 [file DataSheet1.zip › Original images and results for Figure 2/Fig. 2H/Fig. 2H-HE/HE-TAC/3-2.jpg]

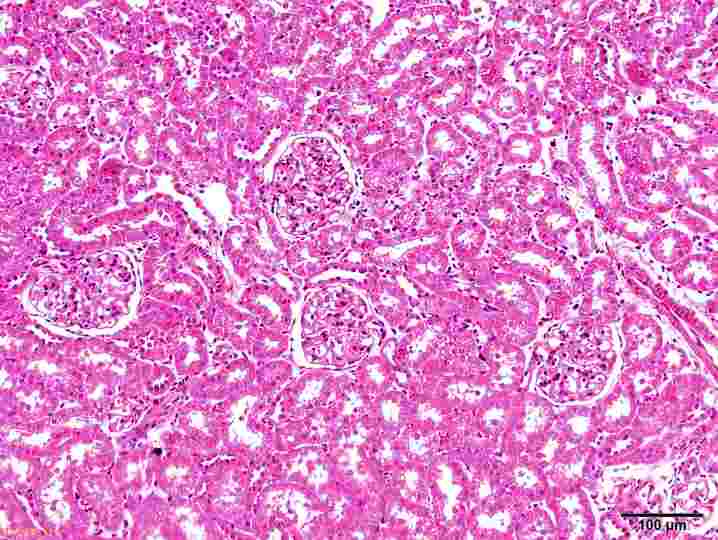

Supplement: Supplementary file 1 [file DataSheet1.zip › Original images and results for Figure 2/Fig. 2H/Fig. 2H-HE/HE-TAC/3-3 image in Fig. 1H-HE.jpg]

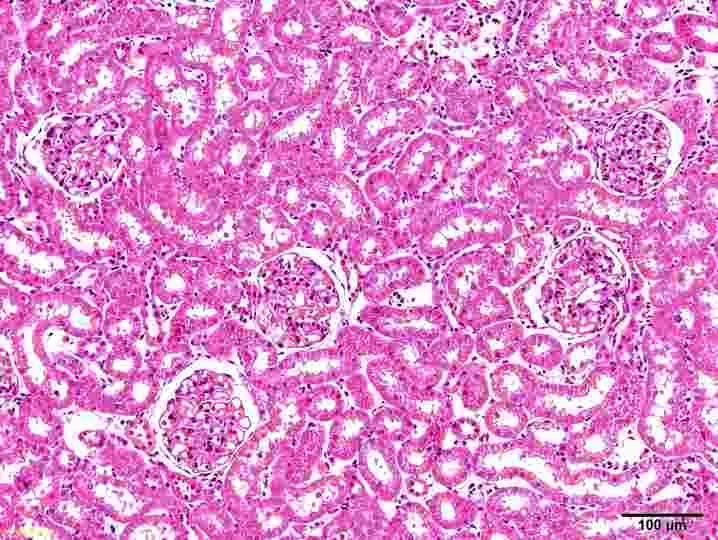

Supplement: Supplementary file 1 [file DataSheet1.zip › Original images and results for Figure 2/Fig. 2H/Fig. 2H-HE/HE-TAC/3-4.jpg]

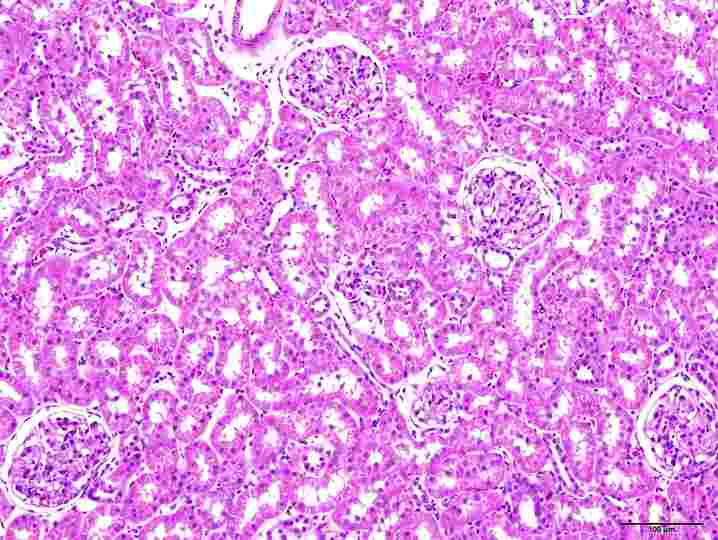

Supplement: Supplementary file 1 [file DataSheet1.zip › Original images and results for Figure 2/Fig. 2H/Fig. 2H-HE/HE-TAC/3-5.jpg]

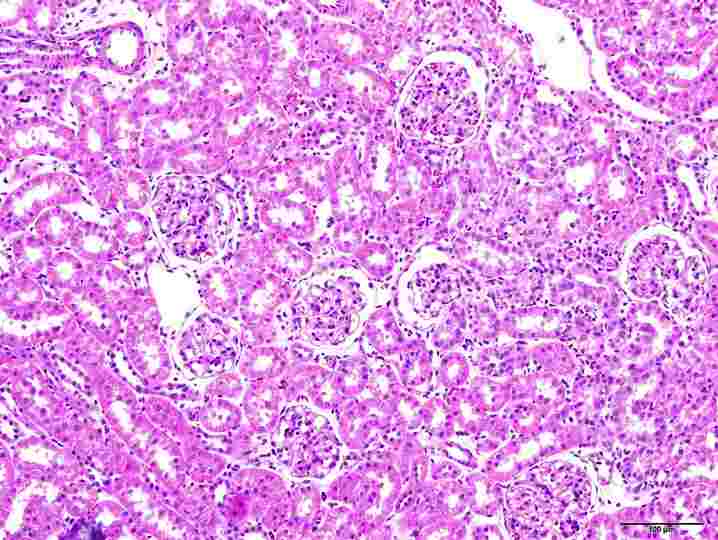

Supplement: Supplementary file 1 [file DataSheet1.zip › Original images and results for Figure 2/Fig. 2H/Fig. 2H-HE/HE-TAC/4-1.jpg]

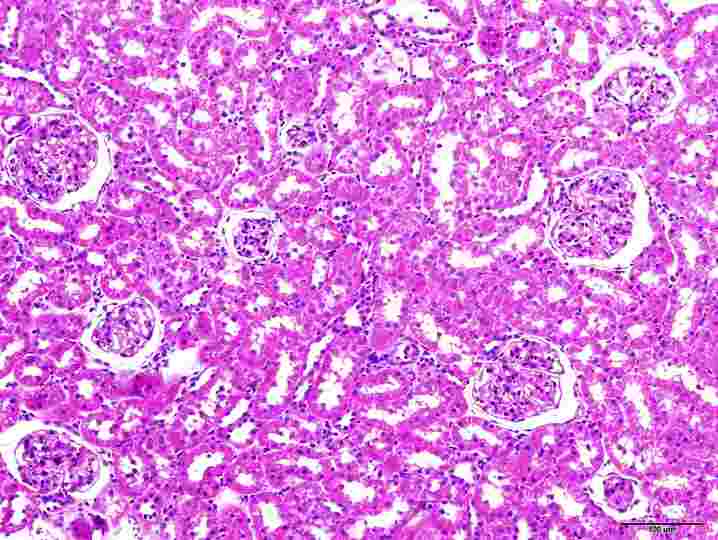

Supplement: Supplementary file 1 [file DataSheet1.zip › Original images and results for Figure 2/Fig. 2H/Fig. 2H-HE/HE-TAC/4-2.jpg]

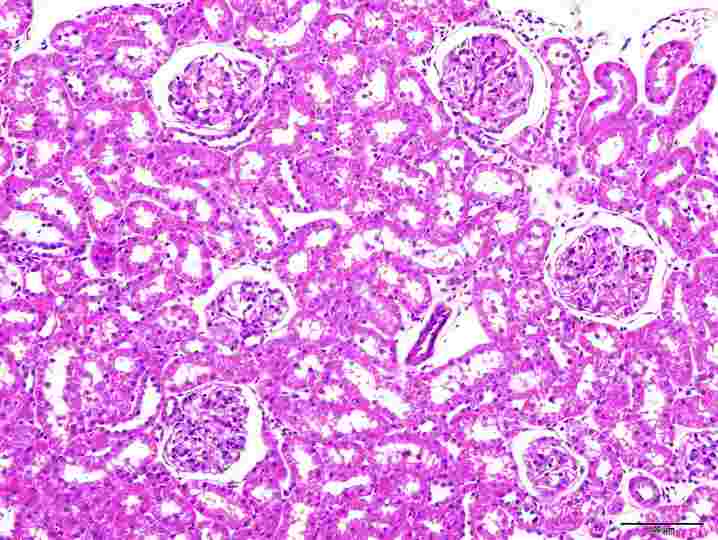

Supplement: Supplementary file 1 [file DataSheet1.zip › Original images and results for Figure 2/Fig. 2H/Fig. 2H-HE/HE-TAC/4-3.jpg]

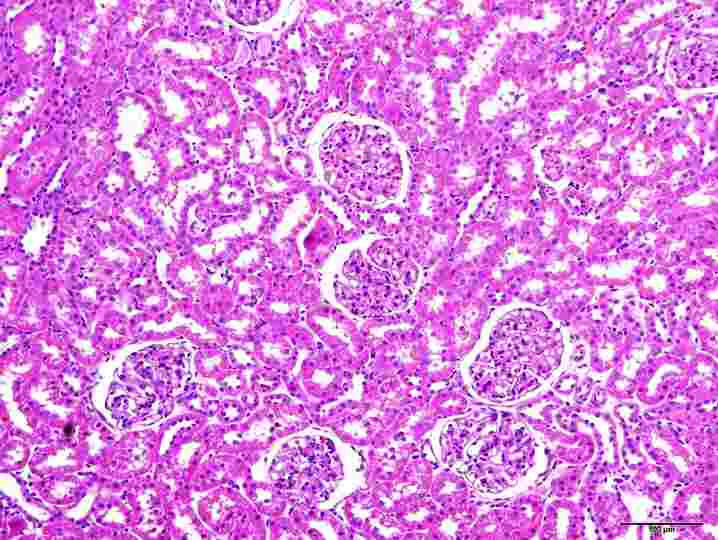

Supplement: Supplementary file 1 [file DataSheet1.zip › Original images and results for Figure 2/Fig. 2H/Fig. 2H-HE/HE-TAC/4-4.jpg]

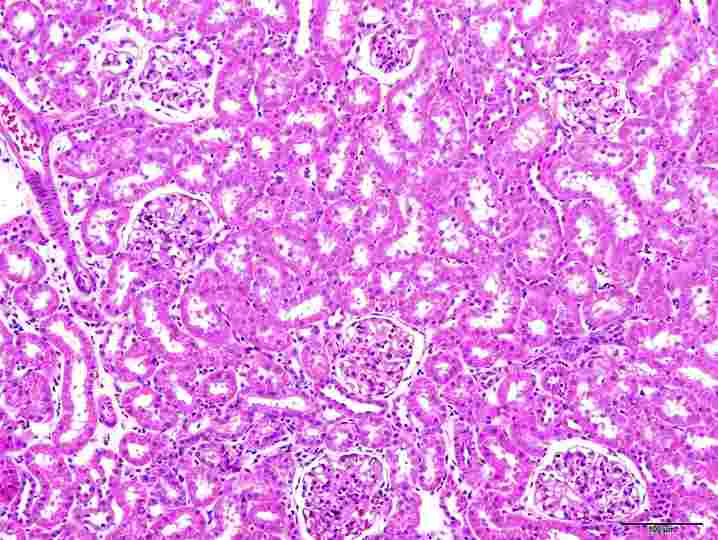

Supplement: Supplementary file 1 [file DataSheet1.zip › Original images and results for Figure 2/Fig. 2H/Fig. 2H-HE/HE-TAC/4-5.jpg]

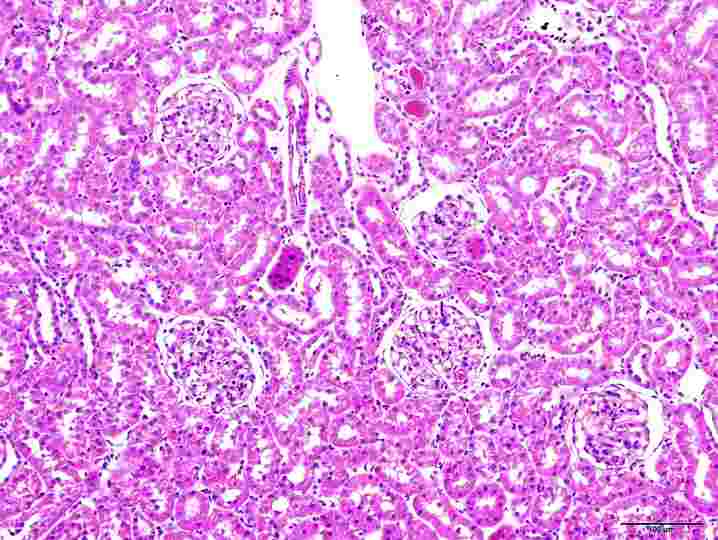

Supplement: Supplementary file 1 [file DataSheet1.zip › Original images and results for Figure 2/Fig. 2H/Fig. 2H-HE/HE-TAC/5-1.jpg]

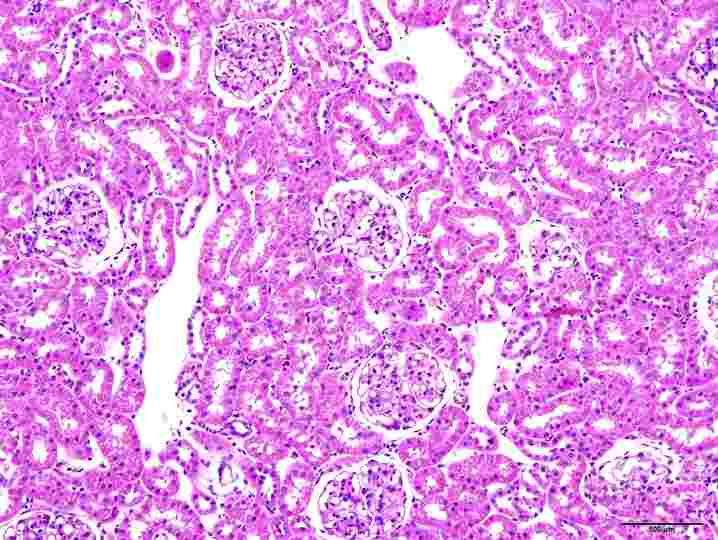

Supplement: Supplementary file 1 [file DataSheet1.zip › Original images and results for Figure 2/Fig. 2H/Fig. 2H-HE/HE-TAC/5-2.jpg]

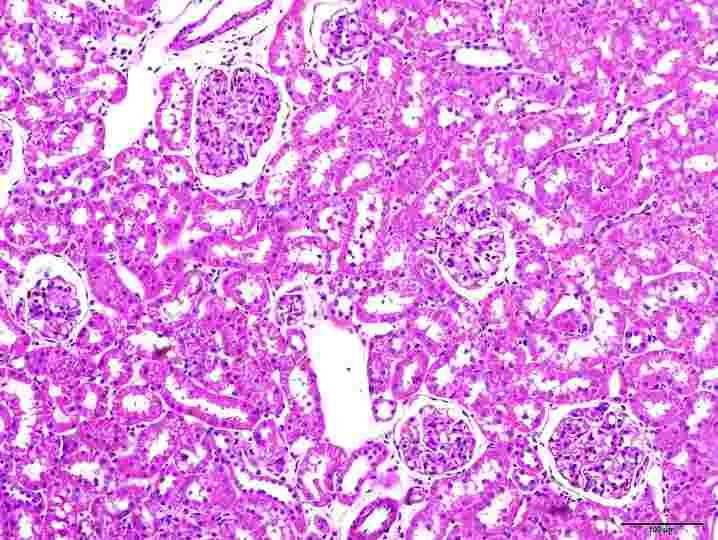

Supplement: Supplementary file 1 [file DataSheet1.zip › Original images and results for Figure 2/Fig. 2H/Fig. 2H-HE/HE-TAC/5-3.jpg]

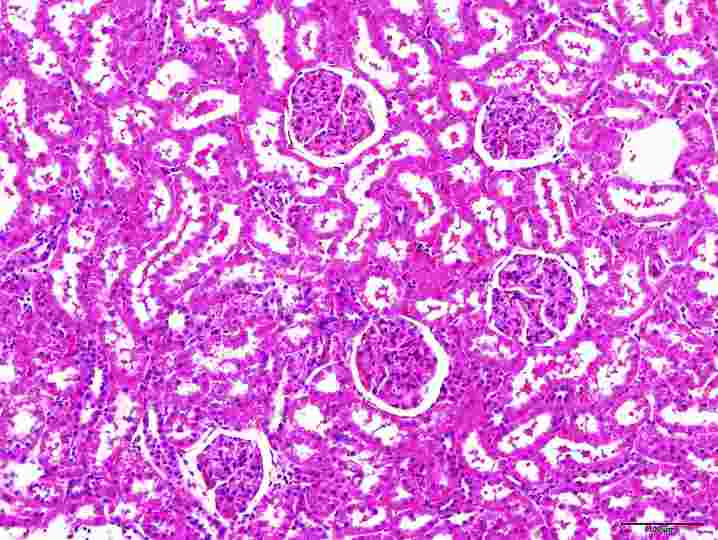

Supplement: Supplementary file 1 [file DataSheet1.zip › Original images and results for Figure 2/Fig. 2H/Fig. 2H-HE/HE-TAC/5-4.jpg]

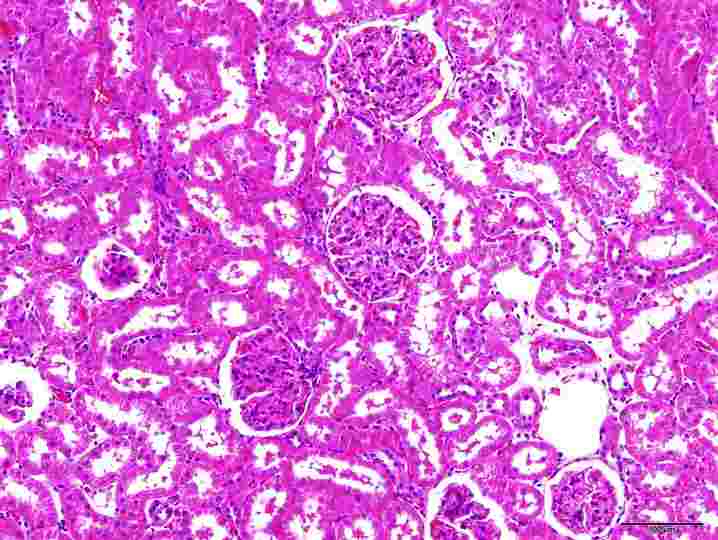

Supplement: Supplementary file 1 [file DataSheet1.zip › Original images and results for Figure 2/Fig. 2H/Fig. 2H-HE/HE-TAC/5-5.jpg]

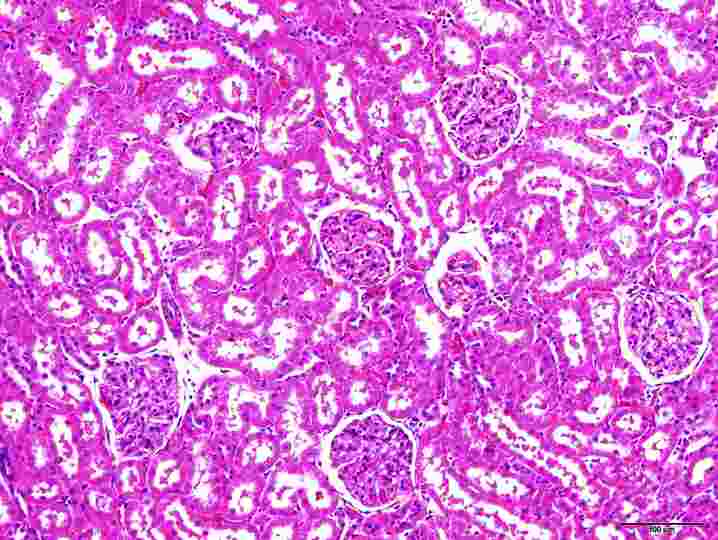

Supplement: Supplementary file 1 [file DataSheet1.zip › Original images and results for Figure 2/Fig. 2H/Fig. 2H-HE/HE-TAC/6-1.jpg]

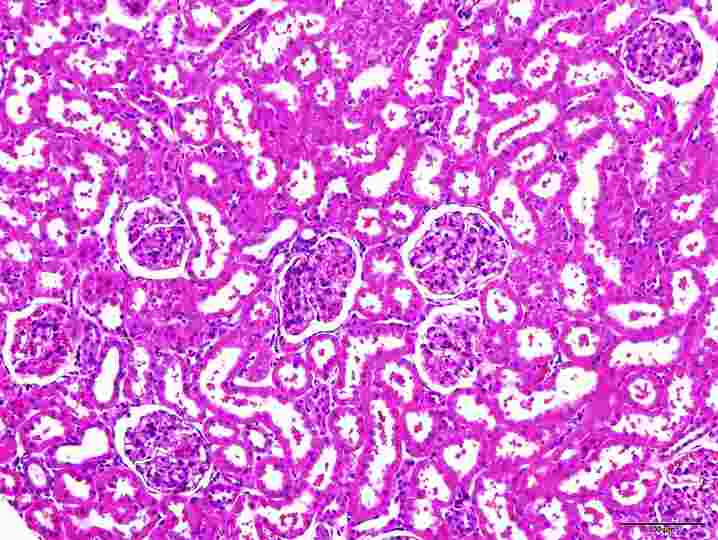

Supplement: Supplementary file 1 [file DataSheet1.zip › Original images and results for Figure 2/Fig. 2H/Fig. 2H-HE/HE-TAC/6-2.jpg]

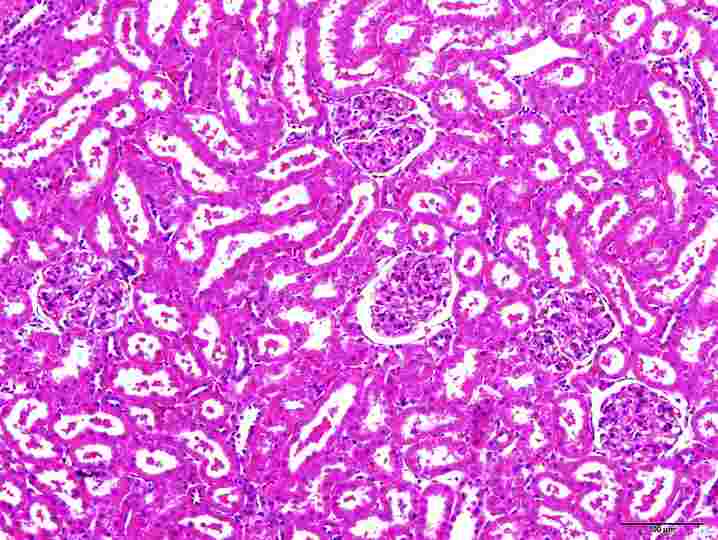

Supplement: Supplementary file 1 [file DataSheet1.zip › Original images and results for Figure 2/Fig. 2H/Fig. 2H-HE/HE-TAC/6-3.jpg]

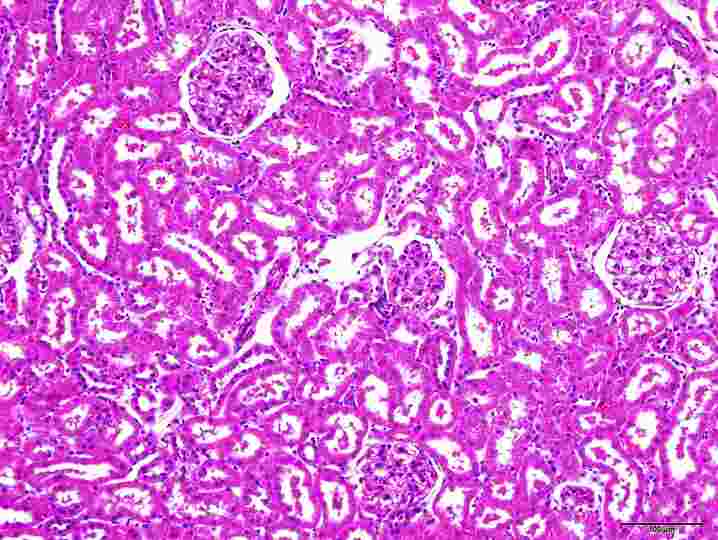

Supplement: Supplementary file 1 [file DataSheet1.zip › Original images and results for Figure 2/Fig. 2H/Fig. 2H-HE/HE-TAC/6-4.jpg]

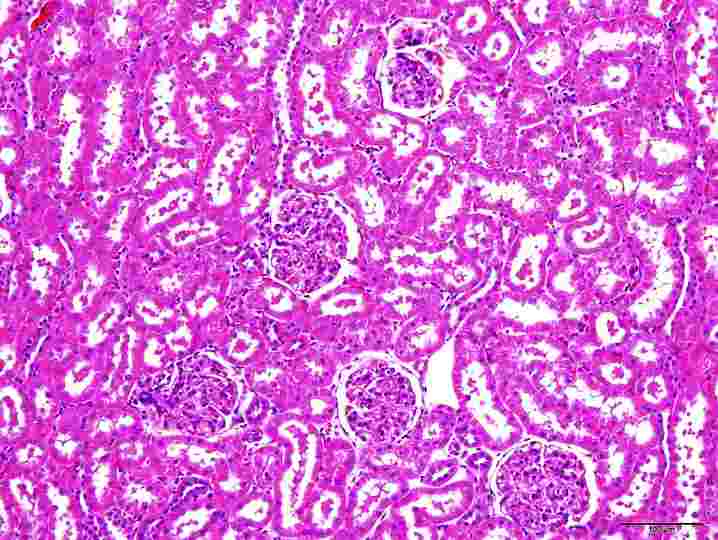

Supplement: Supplementary file 1 [file DataSheet1.zip › Original images and results for Figure 2/Fig. 2H/Fig. 2H-HE/HE-TAC/6-5.jpg]

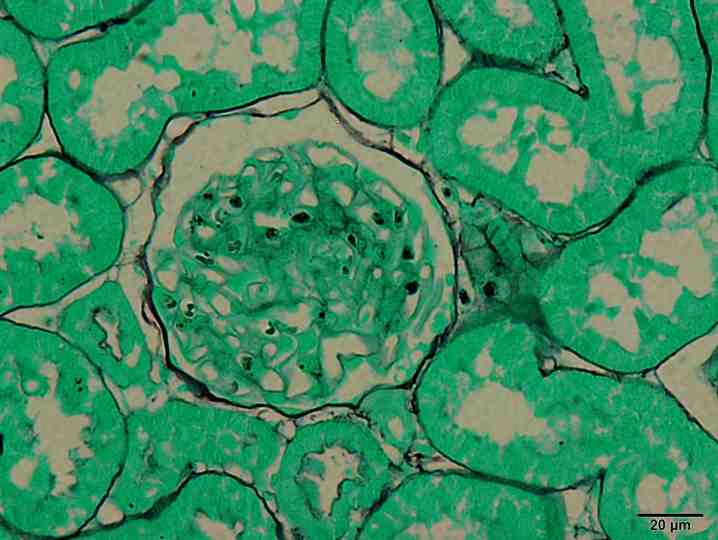

Supplement: Supplementary file 1 [file DataSheet1.zip › Original images and results for Figure 2/Fig. 2H/Fig. 2H-PASM/PASM-CON/1-1.jpg]

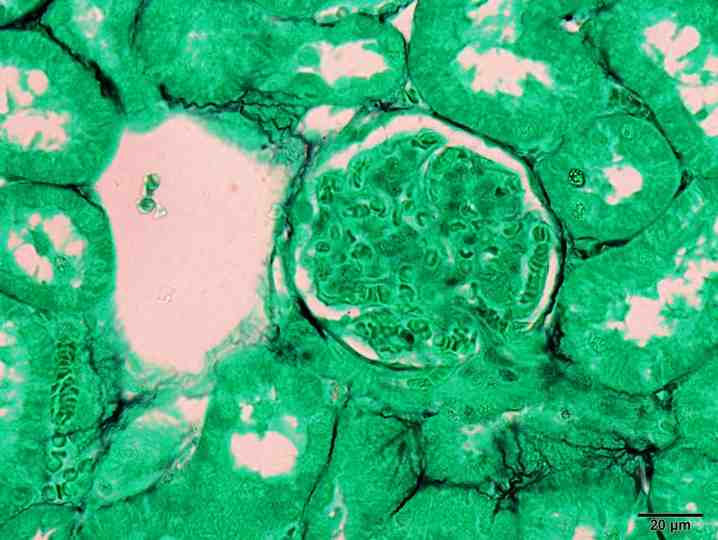

Supplement: Supplementary file 1 [file DataSheet1.zip › Original images and results for Figure 2/Fig. 2H/Fig. 2H-PASM/PASM-CON/1-2.jpg]

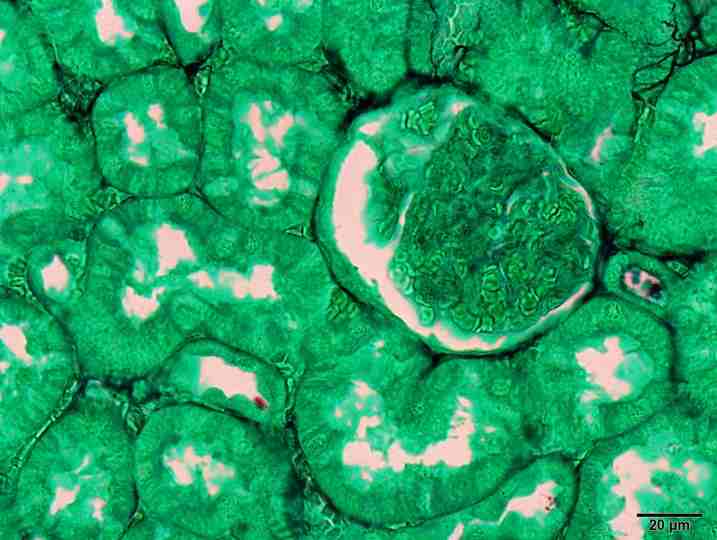

Supplement: Supplementary file 1 [file DataSheet1.zip › Original images and results for Figure 2/Fig. 2H/Fig. 2H-PASM/PASM-CON/1-3.jpg]

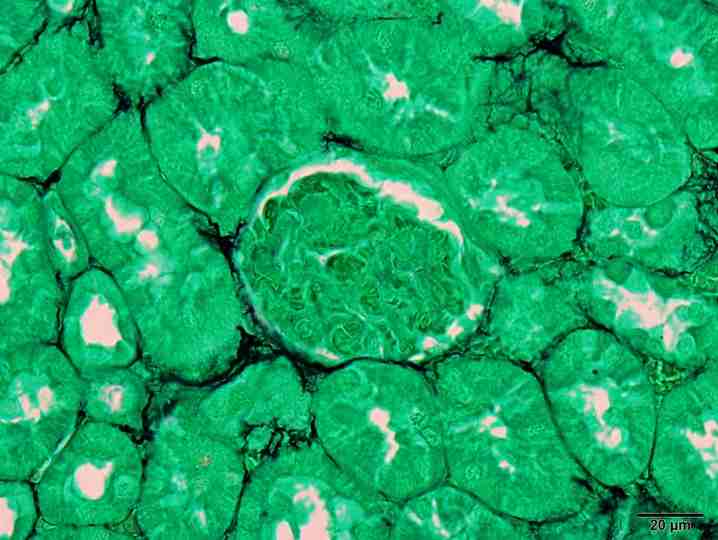

Supplement: Supplementary file 1 [file DataSheet1.zip › Original images and results for Figure 2/Fig. 2H/Fig. 2H-PASM/PASM-CON/1-4.jpg]

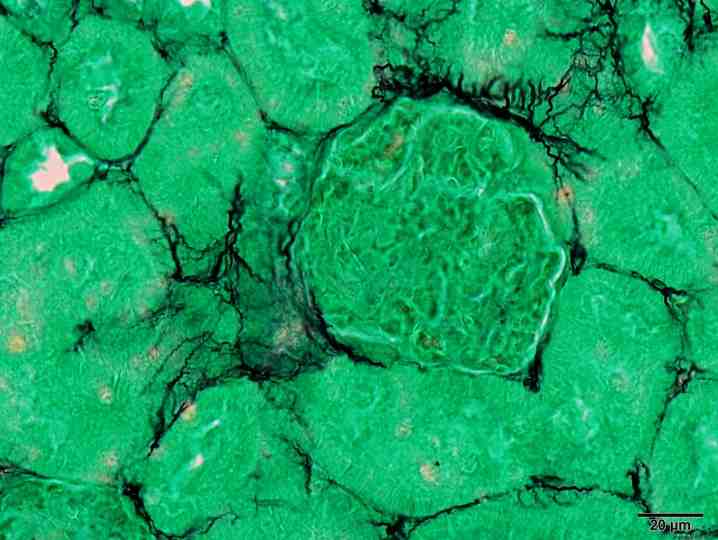

Supplement: Supplementary file 1 [file DataSheet1.zip › Original images and results for Figure 2/Fig. 2H/Fig. 2H-PASM/PASM-CON/1-5.jpg]

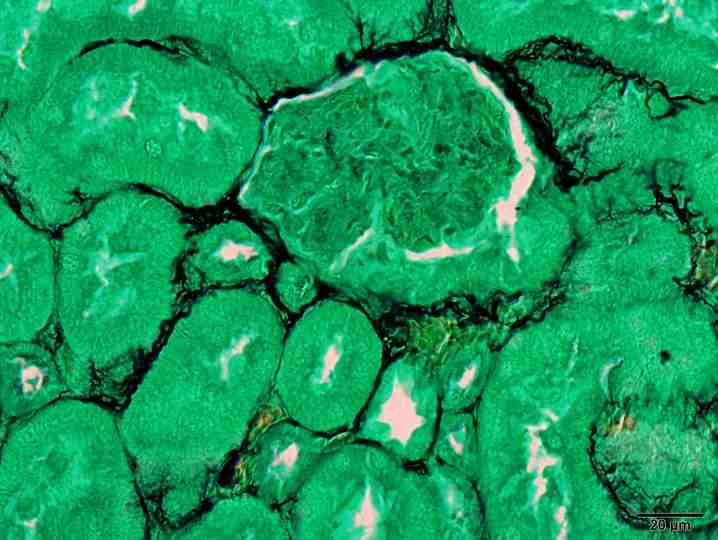

Supplement: Supplementary file 1 [file DataSheet1.zip › Original images and results for Figure 2/Fig. 2H/Fig. 2H-PASM/PASM-CON/2-1.jpg]

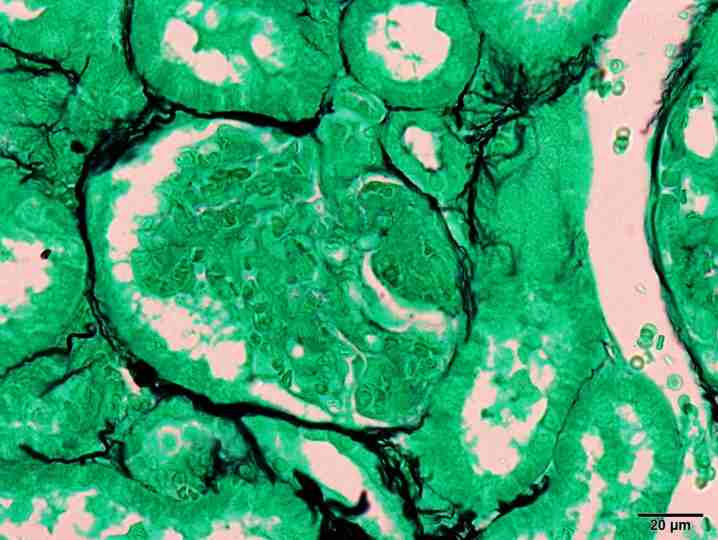

Supplement: Supplementary file 1 [file DataSheet1.zip › Original images and results for Figure 2/Fig. 2H/Fig. 2H-PASM/PASM-CON/2-2.jpg]

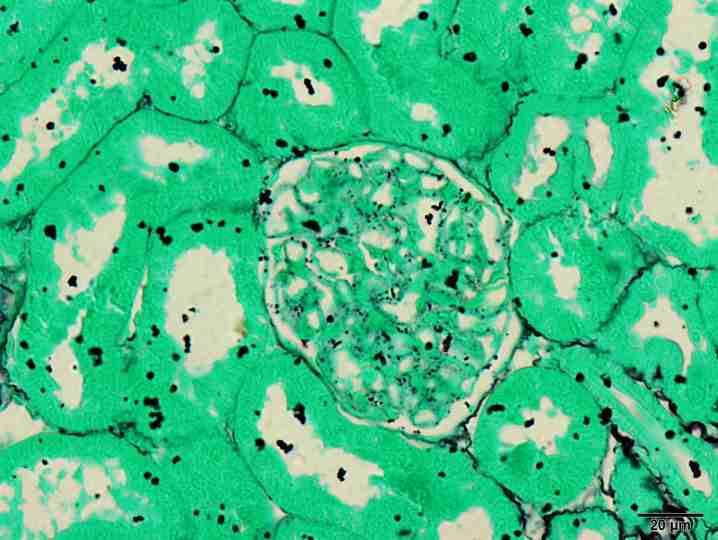

Supplement: Supplementary file 1 [file DataSheet1.zip › Original images and results for Figure 2/Fig. 2H/Fig. 2H-PASM/PASM-CON/2-3.jpg]

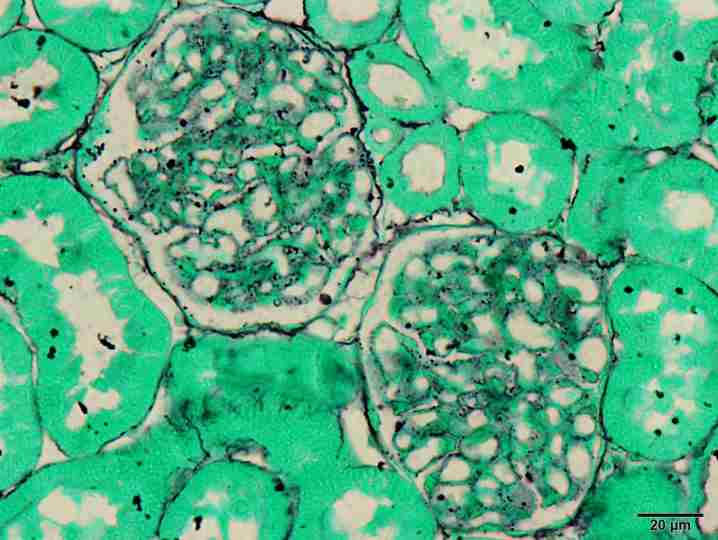

Supplement: Supplementary file 1 [file DataSheet1.zip › Original images and results for Figure 2/Fig. 2H/Fig. 2H-PASM/PASM-CON/2-4.jpg]

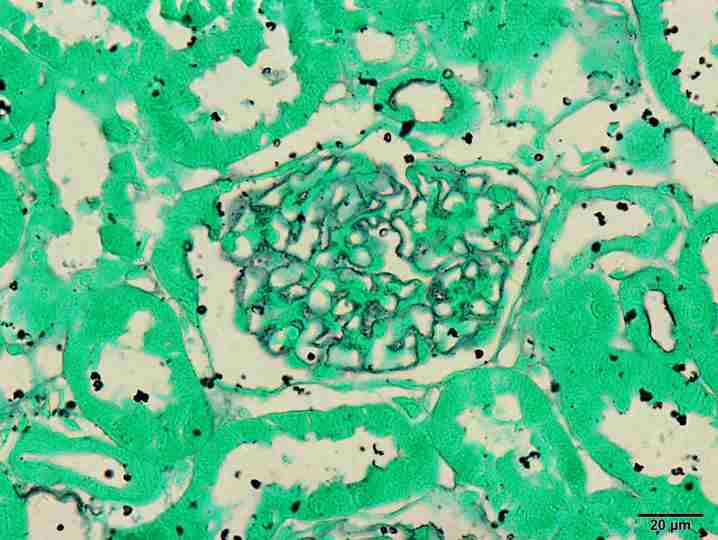

Supplement: Supplementary file 1 [file DataSheet1.zip › Original images and results for Figure 2/Fig. 2H/Fig. 2H-PASM/PASM-CON/2-5.jpg]

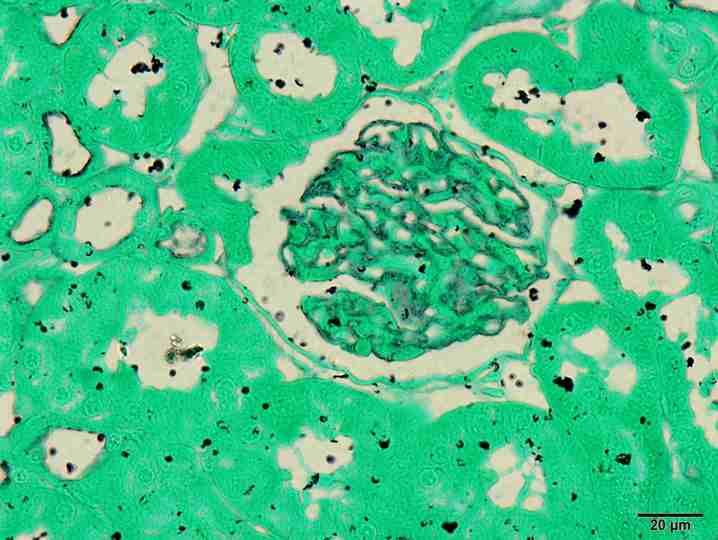

Supplement: Supplementary file 1 [file DataSheet1.zip › Original images and results for Figure 2/Fig. 2H/Fig. 2H-PASM/PASM-CON/3-1.jpg]

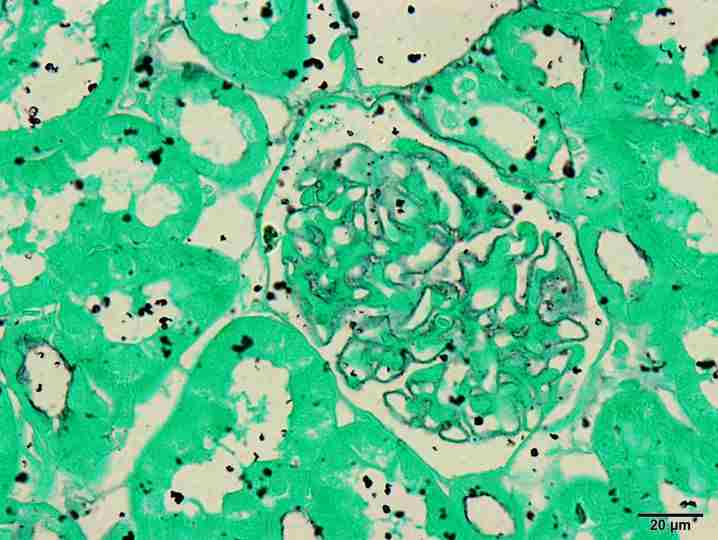

Supplement: Supplementary file 1 [file DataSheet1.zip › Original images and results for Figure 2/Fig. 2H/Fig. 2H-PASM/PASM-CON/3-2.jpg]

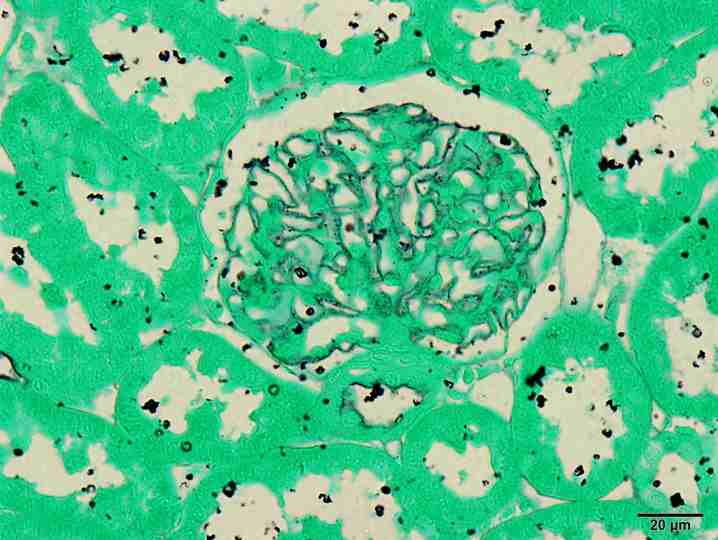

Supplement: Supplementary file 1 [file DataSheet1.zip › Original images and results for Figure 2/Fig. 2H/Fig. 2H-PASM/PASM-CON/3-3.jpg]

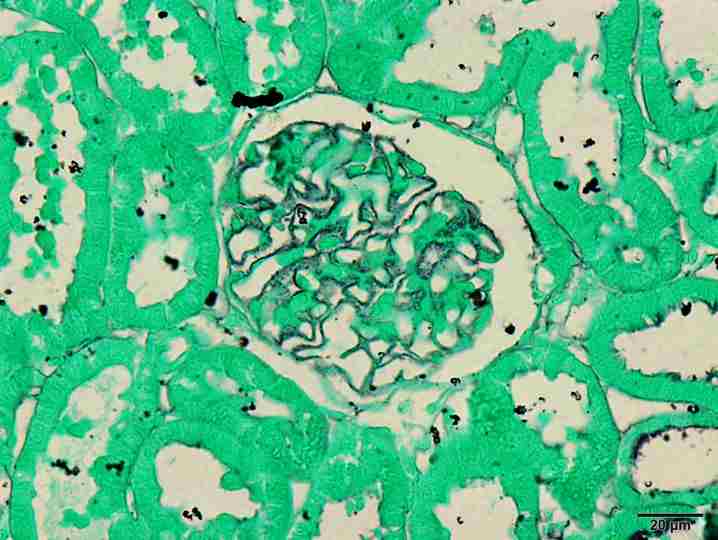

Supplement: Supplementary file 1 [file DataSheet1.zip › Original images and results for Figure 2/Fig. 2H/Fig. 2H-PASM/PASM-CON/3-4.jpg]

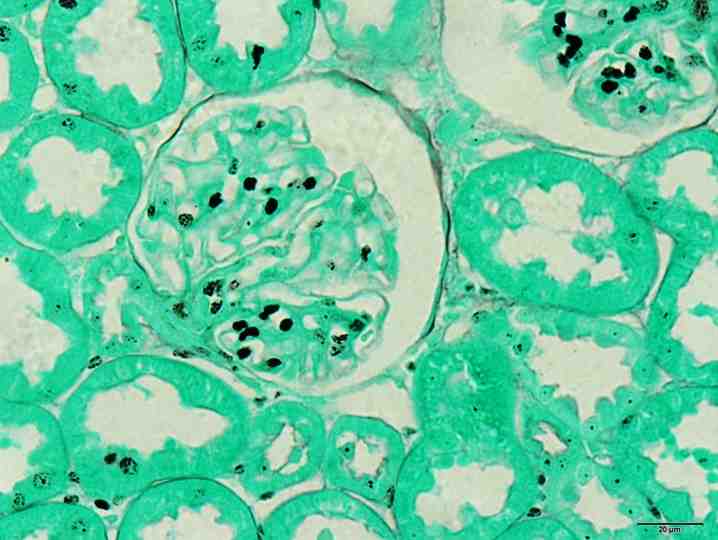

Supplement: Supplementary file 1 [file DataSheet1.zip › Original images and results for Figure 2/Fig. 2H/Fig. 2H-PASM/PASM-CON/3-5.jpg]

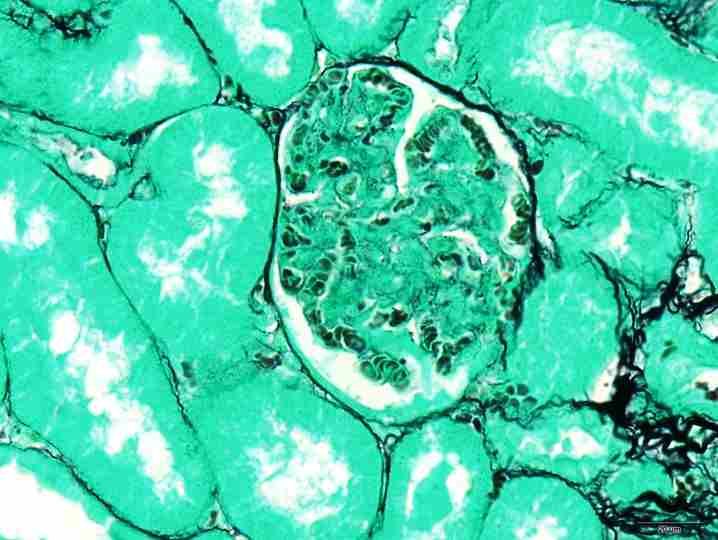

Supplement: Supplementary file 1 [file DataSheet1.zip › Original images and results for Figure 2/Fig. 2H/Fig. 2H-PASM/PASM-CON/4-1.jpg]

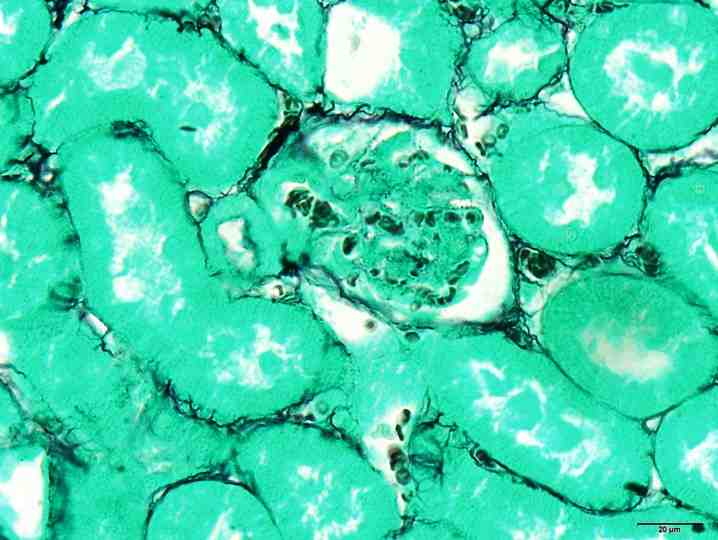

Supplement: Supplementary file 1 [file DataSheet1.zip › Original images and results for Figure 2/Fig. 2H/Fig. 2H-PASM/PASM-CON/4-2.jpg]

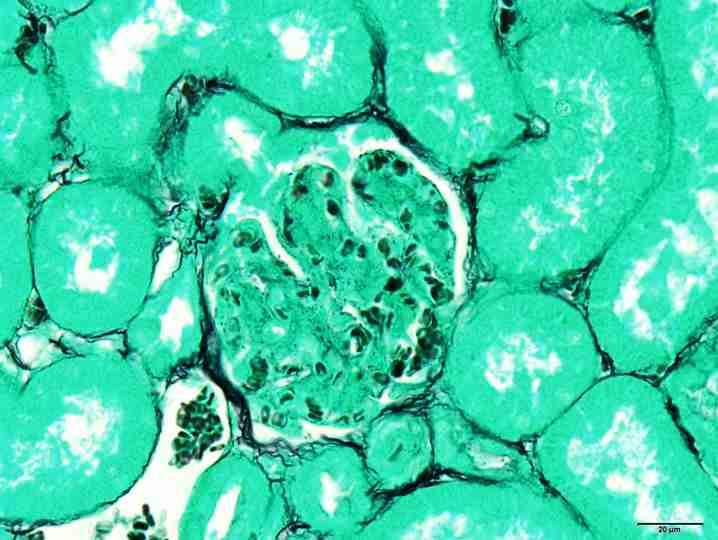

Supplement: Supplementary file 1 [file DataSheet1.zip › Original images and results for Figure 2/Fig. 2H/Fig. 2H-PASM/PASM-CON/4-3.jpg]

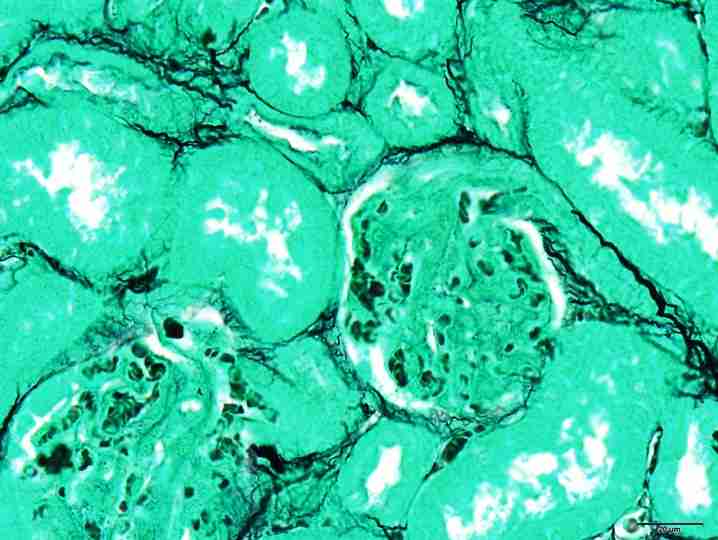

Supplement: Supplementary file 1 [file DataSheet1.zip › Original images and results for Figure 2/Fig. 2H/Fig. 2H-PASM/PASM-CON/4-4.jpg]

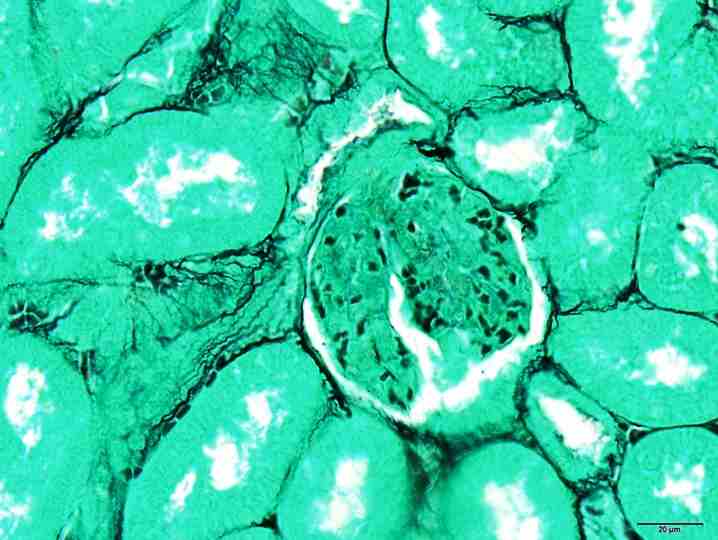

Supplement: Supplementary file 1 [file DataSheet1.zip › Original images and results for Figure 2/Fig. 2H/Fig. 2H-PASM/PASM-CON/4-5.jpg]

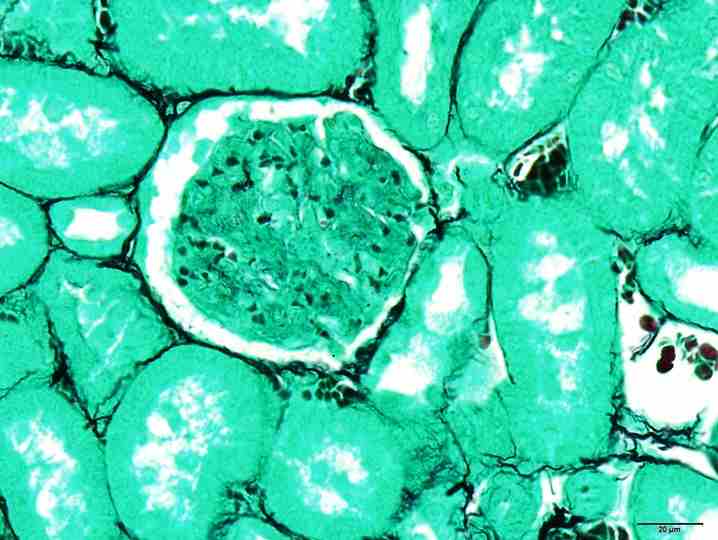

Supplement: Supplementary file 1 [file DataSheet1.zip › Original images and results for Figure 2/Fig. 2H/Fig. 2H-PASM/PASM-CON/5-1.jpg]

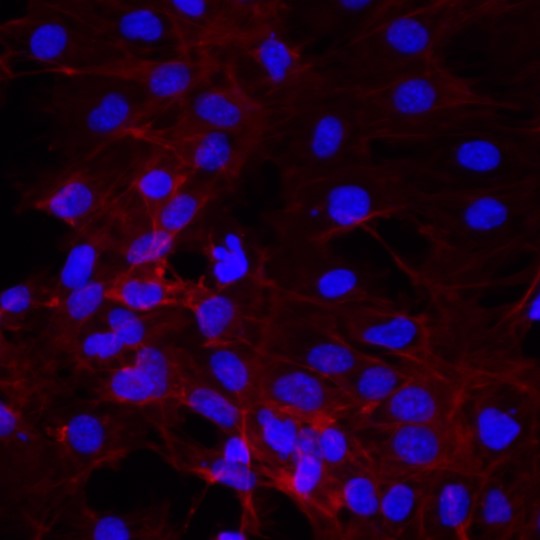

Supplement: Supplementary file 3 [file DataSheet2.zip › Original images and results for Figure 6/Fig. 6B/Fig. 6B Phalloidin/IF-Phalloidin-ADR+SQL-1-1.jpg]

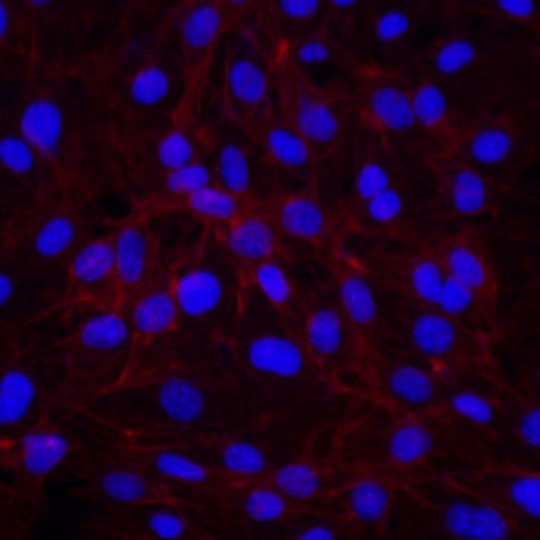

Supplement: Supplementary file 3 [file DataSheet2.zip › Original images and results for Figure 6/Fig. 6B/Fig. 6B Phalloidin/IF-Phalloidin-ADR+SQL-1-2.jpg]

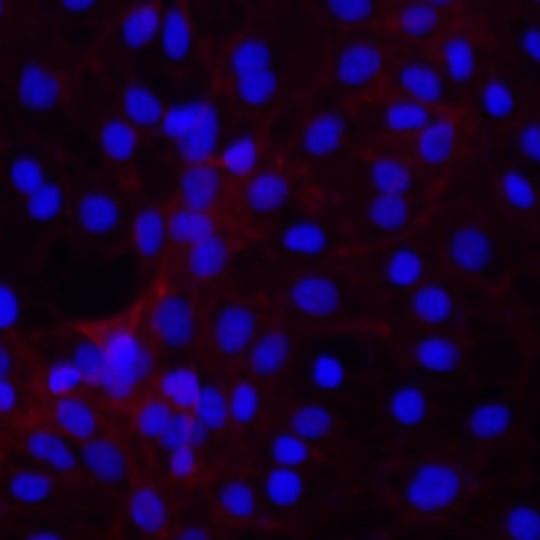

Supplement: Supplementary file 3 [file DataSheet2.zip › Original images and results for Figure 6/Fig. 6B/Fig. 6B Phalloidin/IF-Phalloidin-ADR+SQL-1-3.jpg]

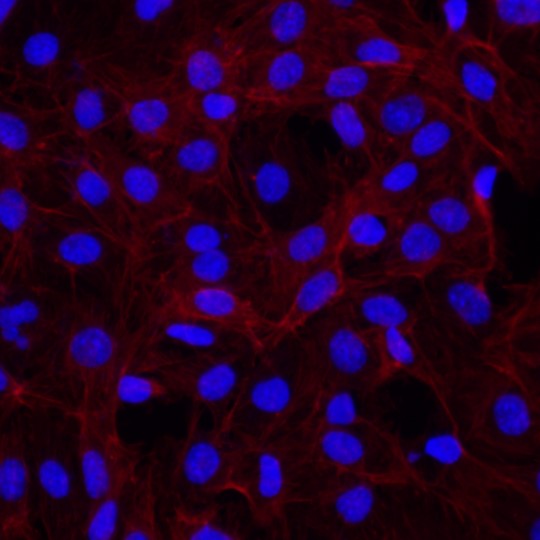

Supplement: Supplementary file 3 [file DataSheet2.zip › Original images and results for Figure 6/Fig. 6B/Fig. 6B Phalloidin/IF-Phalloidin-ADR+SQL-1-4.jpg]

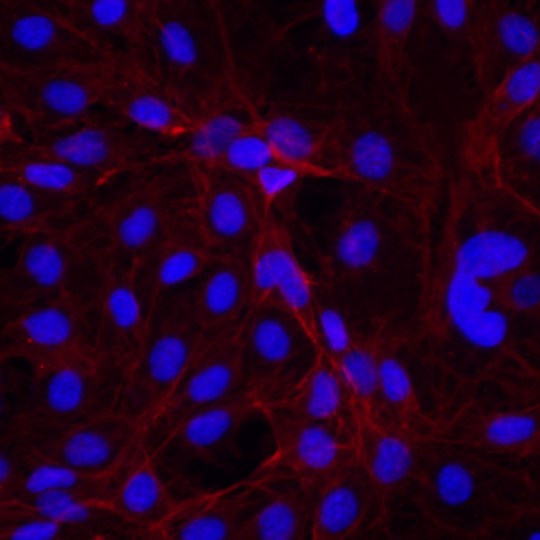

Supplement: Supplementary file 3 [file DataSheet2.zip › Original images and results for Figure 6/Fig. 6B/Fig. 6B Phalloidin/IF-Phalloidin-ADR+SQL-1-5.jpg]

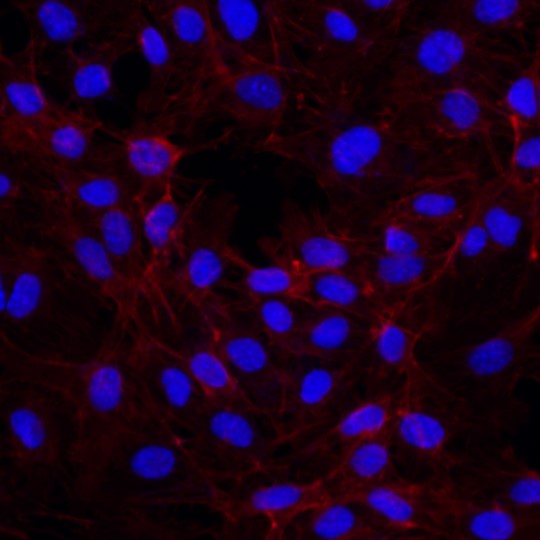

Supplement: Supplementary file 3 [file DataSheet2.zip › Original images and results for Figure 6/Fig. 6B/Fig. 6B Phalloidin/IF-Phalloidin-ADR+SQL-2-1.jpg]

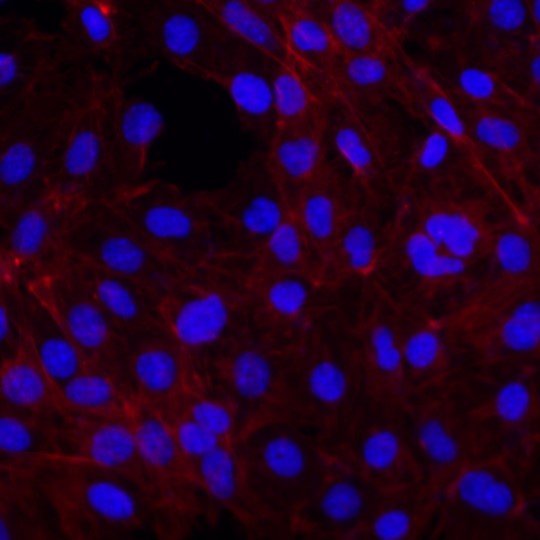

Supplement: Supplementary file 3 [file DataSheet2.zip › Original images and results for Figure 6/Fig. 6B/Fig. 6B Phalloidin/IF-Phalloidin-ADR+SQL-2-2.jpg]

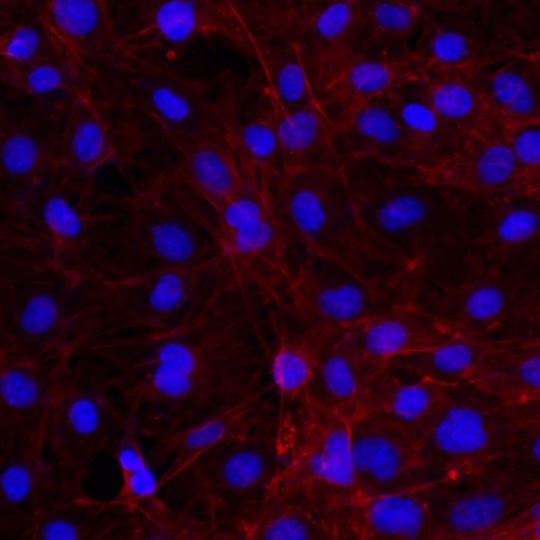

Supplement: Supplementary file 3 [file DataSheet2.zip › Original images and results for Figure 6/Fig. 6B/Fig. 6B Phalloidin/IF-Phalloidin-ADR+SQL-2-3 image in Fig. 6B.jpg]

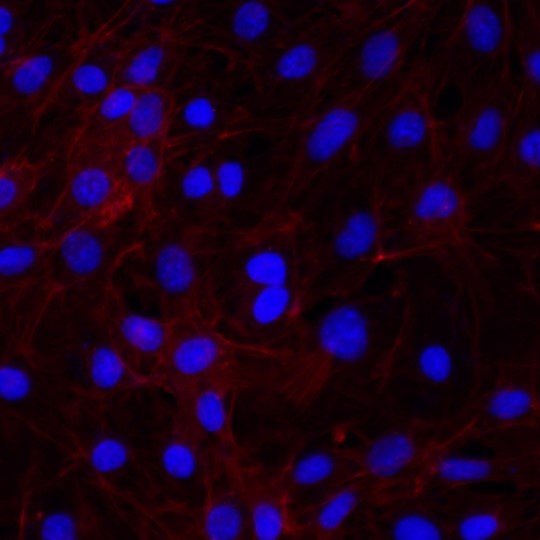

Supplement: Supplementary file 3 [file DataSheet2.zip › Original images and results for Figure 6/Fig. 6B/Fig. 6B Phalloidin/IF-Phalloidin-ADR+SQL-2-4.jpg]

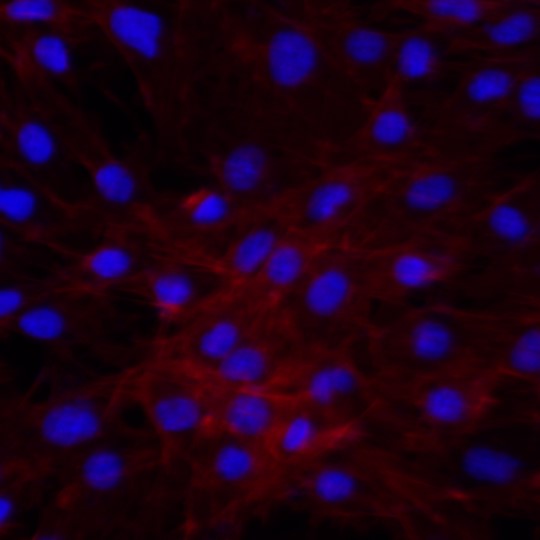

Supplement: Supplementary file 3 [file DataSheet2.zip › Original images and results for Figure 6/Fig. 6B/Fig. 6B Phalloidin/IF-Phalloidin-ADR+SQL-2-5.jpg]

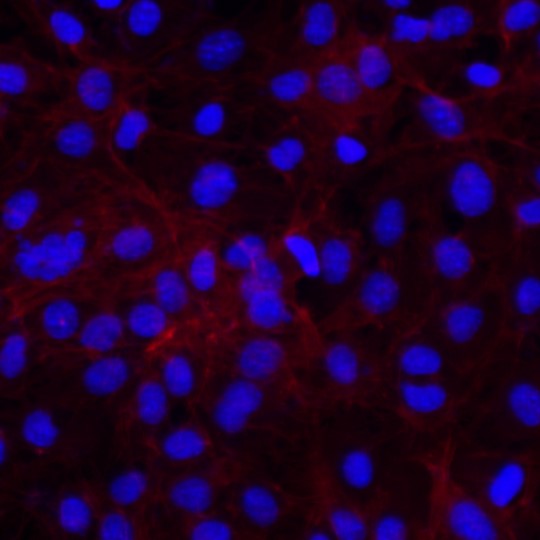

Supplement: Supplementary file 3 [file DataSheet2.zip › Original images and results for Figure 6/Fig. 6B/Fig. 6B Phalloidin/IF-Phalloidin-ADR+SQL-3-1.jpg]

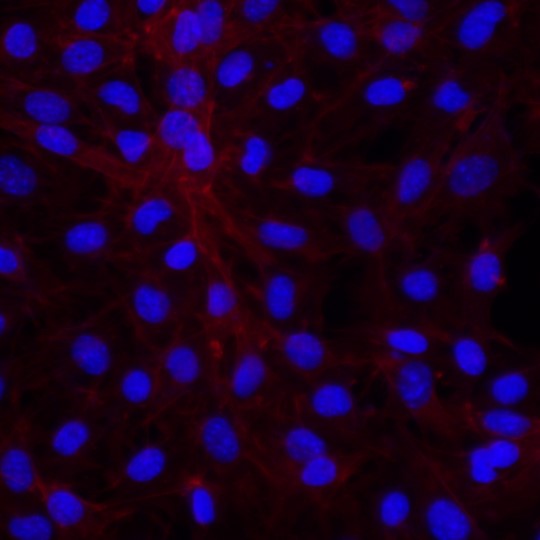

Supplement: Supplementary file 3 [file DataSheet2.zip › Original images and results for Figure 6/Fig. 6B/Fig. 6B Phalloidin/IF-Phalloidin-ADR+SQL-3-2.jpg]

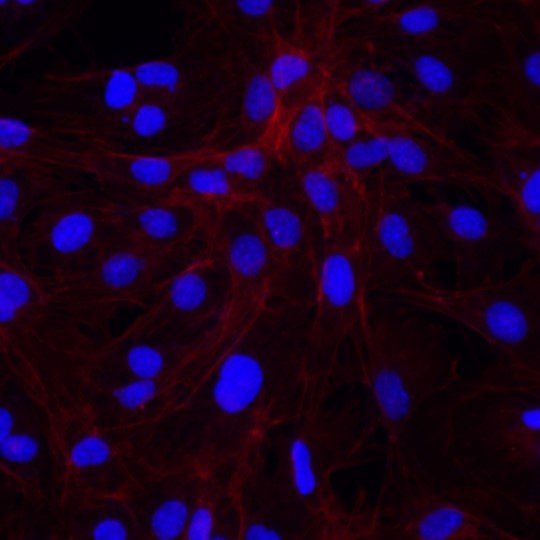

Supplement: Supplementary file 3 [file DataSheet2.zip › Original images and results for Figure 6/Fig. 6B/Fig. 6B Phalloidin/IF-Phalloidin-ADR+SQL-3-3.jpg]

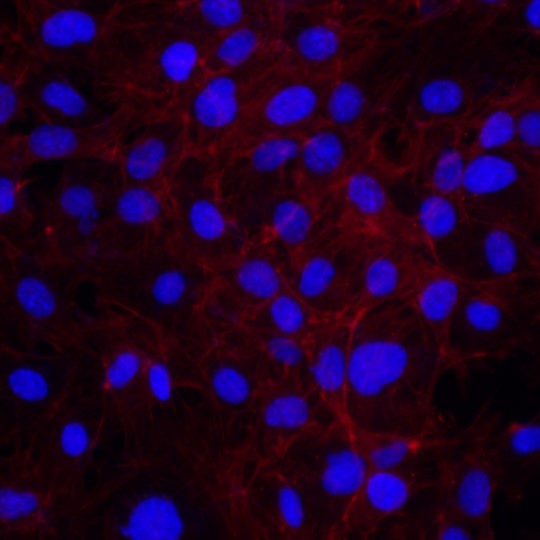

Supplement: Supplementary file 3 [file DataSheet2.zip › Original images and results for Figure 6/Fig. 6B/Fig. 6B Phalloidin/IF-Phalloidin-ADR+SQL-3-4.jpg]

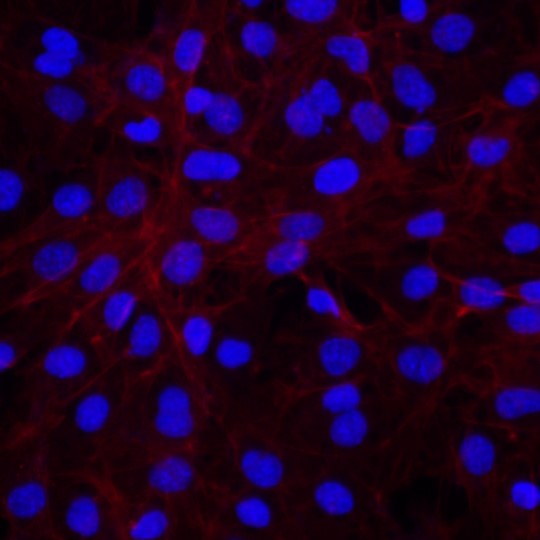

Supplement: Supplementary file 3 [file DataSheet2.zip › Original images and results for Figure 6/Fig. 6B/Fig. 6B Phalloidin/IF-Phalloidin-ADR+SQL-3-5.jpg]

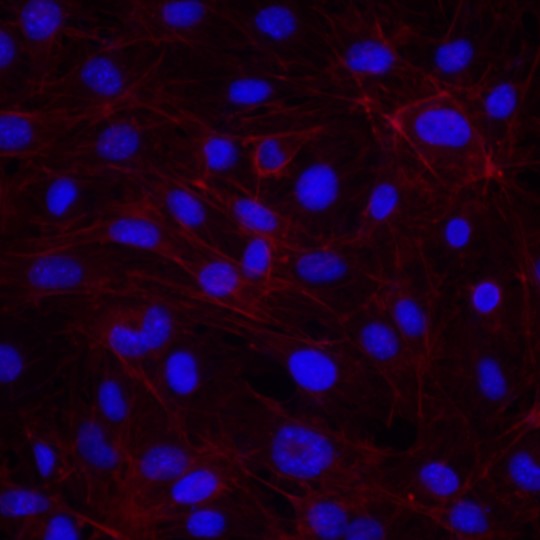

Supplement: Supplementary file 3 [file DataSheet2.zip › Original images and results for Figure 6/Fig. 6B/Fig. 6B Phalloidin/IF-Phalloidin-ADR-1-1.jpg]

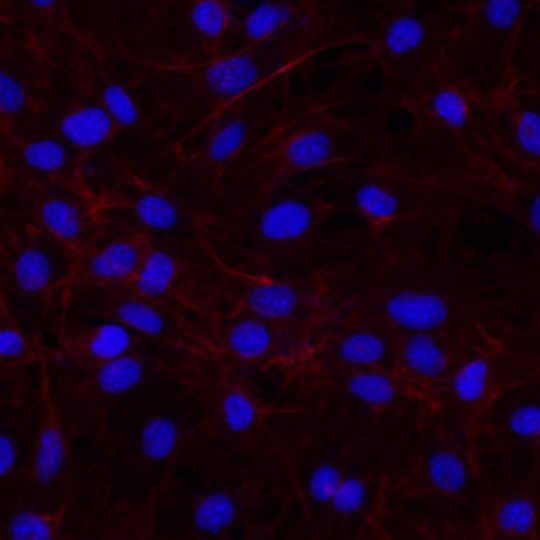

Supplement: Supplementary file 3 [file DataSheet2.zip › Original images and results for Figure 6/Fig. 6B/Fig. 6B Phalloidin/IF-Phalloidin-ADR-1-2.jpg]

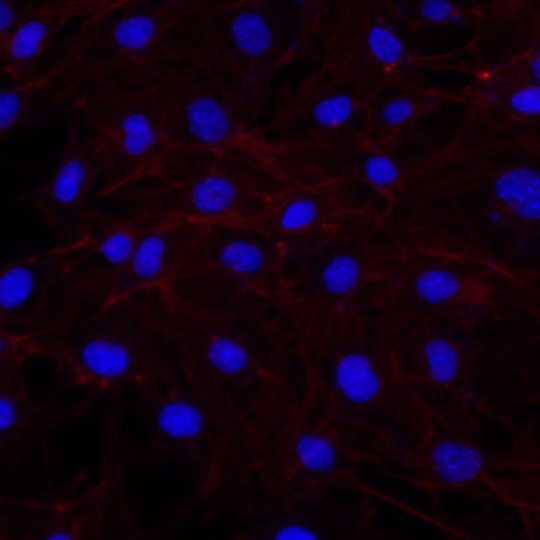

Supplement: Supplementary file 3 [file DataSheet2.zip › Original images and results for Figure 6/Fig. 6B/Fig. 6B Phalloidin/IF-Phalloidin-ADR-1-3 image in Fig. 6B.jpg]

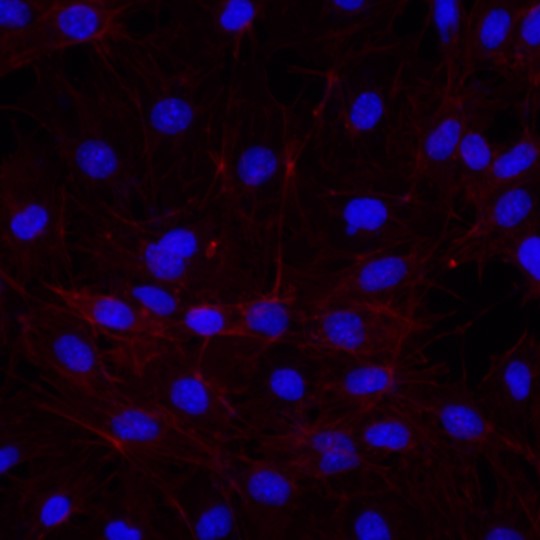

Supplement: Supplementary file 3 [file DataSheet2.zip › Original images and results for Figure 6/Fig. 6B/Fig. 6B Phalloidin/IF-Phalloidin-ADR-1-4.jpg]
