# Supplementary figures and images for: Sanqi oral solution alleviates podocyte apoptosis in experimental membranous nephropathy by mediating EMT through the ERK/CK2-α/β-catenin pathway (part 1 of 4)
Source: Front Pharmacol. 2025 May 9;16:1503961. doi: 10.3389/fphar.2025.1503961 (PMC12098599; doi:10.3389/fphar.2025.1503961)

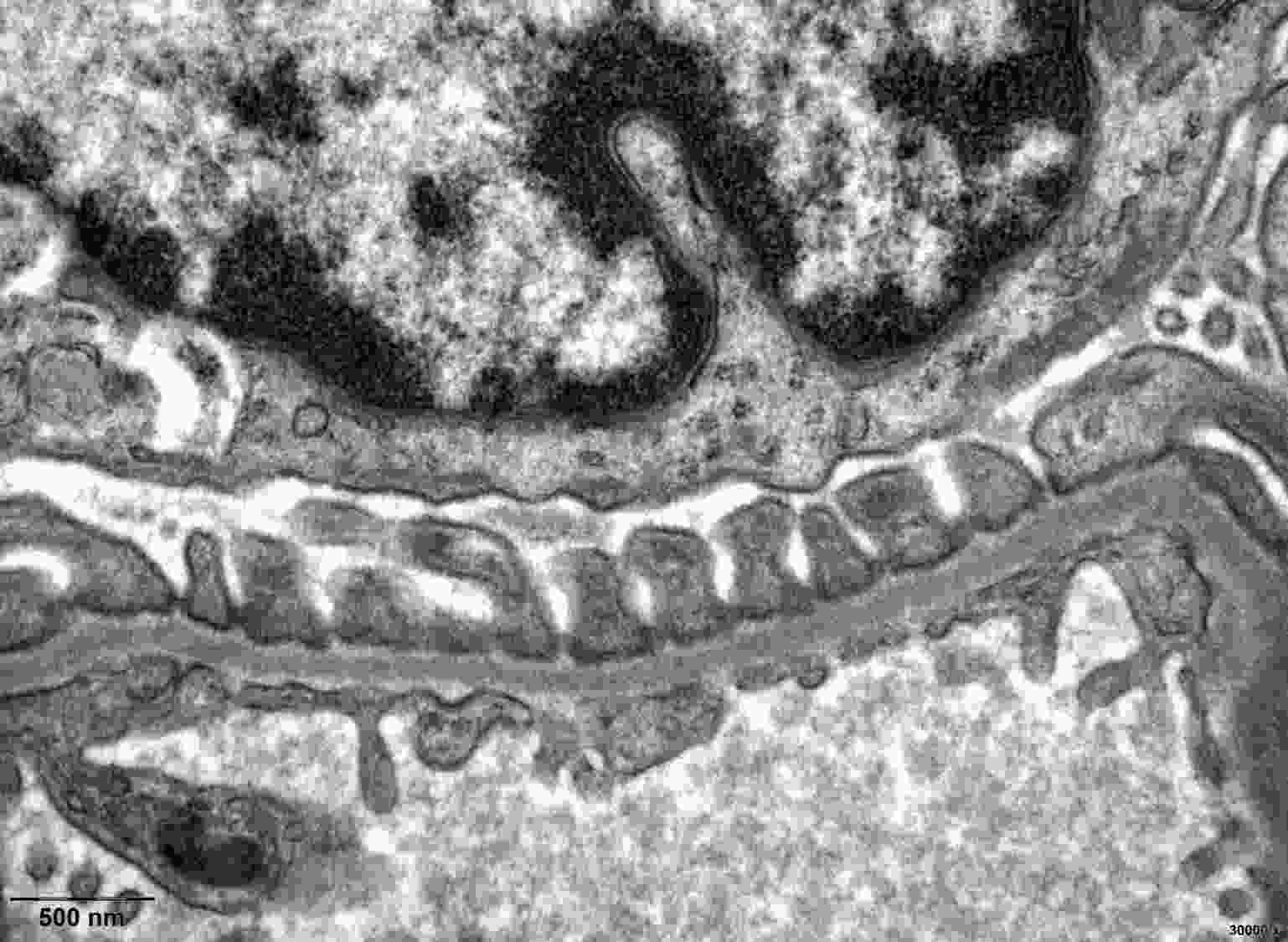

Supplement: Supplementary file 1 [file DataSheet1.zip › Original images and results for Figure 2/Fig. 2H/Fig. 2H-Electron dense deposit/CON-1.jpg]

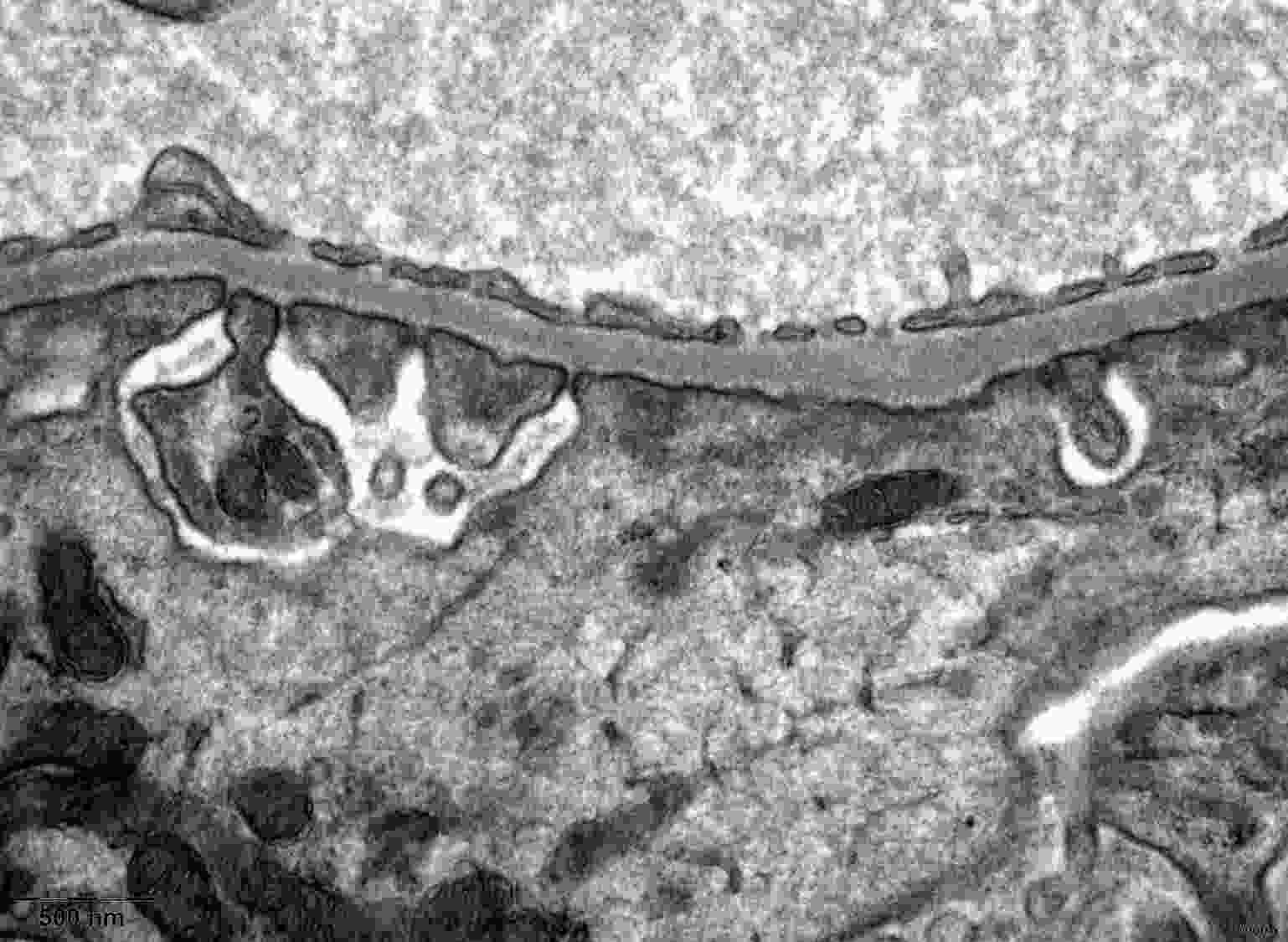

Supplement: Supplementary file 1 [file DataSheet1.zip › Original images and results for Figure 2/Fig. 2H/Fig. 2H-Electron dense deposit/CON-2.jpg]

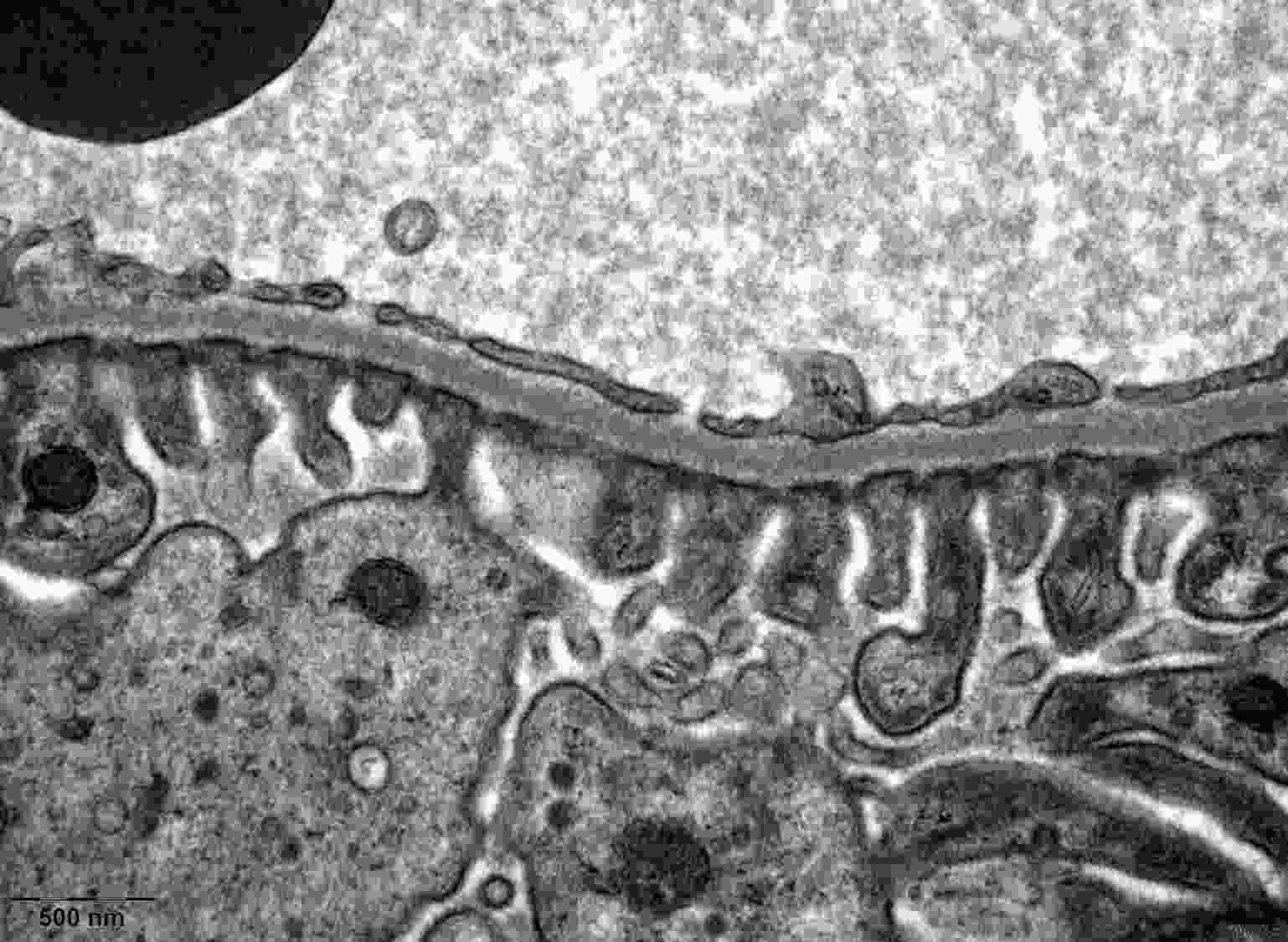

Supplement: Supplementary file 1 [file DataSheet1.zip › Original images and results for Figure 2/Fig. 2H/Fig. 2H-Electron dense deposit/CON-3 image in Fig. 2H .jpg]

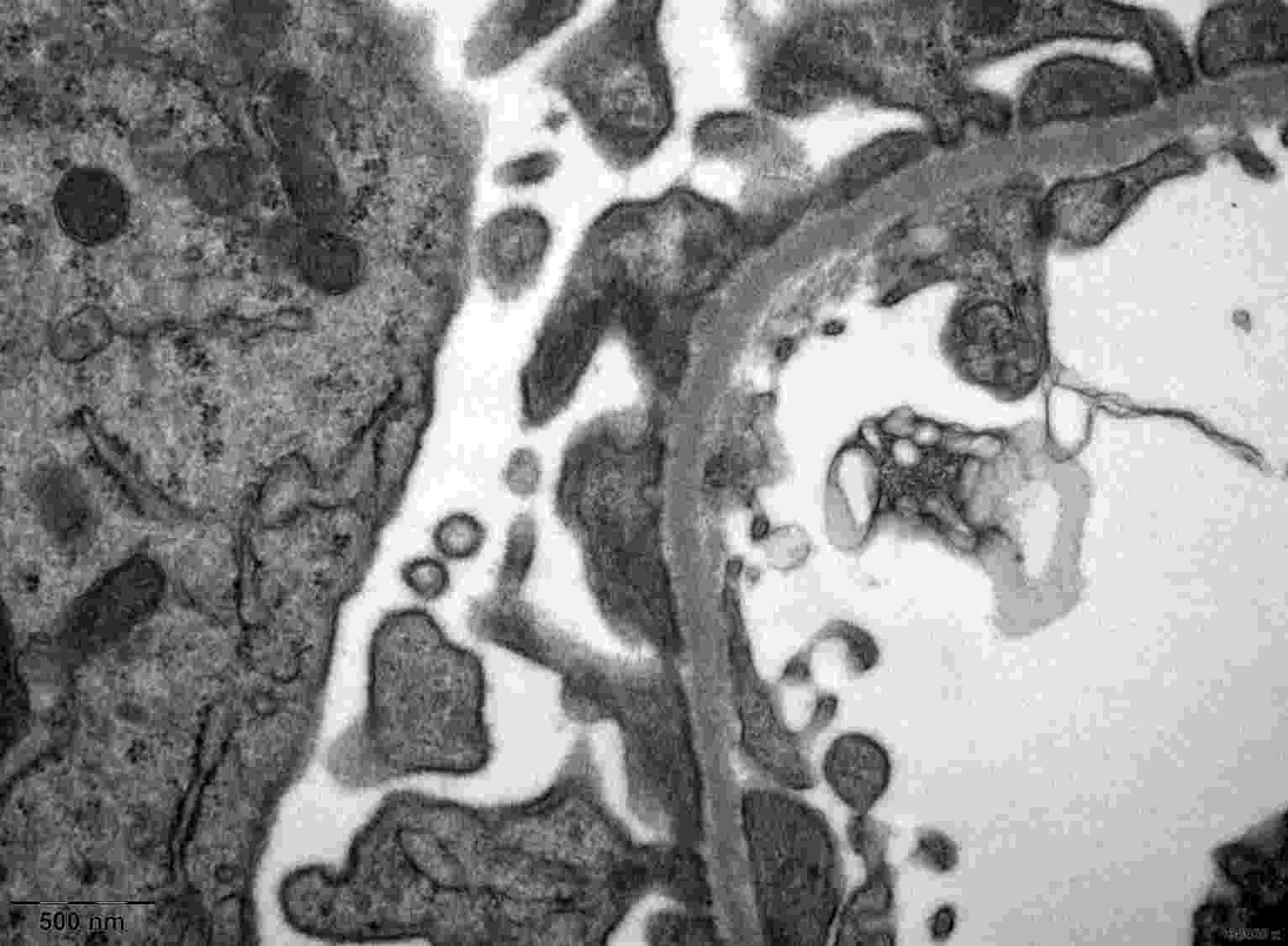

Supplement: Supplementary file 1 [file DataSheet1.zip › Original images and results for Figure 2/Fig. 2H/Fig. 2H-Electron dense deposit/CON-4.jpg]

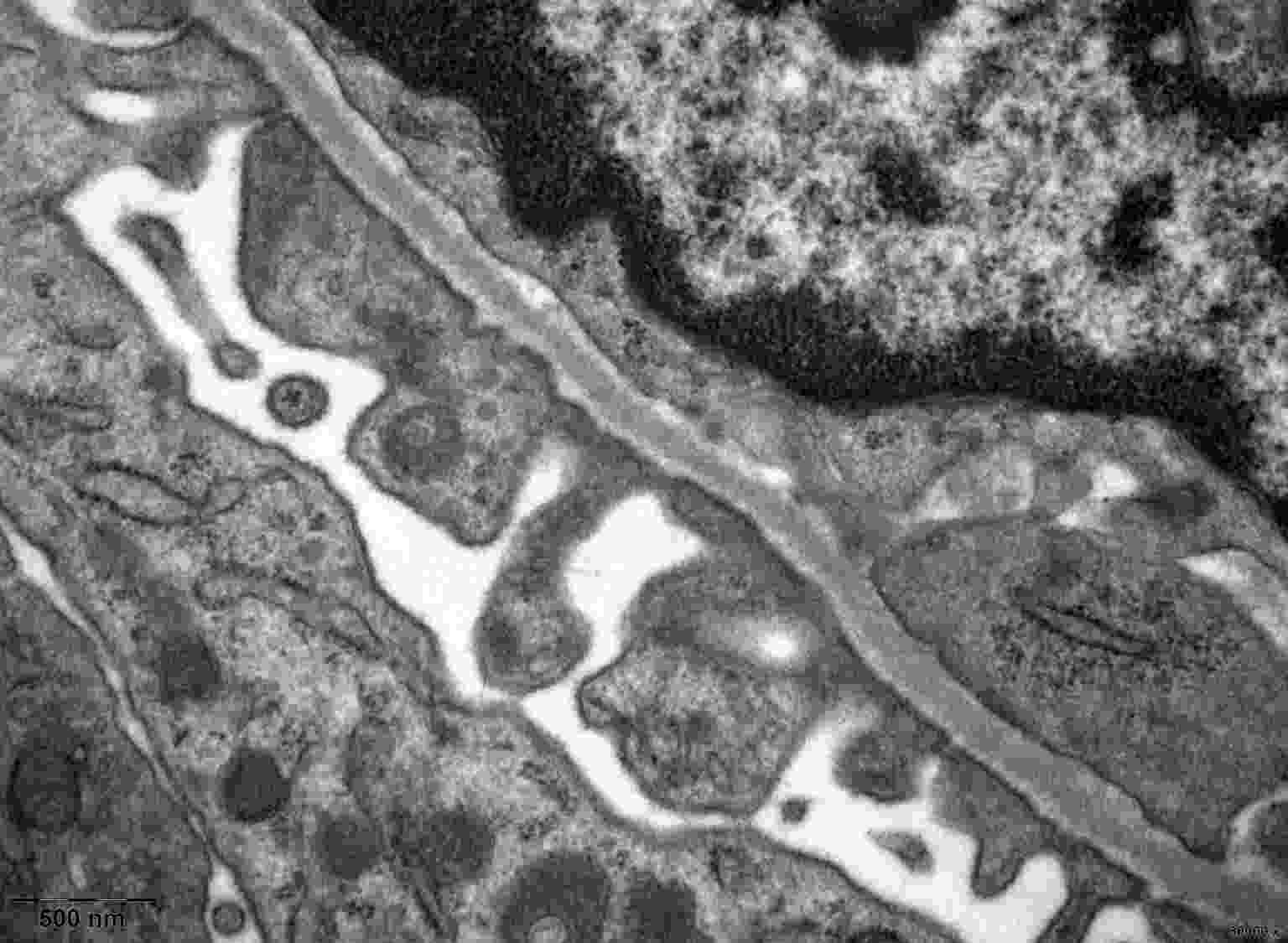

Supplement: Supplementary file 1 [file DataSheet1.zip › Original images and results for Figure 2/Fig. 2H/Fig. 2H-Electron dense deposit/CON-5.jpg]

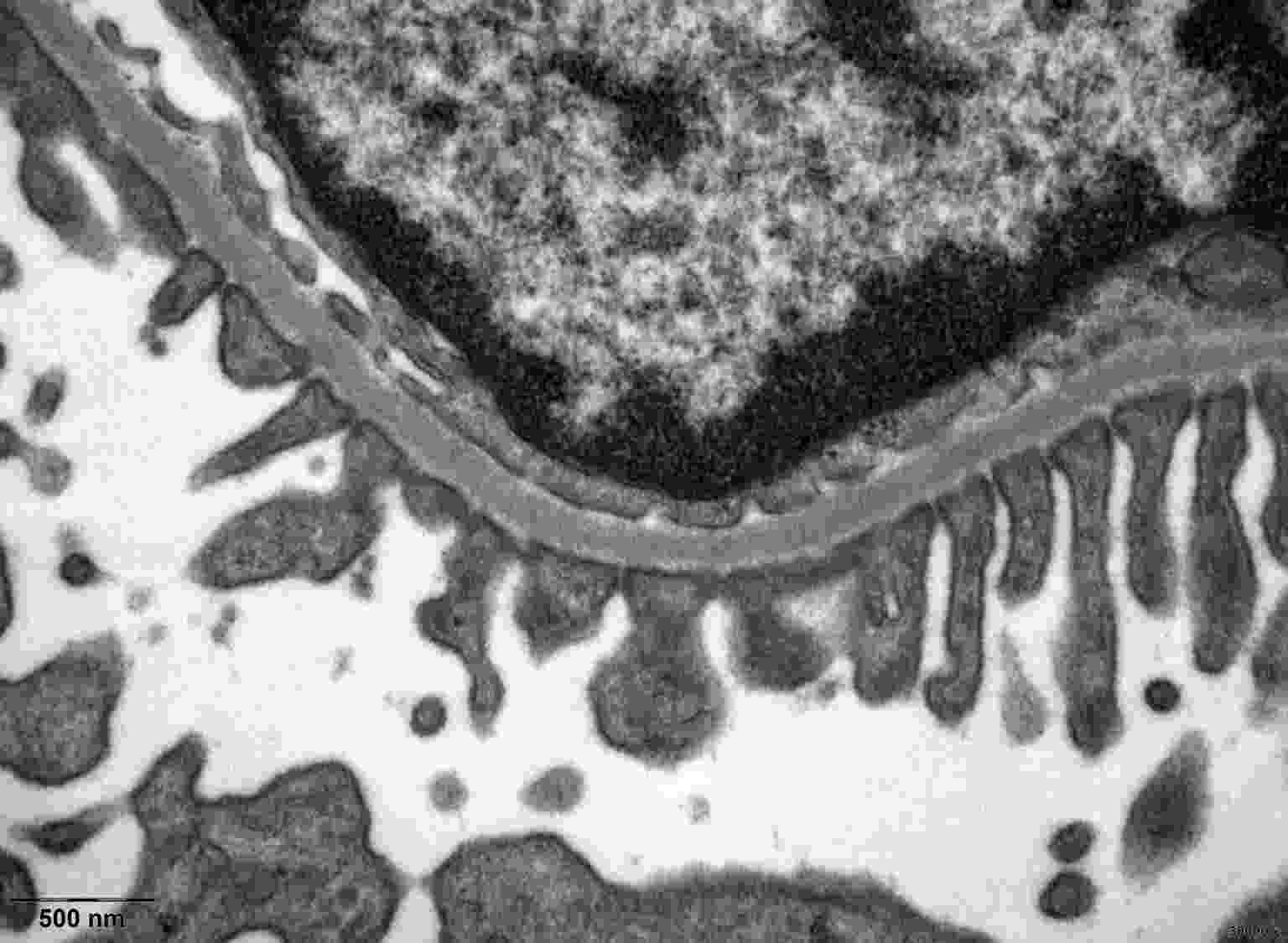

Supplement: Supplementary file 1 [file DataSheet1.zip › Original images and results for Figure 2/Fig. 2H/Fig. 2H-Electron dense deposit/CON-6.jpg]

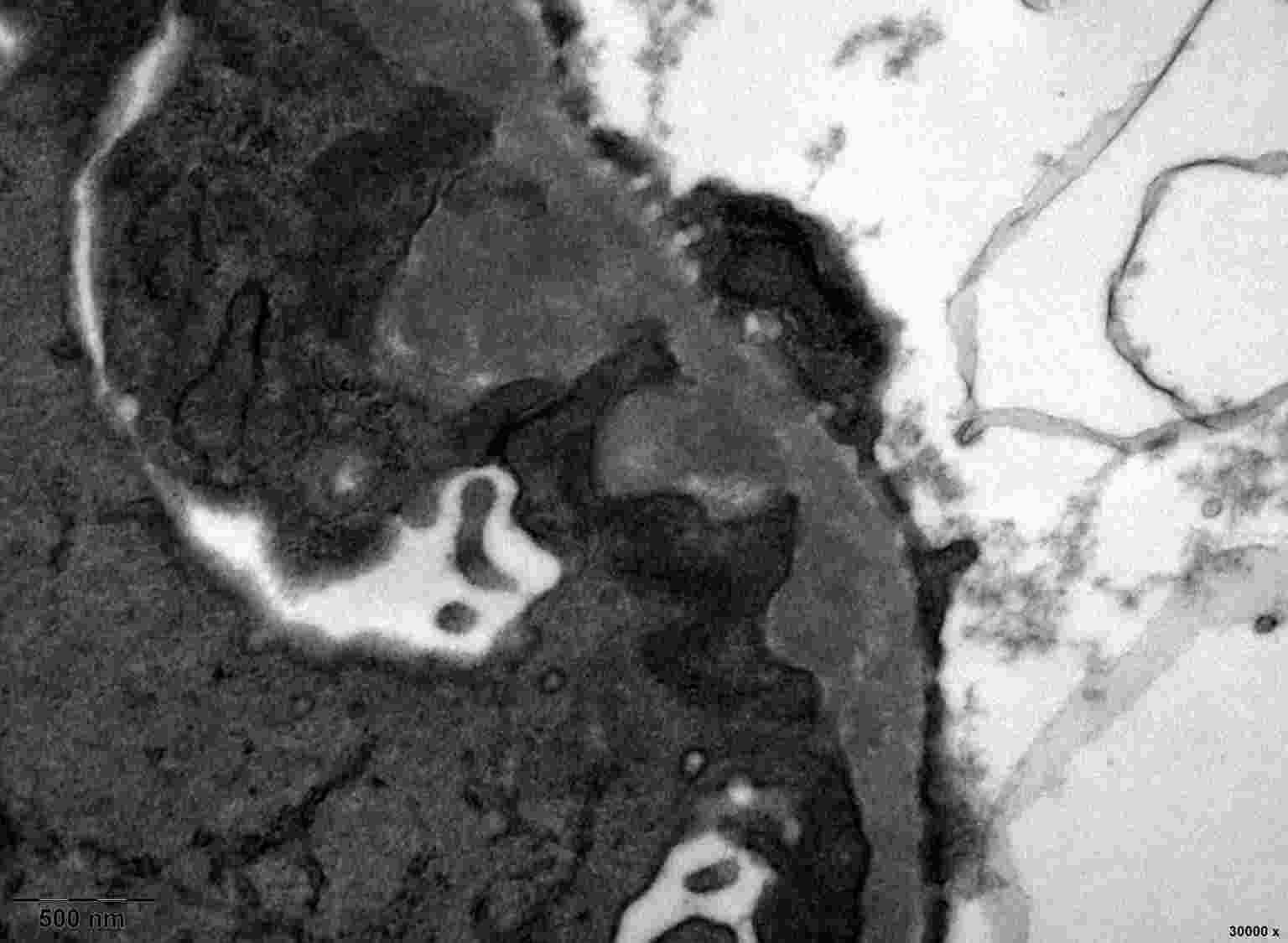

Supplement: Supplementary file 1 [file DataSheet1.zip › Original images and results for Figure 2/Fig. 2H/Fig. 2H-Electron dense deposit/PHN-1.jpg]

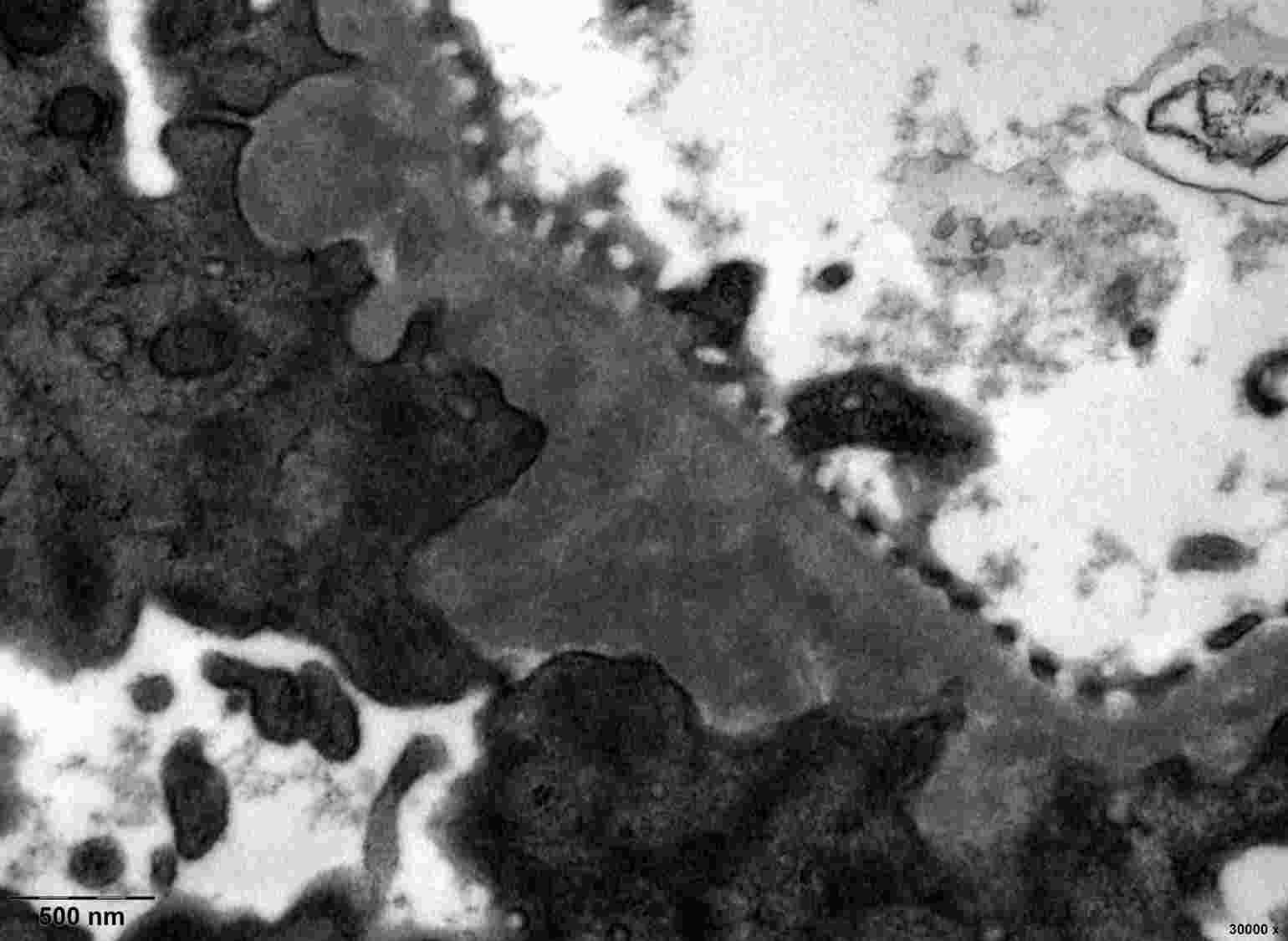

Supplement: Supplementary file 1 [file DataSheet1.zip › Original images and results for Figure 2/Fig. 2H/Fig. 2H-Electron dense deposit/PHN-2.jpg]

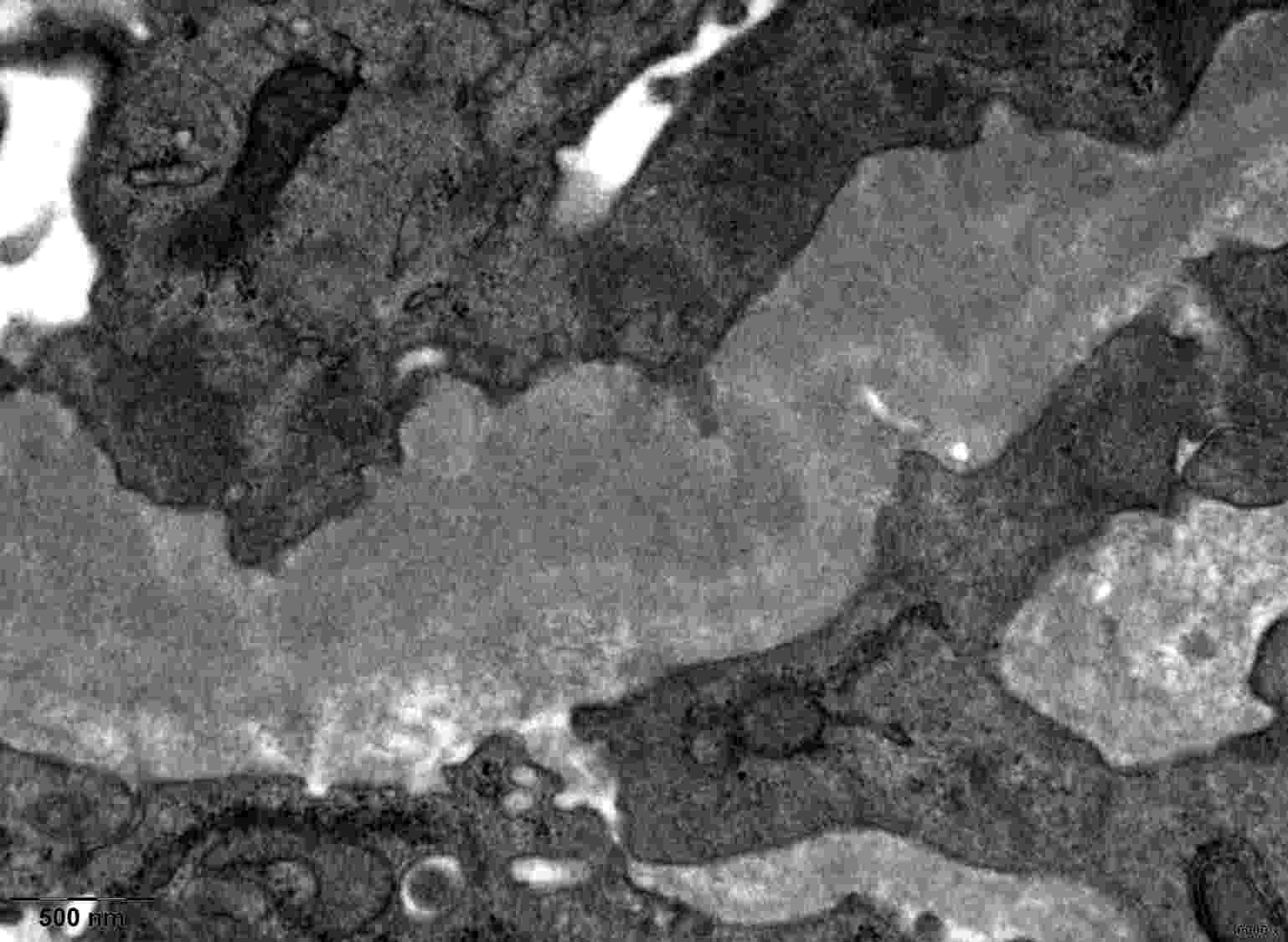

Supplement: Supplementary file 1 [file DataSheet1.zip › Original images and results for Figure 2/Fig. 2H/Fig. 2H-Electron dense deposit/PHN-3.jpg]

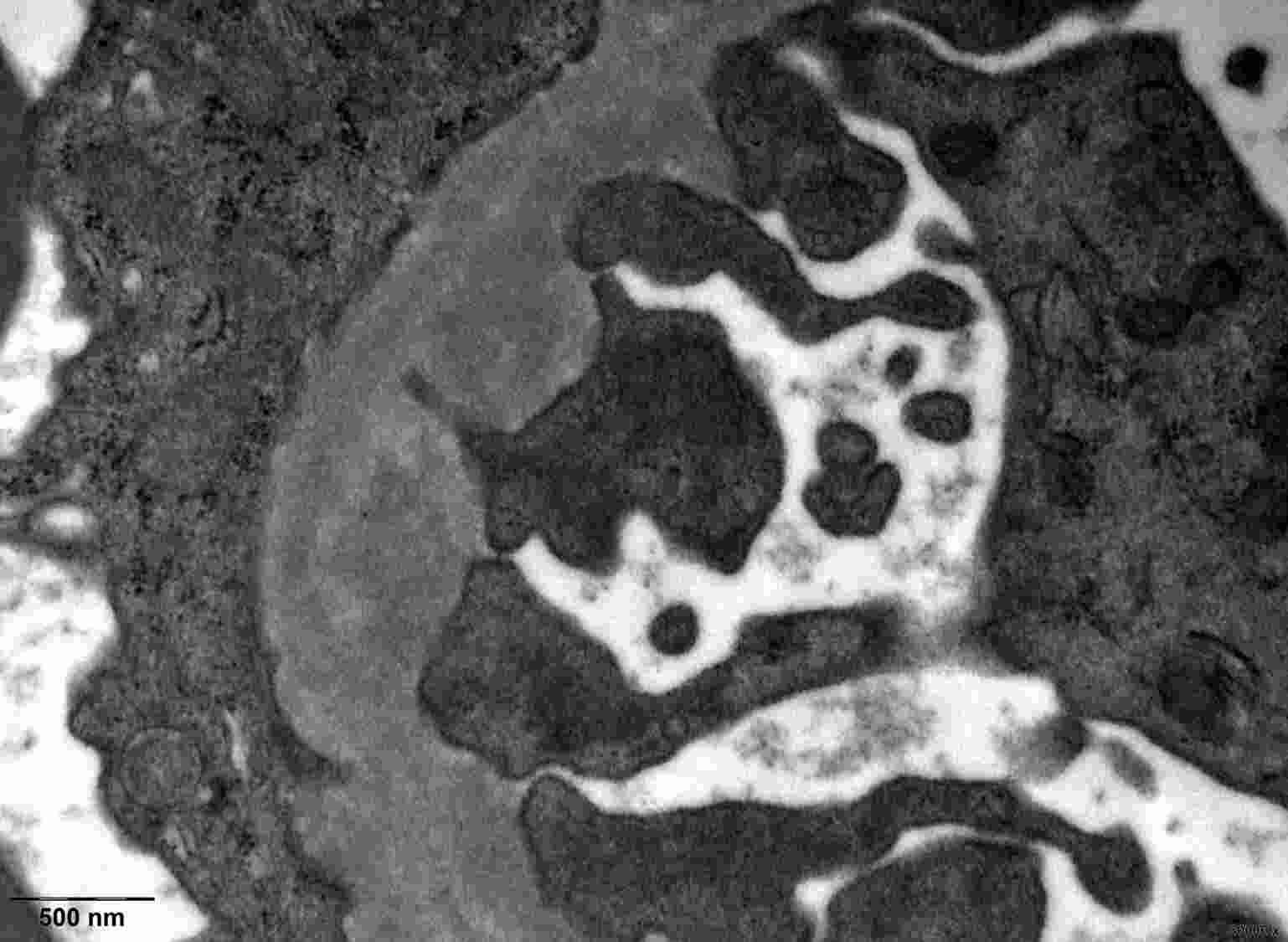

Supplement: Supplementary file 1 [file DataSheet1.zip › Original images and results for Figure 2/Fig. 2H/Fig. 2H-Electron dense deposit/PHN-4.jpg]

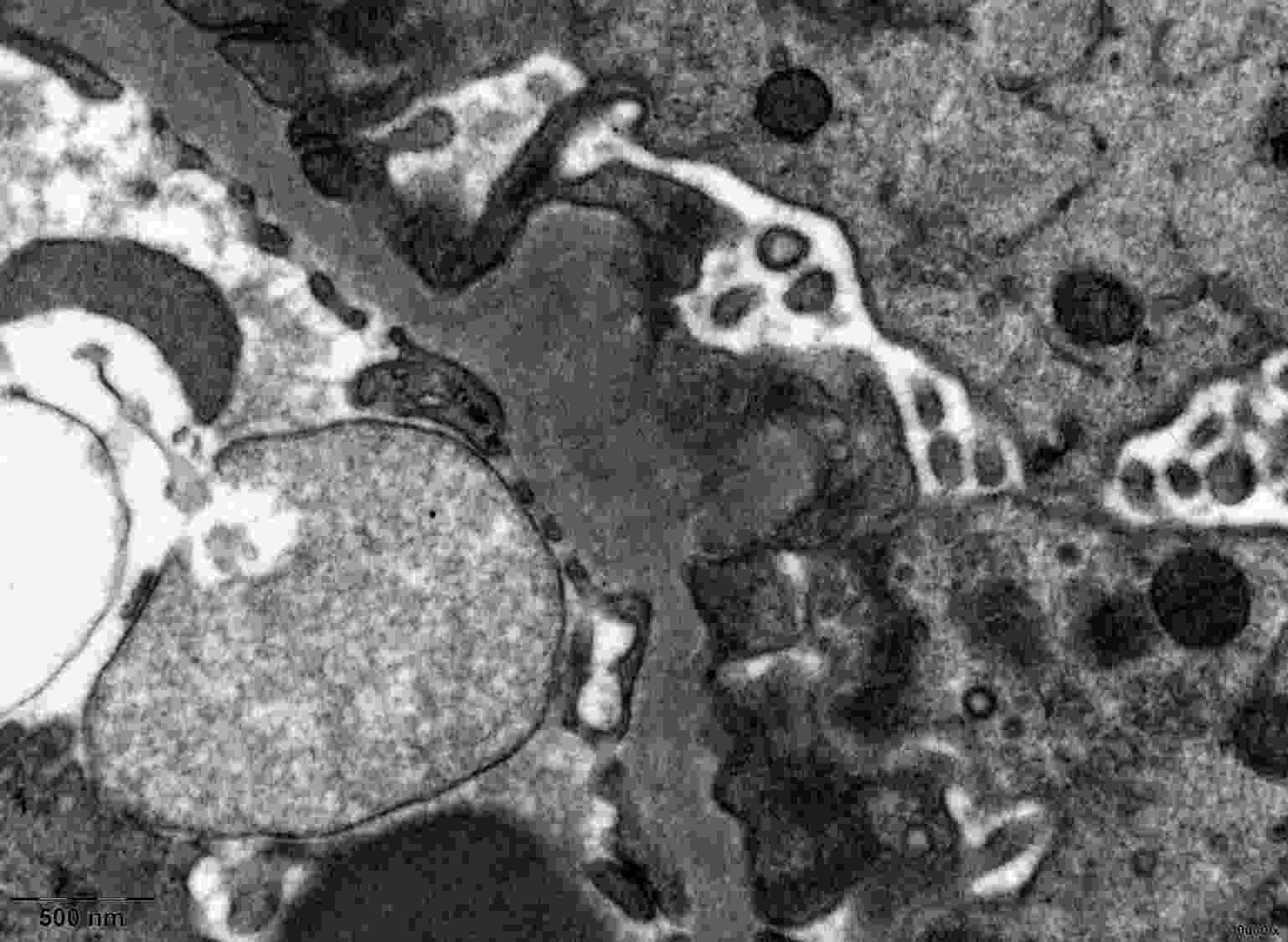

Supplement: Supplementary file 1 [file DataSheet1.zip › Original images and results for Figure 2/Fig. 2H/Fig. 2H-Electron dense deposit/PHN-5.jpg]

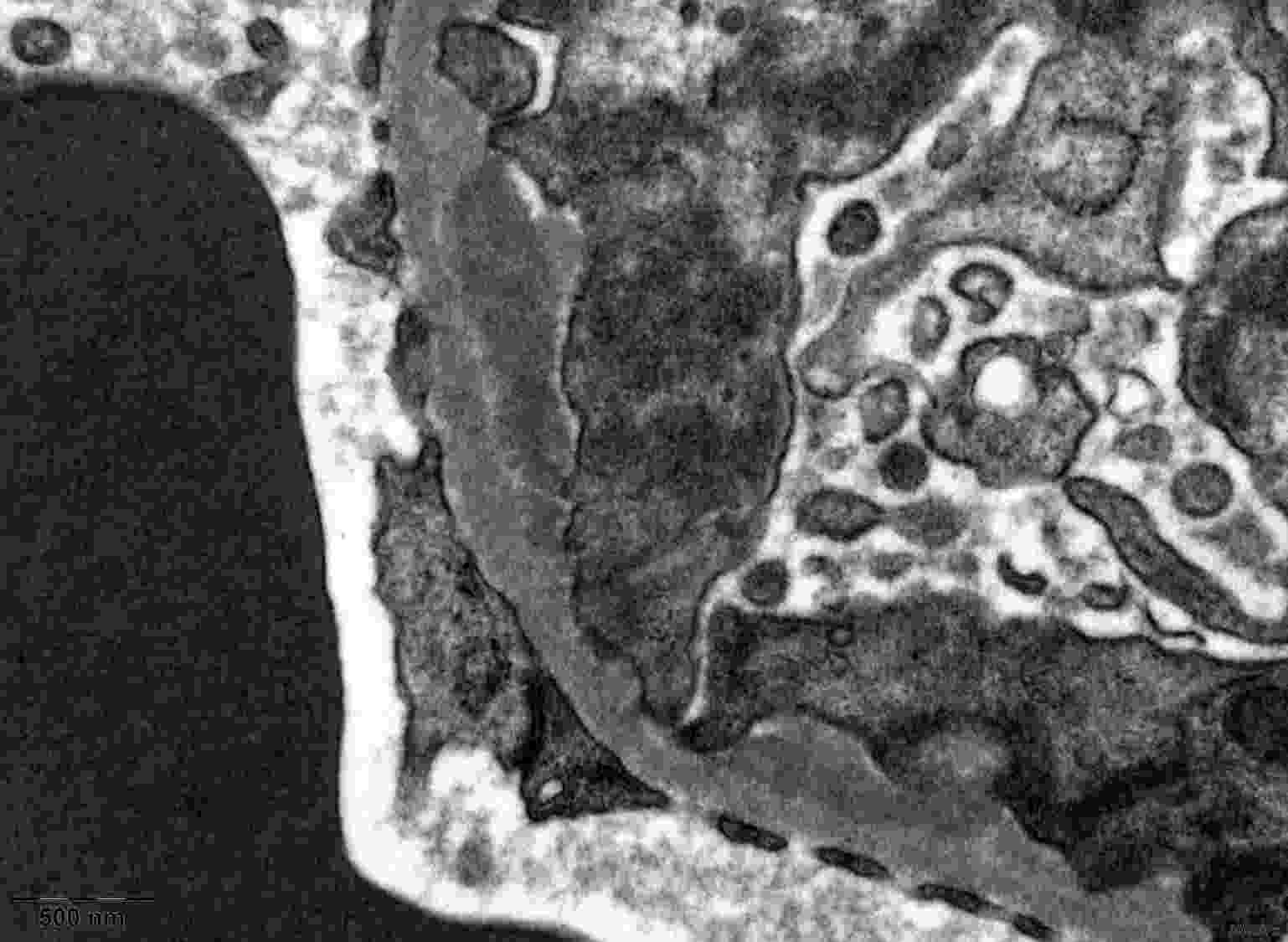

Supplement: Supplementary file 1 [file DataSheet1.zip › Original images and results for Figure 2/Fig. 2H/Fig. 2H-Electron dense deposit/PHN-6 image in Fig. 2H.jpg]

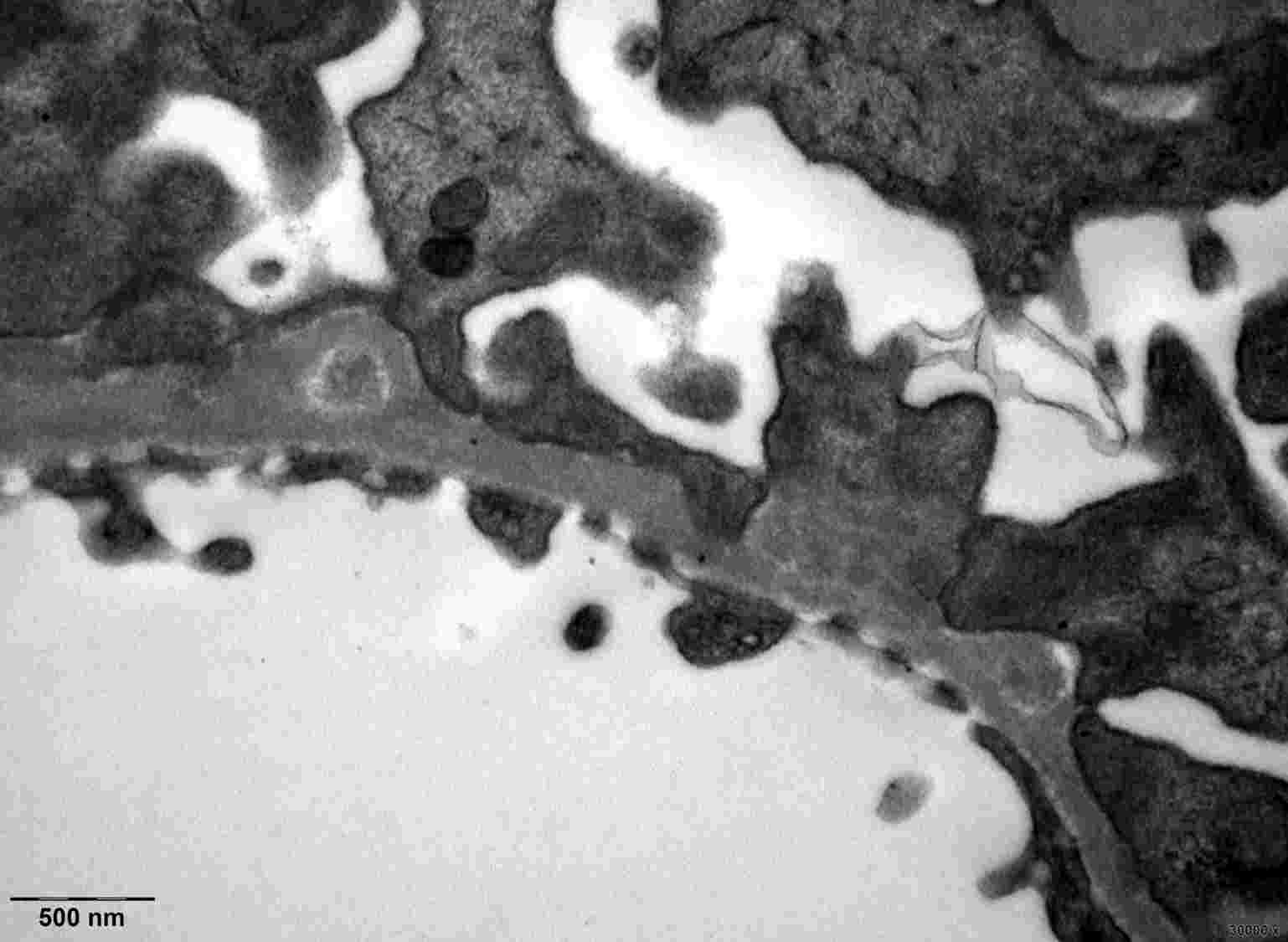

Supplement: Supplementary file 1 [file DataSheet1.zip › Original images and results for Figure 2/Fig. 2H/Fig. 2H-Electron dense deposit/SQ-H-1.jpg]

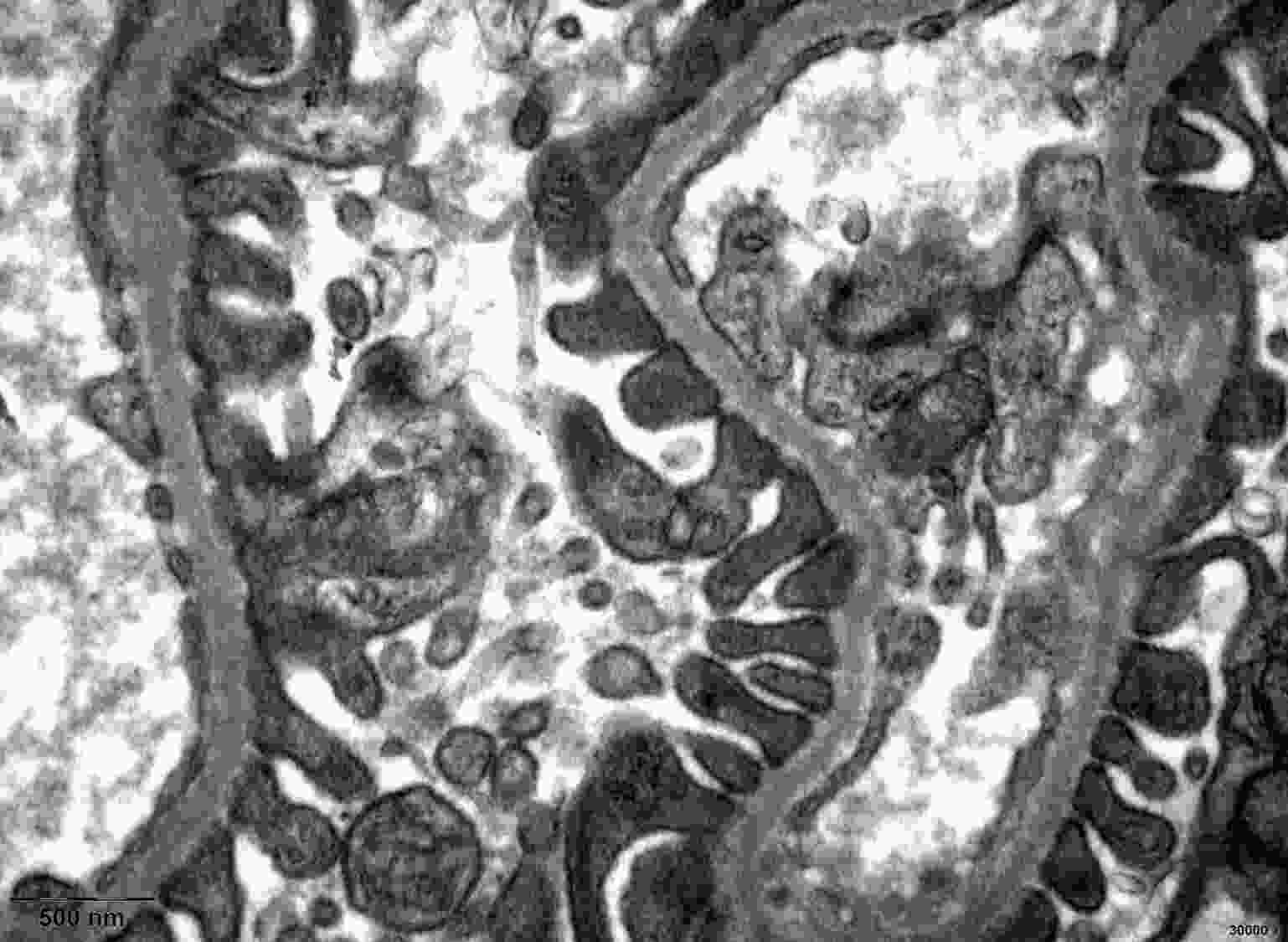

Supplement: Supplementary file 1 [file DataSheet1.zip › Original images and results for Figure 2/Fig. 2H/Fig. 2H-Electron dense deposit/SQ-H-2.jpg]

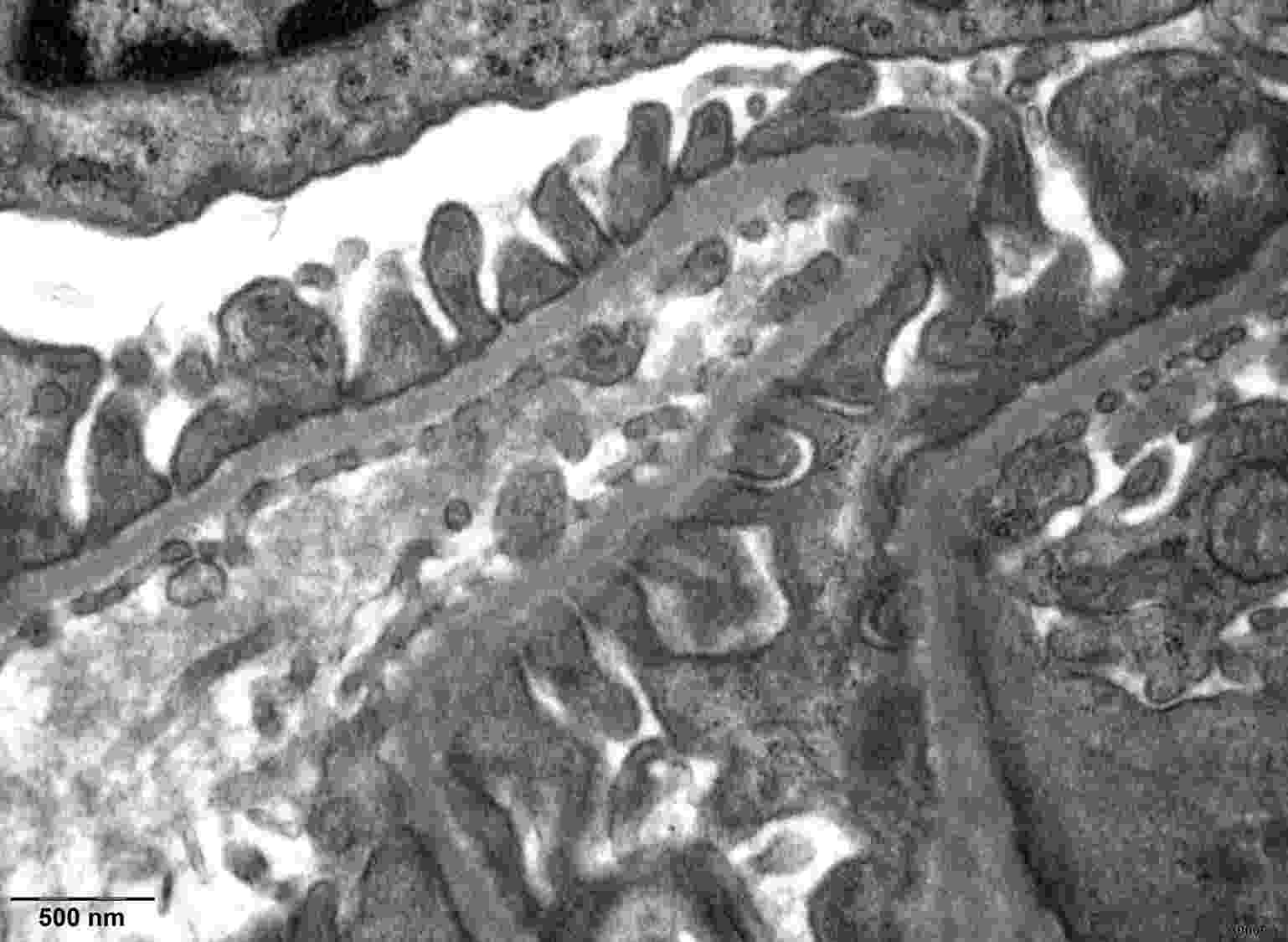

Supplement: Supplementary file 1 [file DataSheet1.zip › Original images and results for Figure 2/Fig. 2H/Fig. 2H-Electron dense deposit/SQ-H-3.jpg]

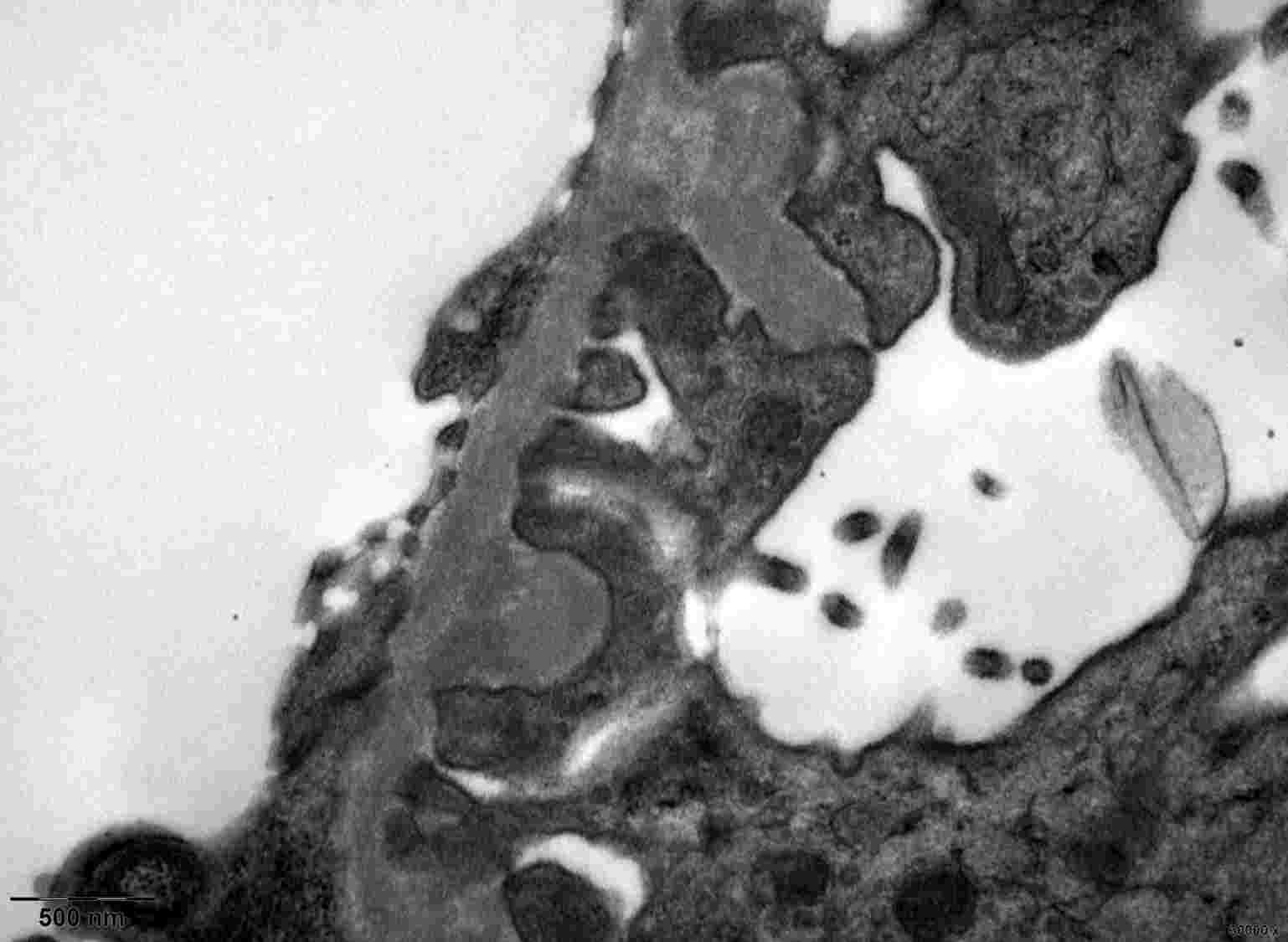

Supplement: Supplementary file 1 [file DataSheet1.zip › Original images and results for Figure 2/Fig. 2H/Fig. 2H-Electron dense deposit/SQ-H-4.jpg]

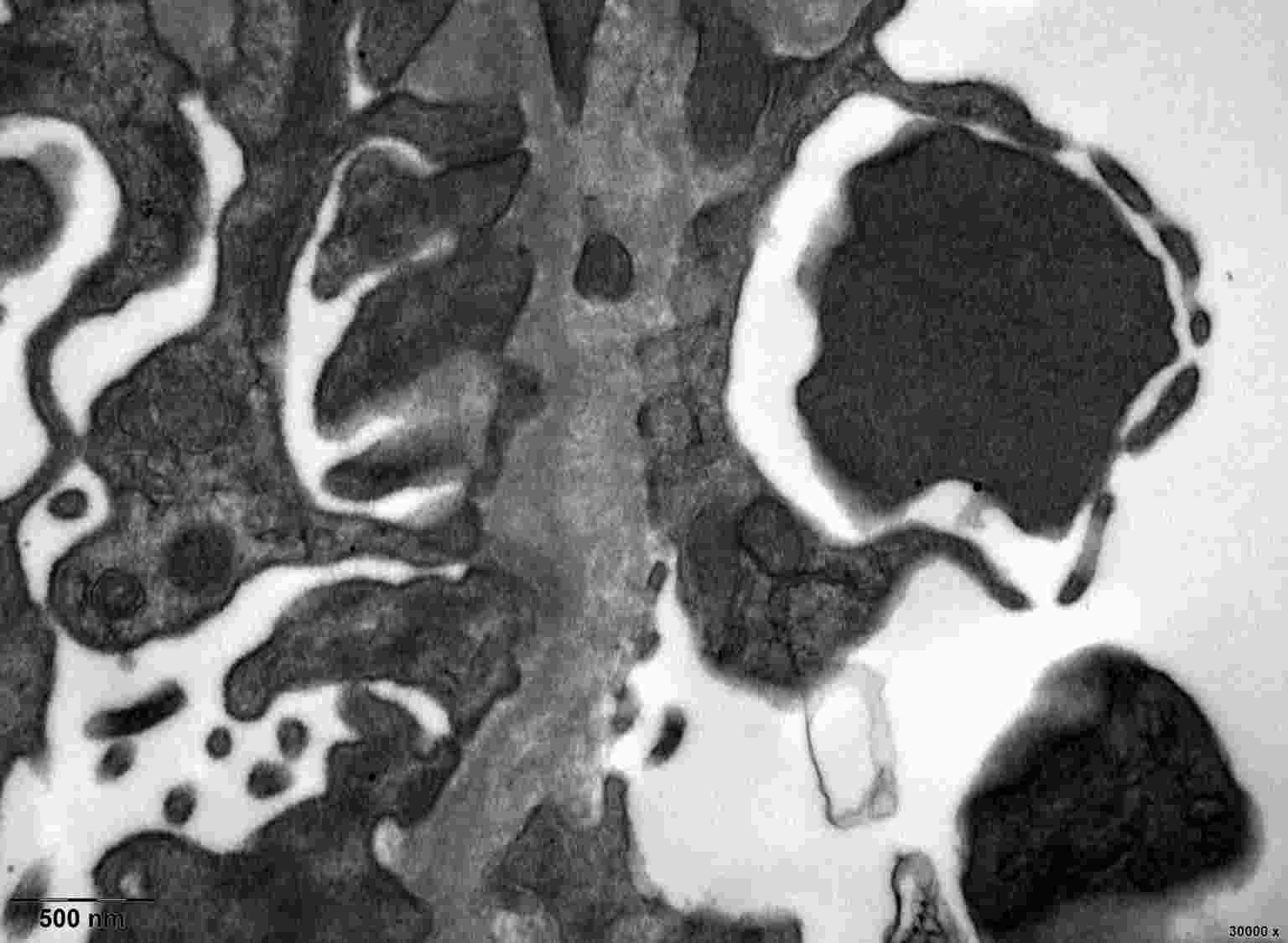

Supplement: Supplementary file 1 [file DataSheet1.zip › Original images and results for Figure 2/Fig. 2H/Fig. 2H-Electron dense deposit/SQ-H-5.jpg]

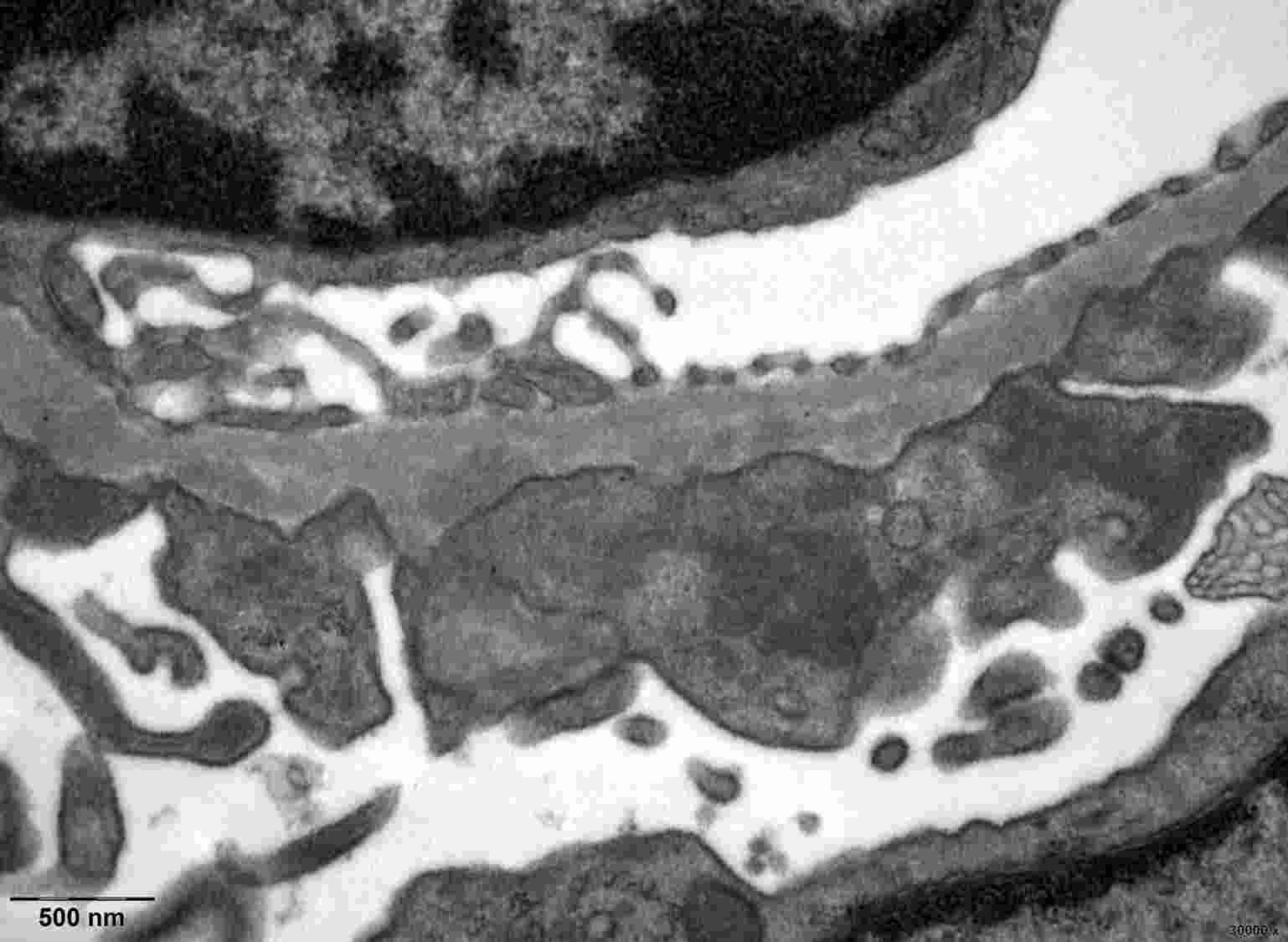

Supplement: Supplementary file 1 [file DataSheet1.zip › Original images and results for Figure 2/Fig. 2H/Fig. 2H-Electron dense deposit/SQ-H-6 image in Fig. 2H.jpg]

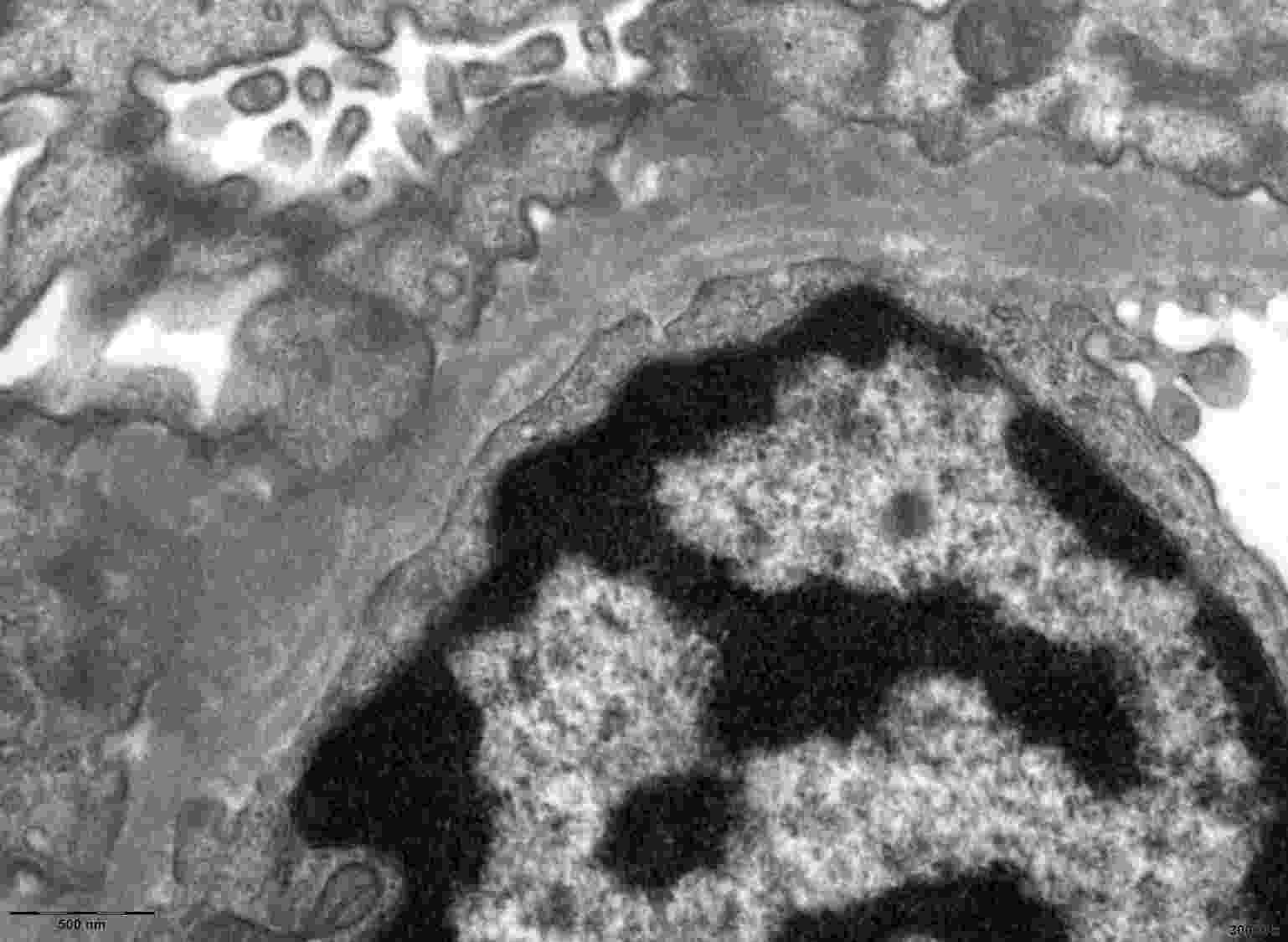

Supplement: Supplementary file 1 [file DataSheet1.zip › Original images and results for Figure 2/Fig. 2H/Fig. 2H-Electron dense deposit/SQ-L-1.jpg]

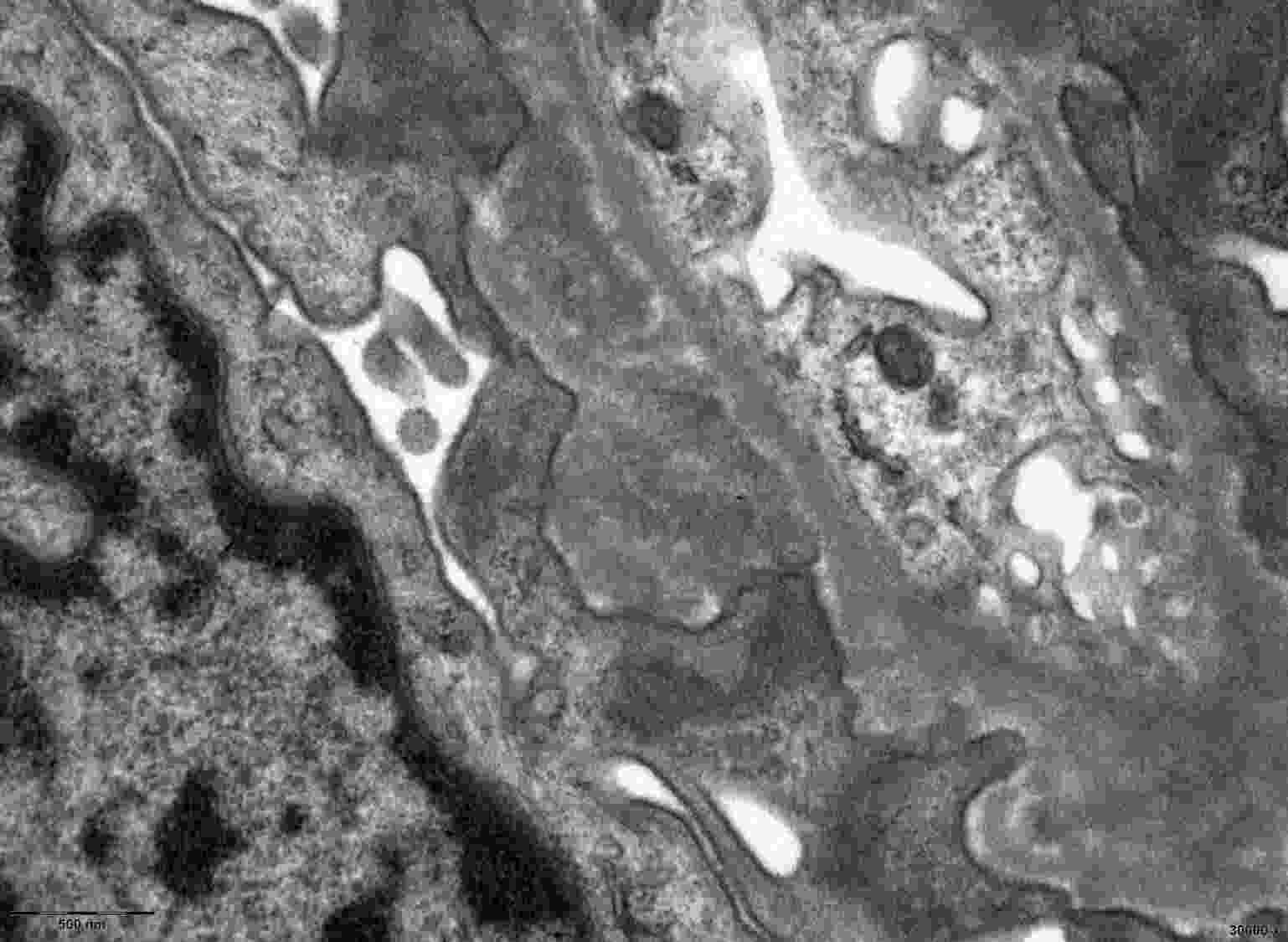

Supplement: Supplementary file 1 [file DataSheet1.zip › Original images and results for Figure 2/Fig. 2H/Fig. 2H-Electron dense deposit/SQ-L-2.jpg]

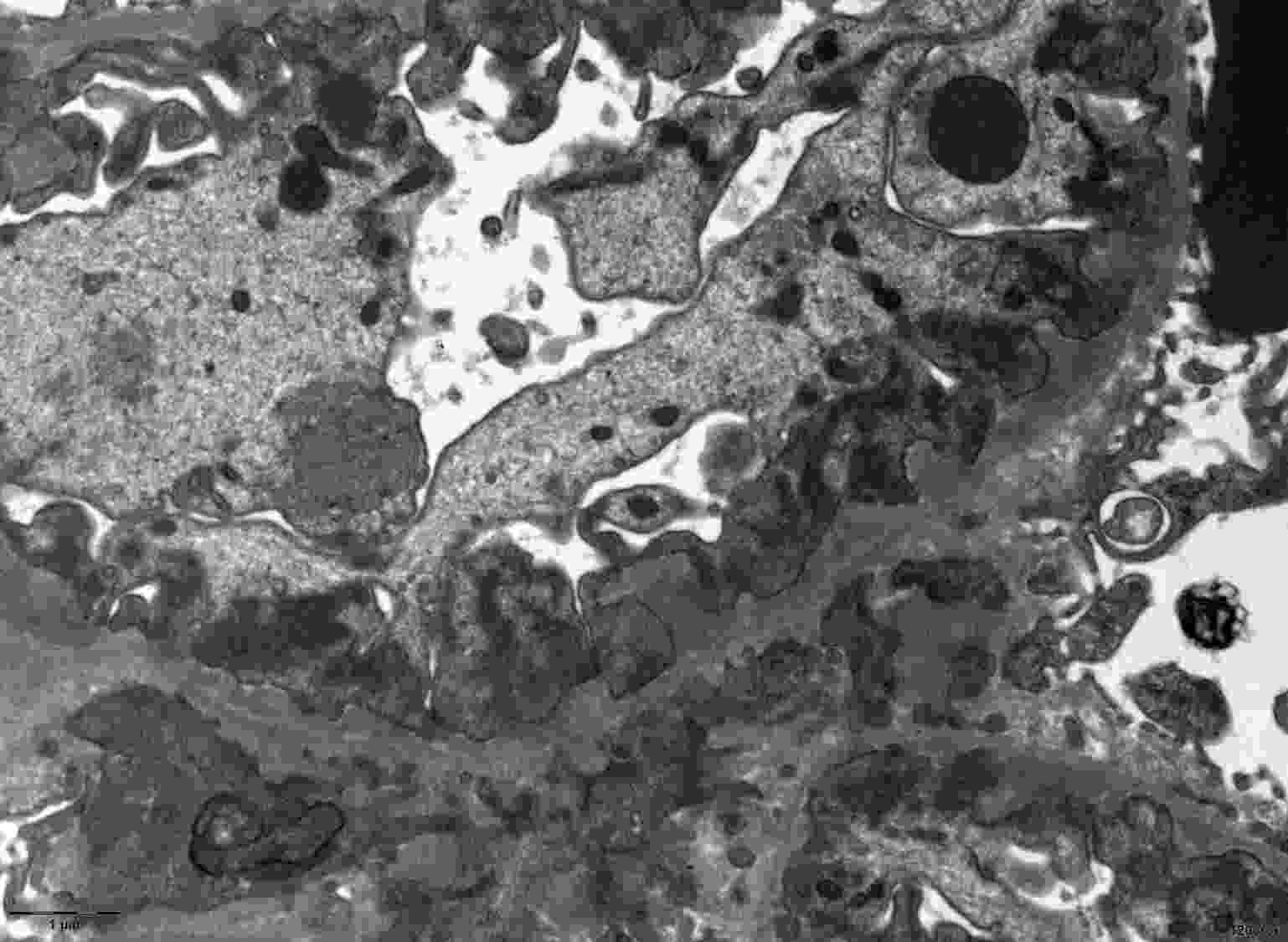

Supplement: Supplementary file 1 [file DataSheet1.zip › Original images and results for Figure 2/Fig. 2H/Fig. 2H-Electron dense deposit/SQ-L-3.jpg]

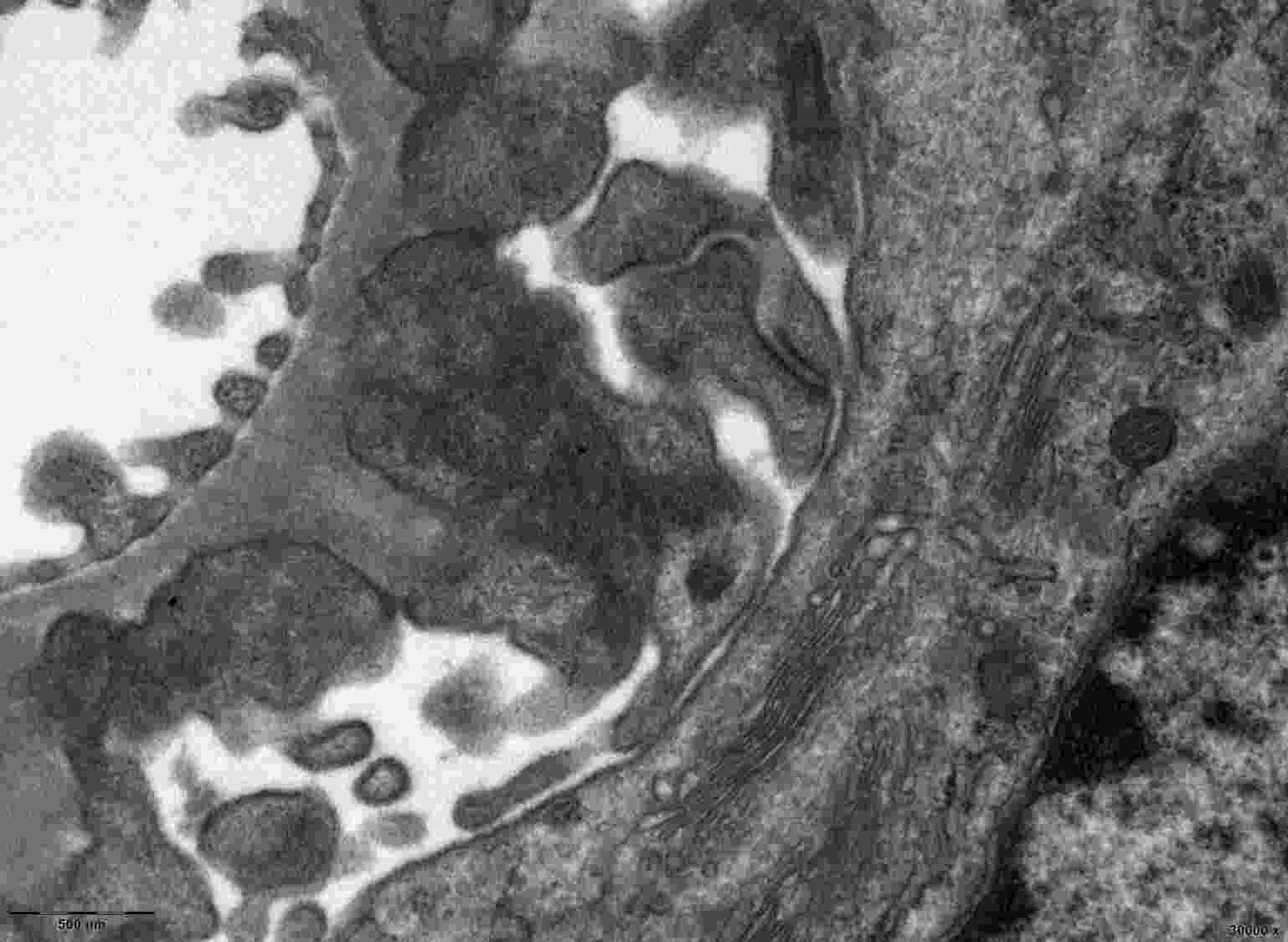

Supplement: Supplementary file 1 [file DataSheet1.zip › Original images and results for Figure 2/Fig. 2H/Fig. 2H-Electron dense deposit/SQ-L-4.jpg]

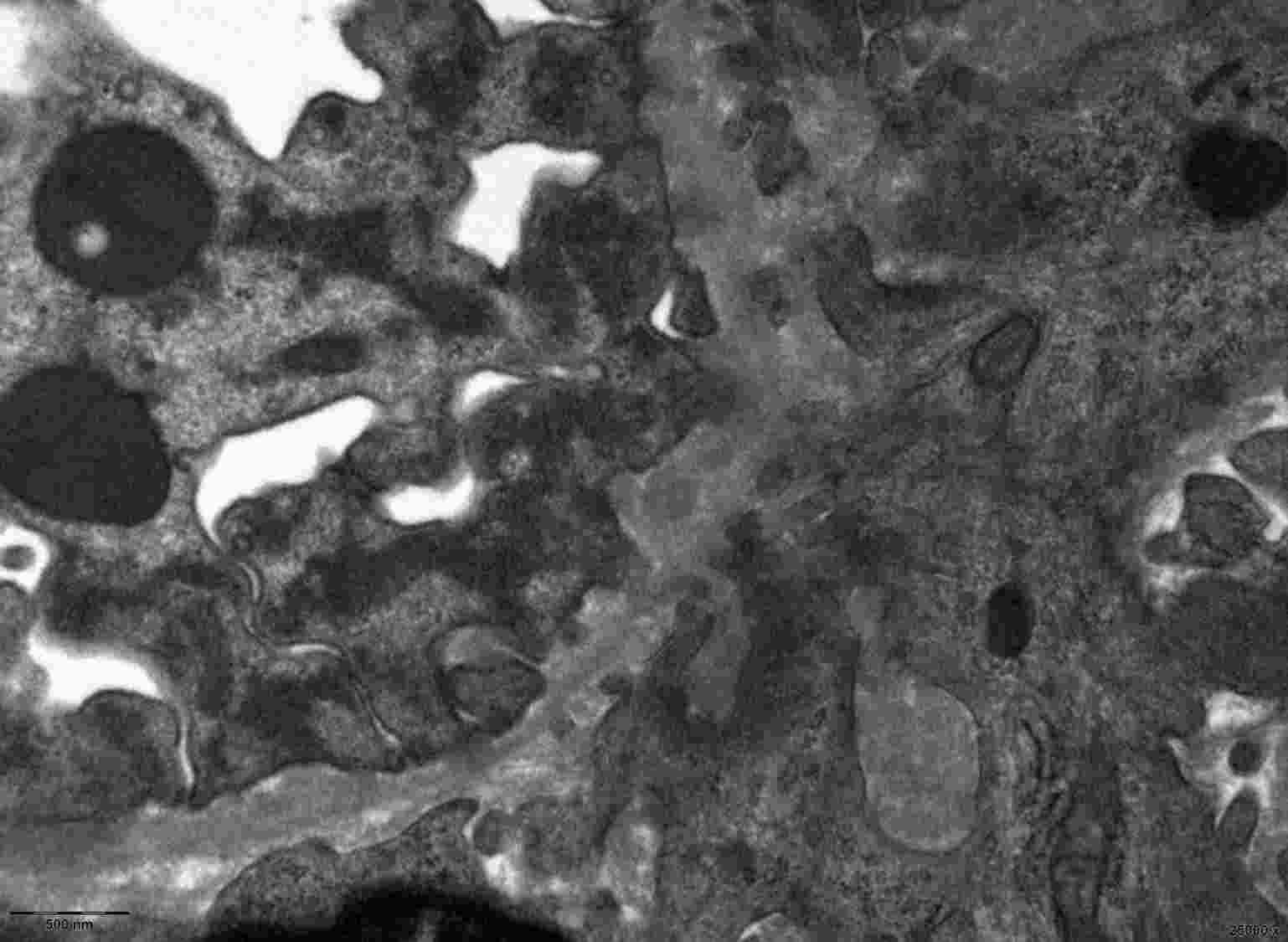

Supplement: Supplementary file 1 [file DataSheet1.zip › Original images and results for Figure 2/Fig. 2H/Fig. 2H-Electron dense deposit/SQ-L-5.jpg]

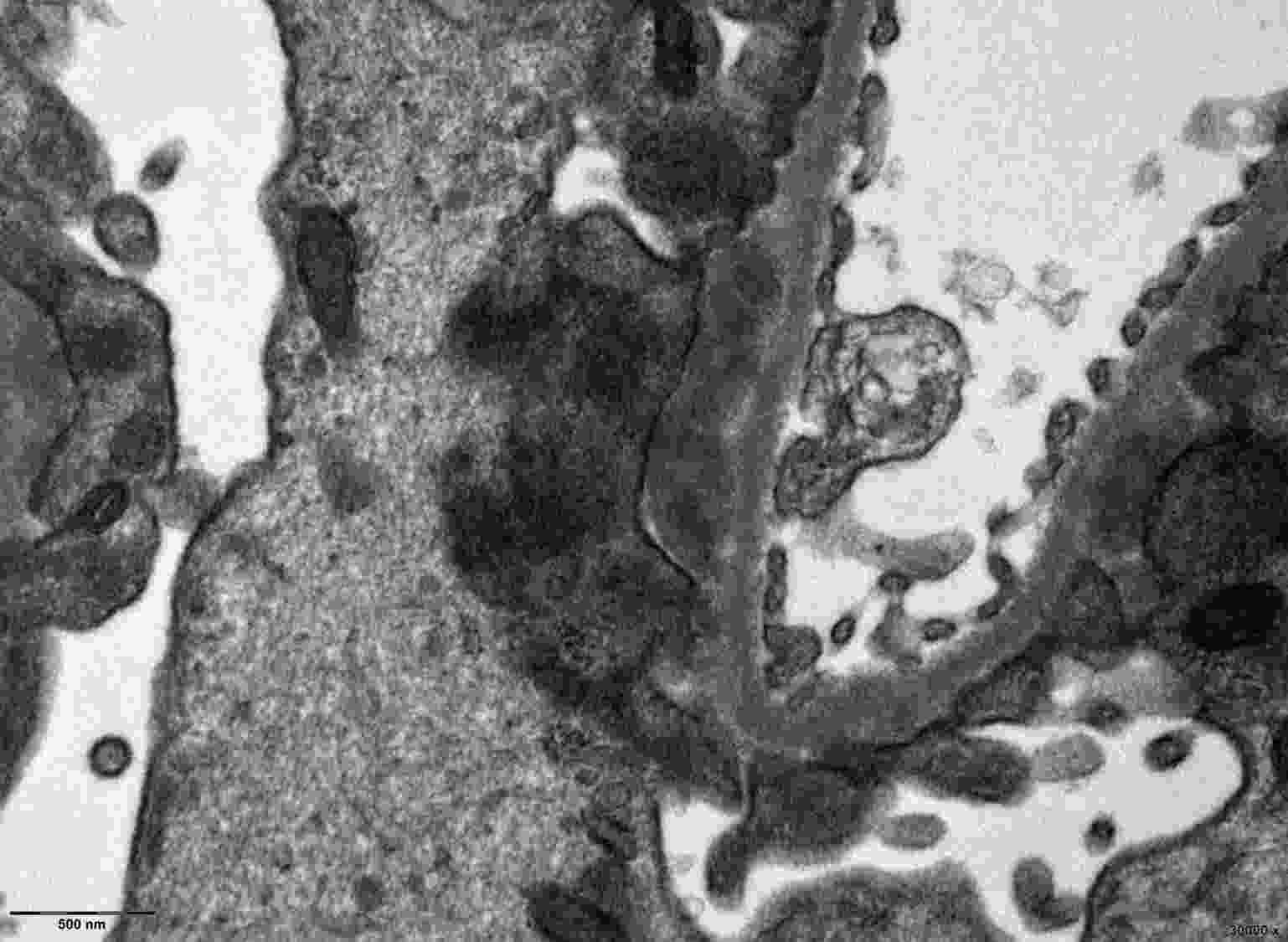

Supplement: Supplementary file 1 [file DataSheet1.zip › Original images and results for Figure 2/Fig. 2H/Fig. 2H-Electron dense deposit/SQ-L-6 image in Fig. 2H.jpg]

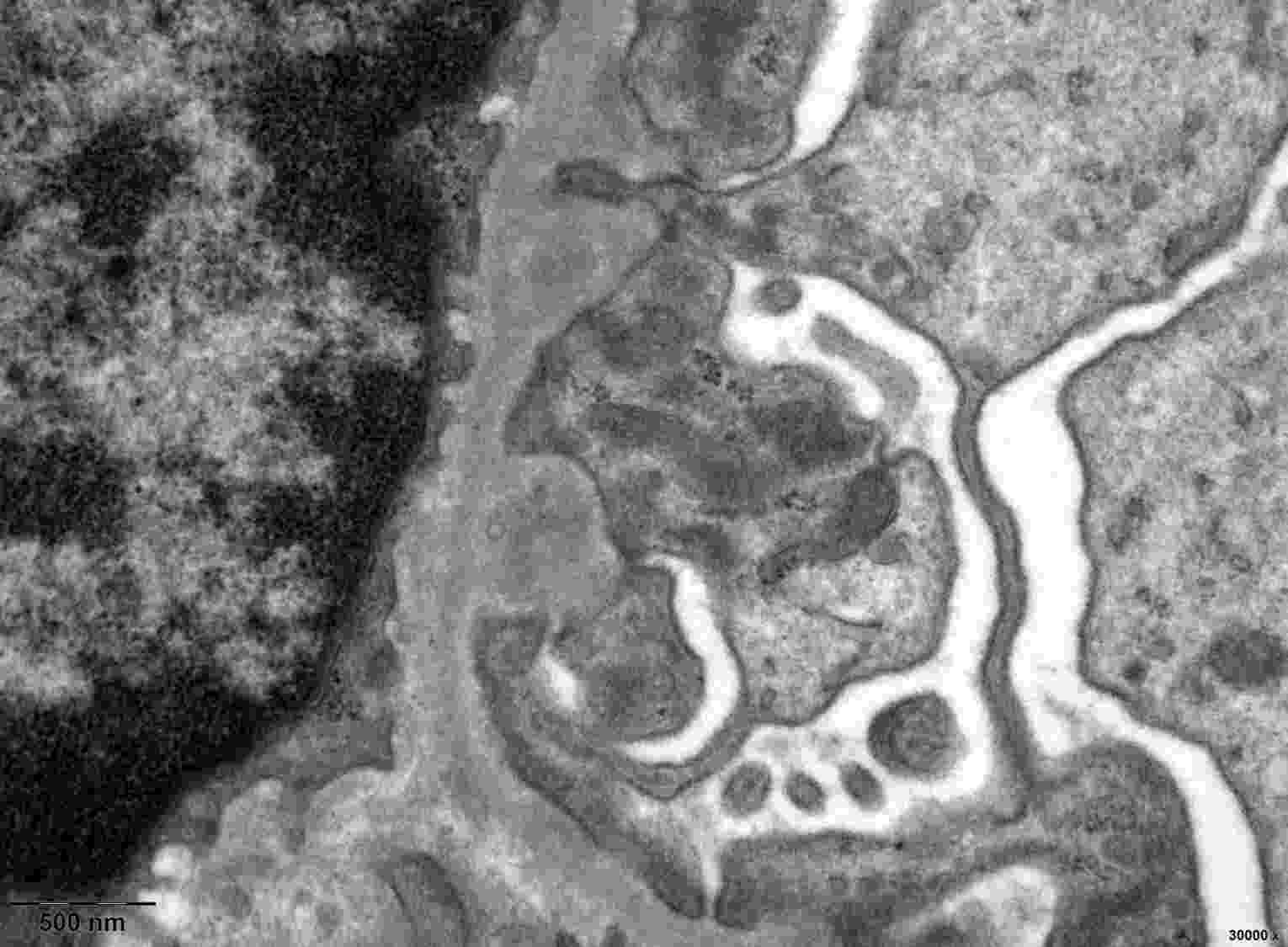

Supplement: Supplementary file 1 [file DataSheet1.zip › Original images and results for Figure 2/Fig. 2H/Fig. 2H-Electron dense deposit/TAC-1.jpg]

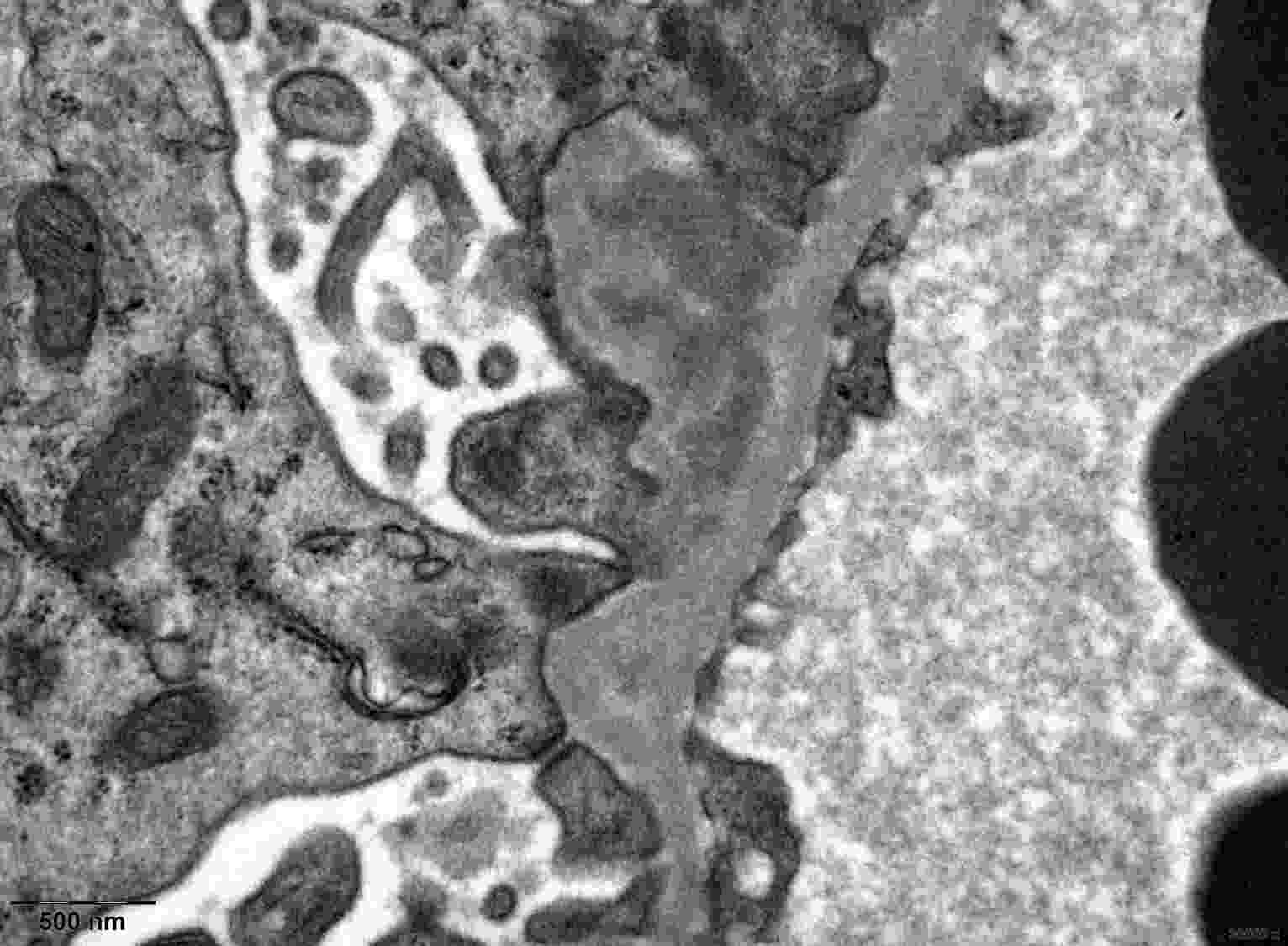

Supplement: Supplementary file 1 [file DataSheet1.zip › Original images and results for Figure 2/Fig. 2H/Fig. 2H-Electron dense deposit/TAC-2.jpg]

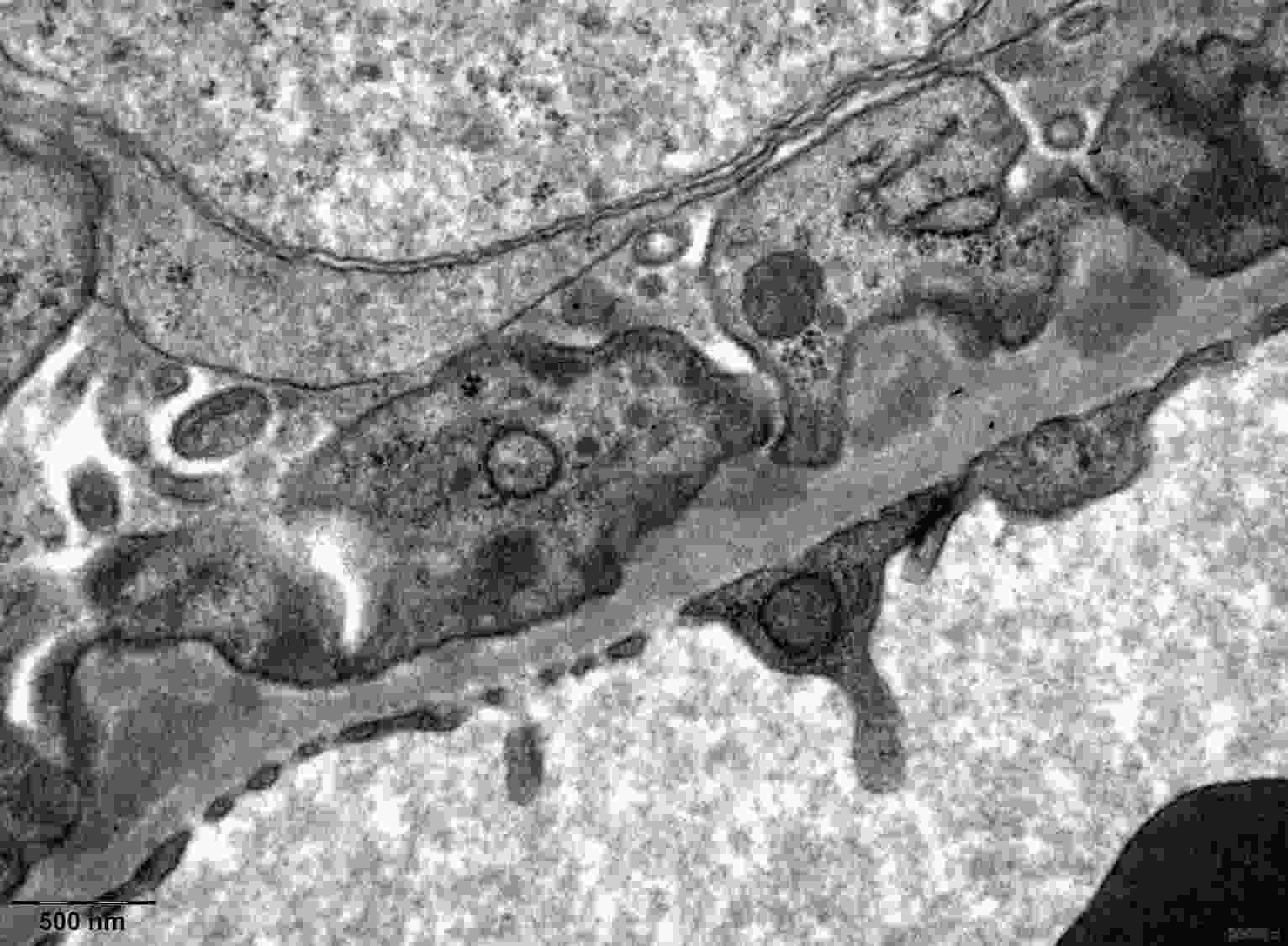

Supplement: Supplementary file 1 [file DataSheet1.zip › Original images and results for Figure 2/Fig. 2H/Fig. 2H-Electron dense deposit/TAC-3.jpg]

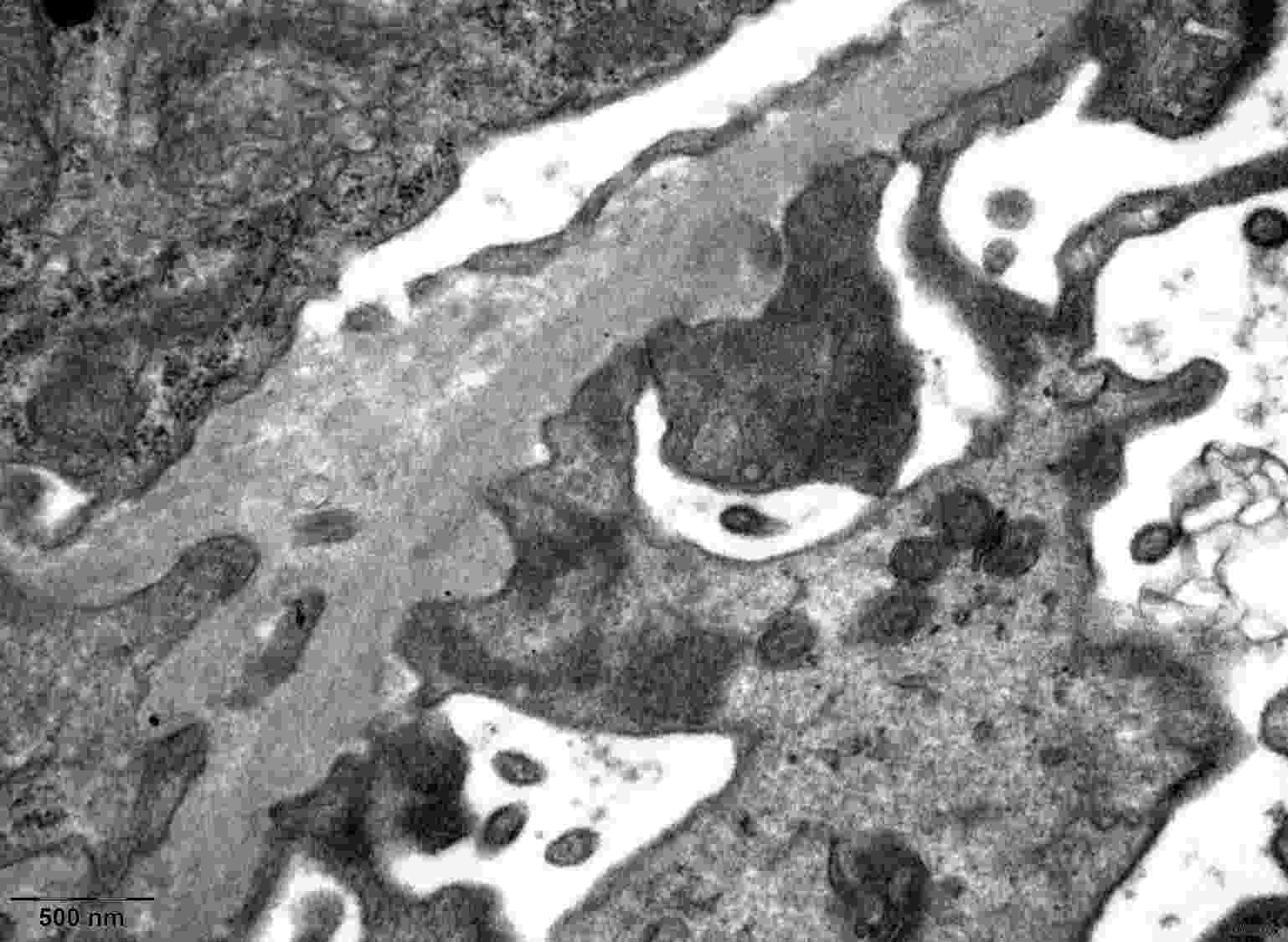

Supplement: Supplementary file 1 [file DataSheet1.zip › Original images and results for Figure 2/Fig. 2H/Fig. 2H-Electron dense deposit/TAC-4.jpg]

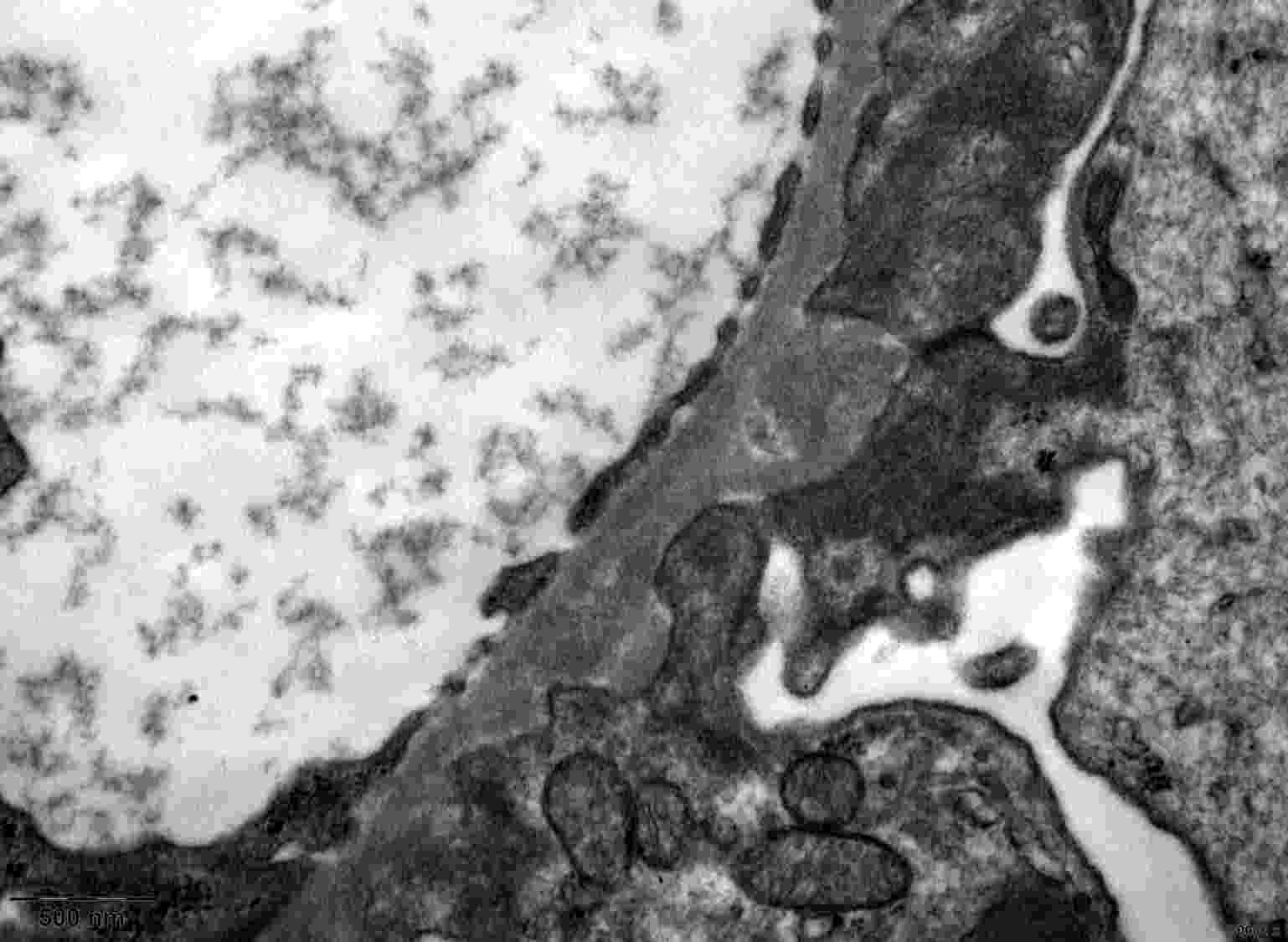

Supplement: Supplementary file 1 [file DataSheet1.zip › Original images and results for Figure 2/Fig. 2H/Fig. 2H-Electron dense deposit/TAC-5 image in Fig. 2H.jpg]

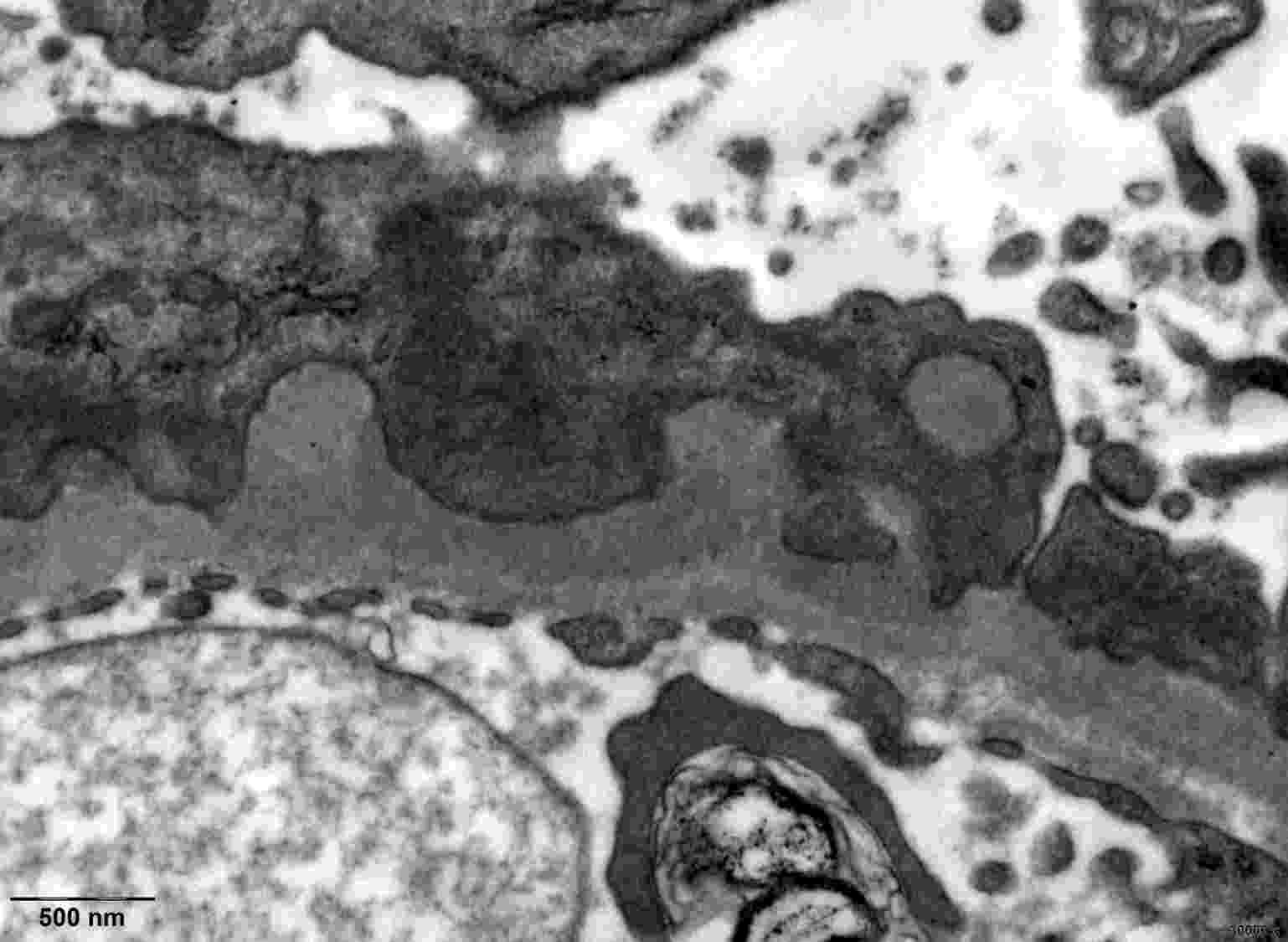

Supplement: Supplementary file 1 [file DataSheet1.zip › Original images and results for Figure 2/Fig. 2H/Fig. 2H-Electron dense deposit/TAC-6.jpg]

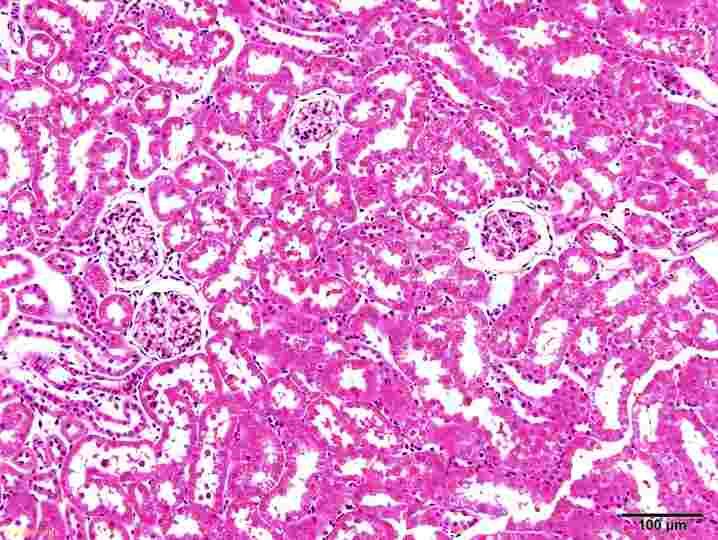

Supplement: Supplementary file 1 [file DataSheet1.zip › Original images and results for Figure 2/Fig. 2H/Fig. 2H-HE/HE-CON/1-1.jpg]

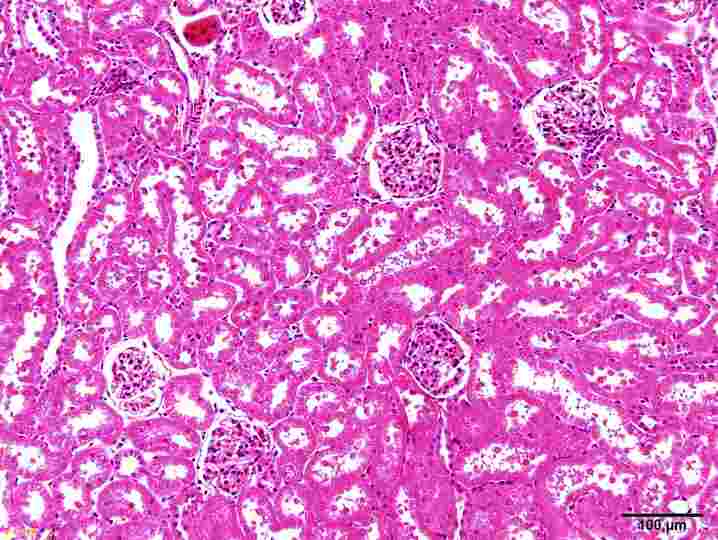

Supplement: Supplementary file 1 [file DataSheet1.zip › Original images and results for Figure 2/Fig. 2H/Fig. 2H-HE/HE-CON/1-2.jpg]

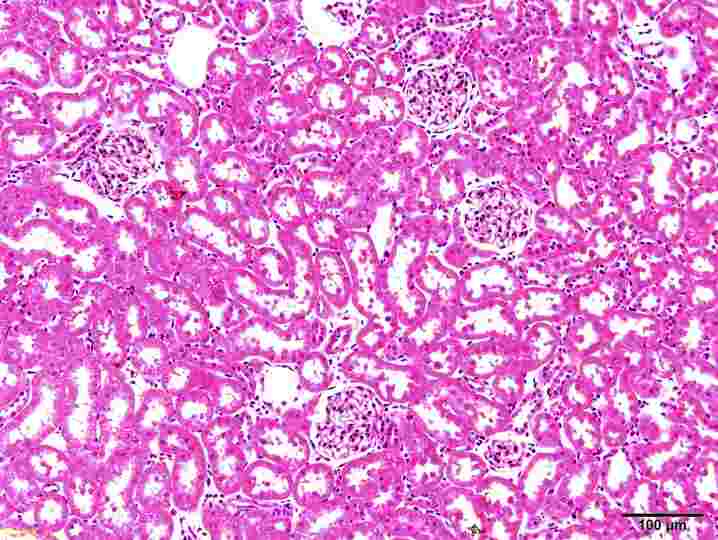

Supplement: Supplementary file 1 [file DataSheet1.zip › Original images and results for Figure 2/Fig. 2H/Fig. 2H-HE/HE-CON/1-3.jpg]

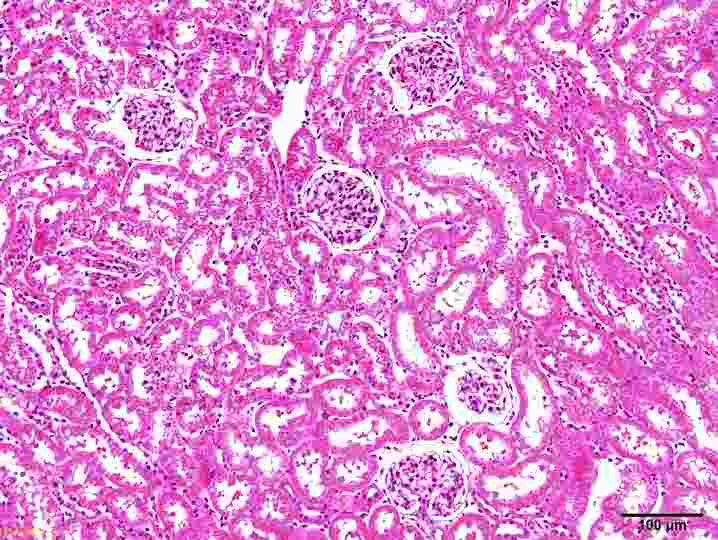

Supplement: Supplementary file 1 [file DataSheet1.zip › Original images and results for Figure 2/Fig. 2H/Fig. 2H-HE/HE-CON/1-4.jpg]

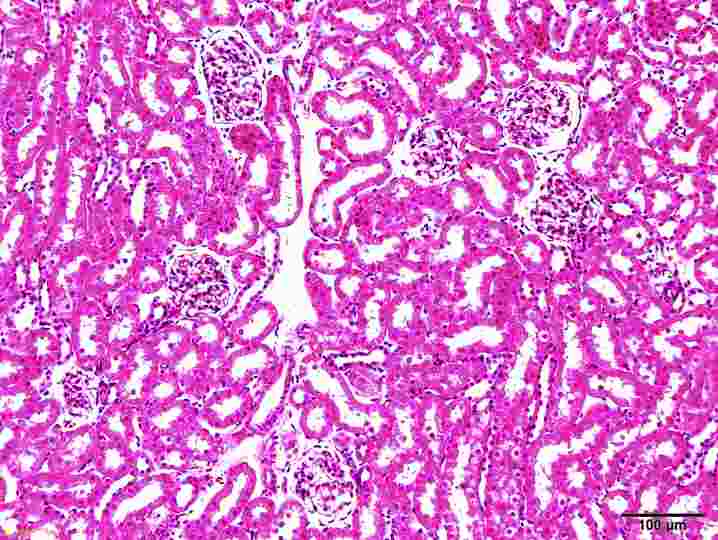

Supplement: Supplementary file 1 [file DataSheet1.zip › Original images and results for Figure 2/Fig. 2H/Fig. 2H-HE/HE-CON/1-5.jpg]

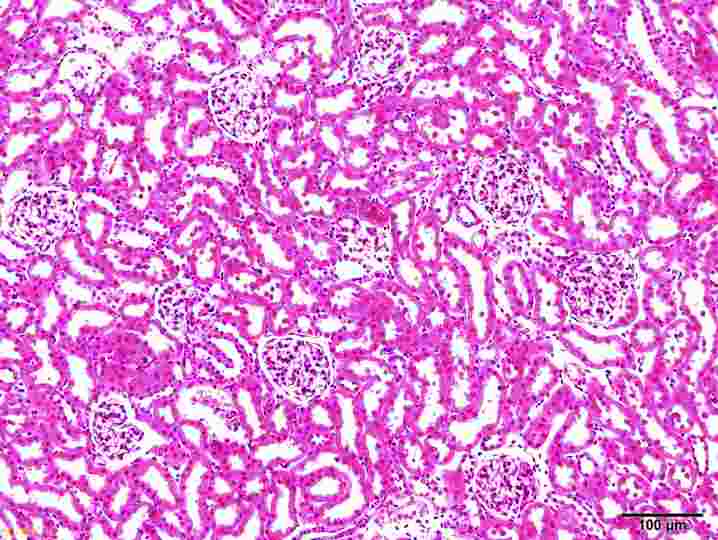

Supplement: Supplementary file 1 [file DataSheet1.zip › Original images and results for Figure 2/Fig. 2H/Fig. 2H-HE/HE-CON/2-1.jpg]

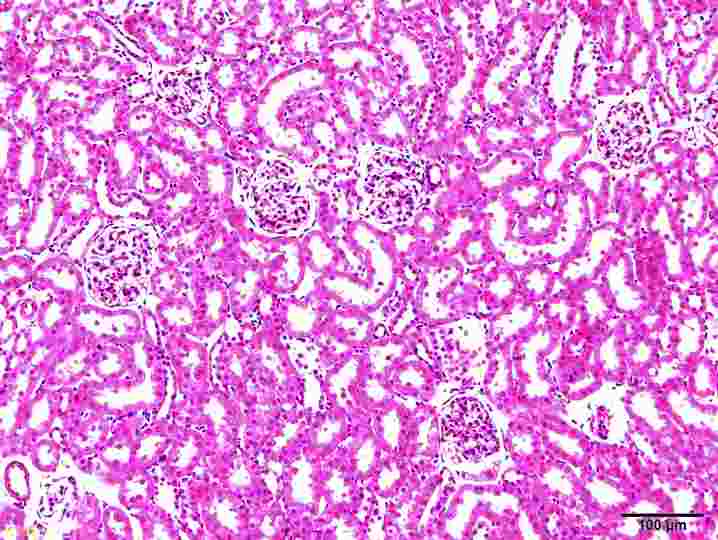

Supplement: Supplementary file 1 [file DataSheet1.zip › Original images and results for Figure 2/Fig. 2H/Fig. 2H-HE/HE-CON/2-2.jpg]

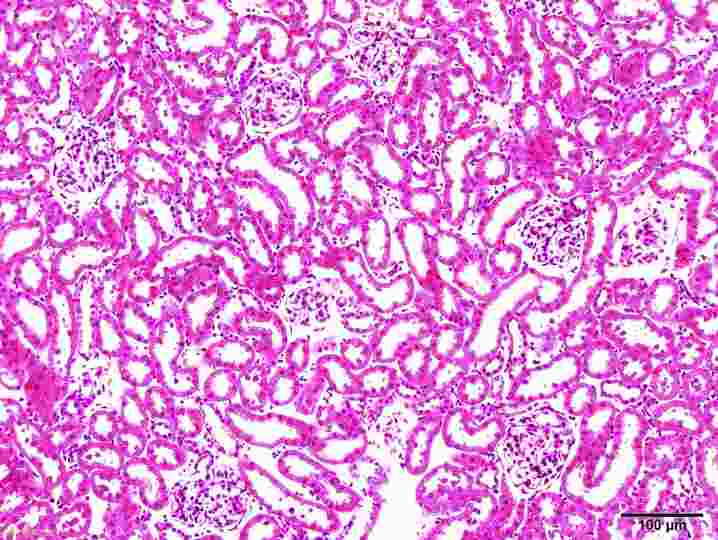

Supplement: Supplementary file 1 [file DataSheet1.zip › Original images and results for Figure 2/Fig. 2H/Fig. 2H-HE/HE-CON/2-4.jpg]

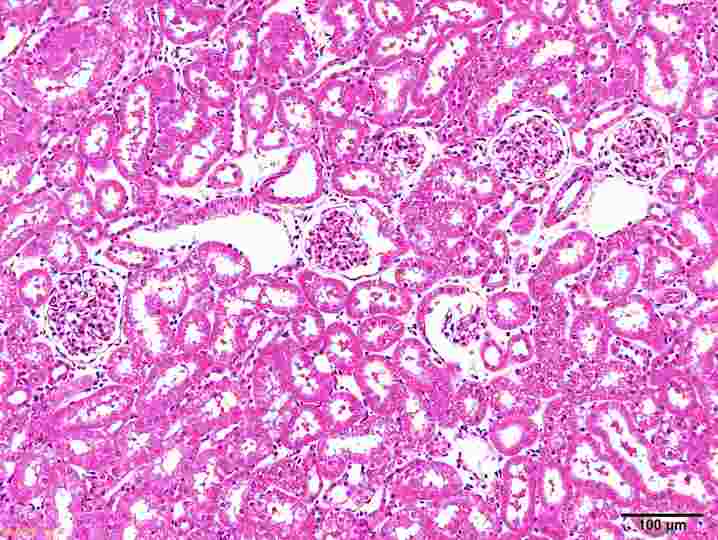

Supplement: Supplementary file 1 [file DataSheet1.zip › Original images and results for Figure 2/Fig. 2H/Fig. 2H-HE/HE-CON/2-5.jpg]

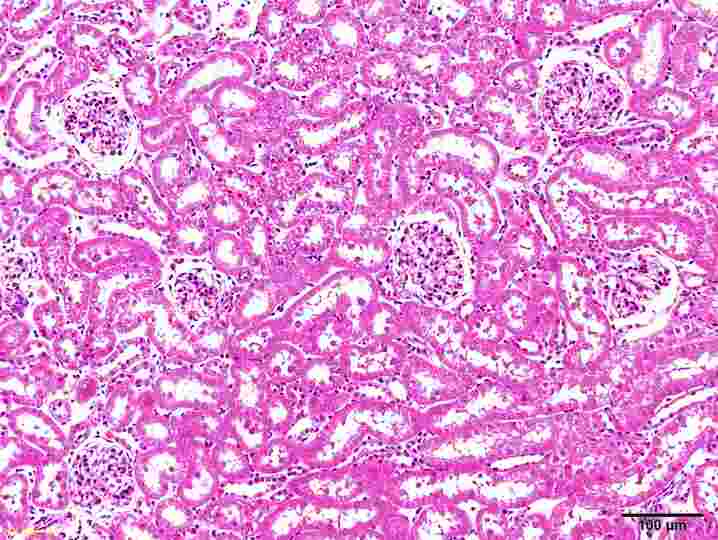

Supplement: Supplementary file 1 [file DataSheet1.zip › Original images and results for Figure 2/Fig. 2H/Fig. 2H-HE/HE-CON/3-1.jpg]

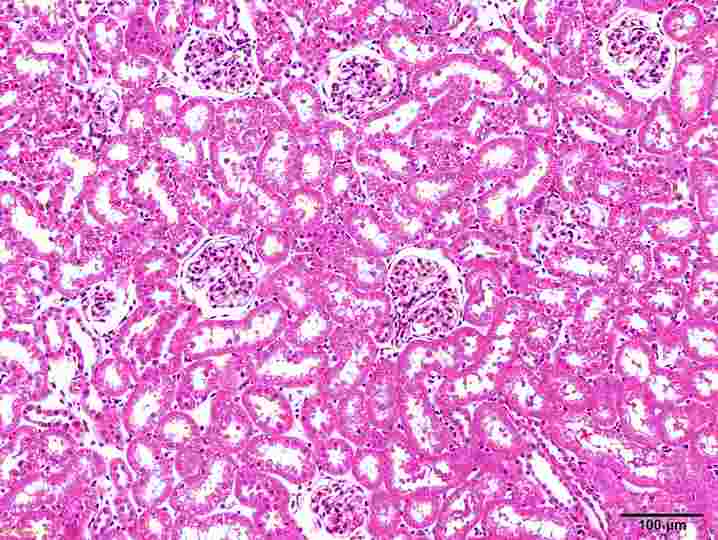

Supplement: Supplementary file 1 [file DataSheet1.zip › Original images and results for Figure 2/Fig. 2H/Fig. 2H-HE/HE-CON/3-3 image in Fig. 1H-HE.jpg]

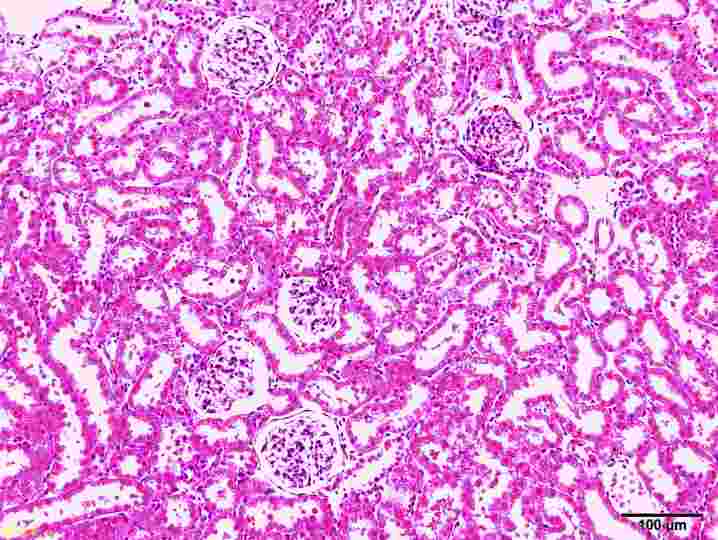

Supplement: Supplementary file 1 [file DataSheet1.zip › Original images and results for Figure 2/Fig. 2H/Fig. 2H-HE/HE-CON/3-4.jpg]

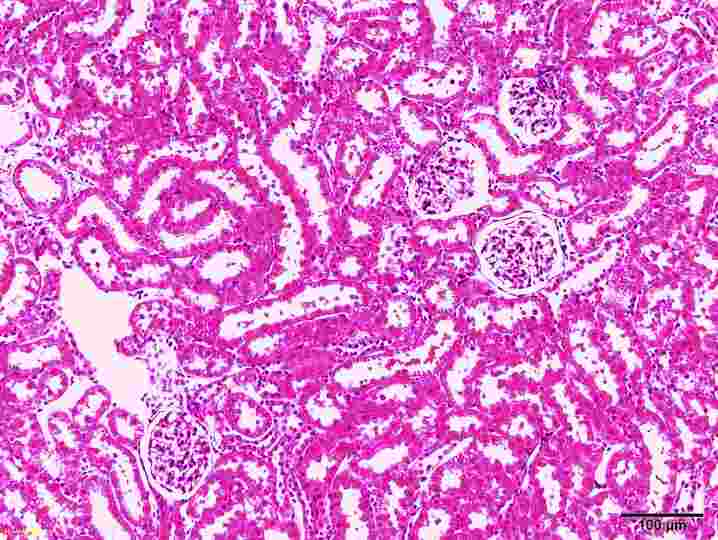

Supplement: Supplementary file 1 [file DataSheet1.zip › Original images and results for Figure 2/Fig. 2H/Fig. 2H-HE/HE-CON/3-5.jpg]

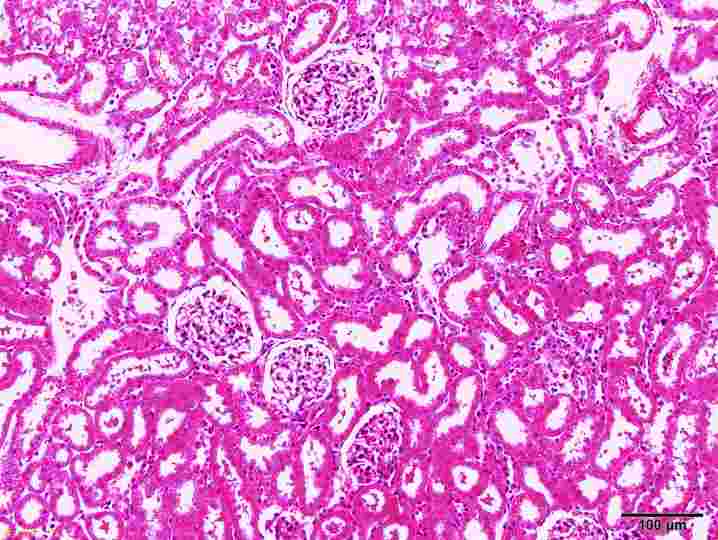

Supplement: Supplementary file 1 [file DataSheet1.zip › Original images and results for Figure 2/Fig. 2H/Fig. 2H-HE/HE-CON/4-1.jpg]

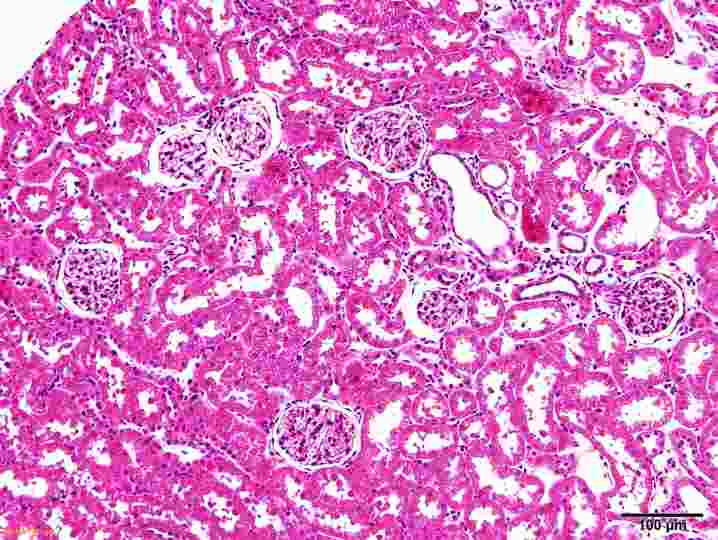

Supplement: Supplementary file 1 [file DataSheet1.zip › Original images and results for Figure 2/Fig. 2H/Fig. 2H-HE/HE-CON/4-2.jpg]

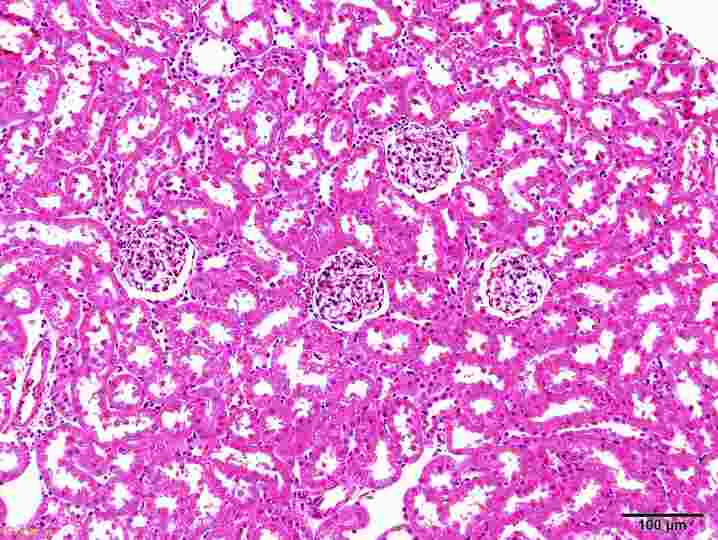

Supplement: Supplementary file 1 [file DataSheet1.zip › Original images and results for Figure 2/Fig. 2H/Fig. 2H-HE/HE-CON/4-4.jpg]

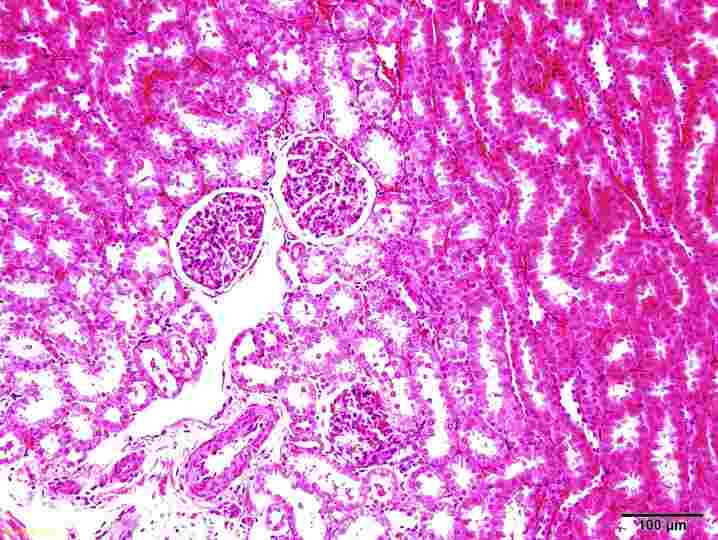

Supplement: Supplementary file 1 [file DataSheet1.zip › Original images and results for Figure 2/Fig. 2H/Fig. 2H-HE/HE-CON/5-1.jpg]

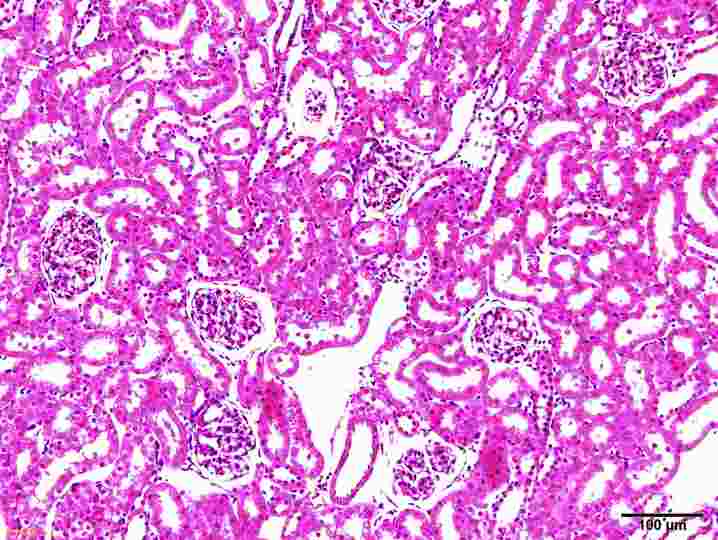

Supplement: Supplementary file 1 [file DataSheet1.zip › Original images and results for Figure 2/Fig. 2H/Fig. 2H-HE/HE-CON/5-3.jpg]

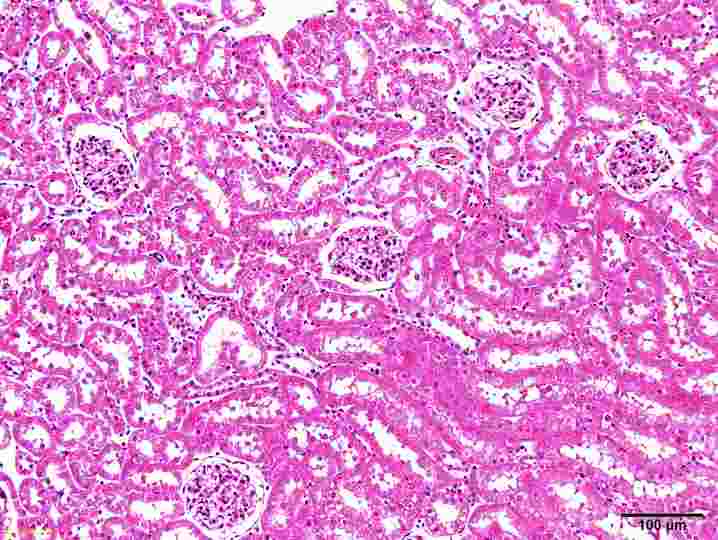

Supplement: Supplementary file 1 [file DataSheet1.zip › Original images and results for Figure 2/Fig. 2H/Fig. 2H-HE/HE-CON/6-1.jpg]

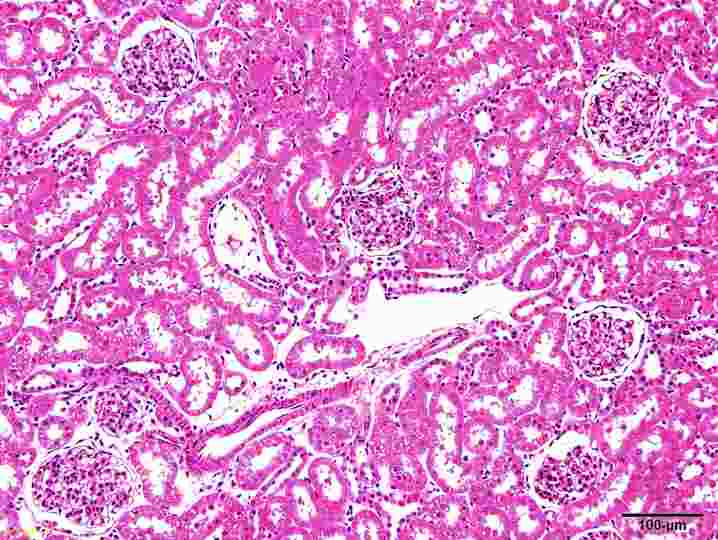

Supplement: Supplementary file 1 [file DataSheet1.zip › Original images and results for Figure 2/Fig. 2H/Fig. 2H-HE/HE-CON/6-3.jpg]

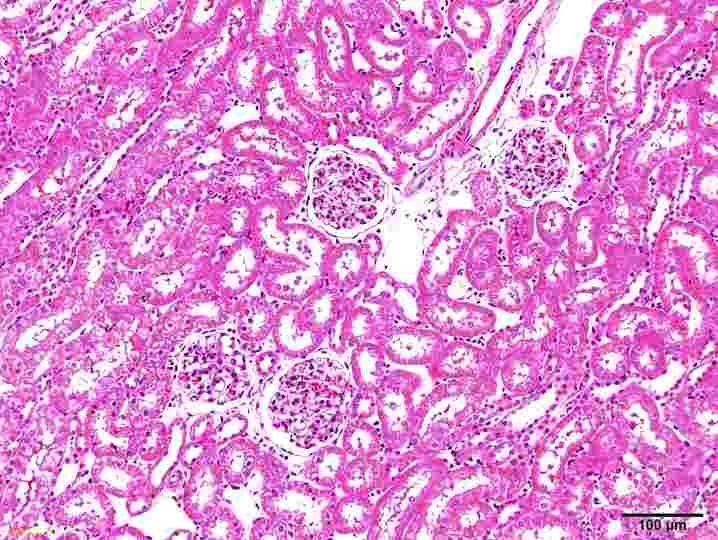

Supplement: Supplementary file 1 [file DataSheet1.zip › Original images and results for Figure 2/Fig. 2H/Fig. 2H-HE/HE-CON/6-5.jpg]

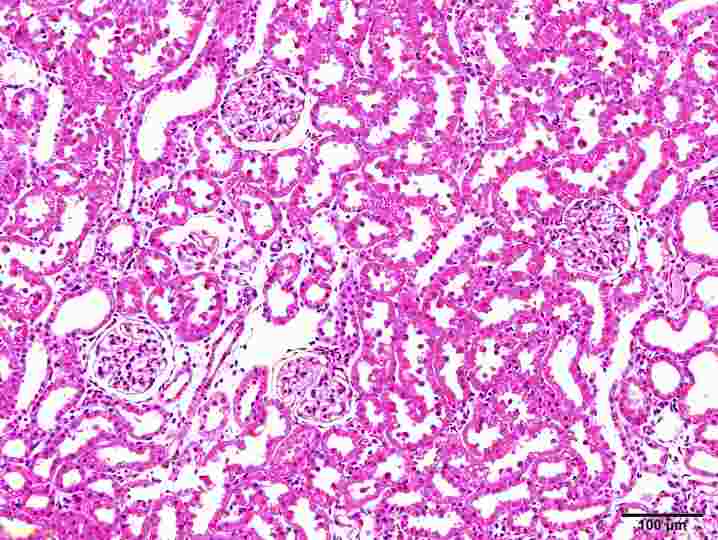

Supplement: Supplementary file 1 [file DataSheet1.zip › Original images and results for Figure 2/Fig. 2H/Fig. 2H-HE/HE-PHN/1-1.jpg]

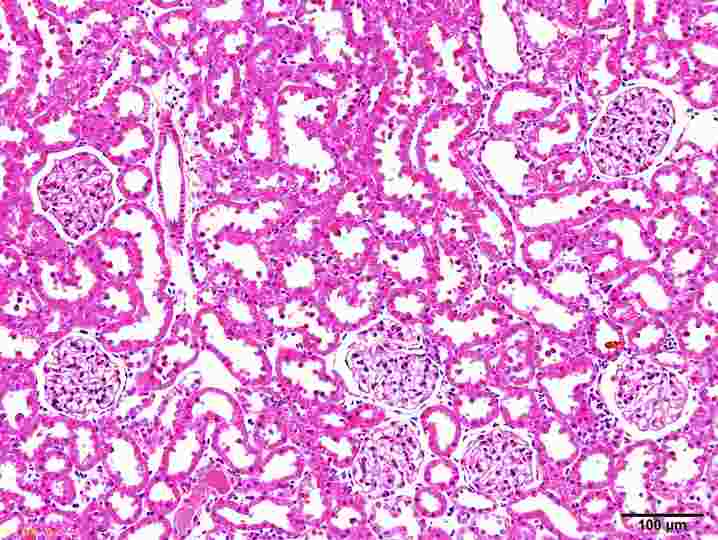

Supplement: Supplementary file 1 [file DataSheet1.zip › Original images and results for Figure 2/Fig. 2H/Fig. 2H-HE/HE-PHN/1-2 image in Fig. 1H-HE.jpg]

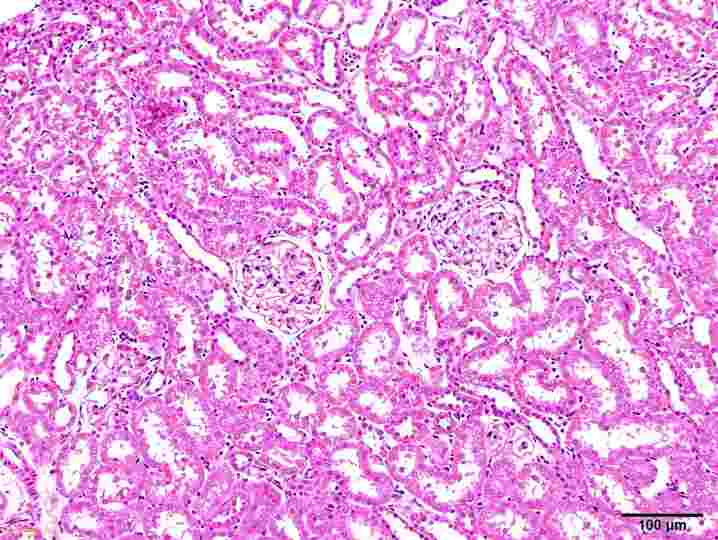

Supplement: Supplementary file 1 [file DataSheet1.zip › Original images and results for Figure 2/Fig. 2H/Fig. 2H-HE/HE-PHN/1-3.jpg]

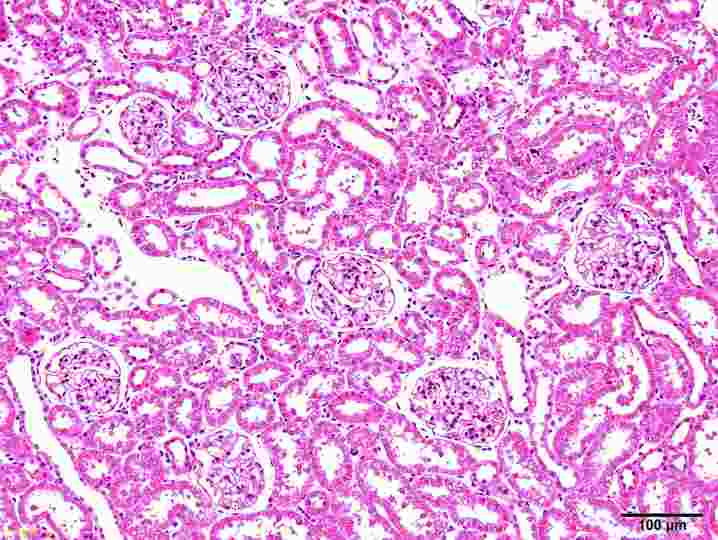

Supplement: Supplementary file 1 [file DataSheet1.zip › Original images and results for Figure 2/Fig. 2H/Fig. 2H-HE/HE-PHN/1-4.jpg]

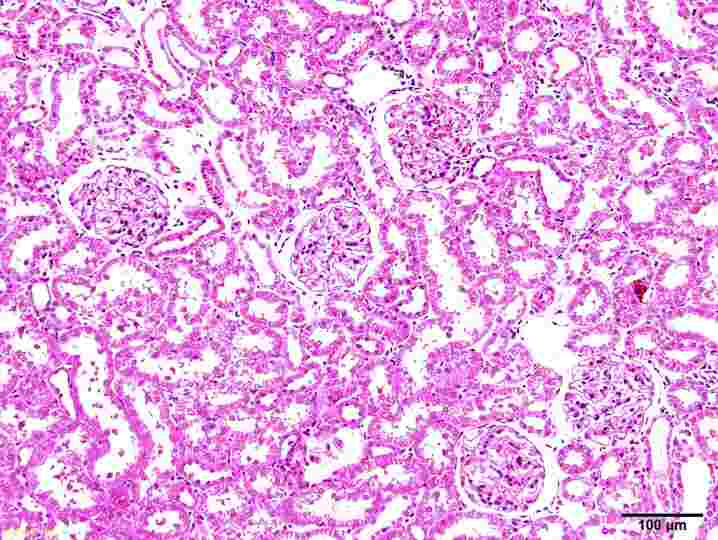

Supplement: Supplementary file 1 [file DataSheet1.zip › Original images and results for Figure 2/Fig. 2H/Fig. 2H-HE/HE-PHN/1-5.jpg]

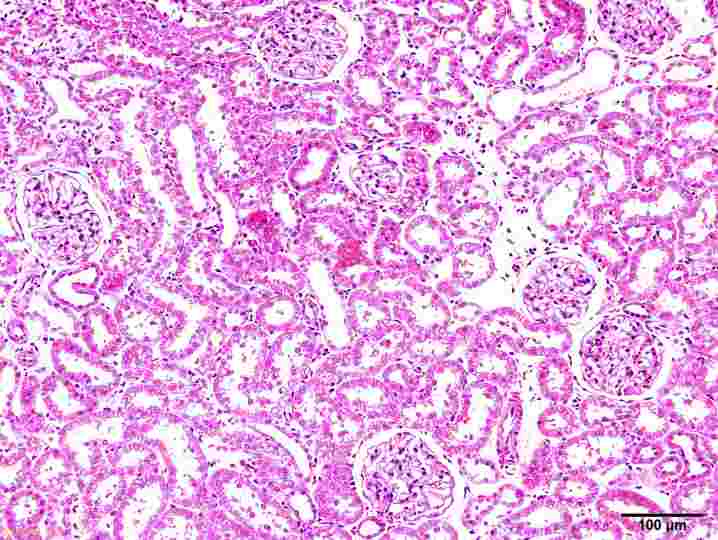

Supplement: Supplementary file 1 [file DataSheet1.zip › Original images and results for Figure 2/Fig. 2H/Fig. 2H-HE/HE-PHN/2-1.jpg]

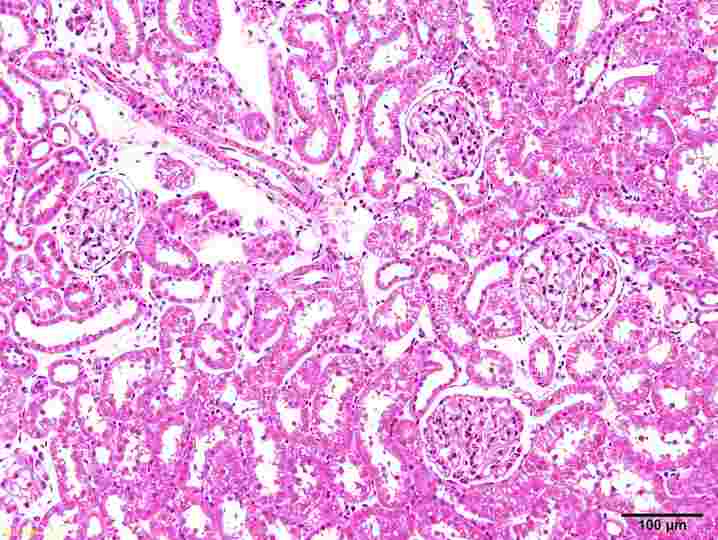

Supplement: Supplementary file 1 [file DataSheet1.zip › Original images and results for Figure 2/Fig. 2H/Fig. 2H-HE/HE-PHN/2-2.jpg]

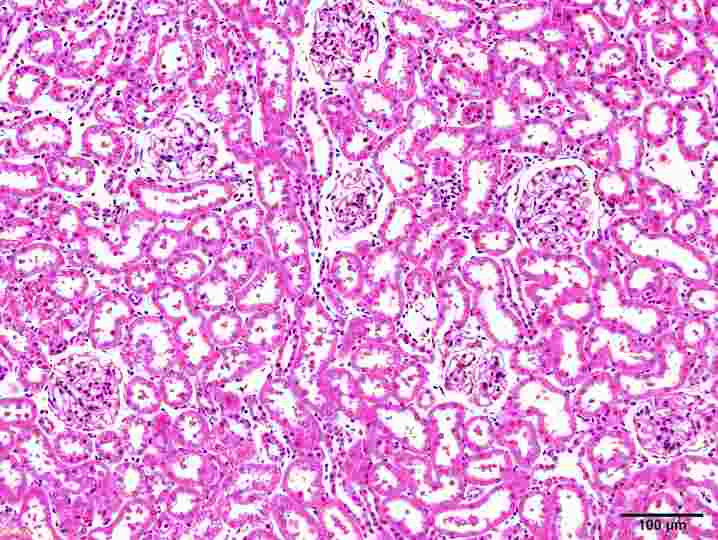

Supplement: Supplementary file 1 [file DataSheet1.zip › Original images and results for Figure 2/Fig. 2H/Fig. 2H-HE/HE-PHN/2-3.jpg]

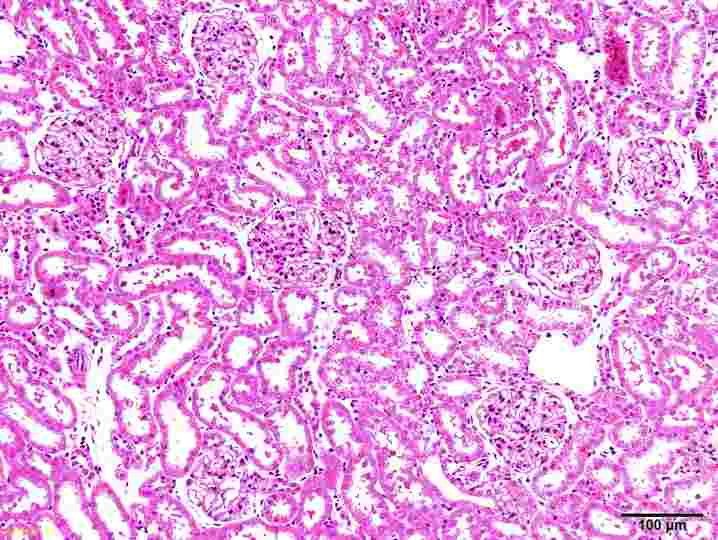

Supplement: Supplementary file 1 [file DataSheet1.zip › Original images and results for Figure 2/Fig. 2H/Fig. 2H-HE/HE-PHN/2-4.jpg]

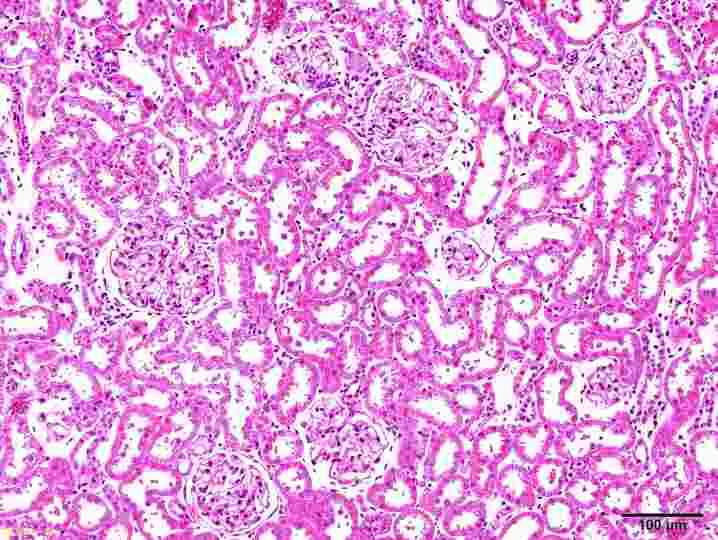

Supplement: Supplementary file 1 [file DataSheet1.zip › Original images and results for Figure 2/Fig. 2H/Fig. 2H-HE/HE-PHN/2-5.jpg]

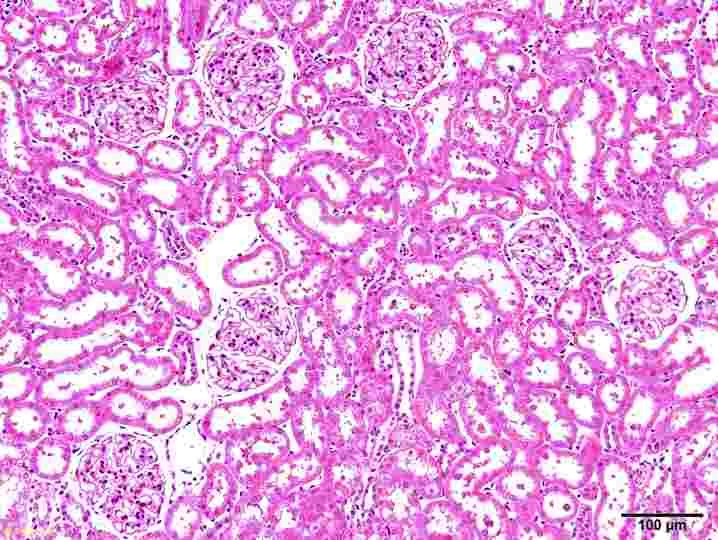

Supplement: Supplementary file 1 [file DataSheet1.zip › Original images and results for Figure 2/Fig. 2H/Fig. 2H-HE/HE-PHN/3-1.jpg]

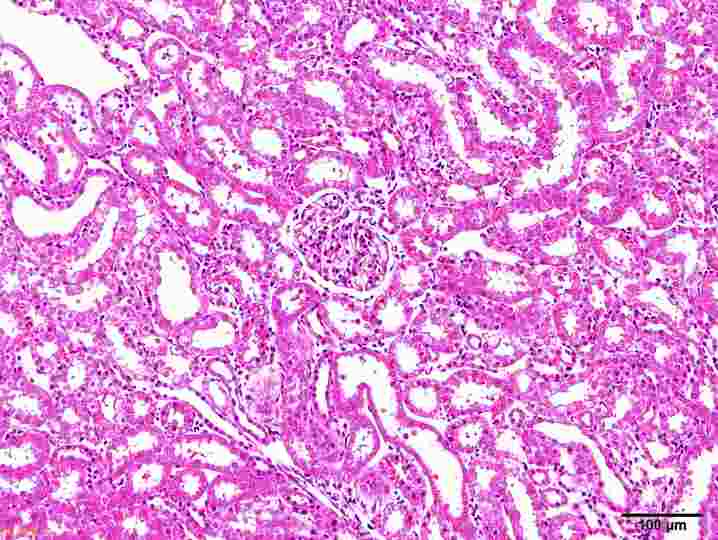

Supplement: Supplementary file 1 [file DataSheet1.zip › Original images and results for Figure 2/Fig. 2H/Fig. 2H-HE/HE-PHN/3-2.jpg]

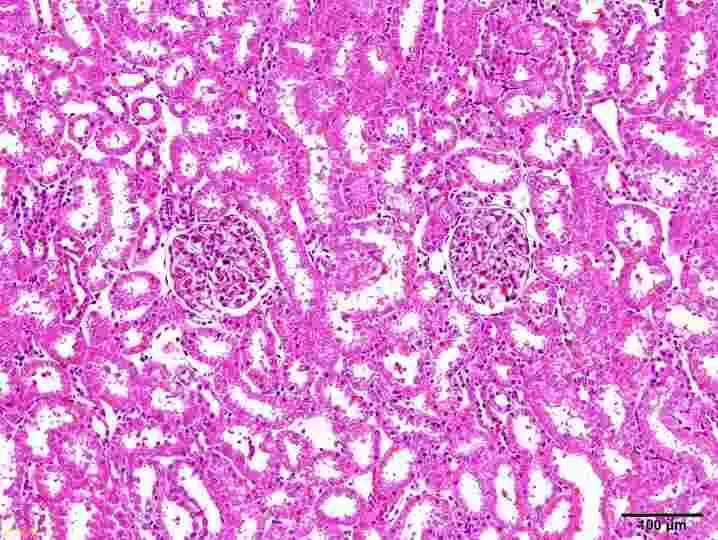

Supplement: Supplementary file 1 [file DataSheet1.zip › Original images and results for Figure 2/Fig. 2H/Fig. 2H-HE/HE-PHN/3-3.jpg]

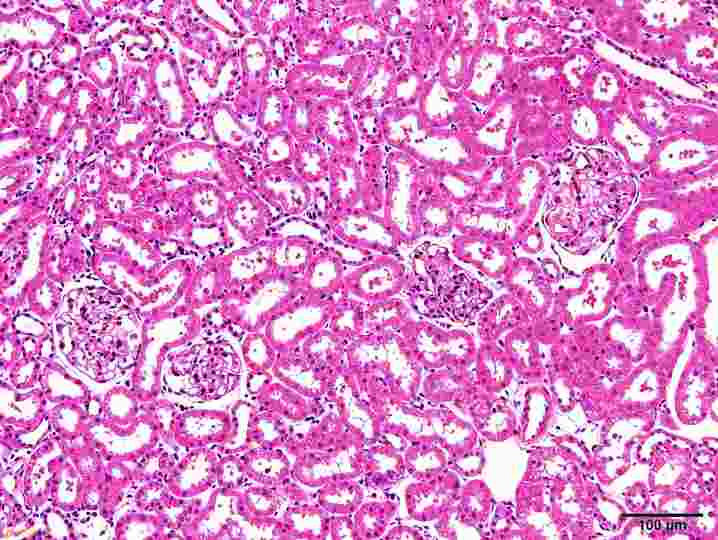

Supplement: Supplementary file 1 [file DataSheet1.zip › Original images and results for Figure 2/Fig. 2H/Fig. 2H-HE/HE-PHN/3-4.jpg]

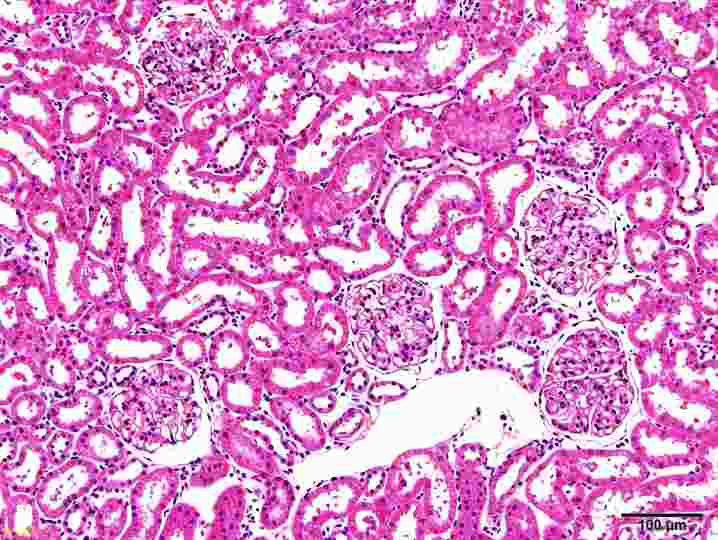

Supplement: Supplementary file 1 [file DataSheet1.zip › Original images and results for Figure 2/Fig. 2H/Fig. 2H-HE/HE-PHN/3-5.jpg]

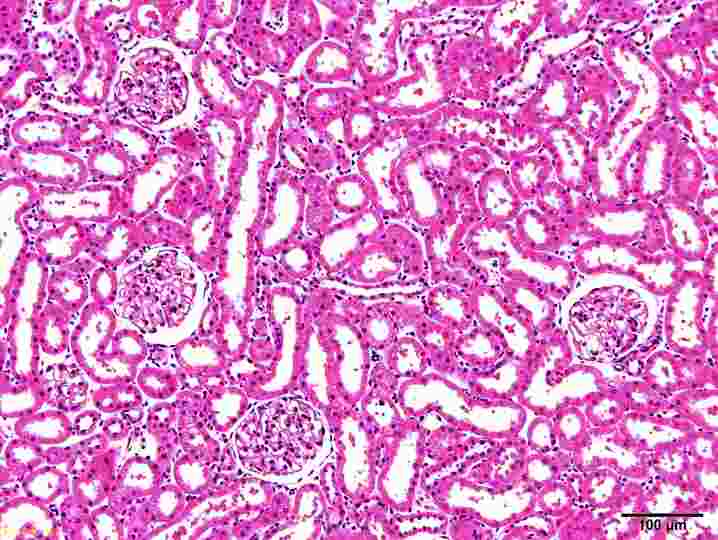

Supplement: Supplementary file 1 [file DataSheet1.zip › Original images and results for Figure 2/Fig. 2H/Fig. 2H-HE/HE-PHN/4-1.jpg]

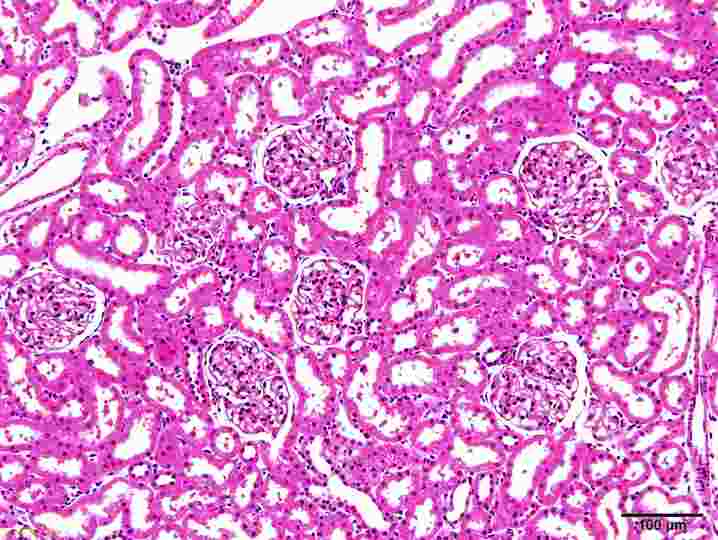

Supplement: Supplementary file 1 [file DataSheet1.zip › Original images and results for Figure 2/Fig. 2H/Fig. 2H-HE/HE-PHN/4-2.jpg]

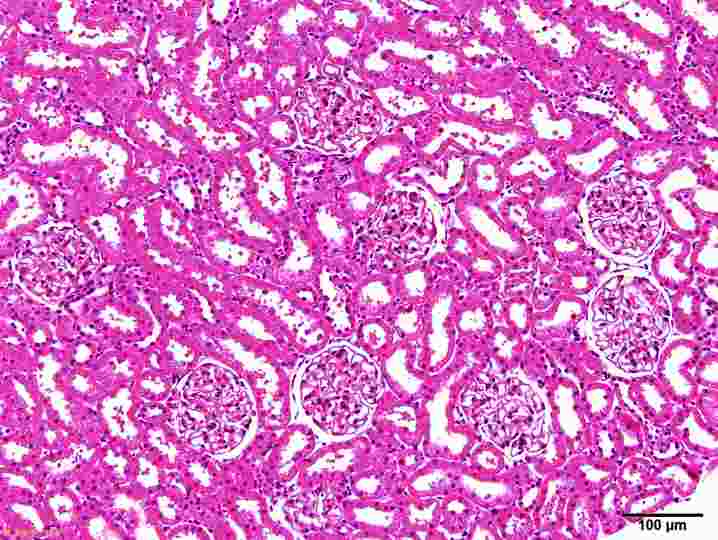

Supplement: Supplementary file 1 [file DataSheet1.zip › Original images and results for Figure 2/Fig. 2H/Fig. 2H-HE/HE-PHN/4-3.jpg]

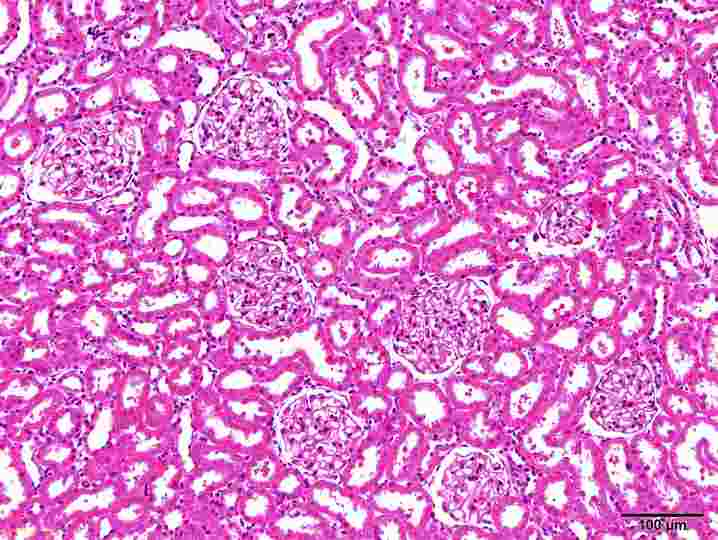

Supplement: Supplementary file 1 [file DataSheet1.zip › Original images and results for Figure 2/Fig. 2H/Fig. 2H-HE/HE-PHN/4-4.jpg]

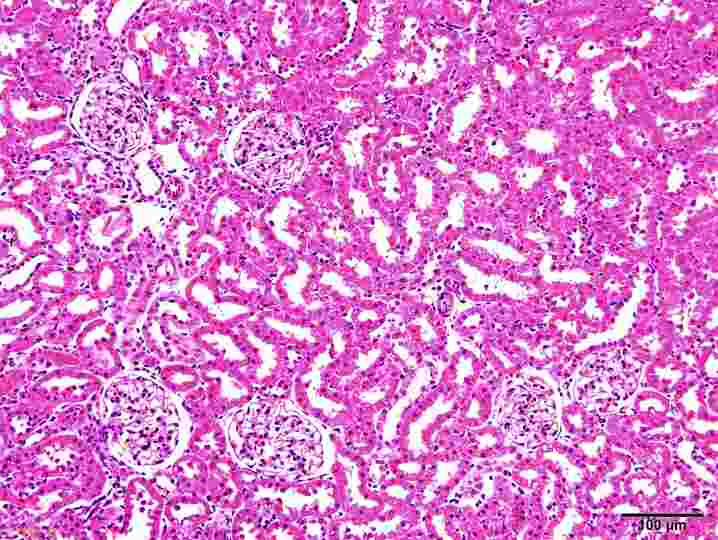

Supplement: Supplementary file 1 [file DataSheet1.zip › Original images and results for Figure 2/Fig. 2H/Fig. 2H-HE/HE-PHN/4-5.jpg]

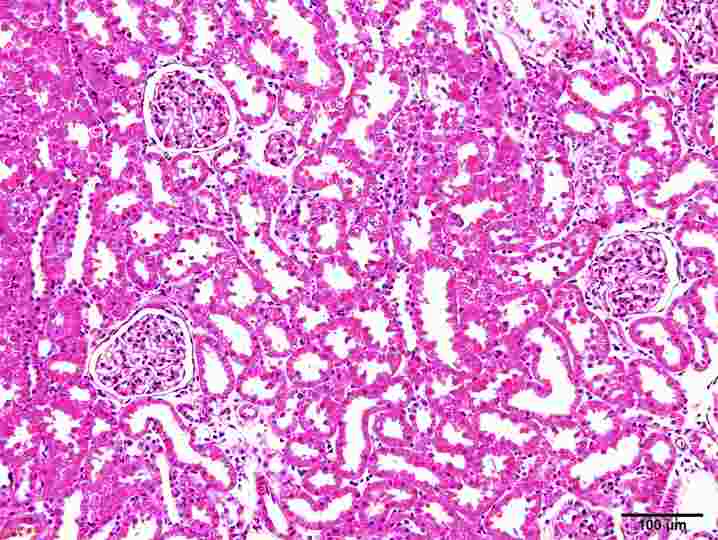

Supplement: Supplementary file 1 [file DataSheet1.zip › Original images and results for Figure 2/Fig. 2H/Fig. 2H-HE/HE-PHN/5-1.jpg]

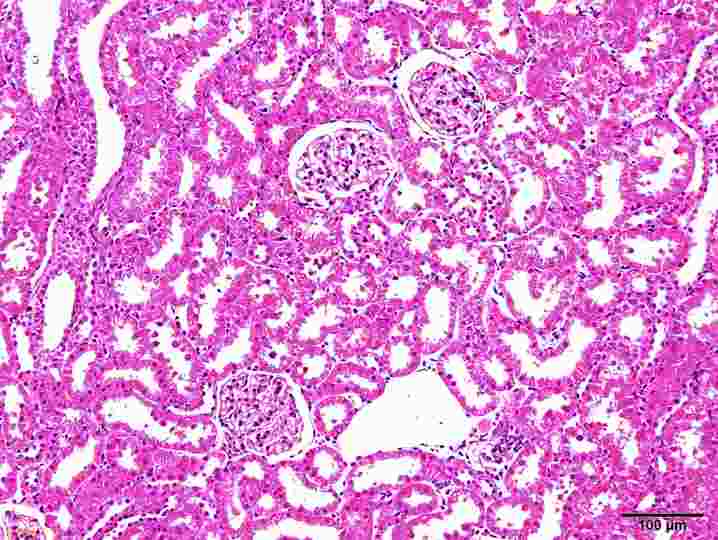

Supplement: Supplementary file 1 [file DataSheet1.zip › Original images and results for Figure 2/Fig. 2H/Fig. 2H-HE/HE-PHN/5-2.jpg]

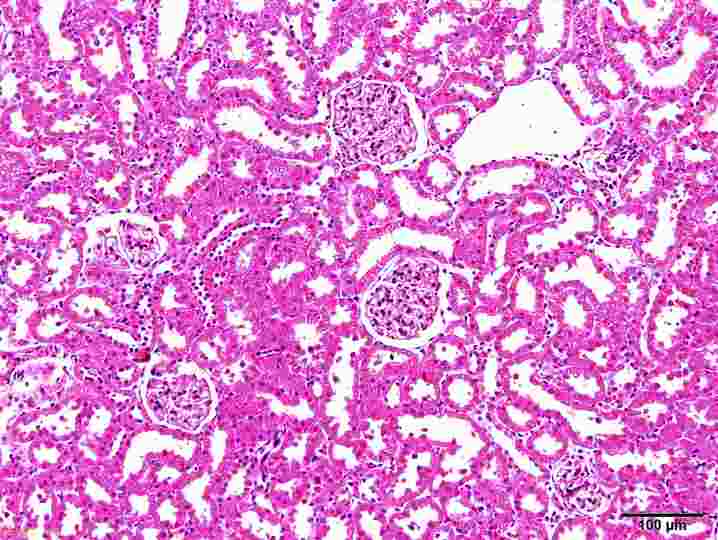

Supplement: Supplementary file 1 [file DataSheet1.zip › Original images and results for Figure 2/Fig. 2H/Fig. 2H-HE/HE-PHN/5-4.jpg]

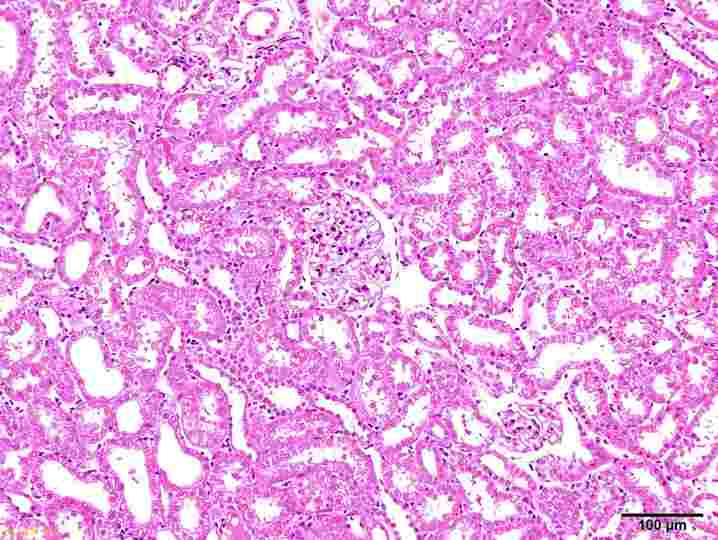

Supplement: Supplementary file 1 [file DataSheet1.zip › Original images and results for Figure 2/Fig. 2H/Fig. 2H-HE/HE-PHN/6-1.jpg]

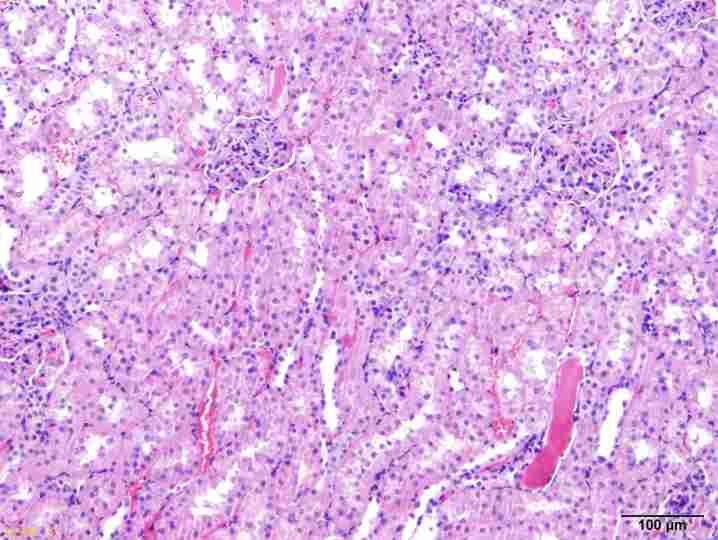

Supplement: Supplementary file 1 [file DataSheet1.zip › Original images and results for Figure 2/Fig. 2H/Fig. 2H-HE/HE-SQ-H/1-1.jpg]

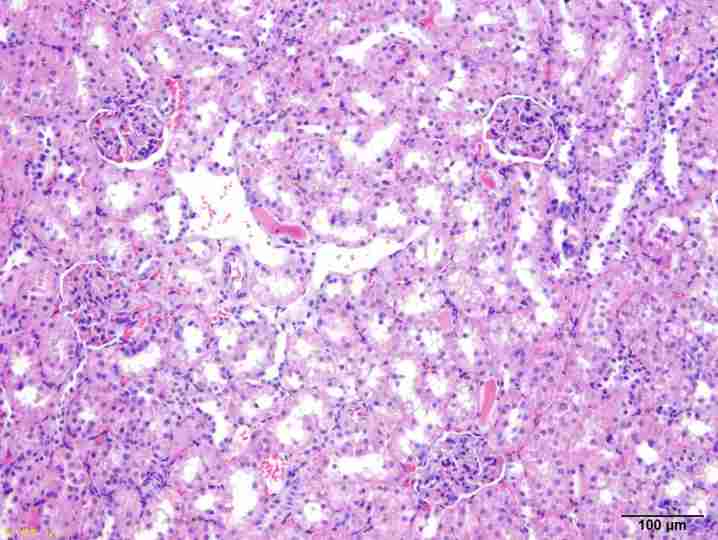

Supplement: Supplementary file 1 [file DataSheet1.zip › Original images and results for Figure 2/Fig. 2H/Fig. 2H-HE/HE-SQ-H/1-2.jpg]

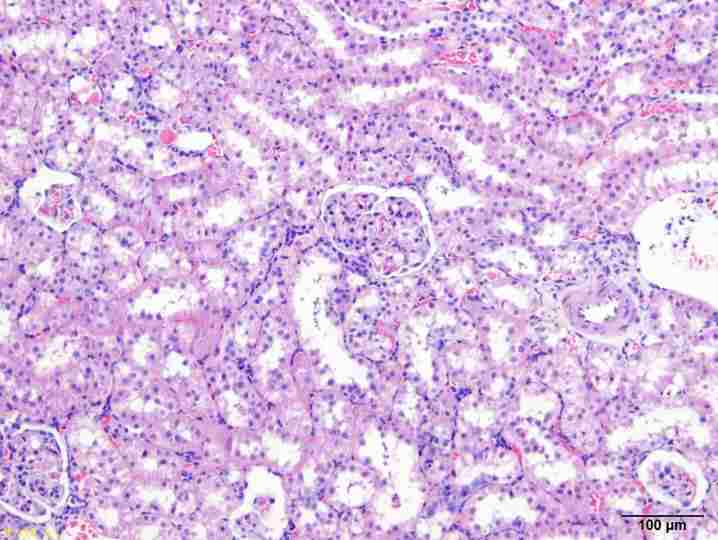

Supplement: Supplementary file 1 [file DataSheet1.zip › Original images and results for Figure 2/Fig. 2H/Fig. 2H-HE/HE-SQ-H/1-3.jpg]

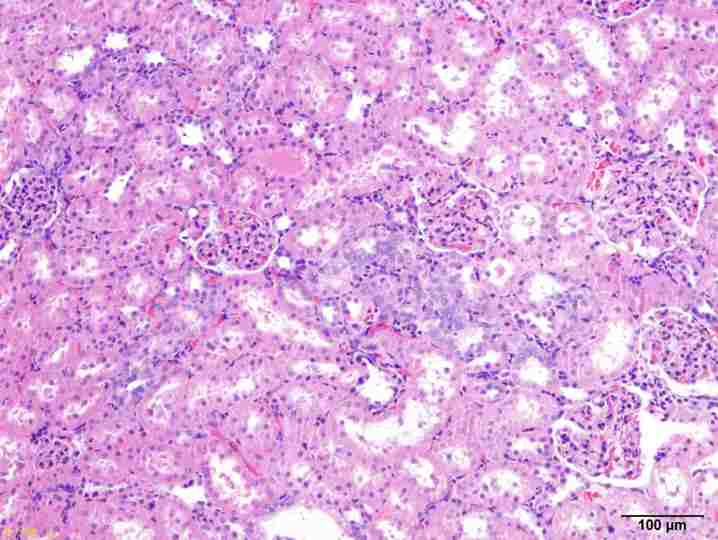

Supplement: Supplementary file 1 [file DataSheet1.zip › Original images and results for Figure 2/Fig. 2H/Fig. 2H-HE/HE-SQ-H/1-4.jpg]

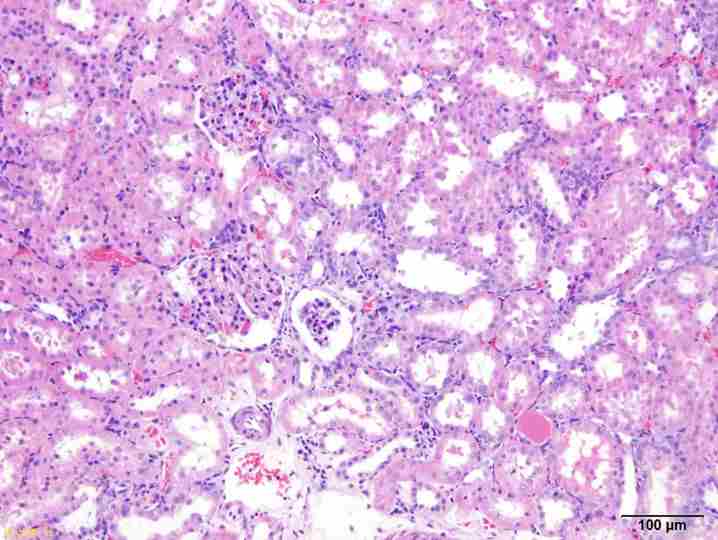

Supplement: Supplementary file 1 [file DataSheet1.zip › Original images and results for Figure 2/Fig. 2H/Fig. 2H-HE/HE-SQ-H/1-5.jpg]

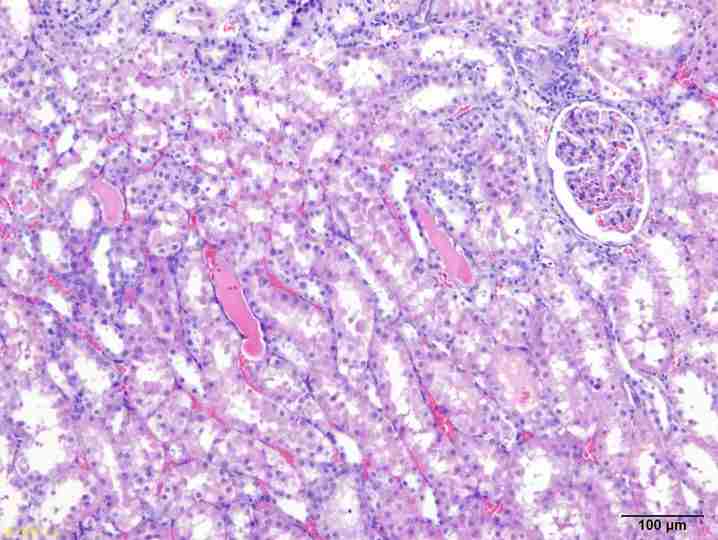

Supplement: Supplementary file 1 [file DataSheet1.zip › Original images and results for Figure 2/Fig. 2H/Fig. 2H-HE/HE-SQ-H/2-1.jpg]

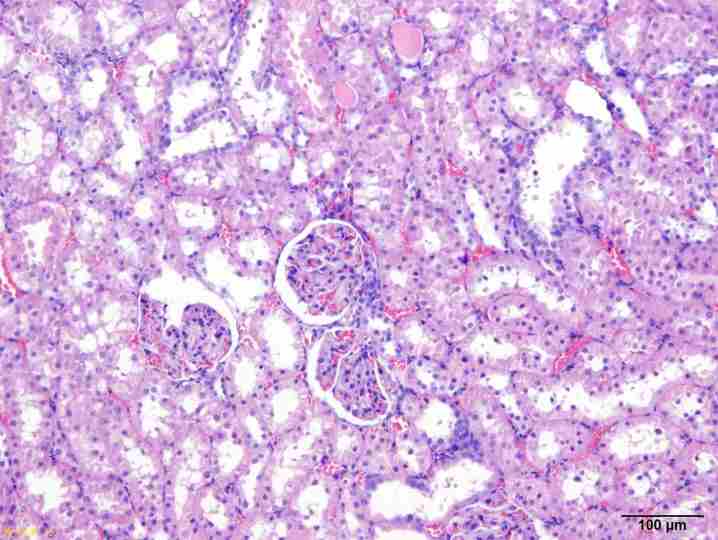

Supplement: Supplementary file 1 [file DataSheet1.zip › Original images and results for Figure 2/Fig. 2H/Fig. 2H-HE/HE-SQ-H/2-2.jpg]

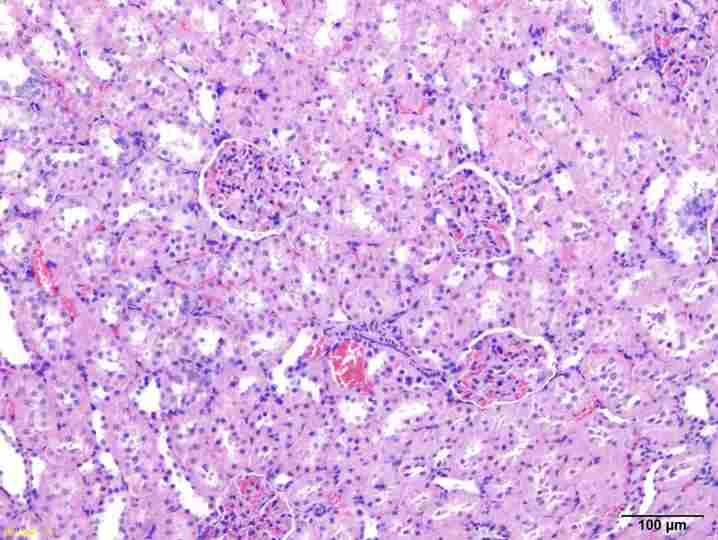

Supplement: Supplementary file 1 [file DataSheet1.zip › Original images and results for Figure 2/Fig. 2H/Fig. 2H-HE/HE-SQ-H/2-3.jpg]

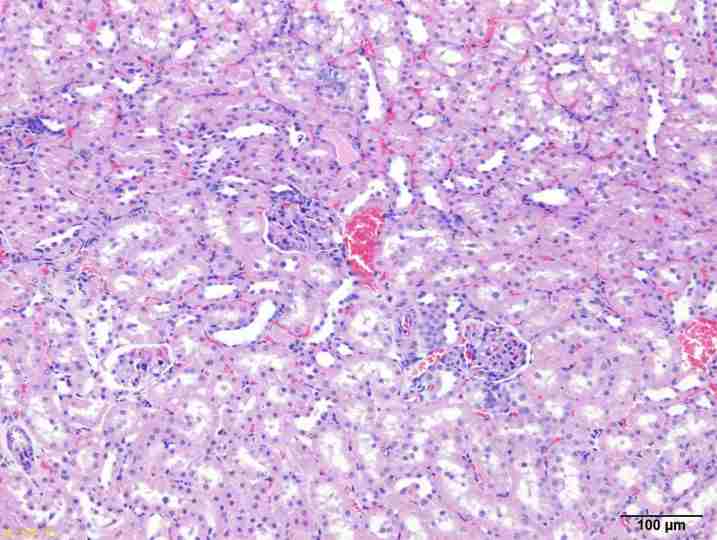

Supplement: Supplementary file 1 [file DataSheet1.zip › Original images and results for Figure 2/Fig. 2H/Fig. 2H-HE/HE-SQ-H/2-4.jpg]

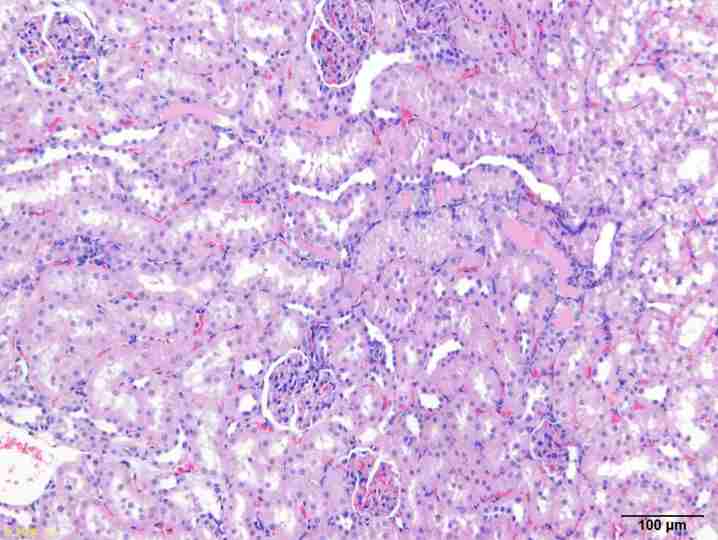

Supplement: Supplementary file 1 [file DataSheet1.zip › Original images and results for Figure 2/Fig. 2H/Fig. 2H-HE/HE-SQ-H/2-5.jpg]

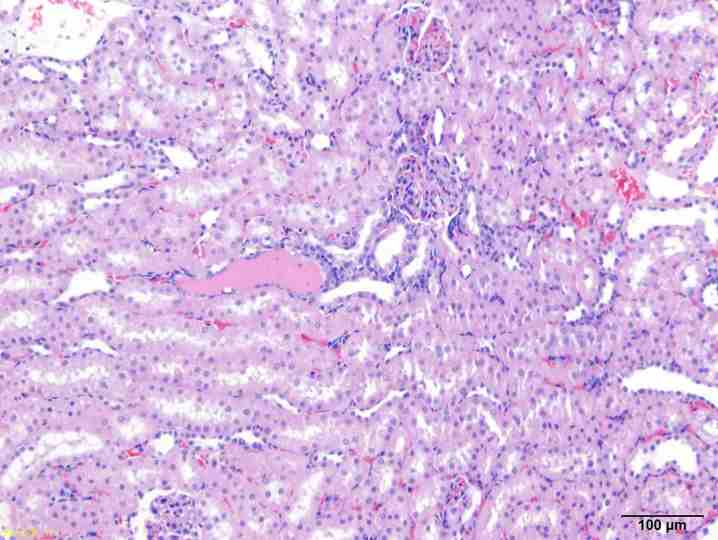

Supplement: Supplementary file 1 [file DataSheet1.zip › Original images and results for Figure 2/Fig. 2H/Fig. 2H-HE/HE-SQ-H/3-1.jpg]

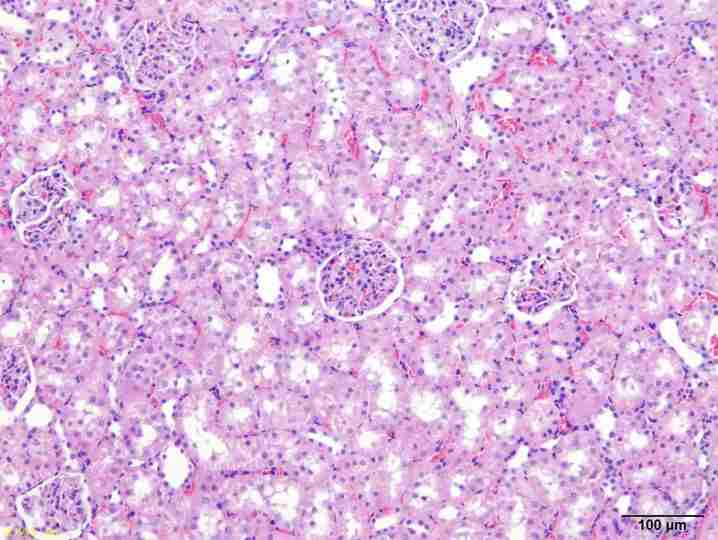

Supplement: Supplementary file 1 [file DataSheet1.zip › Original images and results for Figure 2/Fig. 2H/Fig. 2H-HE/HE-SQ-H/3-2.jpg]

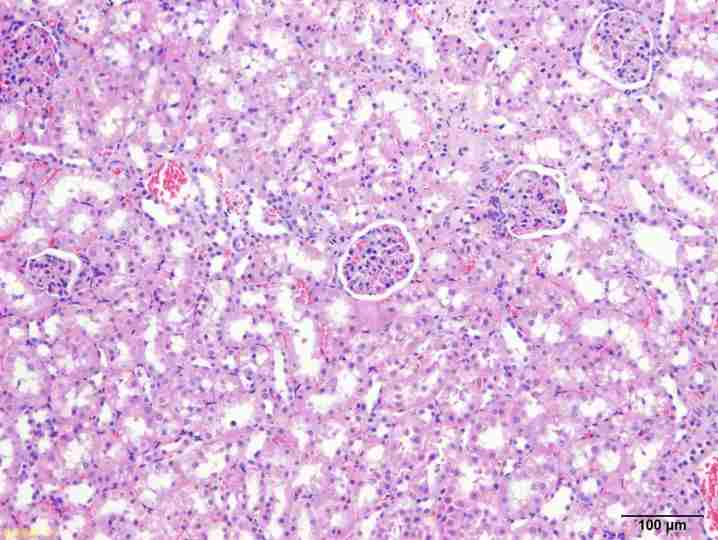

Supplement: Supplementary file 1 [file DataSheet1.zip › Original images and results for Figure 2/Fig. 2H/Fig. 2H-HE/HE-SQ-H/3-3.jpg]

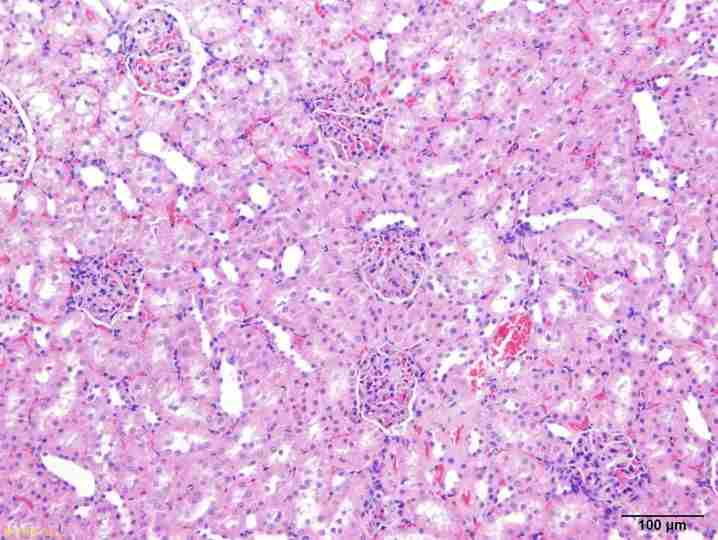

Supplement: Supplementary file 1 [file DataSheet1.zip › Original images and results for Figure 2/Fig. 2H/Fig. 2H-HE/HE-SQ-H/3-4.jpg]

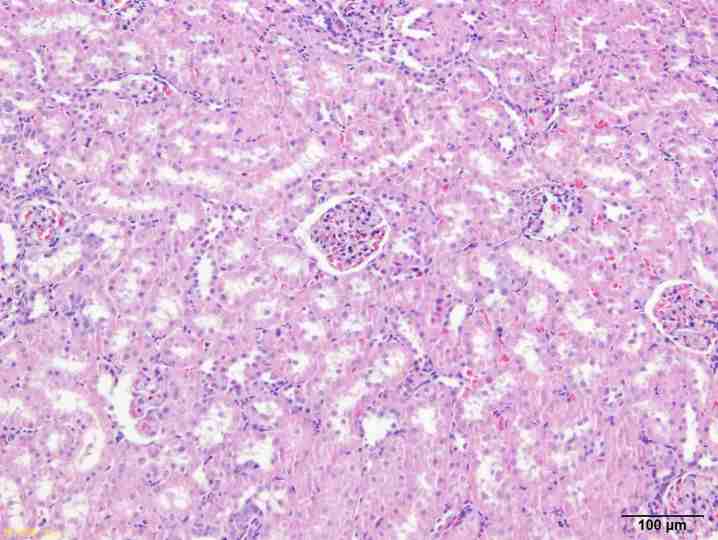

Supplement: Supplementary file 1 [file DataSheet1.zip › Original images and results for Figure 2/Fig. 2H/Fig. 2H-HE/HE-SQ-H/3-5.jpg]

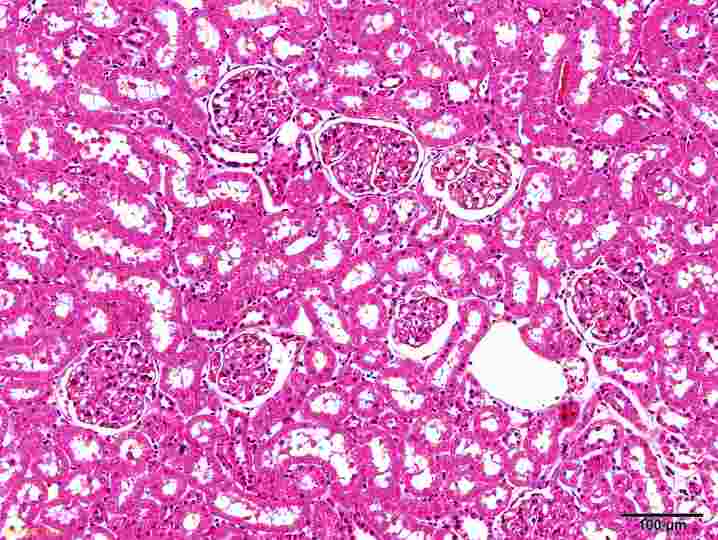

Supplement: Supplementary file 1 [file DataSheet1.zip › Original images and results for Figure 2/Fig. 2H/Fig. 2H-HE/HE-SQ-H/4-1.jpg]

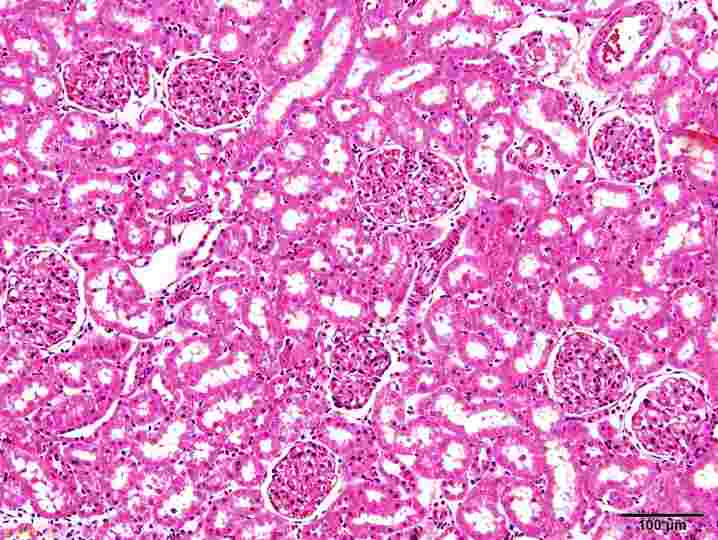

Supplement: Supplementary file 1 [file DataSheet1.zip › Original images and results for Figure 2/Fig. 2H/Fig. 2H-HE/HE-SQ-H/4-2 image in Fig. 1H HE.jpg]

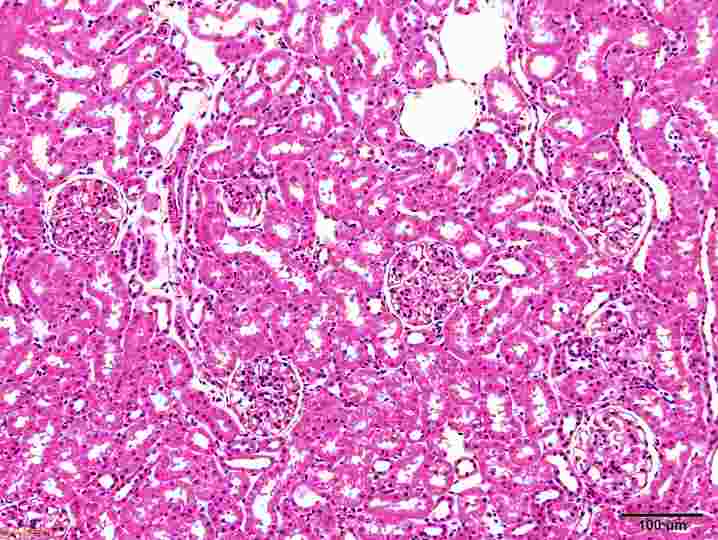

Supplement: Supplementary file 1 [file DataSheet1.zip › Original images and results for Figure 2/Fig. 2H/Fig. 2H-HE/HE-SQ-H/4-3.jpg]

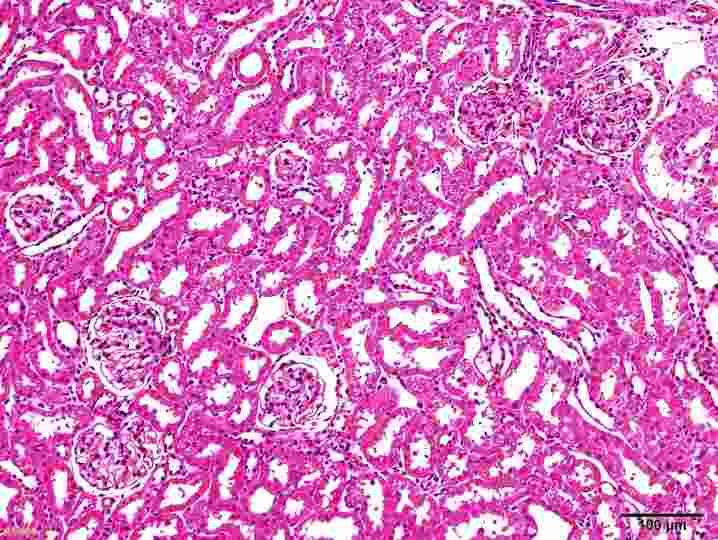

Supplement: Supplementary file 1 [file DataSheet1.zip › Original images and results for Figure 2/Fig. 2H/Fig. 2H-HE/HE-SQ-H/4-4.jpg]

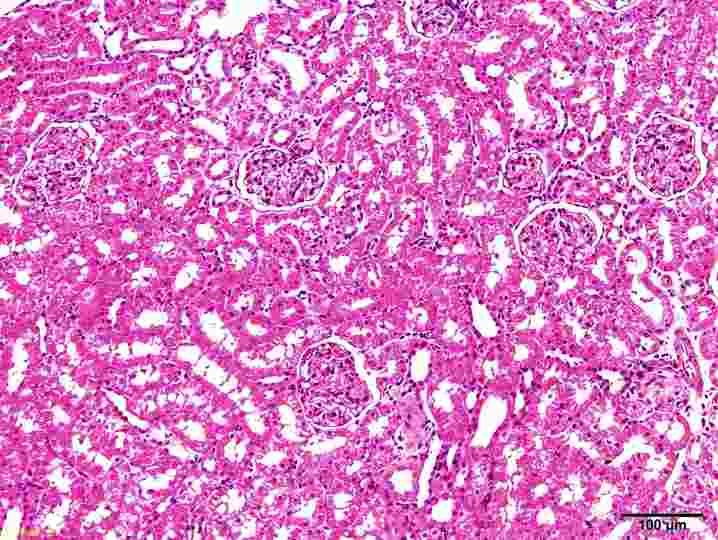

Supplement: Supplementary file 1 [file DataSheet1.zip › Original images and results for Figure 2/Fig. 2H/Fig. 2H-HE/HE-SQ-H/4-5.jpg]

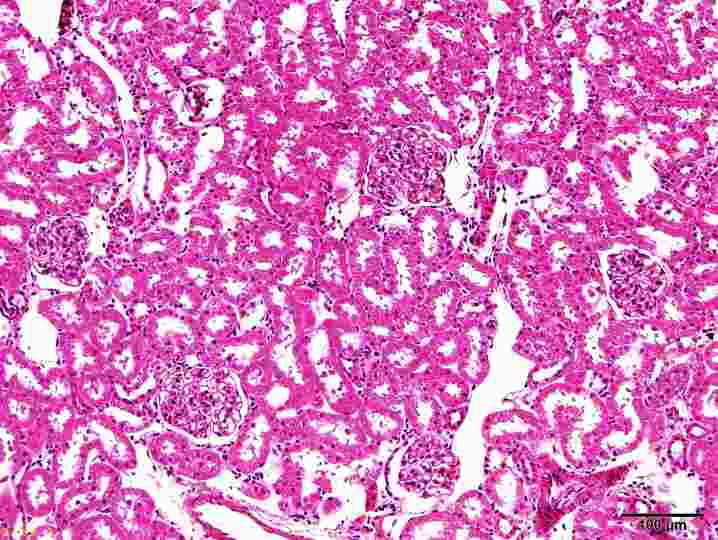

Supplement: Supplementary file 1 [file DataSheet1.zip › Original images and results for Figure 2/Fig. 2H/Fig. 2H-HE/HE-SQ-H/5-1.jpg]

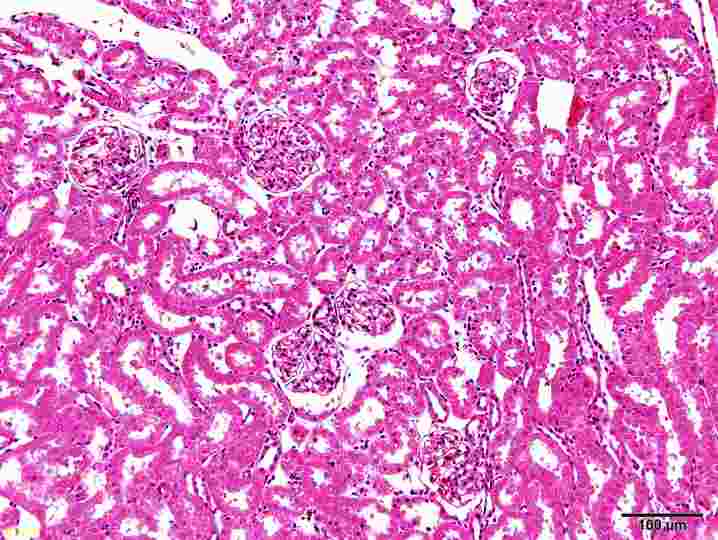

Supplement: Supplementary file 1 [file DataSheet1.zip › Original images and results for Figure 2/Fig. 2H/Fig. 2H-HE/HE-SQ-H/5-2.jpg]

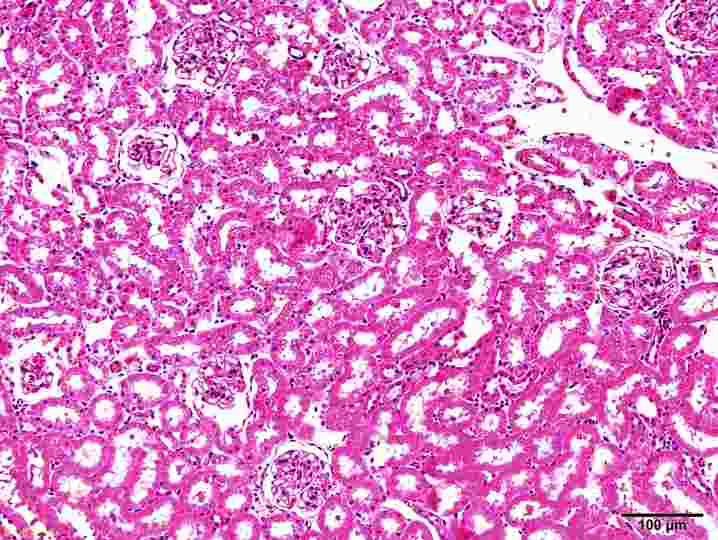

Supplement: Supplementary file 1 [file DataSheet1.zip › Original images and results for Figure 2/Fig. 2H/Fig. 2H-HE/HE-SQ-H/5-3.jpg]

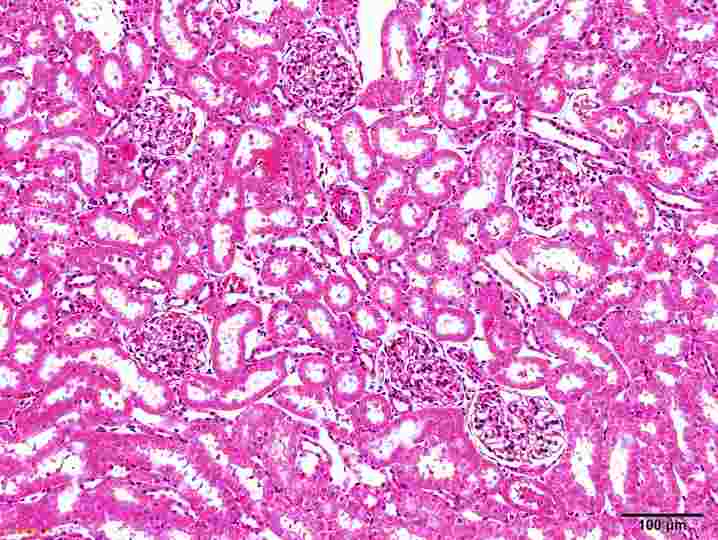

Supplement: Supplementary file 1 [file DataSheet1.zip › Original images and results for Figure 2/Fig. 2H/Fig. 2H-HE/HE-SQ-H/5-4.jpg]

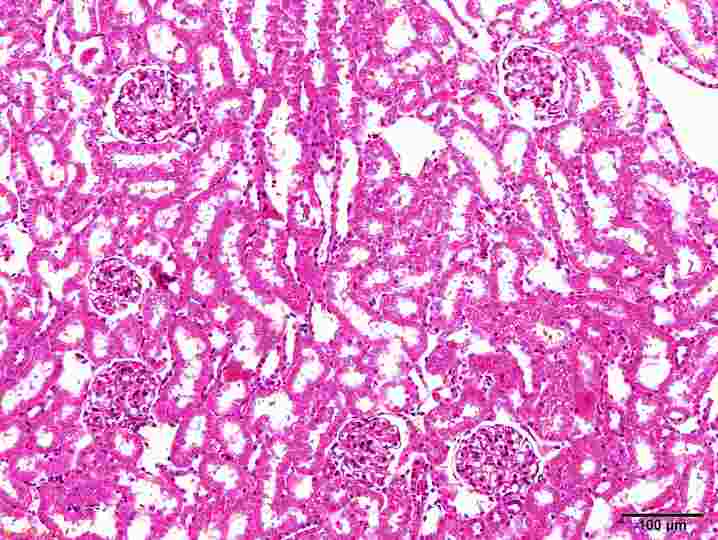

Supplement: Supplementary file 1 [file DataSheet1.zip › Original images and results for Figure 2/Fig. 2H/Fig. 2H-HE/HE-SQ-H/5-5.jpg]
