# Supplementary material for: Sanqi oral solution alleviates podocyte apoptosis in experimental membranous nephropathy by mediating EMT through the ERK/CK2-α/β-catenin pathway
Source: Front Pharmacol. 2025 May 9;16:1503961. doi: 10.3389/fphar.2025.1503961 (PMC12098599; doi:10.3389/fphar.2025.1503961)
Supplement: Supplementary file 2 [file DataSheet3.pdf]

Original data for Fig. 2B-G

Fig. 2B-Urinary protenin/ creatinine ratio(g/g)				
CON	PHN	SQ-L	SQ-H	TAC
0.375	17.652	13.518	13.754	3.397
0.336	13.467	10.726	9.206	16.101
0.341	30.342	9.128	2.583	16.573
0.406	19.399	6.058	8.002	5.506
0.384	26.658	8.153	7.6	13.261
0.372	16.698	10.584	4.697	15.797

Fig. 2C-24 h proteinuria (mg)				
CON	PHN	SQ-L	SQ-H	TAC
2.8	121.92	85.256	100.02	32.922
2.212	164.962	91.64	90.104	116.636
3.46	174.32	104.692	25.058	80.346
1.708	170.434	72.512	13.15	53.98
3.114	101.556	93.868	57.358	108.492
1.344	112.88	29.04	47.216	116.262

Fig. 2D-Serum albumin (g/L)				
CON	PHN	SQ-L	SQ-H	TAC
40.1	37.2	34.9	35.5	34
39.4	33.5	33.2	35.9	34
40.7	33.1	34.5	36.4	33.8
39.2	32.3	35.9	35.8	36.6
40.5	32.5	35.8	36.6	33.2
39.9	30.2	36.8	36.6	34.4

Fig. 2E-Serum TG (mmol/L)				
CON	PHN	SQ-L	SQ-H	TAC
0.29	0.59	0.57	0.52	0.65
0.34	0.86	0.62	0.44	0.59
0.3	0.53	0.56	0.39	0.47
0.3	0.65	0.38	0.39	0.53
0.27	0.48	0.45	0.44	0.65
0.25	0.56	0.42	0.53	0.42

Fig. 2F-Serum TC (mmol/L)				
CON	PHN	SQ-L	SQ-H	TAC
1.36	3.02	1.93	1.61	2.26
1.41	2.77	2.4	2.63	3.2
1.56	3.3	2.86	1.76	2.54
1.48	2.59	2.36	1.86	2.25
1.57	2.88	2.6	2.03	2.14
1.32	3.28	1.96	1.68	3.08

Fig. 2G-Serum LDL-c (mmol/L)				
CON	PHN	SQ-L	SQ-H	TAC
0.27	0.31	0.25	0.22	0.31
0.25	0.32	0.22	0.22	0.32
0.25	0.33	0.3	0.22	0.32
0.24	0.36	0.24	0.23	0.32
0.23	0.5	0.24	0.28	0.36
0.22	0.55	0.27	0.29	0.41

Original Image for Fig. 2H-Electron dense deposit

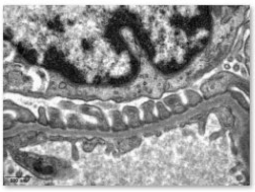
CON-1.jpg

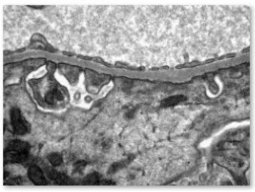
CON-2.jpg

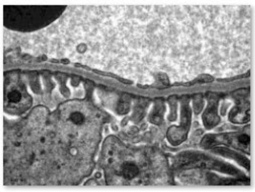
CON-3 image in Fig. 2H .jpg

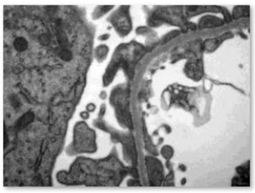
CON-4.jpg

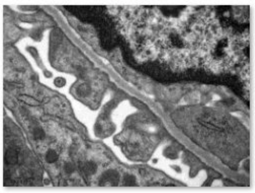
CON-5.jpg

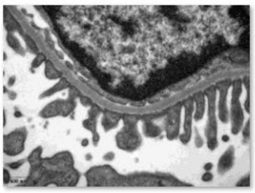
CON-6.jpg

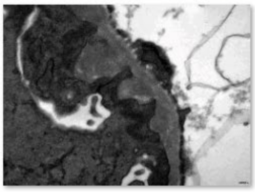
PHN-1.jpg

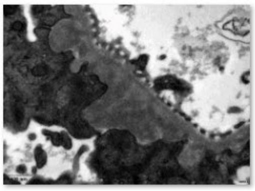
PHN-2.jpg

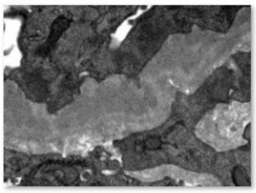
PHN-3.jpg

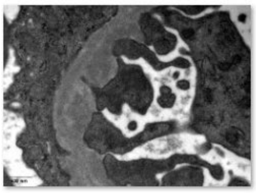
PHN-4.jpg

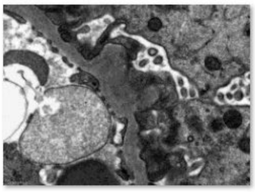
PHN-5.jpg

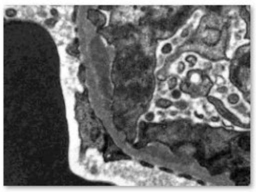
PHN-6 image in Fig. 2H.jpg

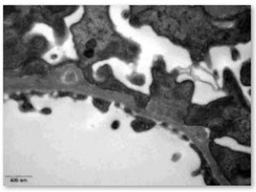
SQ-H-1.jpg
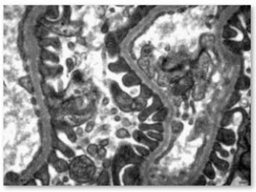
SQ-H-2.jpg
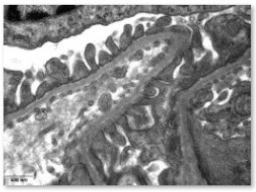
SQ-H-3.jpg
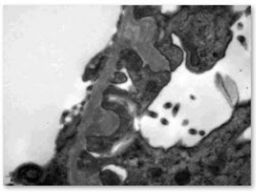
SQ-H-4.jpg
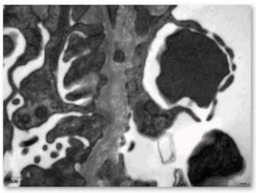
SQ-H-5.jpg
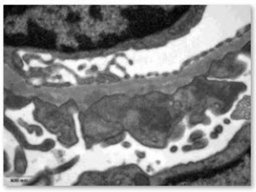
SQ-H-6 image in Fig. 2H.jpg
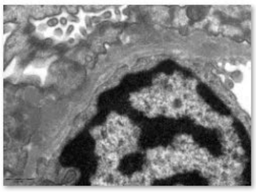
SQ-L-1.jpg
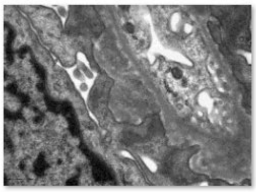
SQ-L-2.jpg
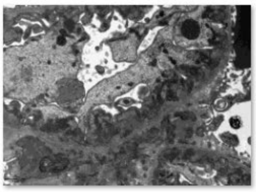
SQ-L-3.jpg
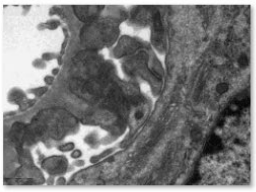
SQ-L-4.jpg
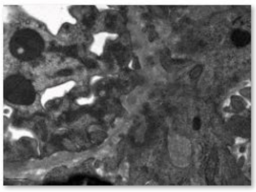
SQ-L-5.jpg
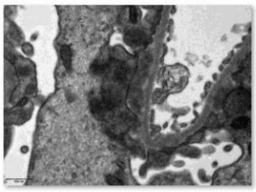
SQ-L-6 image in Fig. 2H.jpg
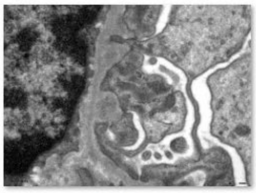
TAC-1.jpg
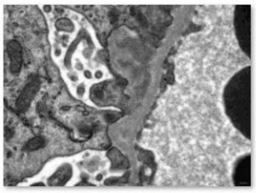
TAC-2.jpg
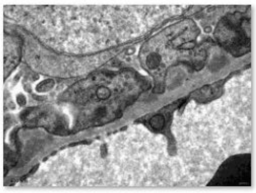
TAC-3.jpg
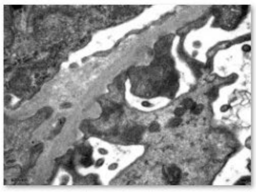
TAC-4.jpg
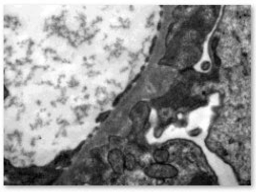
TAC-5 image in Fig. 2H.jpg
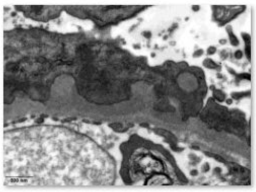
TAC-6.jpg

Original Image for Fig. 2H-HE

CON

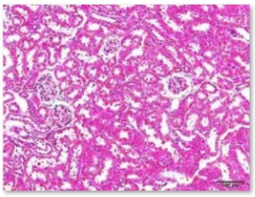
1-1.jpg
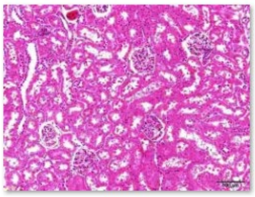
1-2.jpg
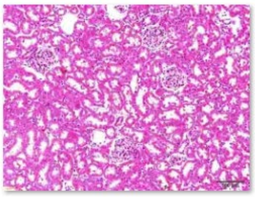
1-3.jpg
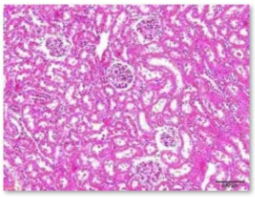
1-4.jpg
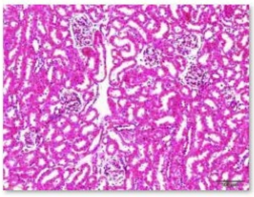
1-5.jpg
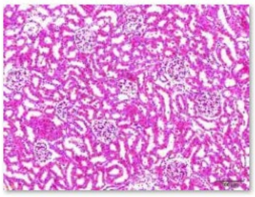
2-1.jpg
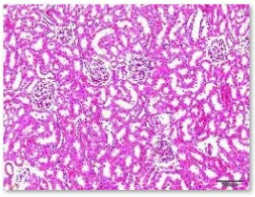
2-2.jpg
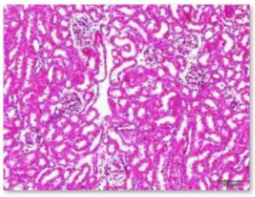
2-3.jpg

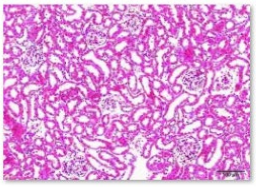
2-4.jpg
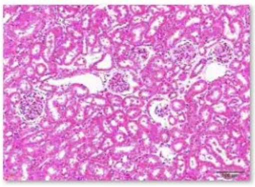
2-5.jpg
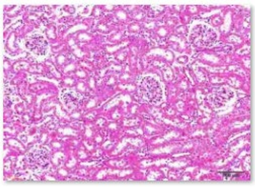
3-1.jpg
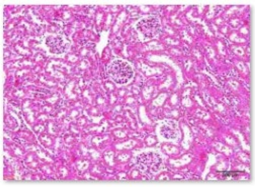
3-2.jpg
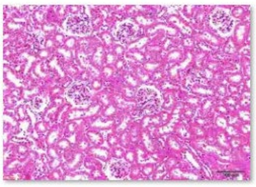
3-3 image in Fig. 1H-HE.jpg
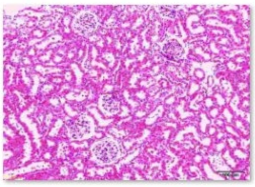
3-4.jpg
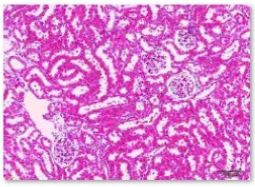
3-5.jpg
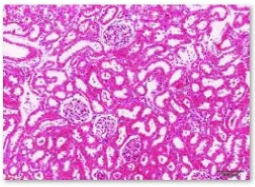
4-1.jpg
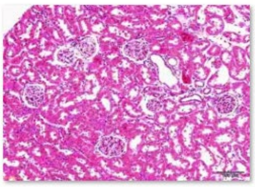
4-2.jpg
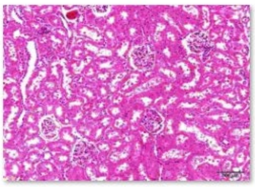
4-3.jpg
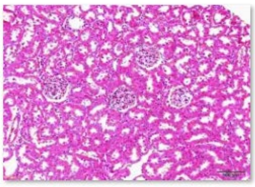
4-4.jpg
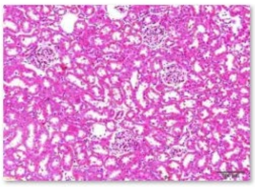
4-5.jpg
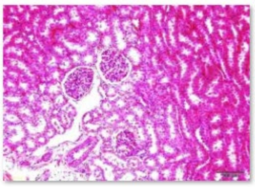
5-1.jpg
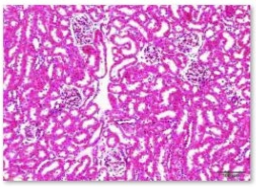
5-2.jpg
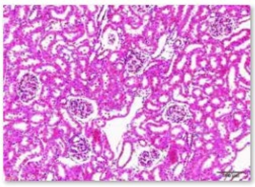
5-3.jpg
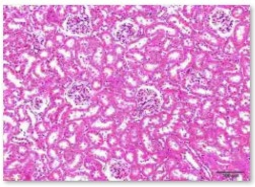
5-4.jpg
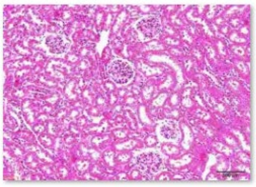
5-5.jpg
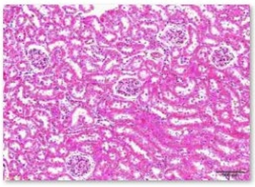
6-1.jpg
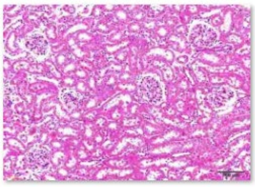
6-2.jpg
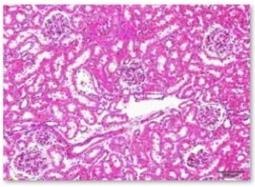
6-3.jpg
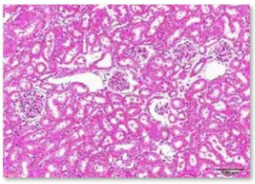
6-4.jpg
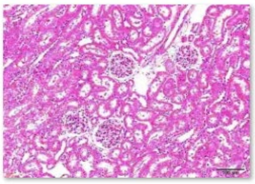
6-5.jpg

PHN

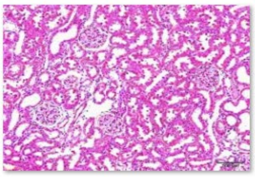
1-1.jpg
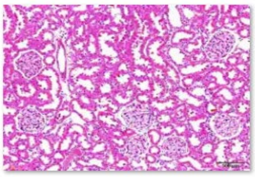
1-2 image in Fig. 1H-HE.jpg
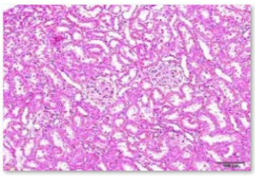
1-3.jpg
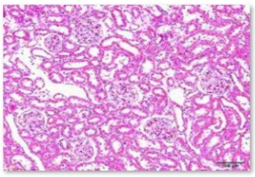
1-4.jpg
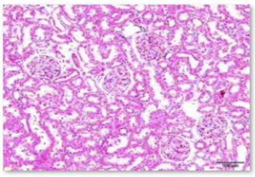
1-5.jpg
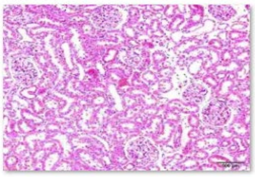
2-1.jpg
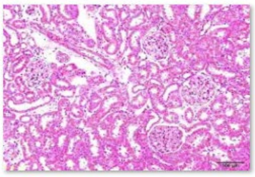
2-2.jpg
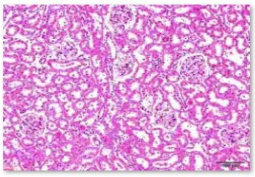
2-3.jpg

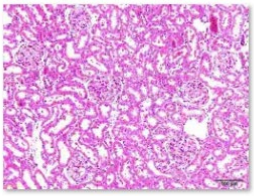
2-4.jpg

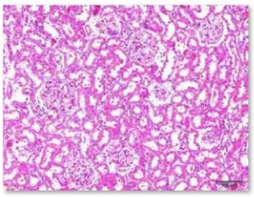
2-5.jpg

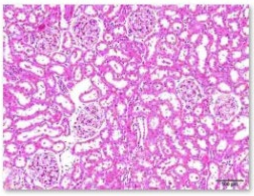
3-1.jpg

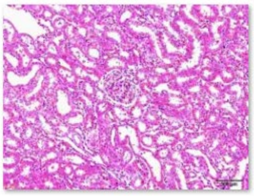
3-2.jpg

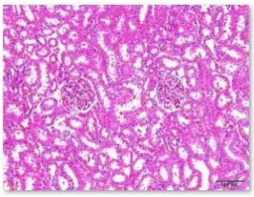
3-3.jpg

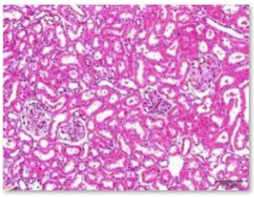
3-4.jpg

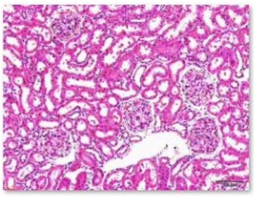
3-5.jpg

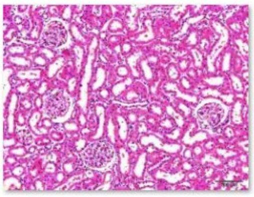
4-1.jpg

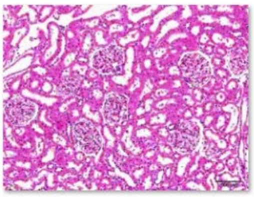
4-2.jpg

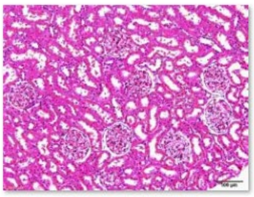
4-3.jpg

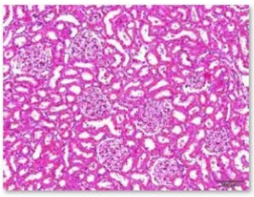
4-4.jpg

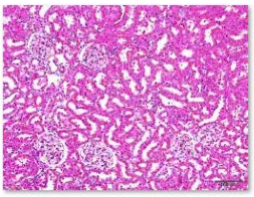
4-5.jpg

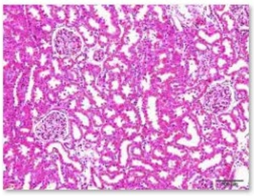
5-1.jpg

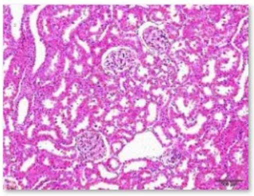
5-2.jpg

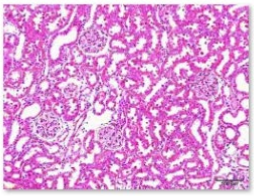
5-3.jpg

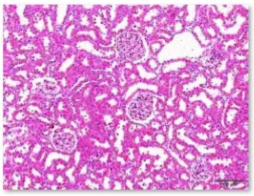
5-4.jpg

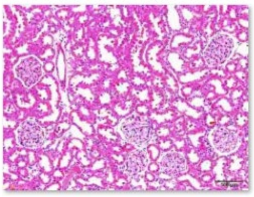
5-5.jpg

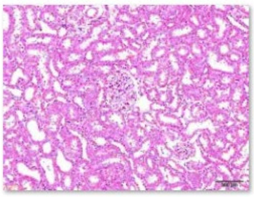
6-1.jpg

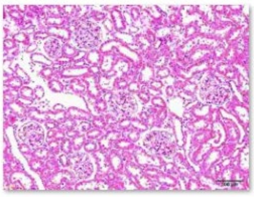
6-2.jpg

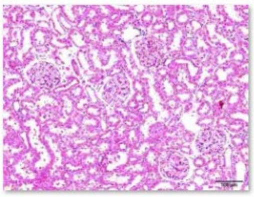
6-3.jpg

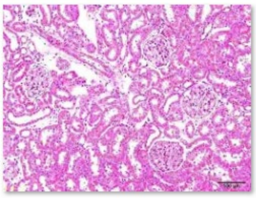
6-4.jpg

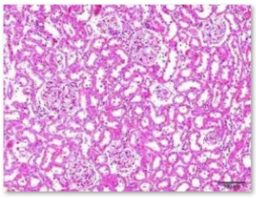
6-5.jpg

SQ-L

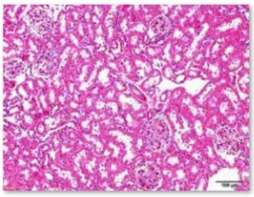
1-1.jpg

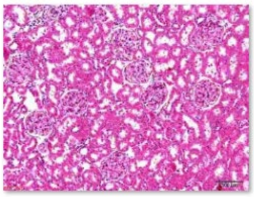
1-2.jpg

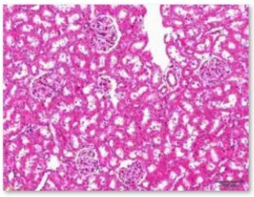
1-3.jpg

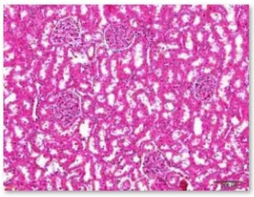
1-4.jpg

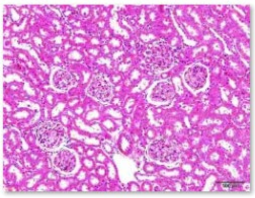
1-5.jpg

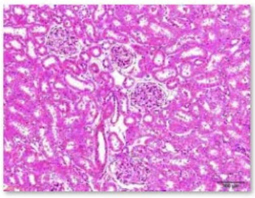
2-1.jpg

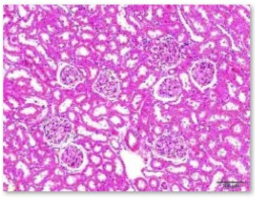
2-2 image in Fig. 1H-HE.jpg

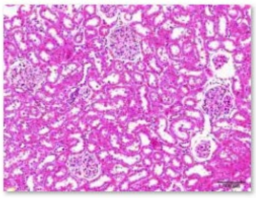
2-3.jpg

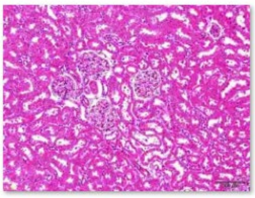
2-4.jpg

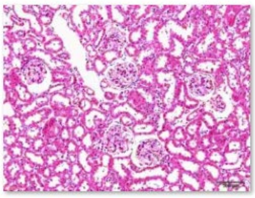
2-5.jpg

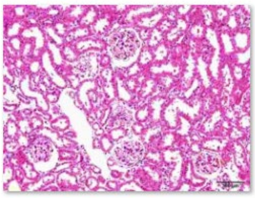
3-1.jpg

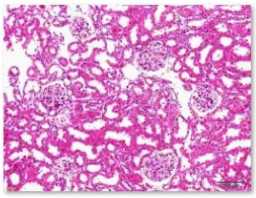
3-2.jpg

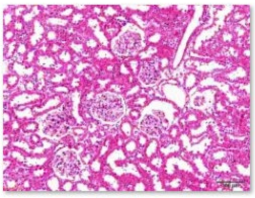
3-3.jpg

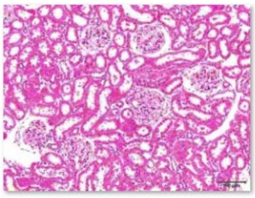
3-4.jpg

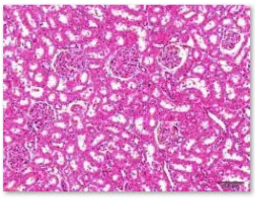
3-5.jpg

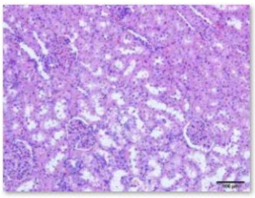
4-1.jpg

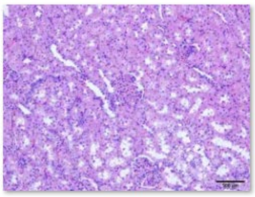
4-2.jpg

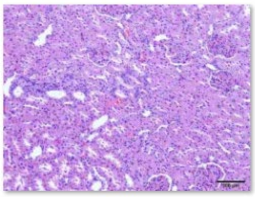
4-3.jpg

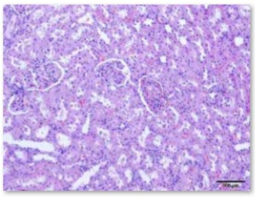
4-4.jpg

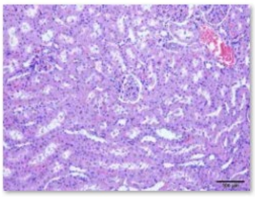
4-5.jpg

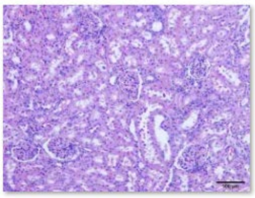
5-1.jpg

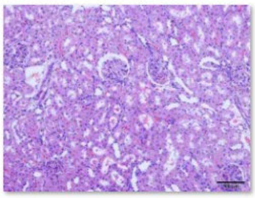
5-2.jpg

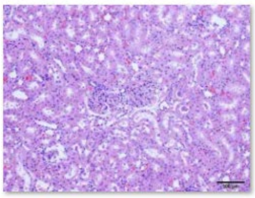
5-3.jpg

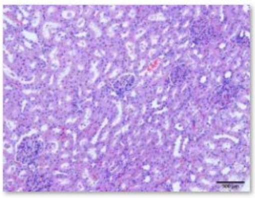
5-4.jpg

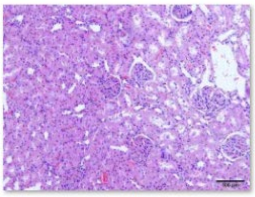
5-5.jpg

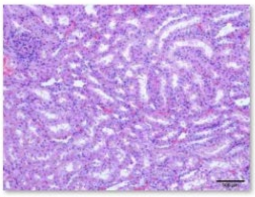
6-1.jpg

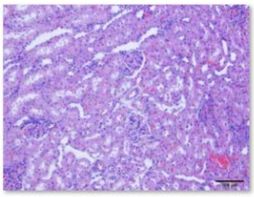
6-2.jpg

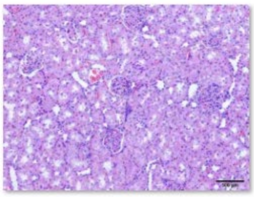
6-3.jpg

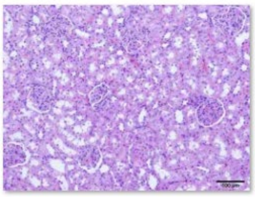
6-4.jpg

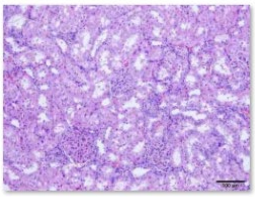
6-5.jpg

SQ-H

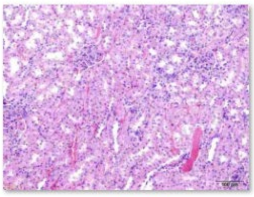
1-1.jpg

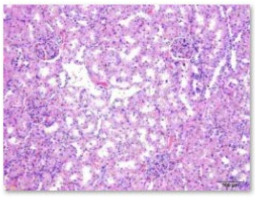
1-2.jpg

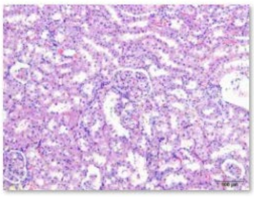
1-3.jpg

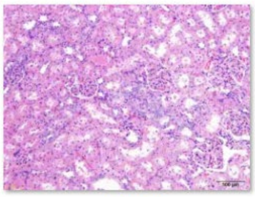
1-4.jpg

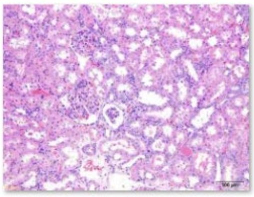
1-5.jpg

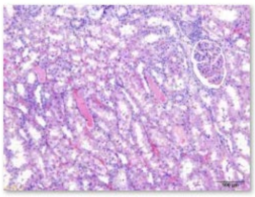
2-1.jpg

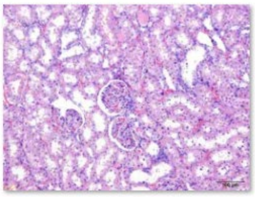
2-2.jpg

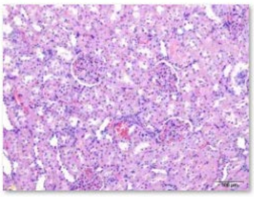
2-3.jpg

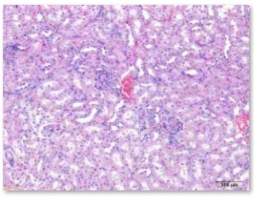
2-4.jpg

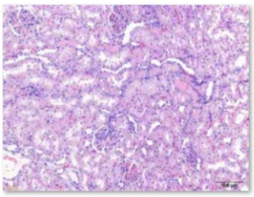
2-5.jpg

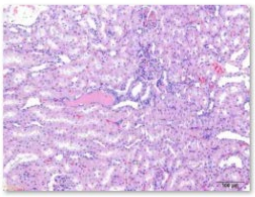
3-1.jpg

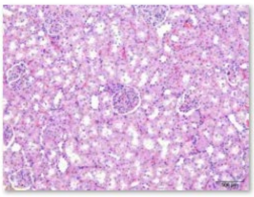
3-2.jpg

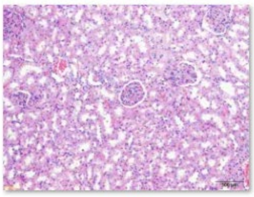
3-3.jpg

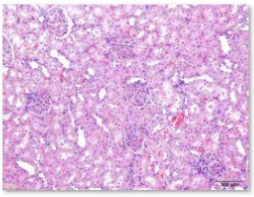
3-4.jpg

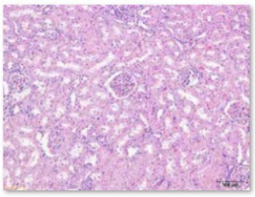
3-5.jpg

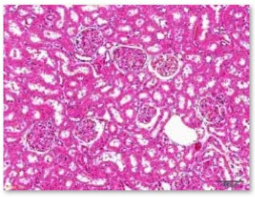
4-1.jpg

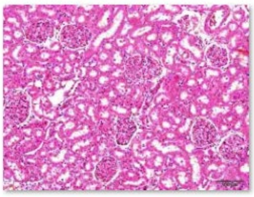
4-2 image in Fig. 1H HE.jpg

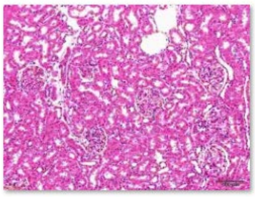
4-3.jpg

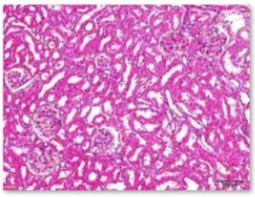
4-4.jpg

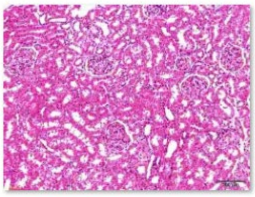
4-5.jpg

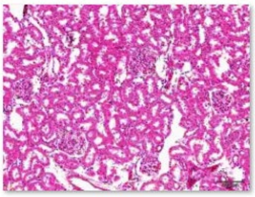
5-1.jpg

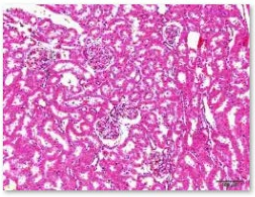
5-2.jpg

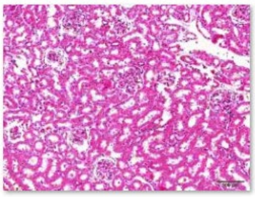
5-3.jpg

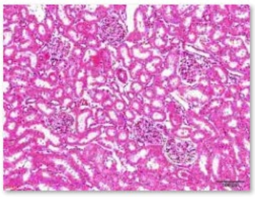
5-4.jpg

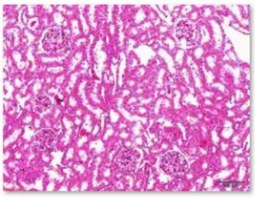
5-5.jpg

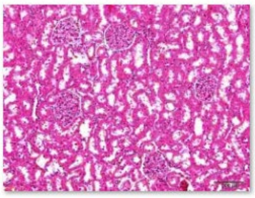
6-1.jpg

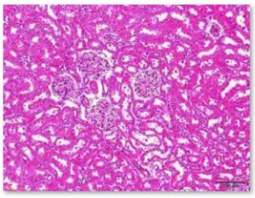
6-3.jpg

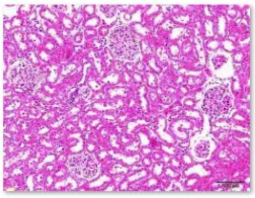
6-4.jpg

TAC

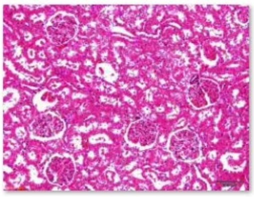
1-1.jpg

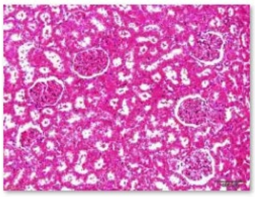
1-2.jpg

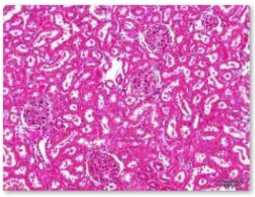
1-3.jpg

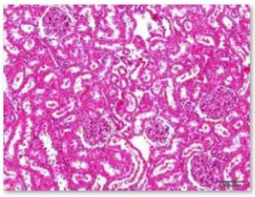
1-4.jpg

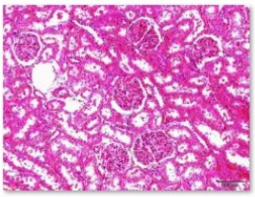
1-5.jpg

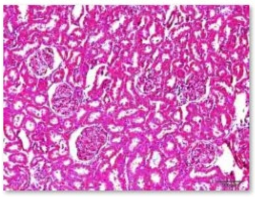
2-1.jpg

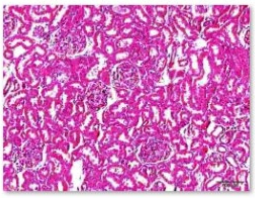
2-2.jpg

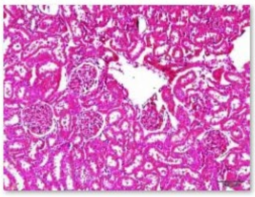
2-3.jpg

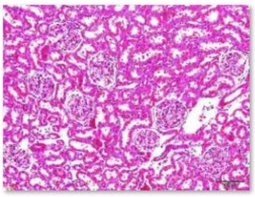
2-4.jpg

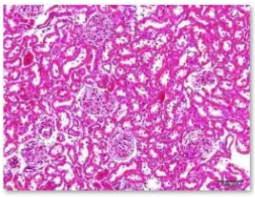
2-5.jpg

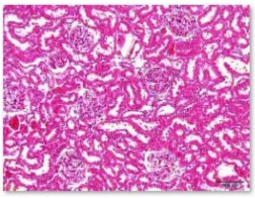
3-1.jpg

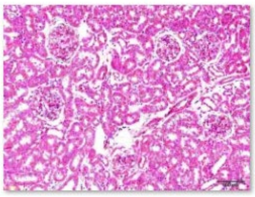
3-2.jpg

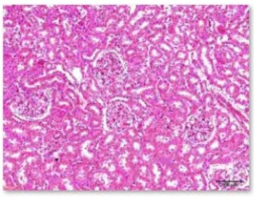
3-3 image in Fig. 1H-HE.jpg

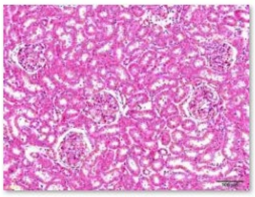
3-4.jpg

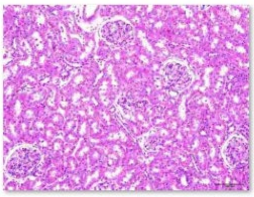
3-5.jpg

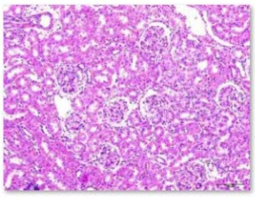
4-1.jpg

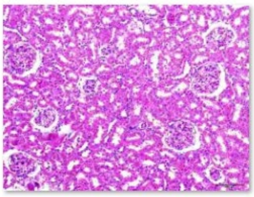
4-2.jpg

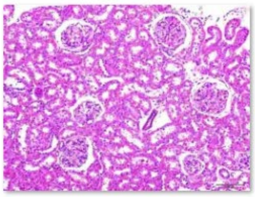
4-3.jpg

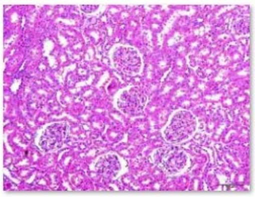
4-4.jpg

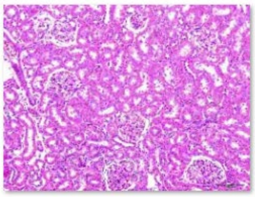
4-5.jpg

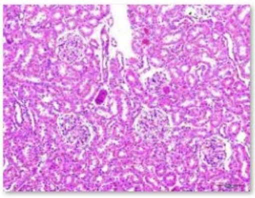
5-1.jpg
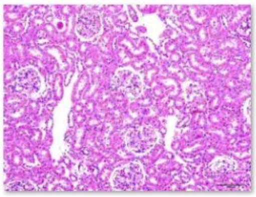
5-2.jpg
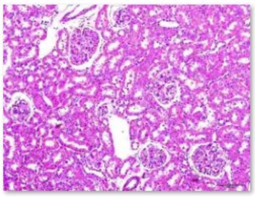
5-3.jpg
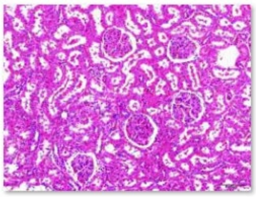
5-4.jpg
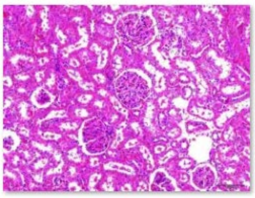
5-5.jpg
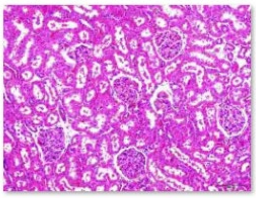
6-1.jpg
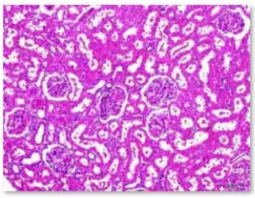
6-2.jpg
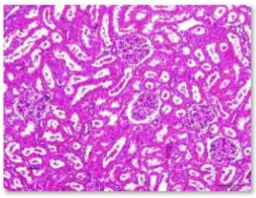
6-3.jpg
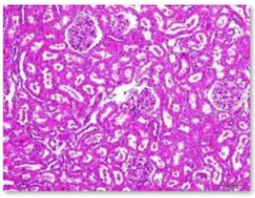
6-4.jpg
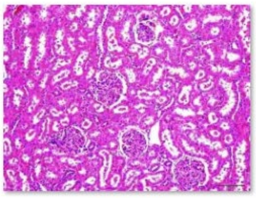
6-5.jpg

Original Image for Fig. 2H-PASM

CON

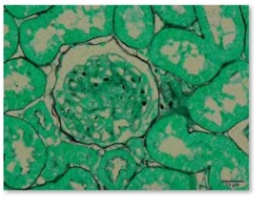
1-1.jpg
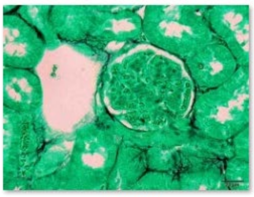
1-2.jpg
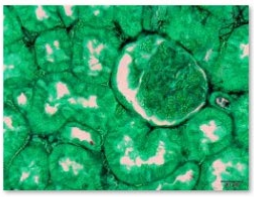
1-3.jpg
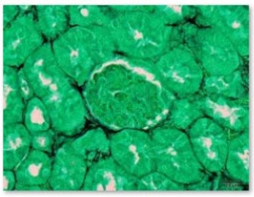
1-4.jpg
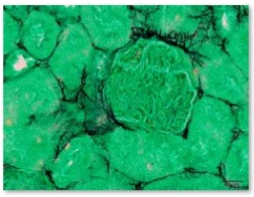
1-5.jpg
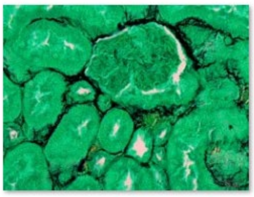
2-1.jpg
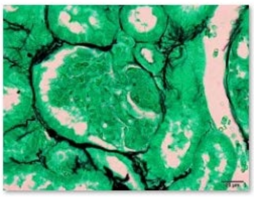
2-2.jpg
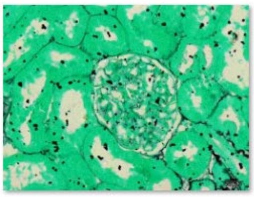
2-3.jpg
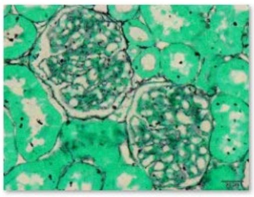
2-4.jpg
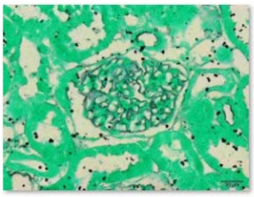
2-5.jpg
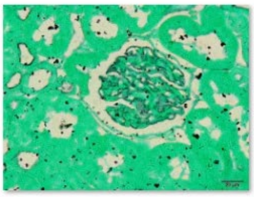
3-1.jpg
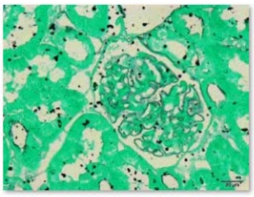
3-2.jpg
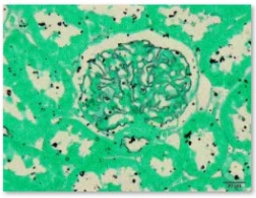
3-3.jpg
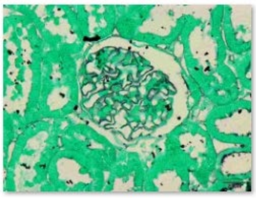
3-4.jpg
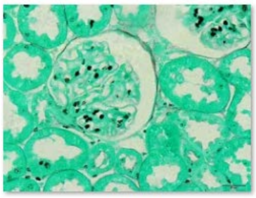
3-5.jpg
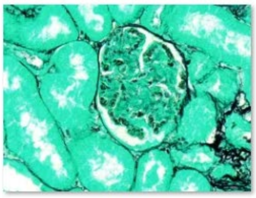
4-1.jpg

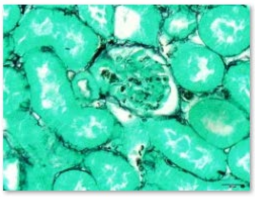
4-2.jpg

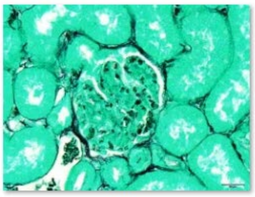
4-3.jpg

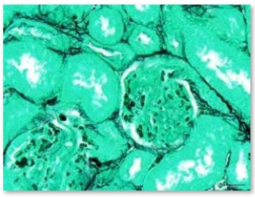
4-4.jpg

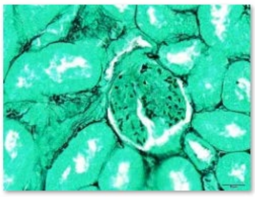
4-5.jpg

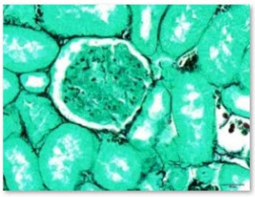
5-1.jpg

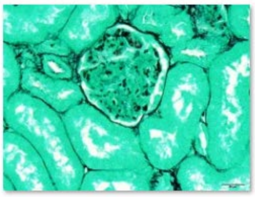
5-2.jpg

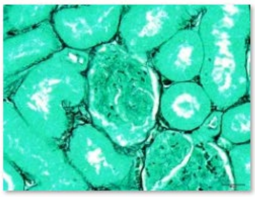
5-3.jpg

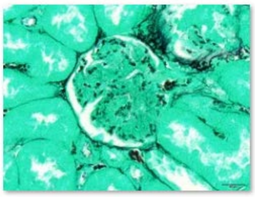
5-4.jpg

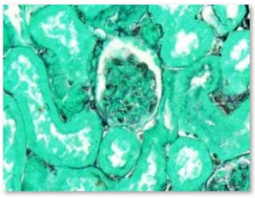
5-5.jpg

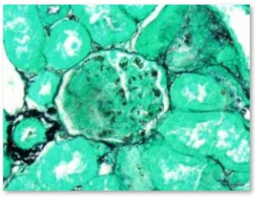
6-1.jpg

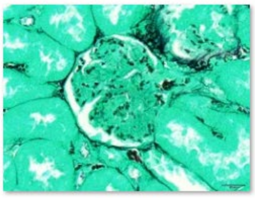
6-2.jpg

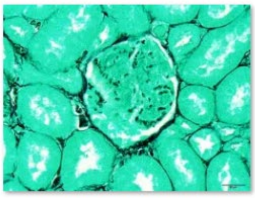
6-3 image in Fig. 1H.jpg

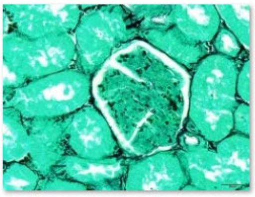
6-4.jpg

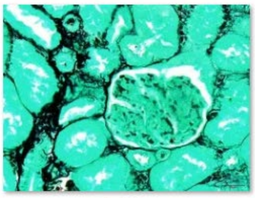
6-5.jpg

PHN

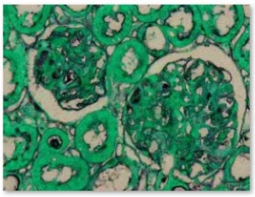
1-1.jpg

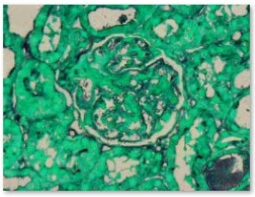
1-2.jpg

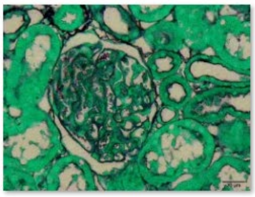
1-3.jpg

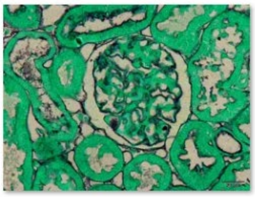
1-4.jpg

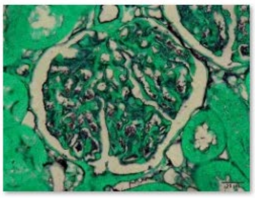
1-5 .jpg

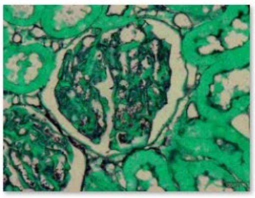
2-1.jpg

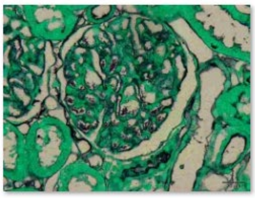
2-2.jpg

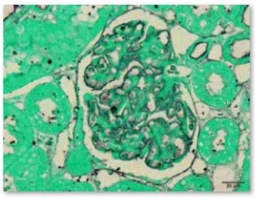
2-3.jpg

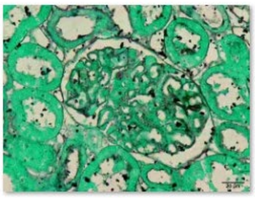
2-4.jpg

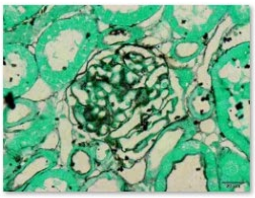
2-5.jpg

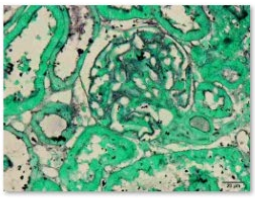
3-1.jpg

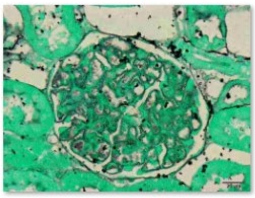
3-2.jpg

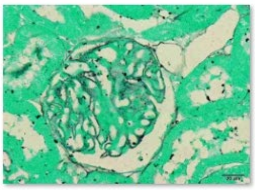
3-3.jpg
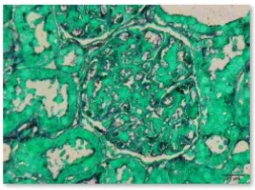
3-4.jpg
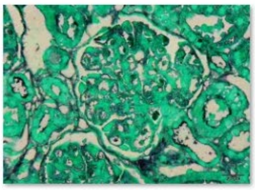
3-5.jpg
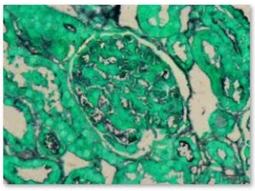
4-1.jpg
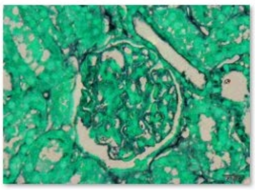
4-2.jpg
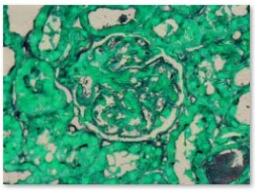
4-3.jpg
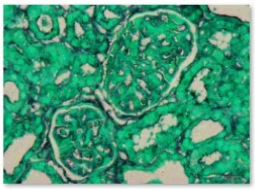
4-4.jpg
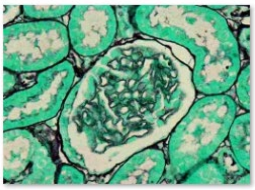
4-5.jpg
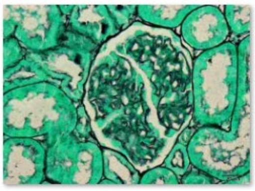
5-1.jpg
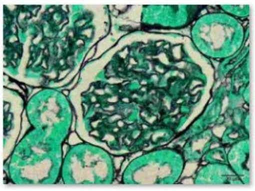
5-2.jpg
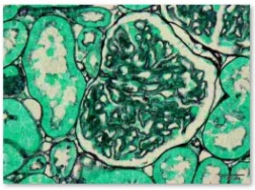
5-3.jpg
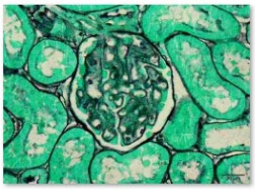
5-4.jpg
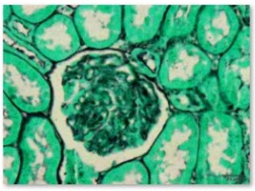
5-5.jpg
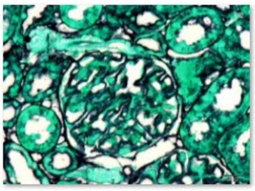
6-1.jpg
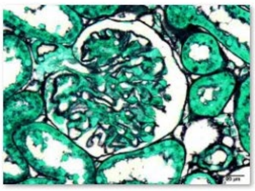
6-2 image in Fig. 1H.jpg
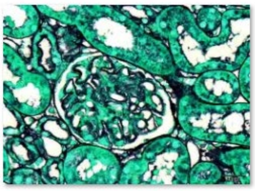
6-3.jpg
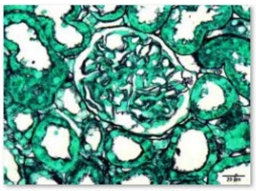
6-4.jpg
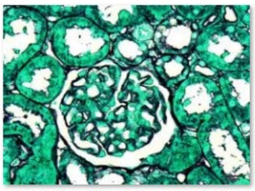
6-5.jpg

SQ-L

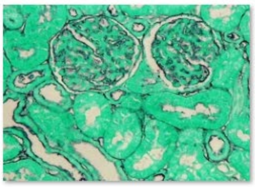
1-1.jpg
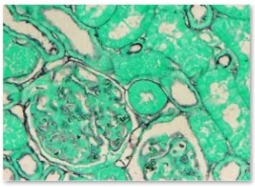
1-2.jpg
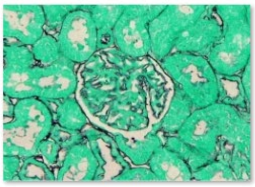
1-3.jpg
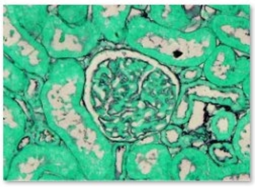
1-4.jpg
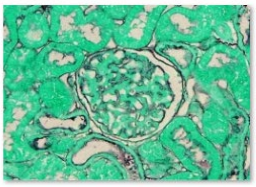
1-5.jpg
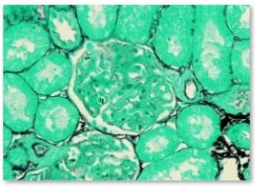
2-1.jpg
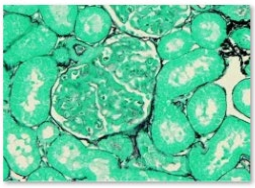
2-2.jpg
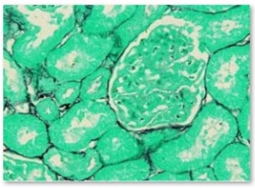
2-3.jpg
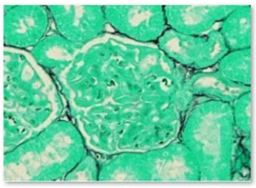
2-4.jpg
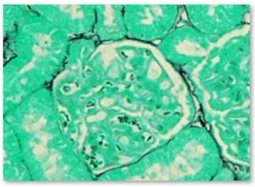
2-5.jpg
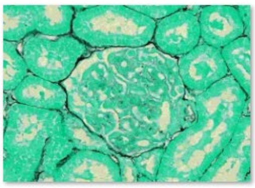
3-1.jpg
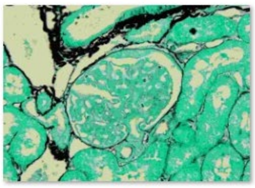
3-2.jpg

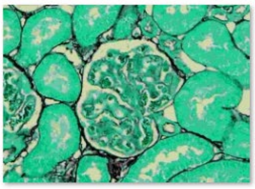
3-3.jpg
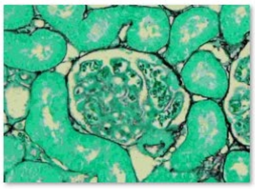
3-4.jpg
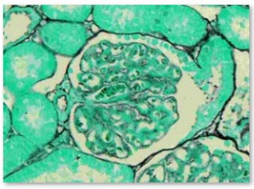
3-5.jpg
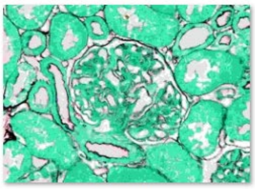
4-1.jpg
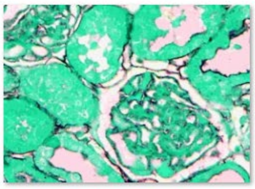
4-2.jpg
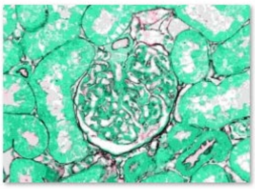
4-3.jpg
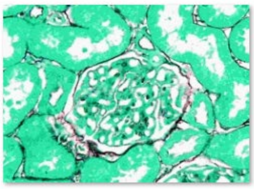
4-4.jpg
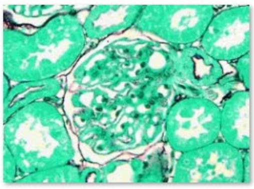
4-5.jpg
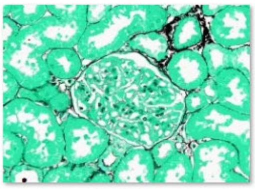
5-1.jpg
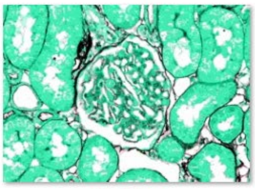
5-2.jpg
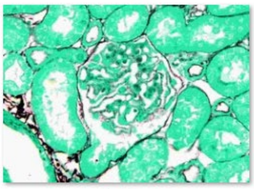
5-3.jpg
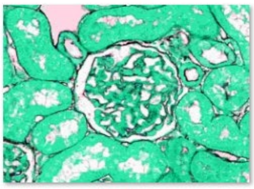
5-4.jpg
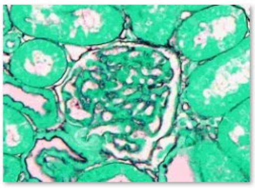
5-5.jpg
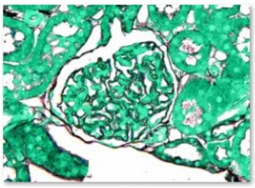
6-1.jpg
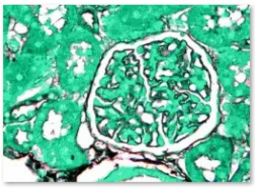
6-2.jpg
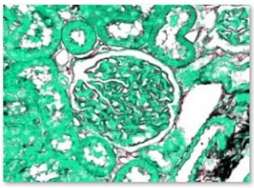
6-3.jpg
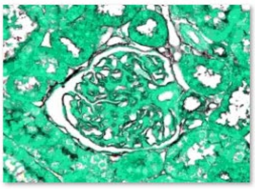
6-4 image in Fig. 1H.jpg
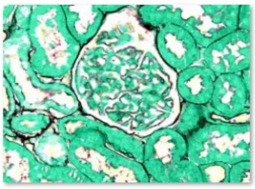
6-5.jpg

SQ-H

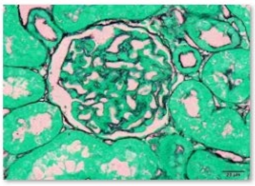
1-1.jpg
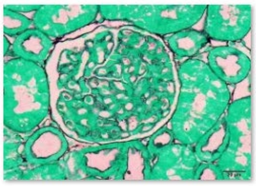
1-2 image in Fig. 1H.jpg
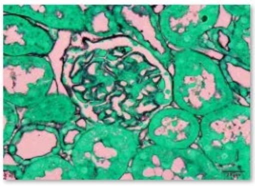
1-3.jpg
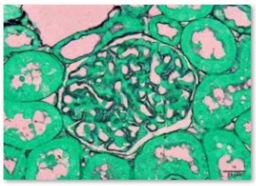
1-4.jpg
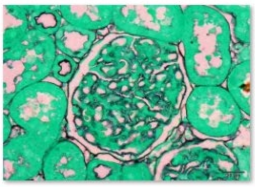
1-5.jpg
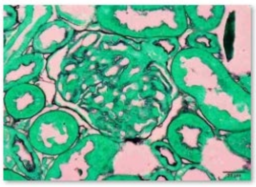
2-1.jpg
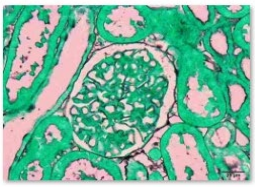
2-2.jpg
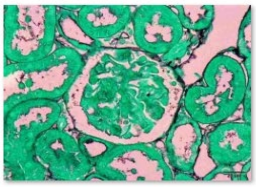
2-3.jpg
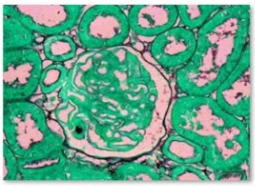
2-4.jpg
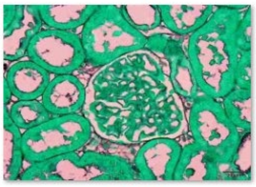
2-5.jpg
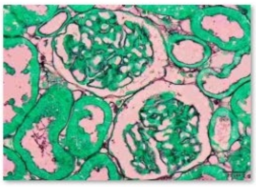
3-1.jpg
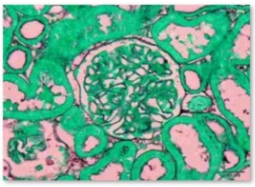
3-2.jpg

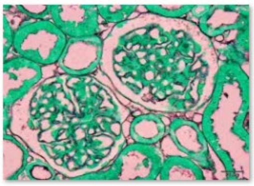
3-3.jpg
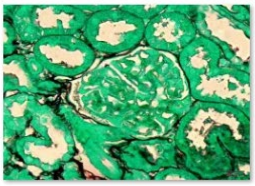
3-4.jpg
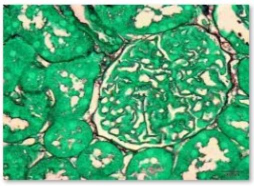
3-5.jpg
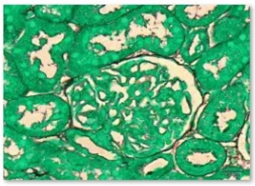
4-1.jpg
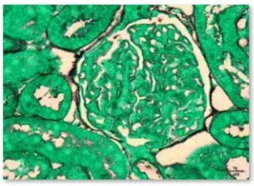
4-2.jpg
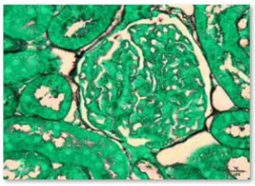
4-3.jpg
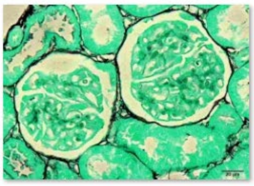
4-4.jpg
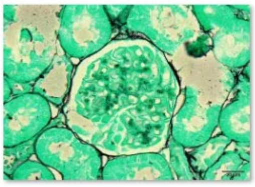
4-5.jpg
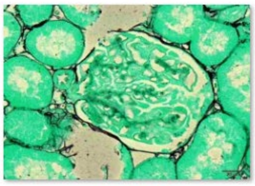
5-1.jpg
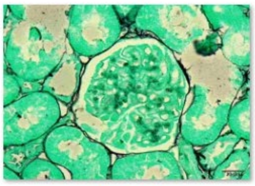
5-2.jpg
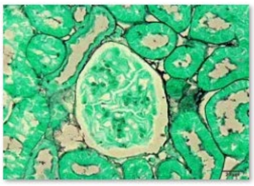
5-3.jpg
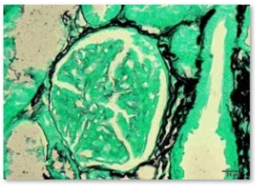
5-4.jpg
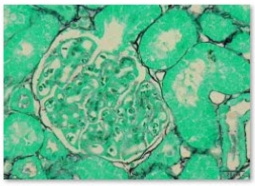
5-5.jpg
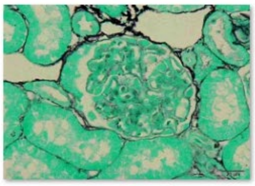
6-1.jpg
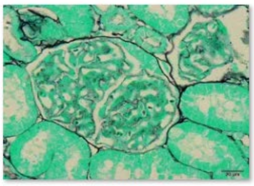
6-2.jpg
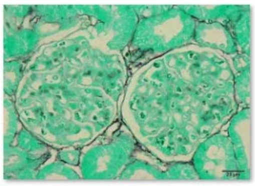
6-3.jpg
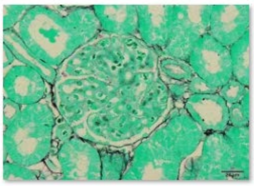
6-4.jpg
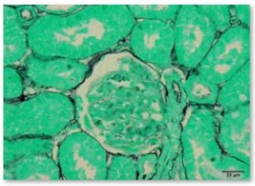
6-5.jpg

TAC

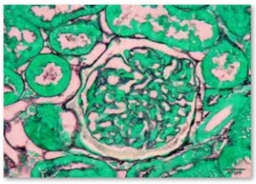
1-1.jpg
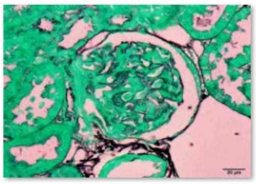
1-2.jpg
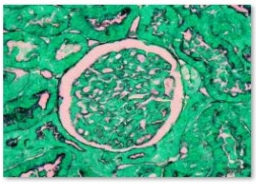
1-3.jpg
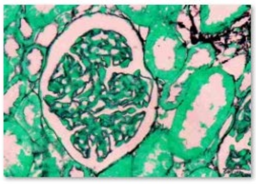
1-4.jpg
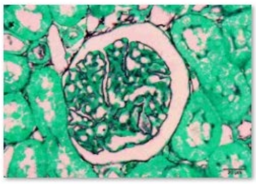
1-5.jpg
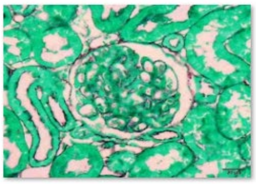
2-1.jpg
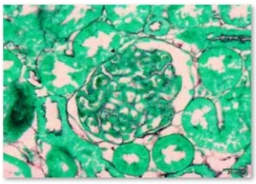
2-2.jpg
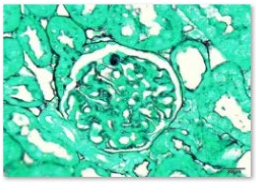
2-3.jpg
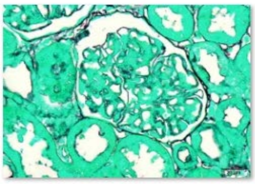
2-4.jpg
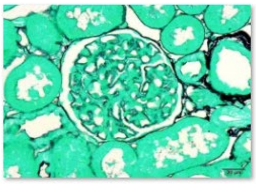
2-5 image in Fig. 1H.jpg
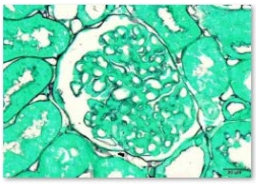
3-1.jpg
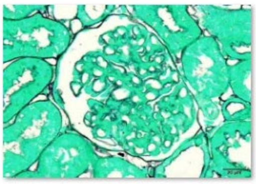
3-2.jpg

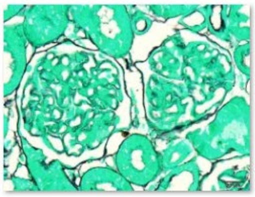
3-3.jpg

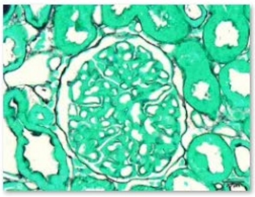
3-4.jpg

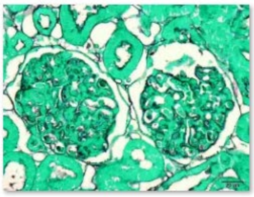
3-5.jpg

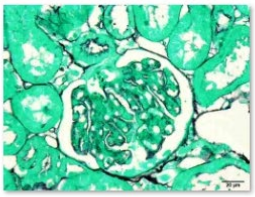
4-1.jpg

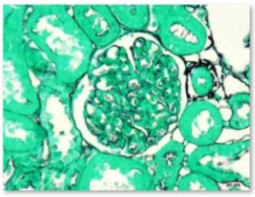
4-2.jpg

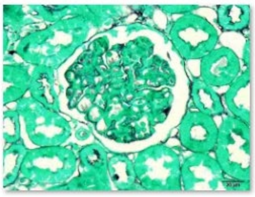
4-3.jpg

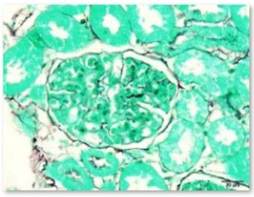
4-4.jpg

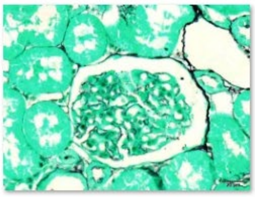
4-5.jpg

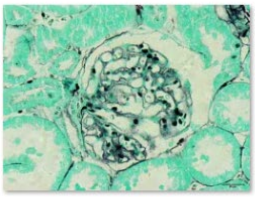
5-1.jpg

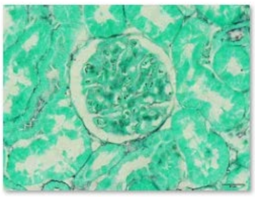
5-2.jpg

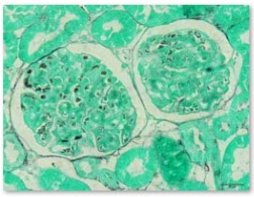
5-3.jpg

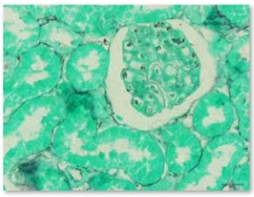
5-4.jpg

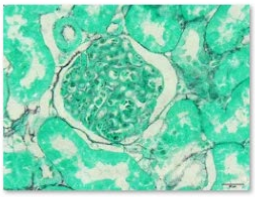
5-5.jpg

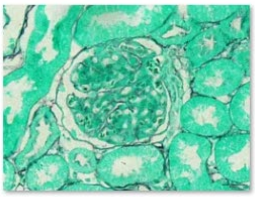
6-1.jpg

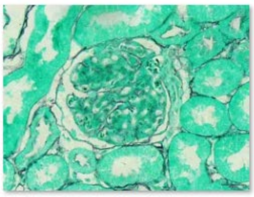
6-2.jpg

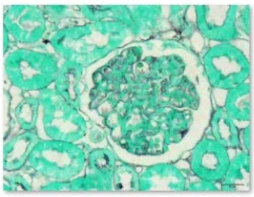
6-3.jpg

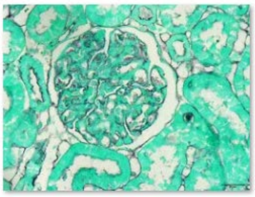
6-4.jpg

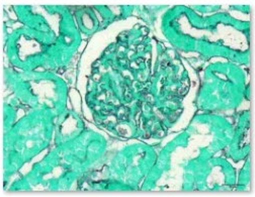
6-5.jpg

Original data for Fig. 2I-PASM mean density

No.	CON	PHN	SQ-L	SQ-H	TAC
1-1	0. 206649183	0. 487572709	0. 364952803	0. 490951174	0. 551117685
1-2	0. 041281732	0. 372331106	0. 58548428	0. 30874378	0. 404901672
1-3	0. 126396074	0. 587308242	0. 252618557	0. 612801244	0. 357019082
1-4	0. 057872808	0. 509461245	0. 422999163	0. 561507819	0. 631962948
1-5	0. 006461285	0. 582149239	0. 362065824	0. 46606522	0. 630203631
2-1	0. 037464991	0. 61984637	0. 432124042	0. 385293417	0. 350122301
2-2	0. 064803291	0. 589814595	0. 496042333	0. 43780509	0. 350082383
2-3	0. 195780581	0. 496360027	0. 257486844	0. 239158863	0. 538551587
2-4	0. 503639314	0. 518787766	0. 245540502	0. 27736226	0. 458611949
2-5	0. 357365965	0. 760977995	0. 356686703	0. 423895674	0. 46010679
3-1	0. 24942133	0. 580935626	0. 392476102	0. 499218654	0. 359802265
3-2	0. 215272606	0. 475264906	0. 381172376	0. 498367248	0. 361000758
3-3	0. 254638239	0. 365416135	0. 540054264	0. 474495158	0. 399095261
3-4	0. 478800014	0. 460016777	0. 556437922	0. 296510672	0. 310272711
3-5	0. 34562374	0. 445006203	0. 535184903	0. 468024988	0. 529987969
4-1	0. 276632987	0. 407355287	0. 1899851	0. 441326629	0. 470793799
4-2	0. 251246414	0. 407225733	0. 197313803	0. 400898732	0. 457117461
4-3	0. 256764616	0. 148472269	0. 504872002	0. 400980855	0. 367454495
4-4	0. 200485017	0. 316029337	0. 370428392	0. 141128647	0. 240474826
4-5	0. 239461358	0. 678762458	0. 152453134	0. 032582848	0. 333441806
5-1	0. 174038693	0. 655928294	0. 150741601	0. 177173389	0. 606143997
5-2	0. 234911087	0. 784431271	0. 409776593	0. 201307493	0. 226302692
5-3	0. 101304603	0. 768987032	0. 3572437	0. 122054736	0. 182963662
5-4	0. 195944852	0. 680500352	0. 500353834	0. 107930451	0. 112116755
5-5	0. 361874747	0. 55786517	0. 55946974	0. 135896549	0. 20205586
6-1	0. 233895142	0. 812730212	0. 514919732	0. 123408246	0. 09177161
6-2	0. 23032698	0. 755010573	0. 397407195	0. 195151123	0. 226524678
6-3	0. 173006637	0. 784893678	0. 271962472	0. 111926789	0. 360536478
6-4	0. 282605492	0. 767442614	0. 580938719	0. 089945478	0. 466114405
6-5	0. 188863324	0. 781553171	0. 469596712	0. 055781061	0. 512461865

Original Image for Fig. 3A-C5b-9

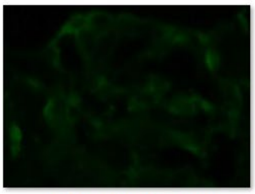
C5b-9-CON1-1.jpg

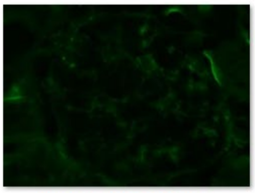
C5b-9-CON1-2.jpg

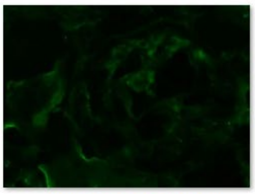
C5b-9-CON1-3.jpg

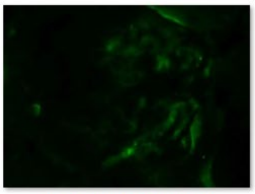
C5b-9-CON1-4.jpg

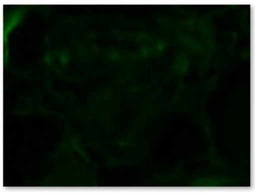
C5b-9-CON1-5.jpg

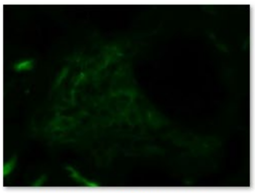
C5b-9-CON2-1.jpg

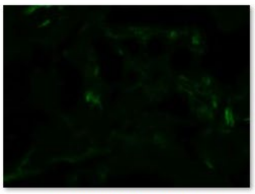
C5b-9-CON2-2.jpg

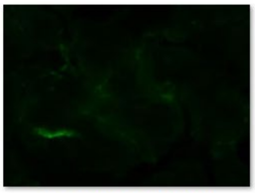
C5b-9-CON2-3.jpg

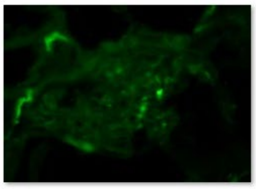
C5b-9-CON2-4.jpg

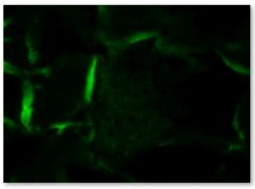
C5b-9-CON2-5.jpg

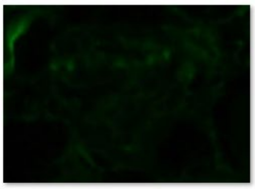
C5b-9-CON3-1.jpg

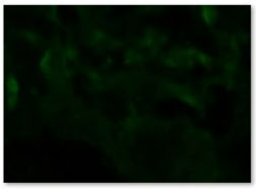
C5b-9-CON3-2.jpg

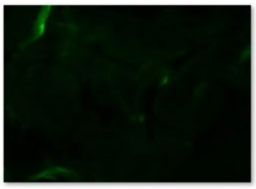
C5b-9-CON3-3.jpg

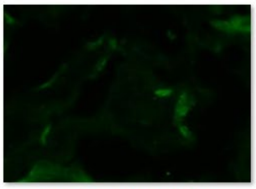
C5b-9-CON3-4.jpg

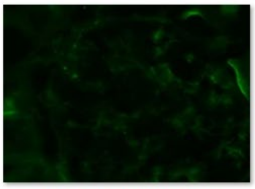
C5b-9-CON3-5.jpg

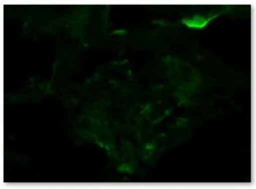
C5b-9-CON4-1 image in Fig. 3A.jpg

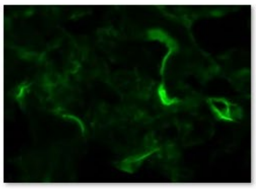
C5b-9-CON4-2.jpg

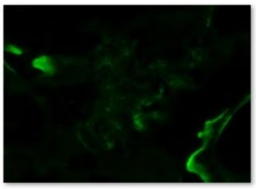
C5b-9-CON4-3.jpg

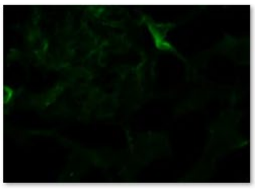
C5b-9-CON4-4.jpg

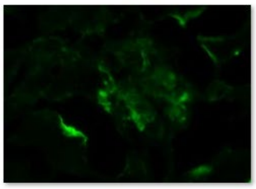
C5b-9-CON4-5.jpg

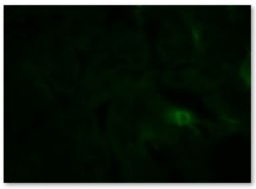
C5b-9-CON5-1.jpg

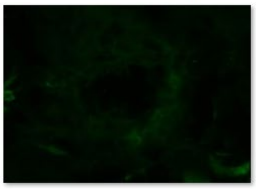
C5b-9-CON5-2.jpg

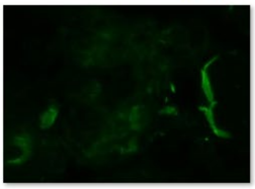
C5b-9-CON5-3.jpg

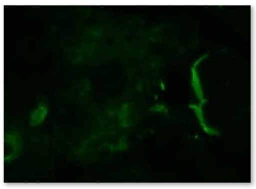
C5b-9-CON5-4.jpg

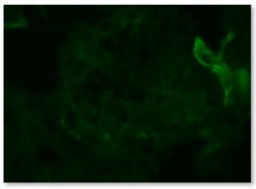
C5b-9-CON5-5.jpg

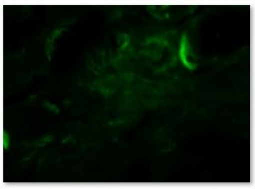
C5b-9-CON6-1.jpg

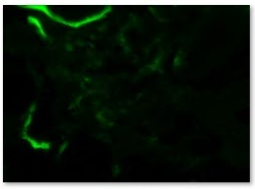
C5b-9-CON6-2.jpg

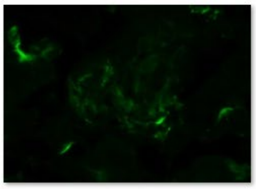
C5b-9-CON6-3.jpg

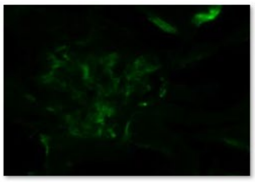
C5b-9-CON6-4.jpg

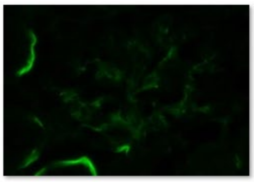
C5b-9-CON6-5.jpg

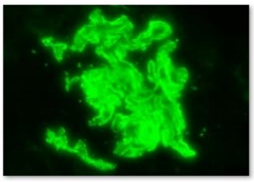
C5b-9-PHN1-1.jpg

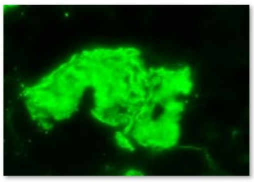
C5b-9-PHN1-2.jpg

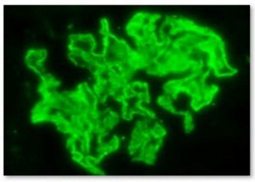
C5b-9-PHN1-3.jpg

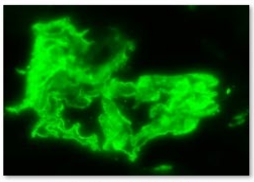
C5b-9-PHN1-4.jpg

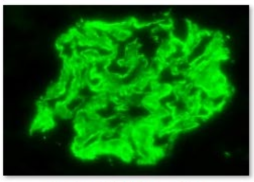
C5b-9-PHN1-5.jpg

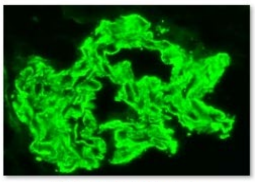
C5b-9-PHN2-1.jpg

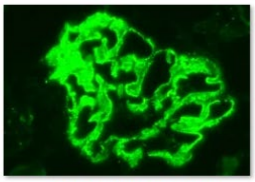
C5b-9-PHN2-2.jpg

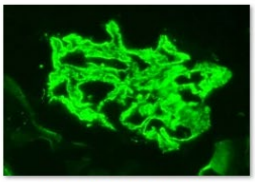
C5b-9-PHN2-3.jpg

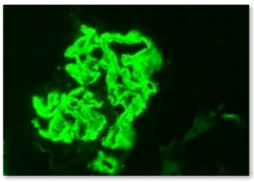
C5b-9-PHN2-4.jpg

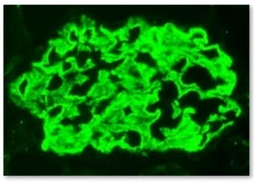
C5b-9-PHN2-5 image in Fig. 3A.jpg

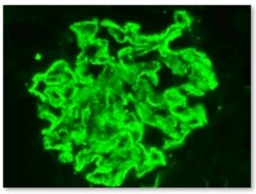
C5b-9-PHN3-1.jpg
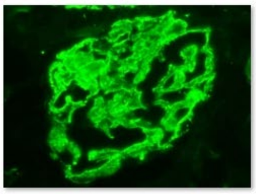
C5b-9-PHN3-2.jpg
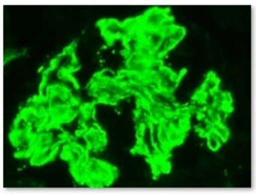
C5b-9-PHN3-3.jpg
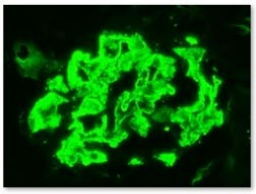
C5b-9-PHN3-4.jpg
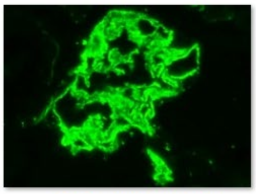
C5b-9-PHN3-5.jpg
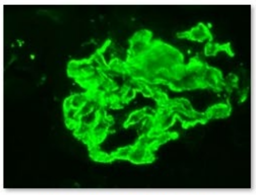
C5b-9-PHN4-1.jpg
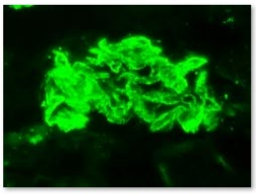
C5b-9-PHN4-2.jpg
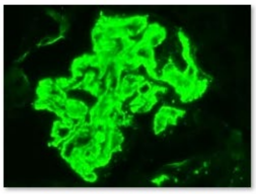
C5b-9-PHN4-3.jpg
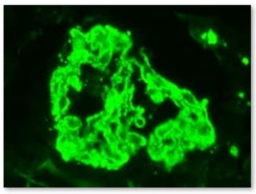
C5b-9-PHN4-4.jpg
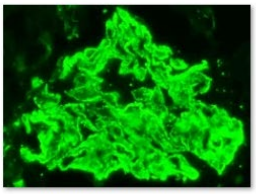
C5b-9-PHN4-5.jpg
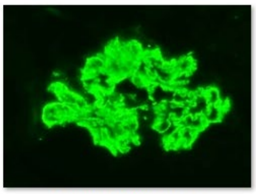
C5b-9-PHN5-1.jpg
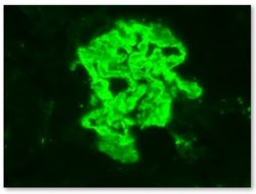
C5b-9-PHN5-2.jpg
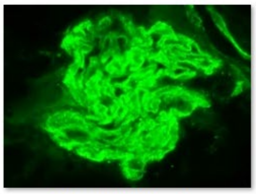
C5b-9-PHN5-3.jpg
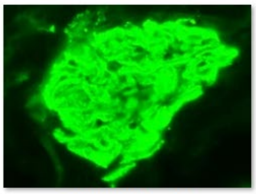
C5b-9-PHN5-4.jpg
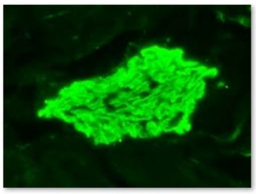
C5b-9-PHN5-5.jpg
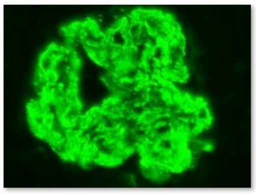
C5b-9-PHN6-1.jpg
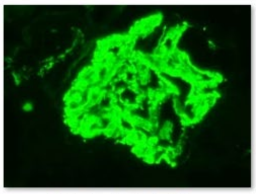
C5b-9-PHN6-2.jpg
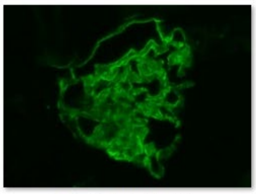
C5b-9-PHN6-3.jpg
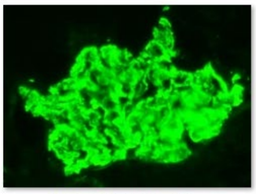
C5b-9-PHN6-4.jpg
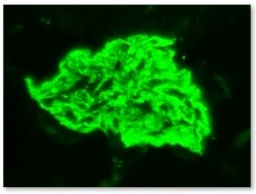
C5b-9-PHN6-5.jpg
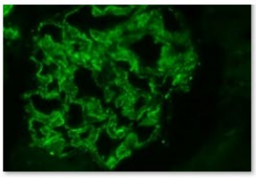
C5b-9-SQ-H-1-1.jpg
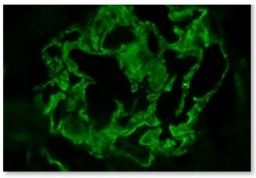
C5b-9-SQ-H-1-2.jpg
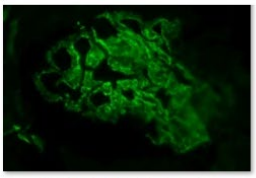
C5b-9-SQ-H-1-3.jpg
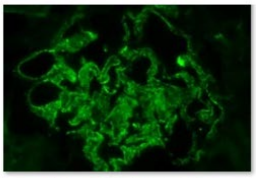
C5b-9-SQ-H-1-4.jpg
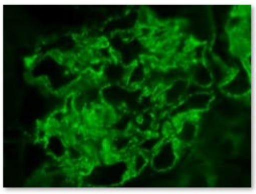
C5b-9-SQ-H-1-5.jpg
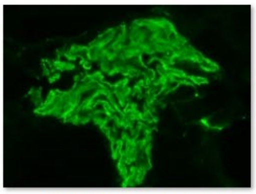
C5b-9-SQ-H-2-1.jpg
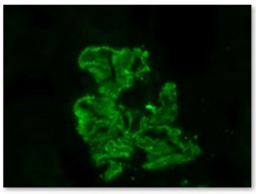
C5b-9-SQ-H-2-2.jpg
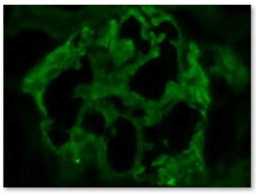
C5b-9-SQ-H-2-3.jpg
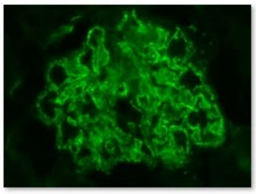
C5b-9-SQ-H-2-4.jpg
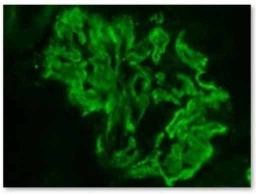
C5b-9-SQ-H-2-5.jpg
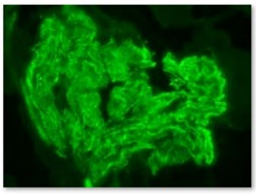
C5b-9-SQ-H-3-1.jpg
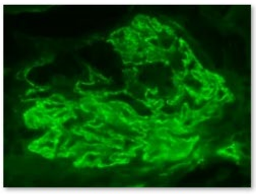
C5b-9-SQ-H-3-2.jpg

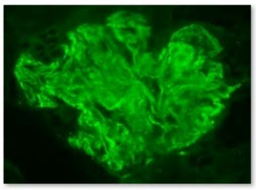
C5b-9-SQ-H-3-3.jpg

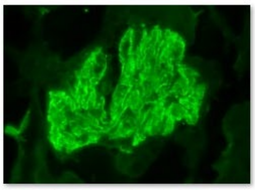
C5b-9-SQ-H-3-4.jpg

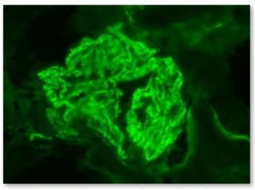
C5b-9-SQ-H-3-5.jpg

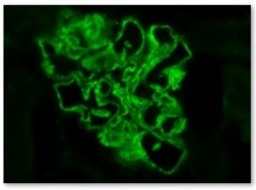
C5b-9-SQ-H-4-1.jpg

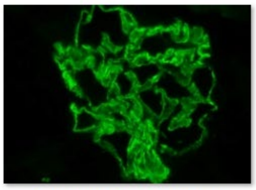
C5b-9-SQ-H-4-2.jpg

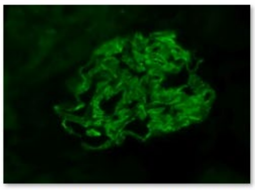
C5b-9-SQ-H-4-3 image in Fig. 3A.jpg

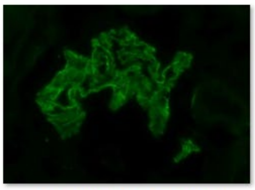
C5b-9-SQ-H-4-4.jpg

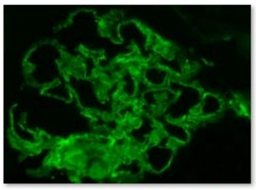
C5b-9-SQ-H-4-5.jpg

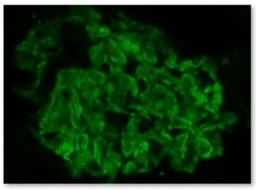
C5b-9-SQ-H-5-1.jpg

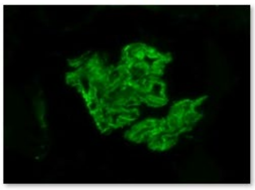
C5b-9-SQ-H-5-2.jpg

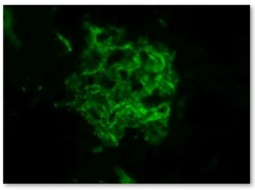
C5b-9-SQ-H-5-3.jpg

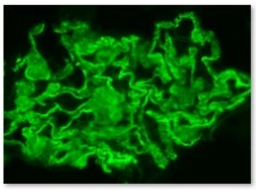
C5b-9-SQ-H-5-4.jpg

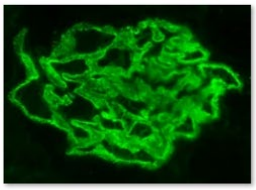
C5b-9-SQ-H-5-5.jpg

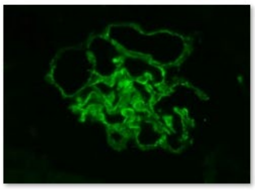
C5b-9-SQ-H-6-1.jpg

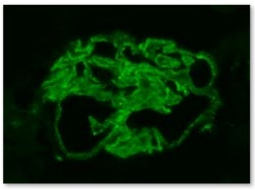
C5b-9-SQ-H-6-2.jpg

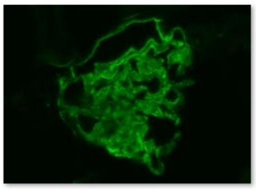
C5b-9-SQ-H-6-3.jpg

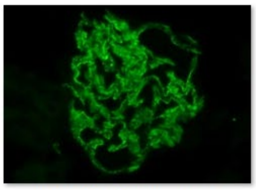
C5b-9-SQ-H-6-4.jpg

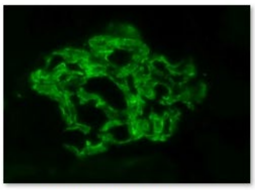
C5b-9-SQ-H-6-5.jpg

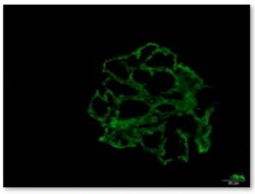
C5b-9-SQ-L-1-1.jpg

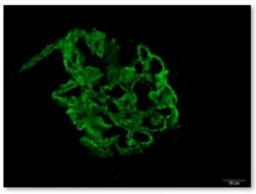
C5b-9-SQ-L-1-2.jpg

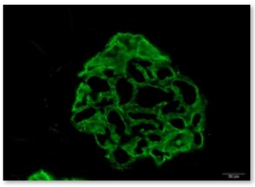
C5b-9-SQ-L-1-3.jpg

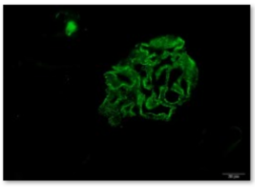
C5b-9-SQ-L-1-4.jpg

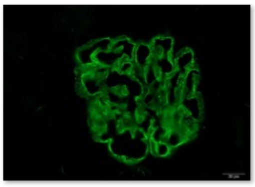
C5b-9-SQ-L-1-5.jpg

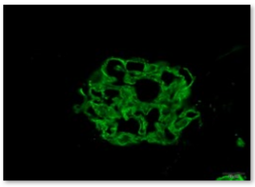
C5b-9-SQ-L-2-1.jpg

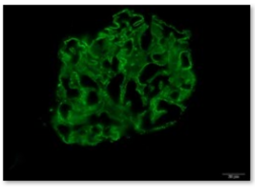
C5b-9-SQ-L-2-2.jpg

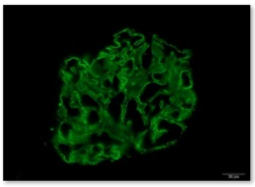
C5b-9-SQ-L-2-3.jpg

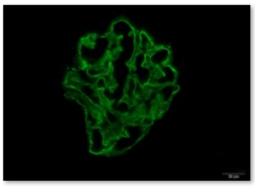
C5b-9-SQ-L-2-4.jpg

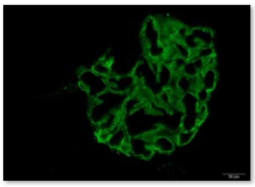
C5b-9-SQ-L-2-5.jpg

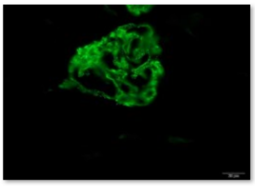
C5b-9-SQ-L-3-1.jpg

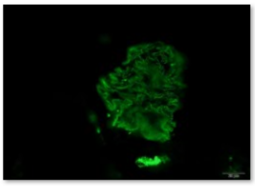
C5b-9-SQ-L-3-2.jpg

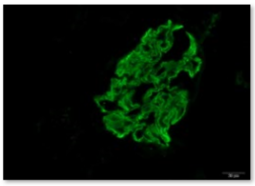
C5b-9-SQ-L-3-3.jpg

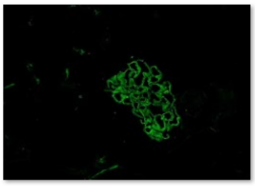
C5b-9-SQ-L-3-4.jpg

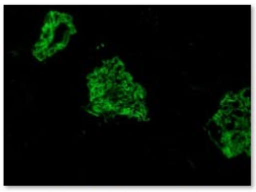
C5b-9-SQ-L-4-4.jpg

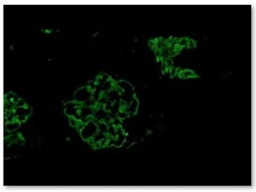
C5b-9-SQ-L-4-5.jpg

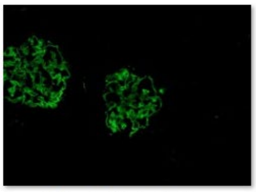
C5b-9-SQ-L-5-1.jpg

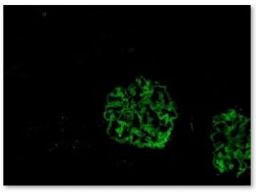
C5b-9-SQ-L-5-2.jpg

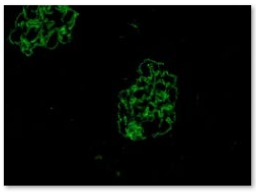
C5b-9-SQ-L-5-3.jpg

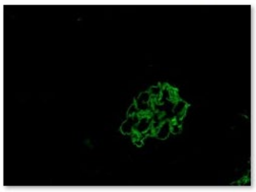
C5b-9-SQ-L-5-4.jpg

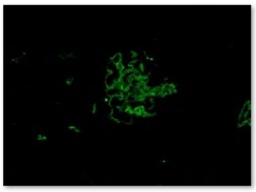
C5b-9-SQ-L-5-5.jpg

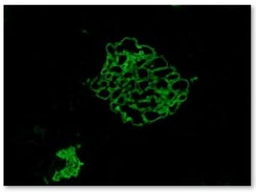
C5b-9-SQ-L-6-1.jpg

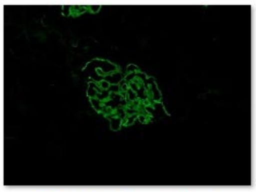
C5b-9-SQ-L-6-2.jpg

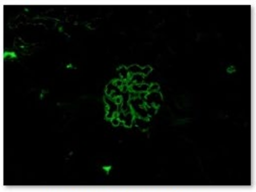
C5b-9-SQ-L-6-3.jpg

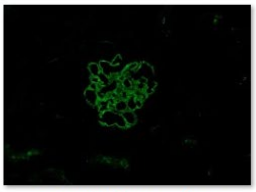
C5b-9-SQ-L-6-4.jpg

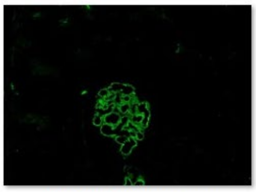
C5b-9-SQ-L-6-5.jpg

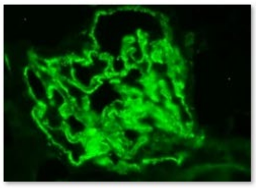
C5b-9-TAC1-1.jpg

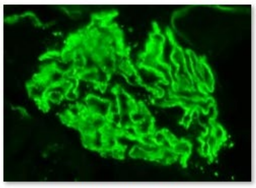
C5b-9-TAC1-2.jpg

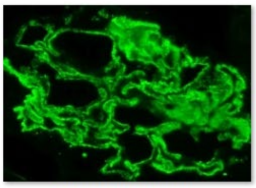
C5b-9-TAC1-3.jpg

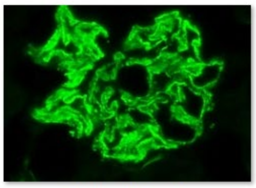
C5b-9-TAC1-4.jpg

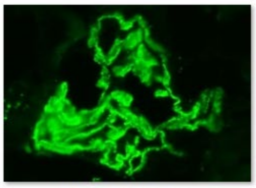
C5b-9-TAC1-5.jpg

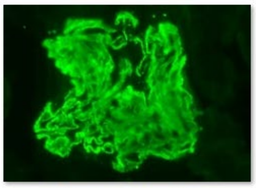
C5b-9-TAC2-1.jpg

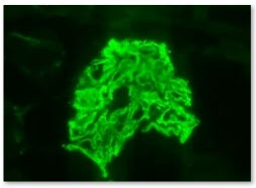
C5b-9-TAC2-2.jpg

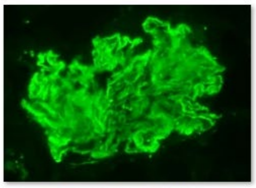
C5b-9-TAC2-3.jpg

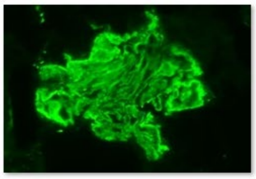
C5b-9-TAC2-4.jpg

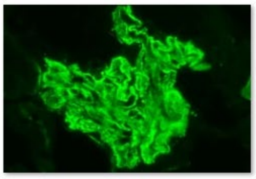
C5b-9-TAC2-5.jpg

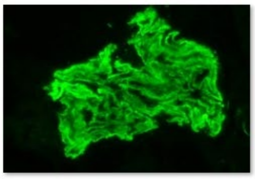
C5b-9-TAC3-1.jpg

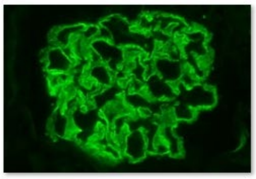
C5b-9-TAC3-2 image in Fig. 3A.jpg

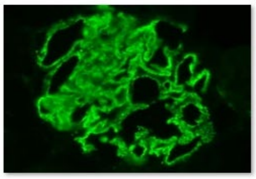
C5b-9-TAC3-3.jpg

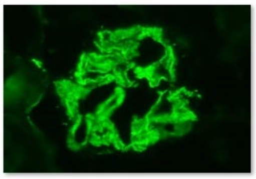
C5b-9-TAC3-4.jpg

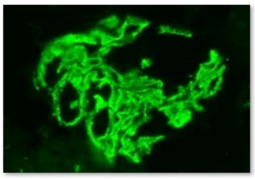
C5b-9-TAC3-5.jpg

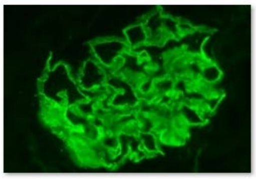
C5b-9-TAC4-1.jpg

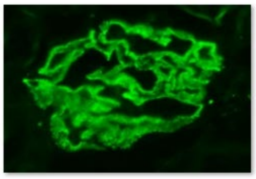
C5b-9-TAC4-2.jpg

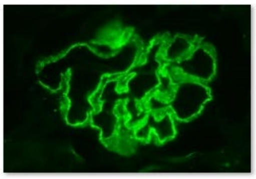
C5b-9-TAC4-3.jpg

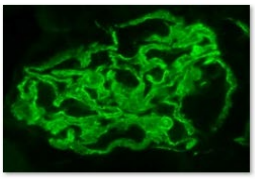
C5b-9-TAC4-4.jpg

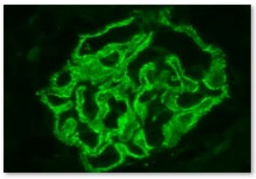
C5b-9-TAC4-5.jpg

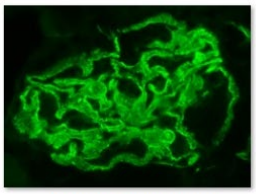
C5b-9-TAC5-1.jpg

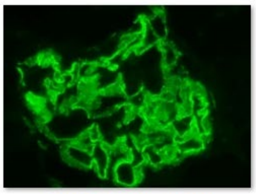
C5b-9-TAC5-2.jpg

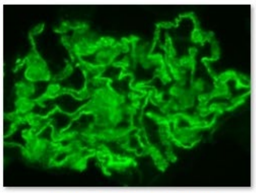
C5b-9-TAC5-3.jpg

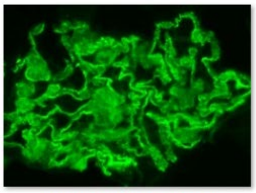
C5b-9-TAC5-4.jpg

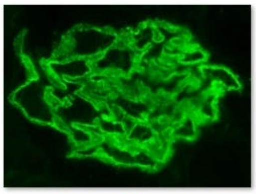
C5b-9-TAC5-5.jpg

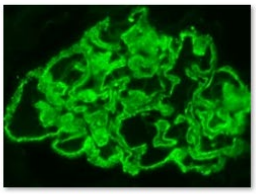
C5b-9-TAC6-1.jpg

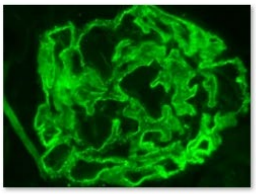
C5b-9-TAC6-2.jpg

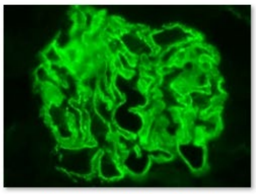
C5b-9-TAC6-3.jpg

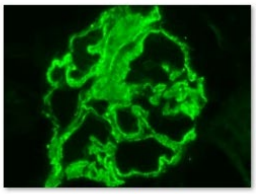
C5b-9-TAC6-4.jpg

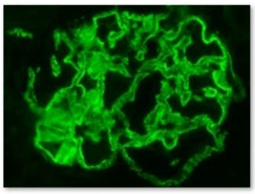
C5b-9-TAC6-5.jpg

Original Image for Fig. 3A-Hoechst33342

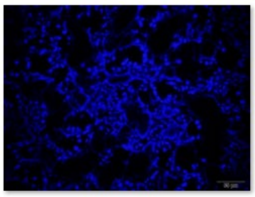
CON 1-1.jpg

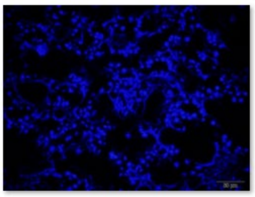
CON 1-2.jpg

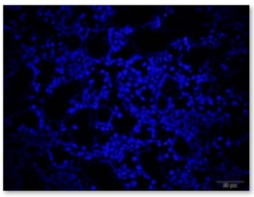
CON 1-3.jpg

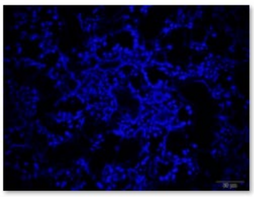
CON 1-4.jpg

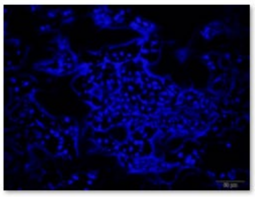
CON 2-1.jpg

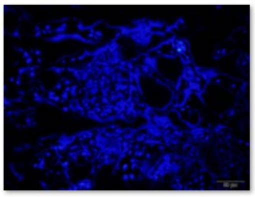
CON 2-2.jpg

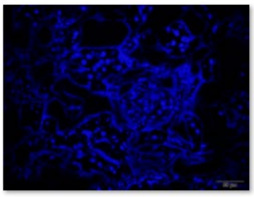
CON 2-3 image in Fig. 2A.jpg

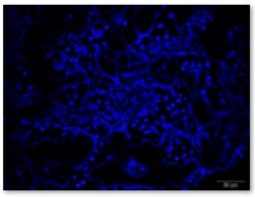
CON 2-4.jpg

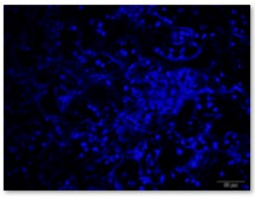
CON 3-1.jpg

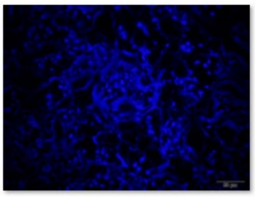
CON 3-2.jpg

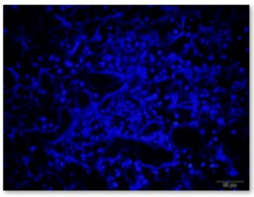
CON 3-3.jpg

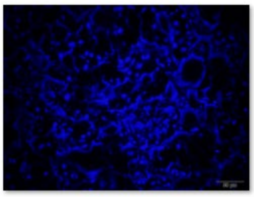
CON 3-4.jpg

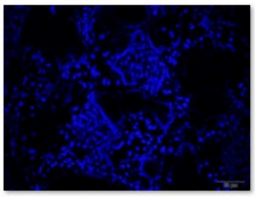
CON 4-1.jpg

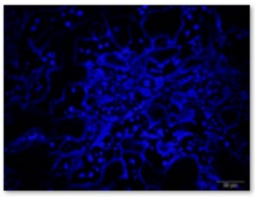
CON 4-2.jpg

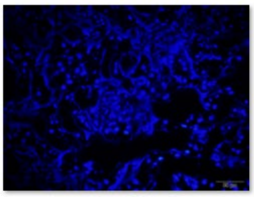
CON 4-3.jpg

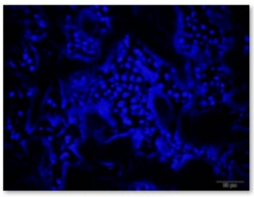
CON 4-4.jpg

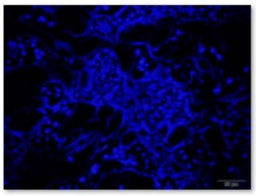
CON 5-1.jpg

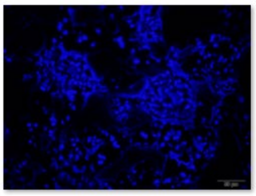
CON 5-2.jpg

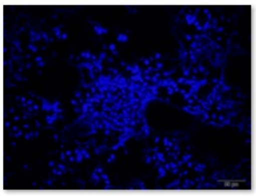
CON 5-3.jpg

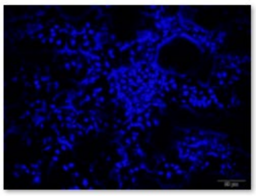
CON 5-4.jpg

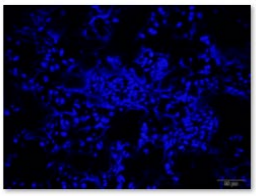
CON 6-1.jpg

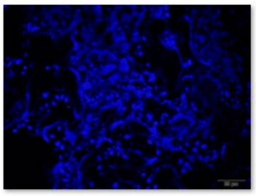
CON 6-2.jpg

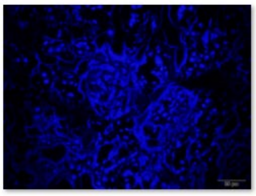
CON 6-3.jpg

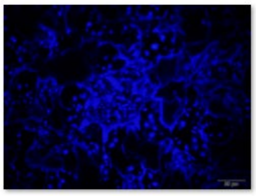
CON 6-4.jpg

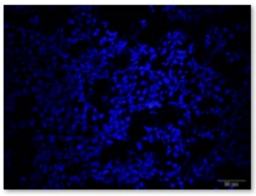
PHN 1-1.jpg

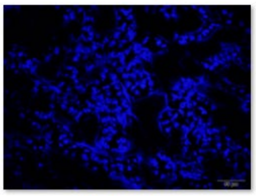
PHN 1-2.jpg

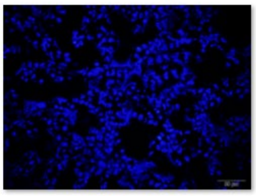
PHN 1-3.jpg

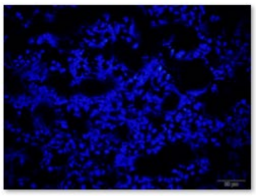
PHN 1-4.jpg

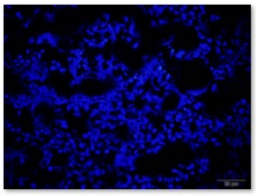
PHN 2-1.jpg

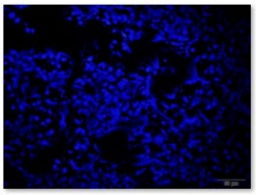
PHN 2-2.jpg

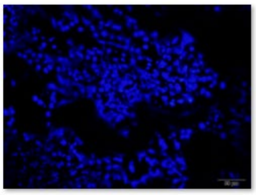
PHN 2-3.jpg

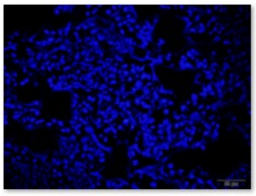
PHN 2-4.jpg

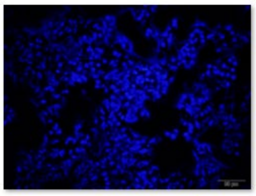
PHN 3-1.jpg

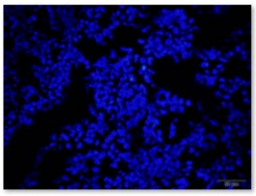
PHN 3-2.jpg

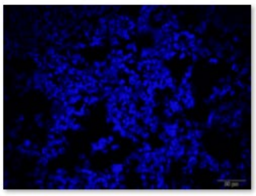
PHN 3-3.jpg

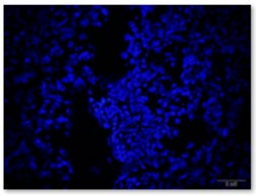
PHN 3-4.jpg

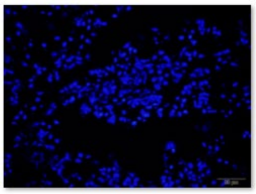
PHN 4-1.jpg

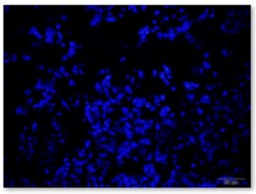
PHN 4-2.jpg

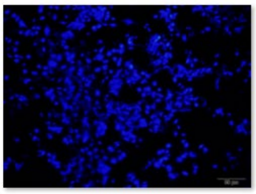
PHN 4-3.jpg

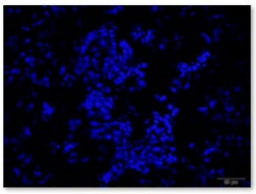
PHN 4-4.jpg

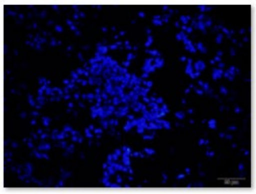
PHN 5-1.jpg

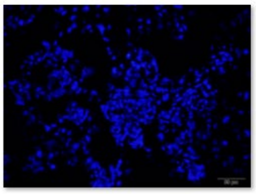
PHN 5-2.jpg

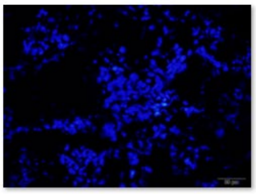
PHN 5-3 image in Fig. 2A.jpg

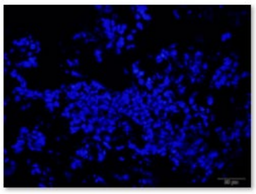
PHN 5-4.jpg

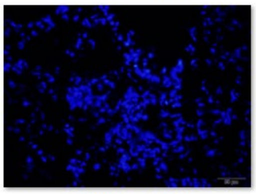
PHN 6-1.jpg

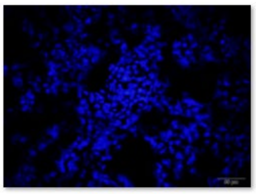
PHN 6-2.jpg

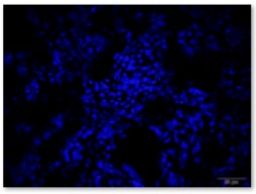
PHN 6-3.jpg

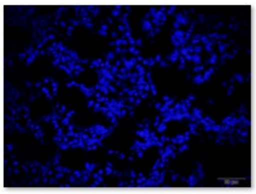
PHN 6-4.jpg

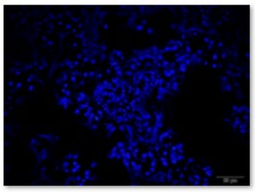
SQ-H 1-1.jpg

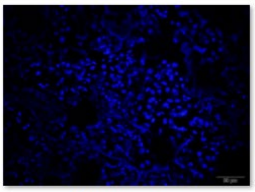
SQ-H 1-2.jpg

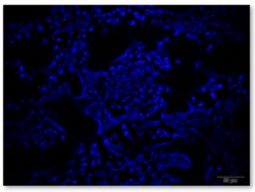
SQ-H 1-3.jpg

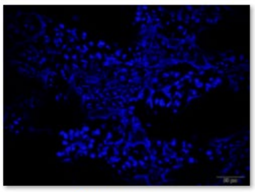
SQ-H 1-4.jpg

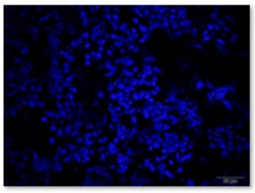
SQ-H 2-1.jpg

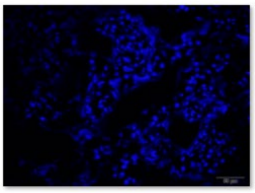
SQ-H 2-2.jpg

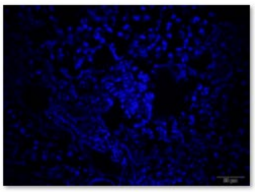
SQ-H 2-3.jpg

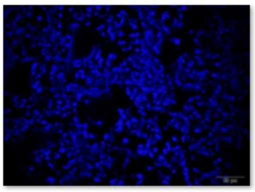
SQ-H 2-4.jpg

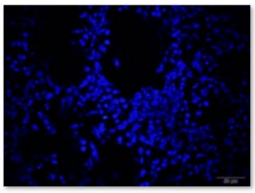
SQ-H 3-1.jpg

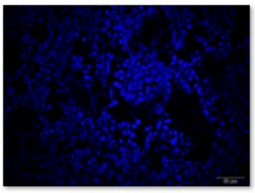
SQ-H 3-2.jpg

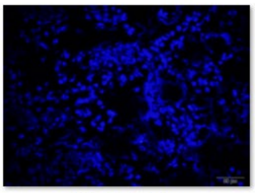
SQ-H 3-3.jpg

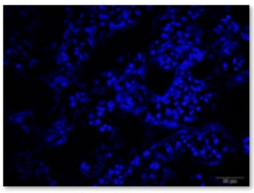
SQ-H 3-4.jpg

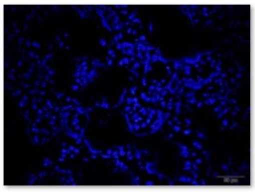
SQ-H 4-1 image in Fig. 2A.jpg

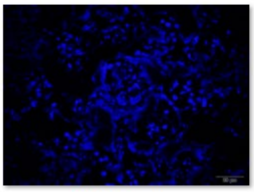
SQ-H 4-2.jpg

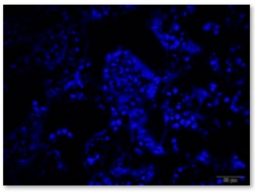
SQ-H 4-3.jpg

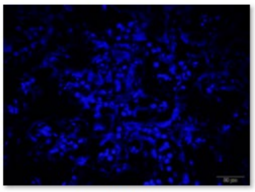
SQ-H 4-4.jpg

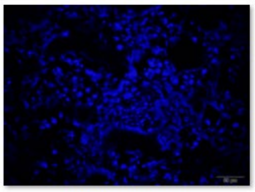
SQ-H 5-1.jpg

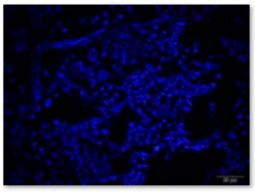
SQ-H 5-2.jpg

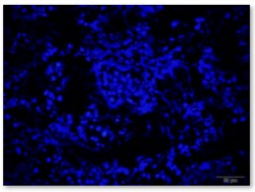
SQ-H 5-3.jpg

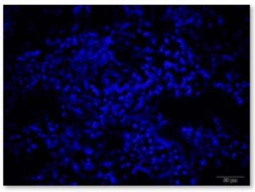
SQ-H 5-4.jpg

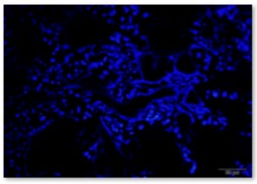
SQ-H 6-1.jpg

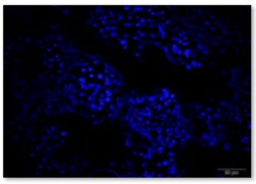
SQ-H 6-2.jpg

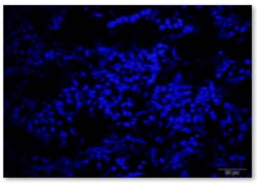
SQ-H 6-3.jpg

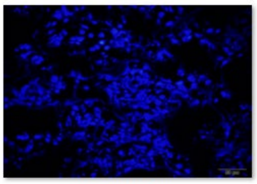
SQ-H 6-4.jpg

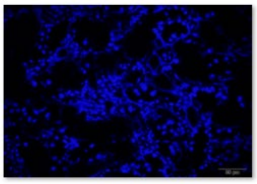
SQ-L 1-1.jpg

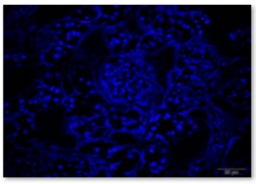
SQ-L 1-2.jpg

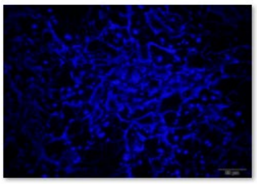
SQ-L 1-3.jpg

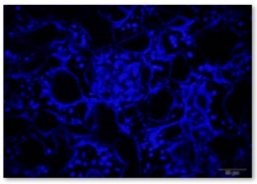
SQ-L 1-4.jpg

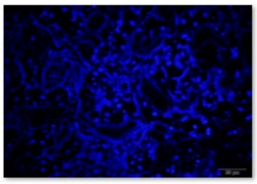
SQ-L 2-1.jpg

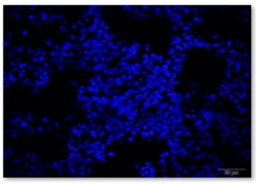
SQ-L 2-2.jpg

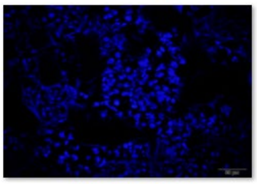
SQ-L 2-3.jpg

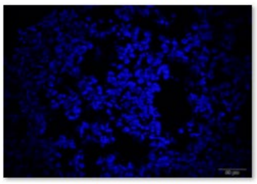
SQ-L 2-4.jpg

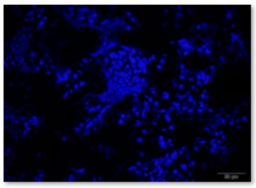
SQ-L 3-1.jpg

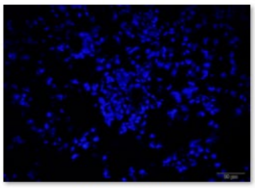
SQ-L 3-2.jpg

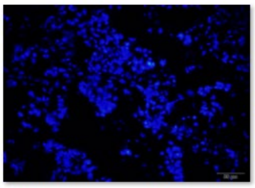
SQ-L 3-3 image in Fig. 2A.jpg

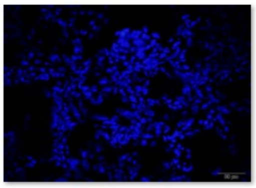
SQ-L 3-4.jpg

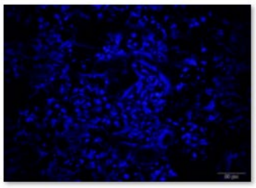
SQ-L 4-1.jpg

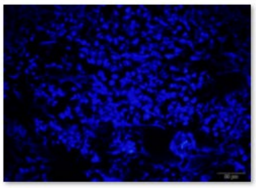
SQ-L 4-2.jpg

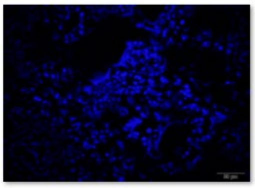
SQ-L 4-3.jpg

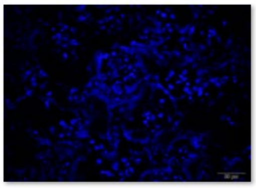
SQ-L 4-4.jpg

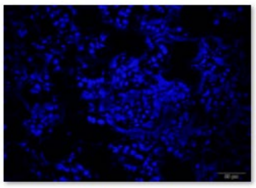
SQ-L 5-1.jpg

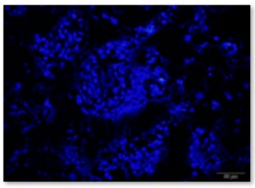
SQ-L 5-2.jpg

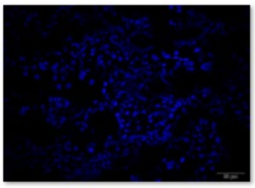
SQ-L 5-3.jpg

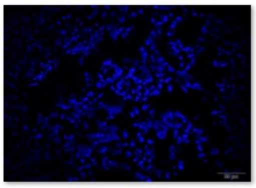
SQ-L 5-4.jpg

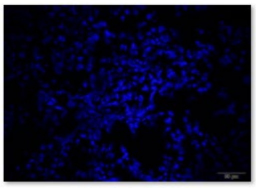
SQ-L 6-1.jpg

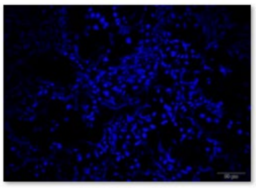
SQ-L 6-2.jpg

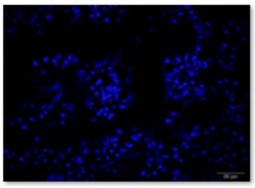
SQ-L 6-3.jpg

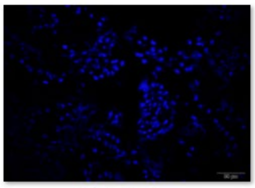
SQ-L 6-4.jpg

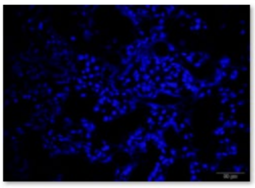
TAC 1-1.jpg

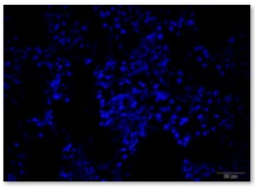
TAC 1-2.jpg

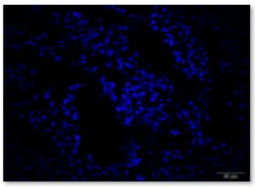
TAC 1-3.jpg

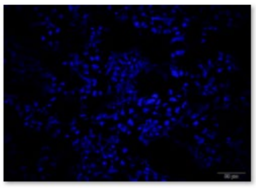
TAC 1-4.jpg

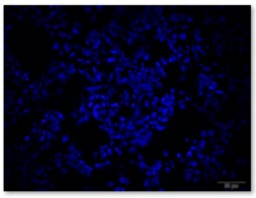
TAC 2-1.jpg

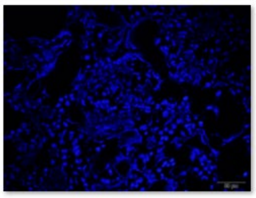
TAC 2-2.jpg

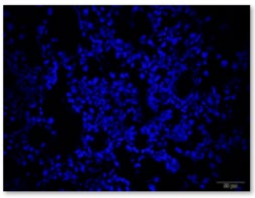
TAC 2-3.jpg

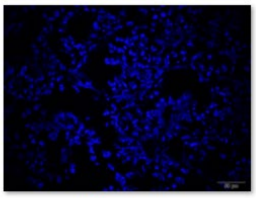
TAC 2-4.jpg

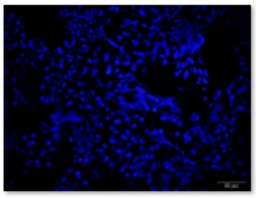
TAC 3-1.jpg

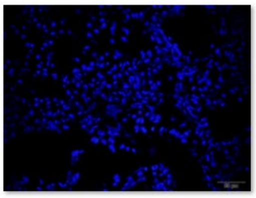
TAC 3-2.jpg

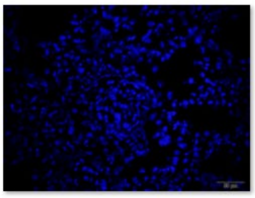
TAC 3-3.jpg

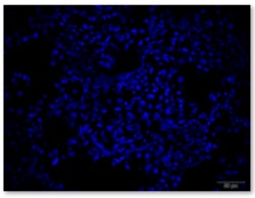
TAC 3-4.jpg

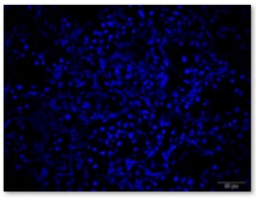
TAC 4-1.jpg

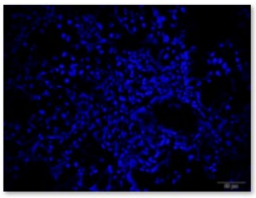
TAC 4-2.jpg

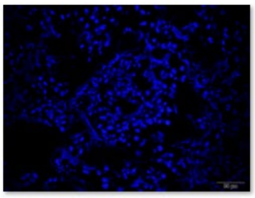
TAC 4-3.jpg

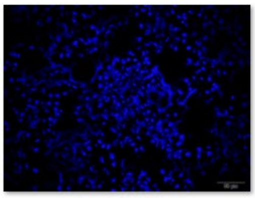
TAC 4-4.jpg

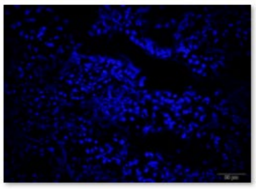

TAC 5-1.jpg

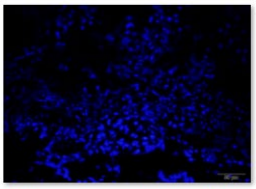

TAC 5-2 image in Fig. 2A.jpg

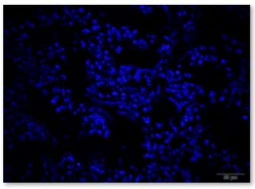

TAC 5-3.jpg

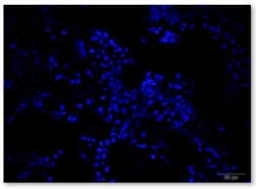

TAC 5-4.jpg

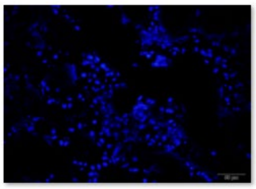

TAC 6-1.jpg

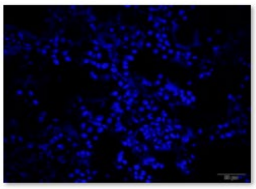

TAC 6-2.jpg

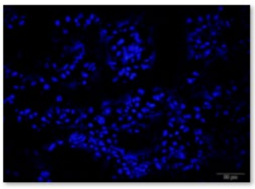

TAC 6-3.jpg

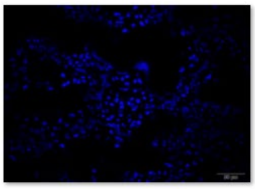

TAC 6-4.jpg

Original data for Fig. 3B-C5b-9 fluorescence intensity

CON	Fluorescence intensity	PHN	Fluorescence intensity	SQ-L	Fluorescence intensity	SQ-H	Fluorescence intensity	TAC	Fluorescence intensity
C5b-9-CON1-1	65.254	C5b-9-PHN1-1	190.668	C5b-9-SQ-L1-1	123.676	C5b-9-SQ-H1-1	98.364	C5b-9-TAC1-1	154.885
C5b-9-CON1-2	25.085	C5b-9-PHN1-2	197.156	C5b-9-SQ-L1-2	147.334	C5b-9-SQ-H1-2	93.512	C5b-9-TAC1-2	143.041
C5b-9-CON1-3	29.591	C5b-9-PHN1-3	163.813	C5b-9-SQ-L1-3	140.340	C5b-9-SQ-H1-3	85.331	C5b-9-TAC1-3	163.43
C5b-9-CON1-4	31.457	C5b-9-PHN1-4	175.847	C5b-9-SQ-L1-4	127.146	C5b-9-SQ-H1-4	97.779	C5b-9-TAC1-4	148.409
C5b-9-CON1-5	40.348	C5b-9-PHN1-5	177.524	C5b-9-SQ-L1-5	109.875	C5b-9-SQ-H1-5	110.126	C5b-9-TAC1-5	160.185
C5b-9-CON2-1	43.388	C5b-9-PHN2-1	183.712	C5b-9-SQ-L2-1	142.328	C5b-9-SQ-H2-1	124.447	C5b-9-TAC2-1	156.73
C5b-9-CON2-2	103.853	C5b-9-PHN2-2	185.743	C5b-9-SQ-L2-2	128.991	C5b-9-SQ-H2-2	89.307	C5b-9-TAC2-2	135.996
C5b-9-CON2-3	31.707	C5b-9-PHN2-3	181.899	C5b-9-SQ-L2-3	134.200	C5b-9-SQ-H2-3	69.187	C5b-9-TAC2-3	158.528
C5b-9-CON2-4	16.486	C5b-9-PHN2-4	190.355	C5b-9-SQ-L2-4	127.357	C5b-9-SQ-H2-4	107.939	C5b-9-TAC2-4	164.62
C5b-9-CON2-5	15.939	C5b-9-PHN2-5	192.806	C5b-9-SQ-L2-5	135.247	C5b-9-SQ-H2-5	107.087	C5b-9-TAC2-5	152.913
C5b-9-CON3-1	21.256	C5b-9-PHN3-1	177.155	C5b-9-SQ-L3-1	139.676	C5b-9-SQ-H3-1	140.138	C5b-9-TAC3-1	160.836
C5b-9-CON3-2	22.061	C5b-9-PHN3-2	174.761	C5b-9-SQ-L3-2	140.356	C5b-9-SQ-H3-2	136.343	C5b-9-TAC3-2	139.636
C5b-9-CON3-3	19.367	C5b-9-PHN3-3	194.123	C5b-9-SQ-L3-3	133.079	C5b-9-SQ-H3-3	137.048	C5b-9-TAC3-3	157.363
C5b-9-CON3-4	48.848	C5b-9-PHN3-4	189.07	C5b-9-SQ-L3-4	114.329	C5b-9-SQ-H3-4	147.63	C5b-9-TAC3-4	157.207
C5b-9-CON3-5	31.929	C5b-9-PHN3-5	177.865	C5b-9-SQ-L3-5	144.844	C5b-9-SQ-H3-5	148.141	C5b-9-TAC3-5	147.4
C5b-9-CON4-1	32.373	C5b-9-PHN4-1	175.266	C5b-9-SQ-L4-1	119.726	C5b-9-SQ-H4-1	113.537	C5b-9-TAC4-1	141.8
C5b-9-CON4-2	77.202	C5b-9-PHN4-2	182.164	C5b-9-SQ-L4-2	127.794	C5b-9-SQ-H4-2	115.918	C5b-9-TAC4-2	139.667
C5b-9-CON4-3	108.386	C5b-9-PHN4-3	195.094	C5b-9-SQ-L4-3	125.893	C5b-9-SQ-H4-3	85.898	C5b-9-TAC4-3	141.615
C5b-9-CON4-4	23.429	C5b-9-PHN4-4	190.037	C5b-9-SQ-L4-4	136.444	C5b-9-SQ-H4-4	67.359	C5b-9-TAC4-4	149.157
C5b-9-CON4-5	66.209	C5b-9-PHN4-5	186.414	C5b-9-SQ-L4-5	115.815	C5b-9-SQ-H4-5	101.601	C5b-9-TAC4-5	146.4
C5b-9-CON5-1	17.304	C5b-9-PHN5-1	194.267	C5b-9-SQ-L5-1	133.746	C5b-9-SQ-H5-1	80.465	C5b-9-TAC6-1	154.517
C5b-9-CON5-2	20.077	C5b-9-PHN5-2	183.003	C5b-9-SQ-L5-2	144.198	C5b-9-SQ-H5-2	97.345	C5b-9-TAC6-2	144.203
C5b-9-CON5-3	60.164	C5b-9-PHN5-3	183.82	C5b-9-SQ-L5-3	131.285	C5b-9-SQ-H5-3	81.605	C5b-9-TAC6-3	135.798
C5b-9-CON5-4	60.241	C5b-9-PHN5-4	206.279	C5b-9-SQ-L5-4	138.133	C5b-9-SQ-H5-4	97.325	C5b-9-TAC6-4	141.011
C5b-9-CON5-5	31.127	C5b-9-PHN5-5	195.703	C5b-9-SQ-L5-5	142.577	C5b-9-SQ-H5-5	98.926	C5b-9-TAC6-5	135.162
C5b-9-CON6-1	41.95	C5b-9-PHN6-1	190.807	C5b-9-SQ-L6-1	140.058	C5b-9-SQ-H6-1	100.485	C5b-9-TAC6-2	131.365
C5b-9-CON6-2	150.443	C5b-9-PHN6-2	191.578	C5b-9-SQ-L6-2	127.490	C5b-9-SQ-H6-2	100.781	C5b-9-TAC6-3	135.197
C5b-9-CON6-3	51.48	C5b-9-PHN6-3	185.675	C5b-9-SQ-L6-3	103.610	C5b-9-SQ-H6-3	92.077	C5b-9-TAC6-4	141.515
C5b-9-CON6-4	44.619	C5b-9-PHN6-4	186.731	C5b-9-SQ-L6-4	137.508	C5b-9-SQ-H6-4	102.808	C5b-9-TAC6-5	139.061
C5b-9-CON6-5	118.211	C5b-9-PHN6-5	183.791	C5b-9-SQ-L6-5	100.484	C5b-9-SQ-H6-5	96.835	C5b-9-TAC6-6	142.263

Original Image for Fig. 4A-Synaptopodin

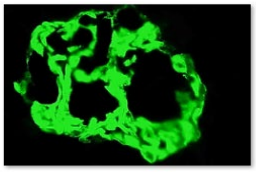

synaptopodin-con1-1.jpg

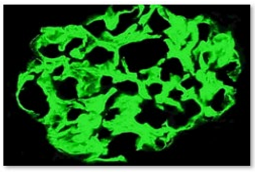

synaptopodin-con1-2 image in Fig. 3A.jpg

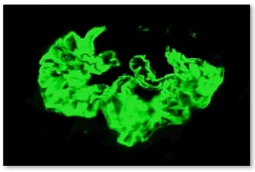

synaptopodin-con1-3.jpg

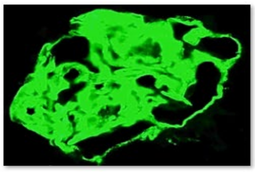

synaptopodin-con1-4.jpg

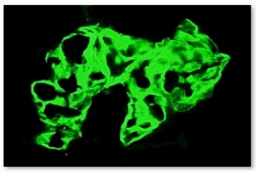

synaptopodin-con1-5.jpg

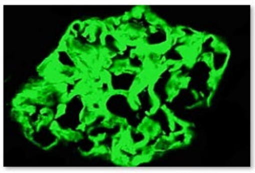

synaptopodin-con2-1.jpg

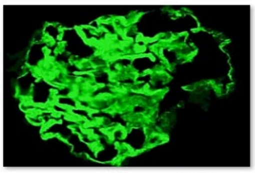

synaptopodin-con2-2.jpg

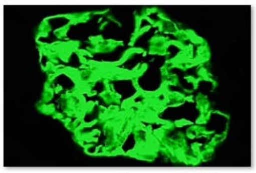

synaptopodin-con2-3.jpg

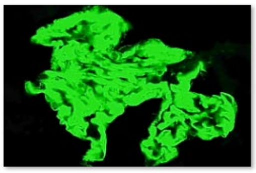

synaptopodin-con2-4.jpg

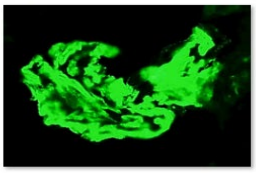

synaptopodin-con2-5.jpg

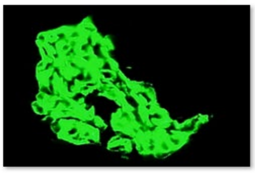

synaptopodin-con3-1.jpg

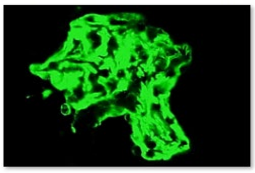

synaptopodin-con3-2.jpg

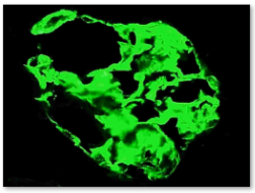
synaptopodin-con3-3.jpg

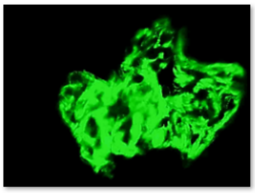
synaptopodin-con3-4.jpg

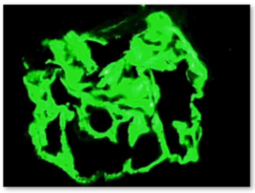
synaptopodin-con3-5.jpg

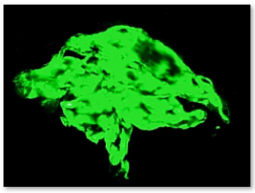
synaptopodin-con4-1.jpg

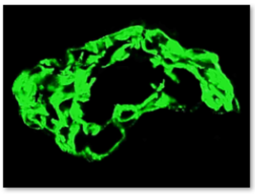
synaptopodin-con4-2.jpg

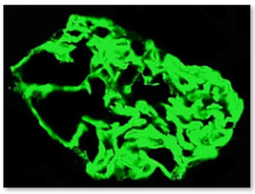
synaptopodin-con4-3.jpg

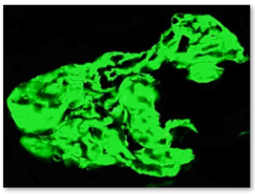
synaptopodin-con4-4.jpg

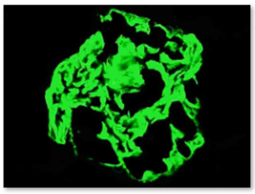
synaptopodin-con4-5.jpg

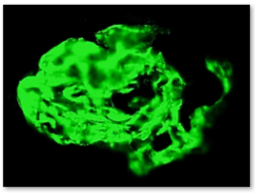
synaptopodin-con5-1.jpg

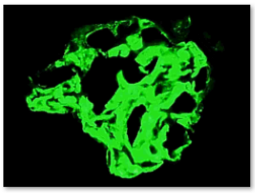
synaptopodin-con5-2.jpg

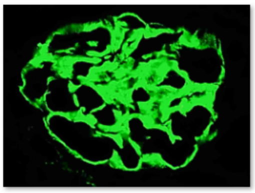
synaptopodin-con5-3.jpg

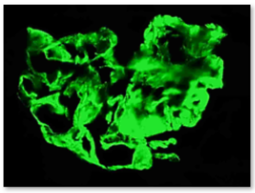
synaptopodin-con5-4.jpg

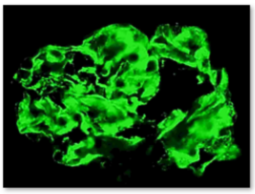
synaptopodin-con5-5.jpg

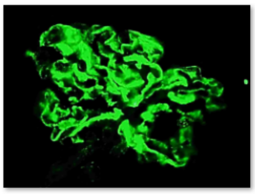
synaptopodin-con6-1.jpg

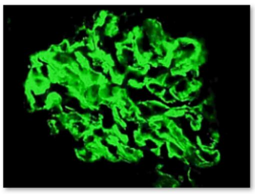
synaptopodin-con6-2.jpg

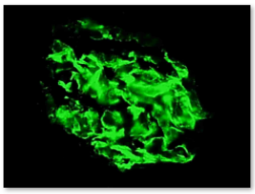
synaptopodin-con6-3.jpg

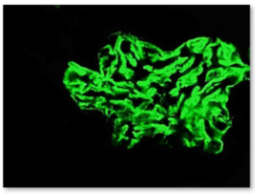
synaptopodin-con6-4.jpg

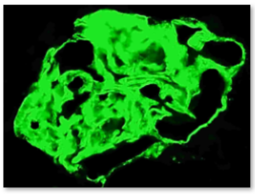
synaptopodin-con6-5.jpg

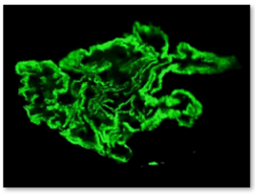
synaptopodin-PHN1-1.jpg

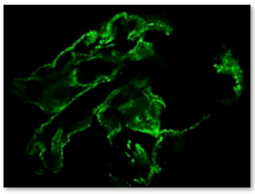
synaptopodin-PHN1-2.jpg

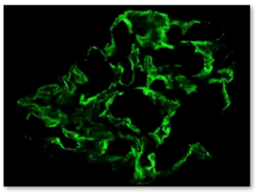
synaptopodin-PHN1-3.jpg

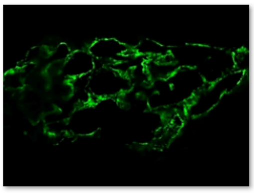
synaptopodin-PHN1-4.jpg

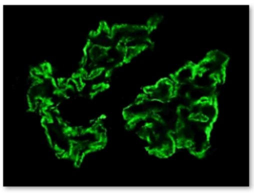
synaptopodin-PHN1-5.jpg

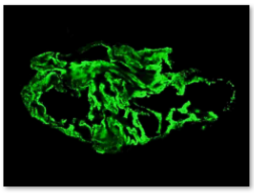
synaptopodin-PHN2-1.jpg

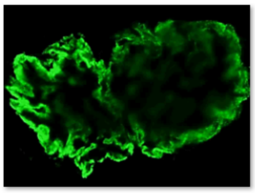
synaptopodin-PHN2-2.jpg

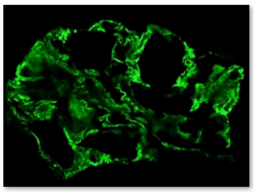
synaptopodin-PHN2-3.jpg

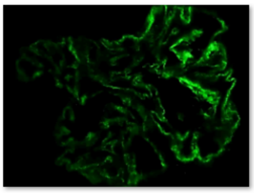
synaptopodin-PHN2-4.jpg

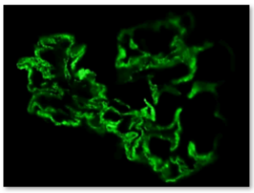
synaptopodin-PHN2-5.jpg

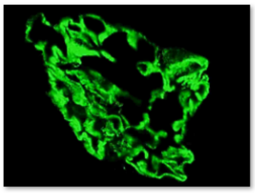
synaptopodin-PHN3-1.jpg

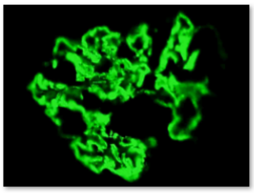
synaptopodin-PHN3-2.jpg

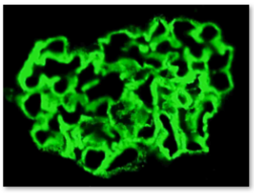
synaptopodin-PHN3-3.jpg

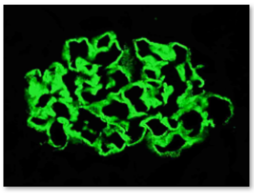
synaptopodin-PHN3-4.jpg

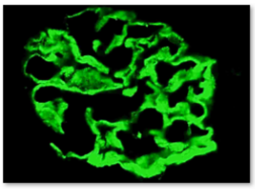
synaptopodin-PHN3-5.jpg

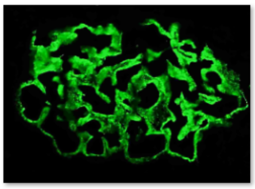
synaptopodin-PHN4-1.jpg

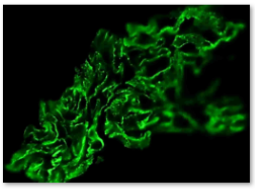
synaptopodin-PHN4-2.jpg

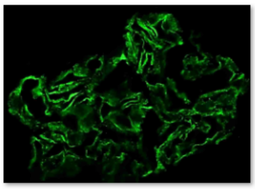
synaptopodin-PHN4-3.jpg

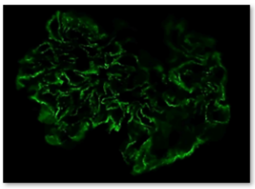
synaptopodin-PHN4-4.jpg

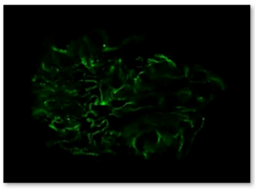
synaptopodin-PHN4-5.jpg

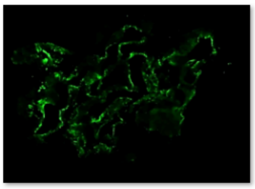
synaptopodin-PHN5-1.jpg

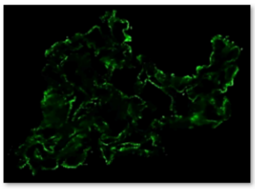
synaptopodin-PHN5-2.jpg

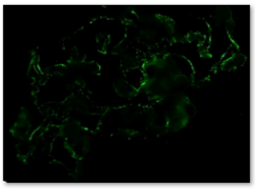
synaptopodin-PHN5-3.jpg

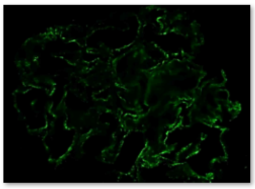
synaptopodin-PHN5-4.jpg

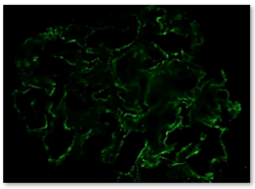
synaptopodin-PHN5-5.jpg

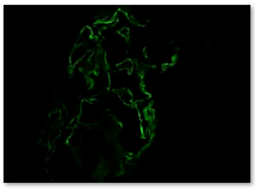
synaptopodin-PHN6-1.jpg

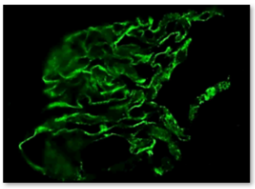
synaptopodin-PHN6-2.jpg

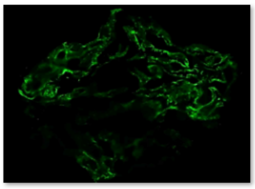
synaptopodin-PHN6-3.jpg

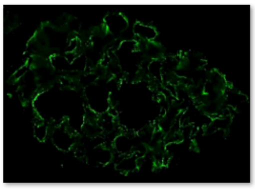
synaptopodin-PHN6-4 image in Fig. 3A.jpg

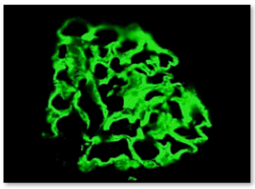
synaptopodin-PHN6-5.jpg

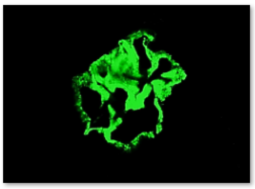
synaptopodin-SQ-H1-1.jpg

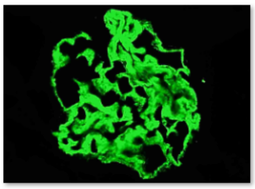
synaptopodin-SQ-H1-2.jpg

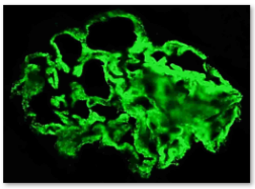
synaptopodin-SQ-H1-3.jpg

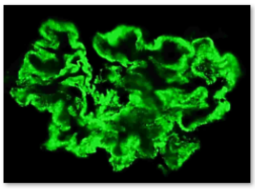
synaptopodin-SQ-H1-4.jpg

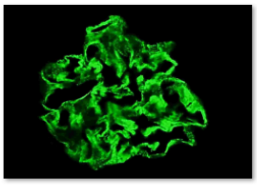
synaptopodin-SQ-H1-5.jpg

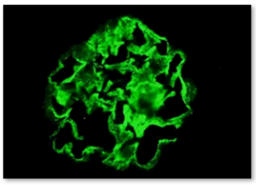
synaptopodin-SQ-H2-1.jpg

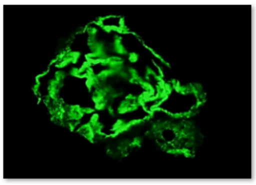
synaptopodin-SQ-H2-2.jpg

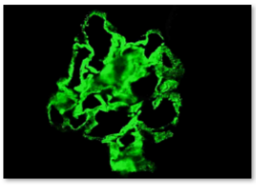
synaptopodin-SQ-H2-3.jpg

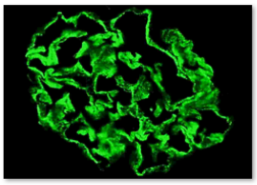
synaptopodin-SQ-H2-4.jpg

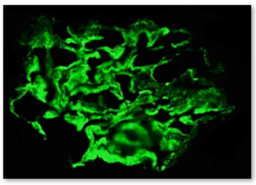
synaptopodin-SQ-H2-5.jpg

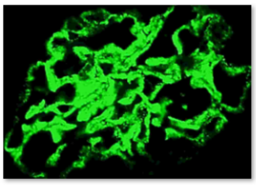
synaptopodin-SQ-H3-1.jpg

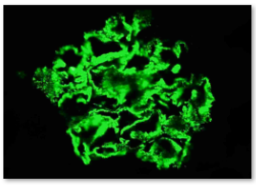
synaptopodin-SQ-H3-2.jpg

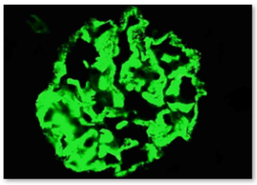
synaptopodin-SQ-H3-3.jpg

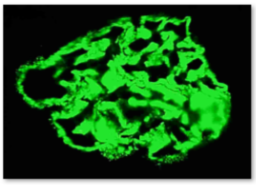
synaptopodin-SQ-H3-4.jpg

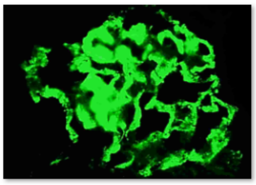
synaptopodin-SQ-H3-5.jpg

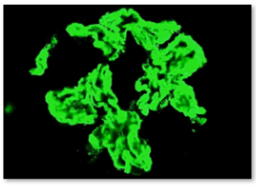
synaptopodin-SQ-H4-1.jpg

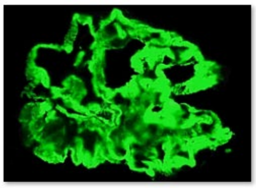
synaptopodin-SQ-H4-2.jpg

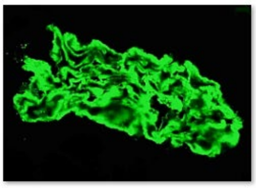
synaptopodin-SQ-H4-3.jpg

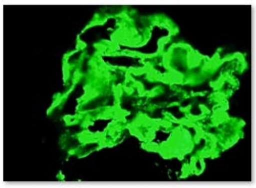
synaptopodin-SQ-H4-4.jpg

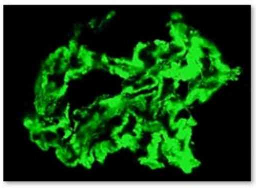
synaptopodin-SQ-H4-5.jpg

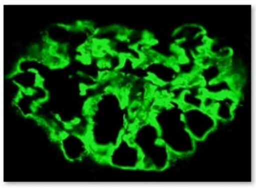
synaptopodin-SQ-H5-1.jpg

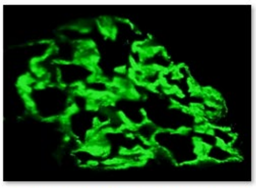
synaptopodin-SQ-H5-2.jpg

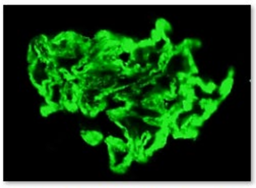
synaptopodin-SQ-H5-3.jpg

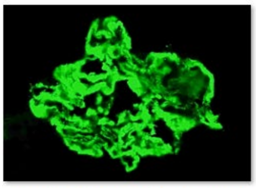
synaptopodin-SQ-H5-4.jpg

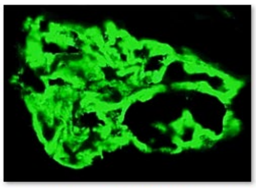
synaptopodin-SQ-H5-5.jpg

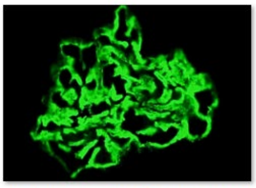
synaptopodin-SQ-H6-1.jpg

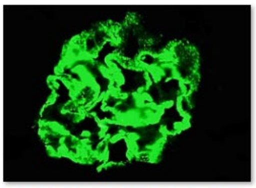
synaptopodin-SQ-H6-2.jpg

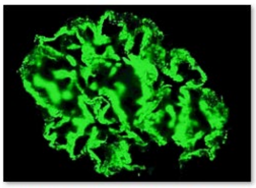
synaptopodin-SQ-H6-3.jpg

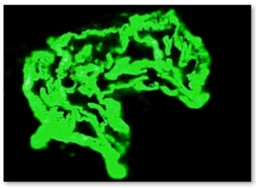
synaptopodin-SQ-H6-4.jpg

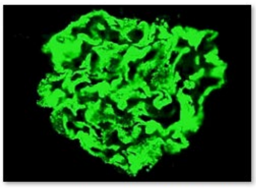
synaptopodin-SQ-H6-5 image in Fig. 3A.jpg

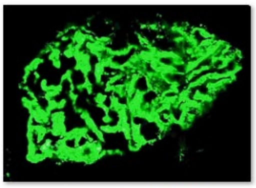
synaptopodin-TAC1-1.jpg

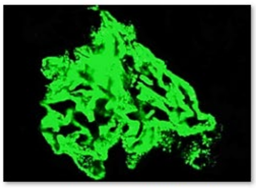
synaptopodin-TAC1-2.jpg

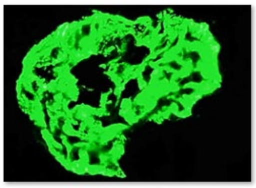
synaptopodin-TAC1-3.jpg

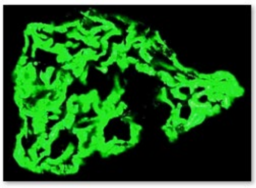
synaptopodin-TAC1-4.jpg

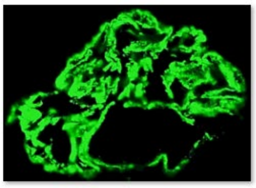
synaptopodin-TAC1-5.jpg

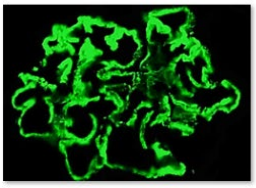
synaptopodin-TAC2-1.jpg

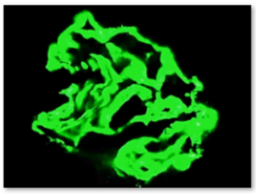
synaptopodin-TAC2-2.jpg

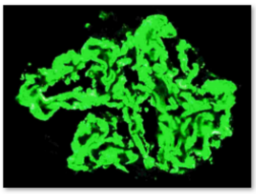
synaptopodin-TAC2-3.jpg

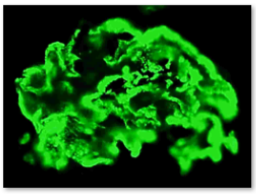
synaptopodin-TAC2-4.jpg

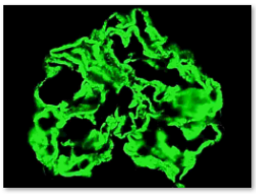
synaptopodin-TAC2-5.jpg

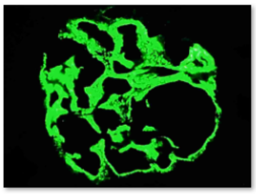
synaptopodin-TAC3-1.jpg

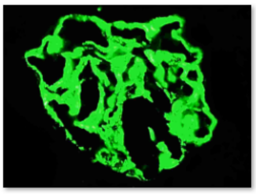
synaptopodin-TAC3-2.jpg

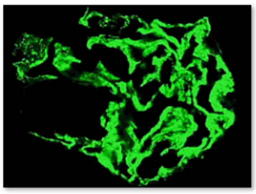
synaptopodin-TAC3-3.jpg

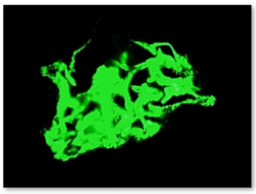
synaptopodin-TAC3-4.jpg

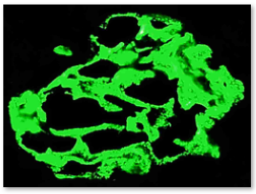
synaptopodin-TAC3-5.jpg

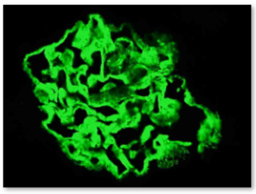
synaptopodin-TAC4-1.jpg

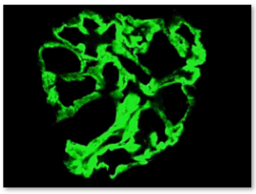
synaptopodin-TAC4-2.jpg

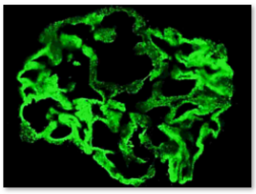
synaptopodin-TAC4-3.jpg

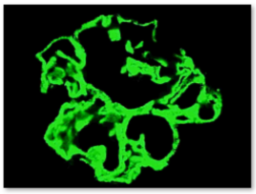
synaptopodin-TAC4-4.jpg

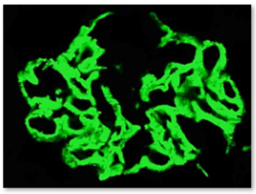
synaptopodin-TAC4-5.jpg

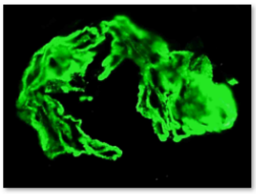
synaptopodin-TAC5-1.jpg

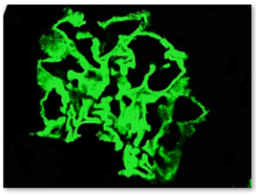
synaptopodin-TAC5-2.jpg

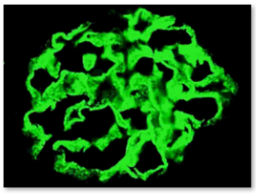
synaptopodin-TAC5-3 image in Fig. 3A.jpg

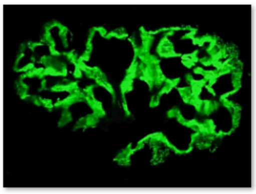
synaptopodin-TAC5-4.jpg

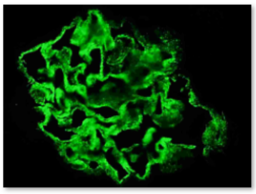
synaptopodin-TAC5-5.jpg

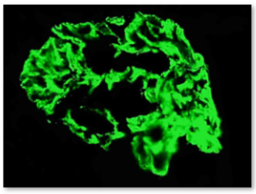
synaptopodin-TAC6-1.jpg

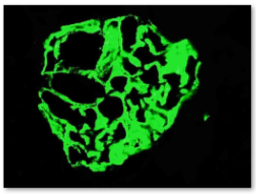
synaptopodin-TAC6-2.jpg

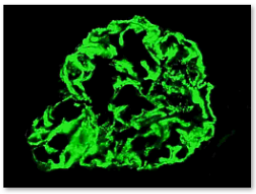
synaptopodin-TAC6-3.jpg

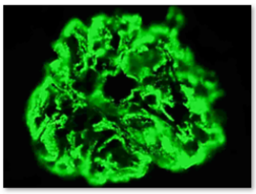
synaptopodin-TAC6-4.jpg

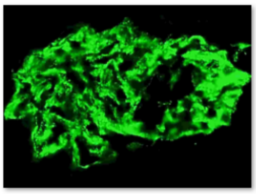
synaptopodin-TAC6-5.jpg

Original Image for Fig. 4A-Vimentin

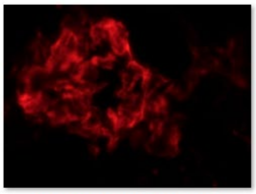
Vimentin-CON1-1.jpg

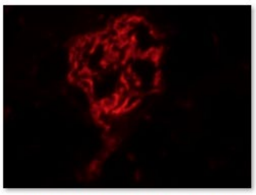
Vimentin-CON1-2.jpg

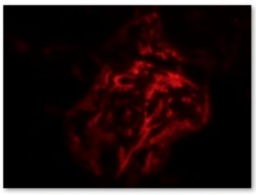
Vimentin-CON1-3.jpg

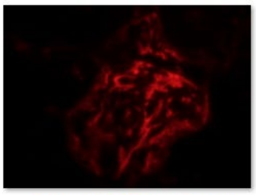
Vimentin-CON1-4.jpg

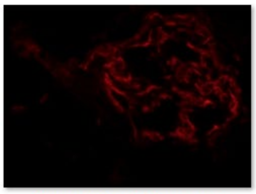
Vimentin-CON1-5.jpg

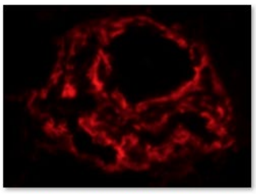
Vimentin-CON2-1.jpg

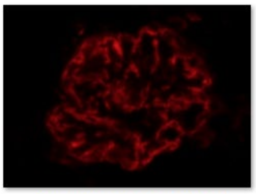
Vimentin-CON2-2.jpg

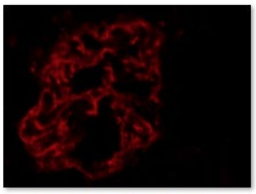
Vimentin-CON2-3.jpg

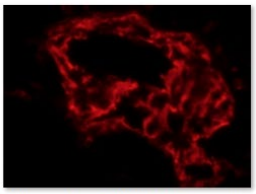
Vimentin-CON2-4 image in Fig. 3A.jpg

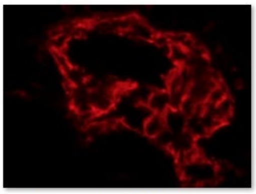
Vimentin-CON2-5.jpg

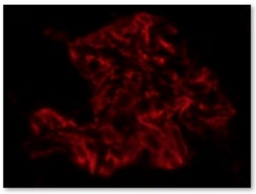
Vimentin-CON3-1.jpg

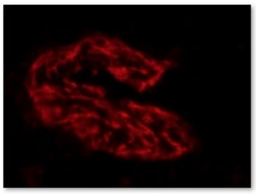
Vimentin-CON3-2.jpg

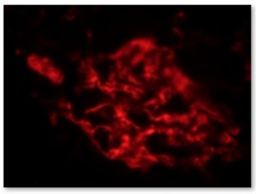
Vimentin-CON3-3.jpg

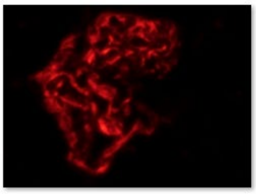
Vimentin-CON3-4.jpg

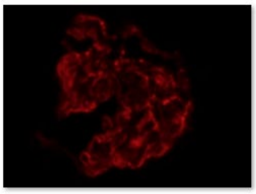
Vimentin-CON3-5.jpg

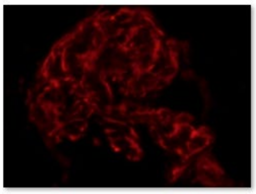
Vimentin-CON4-1.jpg

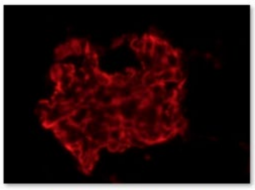
Vimentin-CON4-2.jpg

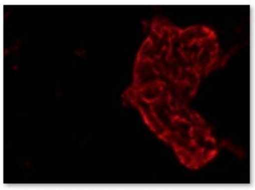
Vimentin-CON4-3.jpg

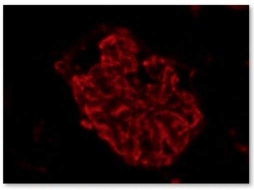
Vimentin-CON4-4.jpg

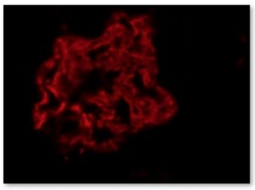
Vimentin-CON4-5.jpg

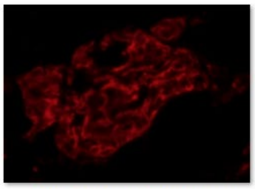
Vimentin-CON5-1.jpg

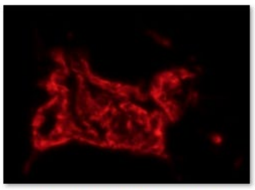
Vimentin-CON5-2.jpg

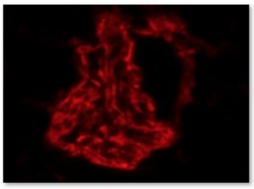
Vimentin-CON5-3.jpg

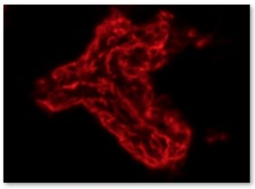
Vimentin-CON5-4.jpg

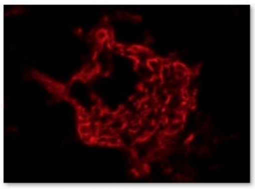
Vimentin-CON5-5.jpg

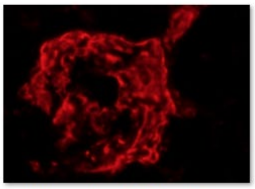
Vimentin-CON6-1.jpg

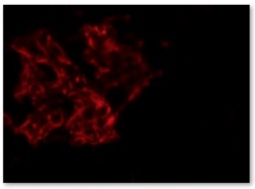
Vimentin-CON6-2.jpg

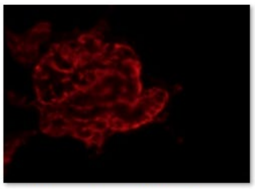
Vimentin-CON6-3.jpg

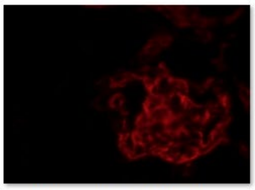
Vimentin-CON6-4.jpg

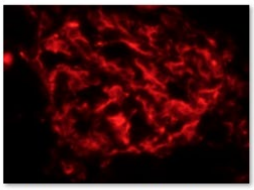
Vimentin-CON6-5.jpg

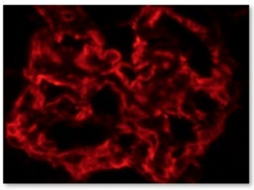
Vimentin-PHN1-1.jpg

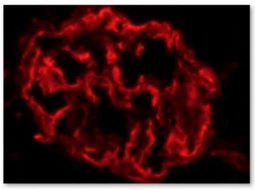
Vimentin-PHN1-2 image in Fig. 3A.jpg

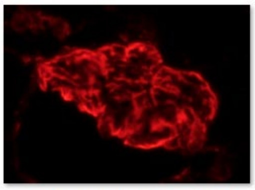
Vimentin-PHN1-3.jpg

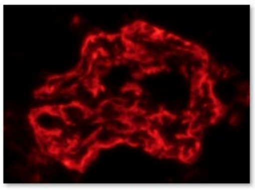
Vimentin-PHN1-4.jpg

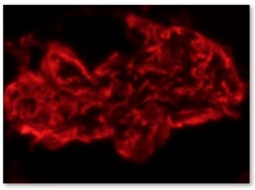
Vimentin-PHN1-5.jpg

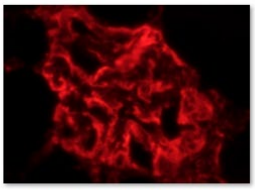
Vimentin-PHN2-1.jpg

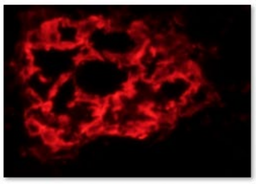
Vimentin-PHN2-2.jpg

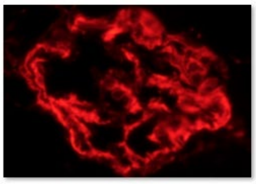
Vimentin-PHN2-3.jpg

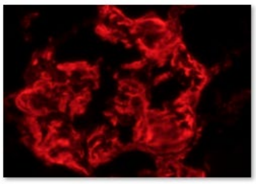
Vimentin-PHN2-4.jpg

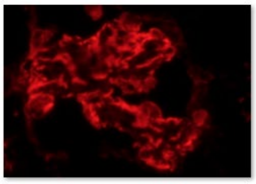
Vimentin-PHN2-5.jpg

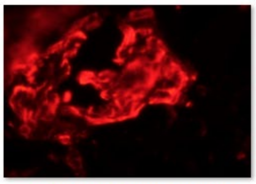
Vimentin-PHN3-1.jpg

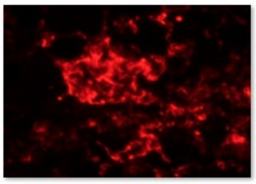
Vimentin-PHN3-2.jpg

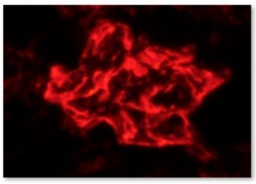
Vimentin-PHN3-3.jpg

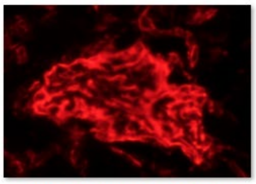
Vimentin-PHN3-4.jpg

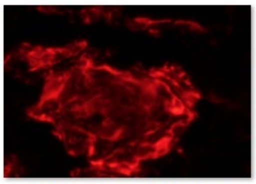
Vimentin-PHN3-5.jpg

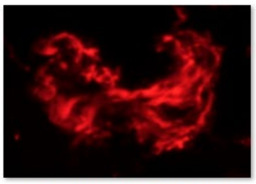
Vimentin-PHN4-1.jpg

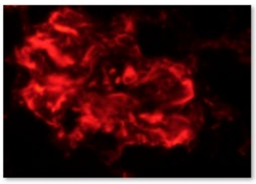
Vimentin-PHN4-2.jpg

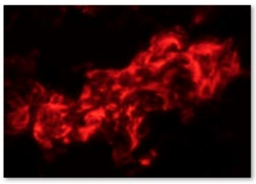
Vimentin-PHN4-3.jpg

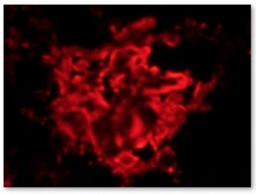
Vimentin-PHN4-4.jpg
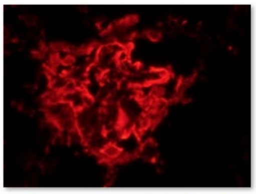
Vimentin-PHN4-5.jpg
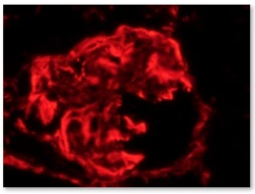
Vimentin-PHN5-1.jpg
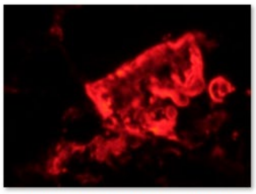
Vimentin-PHN5-2.jpg
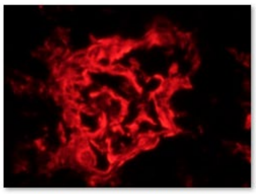
Vimentin-PHN5-3.jpg
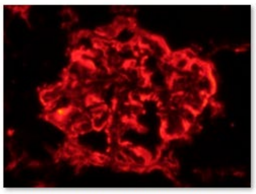
Vimentin-PHN5-4.jpg
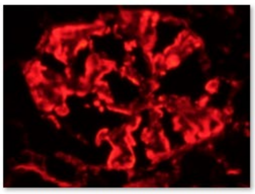
Vimentin-PHN5-5.jpg
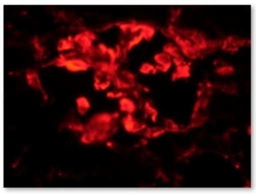
Vimentin-PHN6-1.jpg
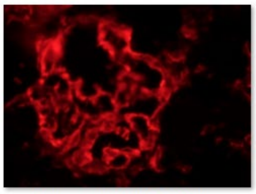
Vimentin-PHN6-2.jpg
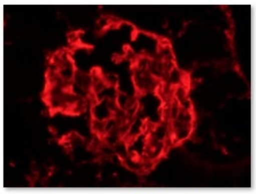
Vimentin-PHN6-3.jpg
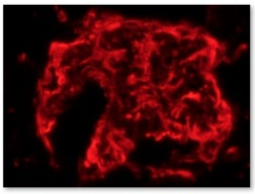
Vimentin-PHN6-4.jpg
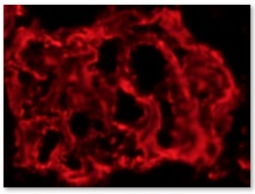
Vimentin-PHN6-5.jpg
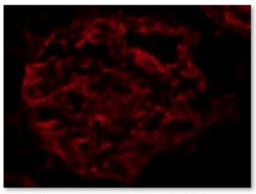
Vimentin-SQ-H1-1.jpg
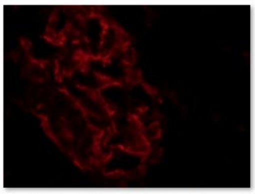
Vimentin-SQ-H1-2.jpg
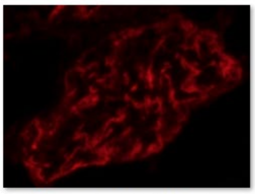
Vimentin-SQ-H1-3.jpg
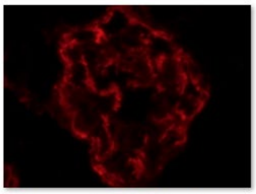
Vimentin-SQ-H1-4.jpg
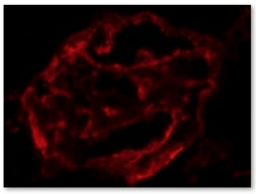
Vimentin-SQ-H1-5.jpg
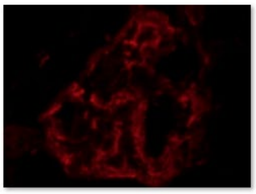
Vimentin-SQ-H2-1.jpg
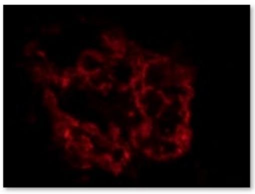
Vimentin-SQ-H2-2.jpg
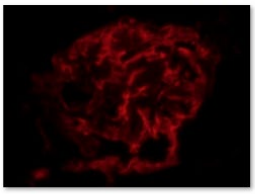
Vimentin-SQ-H2-3.jpg
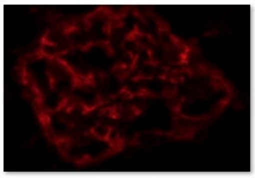
Vimentin-SQ-H2-4.jpg
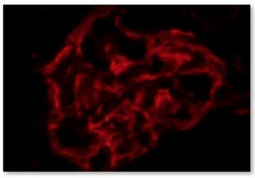
Vimentin-SQ-H2-5.jpg
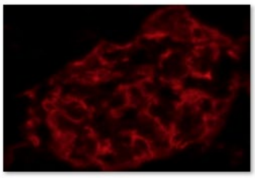
Vimentin-SQ-H3-1.jpg
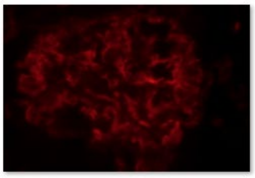
Vimentin-SQ-H3-2 image in Fig. 3A.jpg
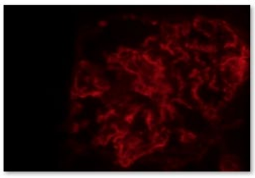
Vimentin-SQ-H3-3.jpg
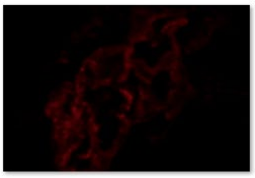
Vimentin-SQ-H3-4.jpg
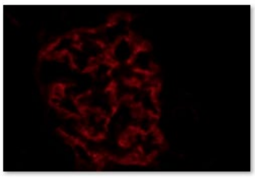
Vimentin-SQ-H3-5.jpg
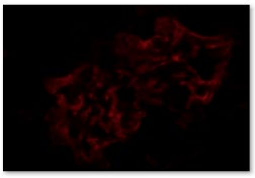
Vimentin-SQ-H4-1.jpg
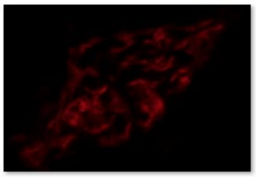
Vimentin-SQ-H4-2.jpg
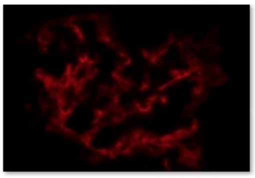
Vimentin-SQ-H4-3.jpg
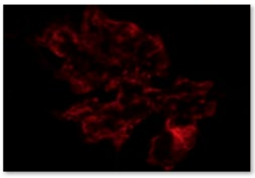
Vimentin-SQ-H4-4.jpg
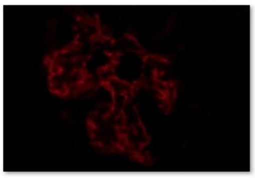
Vimentin-SQ-H4-5.jpg

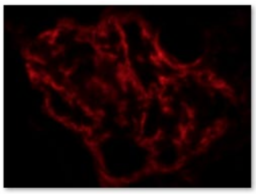
Vimentin-SQ-H5-1.jpg

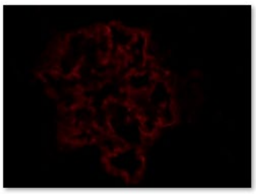
Vimentin-SQ-H5-2.jpg

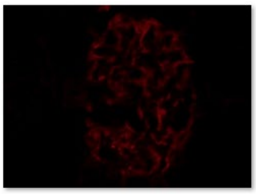
Vimentin-SQ-H5-3.jpg

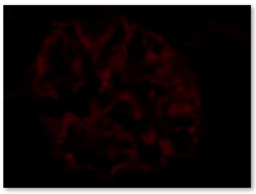
Vimentin-SQ-H5-4.jpg

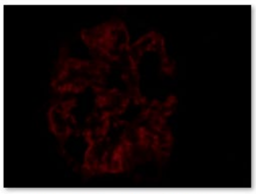
Vimentin-SQ-H5-5.jpg

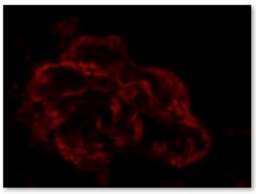
Vimentin-SQ-H6-1.jpg

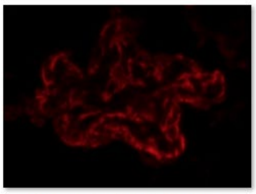
Vimentin-SQ-H6-2.jpg

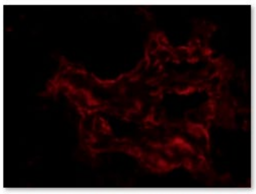
Vimentin-SQ-H6-3.jpg

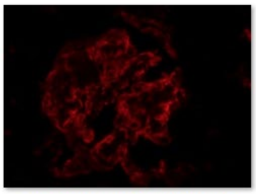
Vimentin-SQ-H6-4.jpg

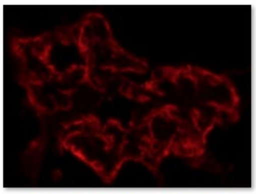
Vimentin-SQ-H6-5.jpg

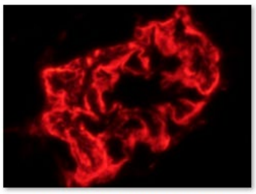
Vimentin-TAC1-1.jpg

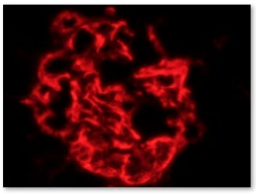
Vimentin-TAC1-2.jpg

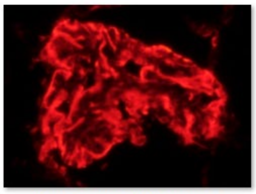
Vimentin-TAC1-3.jpg

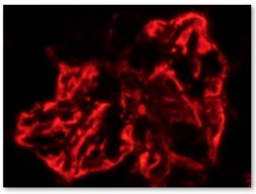
Vimentin-TAC1-4.jpg

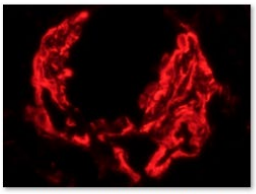
Vimentin-TAC1-5.jpg

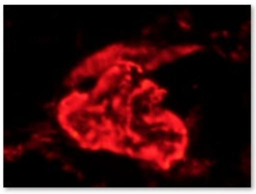
Vimentin-TAC2-1.jpg

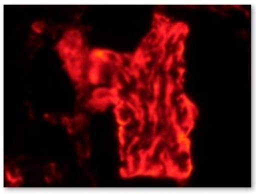
Vimentin-TAC2-2.jpg

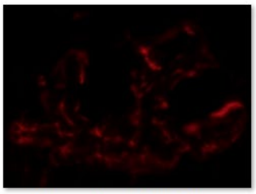
Vimentin-TAC2-3.jpg

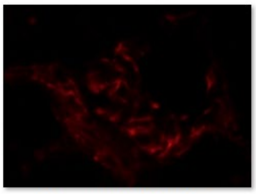
Vimentin-TAC2-4.jpg

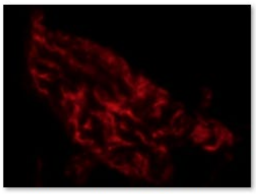
Vimentin-TAC2-5.jpg

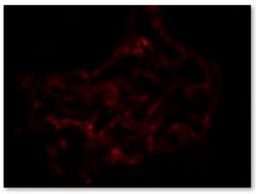
Vimentin-TAC3-1.jpg

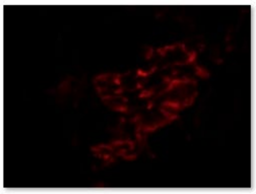
Vimentin-TAC3-2.jpg

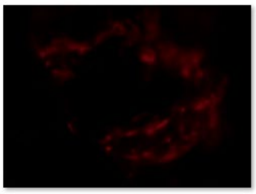
Vimentin-TAC3-3.jpg

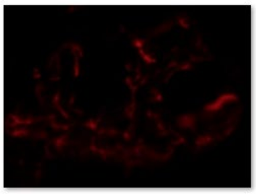
Vimentin-TAC3-4.jpg

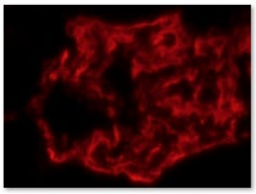
Vimentin-TAC3-5.jpg

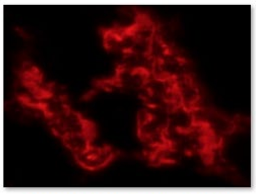
Vimentin-TAC4-1.jpg

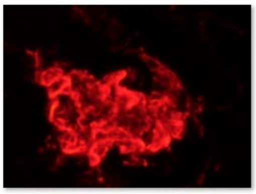
Vimentin-TAC4-2.jpg

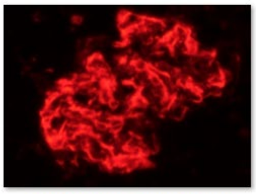
Vimentin-TAC4-3.jpg

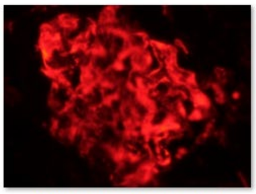
Vimentin-TAC4-4.jpg

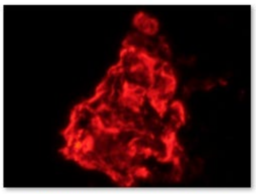
Vimentin-TAC4-5.jpg

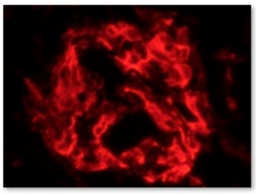
Vimentin-TAC5-1.jpg

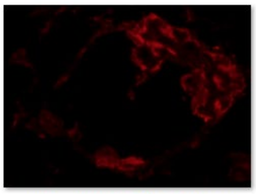
Vimentin-TAC5-2.jpg

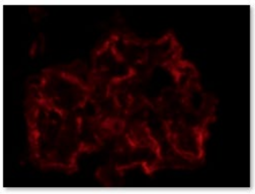

Vimentin-TAC5-3.jpg

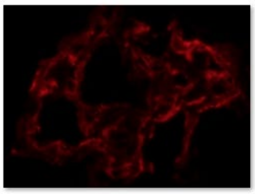

Vimentin-TAC5-4.jpg

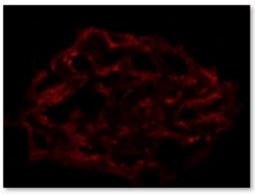

Vimentin-TAC5-5.jpg

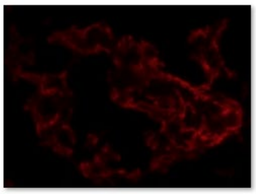

Vimentin-TAC6-1.jpg

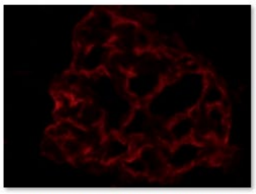

Vimentin-TAC6-2 image in [illegible]

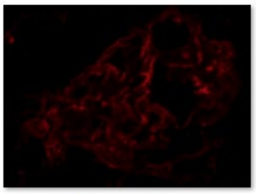

Vimentin-TAC6-3.jpg

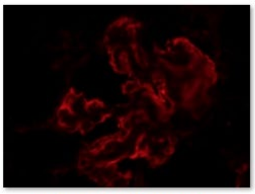

Vimentin-TAC6-4.jpg

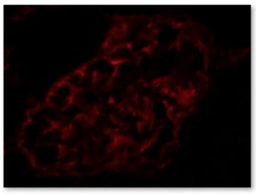

Vimentin-TAC6-5.jpg

Original data for Fig. 4B-C

Synaptopodin

CON	Fluorescence intensity		PHN	Fluorescence intensity		SQ-H	Fluorescence intensity		TAC	Fluorescence intensity
synaptopodin-con1-1	204.028		synaptopodin-PHN1-1	172.349		synaptopodin-SQ-H1-1	201.269		synaptopodin-TAC1-1	210.07
synaptopodin-con1-2	212.872		synaptopodin-PHN1-2	118.524		synaptopodin-SQ-H1-2	199.581		synaptopodin-TAC1-2	212.492
synaptopodin-con1-3	205.146		synaptopodin-PHN1-3	141.213		synaptopodin-SQ-H1-3	179.331		synaptopodin-TAC1-3	217.458
synaptopodin-con1-4	214.942		synaptopodin-PHN1-4	121.045		synaptopodin-SQ-H1-4	183.951		synaptopodin-TAC1-4	218.109
synaptopodin-con1-5	201.018		synaptopodin-PHN1-5	147.909		synaptopodin-SQ-H1-5	169.089		synaptopodin-TAC1-5	196.943
synaptopodin-con2-1	205.547		synaptopodin-PHN2-1	173.57		synaptopodin-SQ-H2-1	170.247		synaptopodin-TAC2-1	194.748
synaptopodin-con2-2	192.615		synaptopodin-PHN2-2	160.204		synaptopodin-SQ-H2-2	174.318		synaptopodin-TAC2-2	218.618
synaptopodin-con2-3	205.425		synaptopodin-PHN2-3	136.092		synaptopodin-SQ-H2-3	186.741		synaptopodin-TAC2-3	219.193
synaptopodin-con2-4	211.071		synaptopodin-PHN2-4	112.742		synaptopodin-SQ-H2-4	174.614		synaptopodin-TAC2-4	196.732
synaptopodin-con2-5	207.324		synaptopodin-PHN2-5	125.662		synaptopodin-SQ-H2-5	171.26		synaptopodin-TAC2-5	206.162
synaptopodin-con3-1	215.451		synaptopodin-PHN3-1	181.933		synaptopodin-SQ-H3-1	201.758		synaptopodin-TAC3-1	216.937
synaptopodin-con3-2	204.257		synaptopodin-PHN3-2	192.001		synaptopodin-SQ-H3-2	194.273		synaptopodin-TAC3-2	217.613
synaptopodin-con3-3	210.596		synaptopodin-PHN3-3	192.443		synaptopodin-SQ-H3-3	211.409		synaptopodin-TAC3-3	201.466
synaptopodin-con3-4	201.714		synaptopodin-PHN3-4	196.69		synaptopodin-SQ-H3-4	208.717		synaptopodin-TAC3-4	217.542
synaptopodin-con3-5	218.695		synaptopodin-PHN3-5	193.386		synaptopodin-SQ-H3-5	206.988		synaptopodin-TAC3-5	217.451
synaptopodin-con4-1	215.166		synaptopodin-PHN4-1	174.788		synaptopodin-SQ-H4-1	210.337		synaptopodin-TAC4-1	195.91
synaptopodin-con4-2	210.75		synaptopodin-PHN4-2	119.667		synaptopodin-SQ-H4-2	195.075		synaptopodin-TAC4-2	201.934
synaptopodin-con4-3	211.709		synaptopodin-PHN4-3	117.714		synaptopodin-SQ-H4-3	197.821		synaptopodin-TAC4-3	183.671
synaptopodin-con4-4	210.977		synaptopodin-PHN4-4	81.063		synaptopodin-SQ-H4-4	203.714		synaptopodin-TAC4-4	211.342
synaptopodin-con4-5	213.538		synaptopodin-PHN4-5	74.022		synaptopodin-SQ-H4-5	186.699		synaptopodin-TAC4-5	209.994
synaptopodin-con5-1	199.263		synaptopodin-PHN5-1	107.464		synaptopodin-SQ-H5-1	190.371		synaptopodin-TAC5-1	192.116
synaptopodin-con5-2	214.624		synaptopodin-PHN5-2	104.099		synaptopodin-SQ-H5-2	186.782		synaptopodin-TAC5-2	208.259
synaptopodin-con5-3	210.563		synaptopodin-PHN5-3	82.51		synaptopodin-SQ-H5-3	189.035		synaptopodin-TAC5-3	205.544
synaptopodin-con5-4	195.665		synaptopodin-PHN5-4	83.431		synaptopodin-SQ-H5-4	195.412		synaptopodin-TAC5-4	182.864
synaptopodin-con5-5	191.843		synaptopodin-PHN5-5	83.431		synaptopodin-SQ-H5-5	202.8		synaptopodin-TAC5-5	165.914
synaptopodin-con6-1	196.631		synaptopodin-PHN6-1	87.615		synaptopodin-SQ-H6-1	194.398		synaptopodin-TAC6-1	199.735
synaptopodin-con6-2	195.17		synaptopodin-PHN6-2	119.055		synaptopodin-SQ-H6-2	207.444		synaptopodin-TAC6-2	217.218
synaptopodin-con6-3	185.023		synaptopodin-PHN6-3	96.819		synaptopodin-SQ-H6-3	201.648		synaptopodin-TAC6-3	199.427
synaptopodin-con6-4	195.227		synaptopodin-PHN6-4	206.925		synaptopodin-SQ-H6-4	216.186		synaptopodin-TAC6-4	190.423
synaptopodin-con6-5	207.989		synaptopodin-PHN6-5	196.446		synaptopodin-SQ-H6-5	208.168		synaptopodin-TAC6-5	184.689

Vimentin

CON	Fluorescence intensity	PHN	Fluorescence intensity	SQ-H	Fluorescence intensity	TAC	Fluorescence intensity
Vimentin-CON1-1	43.747	Vimentin-PHN1-1	55.553	Vimentin-SQ-H1-1	11.564	Vimentin-TAC1-1	68.417
Vimentin-CON1-2	44.311	Vimentin-PHN1-2	55.875	Vimentin-SQ-H1-2	17.312	Vimentin-TAC1-2	64.334
Vimentin-CON1-3	28.435	Vimentin-PHN1-3	69.271	Vimentin-SQ-H1-3	16.1	Vimentin-TAC1-3	70.267
Vimentin-CON1-4	29.489	Vimentin-PHN1-4	72.116	Vimentin-SQ-H1-4	24.813	Vimentin-TAC1-4	65.742
Vimentin-CON1-5	36.999	Vimentin-PHN1-5	65.924	Vimentin-SQ-H1-5	17.792	Vimentin-TAC1-5	69.171
Vimentin-CON2-1	32.781	Vimentin-PHN2-1	66.106	Vimentin-SQ-H2-1	22.621	Vimentin-TAC2-1	73.038
Vimentin-CON2-2	33.057	Vimentin-PHN2-2	74.982	Vimentin-SQ-H2-2	28.881	Vimentin-TAC2-2	75.409
Vimentin-CON2-3	34.41	Vimentin-PHN2-3	71.013	Vimentin-SQ-H2-3	25.877	Vimentin-TAC2-3	17.973
Vimentin-CON2-4	28.438	Vimentin-PHN2-4	51.985	Vimentin-SQ-H2-4	22.936	Vimentin-TAC2-4	17.503
Vimentin-CON2-5	42.79	Vimentin-PHN2-5	61.46	Vimentin-SQ-H2-5	27.537	Vimentin-TAC2-5	28.961
Vimentin-CON3-1	38.81	Vimentin-PHN3-1	57.134	Vimentin-SQ-H3-1	16.057	Vimentin-TAC3-1	14.3
Vimentin-CON3-2	43.572	Vimentin-PHN3-2	49.56	Vimentin-SQ-H3-2	18.157	Vimentin-TAC3-2	22.795
Vimentin-CON3-3	37.113	Vimentin-PHN3-3	45.68	Vimentin-SQ-H3-3	24.055	Vimentin-TAC3-3	15.202
Vimentin-CON3-4	44.728	Vimentin-PHN3-4	50.521	Vimentin-SQ-H3-4	25.991	Vimentin-TAC3-4	17.325
Vimentin-CON3-5	29.464	Vimentin-PHN3-5	53.098	Vimentin-SQ-H3-5	23.886	Vimentin-TAC3-5	38.3
Vimentin-CON4-1	29.773	Vimentin-PHN4-1	53.695	Vimentin-SQ-H4-1	26.54	Vimentin-TAC4-1	43.33
Vimentin-CON4-2	27.642	Vimentin-PHN4-2	45.28	Vimentin-SQ-H4-2	29.165	Vimentin-TAC4-2	65.93
Vimentin-CON4-3	42.424	Vimentin-PHN4-3	53.758	Vimentin-SQ-H4-3	24.192	Vimentin-TAC4-3	64.974
Vimentin-CON4-4	38.098	Vimentin-PHN4-4	60.022	Vimentin-SQ-H4-4	25.59	Vimentin-TAC4-4	69.782
Vimentin-CON4-5	34.433	Vimentin-PHN4-5	63.167	Vimentin-SQ-H4-5	30.082	Vimentin-TAC4-5	73.121
Vimentin-CON5-1	34.433	Vimentin-PHN5-1	55.16	Vimentin-SQ-H5-1	31.448	Vimentin-TAC5-1	69.669
Vimentin-CON5-2	23.628	Vimentin-PHN5-2	49.412	Vimentin-SQ-H5-2	32.279	Vimentin-TAC5-2	28.372
Vimentin-CON5-3	38.9	Vimentin-PHN5-3	65.308	Vimentin-SQ-H5-3	26.732	Vimentin-TAC5-3	26.158
Vimentin-CON5-4	28.583	Vimentin-PHN5-4	60.255	Vimentin-SQ-H5-4	27.159	Vimentin-TAC5-4	25.354
Vimentin-CON5-5	28.584	Vimentin-PHN5-5	67.662	Vimentin-SQ-H5-5	31.57	Vimentin-TAC5-5	21.971
Vimentin-CON6-1	40.127	Vimentin-PHN6-1	67.3	Vimentin-SQ-H6-1	26.757	Vimentin-TAC6-1	21.034
Vimentin-CON6-2	40.127	Vimentin-PHN6-2	47.987	Vimentin-SQ-H6-2	35.091	Vimentin-TAC6-2	22.255
Vimentin-CON6-3	27.997	Vimentin-PHN6-3	56.72	Vimentin-SQ-H6-3	29.257	Vimentin-TAC6-3	17.894
Vimentin-CON6-4	33.691	Vimentin-PHN6-4	61.22	Vimentin-SQ-H6-4	24.225	Vimentin-TAC6-4	28.087
Vimentin-CON6-5	50.791	Vimentin-PHN6-5	59.011	Vimentin-SQ-H6-5	28.734	Vimentin-TAC6-5	21.22

Original image for Fig. 4D

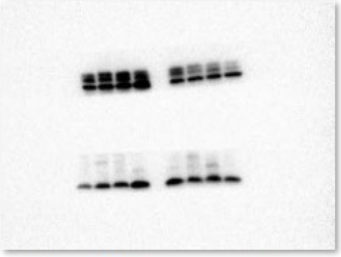

Fig 4D-Caspase-3 1.jpg

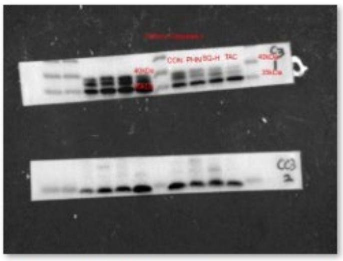

Fig 4D-Kidney-Caspase-3 1-merge image in Fig. 4D.jpg

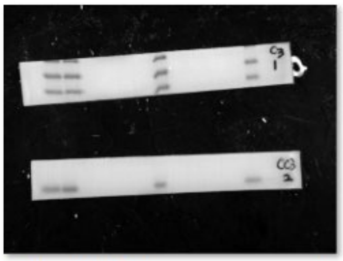

Fig 4D-Kidney-Caspase-3 1-white light.jpg

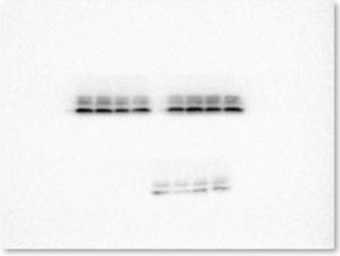

Fig 4D-Kidney-Caspase-3 2 3.jpg

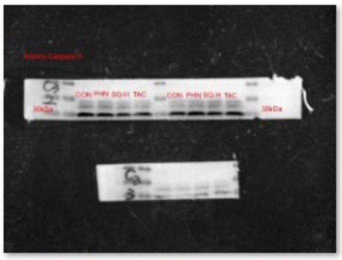

Fig 4D-Kidney-Caspase-3 2 3-merge.jpg

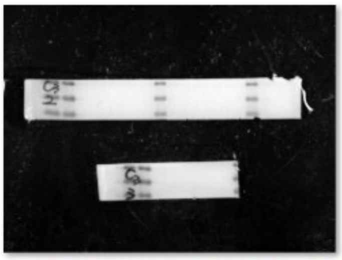

Fig 4D-Kidney-Caspase-3 2 3-white light.jpg

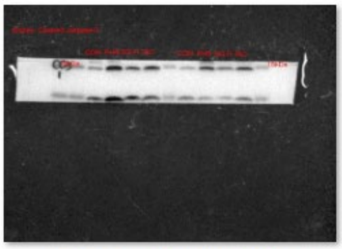

Fig 4D-Kidney-CC-3-1 2merge-imageinFig4D .jpg

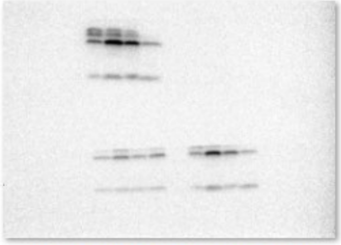

Fig 4D-Kidney-Cleaved caspase-3 3.jpg

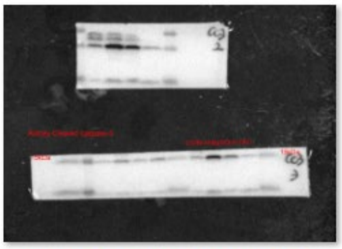

Fig 4D-Kidney-Cleaved caspase-3 3-merge.jpg

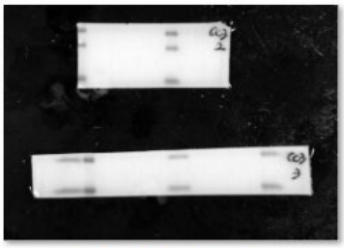

Fig 4D-Kidney-Cleaved caspase-3 3-white light.jpg

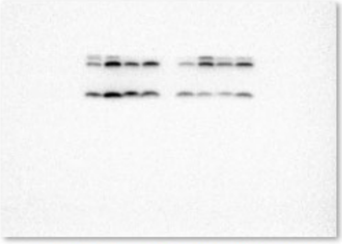

Fig 4D-Kidney-Cleaved caspase-3-1 2.jpg

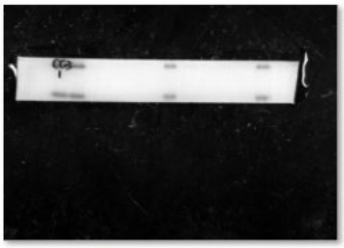

Fig 4D-Kidney-Cleaved caspase-3-1 2-white.jpg

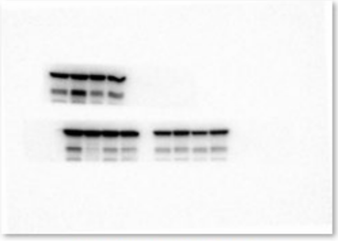

Fig 4D-GAPDH.jpg

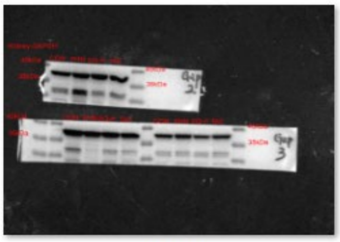

Fig 4D-Kidney-GAPDH-merge down left-image in Fig. 4D.jpg

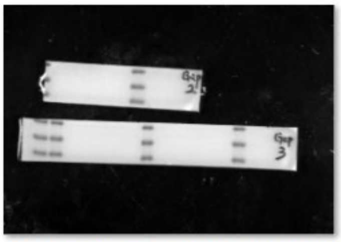

Fig 4D-Kidney-GAPDH-white light.jpg

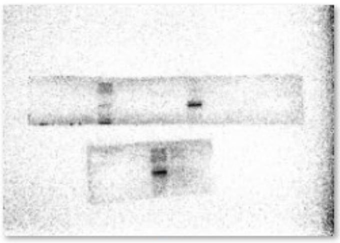

Fig 4D-Kidney-Vimentin-1.jpg

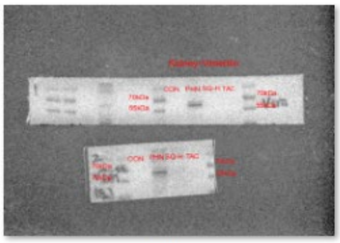

Fig 4D-Kidney-Vimentin-1-merge.jpg

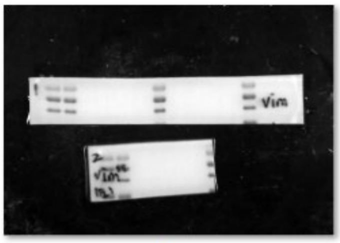

Fig 4D-Kidney-Vimentin-1-white light.jpg

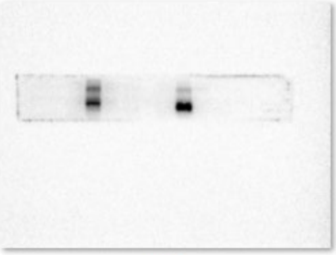

Fig 4D-Kidney-Vimentin-2.jpg

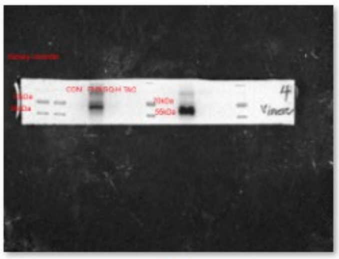

Fig 4D-Kidney-Vimentin-2-merge image in Fig. 4D.jpg

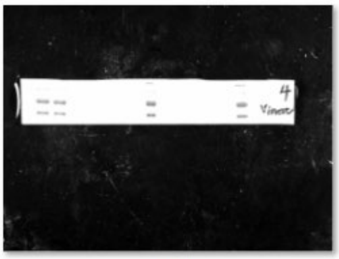

Fig 4D-Kidney-Vimentin-2-white light.jpg

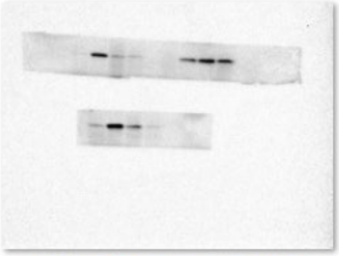

Fig 4D-Kidney-α-SMA.jpg

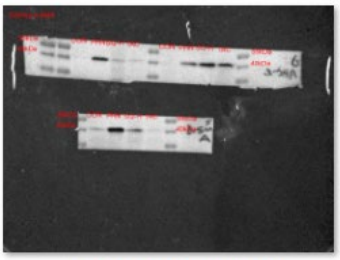

Fig 4D-Kidney-α-SMA-merge up left-image in Fig. 4D.jpg

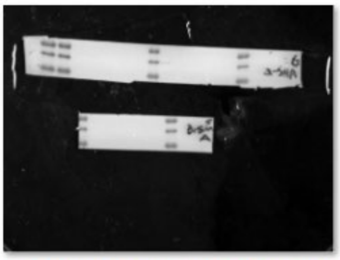

Fig 4D-Kidney-α-SMA-white light.jpg

Original data for WB-gray value

Kidney-total protein Cleaved caspase-3(Gray value)				
Group	Cleaved caspase-3(Adj.Volume)	Caspase-3(Adj.Volume)	Cleaved caspase-3/Caspase-3	Fold to control
CON	396,144.54	1,412,262.86	0.28	1.00
PHN	798,807.14	1,107,258.86	0.72	2.57
SQ-H	391,709.51	941,101.71	0.42	1.48
TAC	628,804.97	1,025,633.71	0.61	2.19
CON	507,318.82	1,035,353.14	0.49	1.00
PHN	1,704,999.29	1,174,259.43	1.45	2.96
SQ-H	744,974.12	1,006,945.71	0.74	1.51
TAC	974,104.24	1,172,593.14	0.83	1.70
CON	307,993.88	521,402.40	0.59	1.00
PHN	941,882.59	532,082.40	1.77	3.00
SQ-H	555,294.82	599,933.80	0.93	1.57
TAC	850,980.94	742,871.40	1.15	1.94

Kidney-total protein-Vimentin(Gray value)				
Group	Vimentin(Adj.Volume)	GAPDH(Adj.Volume)	Vimentin/GAPDH	Fold to control
CON	12,413.89	2,058,970.50	0.01	1.00
PHN	341,130.89	1,954,981.00	0.17	28.94
SQ-H	44,066.37	1,773,557.90	0.02	4.12
TAC	40,460.53	1,735,845.30	0.02	3.87
CON	19,072.74	969,395.70	0.02	1.00
PHN	390,915.42	933,022.76	0.55	26.99
SQ-H	25,983.74	708,564.86	0.03	1.55
TAC	26,677.05	822,152.46	0.02	0.85
CON	33,340.00	1,537,608.00	0.02	1.00
PHN	865,646.42	1,684,922.62	0.61	24.49
SQ-H	41,166.67	1,333,912.00	0.03	1.23
TAC	36,802.55	1,414,263.46	0.03	1.04

Cellular total protein-α-SMA(Gray value)				
Group	α-SMA(Adj.Volume)	GAPDH(Adj.Volume)	α-SMA/GAPDH	Fold to control
CON	33,842.53	2,058,970.50	0.02	1.00
PHN	1,237,176.88	1,954,981.00	0.63	38.50
SQ-H	256,554.65	1,773,557.90	0.14	8.80
TAC	164,104.29	1,735,845.30	0.09	5.75
CON	59,171.76	969,395.70	0.06	1.00
PHN	2,850,690.35	933,022.76	4.02	63.44
SQ-H	254,528.24	708,564.86	0.31	4.88
TAC	40,183.53	822,152.46	0.03	0.41
CON	26,379.76	1,537,608.00	0.02	1.00
PHN	2,982,368.71	1,684,922.62	2.11	106.63
SQ-H	27,732.00	1,333,912.00	0.02	1.05
TAC	42,787.06	1,414,263.46	0.03	1.53

Original image for Fig.5A

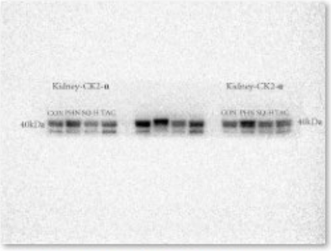

Fig 5A-Kidney-CK2α-1 2 left-image in Fig. 5A.jpg

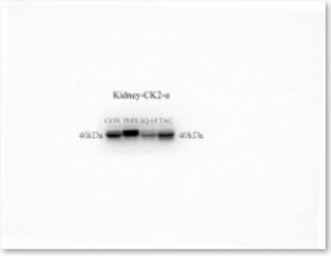

Fig 5A-Kidney-CK2α-3.jpg

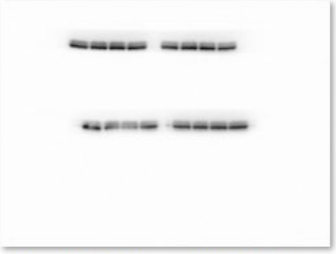

Fig 5A-Kidney-ERK 1 2.jpg

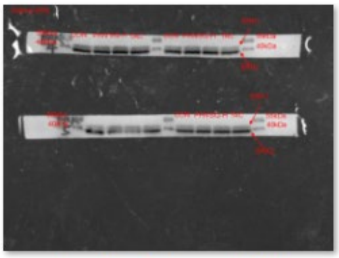

Fig 5A-Kidney-ERK 1 2-merge up right-image in Fig. 5A.jpg

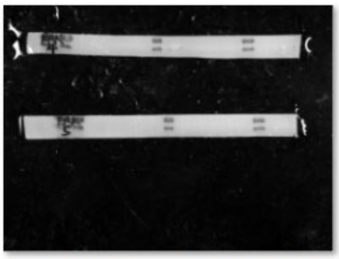

Fig 5A-Kidney-ERK 1 2-white light.jpg

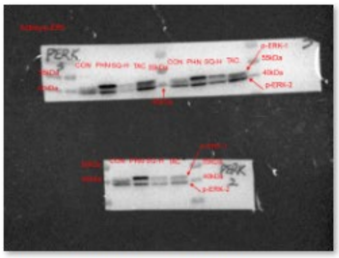

Fig 5A-Kidney-P-ERK-merge up left-image in Fig. 5A.jpg

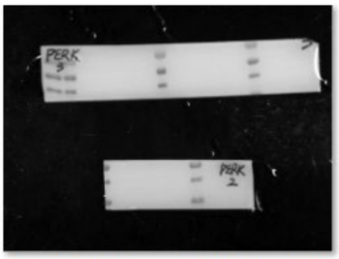

Fig 5A-Kidney-P-ERK-white light.jpg

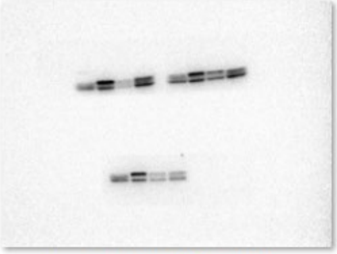

Fig 5A-P-ERK 1 2 3.jpg

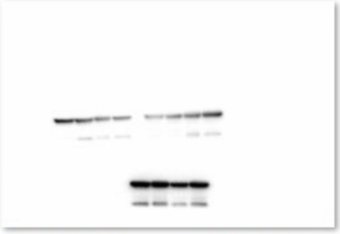

Fig 5A-Kidney-GAPDH-4 .jpg

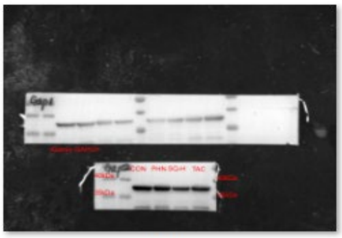

Fig 5A-Kidney-GAPDH-4-merge .jpg

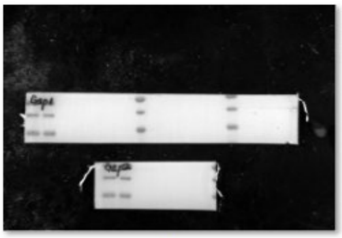

Fig 5A-Kidney-GAPDH-4-white light .jpg

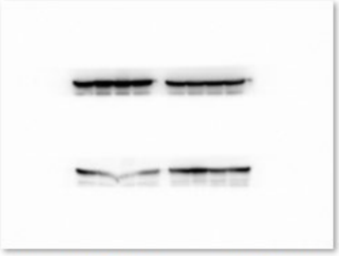

Fig 5A-Kidney-GAPDH-5 6.jpg

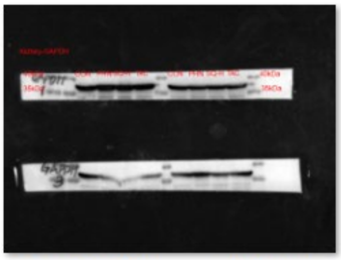

Fig 5A-Kidney-GAPDH-5 6-merge left-image in Fig. 5A.jpg

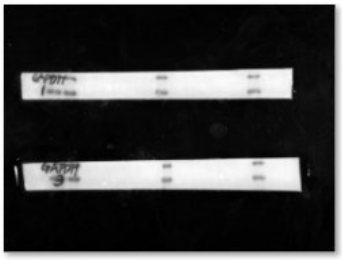

Fig 5A-Kidney-GAPDH-5 6-white light.jpg

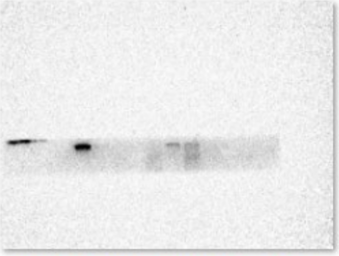

Fig 5A-Kidney-β-catenin 3.jpg

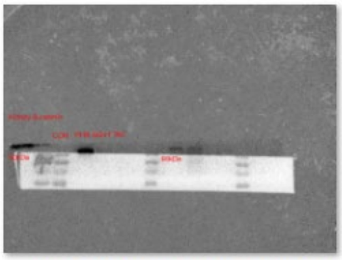

Fig 5A-Kidney-β-catenin 3-merge image in Fig. 5A.jpg

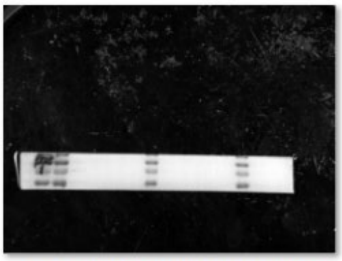

Fig 5A-Kidney-β-catenin 3-white light.jpg

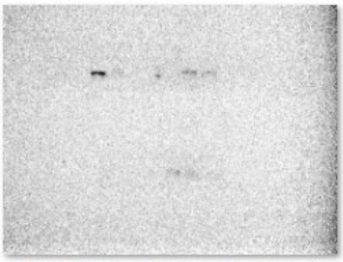

Fig 5A-Kidney-β-catenin-1 2.jpg

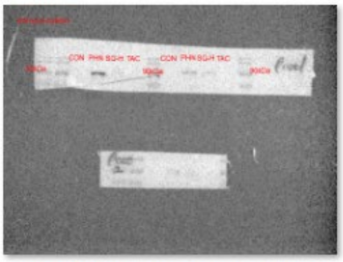

Fig 5A-Kidney-β-catenin-1 2-merge.jpg

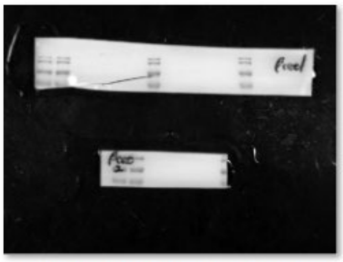

Fig 5A-Kidney-β-catenin-1 2-white light.jpg

Original data for Fig 5B-E-gray value

Kidney-total protein-p-ERK1/ERK1 (Gray value)				
Group	p-ERK1(Adj.Volume)	ERK1(Adj.Volume)	p-ERK1/ERK1	Fold to control
CON	968,902.00	237,571.00	4.08	1.00
PHN	3,052,321.00	311,209.00	9.81	2.40
SQ-H	802,124.00	249,004.00	3.22	0.79
TAC	1,958,913.00	246,654.00	7.94	1.95
CON	1,162,040.00	295,088.00	3.94	1.00
PHN	2,926,732.00	335,173.00	8.73	2.22
SQ-H	1,688,466.00	242,085.00	6.97	1.77
TAC	1,946,753.00	299,540.00	6.50	1.65
CON	1,287,741.00	228,192.00	5.64	1.00
PHN	2,075,110.00	202,085.00	10.27	1.82
SQ-H	697,277.00	214,604.00	3.25	0.58
TAC	977,014.00	220,049.00	4.44	0.79

Kidney-total protein-p-ERK2/ERK2 (Gray value)				
Group	p-ERK2(Adj.Volume)	ERK2(Adj.Volume)	p-ERK2/ERK2	Fold to control
CON	525,922.00	2,064,720.00	0.25	1.00
PHN	938,617.00	1,956,844.00	0.48	1.88
SQ-H	127,259.00	2,021,561.00	0.06	0.25
TAC	1,140,951.00	2,125,815.00	0.54	2.11
CON	616,762.00	2,010,689.00	0.31	1.00
PHN	899,476.00	1,816,262.00	0.50	1.61
SQ-H	517,457.00	2,099,723.00	0.25	0.80
TAC	1,008,350.00	2,008,719.00	0.50	1.64
CON	466,462.00	1,684,499.00	0.28	1.00
PHN	739,437.00	1,797,863.00	0.41	1.49
SQ-H	328,294.00	1,618,061.00	0.20	0.73
TAC	492,481.00	1,914,365.00	0.26	0.93

Kidney total protein-CK2-α(Gray value)				
Group	CK2-α(Adj.Volume)	GAPDH(Adj.Volume)	CK2-α/GAPDH	Fold to control
CON	22,862.48	22,833.07	1.00	1.00
PHN	29,011.46	22,711.11	1.28	1.28
SQ-H	17,133.23	22,325.60	0.77	0.77
TAC	22,630.29	22,207.31	1.02	1.02
CON	24,681.43	31,323.74	0.79	1.00
PHN	34,002.90	30,323.85	1.12	1.42
SQ-H	23,953.00	31,762.83	0.75	0.96
TAC	23,054.24	30,047.23	0.77	0.97
CON	8,420.05	20,291.54	0.41	1.00
PHN	12,284.40	20,250.90	0.61	1.46
SQ-H	6,399.75	20,208.73	0.32	0.76
TAC	8,512.97	20,375.74	0.42	1.01

Kidney total protein-β-catenin(Gray value)				
Group	β-catenin(Adj.Volume)	GAPDH(Adj.Volume)	β-catenin/GAPDH	Fold to control
CON	6,593.33	22,833.07	0.29	1.00
PHN	41,714.67	22,711.11	1.84	6.36
SQ-H	16,504.00	22,325.60	0.74	2.56
TAC	11,297.33	22,207.31	0.51	1.76
CON	37,511.50	31,323.74	1.24	1.00
PHN	167,806.80	30,323.85	5.28	4.27
SQ-H	80,433.20	31,762.83	2.68	2.16
TAC	29,880.90	30,047.23	1.47	1.19
CON	38,957.20	20,291.54	1.93	1.00
PHN	332,133.60	20,250.90	16.30	8.46
SQ-H	54,204.80	20,208.73	2.68	1.39
TAC	14,128.90	20,375.74	0.69	0.36

Original image for Fig.6B-Phalloidin

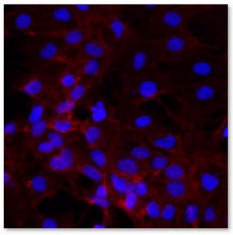
IF-Phalloidin-ADR+SQL-1-1.jpg

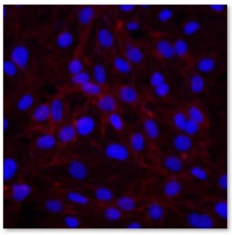
IF-Phalloidin-ADR+SQL-1-2.jpg

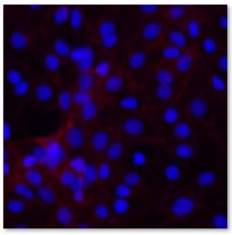
IF-Phalloidin-ADR+SQL-1-3.jpg

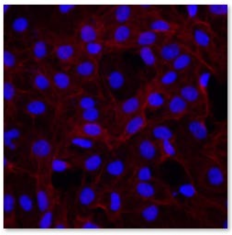
IF-Phalloidin-ADR+SQL-1-4.jpg

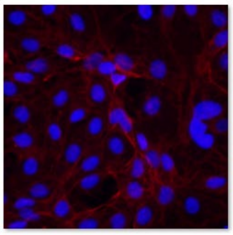
IF-Phalloidin-ADR+SQL-1-5.jpg

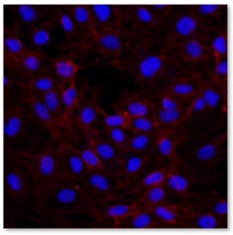
IF-Phalloidin-ADR+SQL-2-1.jpg

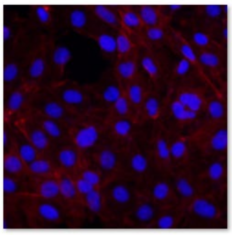
IF-Phalloidin-ADR+SQL-2-2.jpg

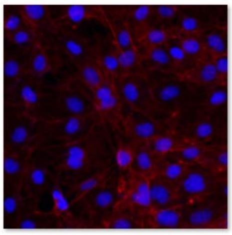
IF-Phalloidin-ADR+SQL-2-3 image in Fig. 6B.jpg

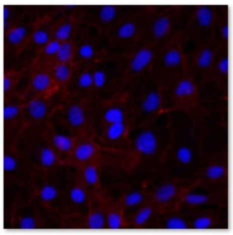
IF-Phalloidin-ADR+SQL-2-4.jpg

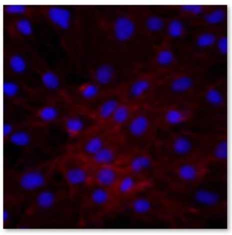
IF-Phalloidin-ADR+SQL-2-5.jpg

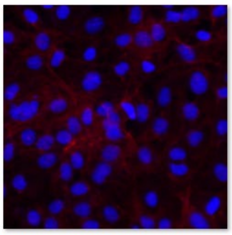
IF-Phalloidin-ADR+SQL-3-1.jpg

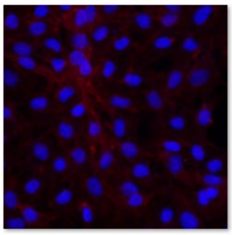
IF-Phalloidin-ADR+SQL-3-2.jpg

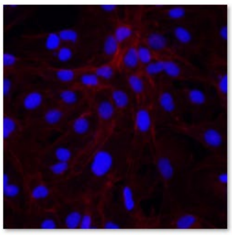
IF-Phalloidin-ADR+SQL-3-3.jpg

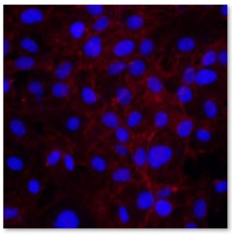
IF-Phalloidin-ADR+SQL-3-4.jpg

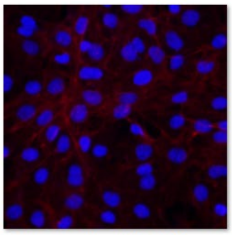
IF-Phalloidin-ADR+SQL-3-5.jpg

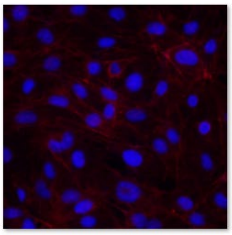
IF-Phalloidin-ADR-1-1.jpg

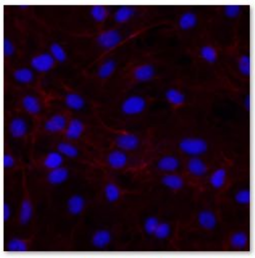
IF-Phalloidin-ADR-1-2.jpg

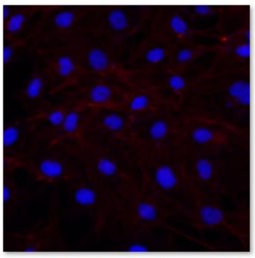
IF-Phalloidin-ADR-1-3 image in Fig. 6B.jpg

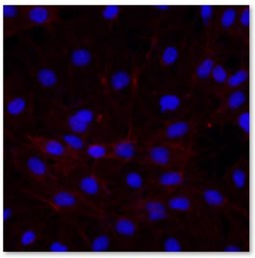
IF-Phalloidin-ADR-1-4.jpg

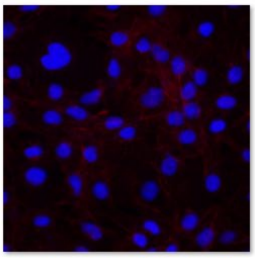
IF-Phalloidin-ADR-1-5.jpg

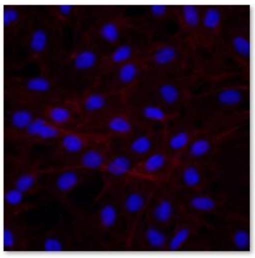
IF-Phalloidin-ADR-2-1.jpg

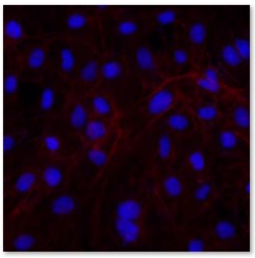
IF-Phalloidin-ADR-2-2.jpg

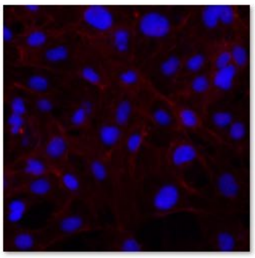
IF-Phalloidin-ADR-2-3.jpg

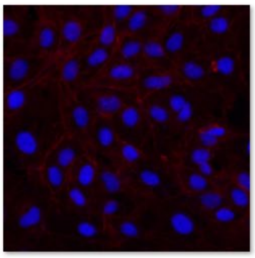
IF-Phalloidin-ADR-2-4.jpg

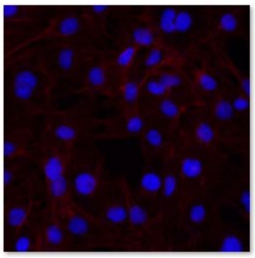
IF-Phalloidin-ADR-2-5.jpg

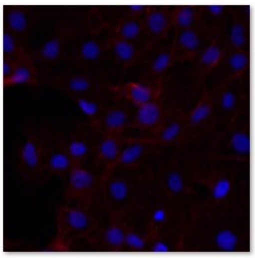
IF-Phalloidin-ADR-3-1.jpg

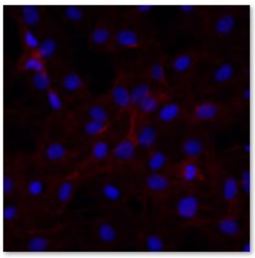
IF-Phalloidin-ADR-3-2.jpg

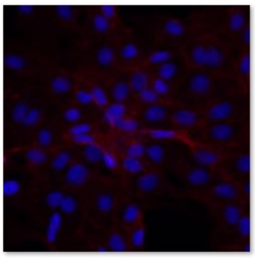
IF-Phalloidin-ADR-3-3.jpg

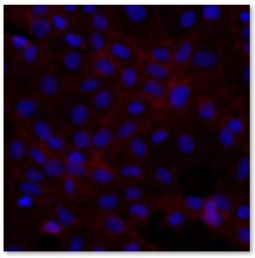
IF-Phalloidin-ADR-3-4.jpg

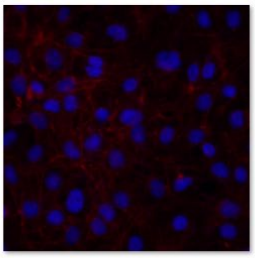
IF-Phalloidin-ADR-3-5.jpg

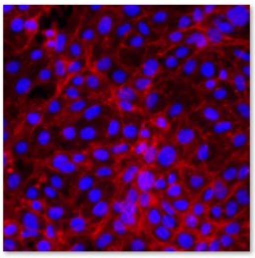
IF-Phalloidin-CON-1-1 image in Fig. 6B.jpg

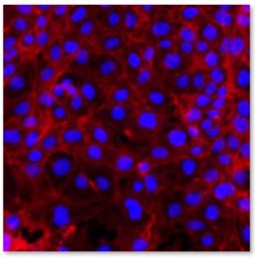
IF-Phalloidin-CON-1-2.jpg

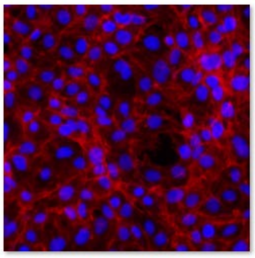
IF-Phalloidin-CON-1-3.jpg

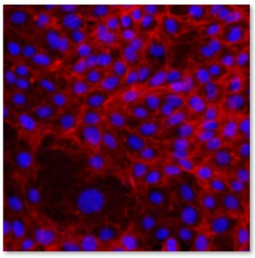
IF-Phalloidin-CON-1-4.jpg

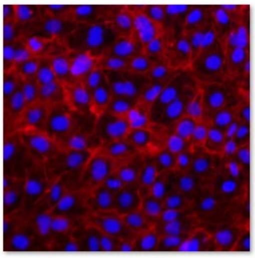
IF-Phalloidin-CON-1-5.jpg

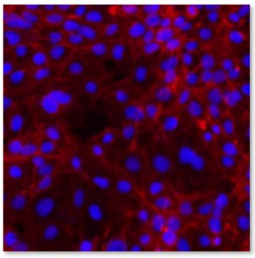
IF-Phalloidin-CON-2-1.jpg

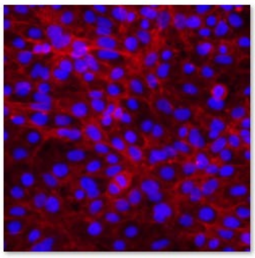
IF-Phalloidin-CON-2-2.jpg

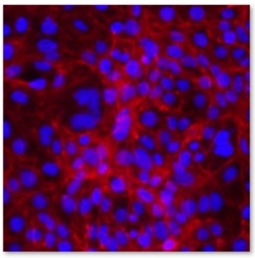
IF-Phalloidin-CON-2-3.jpg

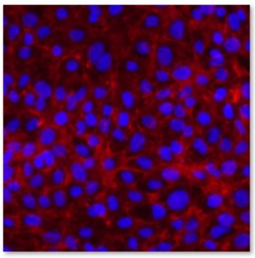
IF-Phalloidin-CON-2-4.jpg

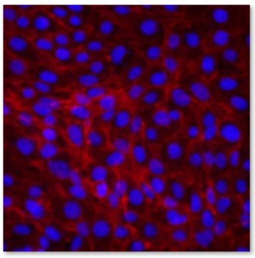
IF-Phalloidin-CON-2-5.jpg

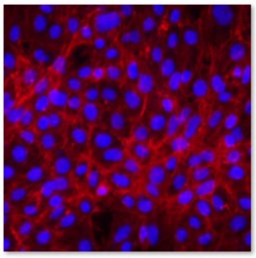

IF-Phalloidin-CON-3-1.jpg

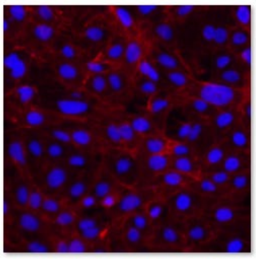

IF-Phalloidin-CON-3-2.jpg

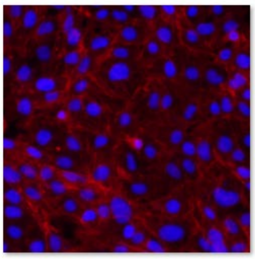

IF-Phalloidin-CON-3-3.jpg

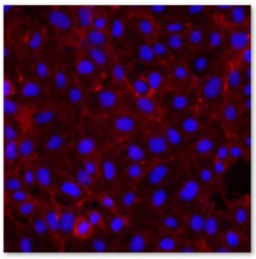

IF-Phalloidin-CON-3-4.jpg

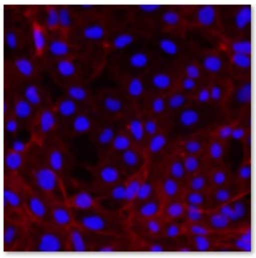

IF-Phalloidin-CON-3-5.jpg

Original image for Fig.6B-Podocyte morphology

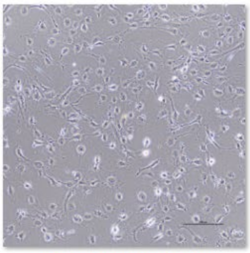

Podocyte-ADR 1.jpg

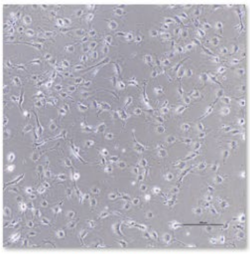

Podocyte-ADR 2 image in Fig. 6B.jpg

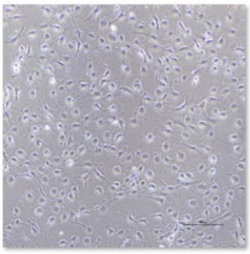

Podocyte-ADR 3.jpg

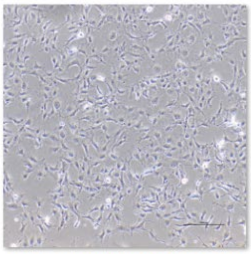

Podocyte-ADR+SQL 1.jpg

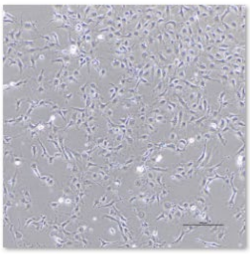

Podocyte-ADR+SQL 2.jpg

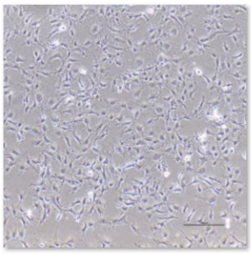

Podocyte-ADR+SQL 3 image in Fig. 6B.jpg

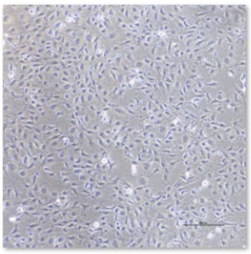

Podocyte-CON 1 image in Fig. 6B.jpg

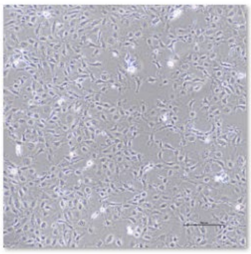

Podocyte-CON 2.jpg

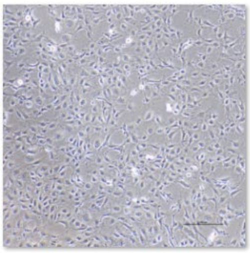

Podocyte-CON 3.jpg

Original data for Fig.6C-Phalloidin-fluorescence intensity

Podocyte-Phalloidin (Fluorescence intensity)			
No.	CON	ADR	ADR+SQL
1-1	57.247	20.928	22.656
1-2	52.162	19.547	24.009
1-3	52.616	12.972	21.130
1-4	55.811	14.642	25.200
1-5	52.140	15.331	27.482
2-1	50.961	15.030	21.872
2-2	59.763	17.381	25.308
2-3	61.420	18.729	27.788
2-4	56.033	20.551	21.104
2-5	35.529	15.529	22.452
3-1	61.394	10.713	24.394
3-2	37.878	11.875	21.606
3-3	45.601	12.687	22.955
3-4	42.806	15.579	22.650
3-5	34.714	15.396	23.176

Original image for Fig.7A

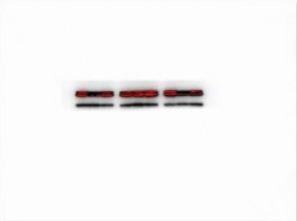
Fig 7A-Podocyte-Caspase-3 1.jpg

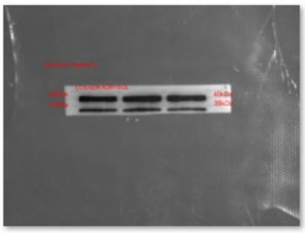
Fig 7A-Podocyte-Caspase-3 1-merge image in Fig. 7A.jpg

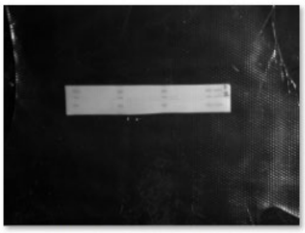
Fig 7A-Podocyte-Caspase-3 1-white light.jpg

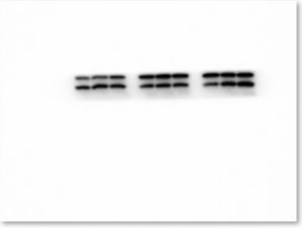
Fig 7A-Podocyte-Caspase-3 2.jpg

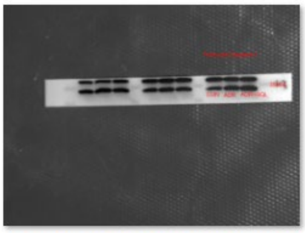
Fig 7A-Podocyte-Caspase-3 2-merge.jpg

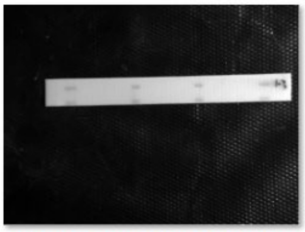
Fig 7A-Podocyte-Caspase-3 2-white light.jpg

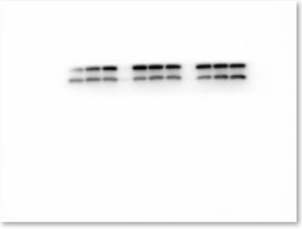
Fig 7A-Podocyte-Caspase-3 3.jpg

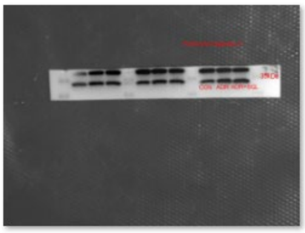
Fig 7A-Podocyte-Caspase-3 3-merge.jpg

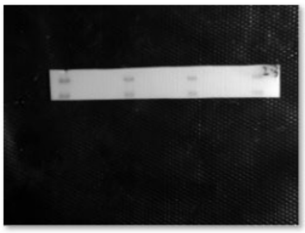
Fig 7A-Podocyte-Caspase-3 3-white light.jpg

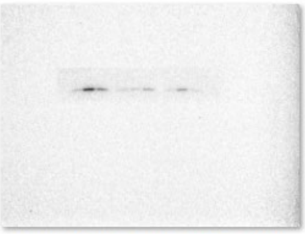

Fig 7A-Podocyte-Cleaved caspase-3 1.jpg

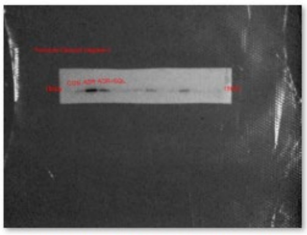

Fig 7A-Podocyte-Cleaved caspase-3 1-merge image in Fig. 7A.jpg

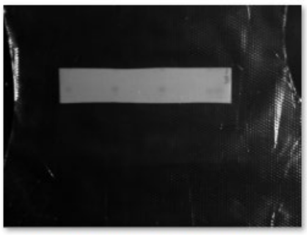

Fig 7A-Podocyte-Cleaved caspase-3 1-white light.jpg

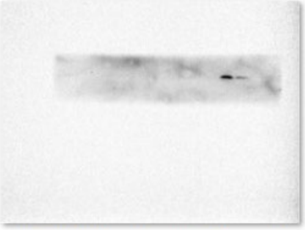

Fig 7A-Podocyte-Cleaved caspase-3 2.jpg

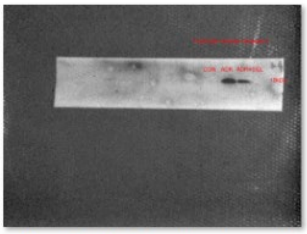

Fig 7A-Podocyte-Cleaved caspase-3 2-merge.jpg

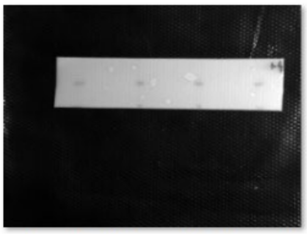

Fig 7A-Podocyte-Cleaved caspase-3 2-white light.jpg

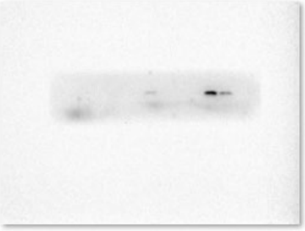

Fig 7A-Podocyte-Cleaved caspase-3 3.jpg

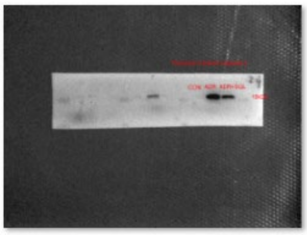

Fig 7A-Podocyte-Cleaved caspase-3 3-merge.jpg

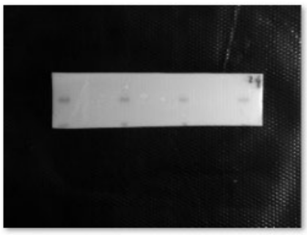

Fig 7A-Podocyte-Cleaved caspase-3 3-white light.jpg

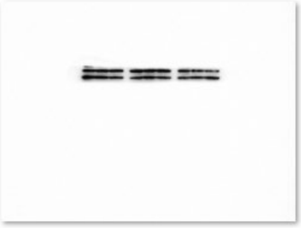

Fig 7A-Podocyte-GAPDH 1.jpg

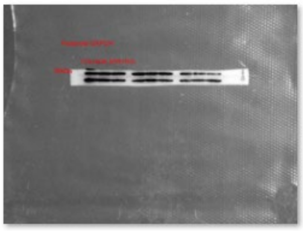

Fig 7A-Podocyte-GAPDH 1-merge.jpg

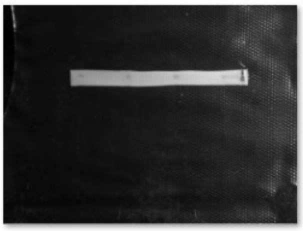

Fig 7A-Podocyte-GAPDH 1-white light.jpg

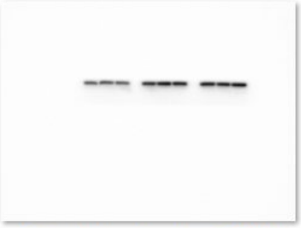

Fig 7A-Podocyte-GAPDH 2.jpg

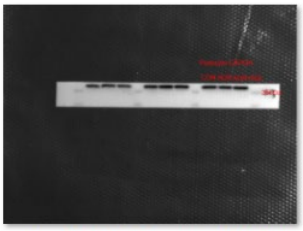

Fig 7A-Podocyte-GAPDH 2-merge image in Fig. 7A.jpg

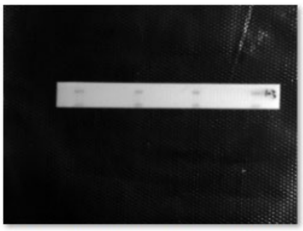

Fig 7A-Podocyte-GAPDH 2-white light.jpg

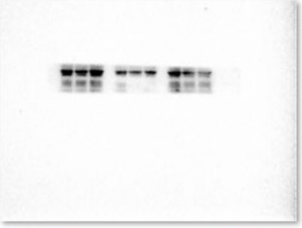

Fig 7A-Podocyte-Podocin 1 2.jpg

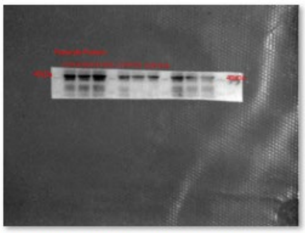

Fig 7A-Podocyte-Podocin 1 2-merge right-image in Fig. 7A.jpg

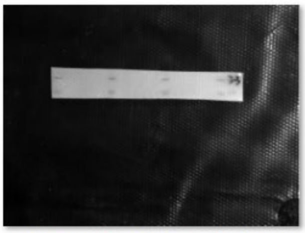

Fig 7A-Podocyte-Podocin 1 2-white light.jpg

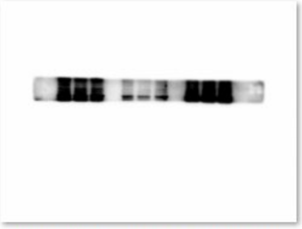

Fig 7A-Podocyte-Podocin 3.jpg

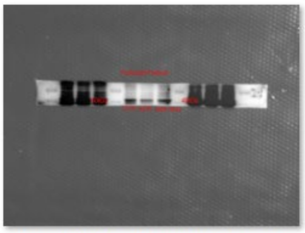

Fig 7A-Podocyte-Podocin 3-merge.jpg

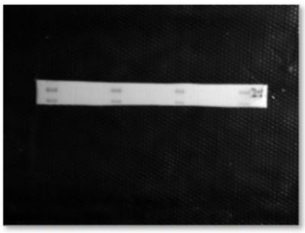

Fig 7A-Podocyte-Podocin 3-white light.jpg

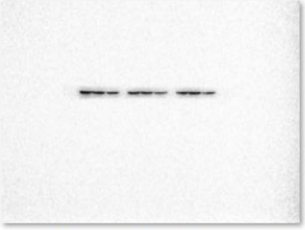

Fig 7A-Podocyte-Vimentin 1.jpg

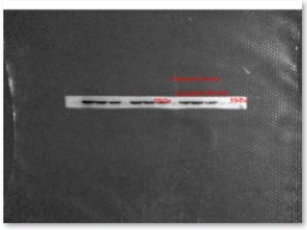

Fig 7A-Podocyte-Vimentin 1-merge image in Fig. 7A.jpg

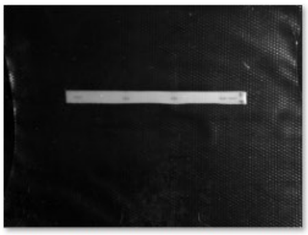

Fig 7A-Podocyte-Vimentin 1-white light.jpg

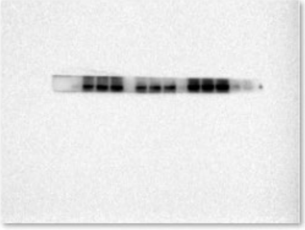

Fig 7A-Podocyte-Vimentin 2.jpg

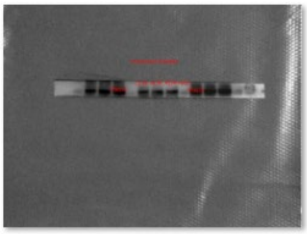

Fig 7A-Podocyte-Vimentin 2-merge.jpg

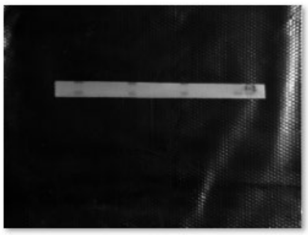

Fig 7A-Podocyte-Vimentin 2-white light.jpg

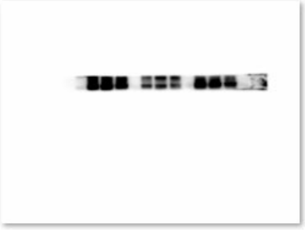

Fig 7A-Podocyte-Vimentin 3.jpg

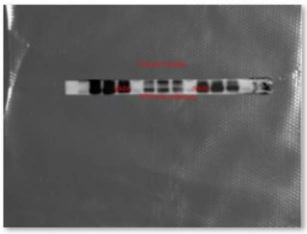

Fig 7A-Podocyte-Vimentin 3-merge.jpg

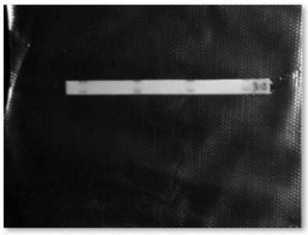

Fig 7A-Podocyte-Vimentin 3-white light.jpg

Original data for Fig. 8B-D-WB gray value

Podocyte-total protein-Cleaved caspase-3/Caspase-3(Gray value)				
Group	Cleaved caspase-3(Adj.Volume)	Caspase-3(Adj.Volume)	Cleaved caspase-3/Caspase-3	Fold to control
CON	110,395	1,539,458	0.071710303	1.00
ADR	2,112,477	1,560,562	1.353664257	18.88
ADR+SQL	465,725	1,483,336	0.313971346	4.38
CON	51,242	2,972,376	0.017239407	1.00
ADR	3,462,727	3,150,984	1.098935126	63.75
ADR+SQL	629,735	6,159,785	0.102233276	5.93
CON	21,575	3,598,438	0.005995657	1.00
ADR	3,235,629	4,480,392	0.722175426	120.44
ADR+SQL	644,988	6,788,825	0.09500731	15.85

Podocyte-total protein-Podocin(Gray value)				
Group	Podocin(Adj.Volume)	GAPDH(Adj.Volume)	Podocin/GAPDH	Fold to control
CON	2,018,792	3,163,739	0.638103206	1.00
ADR	902,467	3,125,179	0.288772899	0.45
ADR+SQL	2,020,513	3,412,666	0.592062921	0.93
CON	1,709,284	2,667,731	0.64072577	1.00
ADR	946,319	2,258,003	0.419095546	0.65
ADR+SQL	1,836,129	2,550,788	0.719828147	1.12
CON	2,056,218	5,041,942	0.407822621	1.00
ADR	1,235,813	4,990,279	0.24764407	0.61
ADR+SQL	2,179,187	4,862,165	0.448192729	1.10

Podocyte-total protein-Vimentin(Gray value)				
Group	Vimentin(Adj.Volume)	GAPDH(Adj.Volume)	Vimentin/GAPDH	Fold to control
CON	874,436	2,406,907	0.363302778	1.00
ADR	1,039,537	2,371,159	0.438408812	1.21
ADR+SQL	702,519	2,209,823	0.317907362	0.88
CON	3,043,436	2,114,785	1.439123126	1.00
ADR	3,381,461	1,622,771	2.083757351	1.45
ADR+SQL	2,801,014	2,196,546	1.27519023	0.89
CON	2,495,449	3,632,646	0.686950779	1.00
ADR	3,729,421	3,160,475	1.180019143	1.72
ADR+SQL	2,401,671	3,658,079	0.656538856	0.96

Original image for Fig. 7E-Vimentin

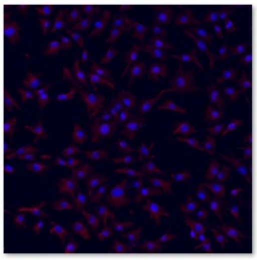
Vimentin-IF-ADR+SQL1-1.jpg

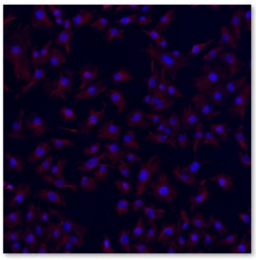
Vimentin-IF-ADR+SQL1-2.jpg

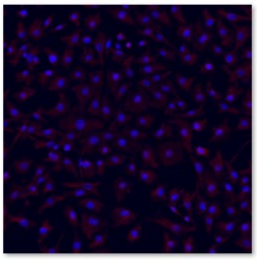
Vimentin-IF-ADR+SQL1-3.jpg

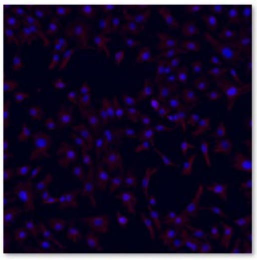
Vimentin-IF-ADR+SQL1-4.jpg

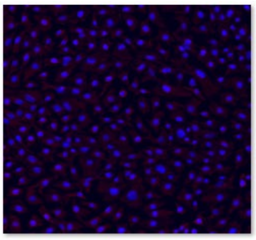
Vimentin-IF-ADR+SQL1-5.jp
g

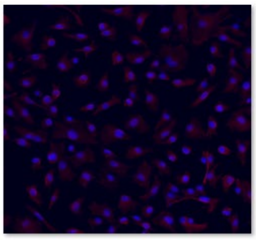
Vimentin-IF-ADR+SQL2-1.jp
g

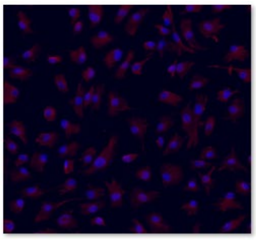
Vimentin-IF-ADR+SQL2-2.jp
g

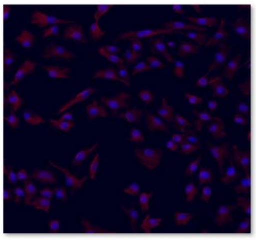
Vimentin-IF-ADR+SQL2-3.jp
g

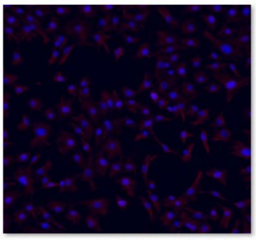
Vimentin-IF-ADR+SQL2-4.jp
g

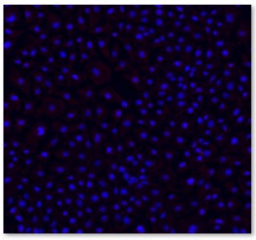
Vimentin-IF-ADR+SQL2-5.jp
g

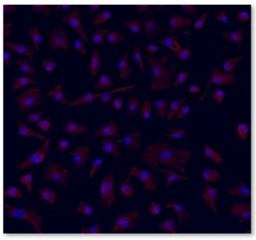
Vimentin-IF-ADR+SQL3-1.jp
g

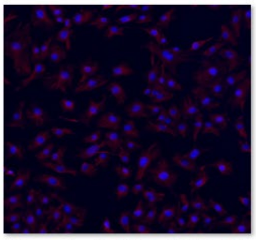
Vimentin-IF-ADR+SQL3-2.jp
g

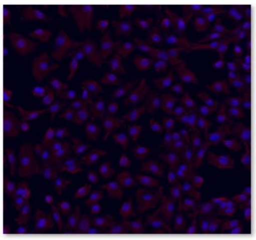
Vimentin-IF-ADR+SQL3-4.jp
g

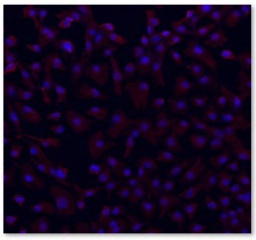
Vimentin-IF-ADR+SQL3-5
image in Fig. 7E.jpg

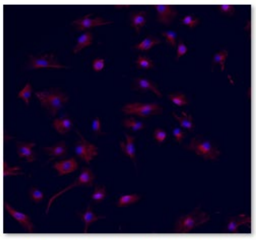
Vimentin-IF-ADR1-1.jpg

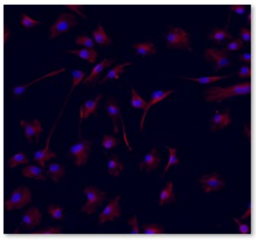
Vimentin-IF-ADR1-2.jpg

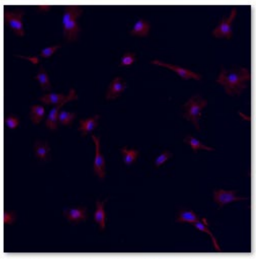
Vimentin-IF-ADR1-3.jpg

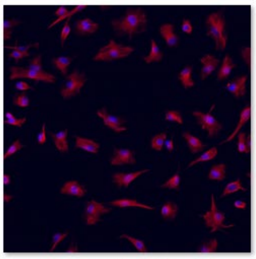
Vimentin-IF-ADR1-4.jpg

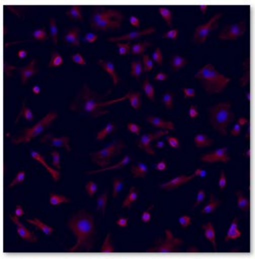
Vimentin-IF-ADR1-5.jpg

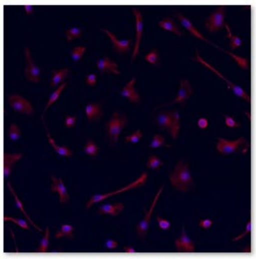
Vimentin-IF-ADR2-1.jpg

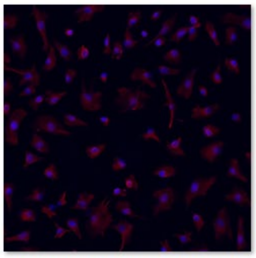
Vimentin-IF-ADR2-2.jpg

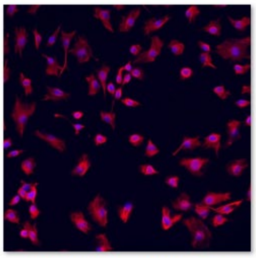
Vimentin-IF-ADR2-3.jpg

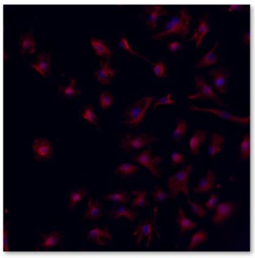
Vimentin-IF-ADR2-4.jpg

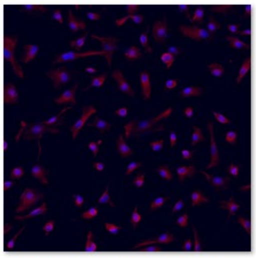
Vimentin-IF-ADR2-5.jpg

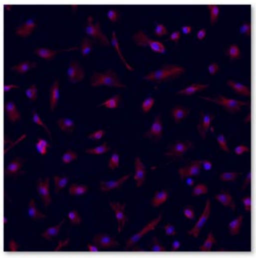
Vimentin-IF-ADR3-1.jpg

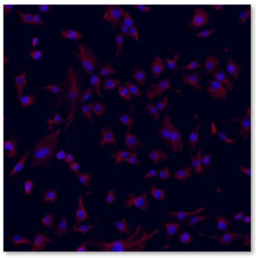
Vimentin-IF-ADR3-2.jpg

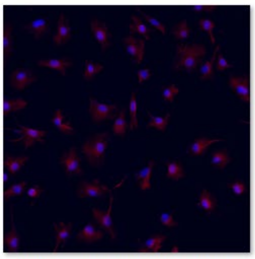
Vimentin-IF-ADR3-3.jpg

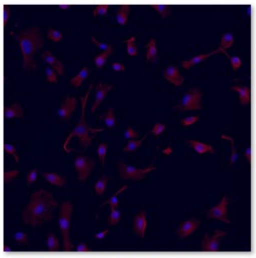
Vimentin-IF-ADR3-4.jpg

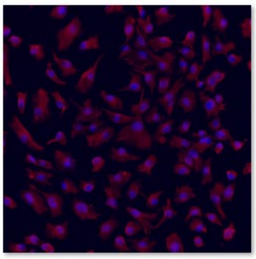
Vimentin-IF-ADR3-5.jpg

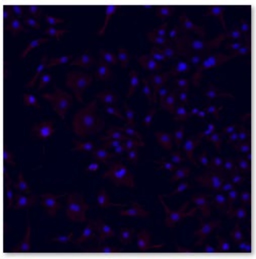
Vimentin-IF-CON1-1.jpg

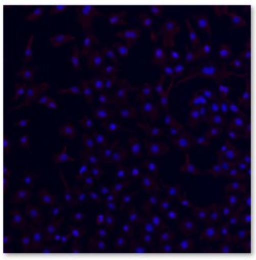
Vimentin-IF-CON1-2.jpg

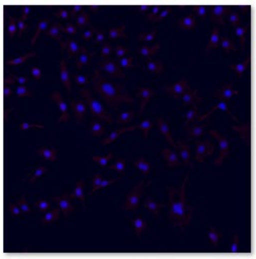
Vimentin-IF-CON1-3.jpg

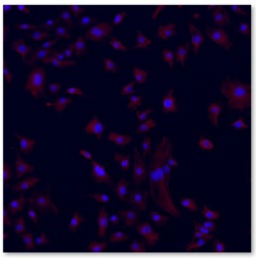
Vimentin-IF-CON1-4.jpg

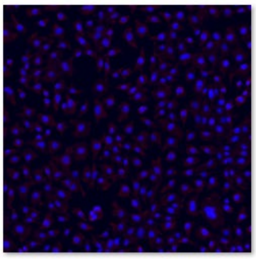
Vimentin-IF-CON1-5.jpg

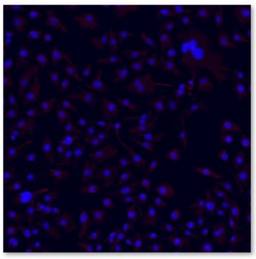
Vimentin-IF-CON2-1.jpg

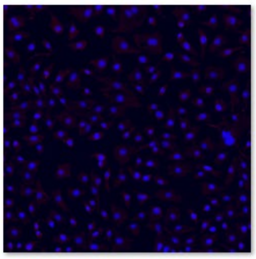
Vimentin-IF-CON2-2.jpg

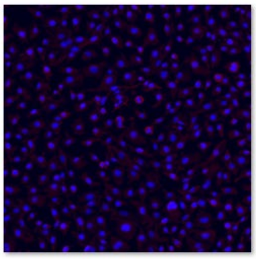
Vimentin-IF-CON2-3.jpg

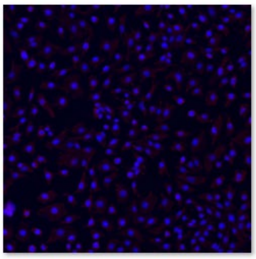
Vimentin-IF-CON2-4 image in Fig. 7E.jpg

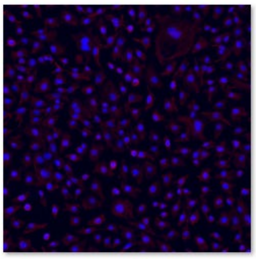
Vimentin-IF-CON2-5.jpg

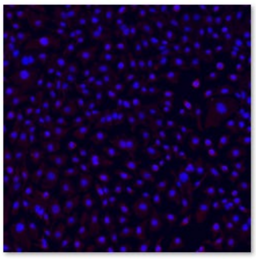
Vimentin-IF-CON3-1.jpg

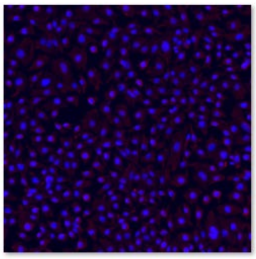
Vimentin-IF-CON3-2.jpg

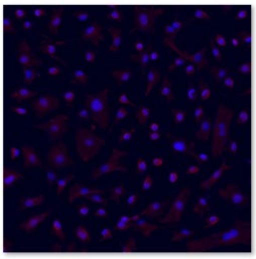
Vimentin-IF-CON3-3.jpg

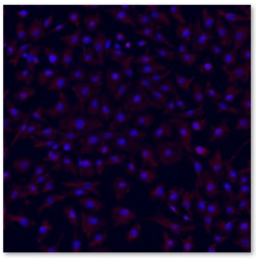
Vimentin-IF-CON3-4.jpg

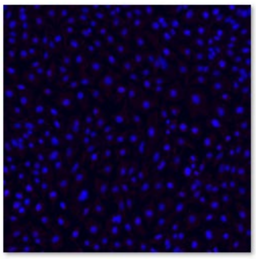
Vimentin-IF-CON3-5.jpg

Original image for Fig. 7E-α-SMA

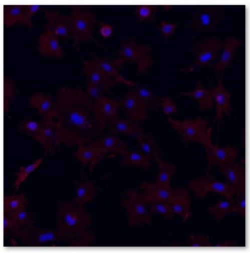
α-SMA-IF-ADR+SQL1-1.jpg

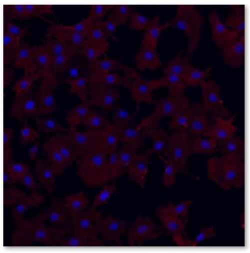
α-SMA-IF-ADR+SQL1-2.jpg

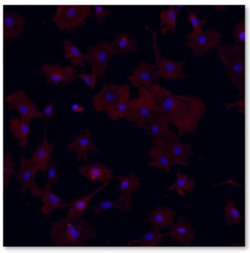
α-SMA-IF-ADR+SQL1-3.jpg

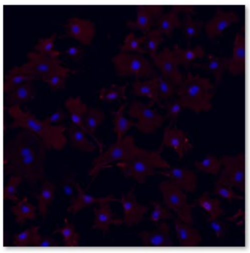
α-SMA-IF-ADR+SQL1-4.jpg

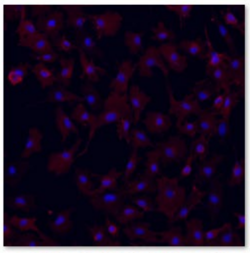
α-SMA-IF-ADR+SQL1-5.jpg

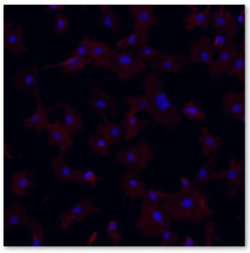
α-SMA-IF-ADR+SQL2-1.jpg

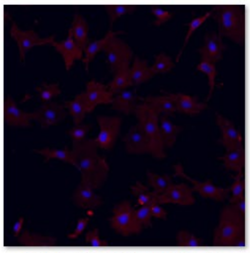
α-SMA-IF-ADR+SQL2-2.jpg

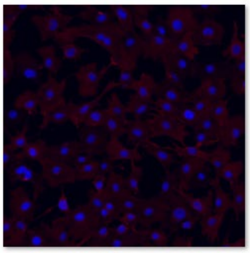
α-SMA-IF-ADR+SQL2-3.jpg

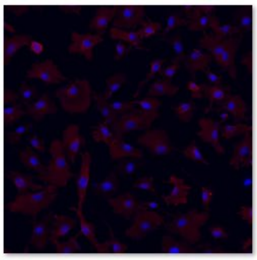
α-SMA-IF-ADR+SQL2-4.jpg

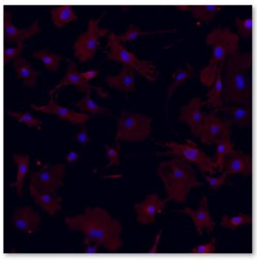
α-SMA-IF-ADR+SQL2-5.jpg

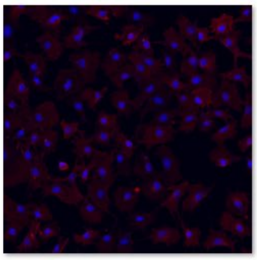
α-SMA-IF-ADR+SQL3-1 image in Fig. 7E.jpg

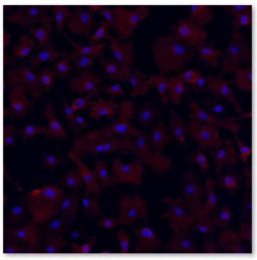
α-SMA-IF-ADR+SQL3-2.jpg

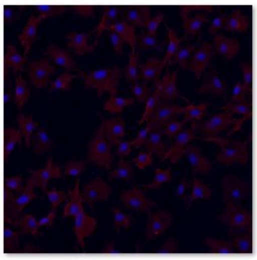
α-SMA-IF-ADR+SQL3-3.jpg

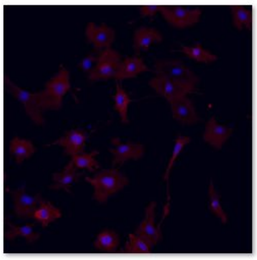
α-SMA-IF-ADR+SQL3-4.jpg

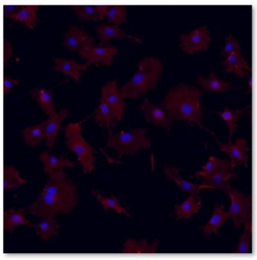
α-SMA-IF-ADR+SQL3-5.jpg

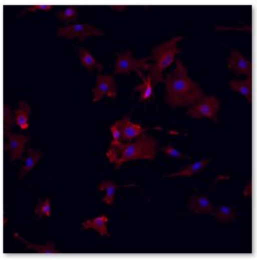
α-SMA-IF-ADR1-1.jpg

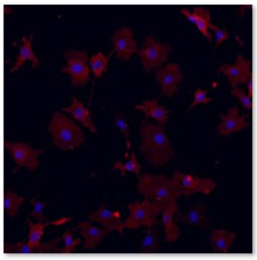
α-SMA-IF-ADR1-2.jpg

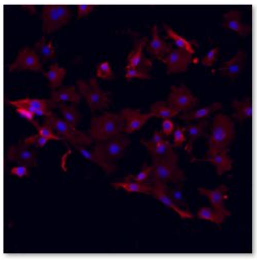
α-SMA-IF-ADR1-3 image in Fig. 7E.jpg

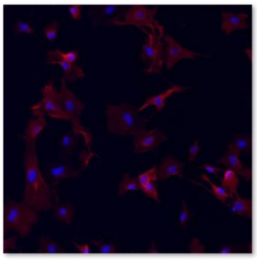
α-SMA-IF-ADR1-4.jpg

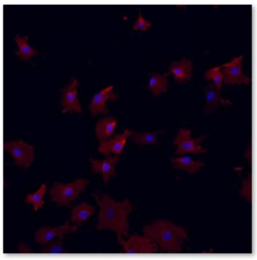
α-SMA-IF-ADR1-5.jpg

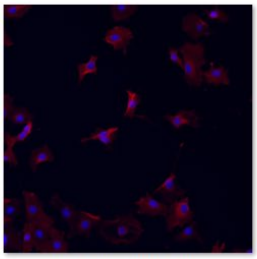
α-SMA-IF-ADR2-1.jpg

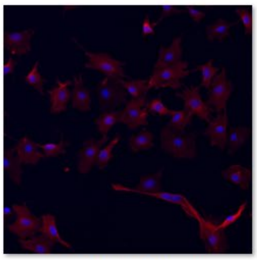
α-SMA-IF-ADR2-2.jpg

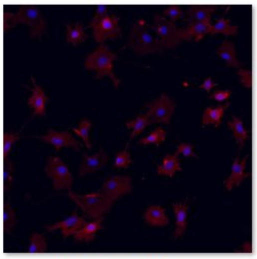
α-SMA-IF-ADR2-3.jpg

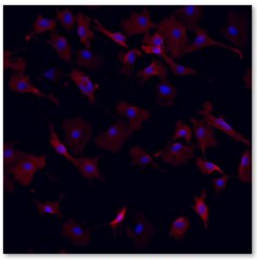
α-SMA-IF-ADR2-4.jpg

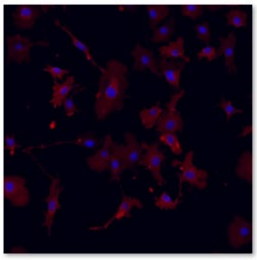
α-SMA-IF-ADR2-5.jpg

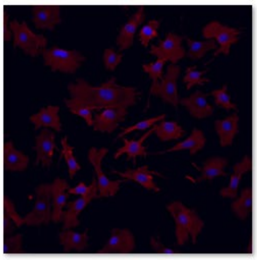
α-SMA-IF-ADR3-1.jpg

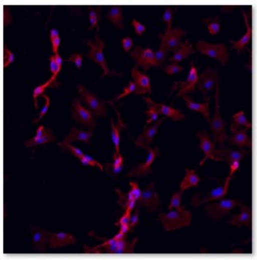
α-SMA-IF-ADR3-2.jpg

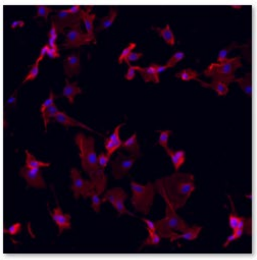
α-SMA-IF-ADR3-3.jpg

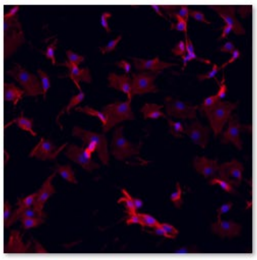
α-SMA-IF-ADR3-4.jpg

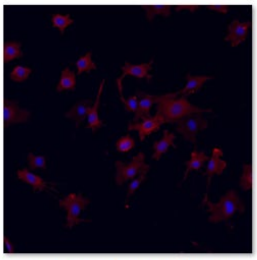
α-SMA-IF-ADR3-5.jpg

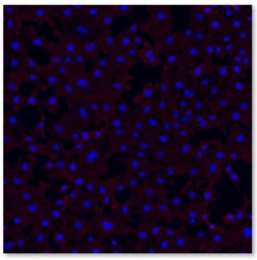
α-SMA-IF-CON1-1.jpg

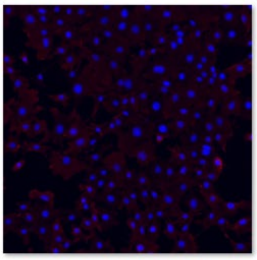
α-SMA-IF-CON1-2.jpg

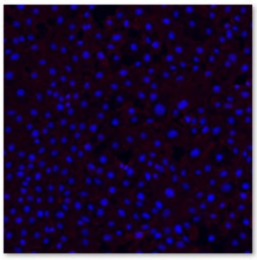
α-SMA-IF-CON1-3.jpg

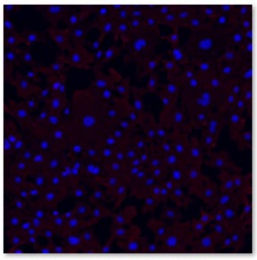
α-SMA-IF-CON1-4.jpg

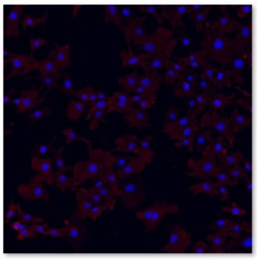
α-SMA-IF-CON1-5.jpg

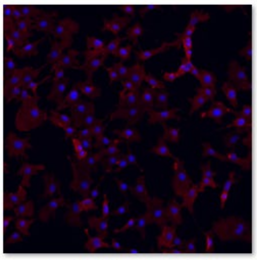
α-SMA-IF-CON2-1.jpg

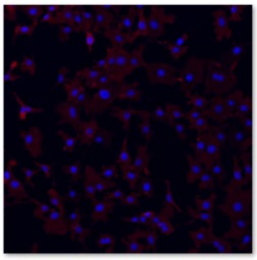
α-SMA-IF-CON2-2.jpg

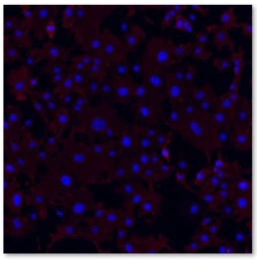
α-SMA-IF-CON2-3 image in Fig. 7E.jpg

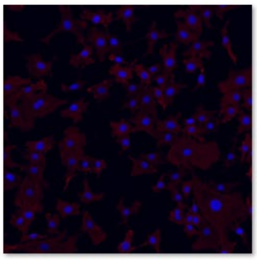
α-SMA-IF-CON2-4.jpg

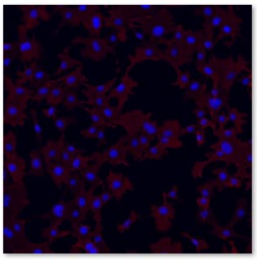
α-SMA-IF-CON2-5.jpg

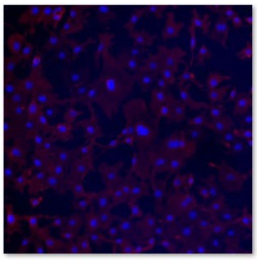
α-SMA-IF-CON3-1.jpg

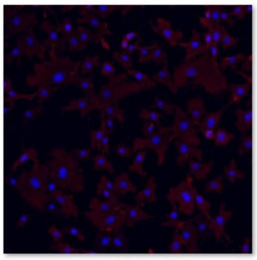
α-SMA-IF-CON3-2.jpg

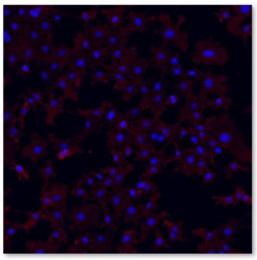
α-SMA-IF-CON3-3.jpg

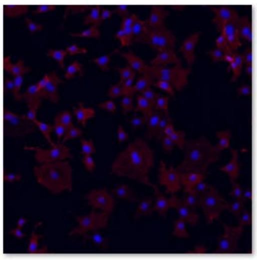
α-SMA-IF-CON3-4.jpg

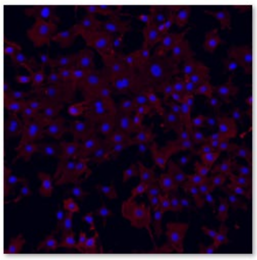
α-SMA-IF-CON3-5.jpg

Original data for Fig. 7F-G

Podocyte-Vimentin (Fluorescence intensity)			
No.	CON	ADR	ADR+SQL
1-1	19.182	21.473	12.019
1-2	7.730	26.293	16.632
1-3	8.355	23.156	23.208
1-4	16.398	21.950	11.812
1-5	20.508	23.905	18.037
2-1	13.323	26.479	16.507
2-2	12.714	26.624	16.410
2-3	9.937	26.388	15.313
2-4	11.236	21.947	13.294
2-5	15.875	19.503	11.587
3-1	18.585	30.552	17.776
3-2	19.799	28.538	14.586
3-3	15.690	32.918	16.910
3-4	13.658	33.826	24.951
3-5	10.241	30.476	13.225

Podocyte-α-SMA (Fluorescence intensity)			
No.	CON	ADR	ADR+SQL
1-1	14.596	16.065	16.172
1-2	15.041	16.903	11.209
1-3	12.927	19.297	14.527
1-4	13.028	21.153	13.013
1-5	12.835	19.085	12.424
2-1	11.637	19.245	16.350
2-2	12.707	16.640	16.337
2-3	12.616	19.250	16.221
2-4	9.167	19.458	10.883
2-5	11.542	21.879	14.425
3-1	9.672	25.132	12.757
3-2	9.675	20.708	12.224
3-3	8.104	17.330	14.986
3-4	8.962	16.486	14.577
3-5	12.464	16.577	15.365

Original image for Fig. 8A

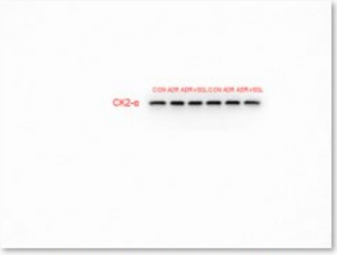

Fig 8A-Podocyte-CK2α 1 2.jpg

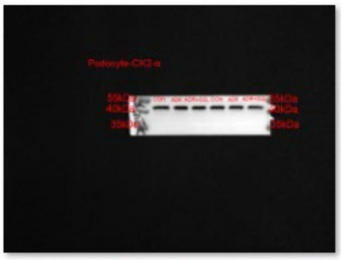

Fig 8A-Podocyte-CK2α 1 2-merge.jpg

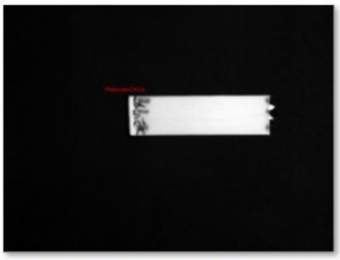

Fig 8A-Podocyte-CK2α 1 2-white light.jpg

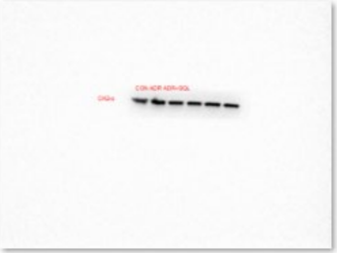

Fig 8A-Podocyte-CK2α 3.jpg

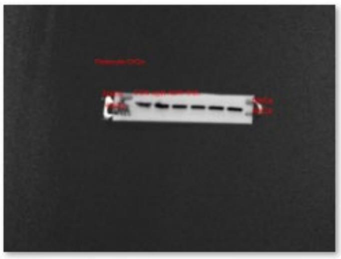

Fig 8A-Podocyte-CK2α 3-merge image in Fig. 8A.jpg

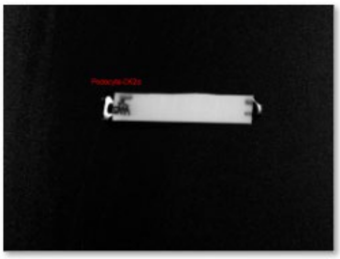

Fig 8A-Podocyte-CK2α 3-white light.jpg

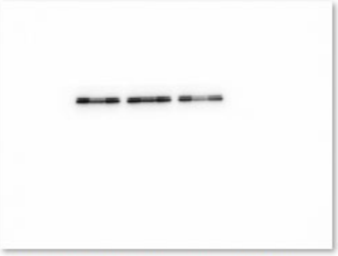

Fig 8A-Podocyte-ERK.jpg

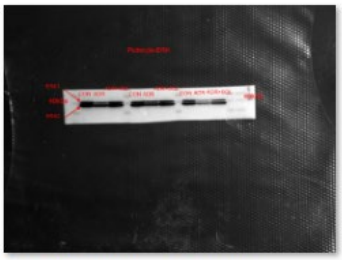

Fig 8A-Podocyte-ERK-merge right-image in Fig.8A.jpg

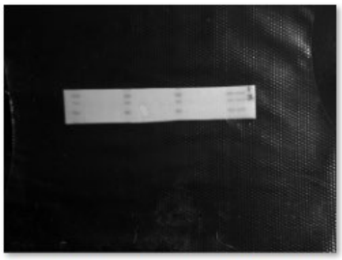

Fig 8A-Podocyte-ERK-white light.jpg

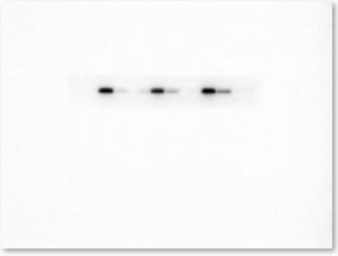

Fig 8A-Podocyte-p-ERK.jpg

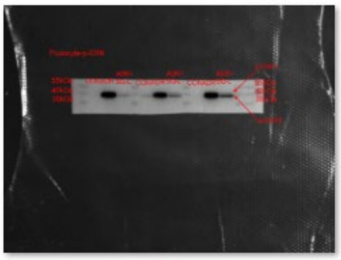

Fig 8A-Podocyte-p-ERK-merge middle-image in Fig. 8A.jpg

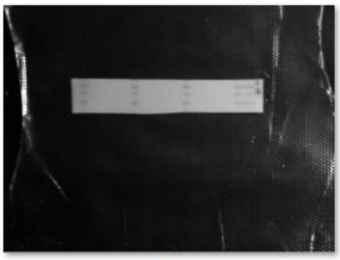

Fig 8A-Podocyte-p-ERK-white light.jpg

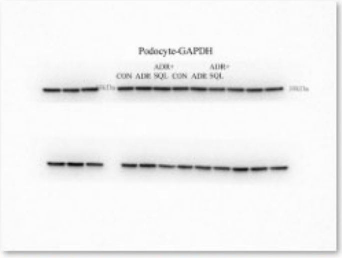

Fig 8A-Podocyte-GAPDH-1 2 left-image in Fig. 8A.jpg

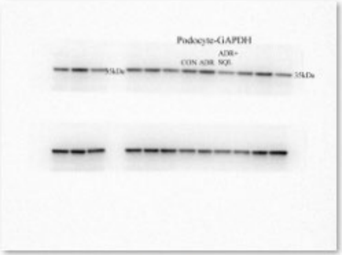

Fig 8A-Podocyte-GAPDH-3.jpg

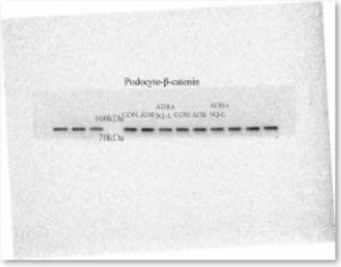

Fig 8A-Podocyte-β-catenin-1 2 left-image in Fig. 8A.jpg

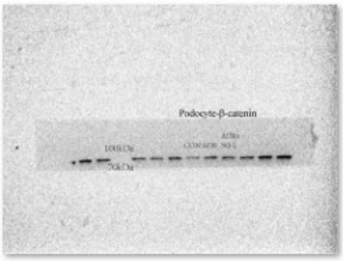

Fig 8A-Podocyte-β-catenin-3.jpg

Original data for Fig. 8B-E

Podocyte-total protein-p-ERK1/ERK1(Gray value)				
Group	p-ERK1(Adj.Volume)	ERK1(Adj.Volume)	p-ERK1/ERK1	Fold to control
CON	1,082	897,754	0.00120523	1.00
ADR	137,562	142,497	0.965367692	800.98
ADR+SQL	1,306	724,206	0.001803354	1.50
CON	1,136	783,029	0.001450776	1.00
ADR	312,776	365,967	0.854656294	589.10
ADR+SQL	38,548	832,481	0.046304961	31.92
CON	6,414	768,328	0.008347997	1.00
ADR	1,253,532	145,013	8.644273272	1035.49
ADR+SQL	512,760	513,597	0.998370318	119.59

Podocyte-total protein-p-ERK2/ERK2(Gray value)				
Group	p-ERK2(Adj.Volume)	ERK2(Adj.Volume)	p-ERK2/ERK2	Fold to control
CON	9,007	1,719,821	0.005237173	1.00
ADR	2,551,561	610,813	4.177319409	797.63
ADR+SQL	70,578	1,964,709	0.035922877	6.86
CON	16,674	1,490,118	0.011189718	1.00
ADR	1,697,593	772,904	2.196382733	196.29
ADR+SQL	310,745	1,507,474	0.206136225	18.42
CON	1,602	1,248,701	0.001282933	1.00
ADR	1,275,438	620,880	2.054242366	1601.21
ADR+SQL	214,118	1,472,273	0.145433625	113.36

Podocyte-total protein-CK2-α(Gray value)				
Group	CK2-α(Adj.Volume)	GAPDH(Adj.Volume)	CK2-α/GAPDH	Fold to control
CON	1,489,759	733,633	2.030659744	1.00
ADR	1,688,171	739,826	2.281848705	1.12
ADR+SQL	1,430,318	711,880	2.009212227	0.99
CON	1,493,089	686,827	2.173893863	1.00
ADR	1,598,980	693,932	2.304231539	1.06
ADR+SQL	1,408,620	707,078	1.992170595	0.92
CON	2,379,933	196,772	12.0948763	1.00
ADR	2,807,917	193,163	14.53651579	1.20
ADR+SQL	2,032,199	200,448	10.13828524	0.84

Podocyte-total protein-β-catenin(Gray value)				
Group	β-catenin(Adj.Volume)	GAPDH(Adj.Volume)	β-catenin/GAPDH	Fold to control
CON	648,316	1,236,908	0.524142458	1.00
ADR	731,967	1,145,334	0.639086066	1.22
ADR+SQL	622,432	1,279,915	0.486307294	0.93
CON	800,897	1,132,095	0.707446813	1.00
ADR	946,444	1,110,148	0.85253858	1.21
ADR+SQL	650,980	1,072,422	0.607018506	0.86
CON	653,707	1,032,713	0.632999681	1.00
ADR	796,259	1,052,676	0.756414129	1.19
ADR+SQL	646,478	1,019,088	0.634369161	1.00
