# Supplementary figures and images for: Sanqi oral solution alleviates podocyte apoptosis in experimental membranous nephropathy by mediating EMT through the ERK/CK2-α/β-catenin pathway (part 4 of 4)
Source: Front Pharmacol. 2025 May 9;16:1503961. doi: 10.3389/fphar.2025.1503961 (PMC12098599; doi:10.3389/fphar.2025.1503961)

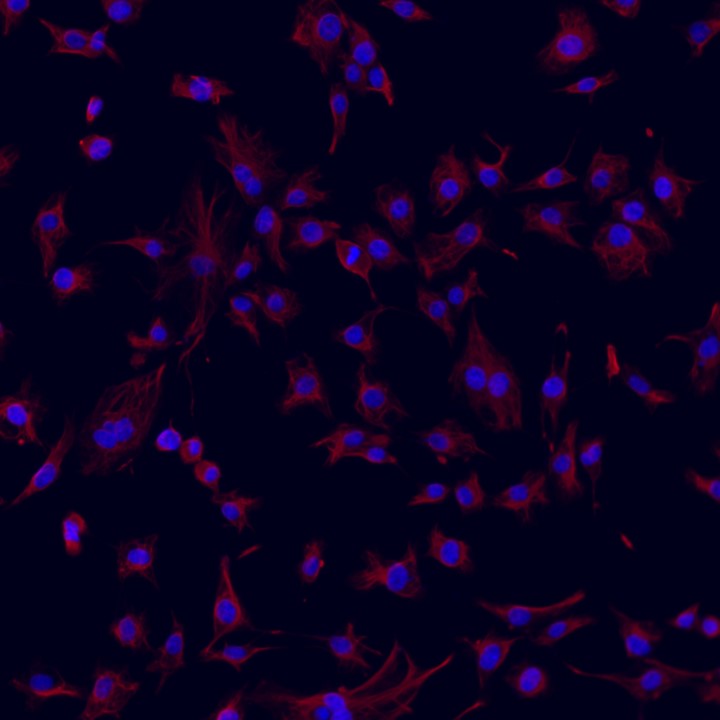

Supplement: Supplementary file 3 [file DataSheet2.zip › Original images and results for Figure 7/Fig. 7E/Fig. 7E Vimentin-IF/Vimentin-IF-ADR3-2.jpg]

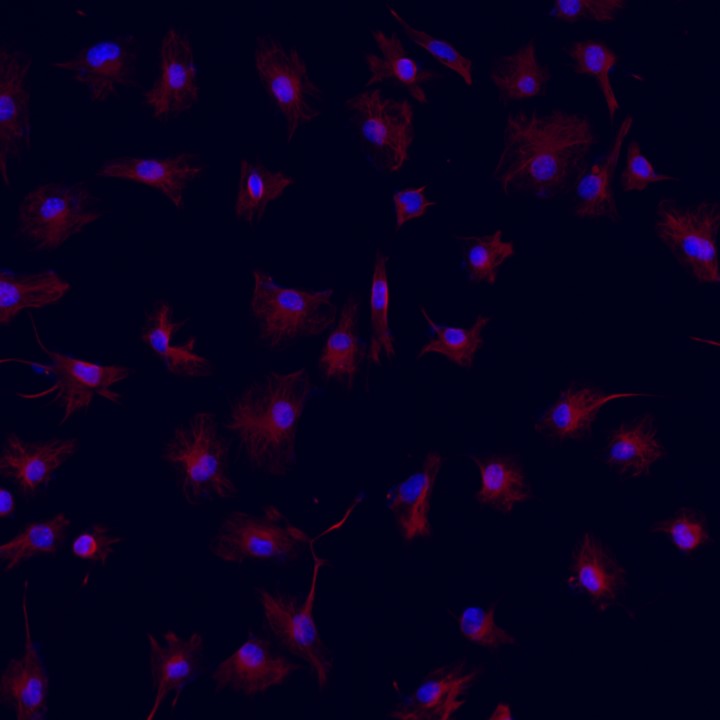

Supplement: Supplementary file 3 [file DataSheet2.zip › Original images and results for Figure 7/Fig. 7E/Fig. 7E Vimentin-IF/Vimentin-IF-ADR3-3.jpg]

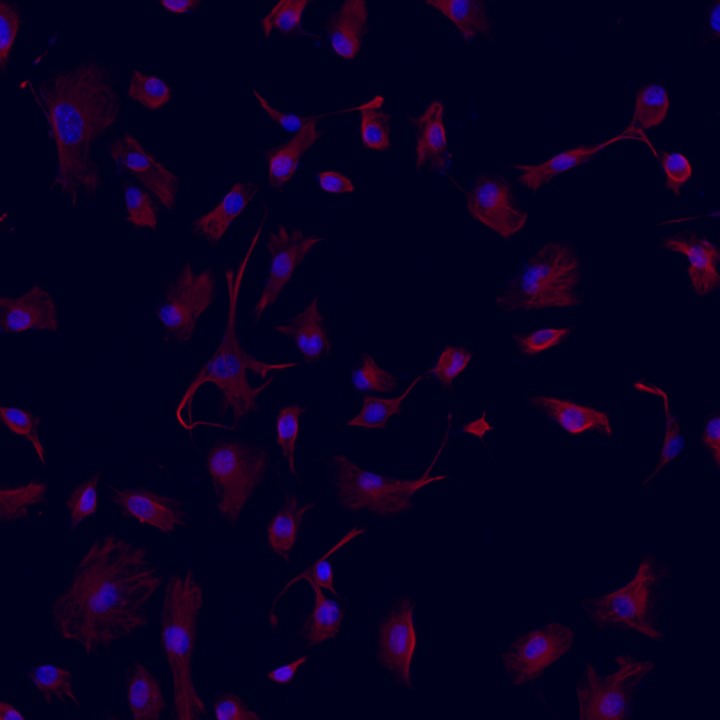

Supplement: Supplementary file 3 [file DataSheet2.zip › Original images and results for Figure 7/Fig. 7E/Fig. 7E Vimentin-IF/Vimentin-IF-ADR3-4.jpg]

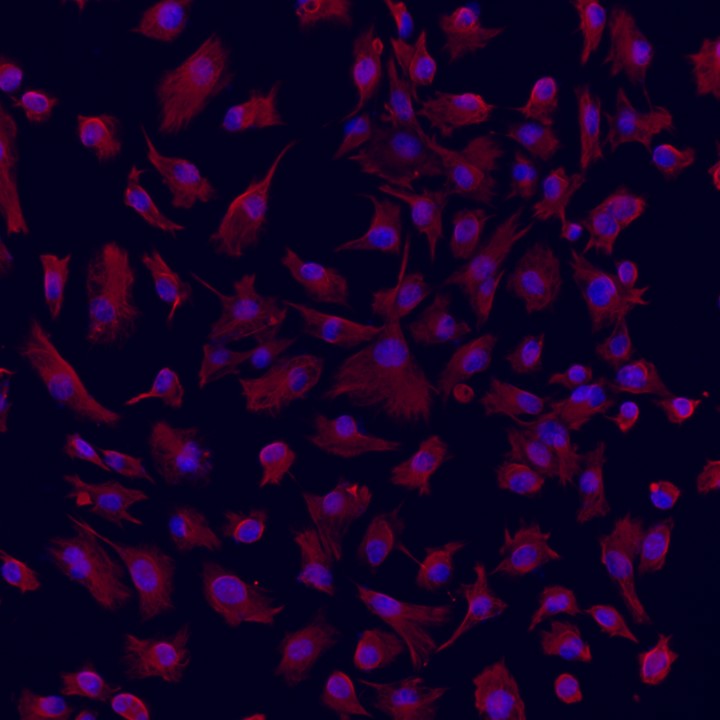

Supplement: Supplementary file 3 [file DataSheet2.zip › Original images and results for Figure 7/Fig. 7E/Fig. 7E Vimentin-IF/Vimentin-IF-ADR3-5.jpg]

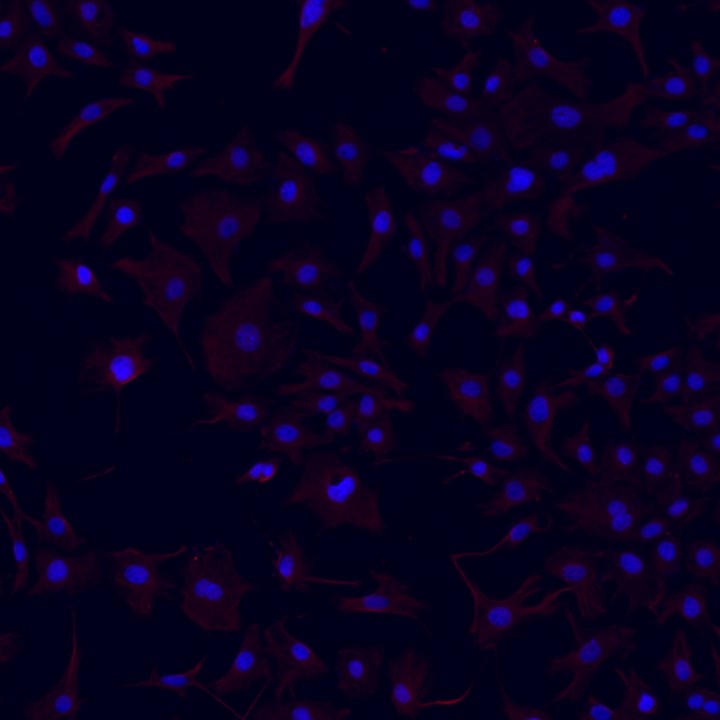

Supplement: Supplementary file 3 [file DataSheet2.zip › Original images and results for Figure 7/Fig. 7E/Fig. 7E Vimentin-IF/Vimentin-IF-CON1-1.jpg]

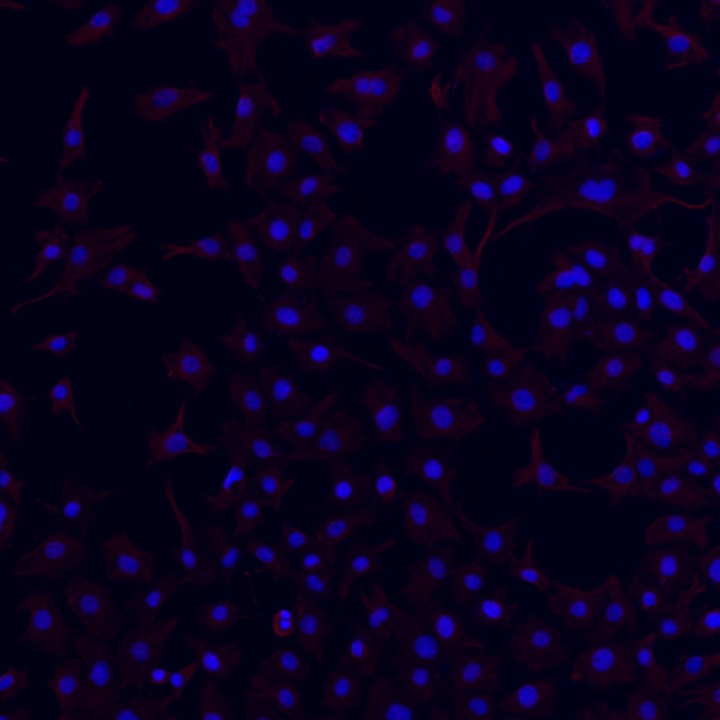

Supplement: Supplementary file 3 [file DataSheet2.zip › Original images and results for Figure 7/Fig. 7E/Fig. 7E Vimentin-IF/Vimentin-IF-CON1-2.jpg]

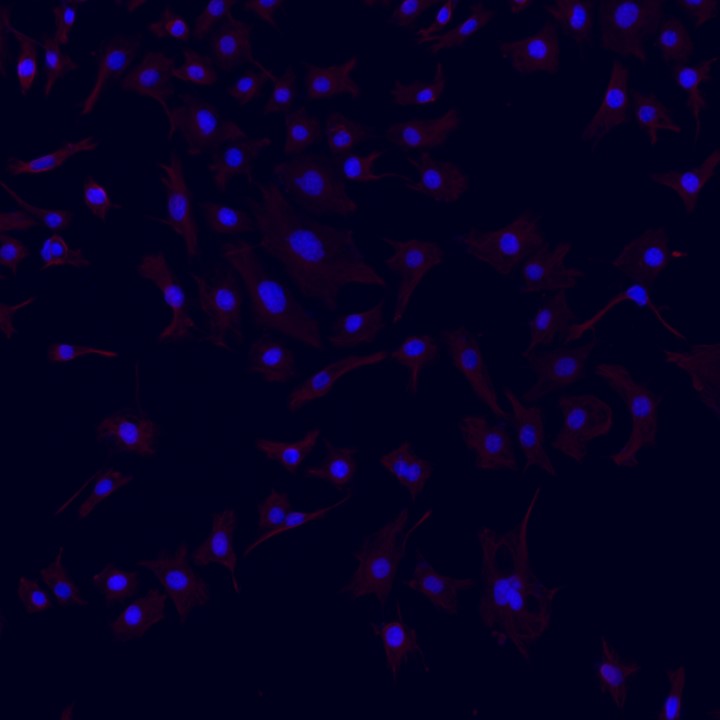

Supplement: Supplementary file 3 [file DataSheet2.zip › Original images and results for Figure 7/Fig. 7E/Fig. 7E Vimentin-IF/Vimentin-IF-CON1-3.jpg]

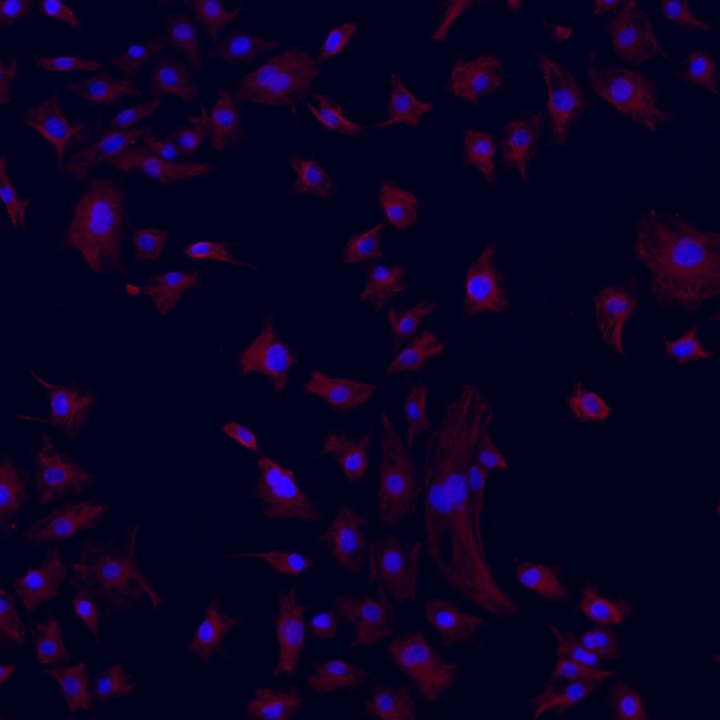

Supplement: Supplementary file 3 [file DataSheet2.zip › Original images and results for Figure 7/Fig. 7E/Fig. 7E Vimentin-IF/Vimentin-IF-CON1-4.jpg]

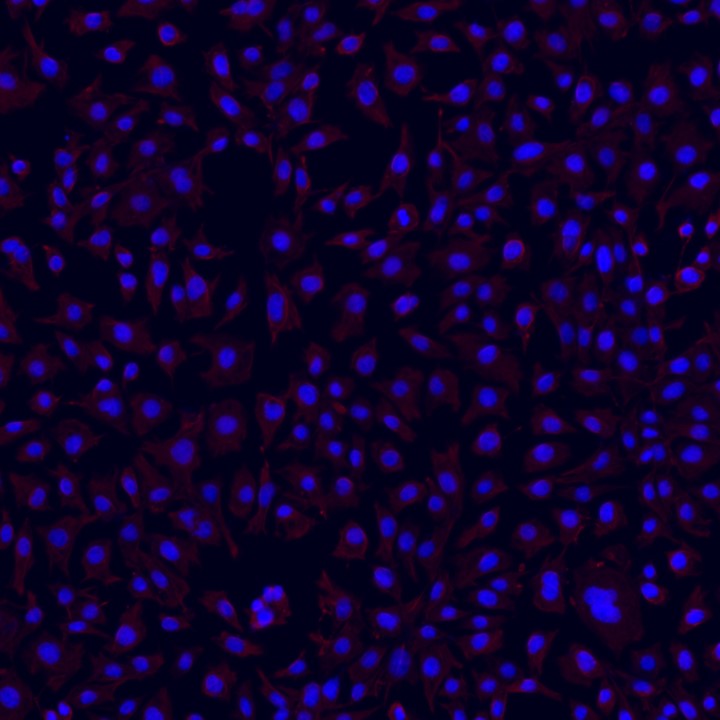

Supplement: Supplementary file 3 [file DataSheet2.zip › Original images and results for Figure 7/Fig. 7E/Fig. 7E Vimentin-IF/Vimentin-IF-CON1-5.jpg]

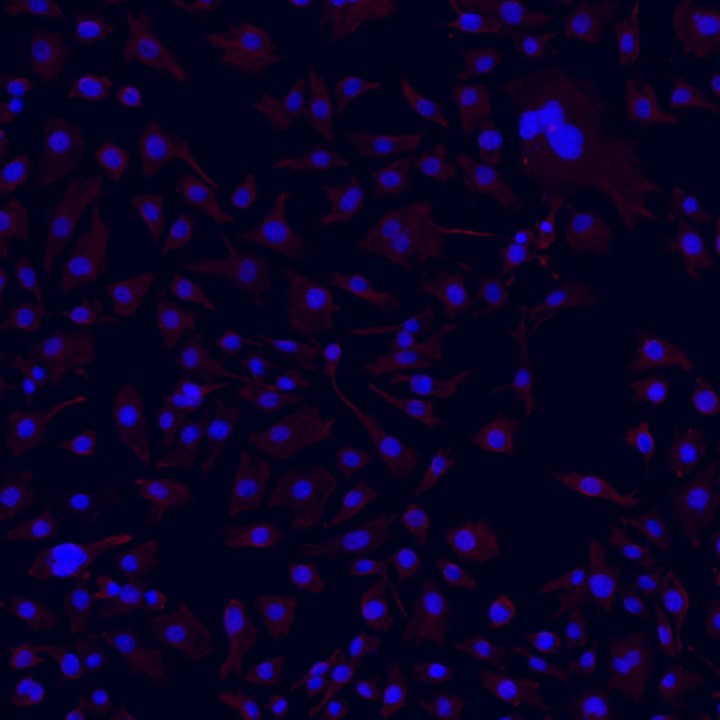

Supplement: Supplementary file 3 [file DataSheet2.zip › Original images and results for Figure 7/Fig. 7E/Fig. 7E Vimentin-IF/Vimentin-IF-CON2-1.jpg]

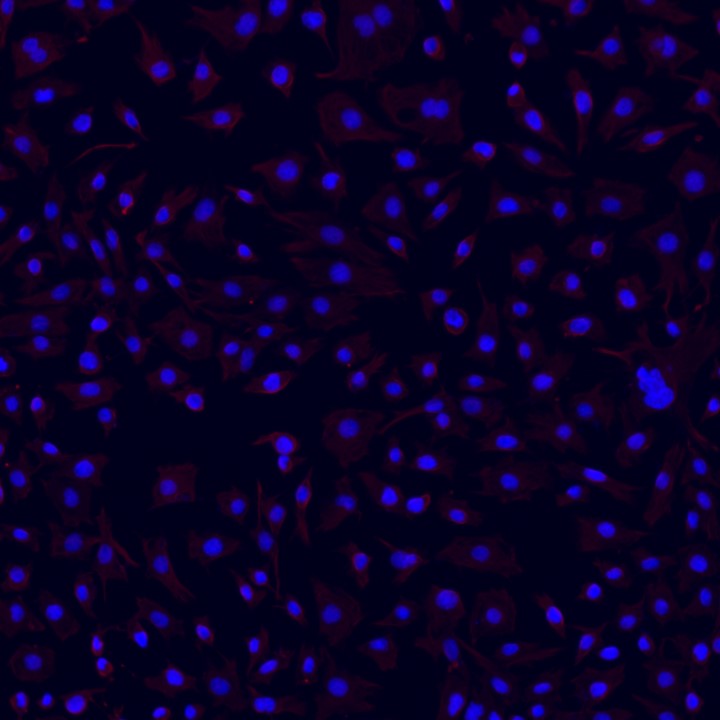

Supplement: Supplementary file 3 [file DataSheet2.zip › Original images and results for Figure 7/Fig. 7E/Fig. 7E Vimentin-IF/Vimentin-IF-CON2-2.jpg]

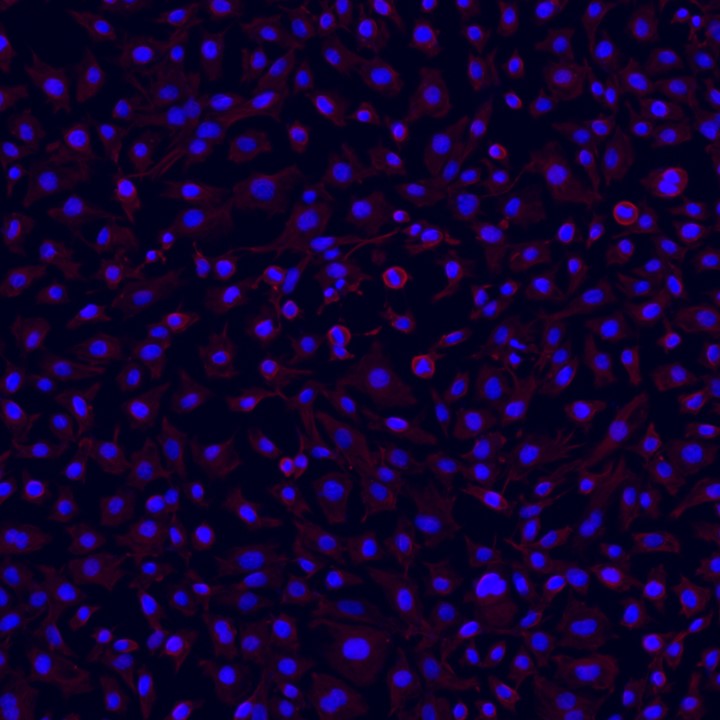

Supplement: Supplementary file 3 [file DataSheet2.zip › Original images and results for Figure 7/Fig. 7E/Fig. 7E Vimentin-IF/Vimentin-IF-CON2-3.jpg]

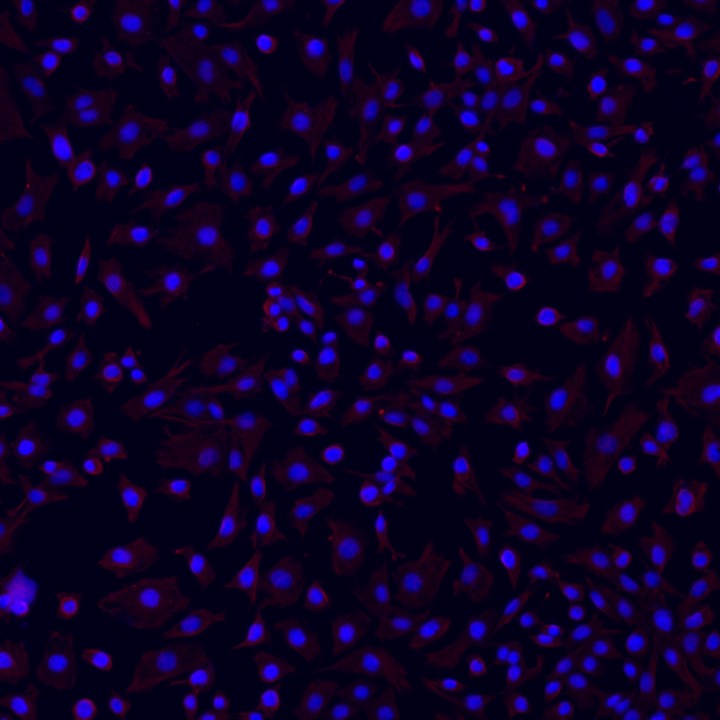

Supplement: Supplementary file 3 [file DataSheet2.zip › Original images and results for Figure 7/Fig. 7E/Fig. 7E Vimentin-IF/Vimentin-IF-CON2-4 image in Fig. 7E.jpg]

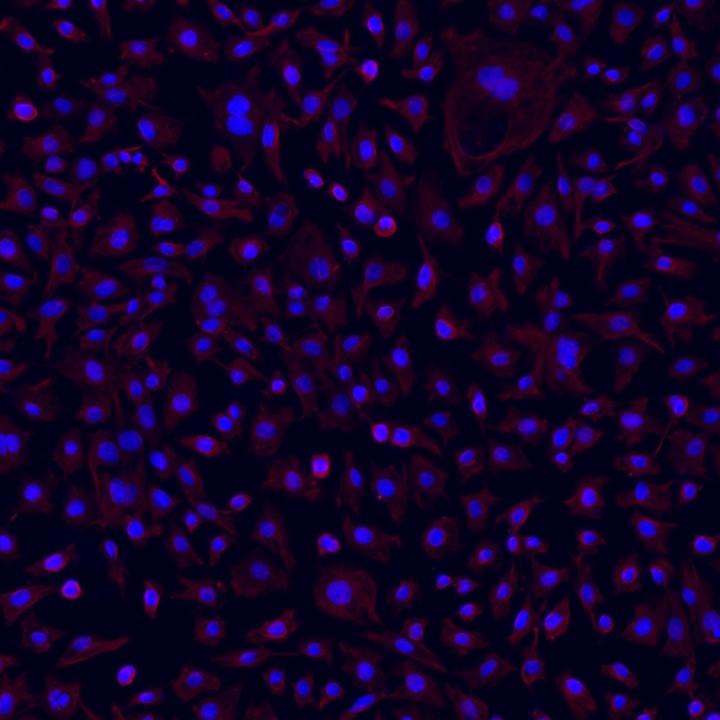

Supplement: Supplementary file 3 [file DataSheet2.zip › Original images and results for Figure 7/Fig. 7E/Fig. 7E Vimentin-IF/Vimentin-IF-CON2-5.jpg]

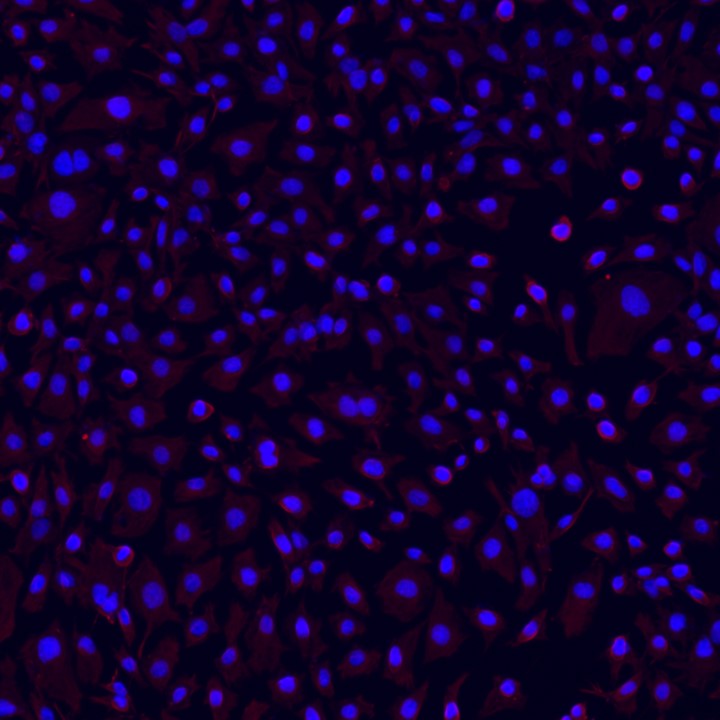

Supplement: Supplementary file 3 [file DataSheet2.zip › Original images and results for Figure 7/Fig. 7E/Fig. 7E Vimentin-IF/Vimentin-IF-CON3-1.jpg]

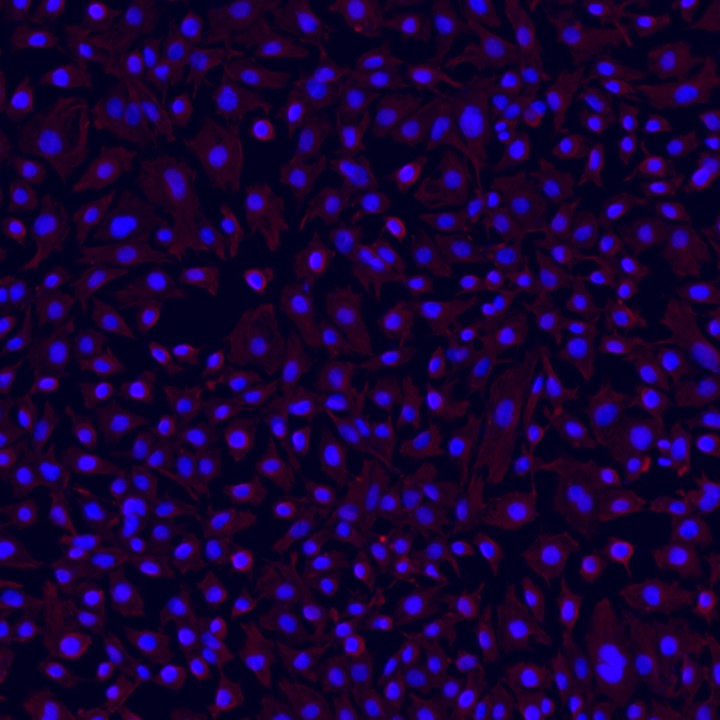

Supplement: Supplementary file 3 [file DataSheet2.zip › Original images and results for Figure 7/Fig. 7E/Fig. 7E Vimentin-IF/Vimentin-IF-CON3-2.jpg]

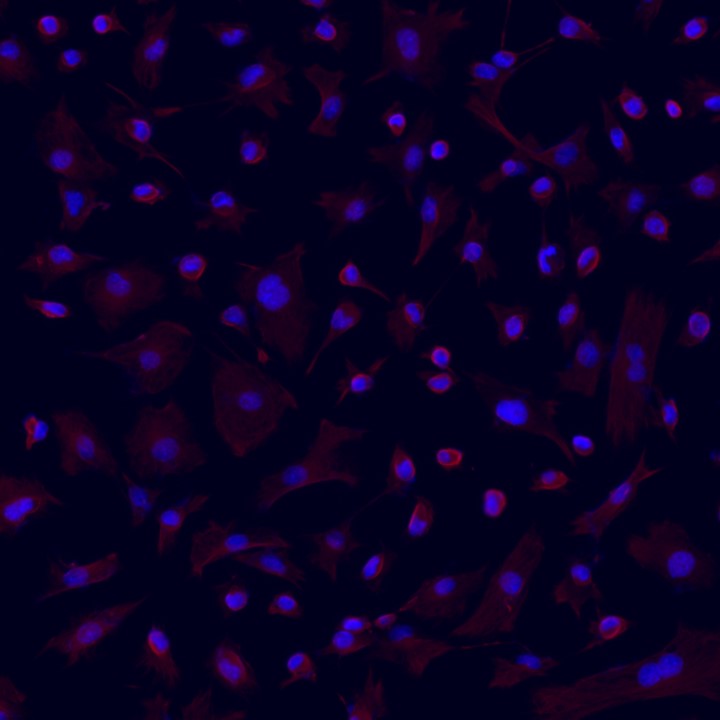

Supplement: Supplementary file 3 [file DataSheet2.zip › Original images and results for Figure 7/Fig. 7E/Fig. 7E Vimentin-IF/Vimentin-IF-CON3-3.jpg]

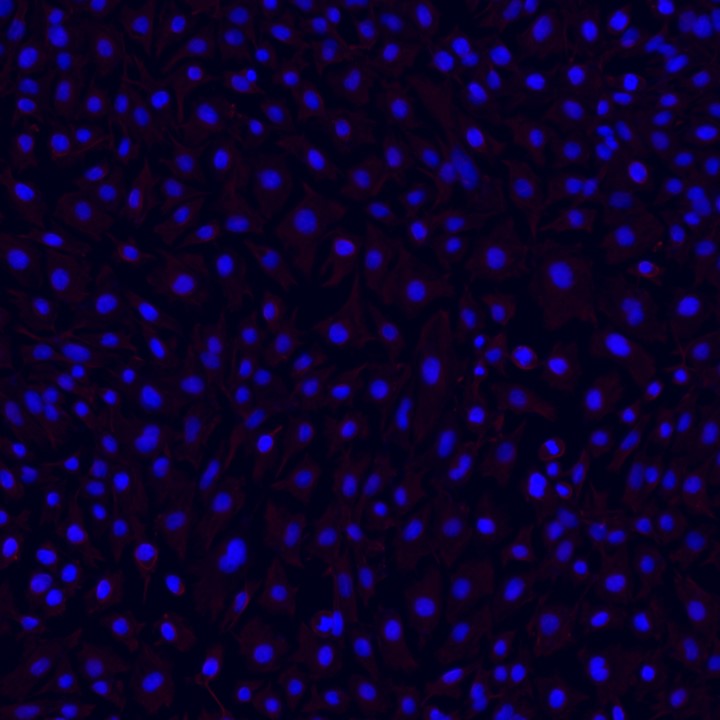

Supplement: Supplementary file 3 [file DataSheet2.zip › Original images and results for Figure 7/Fig. 7E/Fig. 7E Vimentin-IF/Vimentin-IF-CON3-5.jpg]

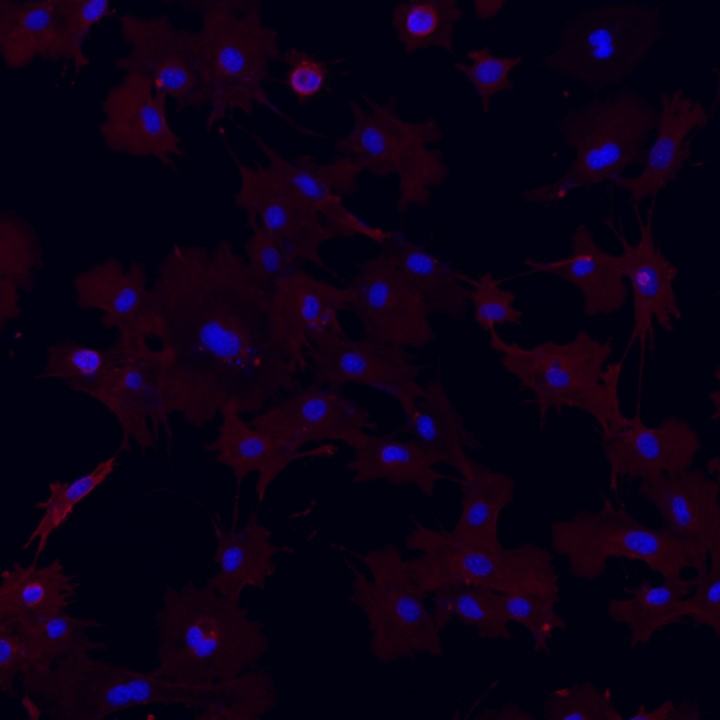

Supplement: Supplementary file 3 [file DataSheet2.zip › Original images and results for Figure 7/Fig. 7E/Fig. 7E α-SMA-IF/α-SMA-IF-ADR+SQL1-1.jpg]

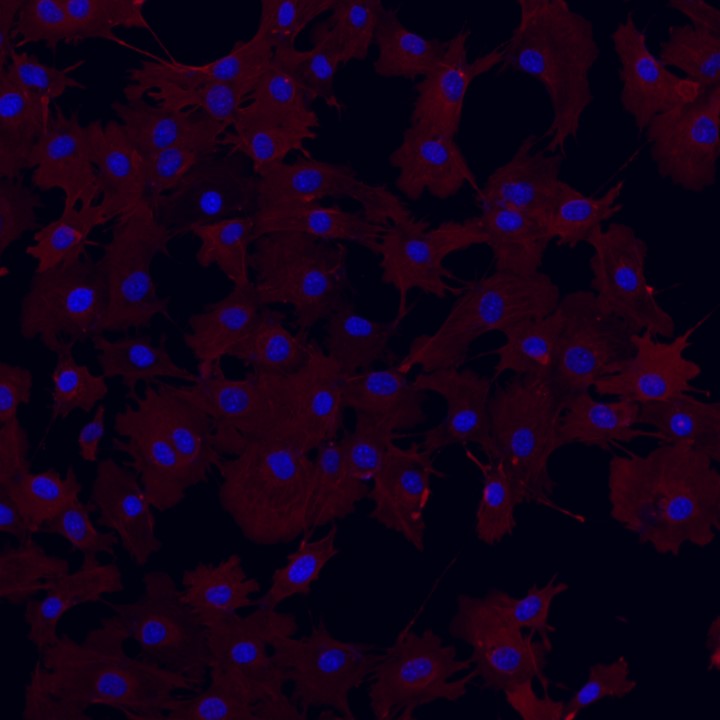

Supplement: Supplementary file 3 [file DataSheet2.zip › Original images and results for Figure 7/Fig. 7E/Fig. 7E α-SMA-IF/α-SMA-IF-ADR+SQL1-2.jpg]

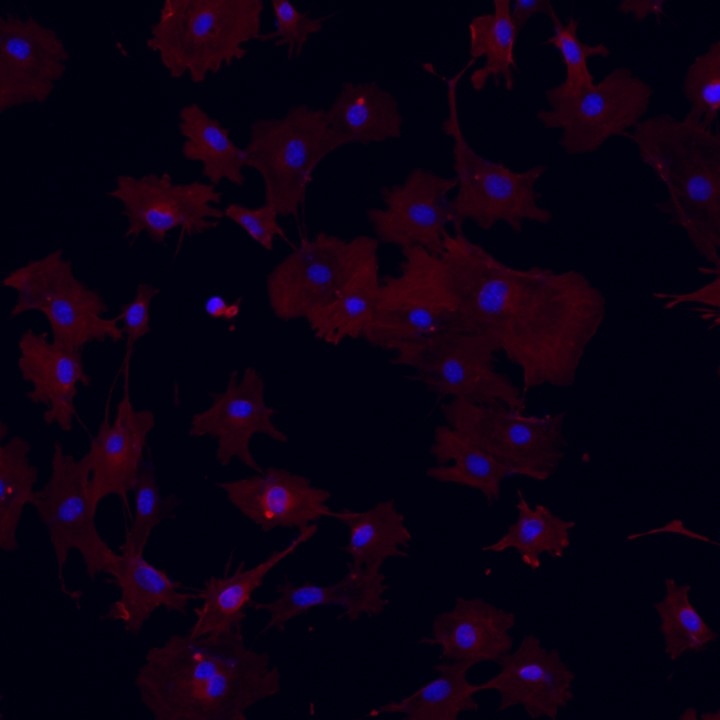

Supplement: Supplementary file 3 [file DataSheet2.zip › Original images and results for Figure 7/Fig. 7E/Fig. 7E α-SMA-IF/α-SMA-IF-ADR+SQL1-3.jpg]

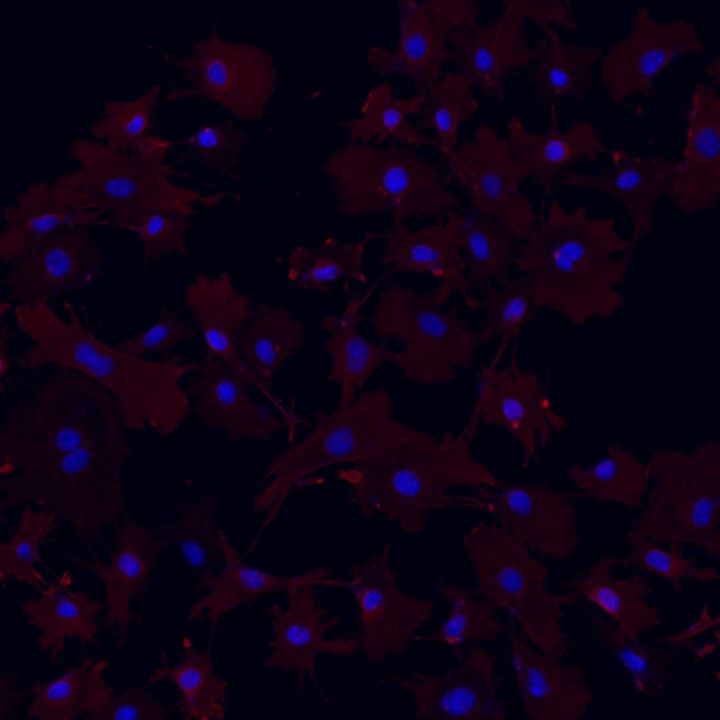

Supplement: Supplementary file 3 [file DataSheet2.zip › Original images and results for Figure 7/Fig. 7E/Fig. 7E α-SMA-IF/α-SMA-IF-ADR+SQL1-4.jpg]

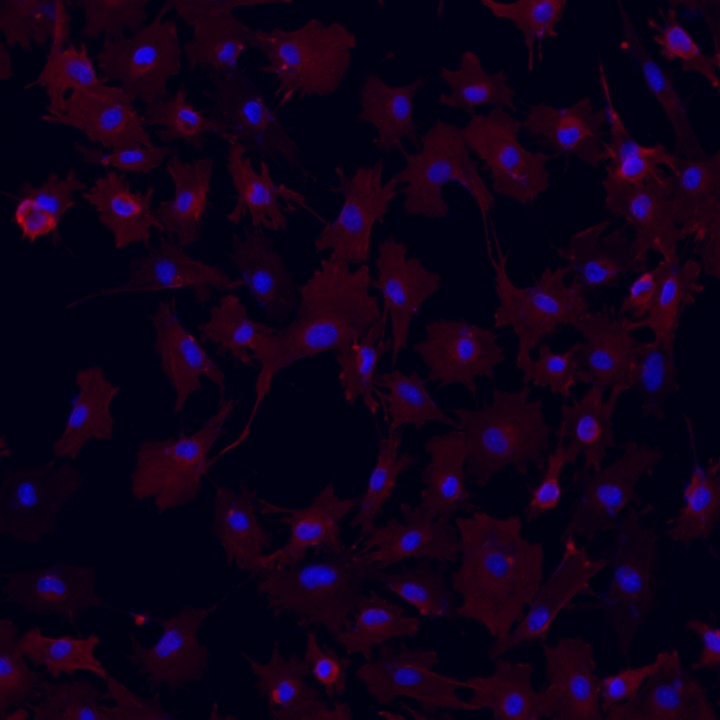

Supplement: Supplementary file 3 [file DataSheet2.zip › Original images and results for Figure 7/Fig. 7E/Fig. 7E α-SMA-IF/α-SMA-IF-ADR+SQL1-5.jpg]

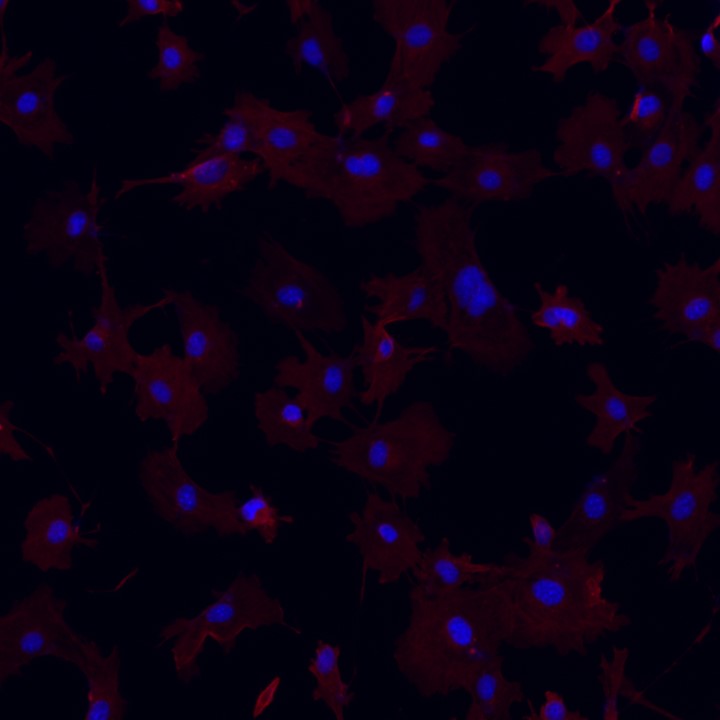

Supplement: Supplementary file 3 [file DataSheet2.zip › Original images and results for Figure 7/Fig. 7E/Fig. 7E α-SMA-IF/α-SMA-IF-ADR+SQL2-1.jpg]

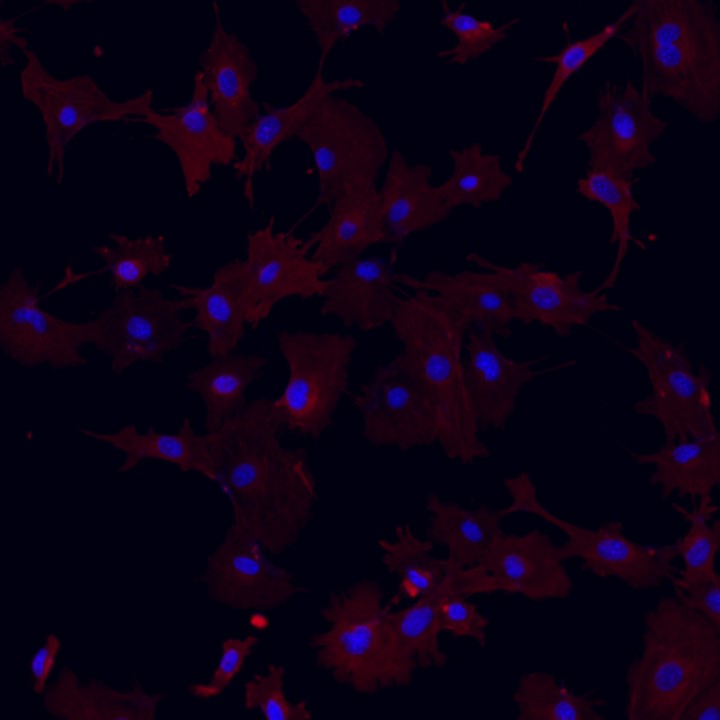

Supplement: Supplementary file 3 [file DataSheet2.zip › Original images and results for Figure 7/Fig. 7E/Fig. 7E α-SMA-IF/α-SMA-IF-ADR+SQL2-2.jpg]

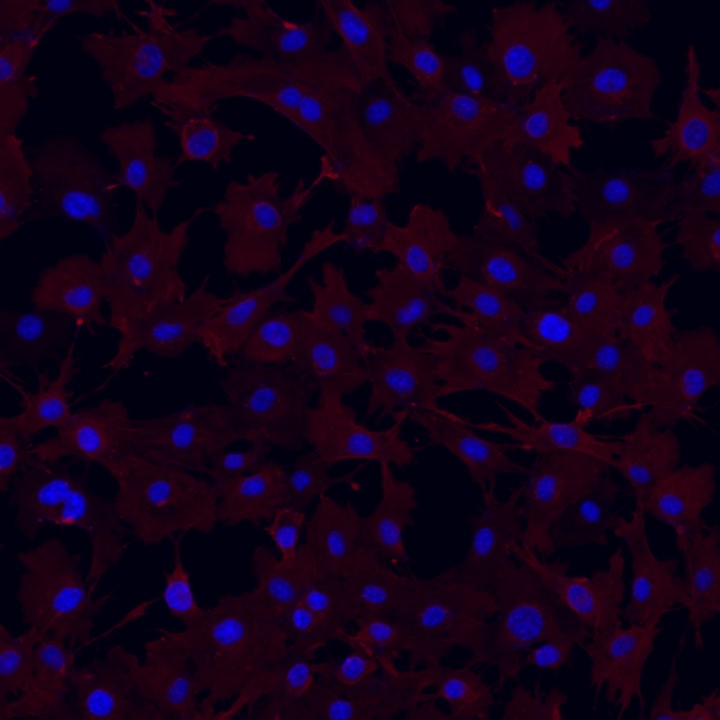

Supplement: Supplementary file 3 [file DataSheet2.zip › Original images and results for Figure 7/Fig. 7E/Fig. 7E α-SMA-IF/α-SMA-IF-ADR+SQL2-3.jpg]

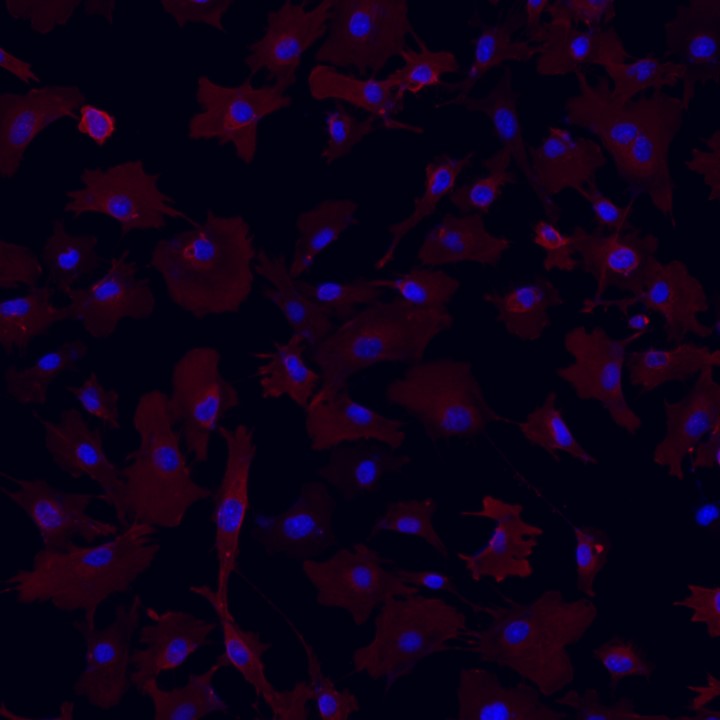

Supplement: Supplementary file 3 [file DataSheet2.zip › Original images and results for Figure 7/Fig. 7E/Fig. 7E α-SMA-IF/α-SMA-IF-ADR+SQL2-4.jpg]

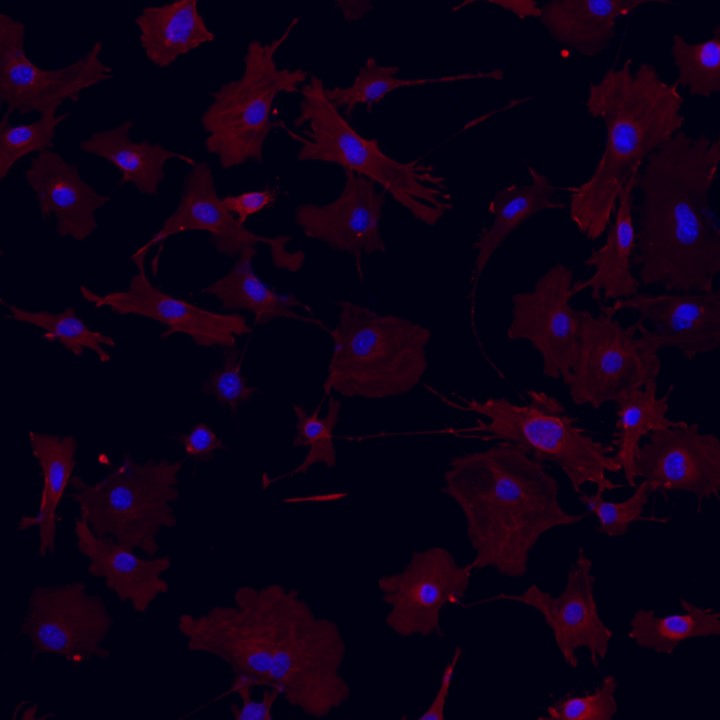

Supplement: Supplementary file 3 [file DataSheet2.zip › Original images and results for Figure 7/Fig. 7E/Fig. 7E α-SMA-IF/α-SMA-IF-ADR+SQL2-5.jpg]

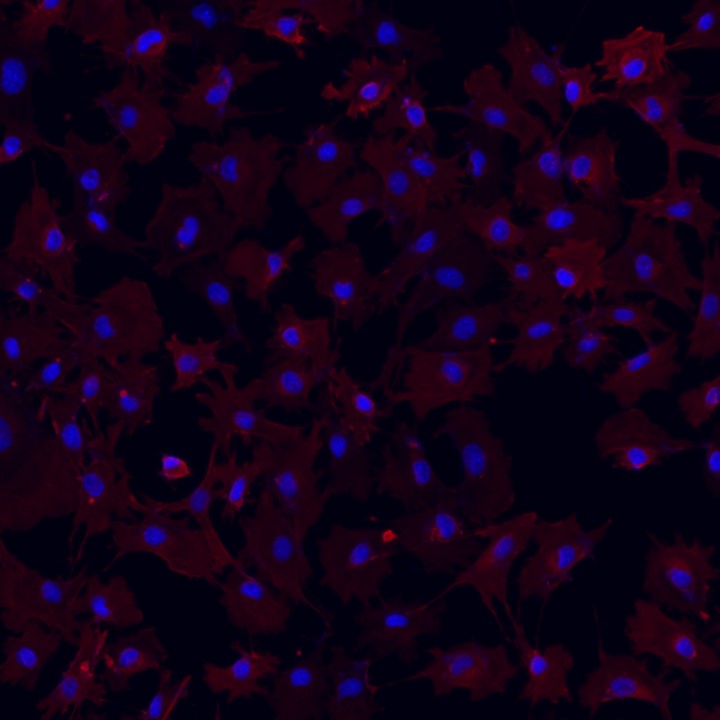

Supplement: Supplementary file 3 [file DataSheet2.zip › Original images and results for Figure 7/Fig. 7E/Fig. 7E α-SMA-IF/α-SMA-IF-ADR+SQL3-1 image in Fig. 7E.jpg]

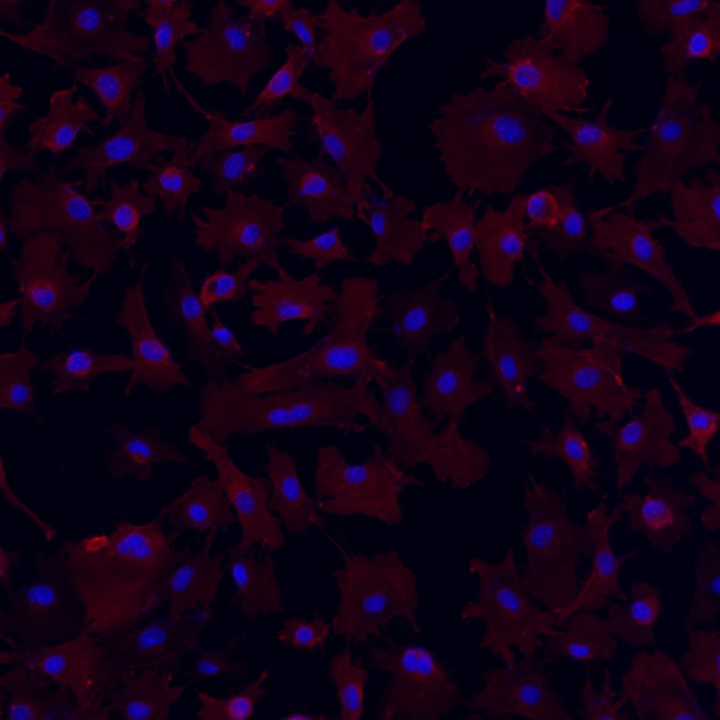

Supplement: Supplementary file 3 [file DataSheet2.zip › Original images and results for Figure 7/Fig. 7E/Fig. 7E α-SMA-IF/α-SMA-IF-ADR+SQL3-2.jpg]

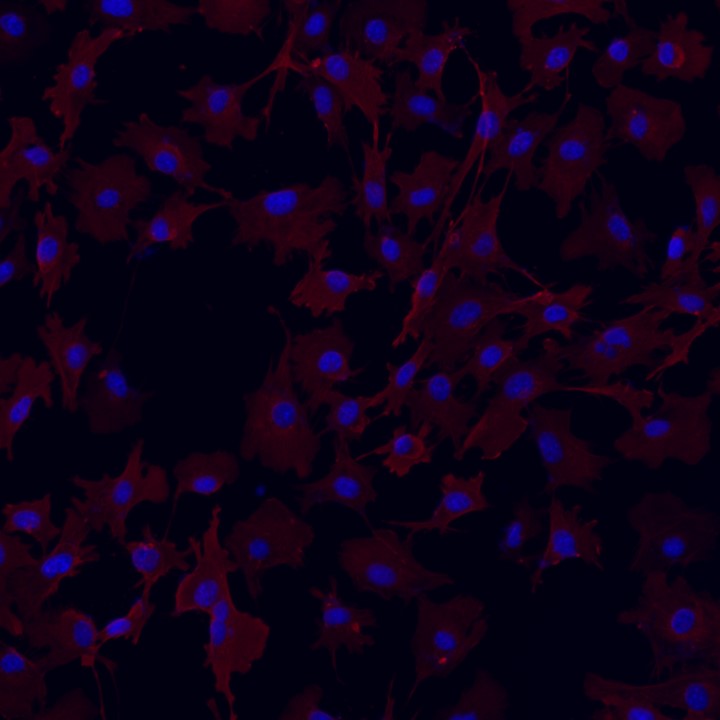

Supplement: Supplementary file 3 [file DataSheet2.zip › Original images and results for Figure 7/Fig. 7E/Fig. 7E α-SMA-IF/α-SMA-IF-ADR+SQL3-3.jpg]

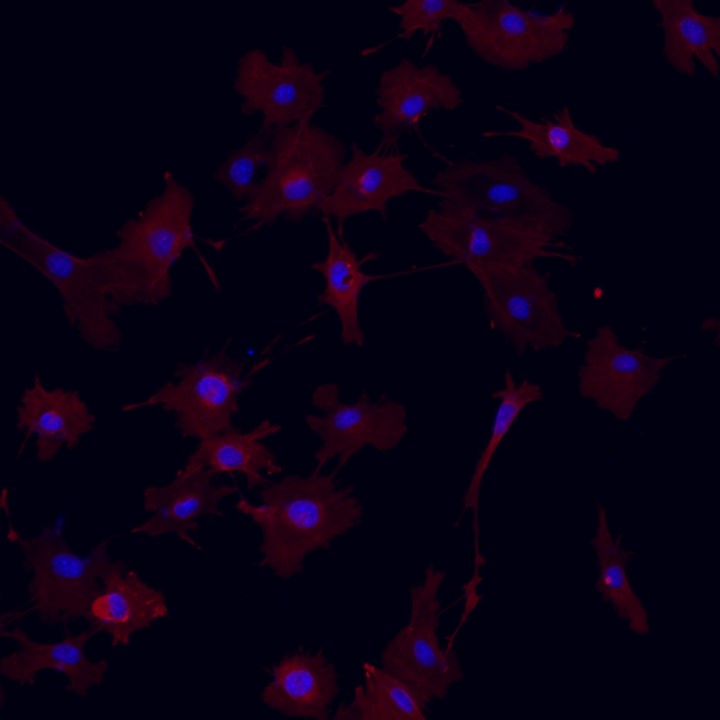

Supplement: Supplementary file 3 [file DataSheet2.zip › Original images and results for Figure 7/Fig. 7E/Fig. 7E α-SMA-IF/α-SMA-IF-ADR+SQL3-4.jpg]

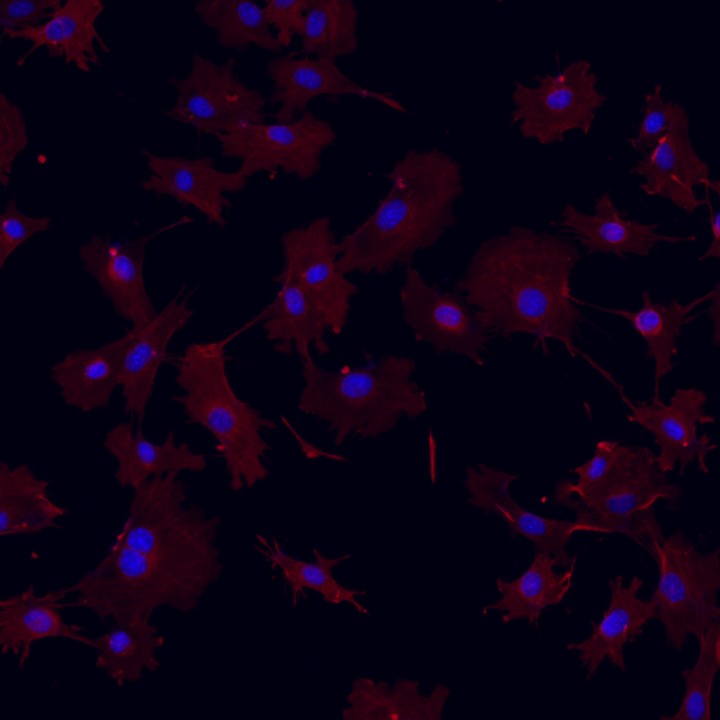

Supplement: Supplementary file 3 [file DataSheet2.zip › Original images and results for Figure 7/Fig. 7E/Fig. 7E α-SMA-IF/α-SMA-IF-ADR+SQL3-5.jpg]

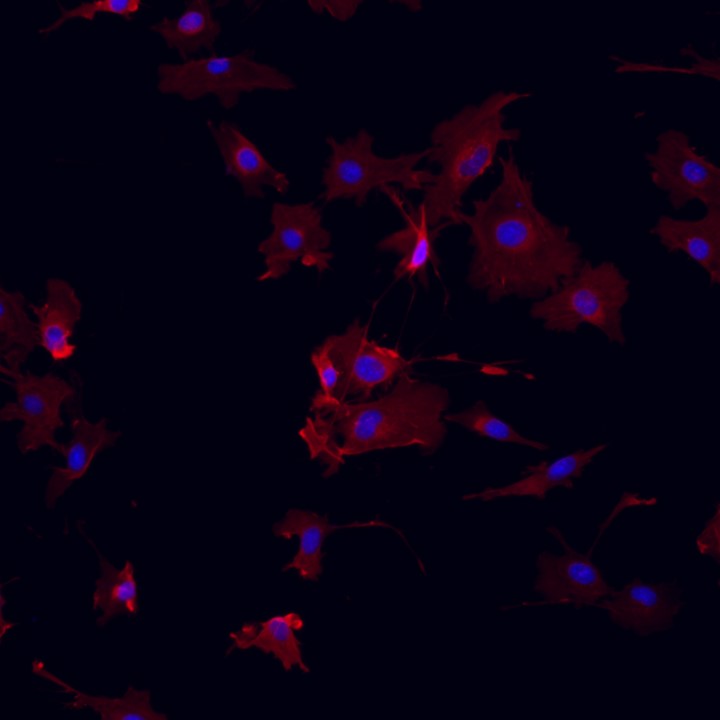

Supplement: Supplementary file 3 [file DataSheet2.zip › Original images and results for Figure 7/Fig. 7E/Fig. 7E α-SMA-IF/α-SMA-IF-ADR1-1.jpg]

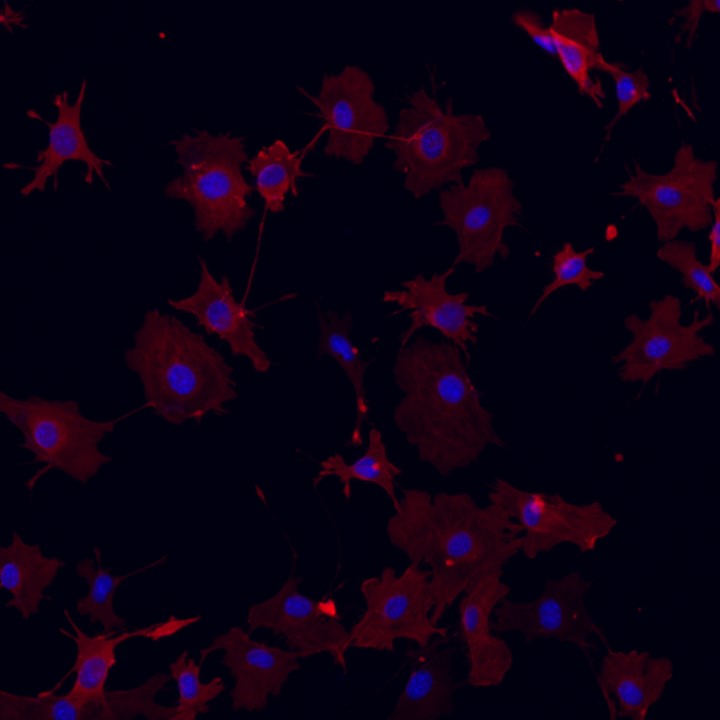

Supplement: Supplementary file 3 [file DataSheet2.zip › Original images and results for Figure 7/Fig. 7E/Fig. 7E α-SMA-IF/α-SMA-IF-ADR1-2.jpg]

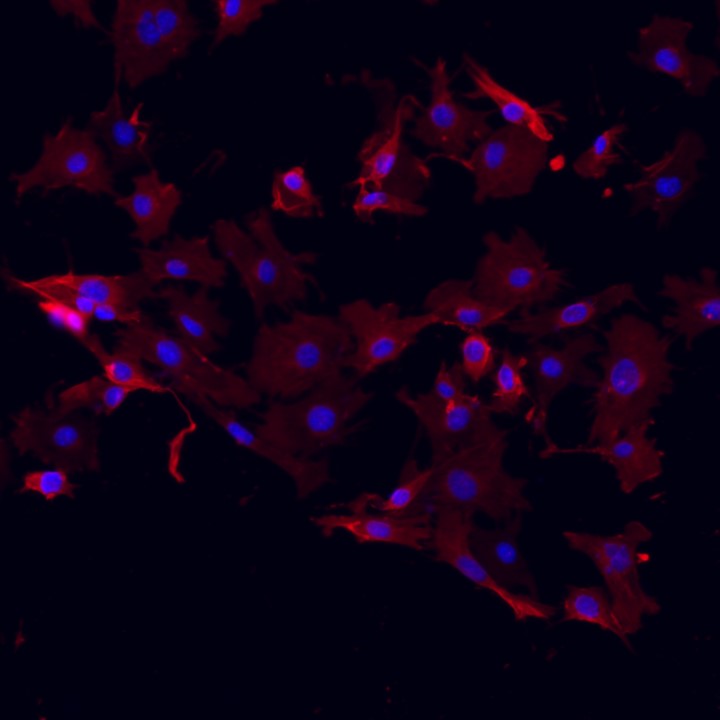

Supplement: Supplementary file 3 [file DataSheet2.zip › Original images and results for Figure 7/Fig. 7E/Fig. 7E α-SMA-IF/α-SMA-IF-ADR1-3 image in Fig. 7E.jpg]

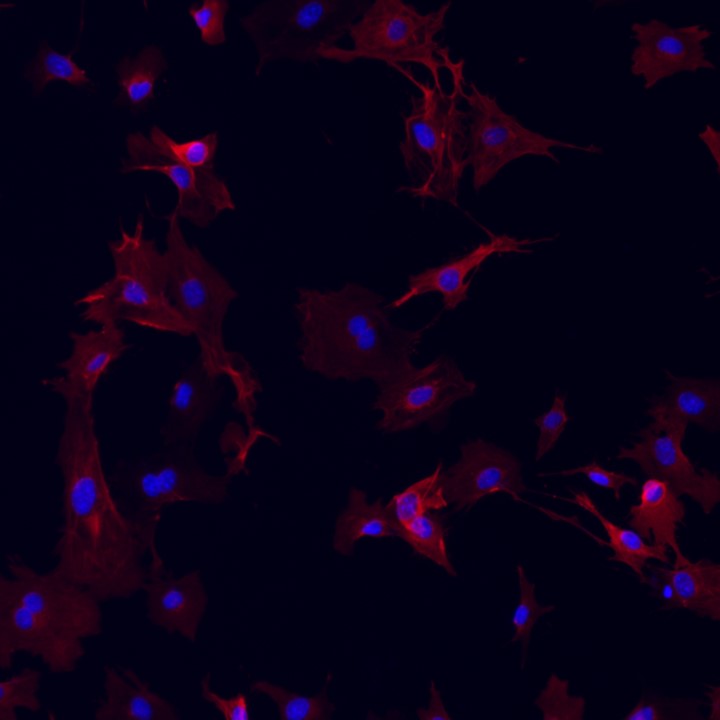

Supplement: Supplementary file 3 [file DataSheet2.zip › Original images and results for Figure 7/Fig. 7E/Fig. 7E α-SMA-IF/α-SMA-IF-ADR1-4.jpg]

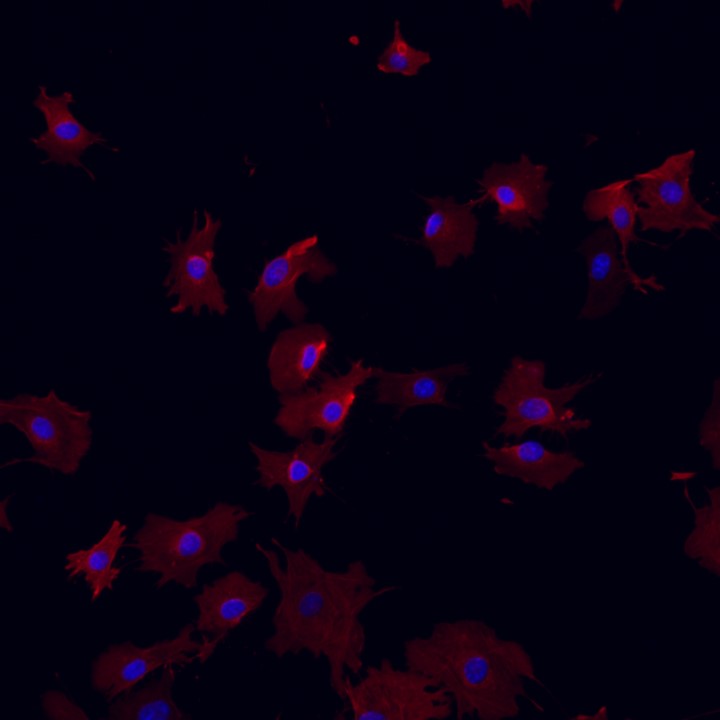

Supplement: Supplementary file 3 [file DataSheet2.zip › Original images and results for Figure 7/Fig. 7E/Fig. 7E α-SMA-IF/α-SMA-IF-ADR1-5.jpg]

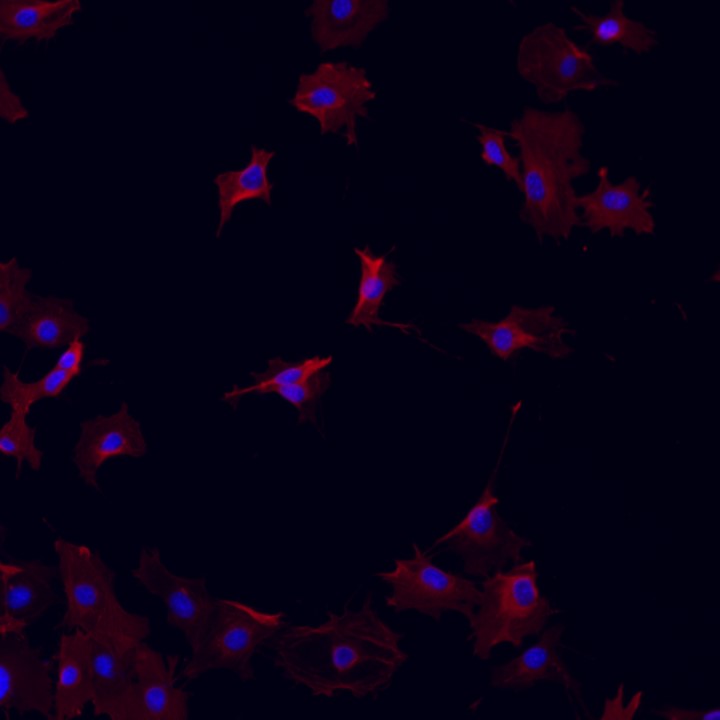

Supplement: Supplementary file 3 [file DataSheet2.zip › Original images and results for Figure 7/Fig. 7E/Fig. 7E α-SMA-IF/α-SMA-IF-ADR2-1.jpg]

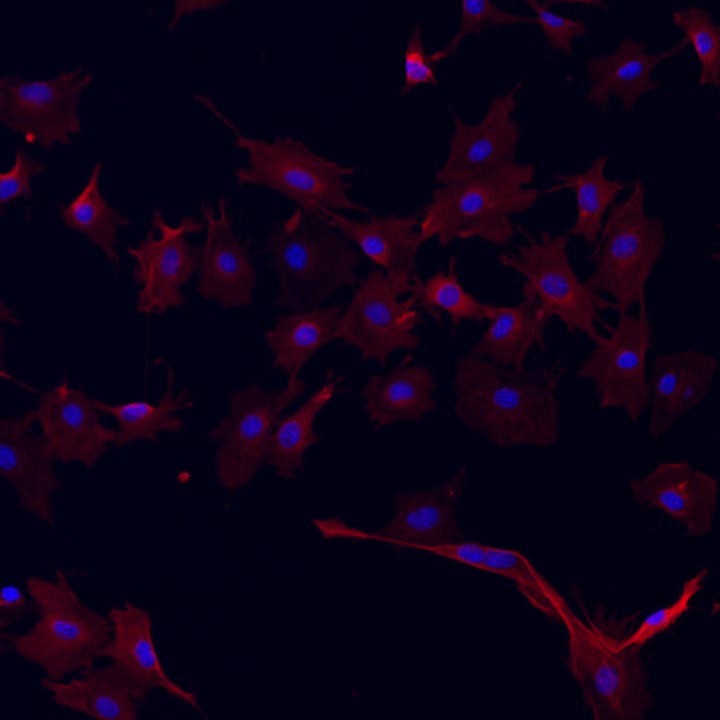

Supplement: Supplementary file 3 [file DataSheet2.zip › Original images and results for Figure 7/Fig. 7E/Fig. 7E α-SMA-IF/α-SMA-IF-ADR2-2.jpg]

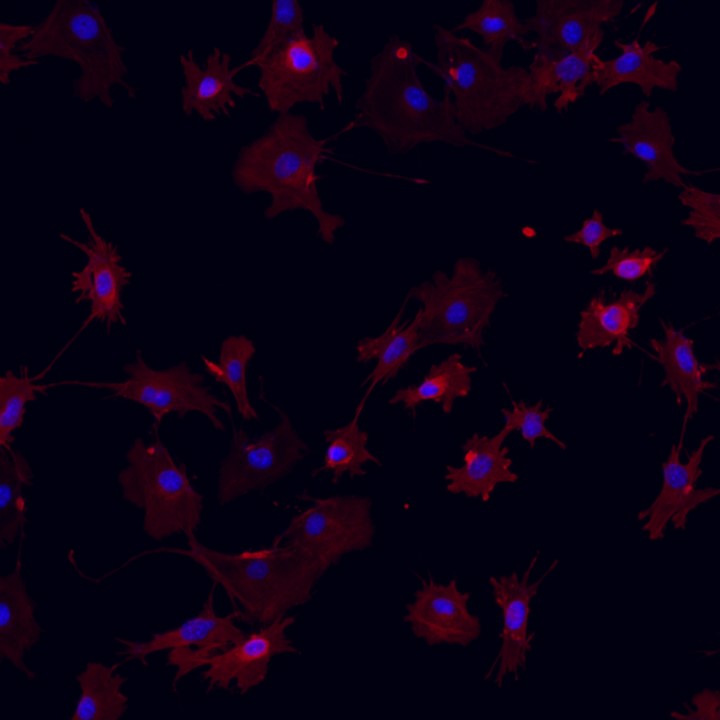

Supplement: Supplementary file 3 [file DataSheet2.zip › Original images and results for Figure 7/Fig. 7E/Fig. 7E α-SMA-IF/α-SMA-IF-ADR2-3.jpg]

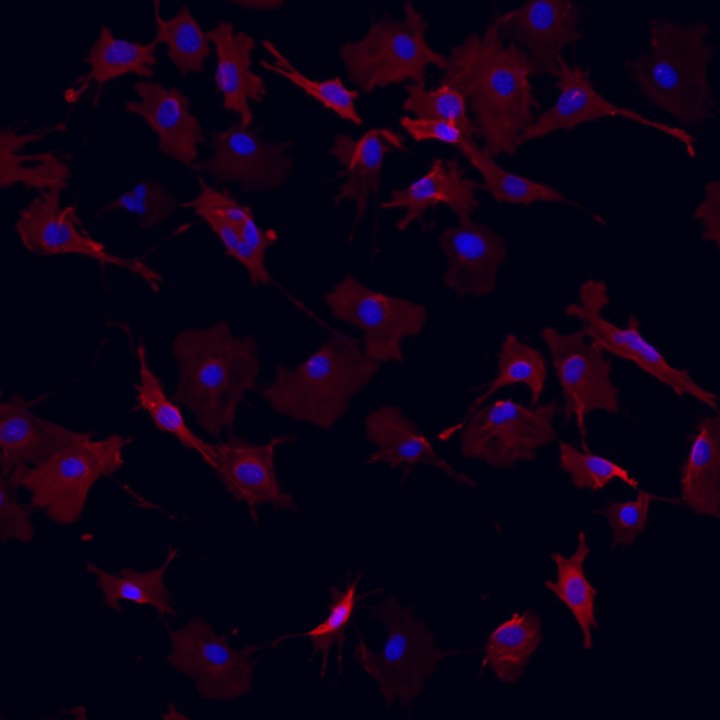

Supplement: Supplementary file 3 [file DataSheet2.zip › Original images and results for Figure 7/Fig. 7E/Fig. 7E α-SMA-IF/α-SMA-IF-ADR2-4.jpg]

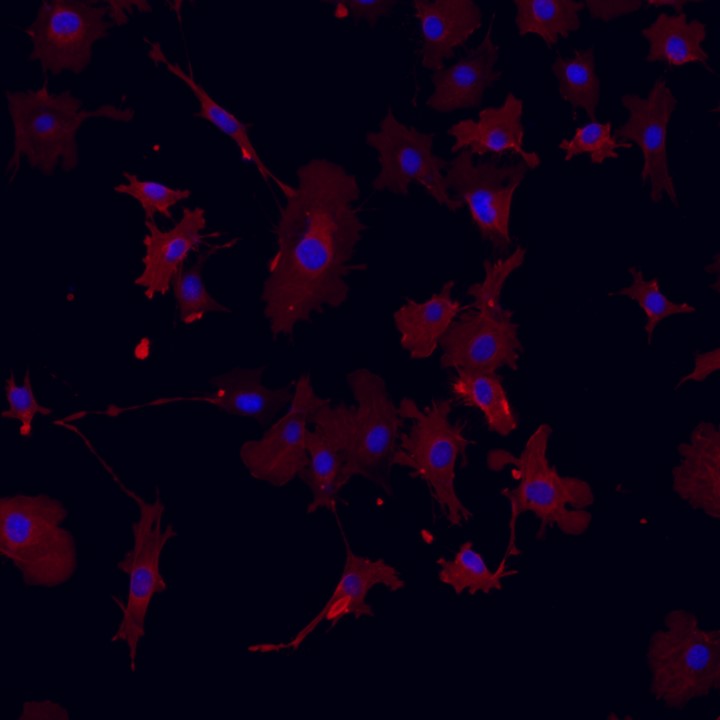

Supplement: Supplementary file 3 [file DataSheet2.zip › Original images and results for Figure 7/Fig. 7E/Fig. 7E α-SMA-IF/α-SMA-IF-ADR2-5.jpg]

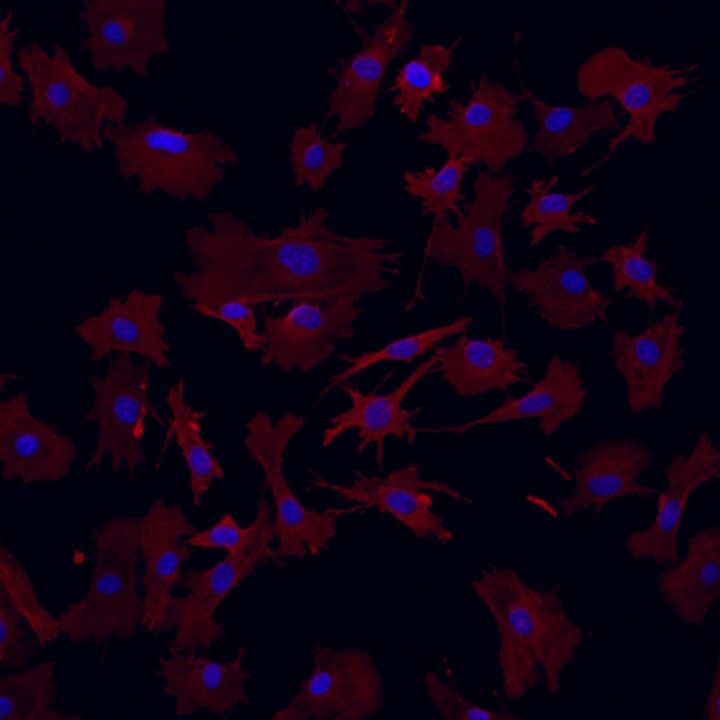

Supplement: Supplementary file 3 [file DataSheet2.zip › Original images and results for Figure 7/Fig. 7E/Fig. 7E α-SMA-IF/α-SMA-IF-ADR3-1.jpg]

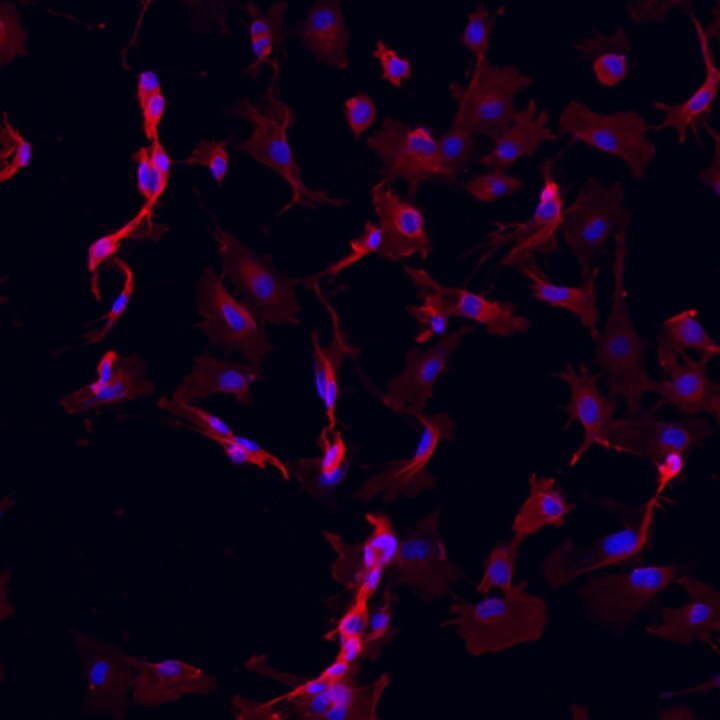

Supplement: Supplementary file 3 [file DataSheet2.zip › Original images and results for Figure 7/Fig. 7E/Fig. 7E α-SMA-IF/α-SMA-IF-ADR3-2.jpg]

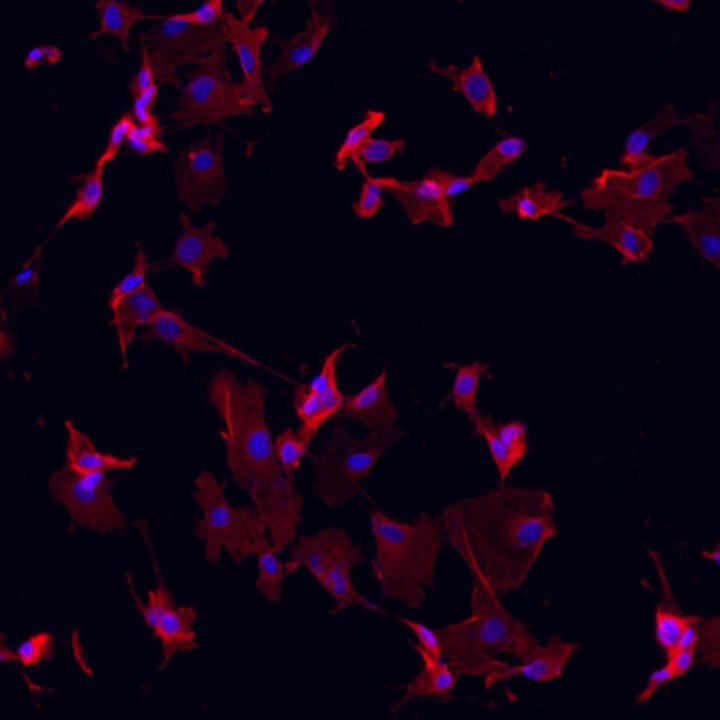

Supplement: Supplementary file 3 [file DataSheet2.zip › Original images and results for Figure 7/Fig. 7E/Fig. 7E α-SMA-IF/α-SMA-IF-ADR3-3.jpg]

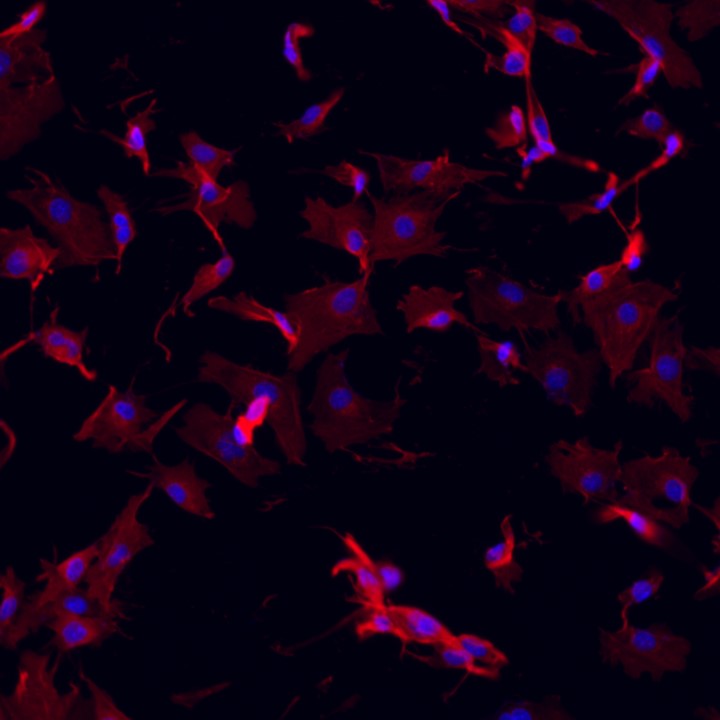

Supplement: Supplementary file 3 [file DataSheet2.zip › Original images and results for Figure 7/Fig. 7E/Fig. 7E α-SMA-IF/α-SMA-IF-ADR3-4.jpg]

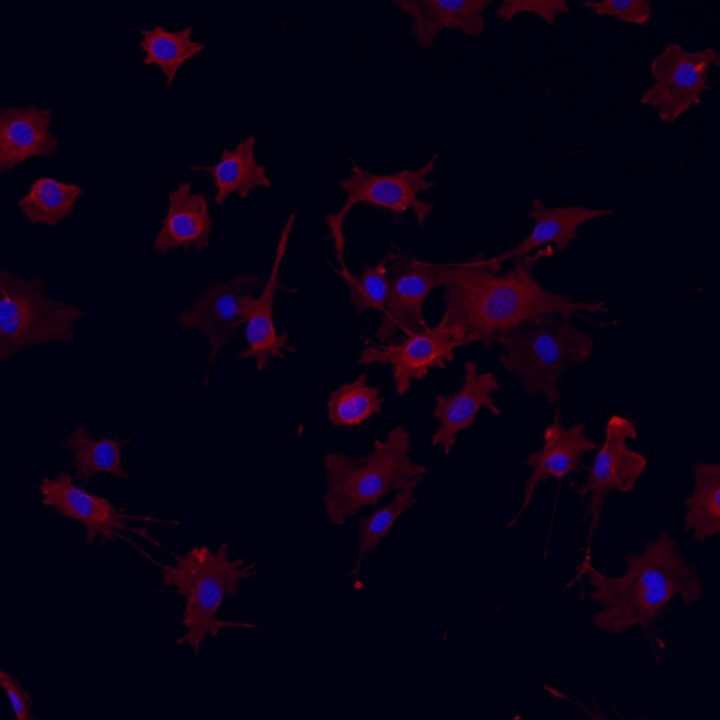

Supplement: Supplementary file 3 [file DataSheet2.zip › Original images and results for Figure 7/Fig. 7E/Fig. 7E α-SMA-IF/α-SMA-IF-ADR3-5.jpg]

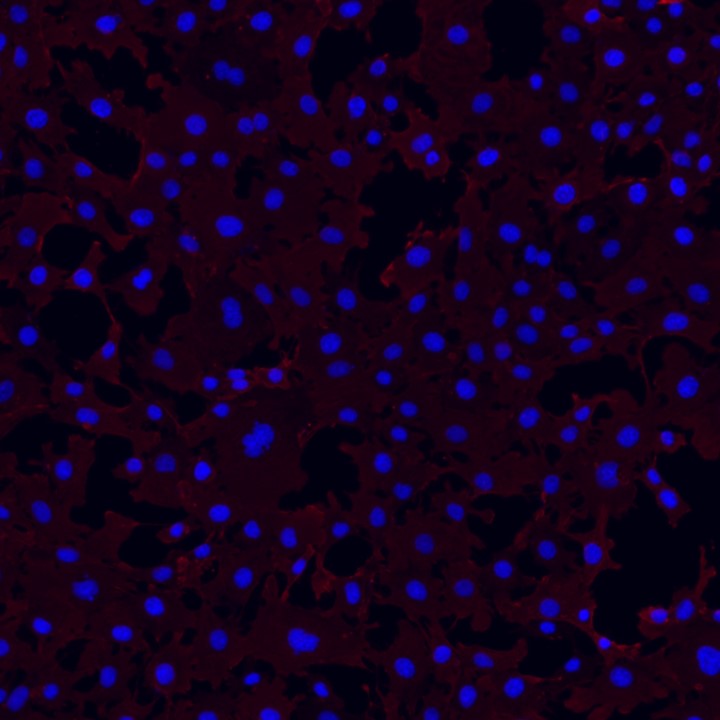

Supplement: Supplementary file 3 [file DataSheet2.zip › Original images and results for Figure 7/Fig. 7E/Fig. 7E α-SMA-IF/α-SMA-IF-CON1-1.jpg]

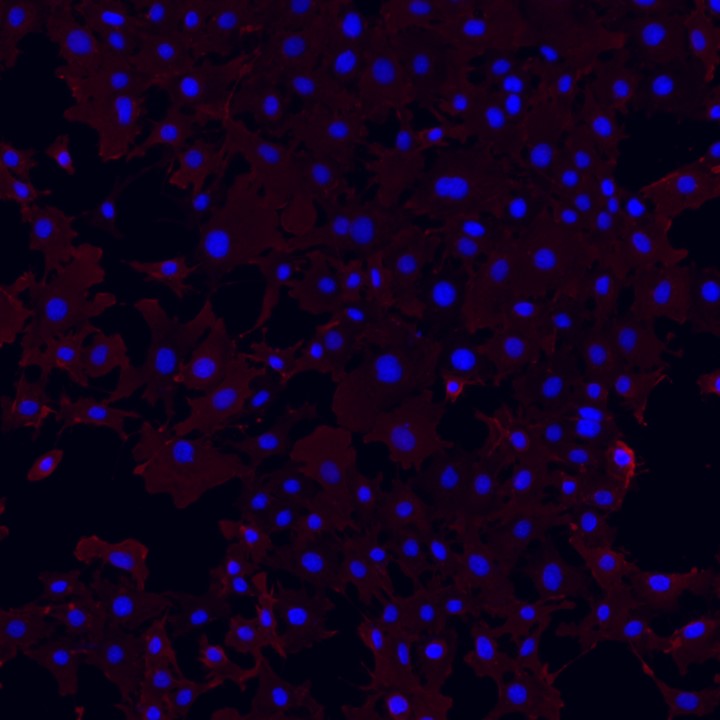

Supplement: Supplementary file 3 [file DataSheet2.zip › Original images and results for Figure 7/Fig. 7E/Fig. 7E α-SMA-IF/α-SMA-IF-CON1-2.jpg]

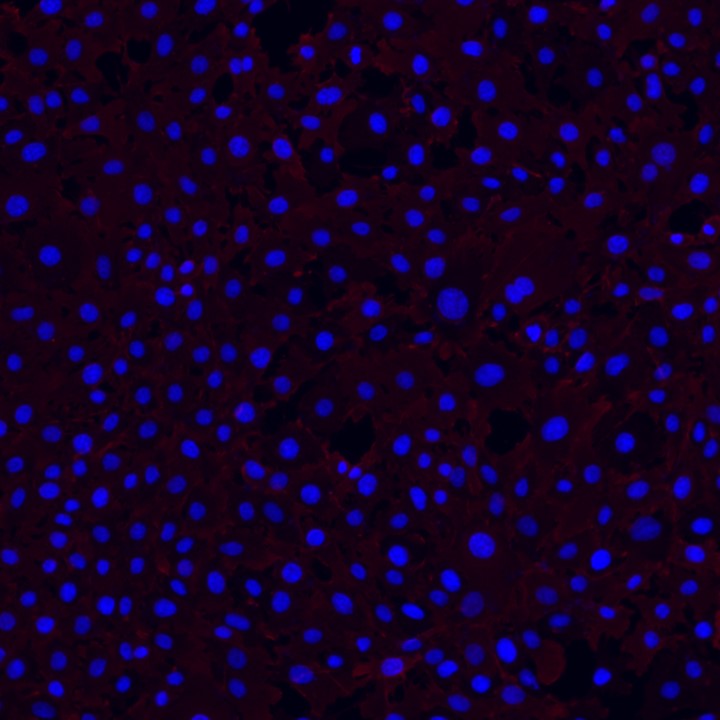

Supplement: Supplementary file 3 [file DataSheet2.zip › Original images and results for Figure 7/Fig. 7E/Fig. 7E α-SMA-IF/α-SMA-IF-CON1-3.jpg]

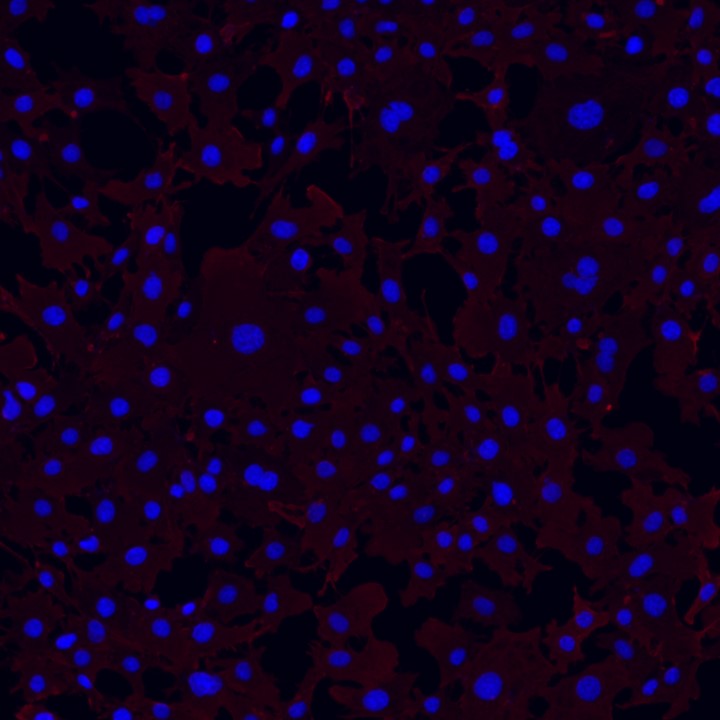

Supplement: Supplementary file 3 [file DataSheet2.zip › Original images and results for Figure 7/Fig. 7E/Fig. 7E α-SMA-IF/α-SMA-IF-CON1-4.jpg]

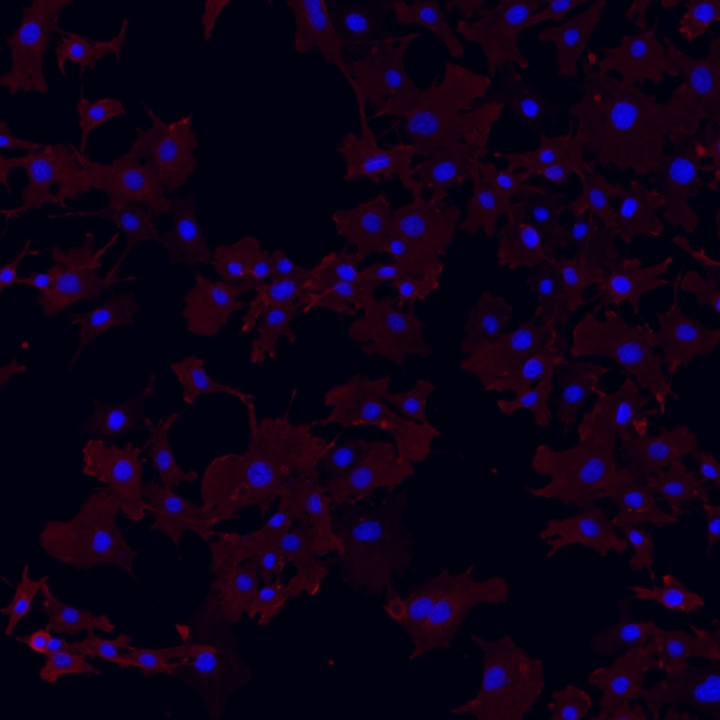

Supplement: Supplementary file 3 [file DataSheet2.zip › Original images and results for Figure 7/Fig. 7E/Fig. 7E α-SMA-IF/α-SMA-IF-CON1-5.jpg]

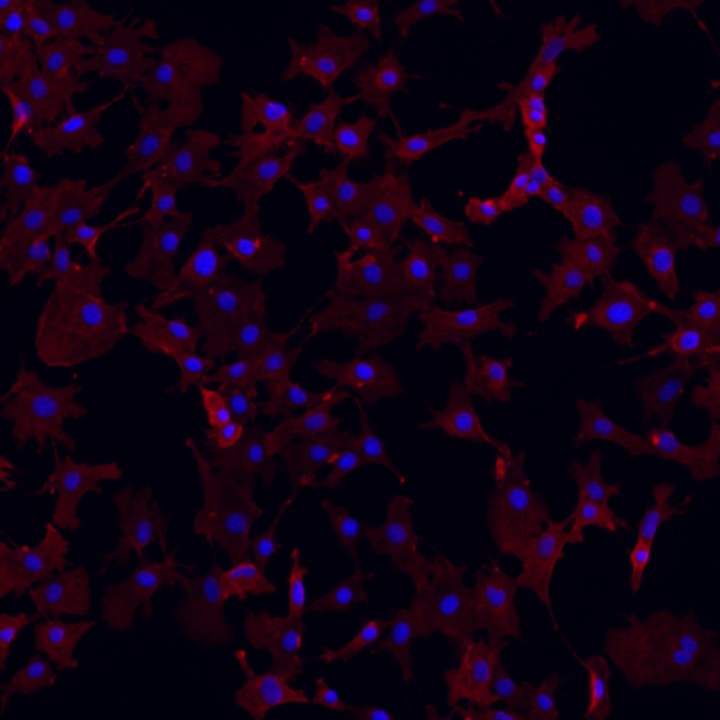

Supplement: Supplementary file 3 [file DataSheet2.zip › Original images and results for Figure 7/Fig. 7E/Fig. 7E α-SMA-IF/α-SMA-IF-CON2-1.jpg]

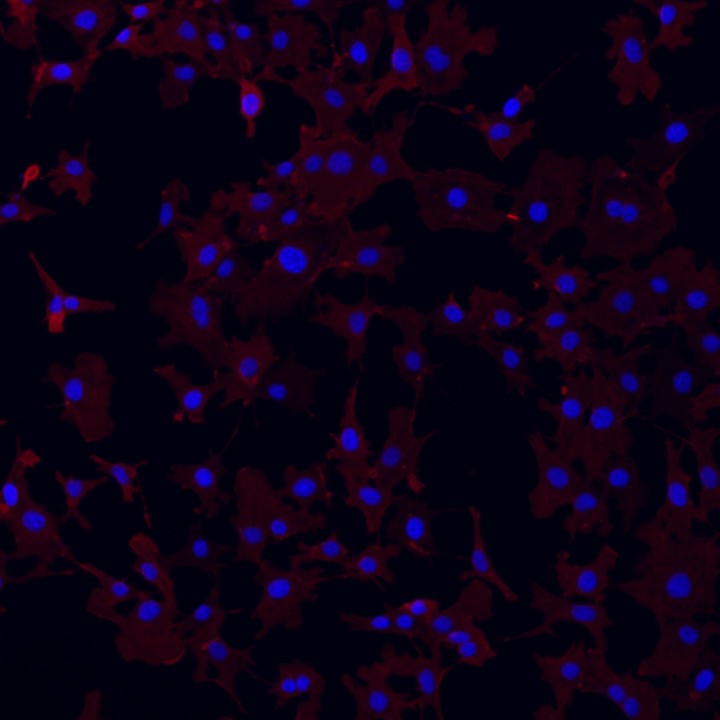

Supplement: Supplementary file 3 [file DataSheet2.zip › Original images and results for Figure 7/Fig. 7E/Fig. 7E α-SMA-IF/α-SMA-IF-CON2-2.jpg]

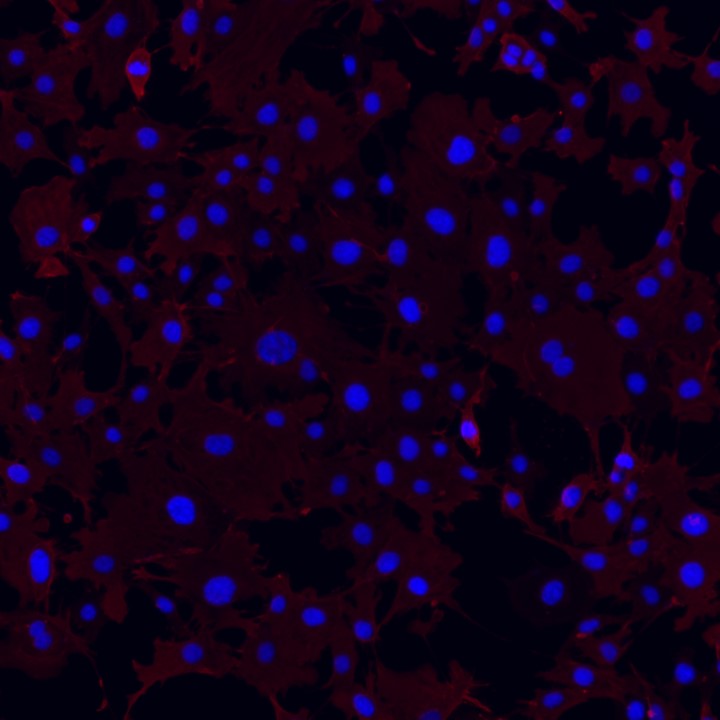

Supplement: Supplementary file 3 [file DataSheet2.zip › Original images and results for Figure 7/Fig. 7E/Fig. 7E α-SMA-IF/α-SMA-IF-CON2-3 image in Fig. 7E.jpg]

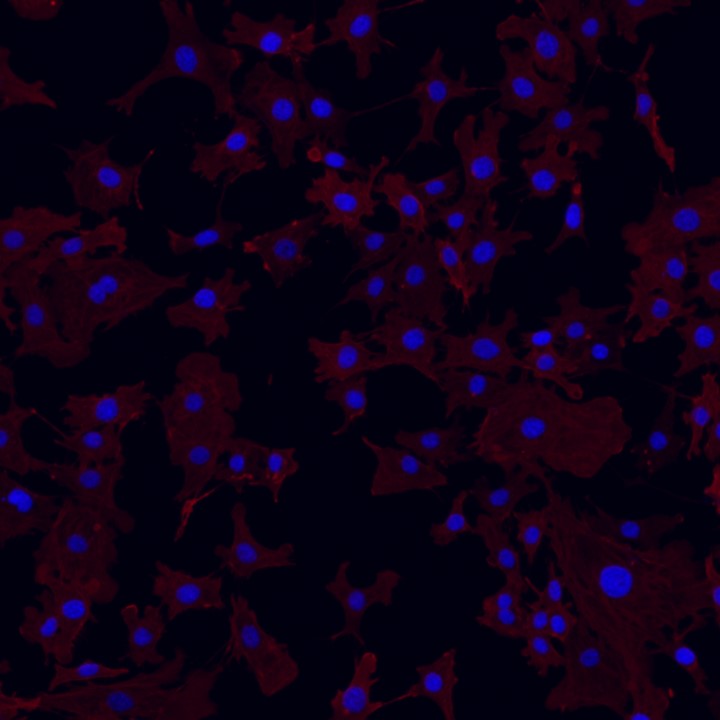

Supplement: Supplementary file 3 [file DataSheet2.zip › Original images and results for Figure 7/Fig. 7E/Fig. 7E α-SMA-IF/α-SMA-IF-CON2-4.jpg]

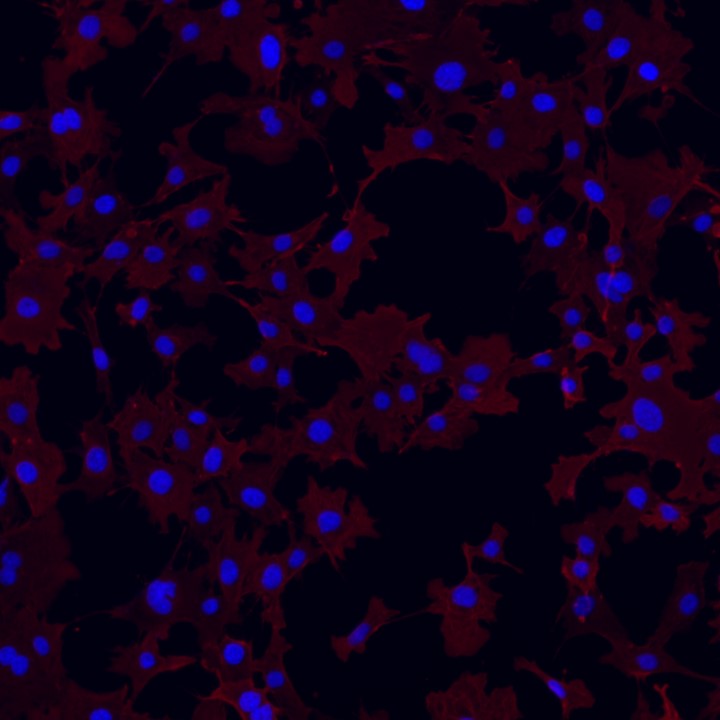

Supplement: Supplementary file 3 [file DataSheet2.zip › Original images and results for Figure 7/Fig. 7E/Fig. 7E α-SMA-IF/α-SMA-IF-CON2-5.jpg]

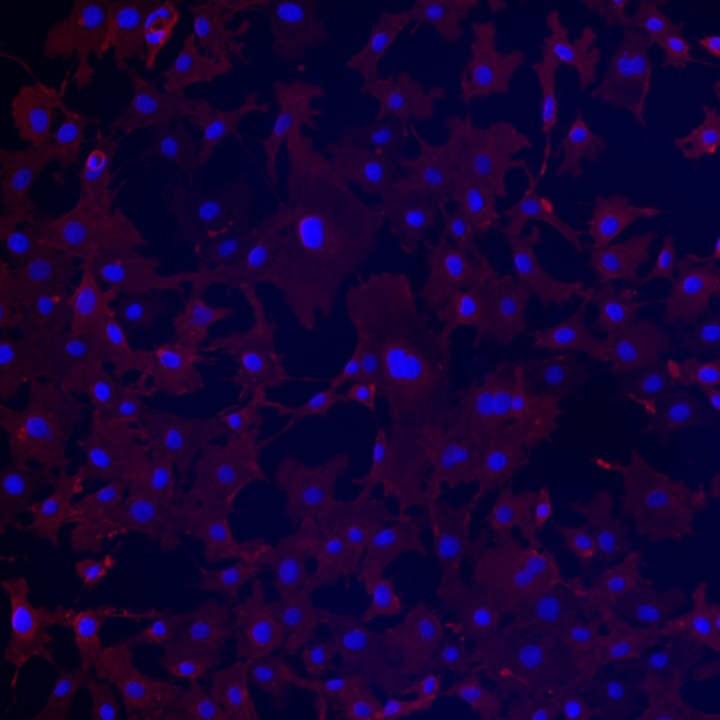

Supplement: Supplementary file 3 [file DataSheet2.zip › Original images and results for Figure 7/Fig. 7E/Fig. 7E α-SMA-IF/α-SMA-IF-CON3-1.jpg]

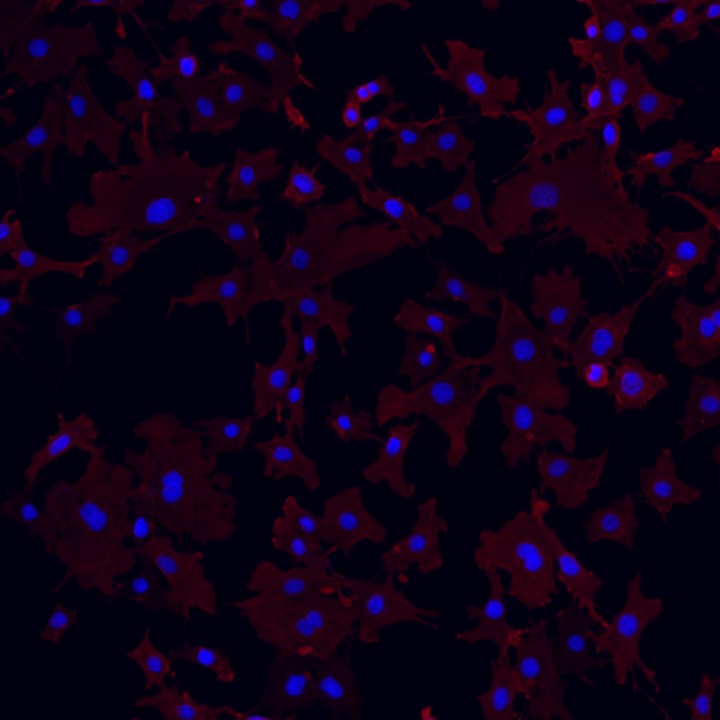

Supplement: Supplementary file 3 [file DataSheet2.zip › Original images and results for Figure 7/Fig. 7E/Fig. 7E α-SMA-IF/α-SMA-IF-CON3-2.jpg]

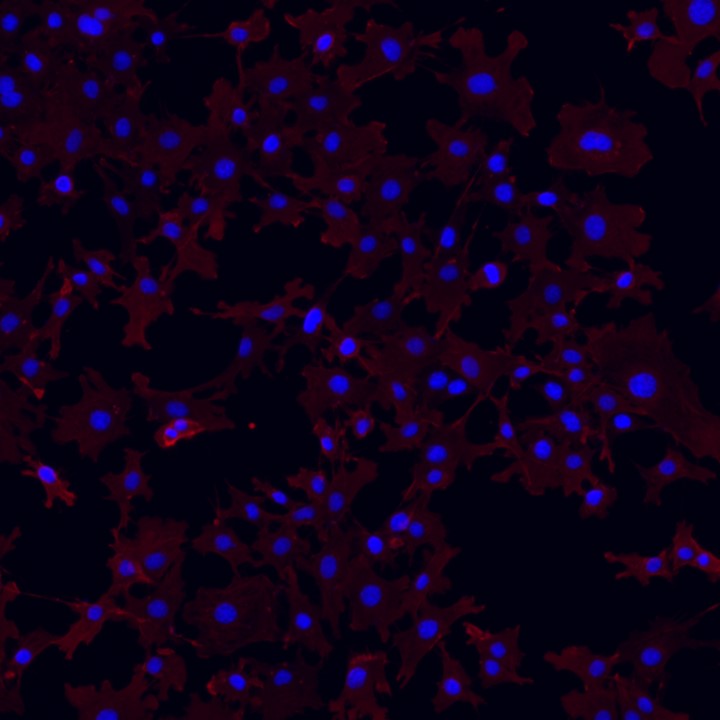

Supplement: Supplementary file 3 [file DataSheet2.zip › Original images and results for Figure 7/Fig. 7E/Fig. 7E α-SMA-IF/α-SMA-IF-CON3-3.jpg]

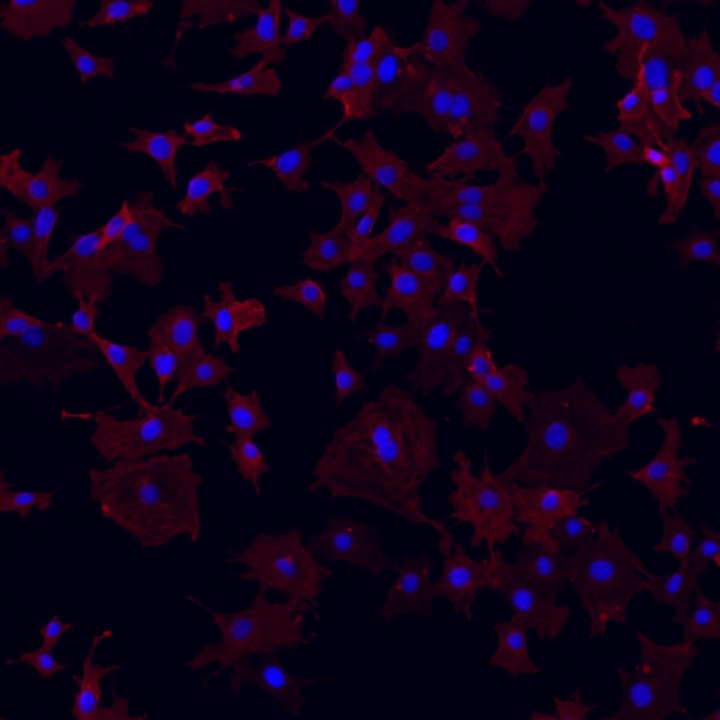

Supplement: Supplementary file 3 [file DataSheet2.zip › Original images and results for Figure 7/Fig. 7E/Fig. 7E α-SMA-IF/α-SMA-IF-CON3-4.jpg]

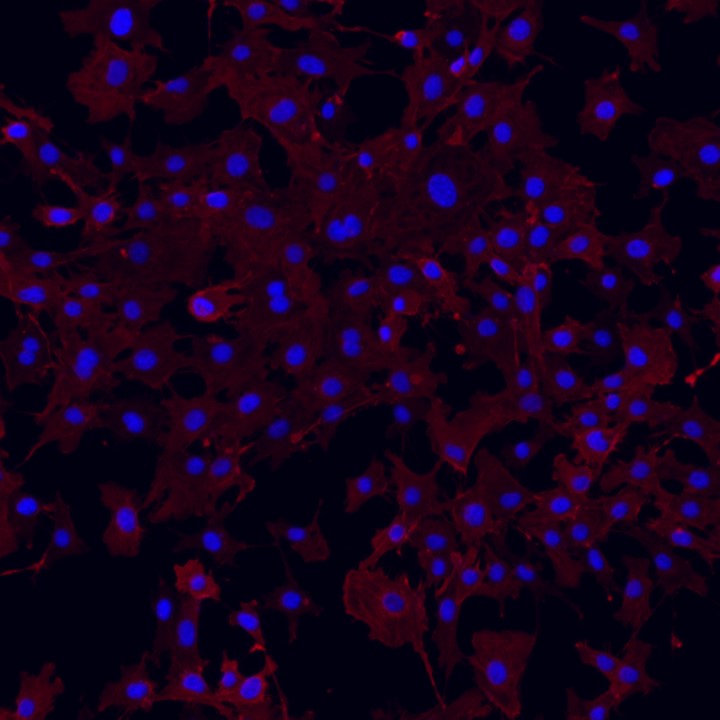

Supplement: Supplementary file 3 [file DataSheet2.zip › Original images and results for Figure 7/Fig. 7E/Fig. 7E α-SMA-IF/α-SMA-IF-CON3-5.jpg]

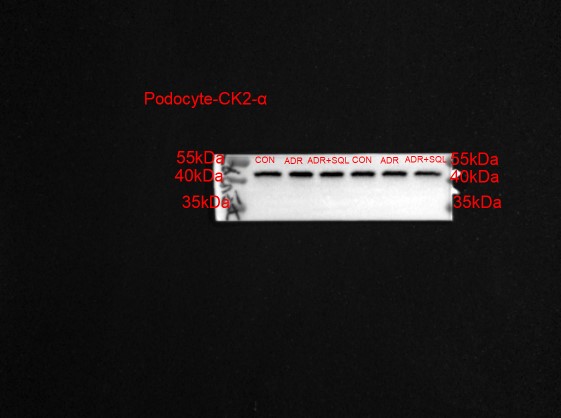

Supplement: Supplementary file 3 [file DataSheet2.zip › Original images and results for Figure 8/Fig. 8A/CK2-α/Fig 8A-Podocyte-CK2α 1 2-merge.jpg]

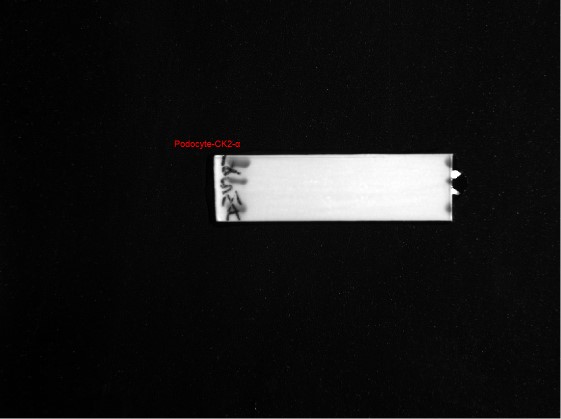

Supplement: Supplementary file 3 [file DataSheet2.zip › Original images and results for Figure 8/Fig. 8A/CK2-α/Fig 8A-Podocyte-CK2α 1 2-white light.jpg]

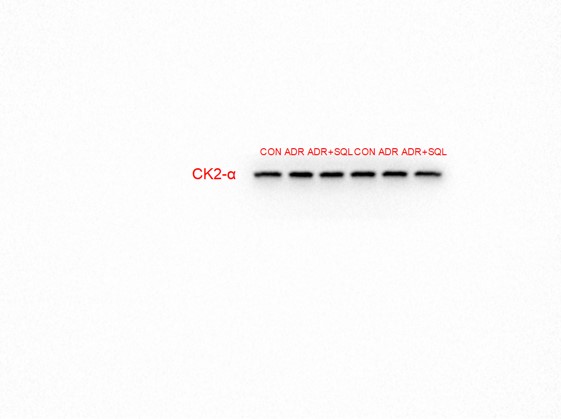

Supplement: Supplementary file 3 [file DataSheet2.zip › Original images and results for Figure 8/Fig. 8A/CK2-α/Fig 8A-Podocyte-CK2α 1 2.jpg]

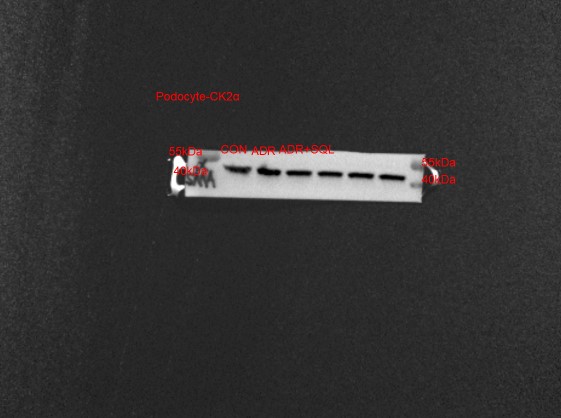

Supplement: Supplementary file 3 [file DataSheet2.zip › Original images and results for Figure 8/Fig. 8A/CK2-α/Fig 8A-Podocyte-CK2α 3-merge image in Fig. 8A.jpg]

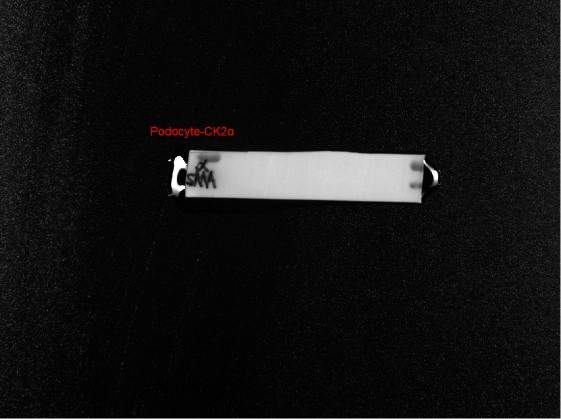

Supplement: Supplementary file 3 [file DataSheet2.zip › Original images and results for Figure 8/Fig. 8A/CK2-α/Fig 8A-Podocyte-CK2α 3-white light.jpg]

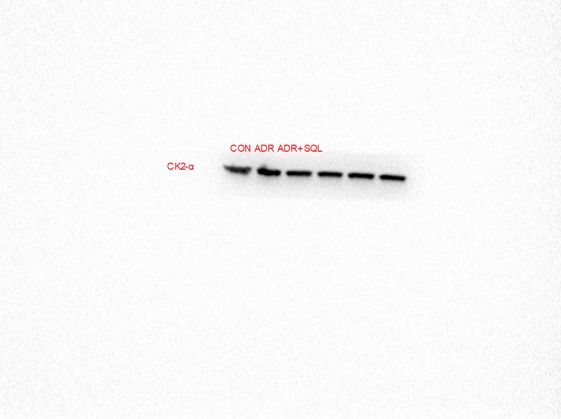

Supplement: Supplementary file 3 [file DataSheet2.zip › Original images and results for Figure 8/Fig. 8A/CK2-α/Fig 8A-Podocyte-CK2α 3.jpg]

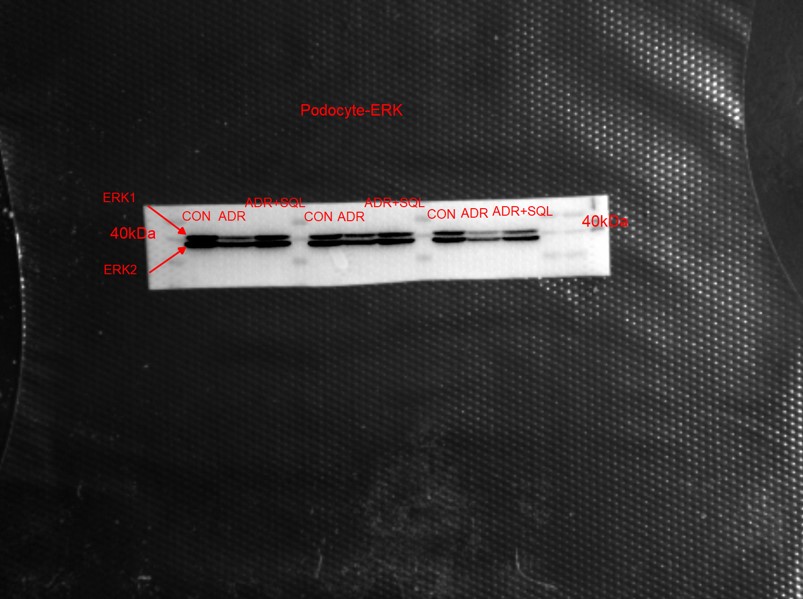

Supplement: Supplementary file 3 [file DataSheet2.zip › Original images and results for Figure 8/Fig. 8A/ERK/Fig 8A-Podocyte-ERK-merge right-image in Fig.8A.jpg]

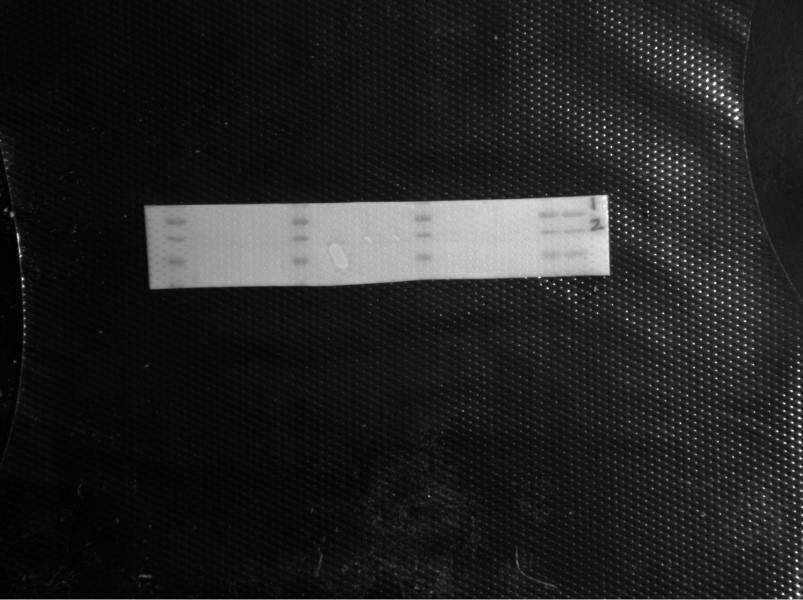

Supplement: Supplementary file 3 [file DataSheet2.zip › Original images and results for Figure 8/Fig. 8A/ERK/Fig 8A-Podocyte-ERK-white light.jpg]

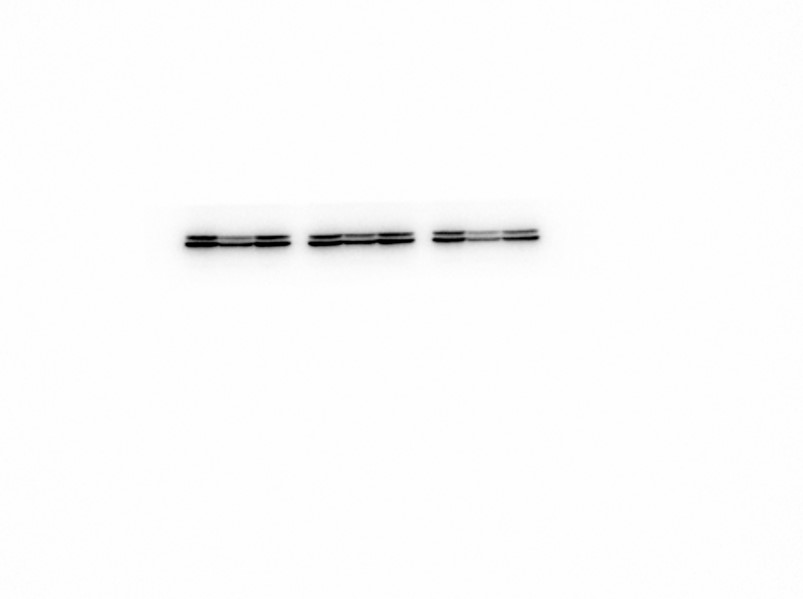

Supplement: Supplementary file 3 [file DataSheet2.zip › Original images and results for Figure 8/Fig. 8A/ERK/Fig 8A-Podocyte-ERK.jpg]

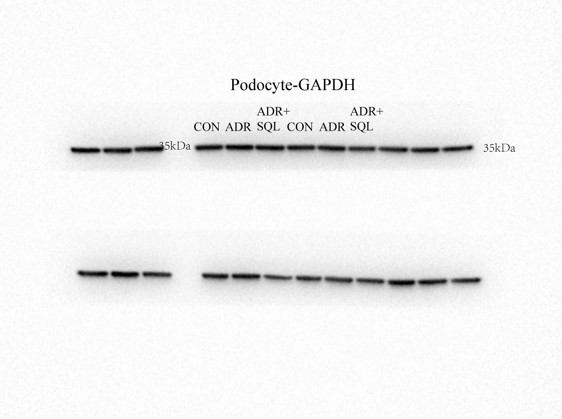

Supplement: Supplementary file 3 [file DataSheet2.zip › Original images and results for Figure 8/Fig. 8A/GAPDH/Fig 8A-Podocyte-GAPDH-1 2 left-image in Fig. 8A.jpg]

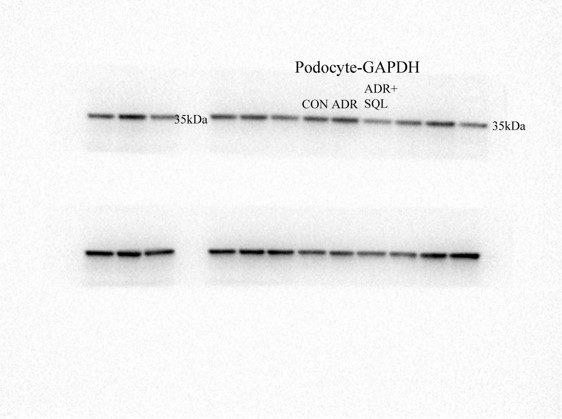

Supplement: Supplementary file 3 [file DataSheet2.zip › Original images and results for Figure 8/Fig. 8A/GAPDH/Fig 8A-Podocyte-GAPDH-3.jpg]

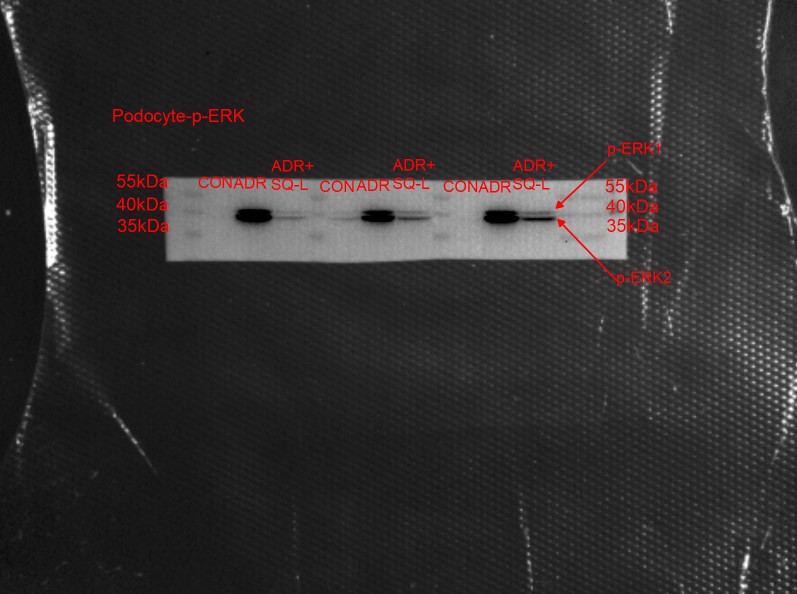

Supplement: Supplementary file 3 [file DataSheet2.zip › Original images and results for Figure 8/Fig. 8A/p-ERK/Fig 8A-Podocyte-p-ERK-merge middle-image in Fig. 8A.jpg]
