# Supplementary figures and images for: Sanqi oral solution alleviates podocyte apoptosis in experimental membranous nephropathy by mediating EMT through the ERK/CK2-α/β-catenin pathway (part 3 of 4)
Source: Front Pharmacol. 2025 May 9;16:1503961. doi: 10.3389/fphar.2025.1503961 (PMC12098599; doi:10.3389/fphar.2025.1503961)

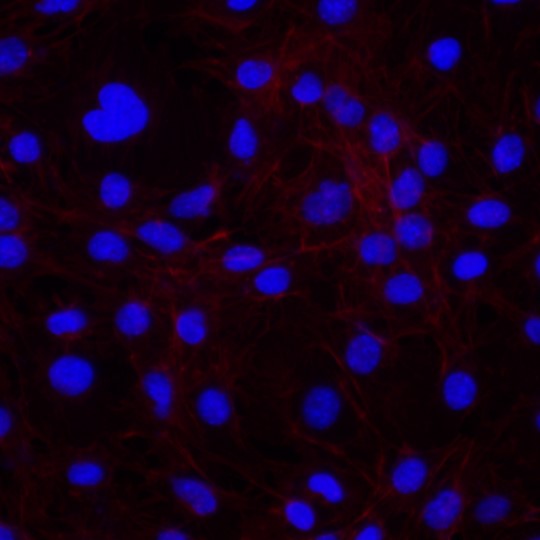

Supplement: Supplementary file 3 [file DataSheet2.zip › Original images and results for Figure 6/Fig. 6B/Fig. 6B Phalloidin/IF-Phalloidin-ADR-1-5.jpg]

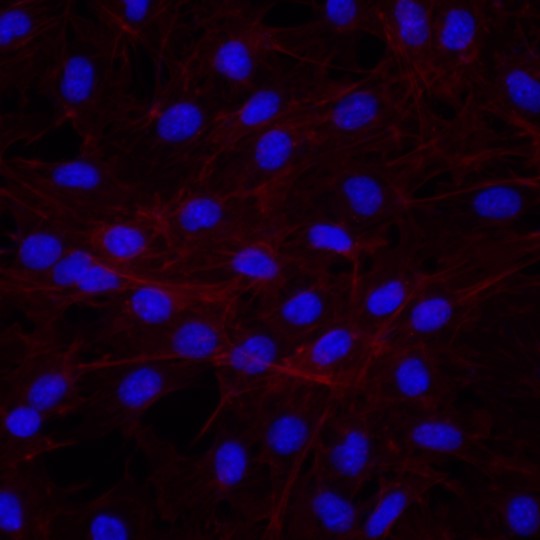

Supplement: Supplementary file 3 [file DataSheet2.zip › Original images and results for Figure 6/Fig. 6B/Fig. 6B Phalloidin/IF-Phalloidin-ADR-2-1.jpg]

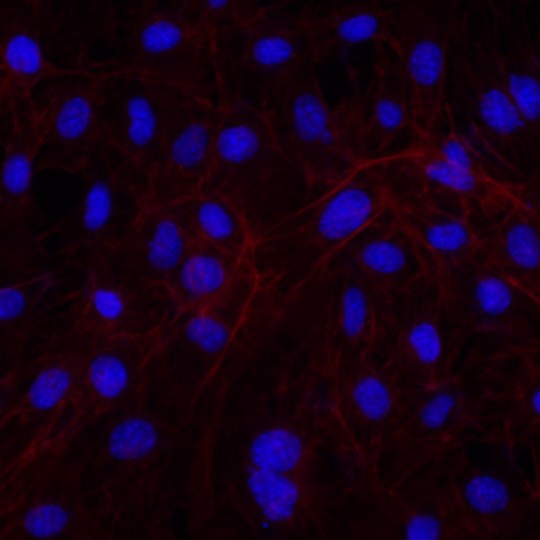

Supplement: Supplementary file 3 [file DataSheet2.zip › Original images and results for Figure 6/Fig. 6B/Fig. 6B Phalloidin/IF-Phalloidin-ADR-2-2.jpg]

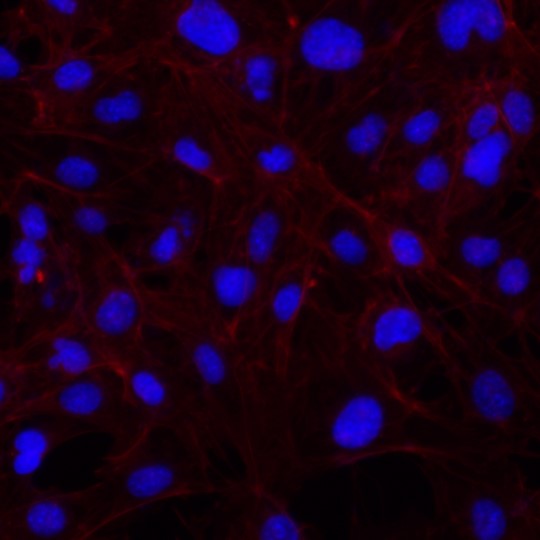

Supplement: Supplementary file 3 [file DataSheet2.zip › Original images and results for Figure 6/Fig. 6B/Fig. 6B Phalloidin/IF-Phalloidin-ADR-2-3.jpg]

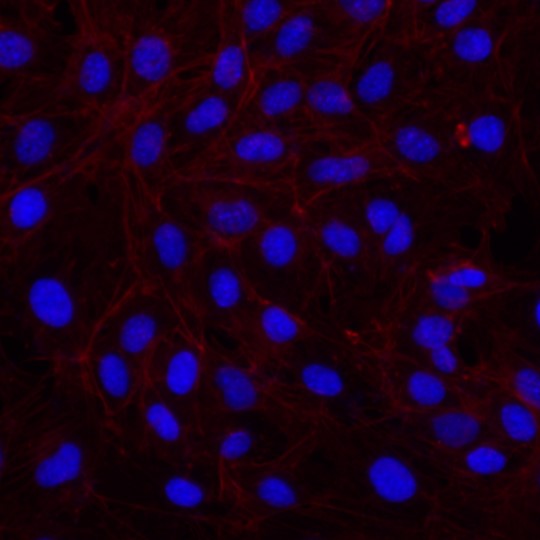

Supplement: Supplementary file 3 [file DataSheet2.zip › Original images and results for Figure 6/Fig. 6B/Fig. 6B Phalloidin/IF-Phalloidin-ADR-2-4.jpg]

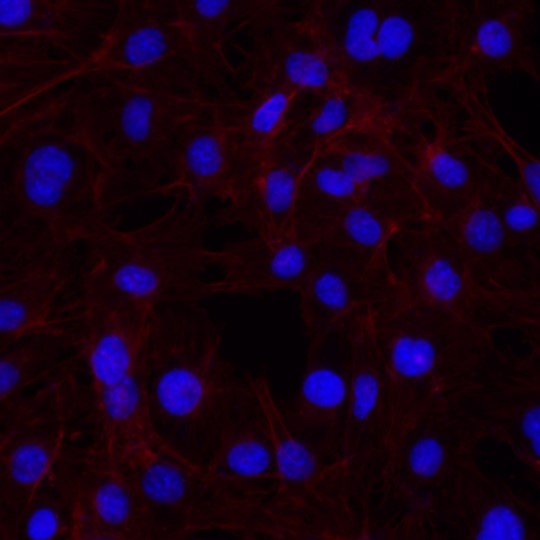

Supplement: Supplementary file 3 [file DataSheet2.zip › Original images and results for Figure 6/Fig. 6B/Fig. 6B Phalloidin/IF-Phalloidin-ADR-2-5.jpg]

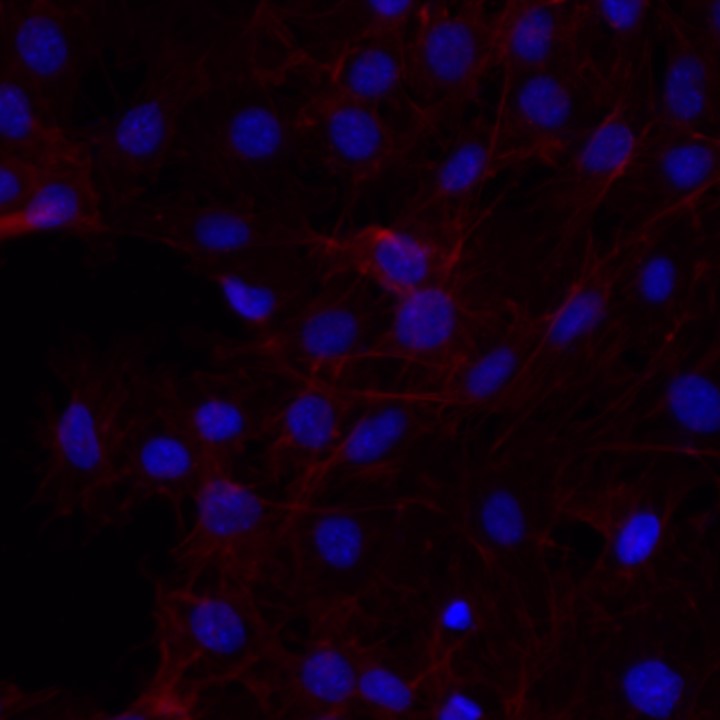

Supplement: Supplementary file 3 [file DataSheet2.zip › Original images and results for Figure 6/Fig. 6B/Fig. 6B Phalloidin/IF-Phalloidin-ADR-3-1.jpg]

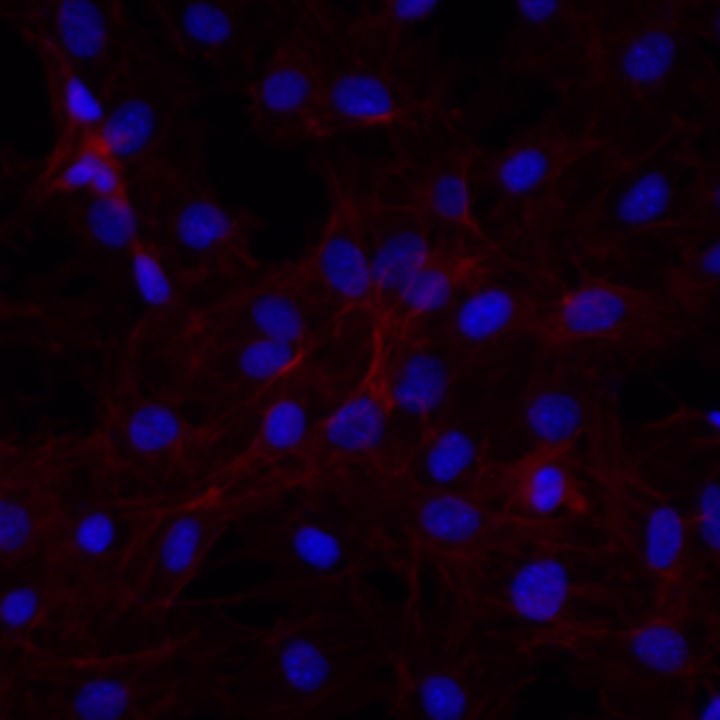

Supplement: Supplementary file 3 [file DataSheet2.zip › Original images and results for Figure 6/Fig. 6B/Fig. 6B Phalloidin/IF-Phalloidin-ADR-3-2.jpg]

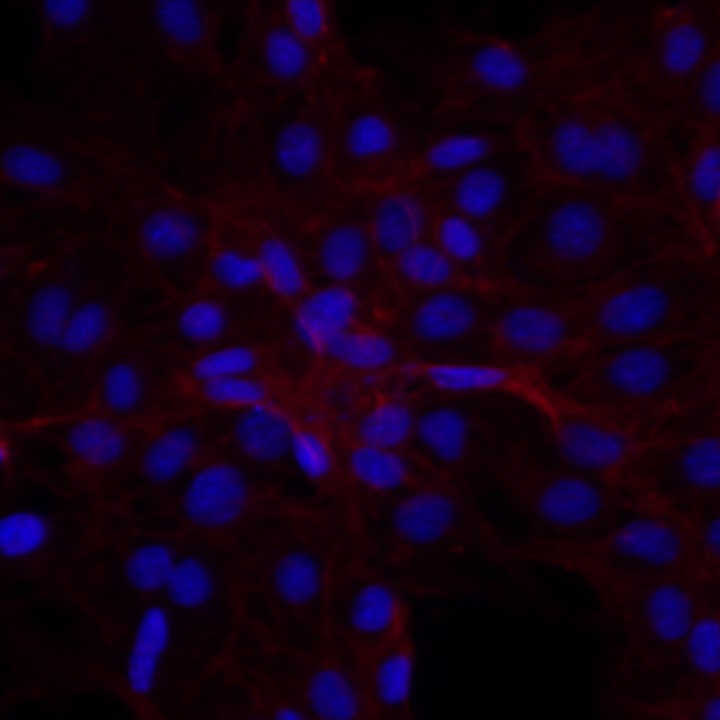

Supplement: Supplementary file 3 [file DataSheet2.zip › Original images and results for Figure 6/Fig. 6B/Fig. 6B Phalloidin/IF-Phalloidin-ADR-3-3.jpg]

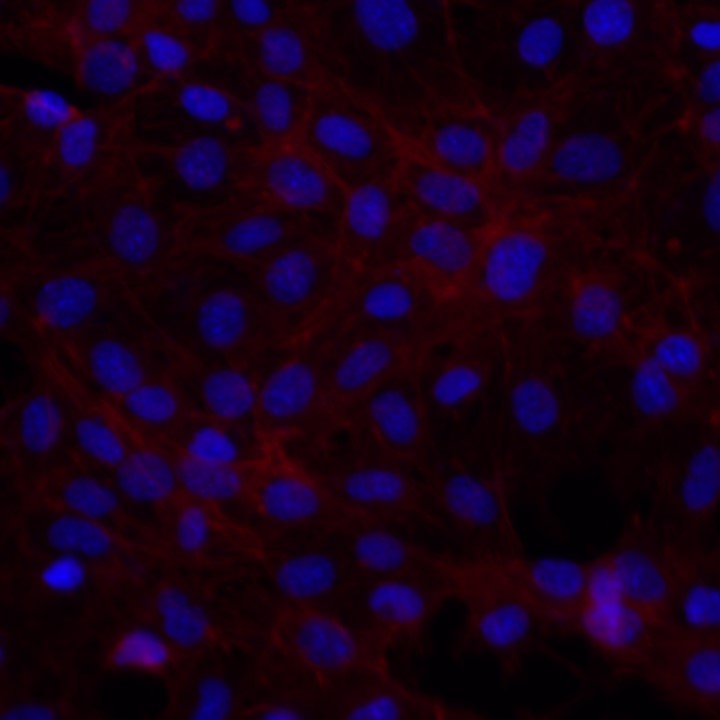

Supplement: Supplementary file 3 [file DataSheet2.zip › Original images and results for Figure 6/Fig. 6B/Fig. 6B Phalloidin/IF-Phalloidin-ADR-3-4.jpg]

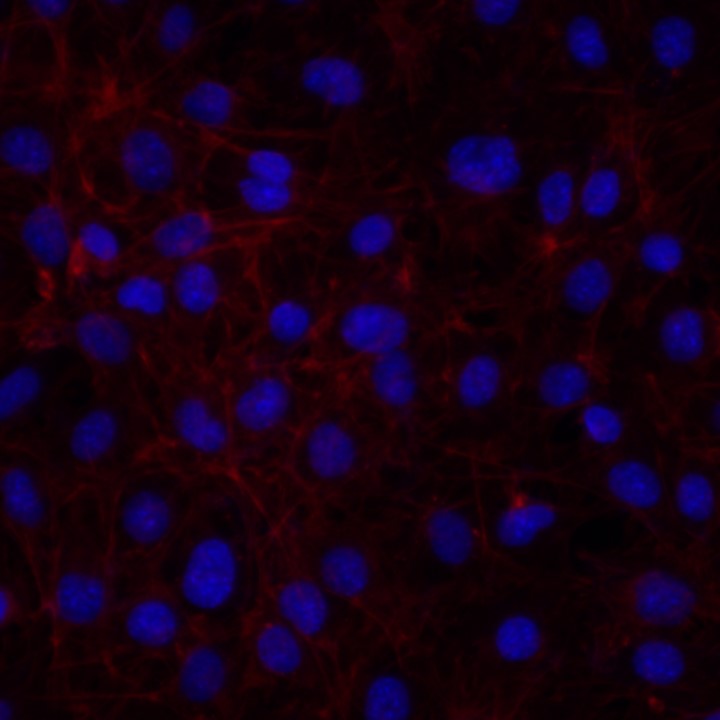

Supplement: Supplementary file 3 [file DataSheet2.zip › Original images and results for Figure 6/Fig. 6B/Fig. 6B Phalloidin/IF-Phalloidin-ADR-3-5.jpg]

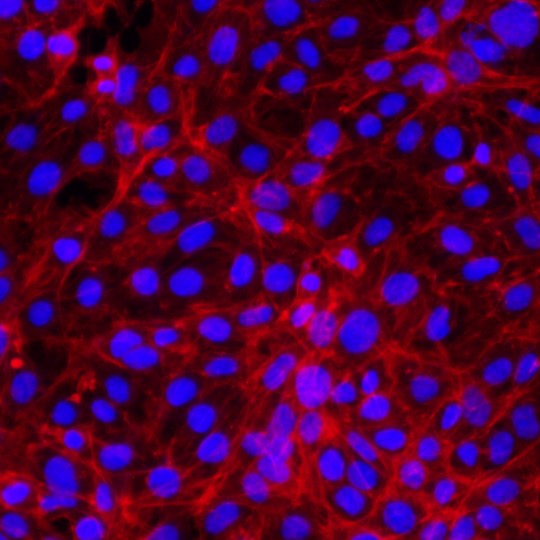

Supplement: Supplementary file 3 [file DataSheet2.zip › Original images and results for Figure 6/Fig. 6B/Fig. 6B Phalloidin/IF-Phalloidin-CON-1-1 image in Fig. 6B.jpg]

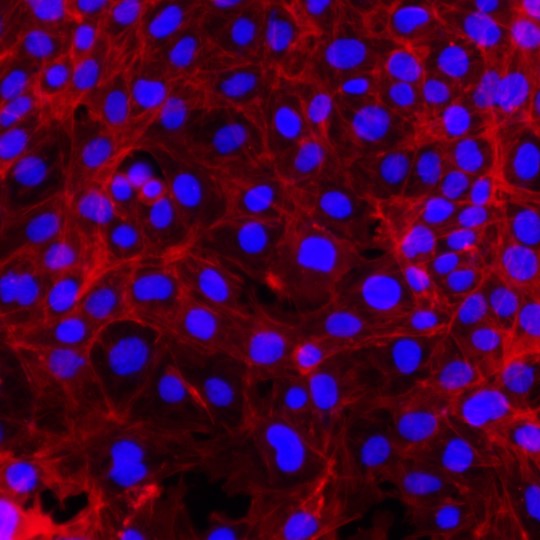

Supplement: Supplementary file 3 [file DataSheet2.zip › Original images and results for Figure 6/Fig. 6B/Fig. 6B Phalloidin/IF-Phalloidin-CON-1-2.jpg]

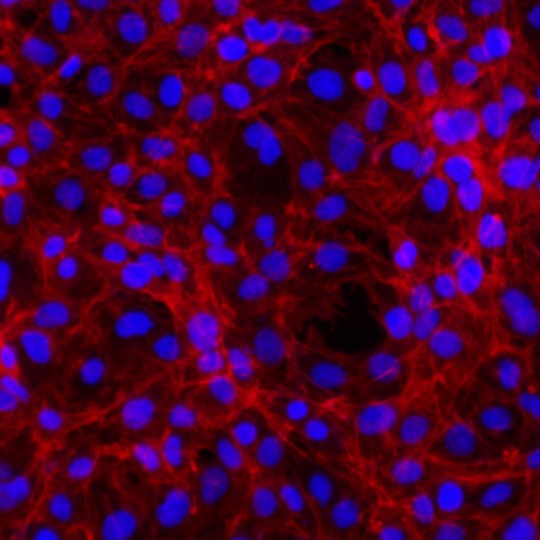

Supplement: Supplementary file 3 [file DataSheet2.zip › Original images and results for Figure 6/Fig. 6B/Fig. 6B Phalloidin/IF-Phalloidin-CON-1-3.jpg]

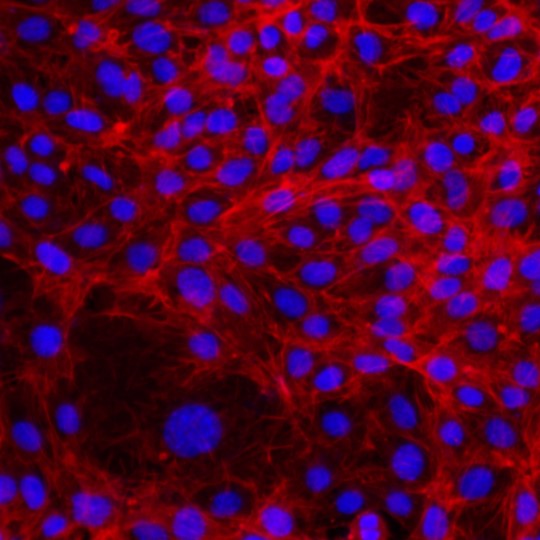

Supplement: Supplementary file 3 [file DataSheet2.zip › Original images and results for Figure 6/Fig. 6B/Fig. 6B Phalloidin/IF-Phalloidin-CON-1-4.jpg]

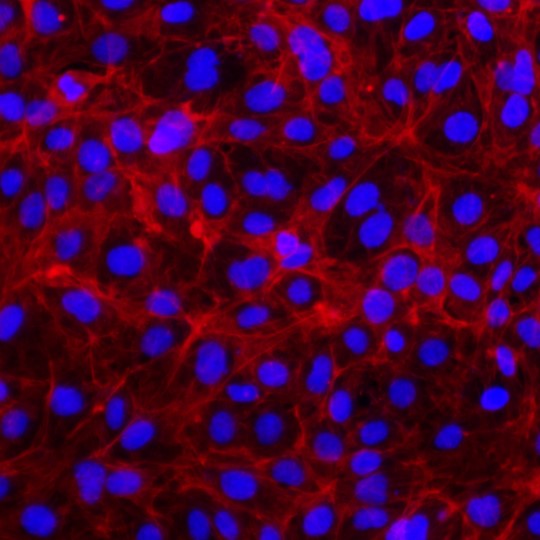

Supplement: Supplementary file 3 [file DataSheet2.zip › Original images and results for Figure 6/Fig. 6B/Fig. 6B Phalloidin/IF-Phalloidin-CON-1-5.jpg]

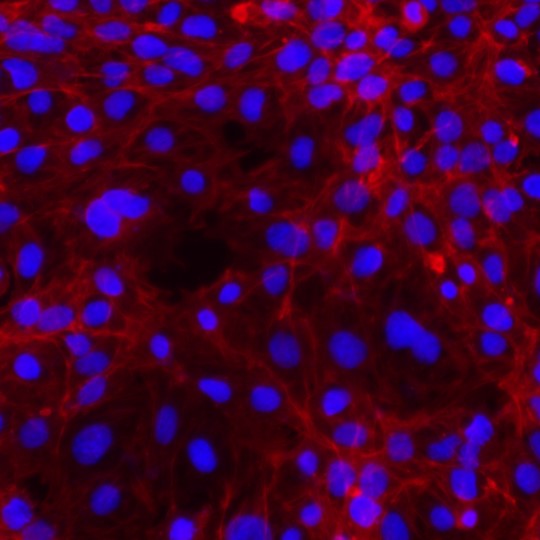

Supplement: Supplementary file 3 [file DataSheet2.zip › Original images and results for Figure 6/Fig. 6B/Fig. 6B Phalloidin/IF-Phalloidin-CON-2-1.jpg]

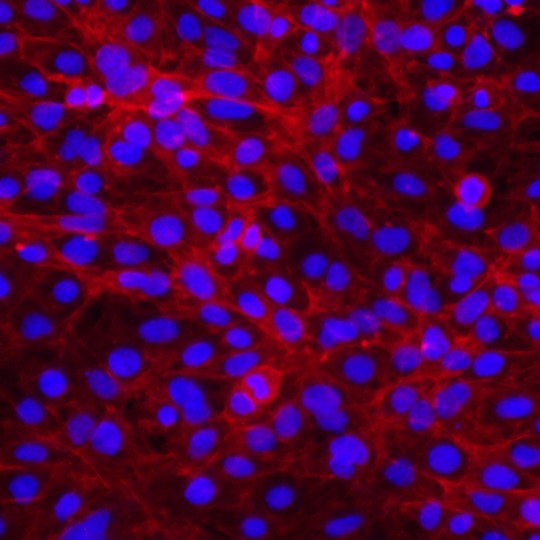

Supplement: Supplementary file 3 [file DataSheet2.zip › Original images and results for Figure 6/Fig. 6B/Fig. 6B Phalloidin/IF-Phalloidin-CON-2-2.jpg]

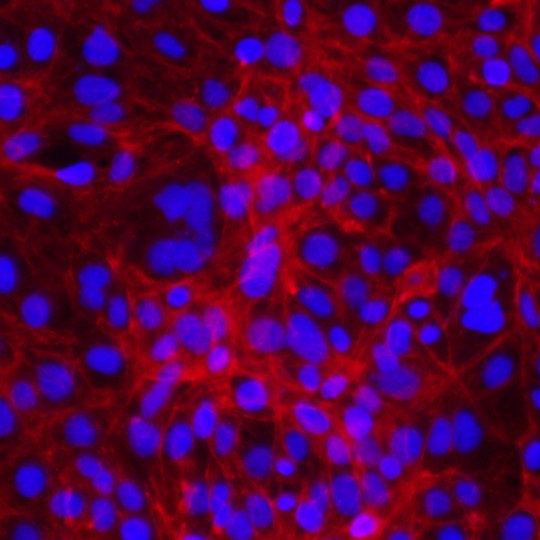

Supplement: Supplementary file 3 [file DataSheet2.zip › Original images and results for Figure 6/Fig. 6B/Fig. 6B Phalloidin/IF-Phalloidin-CON-2-3.jpg]

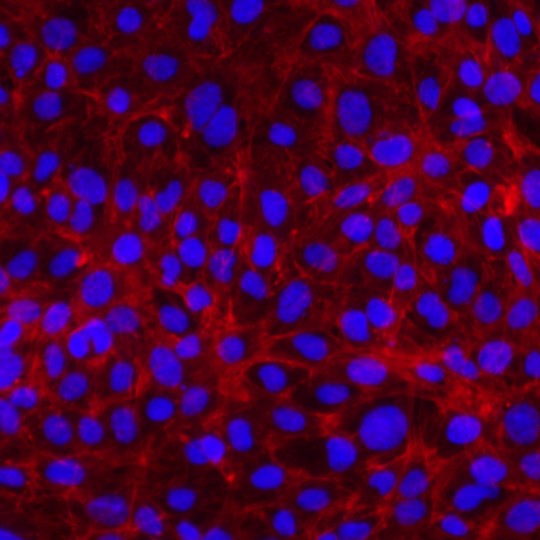

Supplement: Supplementary file 3 [file DataSheet2.zip › Original images and results for Figure 6/Fig. 6B/Fig. 6B Phalloidin/IF-Phalloidin-CON-2-4.jpg]

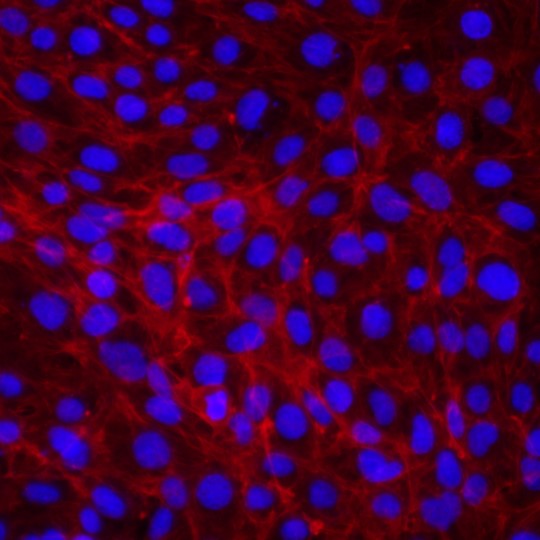

Supplement: Supplementary file 3 [file DataSheet2.zip › Original images and results for Figure 6/Fig. 6B/Fig. 6B Phalloidin/IF-Phalloidin-CON-2-5.jpg]

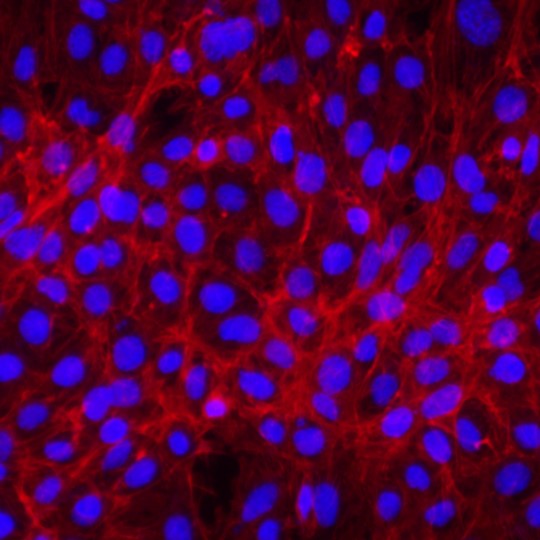

Supplement: Supplementary file 3 [file DataSheet2.zip › Original images and results for Figure 6/Fig. 6B/Fig. 6B Phalloidin/IF-Phalloidin-CON-3-1.jpg]

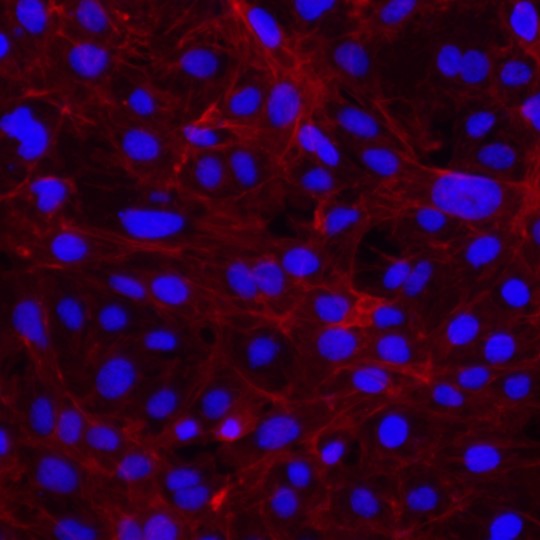

Supplement: Supplementary file 3 [file DataSheet2.zip › Original images and results for Figure 6/Fig. 6B/Fig. 6B Phalloidin/IF-Phalloidin-CON-3-2.jpg]

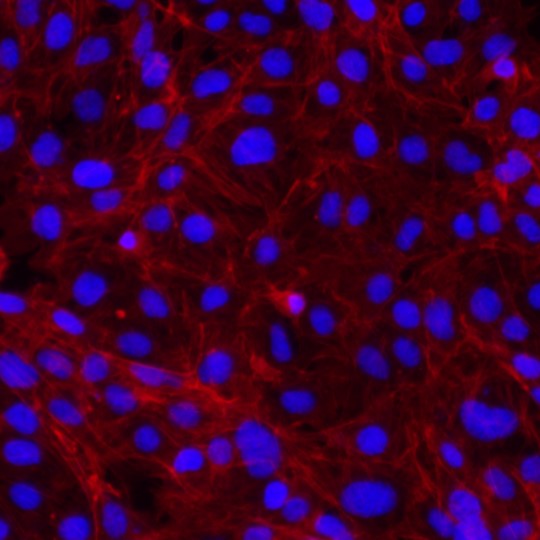

Supplement: Supplementary file 3 [file DataSheet2.zip › Original images and results for Figure 6/Fig. 6B/Fig. 6B Phalloidin/IF-Phalloidin-CON-3-3.jpg]

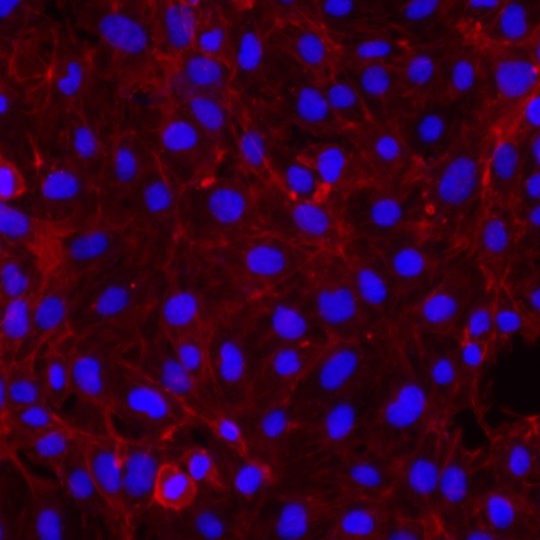

Supplement: Supplementary file 3 [file DataSheet2.zip › Original images and results for Figure 6/Fig. 6B/Fig. 6B Phalloidin/IF-Phalloidin-CON-3-4.jpg]

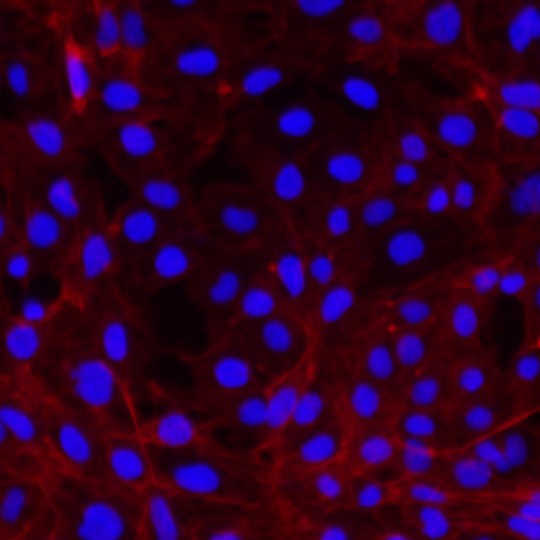

Supplement: Supplementary file 3 [file DataSheet2.zip › Original images and results for Figure 6/Fig. 6B/Fig. 6B Phalloidin/IF-Phalloidin-CON-3-5.jpg]

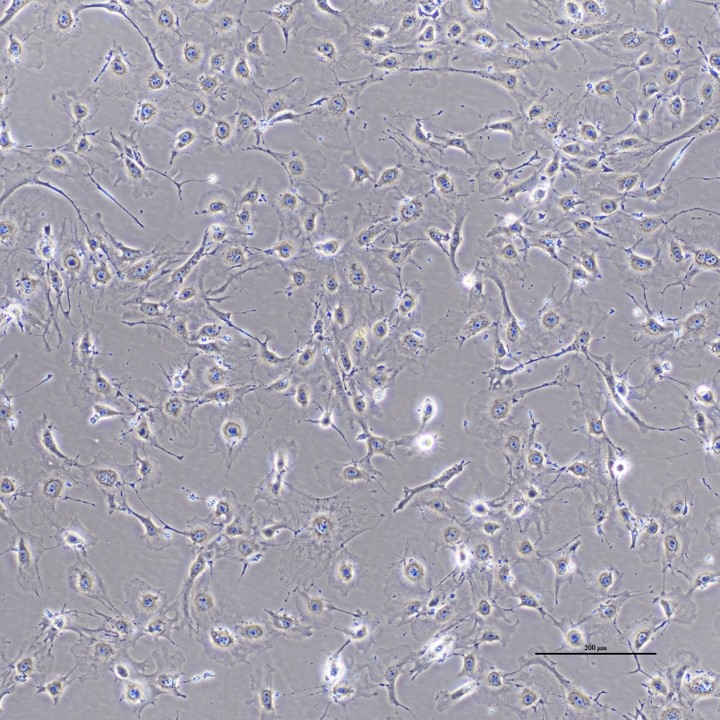

Supplement: Supplementary file 3 [file DataSheet2.zip › Original images and results for Figure 6/Fig. 6B/Fig. 6B Podocyte morphology/Podocyte-ADR 1.jpg]

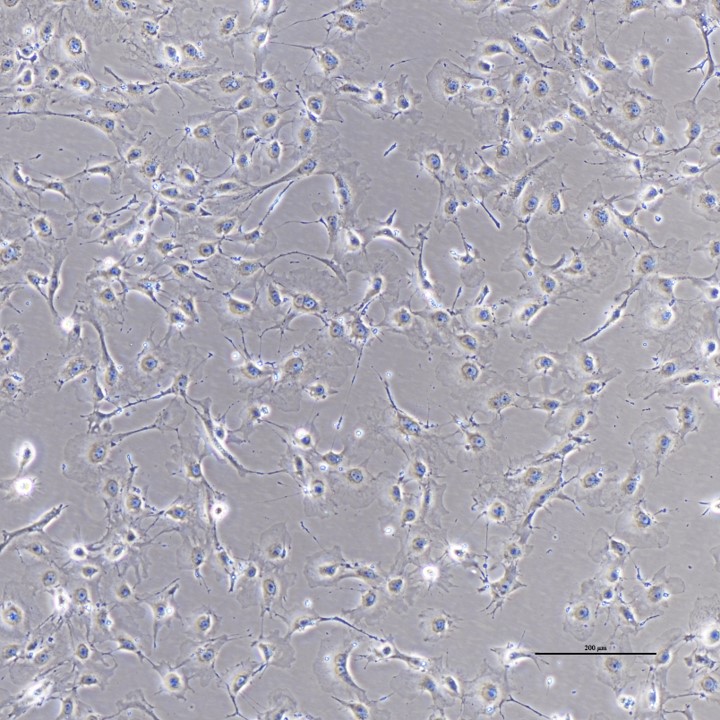

Supplement: Supplementary file 3 [file DataSheet2.zip › Original images and results for Figure 6/Fig. 6B/Fig. 6B Podocyte morphology/Podocyte-ADR 2 image in Fig. 6B.jpg]

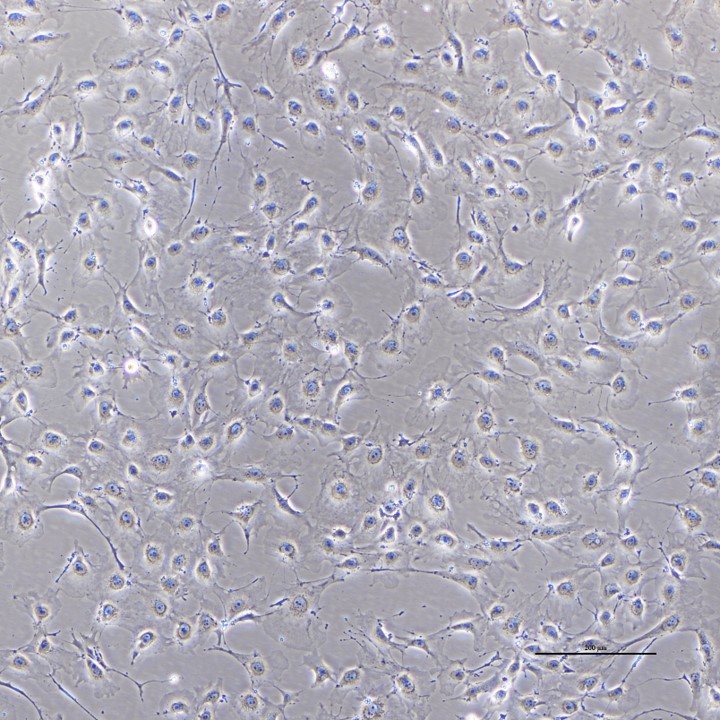

Supplement: Supplementary file 3 [file DataSheet2.zip › Original images and results for Figure 6/Fig. 6B/Fig. 6B Podocyte morphology/Podocyte-ADR 3.jpg]

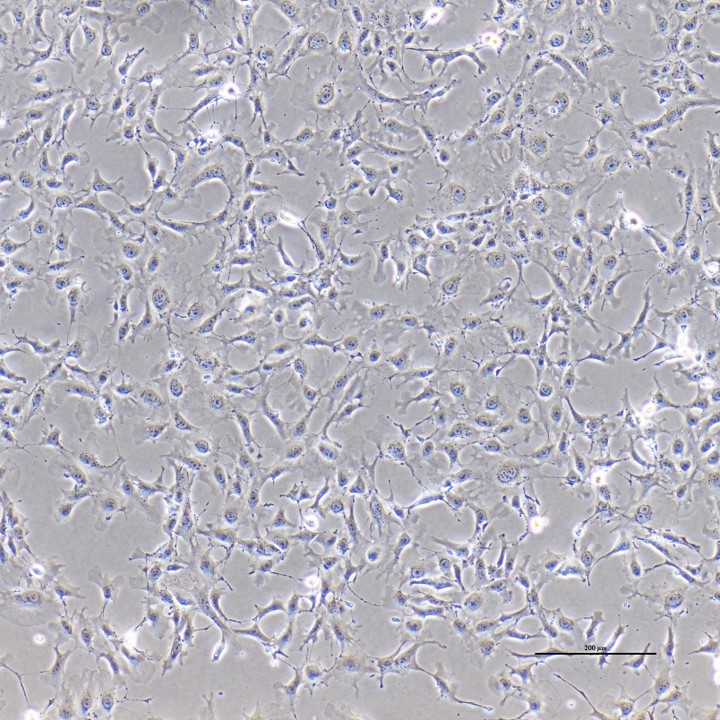

Supplement: Supplementary file 3 [file DataSheet2.zip › Original images and results for Figure 6/Fig. 6B/Fig. 6B Podocyte morphology/Podocyte-ADR+SQL 1.jpg]

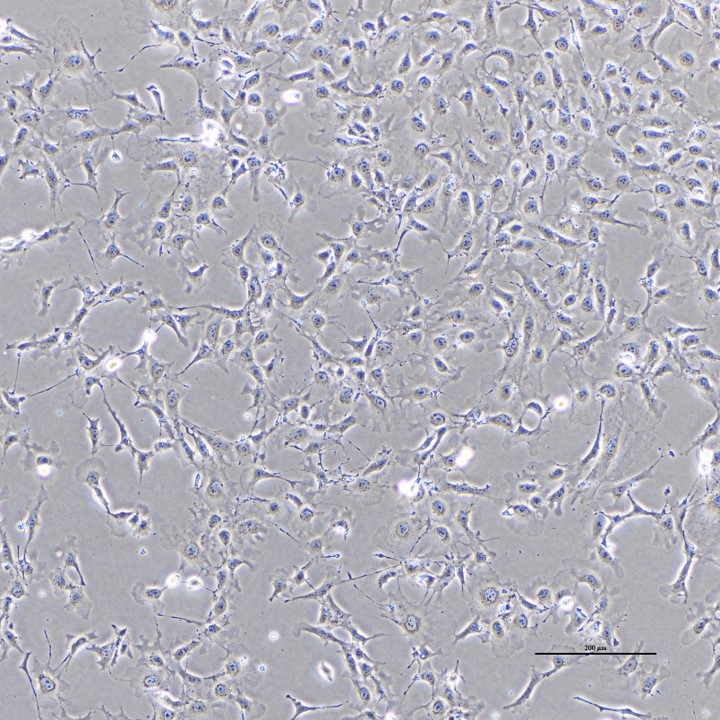

Supplement: Supplementary file 3 [file DataSheet2.zip › Original images and results for Figure 6/Fig. 6B/Fig. 6B Podocyte morphology/Podocyte-ADR+SQL 2.jpg]

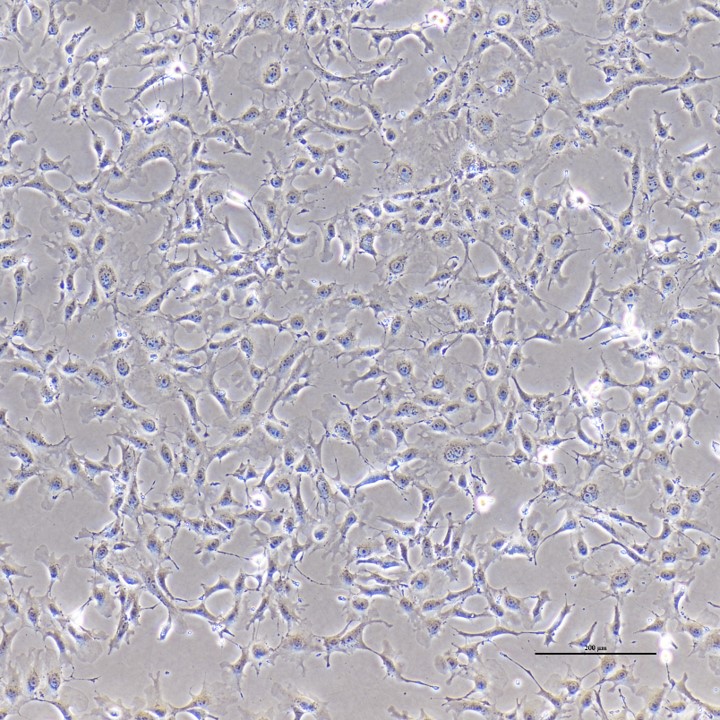

Supplement: Supplementary file 3 [file DataSheet2.zip › Original images and results for Figure 6/Fig. 6B/Fig. 6B Podocyte morphology/Podocyte-ADR+SQL 3 image in Fig. 6B.jpg]

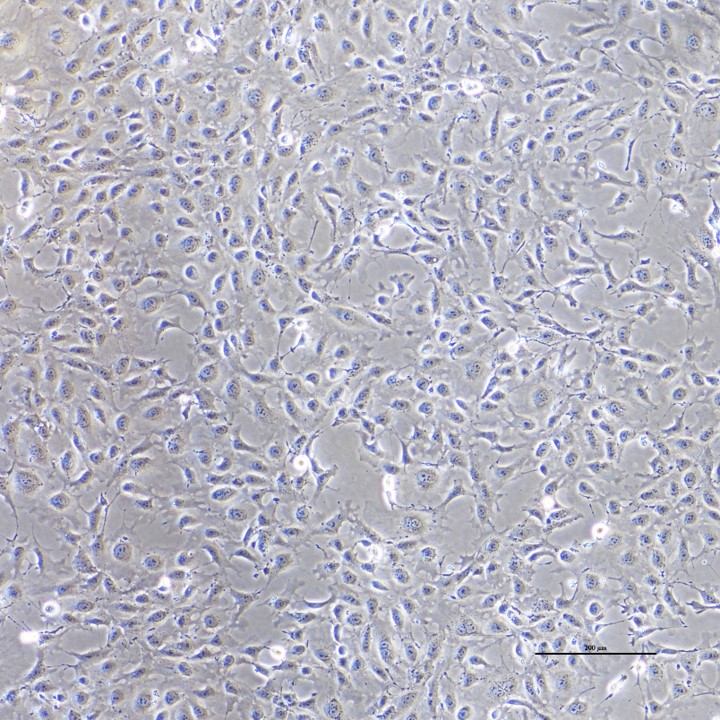

Supplement: Supplementary file 3 [file DataSheet2.zip › Original images and results for Figure 6/Fig. 6B/Fig. 6B Podocyte morphology/Podocyte-CON 1 image in Fig. 6B.jpg]

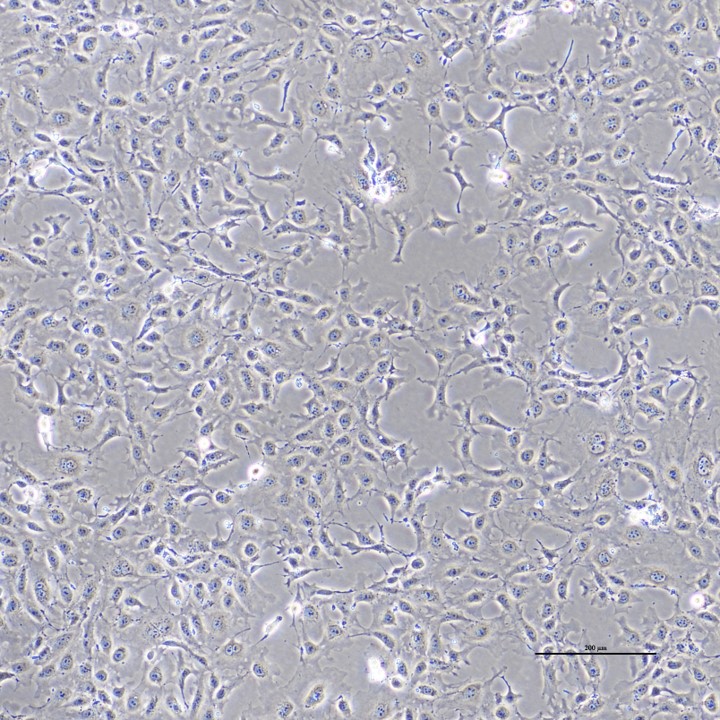

Supplement: Supplementary file 3 [file DataSheet2.zip › Original images and results for Figure 6/Fig. 6B/Fig. 6B Podocyte morphology/Podocyte-CON 2.jpg]

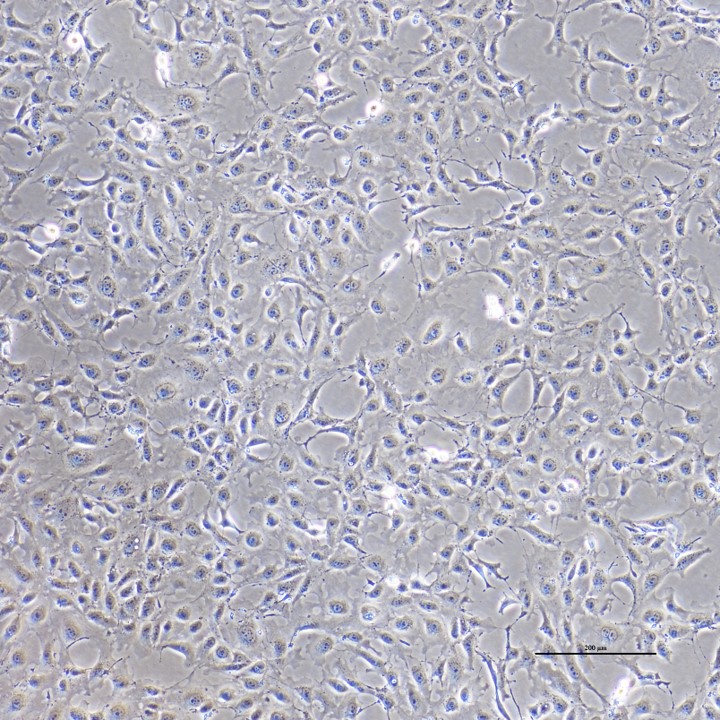

Supplement: Supplementary file 3 [file DataSheet2.zip › Original images and results for Figure 6/Fig. 6B/Fig. 6B Podocyte morphology/Podocyte-CON 3.jpg]

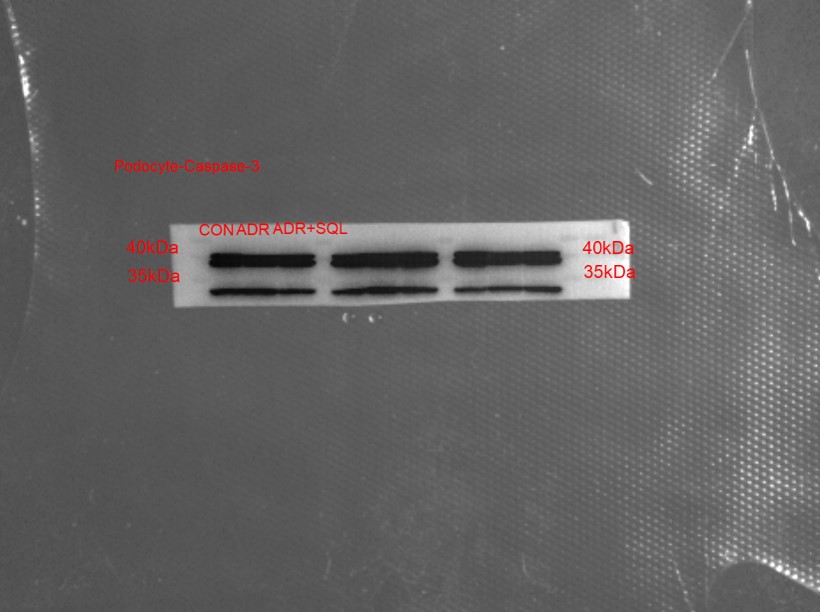

Supplement: Supplementary file 3 [file DataSheet2.zip › Original images and results for Figure 7/Fig. 7A/Caspase-3/Fig 7A-Podocyte-Caspase-3 1-merge image in Fig. 7A.jpg]

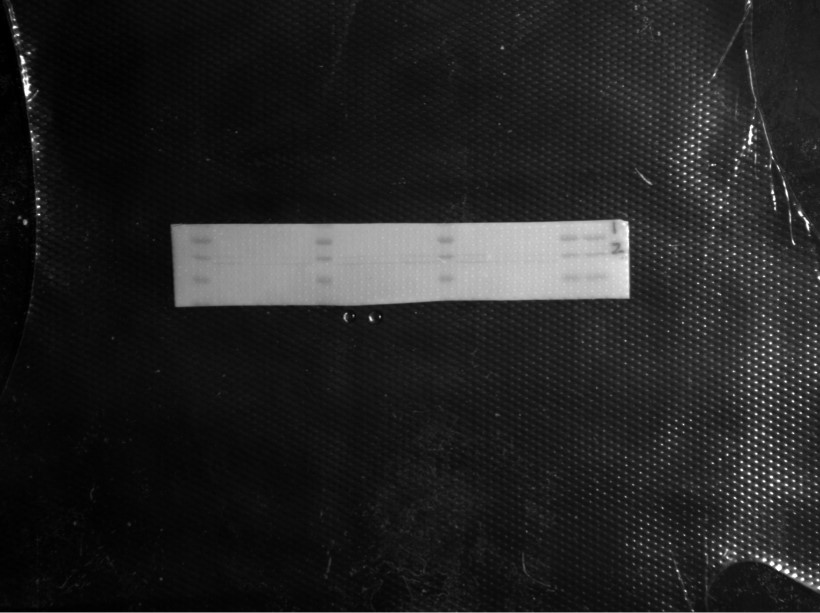

Supplement: Supplementary file 3 [file DataSheet2.zip › Original images and results for Figure 7/Fig. 7A/Caspase-3/Fig 7A-Podocyte-Caspase-3 1-white light.jpg]

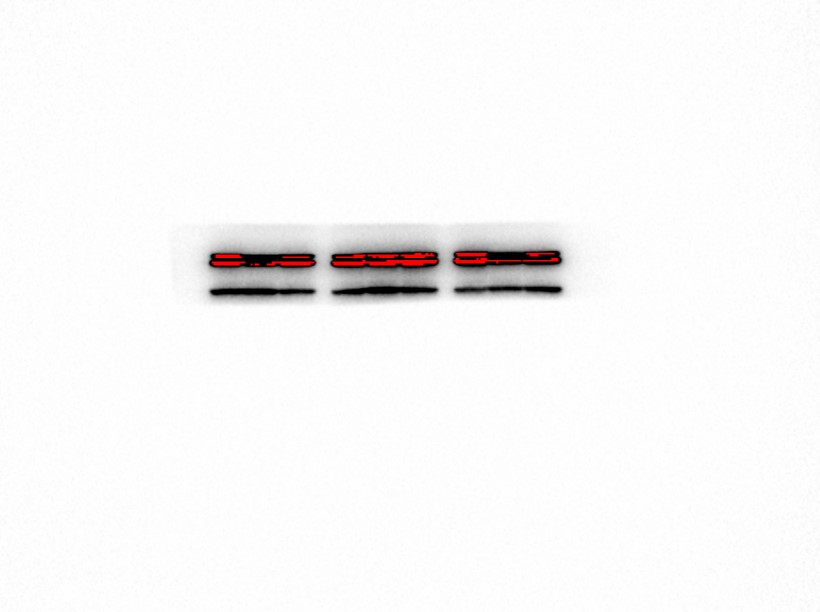

Supplement: Supplementary file 3 [file DataSheet2.zip › Original images and results for Figure 7/Fig. 7A/Caspase-3/Fig 7A-Podocyte-Caspase-3 1.jpg]

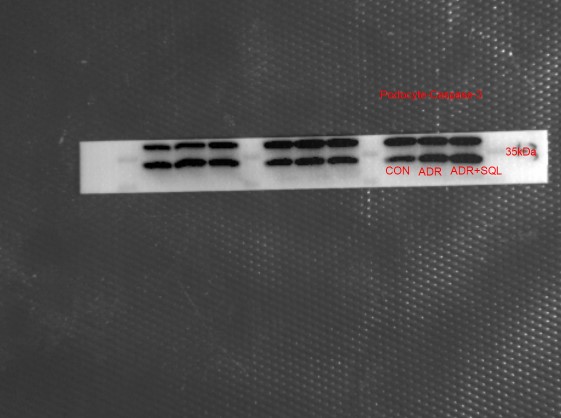

Supplement: Supplementary file 3 [file DataSheet2.zip › Original images and results for Figure 7/Fig. 7A/Caspase-3/Fig 7A-Podocyte-Caspase-3 2-merge.jpg]

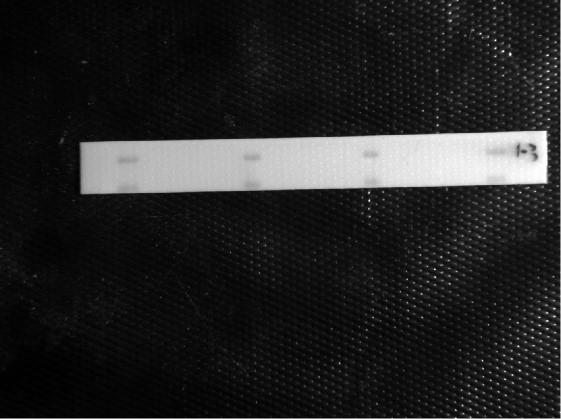

Supplement: Supplementary file 3 [file DataSheet2.zip › Original images and results for Figure 7/Fig. 7A/Caspase-3/Fig 7A-Podocyte-Caspase-3 2-white light.jpg]

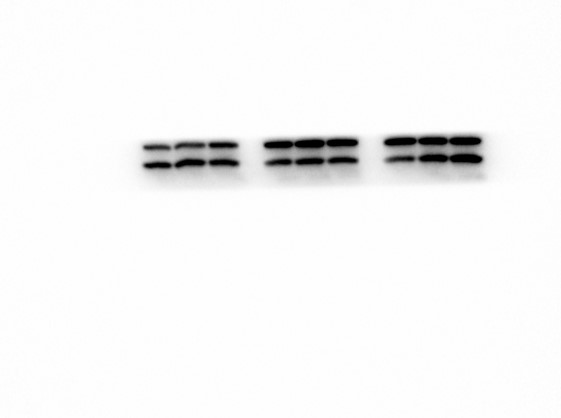

Supplement: Supplementary file 3 [file DataSheet2.zip › Original images and results for Figure 7/Fig. 7A/Caspase-3/Fig 7A-Podocyte-Caspase-3 2.jpg]

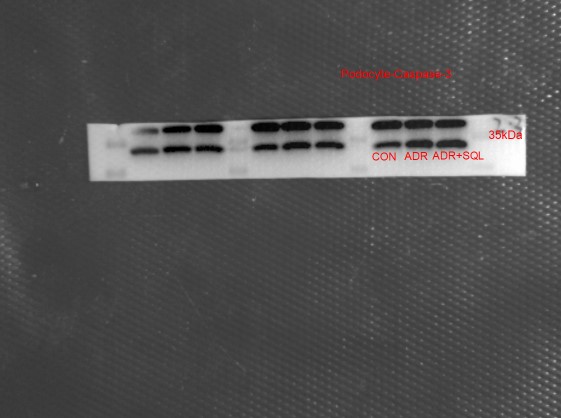

Supplement: Supplementary file 3 [file DataSheet2.zip › Original images and results for Figure 7/Fig. 7A/Caspase-3/Fig 7A-Podocyte-Caspase-3 3-merge.jpg]

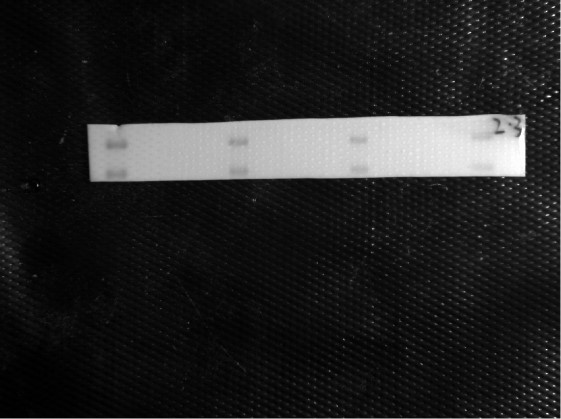

Supplement: Supplementary file 3 [file DataSheet2.zip › Original images and results for Figure 7/Fig. 7A/Caspase-3/Fig 7A-Podocyte-Caspase-3 3-white light.jpg]

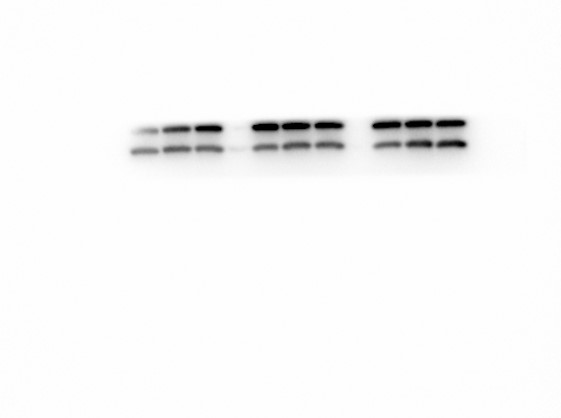

Supplement: Supplementary file 3 [file DataSheet2.zip › Original images and results for Figure 7/Fig. 7A/Caspase-3/Fig 7A-Podocyte-Caspase-3 3.jpg]

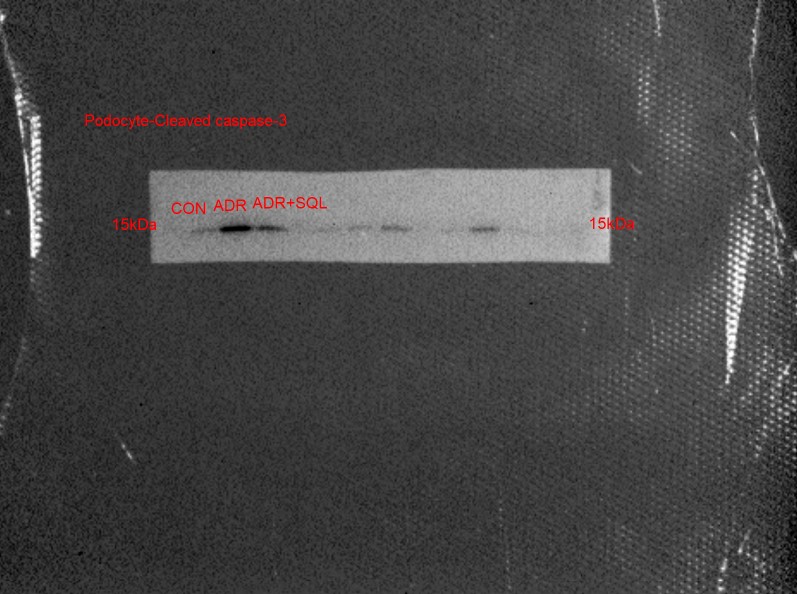

Supplement: Supplementary file 3 [file DataSheet2.zip › Original images and results for Figure 7/Fig. 7A/Cleaved caspase-3/Fig 7A-Podocyte-Cleaved caspase-3 1-merge image in Fig. 7A.jpg]

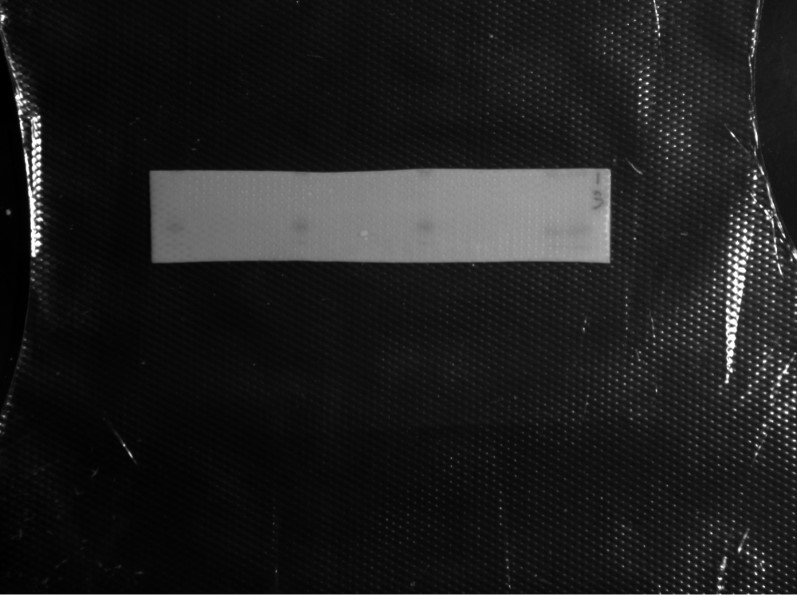

Supplement: Supplementary file 3 [file DataSheet2.zip › Original images and results for Figure 7/Fig. 7A/Cleaved caspase-3/Fig 7A-Podocyte-Cleaved caspase-3 1-white light.jpg]

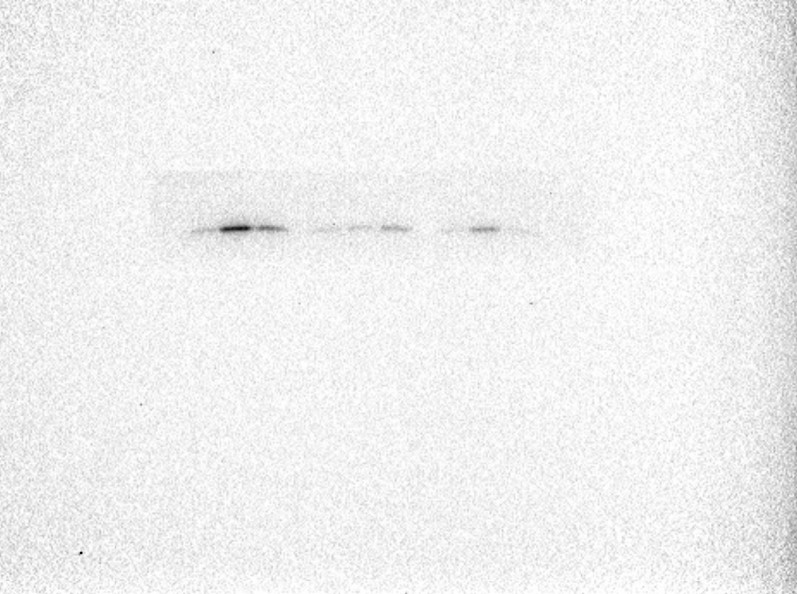

Supplement: Supplementary file 3 [file DataSheet2.zip › Original images and results for Figure 7/Fig. 7A/Cleaved caspase-3/Fig 7A-Podocyte-Cleaved caspase-3 1.jpg]

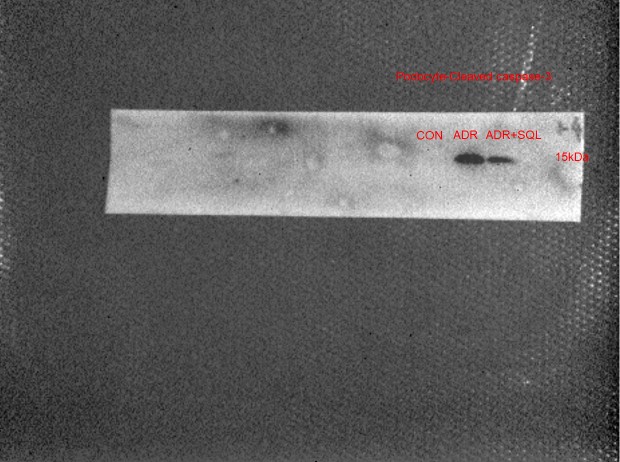

Supplement: Supplementary file 3 [file DataSheet2.zip › Original images and results for Figure 7/Fig. 7A/Cleaved caspase-3/Fig 7A-Podocyte-Cleaved caspase-3 2-merge.jpg]

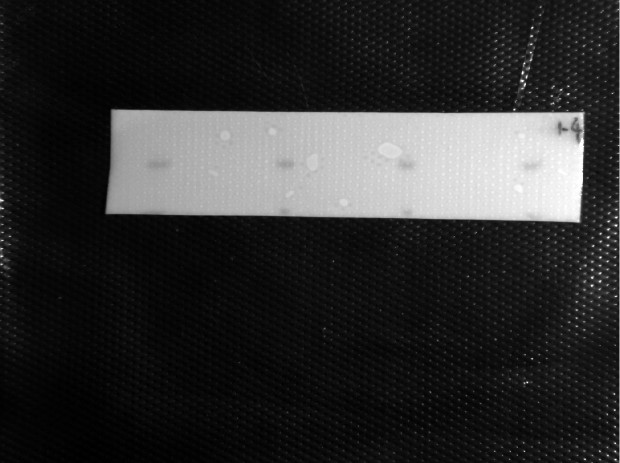

Supplement: Supplementary file 3 [file DataSheet2.zip › Original images and results for Figure 7/Fig. 7A/Cleaved caspase-3/Fig 7A-Podocyte-Cleaved caspase-3 2-white light.jpg]

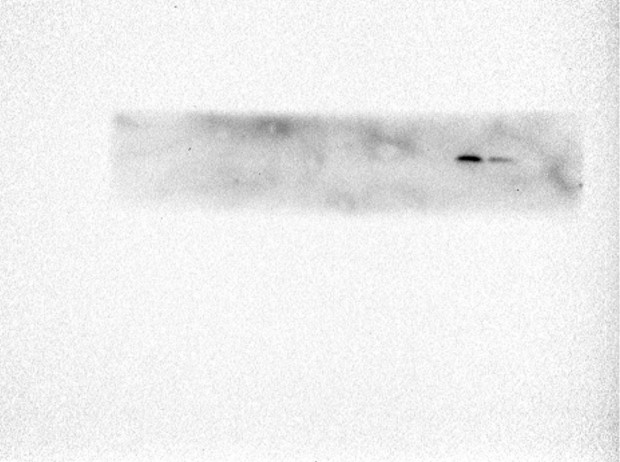

Supplement: Supplementary file 3 [file DataSheet2.zip › Original images and results for Figure 7/Fig. 7A/Cleaved caspase-3/Fig 7A-Podocyte-Cleaved caspase-3 2.jpg]

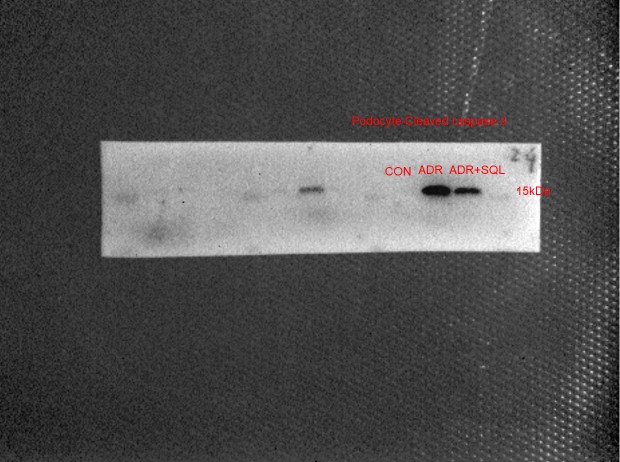

Supplement: Supplementary file 3 [file DataSheet2.zip › Original images and results for Figure 7/Fig. 7A/Cleaved caspase-3/Fig 7A-Podocyte-Cleaved caspase-3 3-merge.jpg]

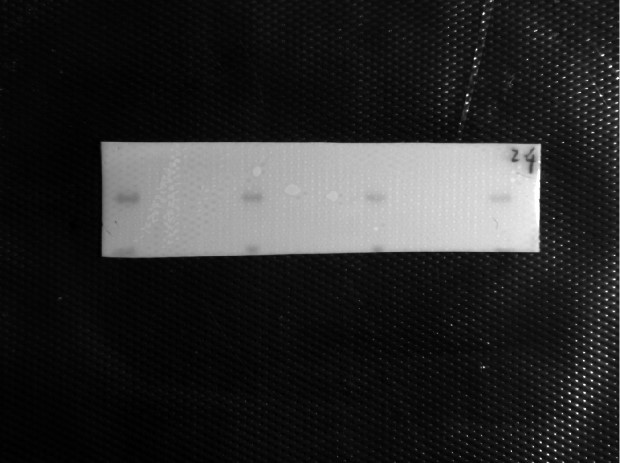

Supplement: Supplementary file 3 [file DataSheet2.zip › Original images and results for Figure 7/Fig. 7A/Cleaved caspase-3/Fig 7A-Podocyte-Cleaved caspase-3 3-white light.jpg]

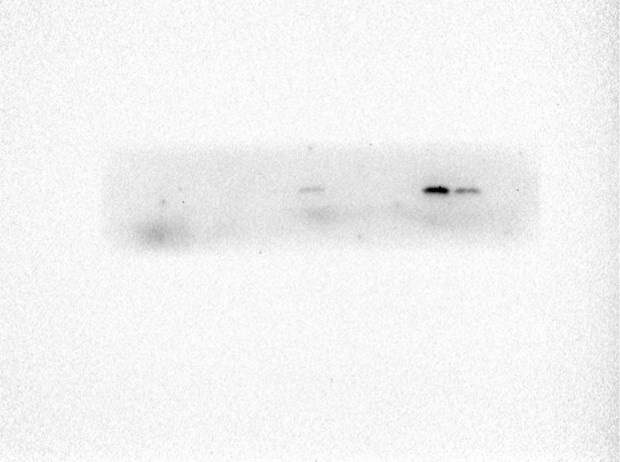

Supplement: Supplementary file 3 [file DataSheet2.zip › Original images and results for Figure 7/Fig. 7A/Cleaved caspase-3/Fig 7A-Podocyte-Cleaved caspase-3 3.jpg]

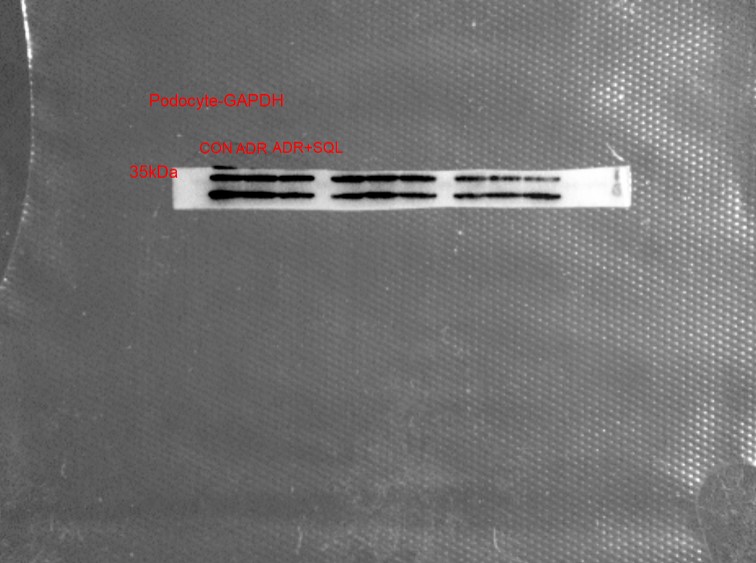

Supplement: Supplementary file 3 [file DataSheet2.zip › Original images and results for Figure 7/Fig. 7A/GAPDH/Fig 7A-Podocyte-GAPDH 1-merge.jpg]

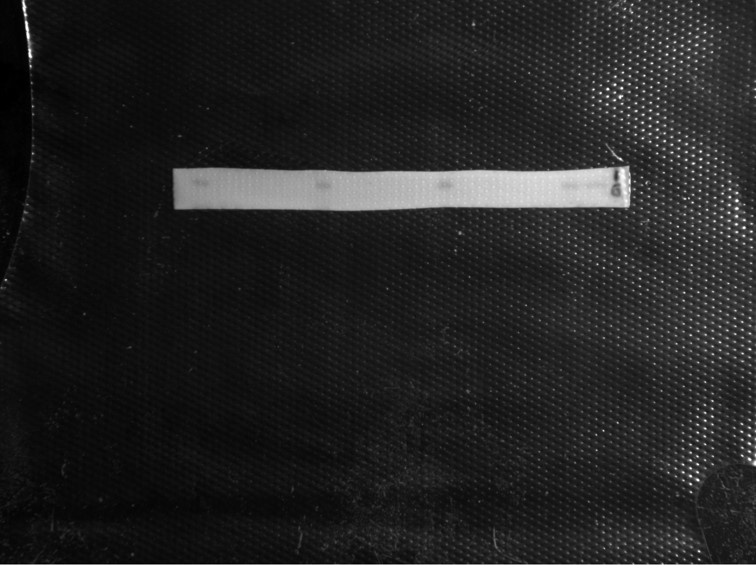

Supplement: Supplementary file 3 [file DataSheet2.zip › Original images and results for Figure 7/Fig. 7A/GAPDH/Fig 7A-Podocyte-GAPDH 1-white light.jpg]

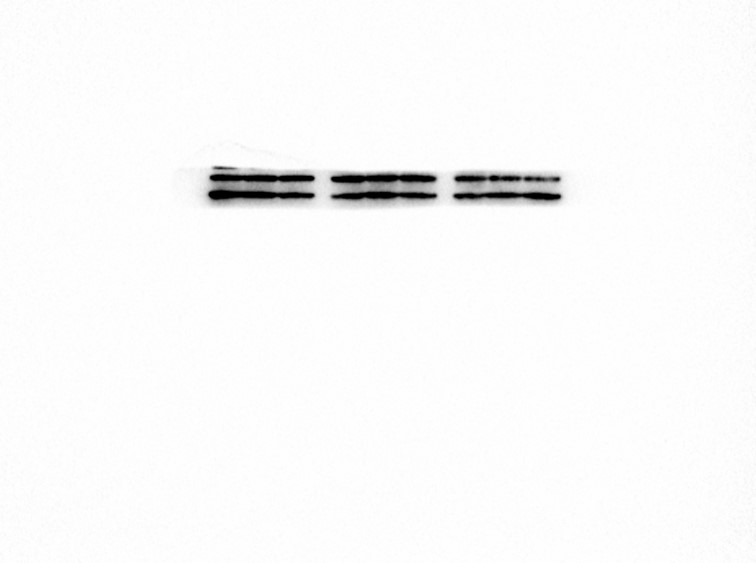

Supplement: Supplementary file 3 [file DataSheet2.zip › Original images and results for Figure 7/Fig. 7A/GAPDH/Fig 7A-Podocyte-GAPDH 1.jpg]

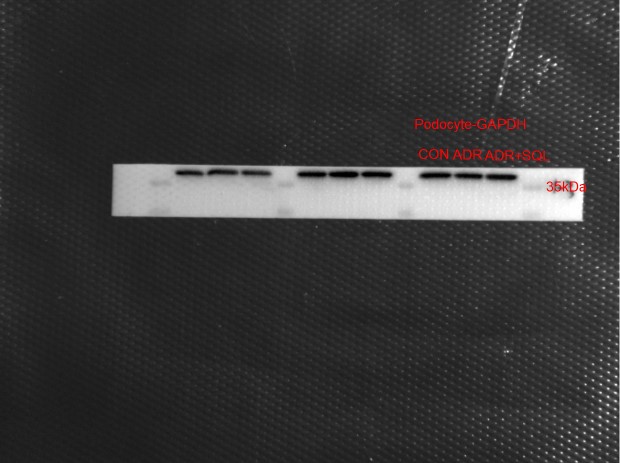

Supplement: Supplementary file 3 [file DataSheet2.zip › Original images and results for Figure 7/Fig. 7A/GAPDH/Fig 7A-Podocyte-GAPDH 2-merge image in Fig. 7A.jpg]

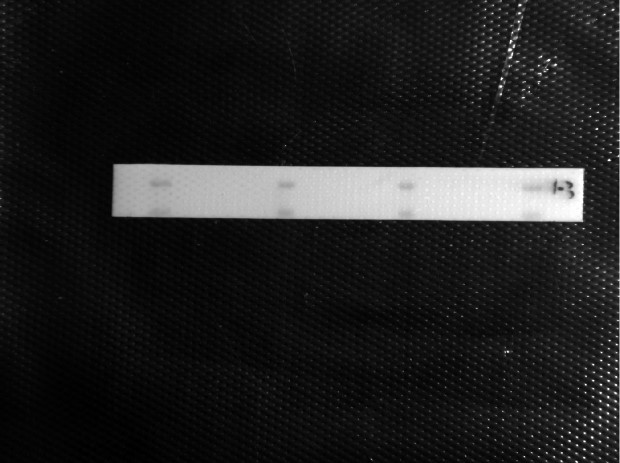

Supplement: Supplementary file 3 [file DataSheet2.zip › Original images and results for Figure 7/Fig. 7A/GAPDH/Fig 7A-Podocyte-GAPDH 2-white light.jpg]

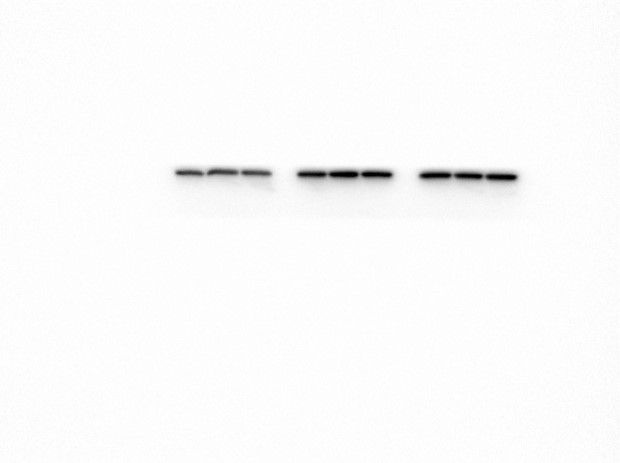

Supplement: Supplementary file 3 [file DataSheet2.zip › Original images and results for Figure 7/Fig. 7A/GAPDH/Fig 7A-Podocyte-GAPDH 2.jpg]

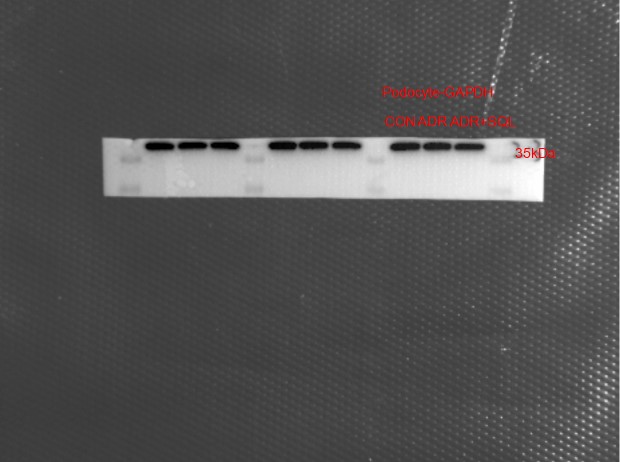

Supplement: Supplementary file 3 [file DataSheet2.zip › Original images and results for Figure 7/Fig. 7A/GAPDH/Fig 7A-Podocyte-GAPDH 3-merge.jpg]

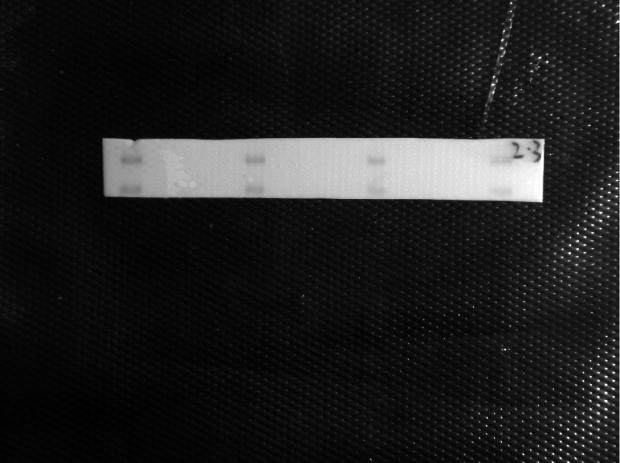

Supplement: Supplementary file 3 [file DataSheet2.zip › Original images and results for Figure 7/Fig. 7A/GAPDH/Fig 7A-Podocyte-GAPDH 3-white light.jpg]

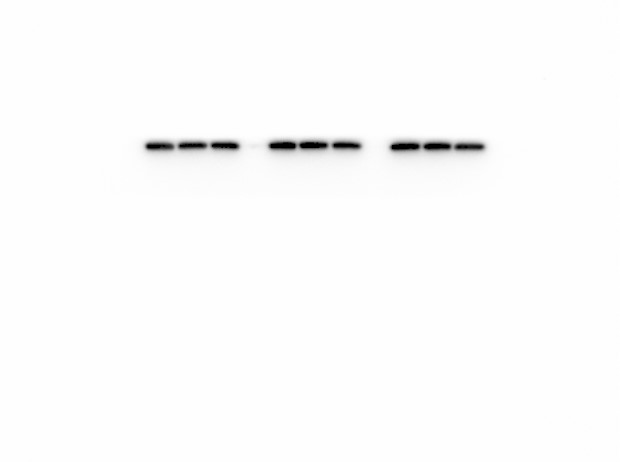

Supplement: Supplementary file 3 [file DataSheet2.zip › Original images and results for Figure 7/Fig. 7A/GAPDH/Fig 7A-Podocyte-GAPDH 3.jpg]

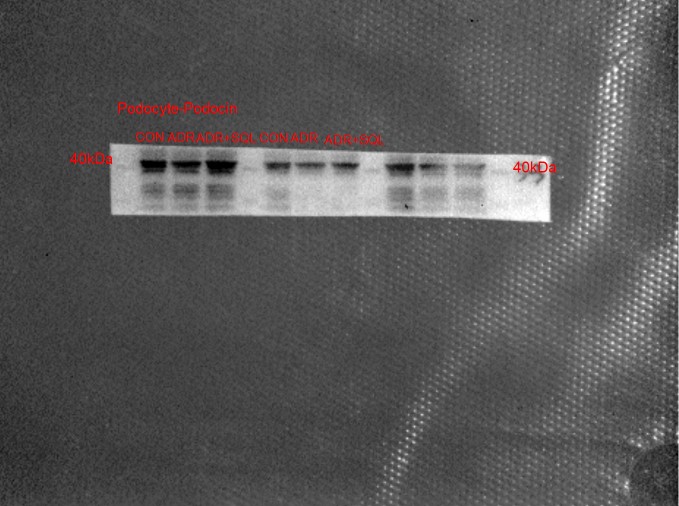

Supplement: Supplementary file 3 [file DataSheet2.zip › Original images and results for Figure 7/Fig. 7A/Podocin/Fig 7A-Podocyte-Podocin 1 2-merge right-image in Fig. 7A.jpg]

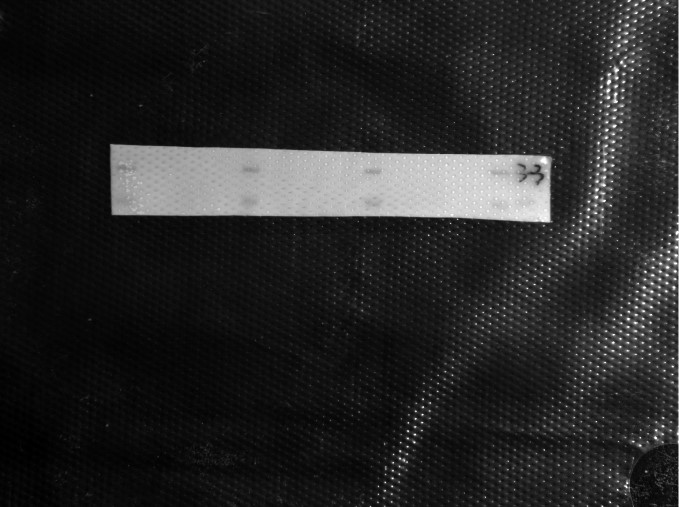

Supplement: Supplementary file 3 [file DataSheet2.zip › Original images and results for Figure 7/Fig. 7A/Podocin/Fig 7A-Podocyte-Podocin 1 2-white light.jpg]

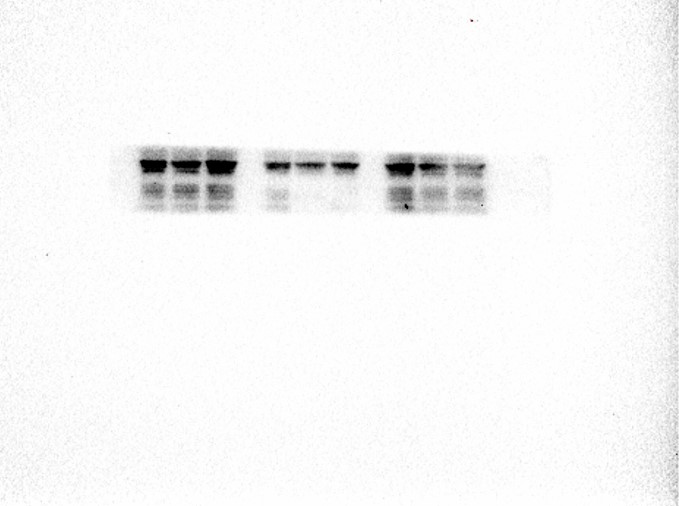

Supplement: Supplementary file 3 [file DataSheet2.zip › Original images and results for Figure 7/Fig. 7A/Podocin/Fig 7A-Podocyte-Podocin 1 2.jpg]

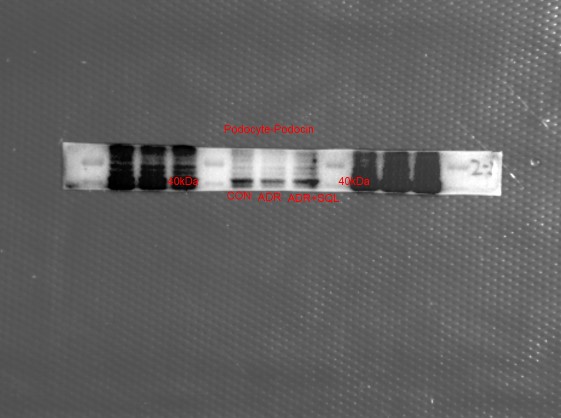

Supplement: Supplementary file 3 [file DataSheet2.zip › Original images and results for Figure 7/Fig. 7A/Podocin/Fig 7A-Podocyte-Podocin 3-merge.jpg]

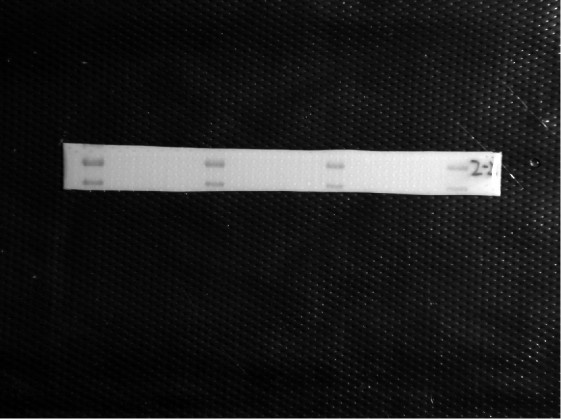

Supplement: Supplementary file 3 [file DataSheet2.zip › Original images and results for Figure 7/Fig. 7A/Podocin/Fig 7A-Podocyte-Podocin 3-white light.jpg]

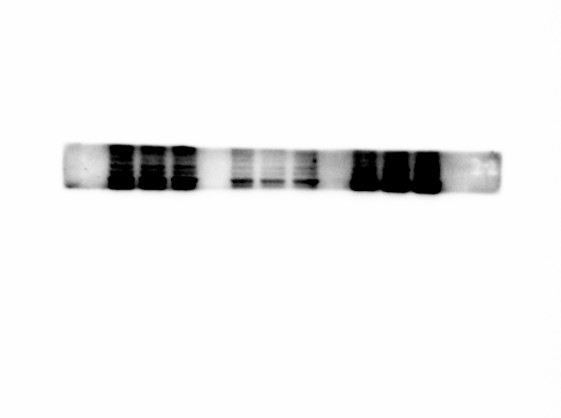

Supplement: Supplementary file 3 [file DataSheet2.zip › Original images and results for Figure 7/Fig. 7A/Podocin/Fig 7A-Podocyte-Podocin 3.jpg]

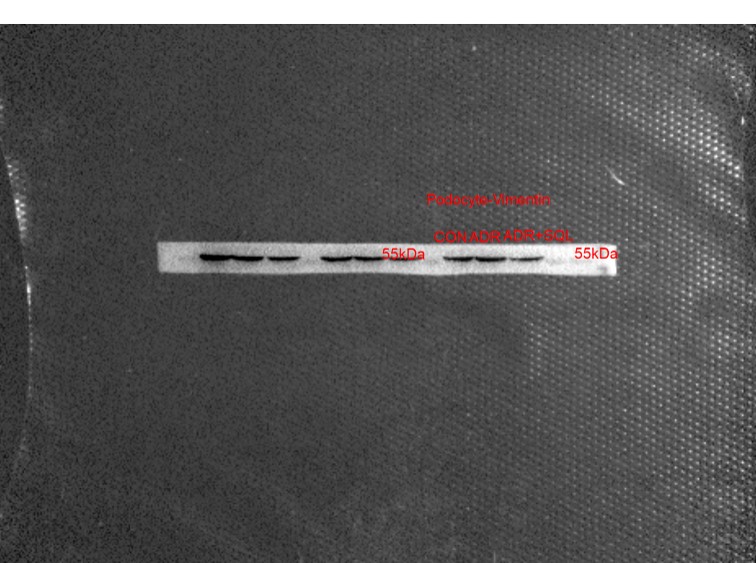

Supplement: Supplementary file 3 [file DataSheet2.zip › Original images and results for Figure 7/Fig. 7A/Vimentin/Fig 7A-Podocyte-Vimentin 1-merge image in Fig. 7A.jpg]

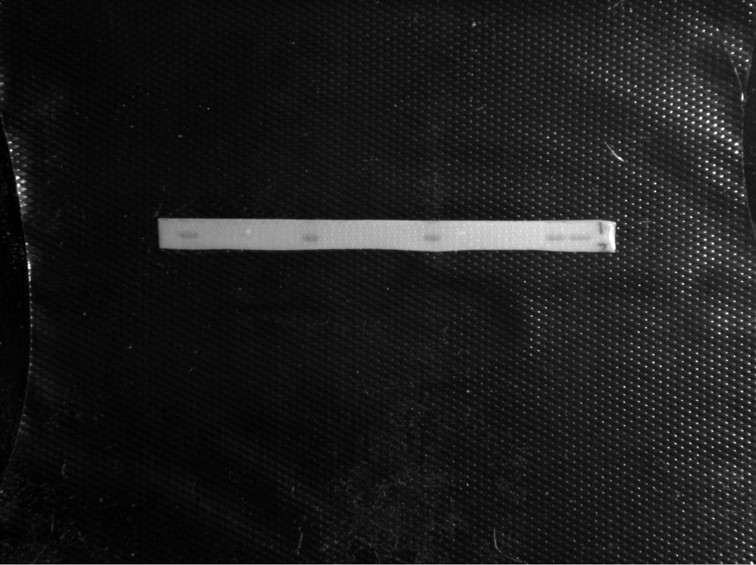

Supplement: Supplementary file 3 [file DataSheet2.zip › Original images and results for Figure 7/Fig. 7A/Vimentin/Fig 7A-Podocyte-Vimentin 1-white light.jpg]

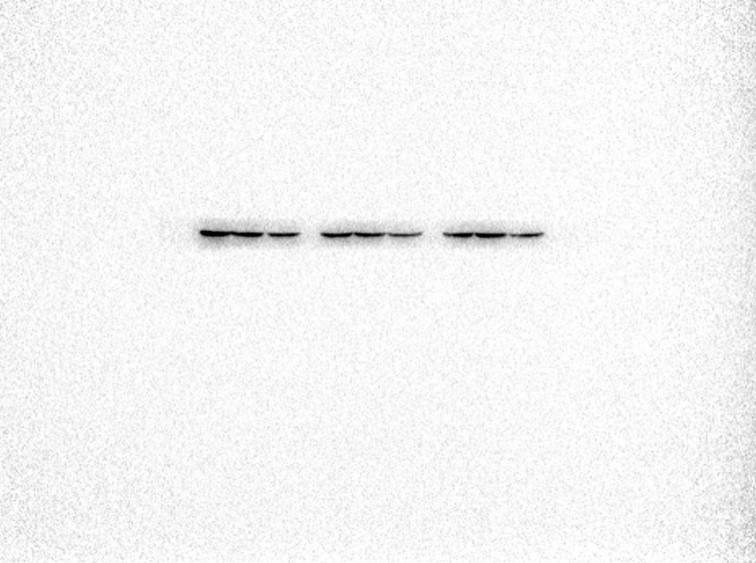

Supplement: Supplementary file 3 [file DataSheet2.zip › Original images and results for Figure 7/Fig. 7A/Vimentin/Fig 7A-Podocyte-Vimentin 1.jpg]

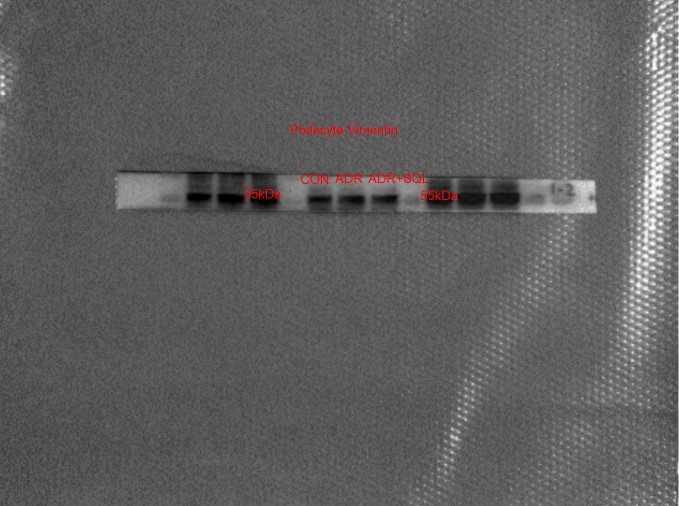

Supplement: Supplementary file 3 [file DataSheet2.zip › Original images and results for Figure 7/Fig. 7A/Vimentin/Fig 7A-Podocyte-Vimentin 2-merge.jpg]

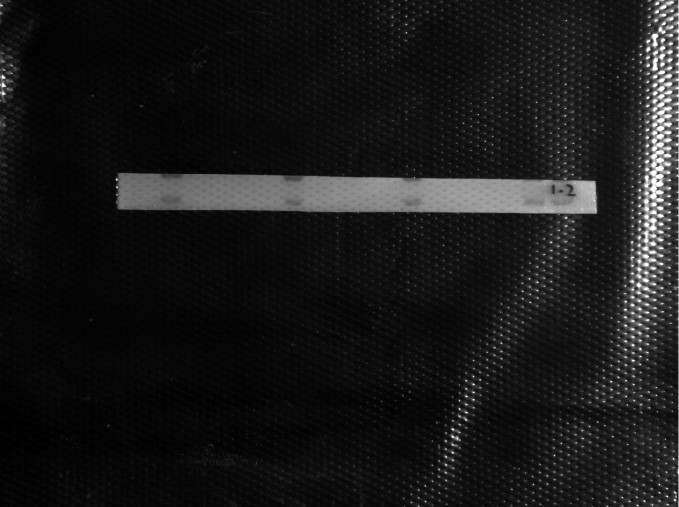

Supplement: Supplementary file 3 [file DataSheet2.zip › Original images and results for Figure 7/Fig. 7A/Vimentin/Fig 7A-Podocyte-Vimentin 2-white light.jpg]

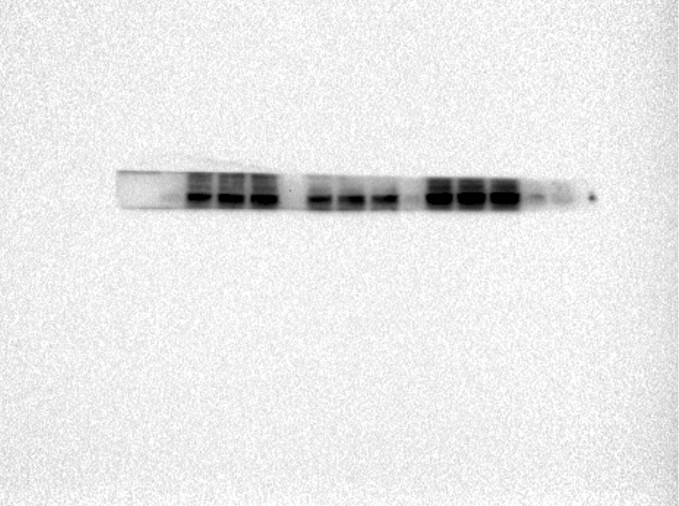

Supplement: Supplementary file 3 [file DataSheet2.zip › Original images and results for Figure 7/Fig. 7A/Vimentin/Fig 7A-Podocyte-Vimentin 2.jpg]

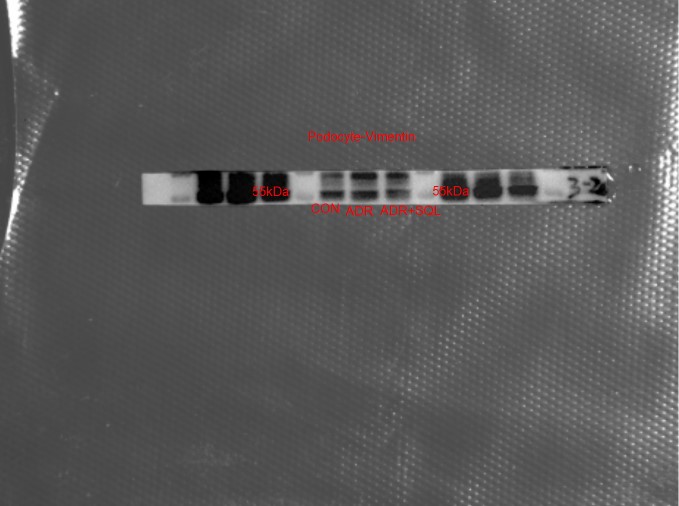

Supplement: Supplementary file 3 [file DataSheet2.zip › Original images and results for Figure 7/Fig. 7A/Vimentin/Fig 7A-Podocyte-Vimentin 3-merge.jpg]

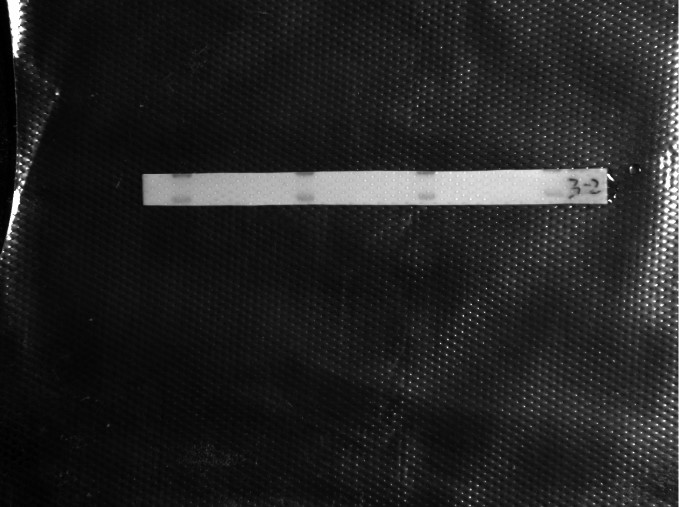

Supplement: Supplementary file 3 [file DataSheet2.zip › Original images and results for Figure 7/Fig. 7A/Vimentin/Fig 7A-Podocyte-Vimentin 3-white light.jpg]

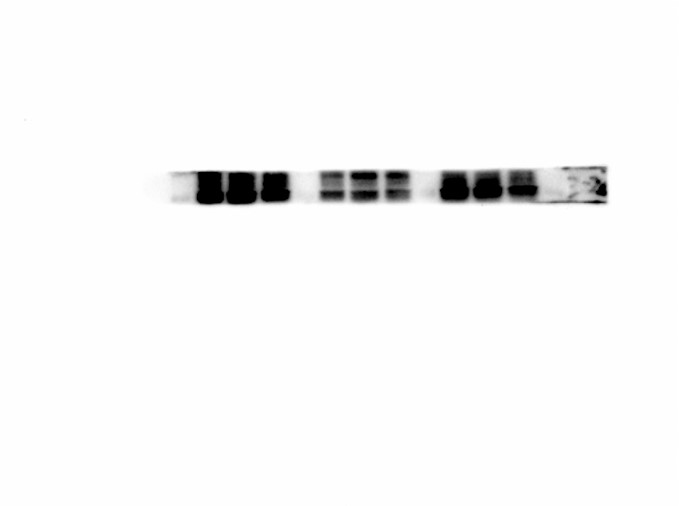

Supplement: Supplementary file 3 [file DataSheet2.zip › Original images and results for Figure 7/Fig. 7A/Vimentin/Fig 7A-Podocyte-Vimentin 3.jpg]

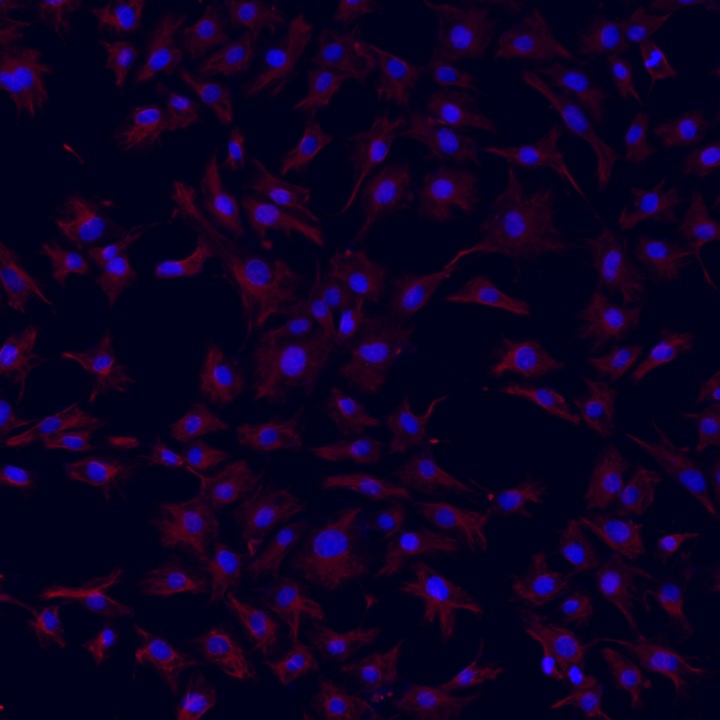

Supplement: Supplementary file 3 [file DataSheet2.zip › Original images and results for Figure 7/Fig. 7E/Fig. 7E Vimentin-IF/Vimentin-IF-ADR+SQL1-1.jpg]

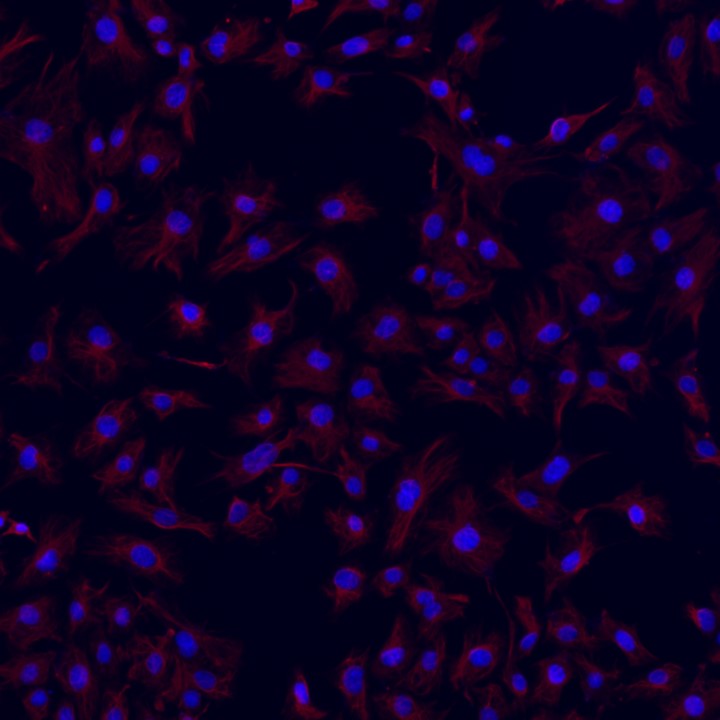

Supplement: Supplementary file 3 [file DataSheet2.zip › Original images and results for Figure 7/Fig. 7E/Fig. 7E Vimentin-IF/Vimentin-IF-ADR+SQL1-2.jpg]

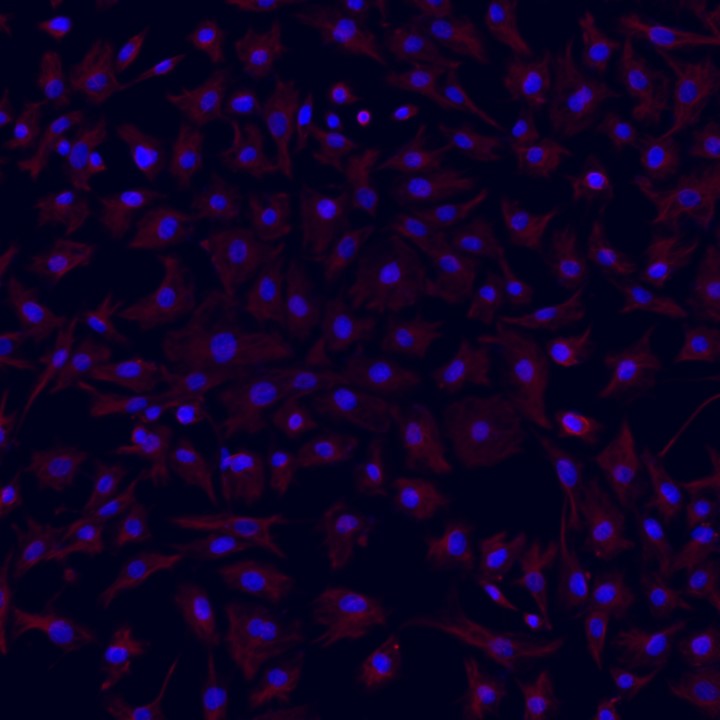

Supplement: Supplementary file 3 [file DataSheet2.zip › Original images and results for Figure 7/Fig. 7E/Fig. 7E Vimentin-IF/Vimentin-IF-ADR+SQL1-3.jpg]

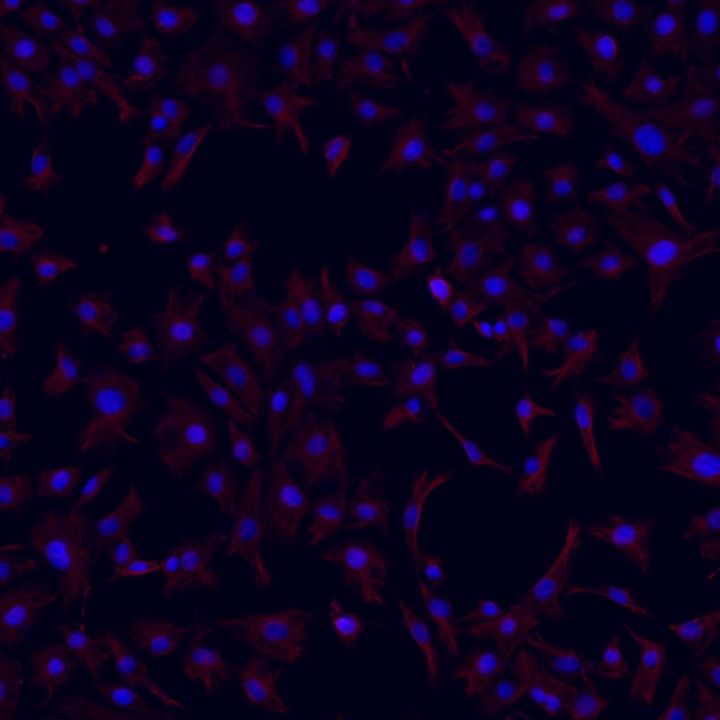

Supplement: Supplementary file 3 [file DataSheet2.zip › Original images and results for Figure 7/Fig. 7E/Fig. 7E Vimentin-IF/Vimentin-IF-ADR+SQL1-4.jpg]

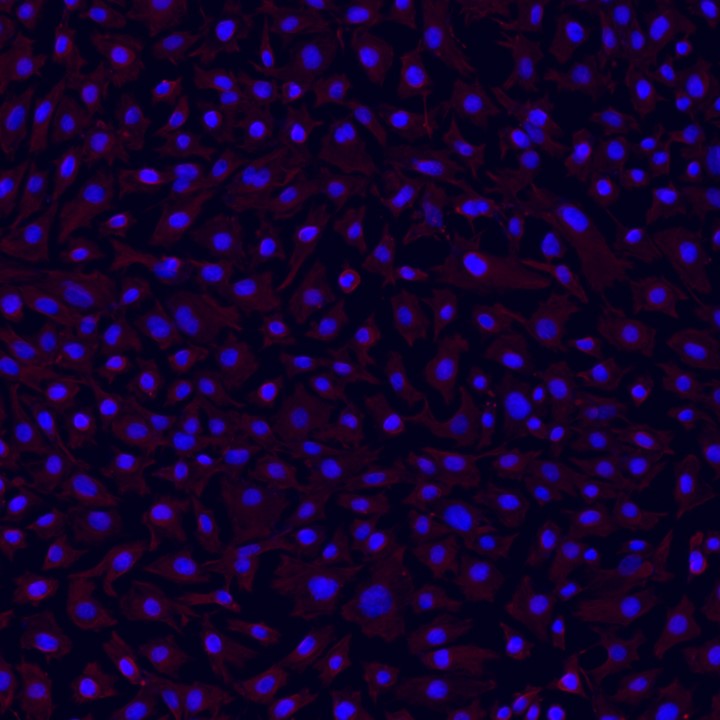

Supplement: Supplementary file 3 [file DataSheet2.zip › Original images and results for Figure 7/Fig. 7E/Fig. 7E Vimentin-IF/Vimentin-IF-ADR+SQL1-5.jpg]

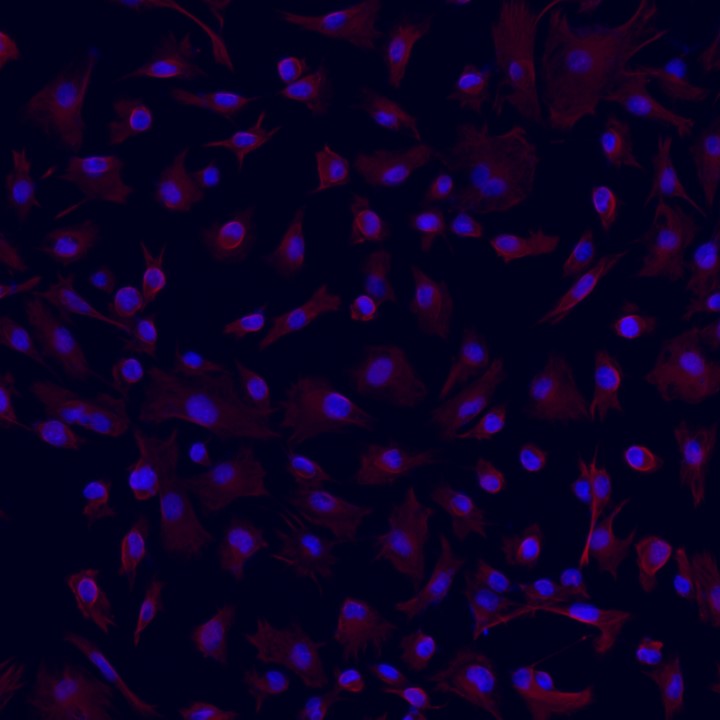

Supplement: Supplementary file 3 [file DataSheet2.zip › Original images and results for Figure 7/Fig. 7E/Fig. 7E Vimentin-IF/Vimentin-IF-ADR+SQL2-1.jpg]

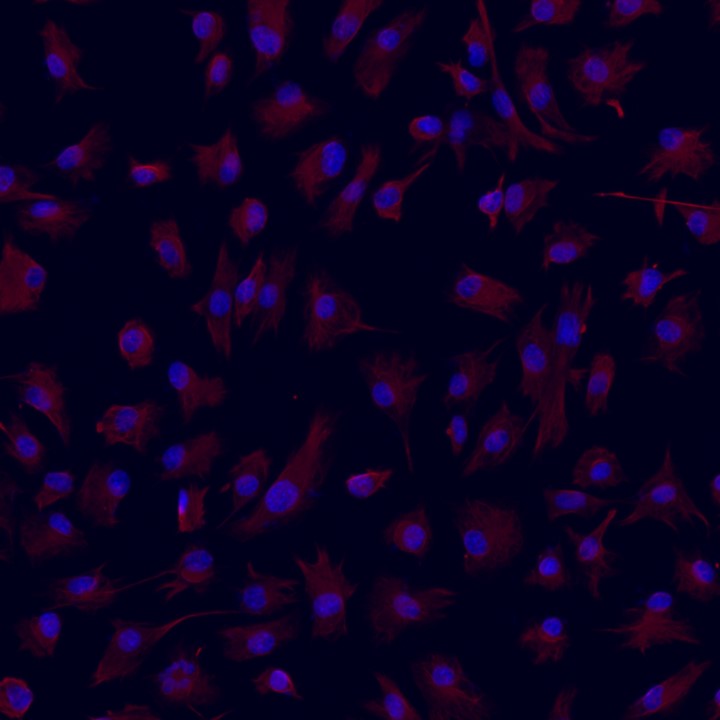

Supplement: Supplementary file 3 [file DataSheet2.zip › Original images and results for Figure 7/Fig. 7E/Fig. 7E Vimentin-IF/Vimentin-IF-ADR+SQL2-2.jpg]

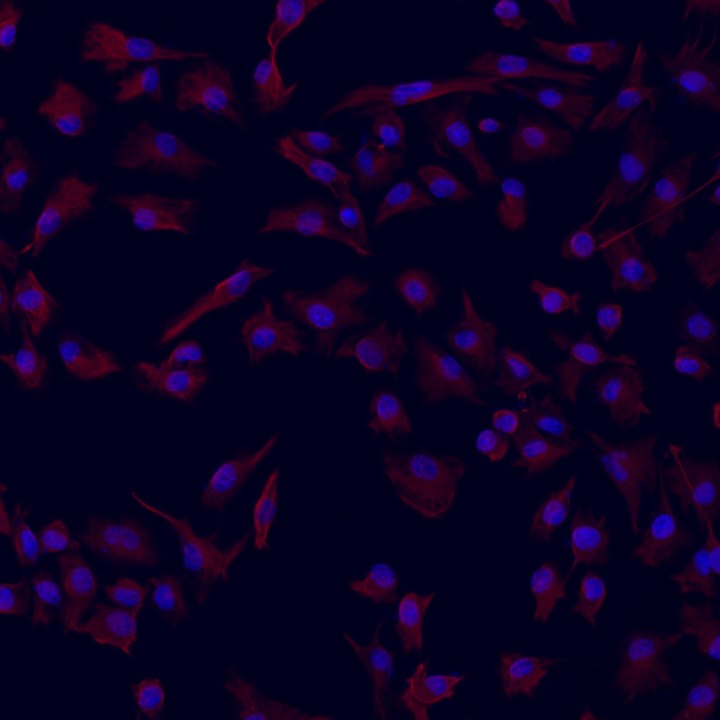

Supplement: Supplementary file 3 [file DataSheet2.zip › Original images and results for Figure 7/Fig. 7E/Fig. 7E Vimentin-IF/Vimentin-IF-ADR+SQL2-3.jpg]

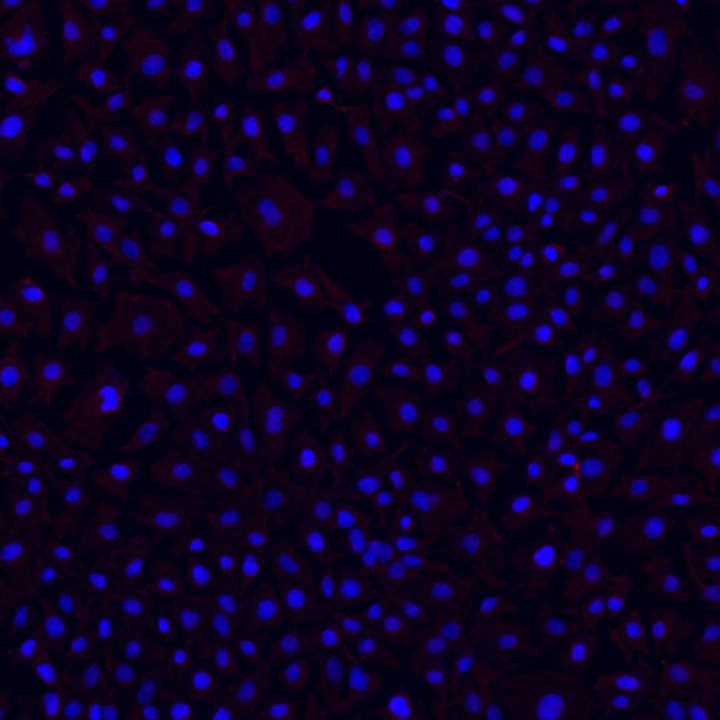

Supplement: Supplementary file 3 [file DataSheet2.zip › Original images and results for Figure 7/Fig. 7E/Fig. 7E Vimentin-IF/Vimentin-IF-ADR+SQL2-5.jpg]

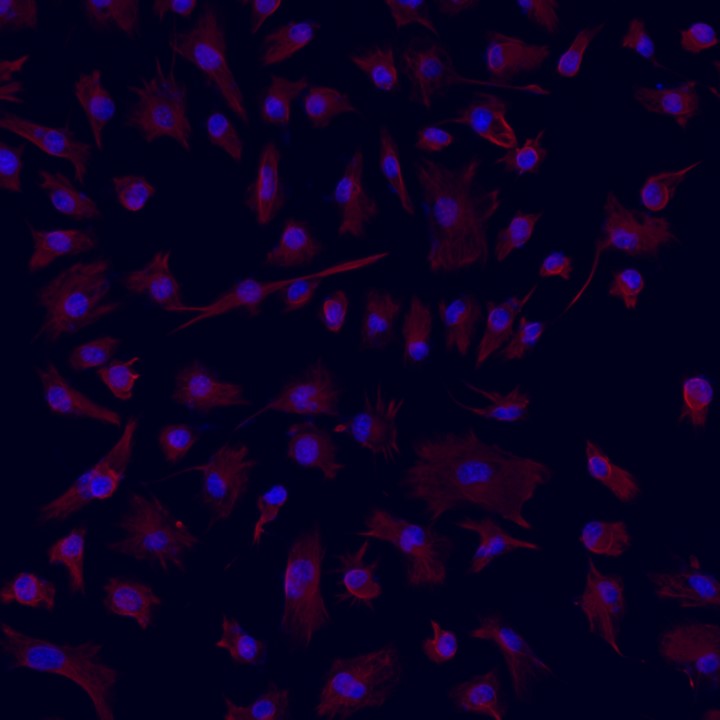

Supplement: Supplementary file 3 [file DataSheet2.zip › Original images and results for Figure 7/Fig. 7E/Fig. 7E Vimentin-IF/Vimentin-IF-ADR+SQL3-1.jpg]

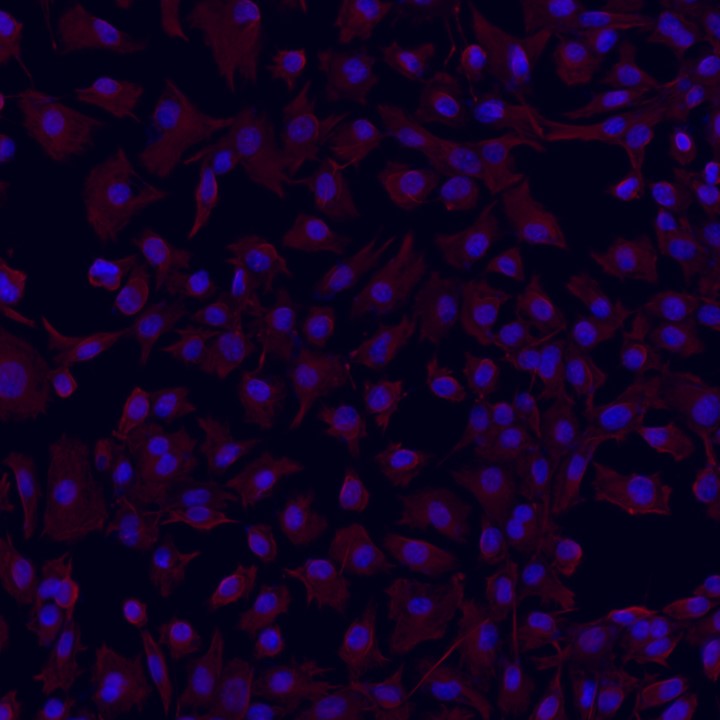

Supplement: Supplementary file 3 [file DataSheet2.zip › Original images and results for Figure 7/Fig. 7E/Fig. 7E Vimentin-IF/Vimentin-IF-ADR+SQL3-4.jpg]

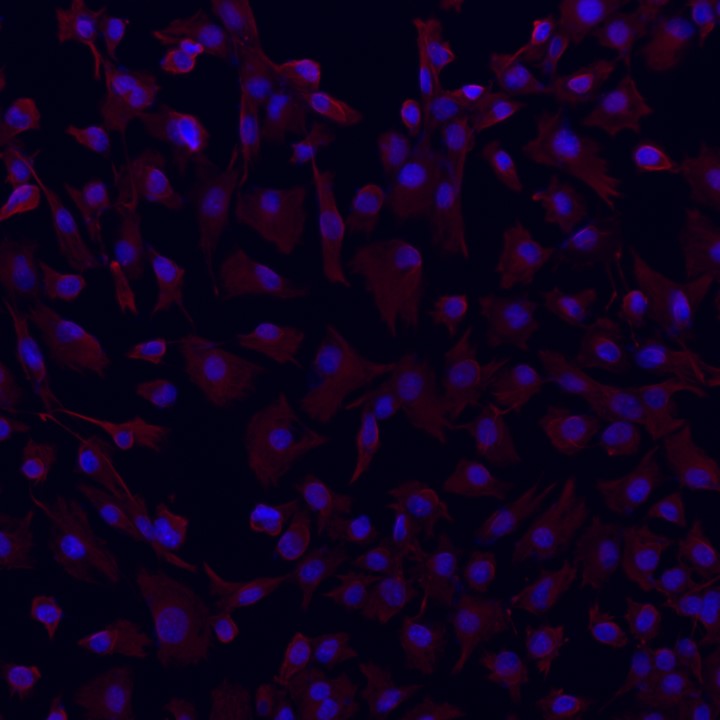

Supplement: Supplementary file 3 [file DataSheet2.zip › Original images and results for Figure 7/Fig. 7E/Fig. 7E Vimentin-IF/Vimentin-IF-ADR+SQL3-5 image in Fig. 7E.jpg]

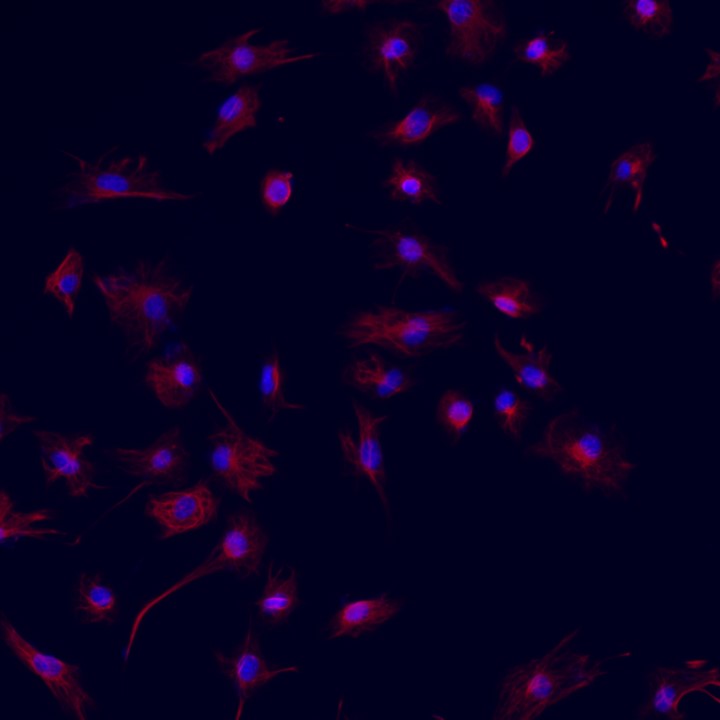

Supplement: Supplementary file 3 [file DataSheet2.zip › Original images and results for Figure 7/Fig. 7E/Fig. 7E Vimentin-IF/Vimentin-IF-ADR1-1.jpg]

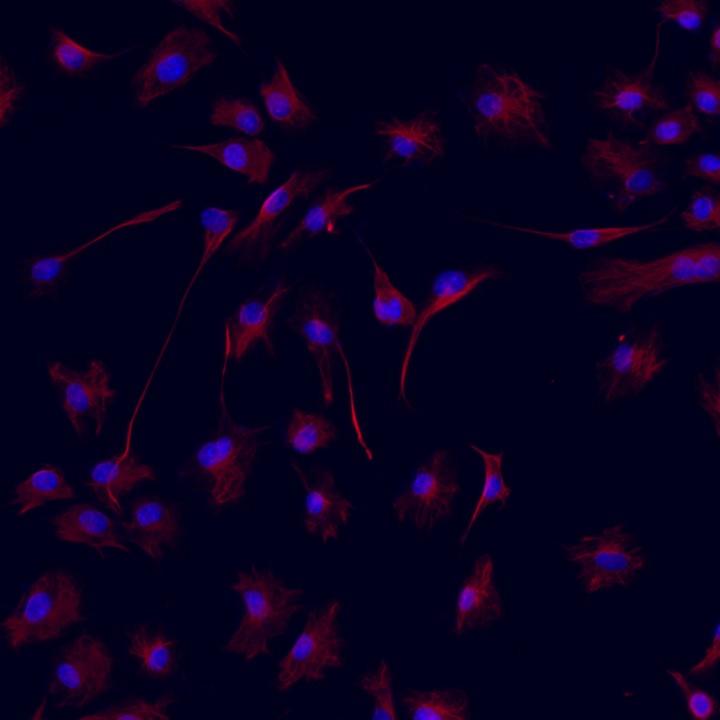

Supplement: Supplementary file 3 [file DataSheet2.zip › Original images and results for Figure 7/Fig. 7E/Fig. 7E Vimentin-IF/Vimentin-IF-ADR1-2.jpg]

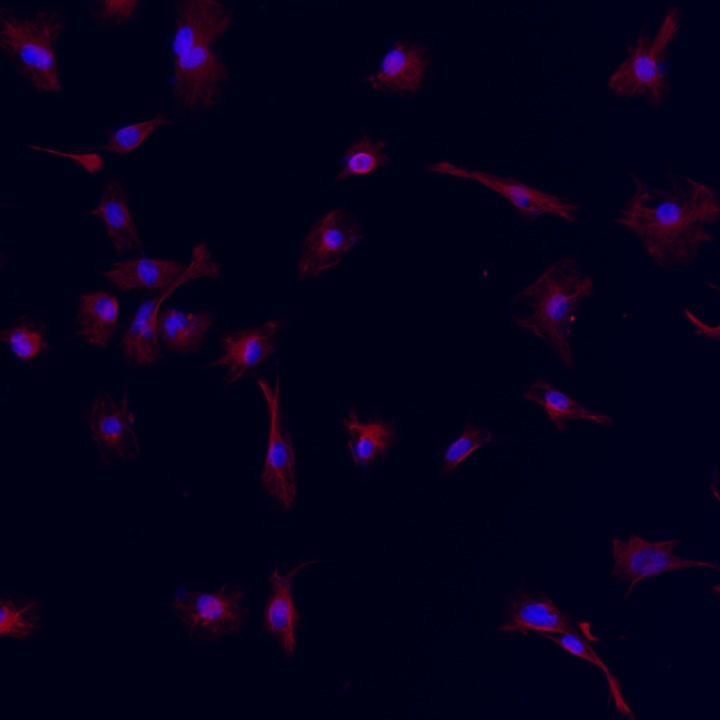

Supplement: Supplementary file 3 [file DataSheet2.zip › Original images and results for Figure 7/Fig. 7E/Fig. 7E Vimentin-IF/Vimentin-IF-ADR1-3.jpg]

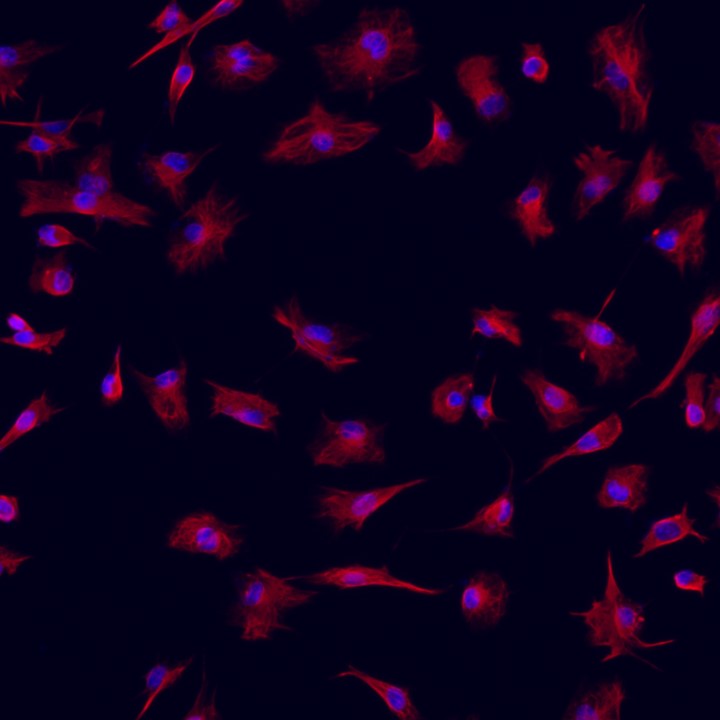

Supplement: Supplementary file 3 [file DataSheet2.zip › Original images and results for Figure 7/Fig. 7E/Fig. 7E Vimentin-IF/Vimentin-IF-ADR1-4.jpg]

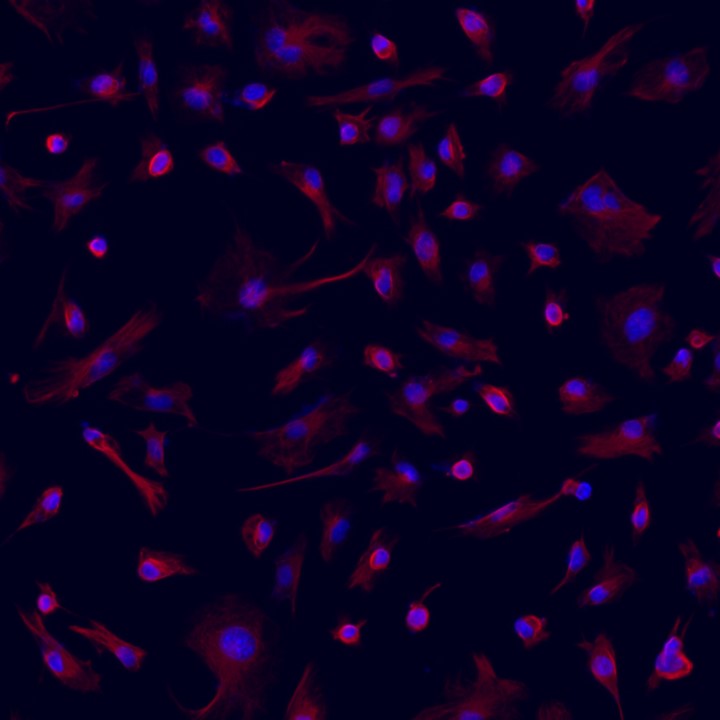

Supplement: Supplementary file 3 [file DataSheet2.zip › Original images and results for Figure 7/Fig. 7E/Fig. 7E Vimentin-IF/Vimentin-IF-ADR1-5.jpg]

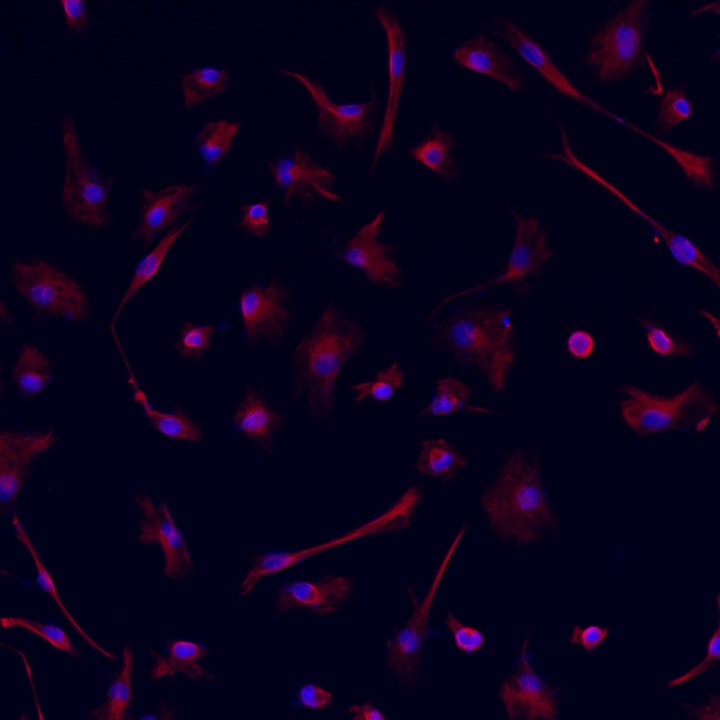

Supplement: Supplementary file 3 [file DataSheet2.zip › Original images and results for Figure 7/Fig. 7E/Fig. 7E Vimentin-IF/Vimentin-IF-ADR2-1.jpg]

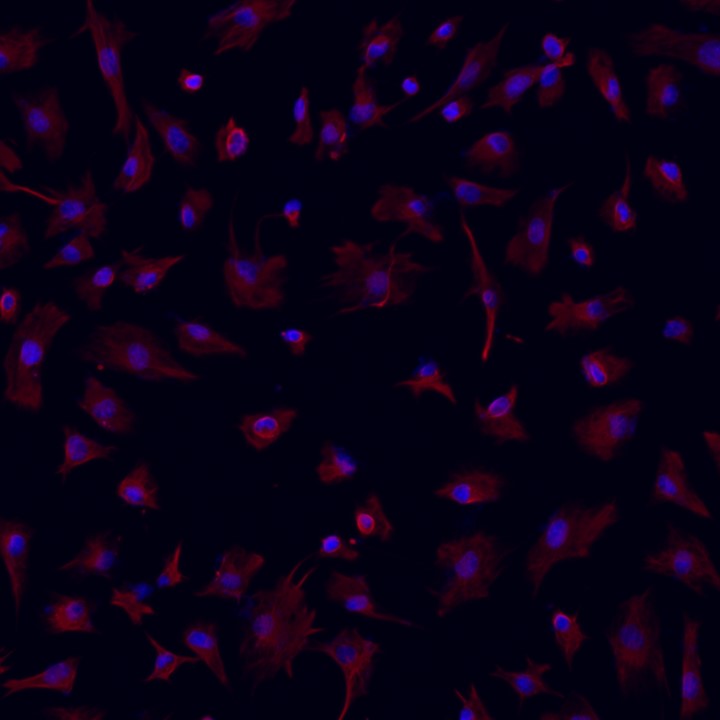

Supplement: Supplementary file 3 [file DataSheet2.zip › Original images and results for Figure 7/Fig. 7E/Fig. 7E Vimentin-IF/Vimentin-IF-ADR2-2.jpg]

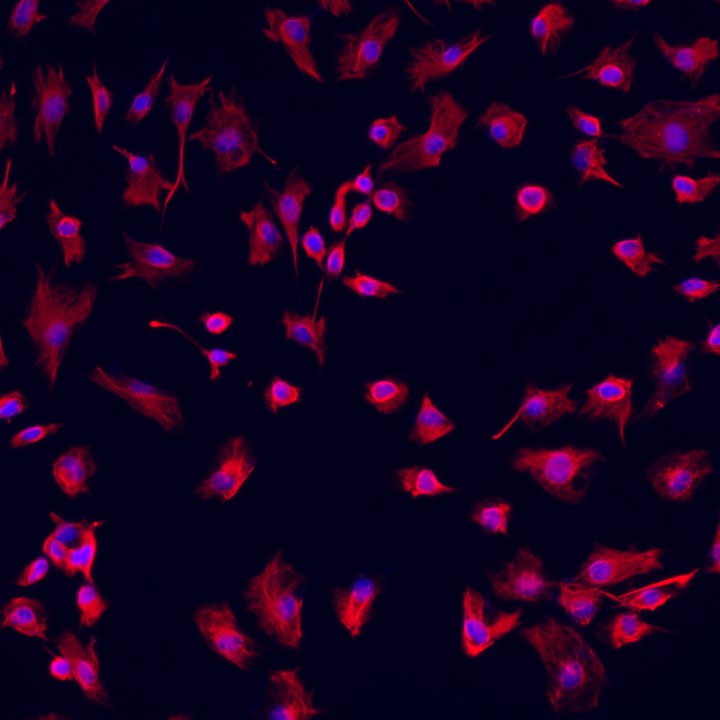

Supplement: Supplementary file 3 [file DataSheet2.zip › Original images and results for Figure 7/Fig. 7E/Fig. 7E Vimentin-IF/Vimentin-IF-ADR2-3.jpg]

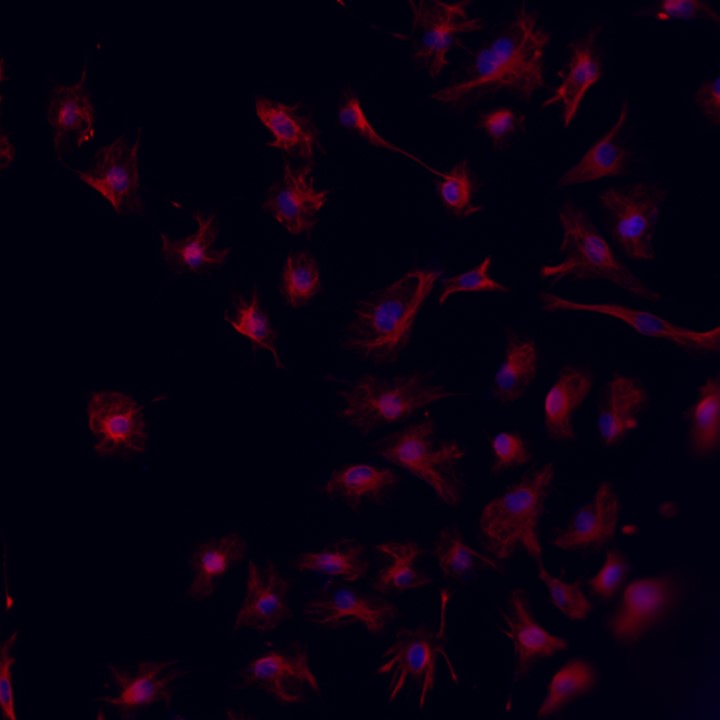

Supplement: Supplementary file 3 [file DataSheet2.zip › Original images and results for Figure 7/Fig. 7E/Fig. 7E Vimentin-IF/Vimentin-IF-ADR2-4.jpg]

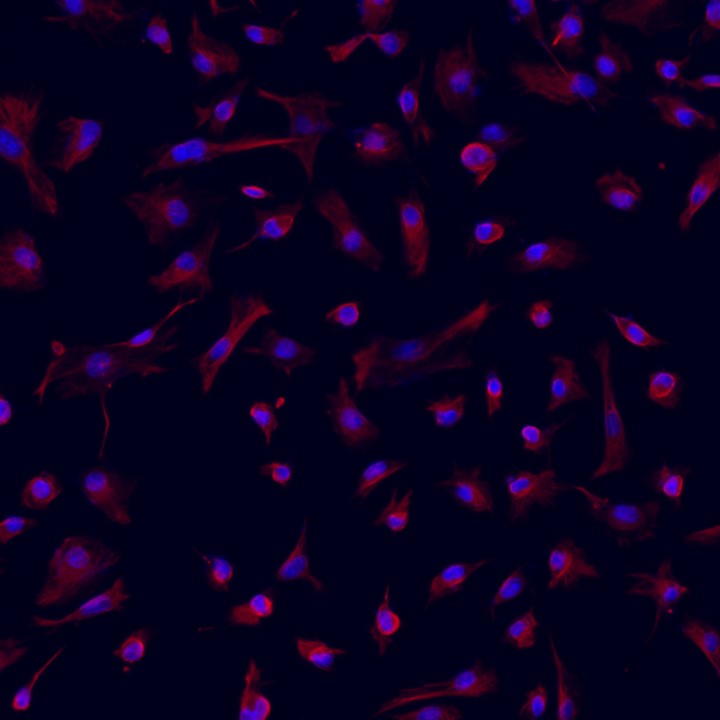

Supplement: Supplementary file 3 [file DataSheet2.zip › Original images and results for Figure 7/Fig. 7E/Fig. 7E Vimentin-IF/Vimentin-IF-ADR2-5.jpg]

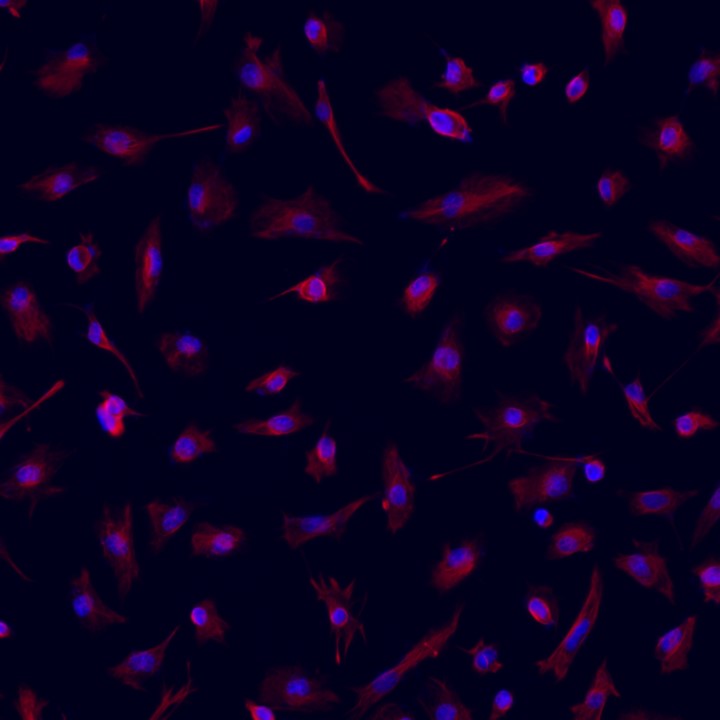

Supplement: Supplementary file 3 [file DataSheet2.zip › Original images and results for Figure 7/Fig. 7E/Fig. 7E Vimentin-IF/Vimentin-IF-ADR3-1.jpg]
