# Supplementary material for: Sanqi oral solution alleviates podocyte apoptosis in experimental membranous nephropathy by mediating EMT through the ERK/CK2-α/β-catenin pathway
Source: Front Pharmacol. 2025 May 9;16:1503961. doi: 10.3389/fphar.2025.1503961 (PMC12098599; doi:10.3389/fphar.2025.1503961)
Supplement: Supplementary file 4 [file Supplementaryfile1.doc]

**Supplementary materials**

**Part 1**

**Preparation and Chemical Profiles of SQ**

**Materials and Methods**

***Preparation of SQ***

SQ (Batch No. 210102; Cantonese medicine ratification No. Z20071155) was obtained from Guangdong Provincial Hospital of Chinese Medicine and extracted from *Astragalus mongholicus Bunge* and *Panax notoginseng (Burkill) F.H. Chen* by Water extraction alcohol precipitation method according to the Chinese Pharmacopoeia.

***Components Analysis of SQ***

The preparation process was implemented refer to our published article(Tian et al., 2020). An Agilent 1200 HPLC system with DAD detector was adopted for quality control analysis of SQ. Briefly, the LC separation was performed over a Kinetex C18 column (4.6 × 100 mm, 2.6 mm, Phenomenex Inc., Torrance, USA) at 30 °C. Samples were eluted by gradients in line with the program of elution as: 0–12 min, 88–80% A; 12–26 min, 80–74% A; 26–40 min, 74 35% A. The UV detection wavelengths were set at 205 and 284 nm.

**Results**

***Component of SQ***

As shown in Supplementary Table S1, the concentration of *Astragalus mongholicus Bunge* and *Panax notoginseng (Burkill) F.H. Chen* in SQ was 0.333 and 0.056 g/mL, respectively.

***Chemical Profiles of SQ***

As shown in Supplementary Figure S1, 11 chemical profiles of SQ were identified as follows: (1) caylcosin-7-O-β-D-glucopyranoside, (2) ononin, (3) 6aR, 11aR-3-hydroxy-9,10-dimethoxypterocarpan-3-O-β-D- glucopyranoside, (4) calycosin, (5) notoginsenoside R1, (6) isomucronulatol-7-O-β-D-glucopyranoside, (7) ginsenoside Rg1, (8) ginsenoside Re, (9) formononetin, (10) ginsenoside Rb1, (11) ginsenoside Rd.

**Table**

**Supplementary Table 1.** Component of SQ (三芪口服液).

| **Plant name** | **Chinese name** | **Concentration** (crude drug) |
| --- | --- | --- |
| *Astragalus mongholicus Bunge* | Huangqi (黄芪) | 0.333 g/mL |
| *Panax notoginseng (Burkill) F.H. Chen* | Sanqi (三七) | 0.056 g/mL |

**Figure**


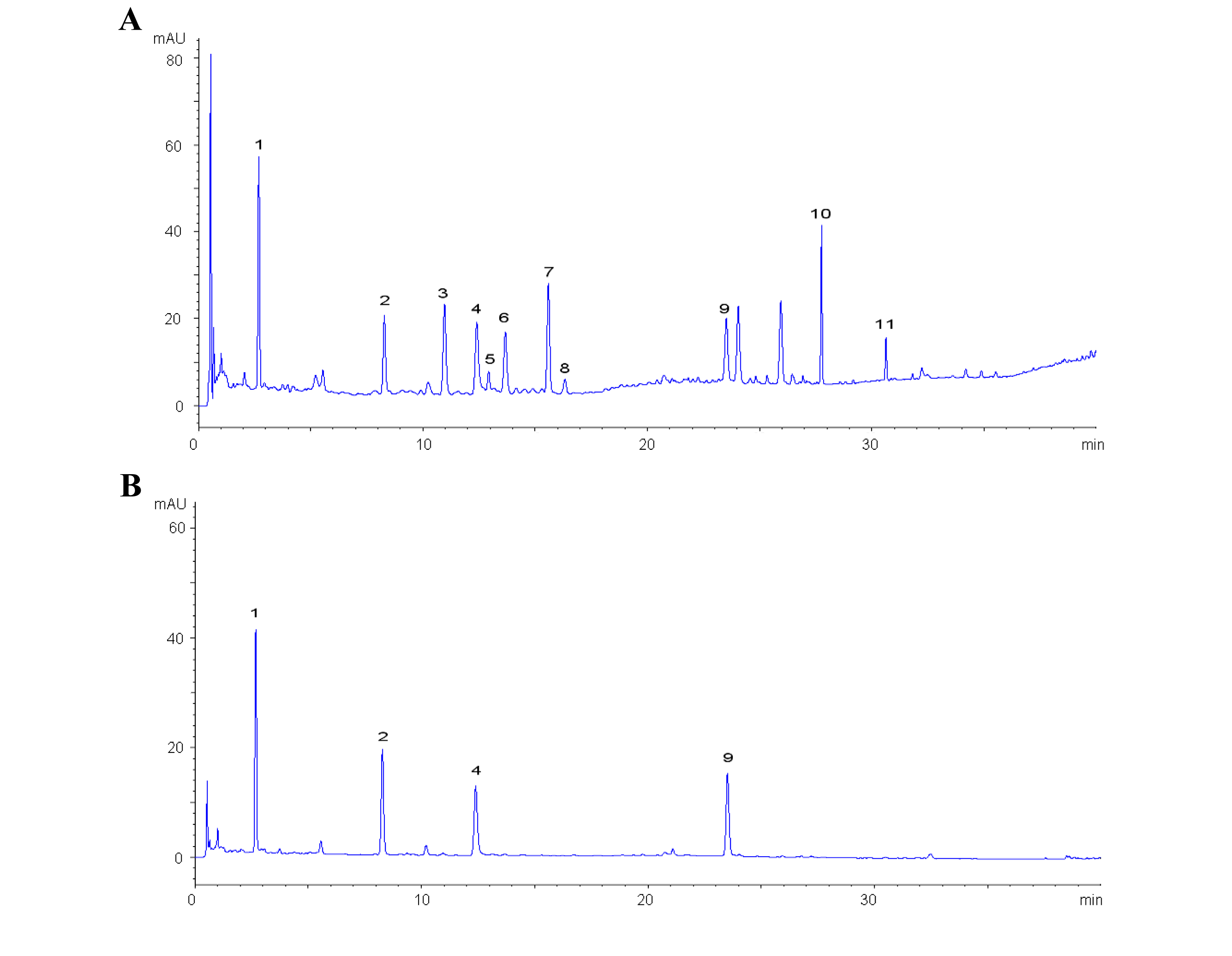


**Supplementary Fig. 1.** Chemical profiles of SQ. UV chromatogram of SQ at 205 nm (A) and 284 nm (B) was presented. 11 peaks were identified as follows: (1) caylcosin-7-O-β-D-glucopyranoside, (2) ononin, (3) 6aR, 11aR-3-hydroxy-9,10-dimethoxypterocarpan-3-O-β-D- glucopyranoside, (4) calycosin, (5) notoginsenoside R1, (6) isomucronulatol-7-O-β-D-glucopyranoside, (7) ginsenoside Rg1, (8) ginsenoside Re, (9) formononetin, (10) ginsenoside Rb1, (11) ginsenoside Rd.

**Part2**

**Analysis of ingredients of SQ absorbed into rat serum**

**Materials and Methods**

***Animals and Experimental Design***

Special pathogen-free (SPF) male Sprague-Dawley (SD) rats were used in this study. The rats were purchased from the Medical Experimental Animal Center of Guangdong Province (Production certification No. SCXK2019-0035, Guangzhou) and maintained in Experimental Animal Center of Guangdong Provincial Hospital of Chinese Medicine (Use certification No. SYXK2018-0094, Guangzhou). All rats were acclimatized for 3 days before experiment and housed in standard condition with constant temperature (20 ± 2 °C), humidity (50 % ± 10 % humidity) and sufficient artificial lighting from 07:00 to 19:00. The rats were given free access to standard laboratory chow and water ad libitum. All animal protocols were conducted in accordance with the Association for Assessment and Accreditation of Laboratory Animals Care International, and approved by Animal Care and Use Committee in Guangdong Provincial Hospital of Chinese Medicine (Guangzhou, China).

Twelve SD rats were randomly divided into two group (n=6 in each group): (1) Control group, (2) SQ group. The dose of SQ was converted in accordance with clinical adult dose. SQ group were given SQ (12.6 mL/kg) by single gavage. Before and 1 hour after gavage, blood samples (0.5 mL) were obtained from the retrobulbar venous plexus using capillary and collected in coagulation-promoting vacuum tubes. After standing on ice for 20 min, blood samples were centrifuged at 3000 rpm for 15 min to collect serum. The serum was stored at -80 ℃ till detection.

***Chromatography and Mass Spectrometry Conditions***

The chromatographic column was Acquity UPLC BEH C18 column (1.7 µm, 100 ×2.1 mm), and the mobile phase included 5 mM ammonium acetate aqueous solution (A) and acetonitrile (B) in gradient mode. Elution procedure was as follows: 0–0.5 minutes, 10–10% B; 0.5–2.5 minutes, 10–40% B; 2.5–4.5 minutes, 40–95% B; 4.5–5.5 minutes, 95–95% B; 5.5–5.6 minutes, 95–10% B; 5.6–6.5 minutes, 10% B, with the flow rate was maintained at 0.3 mL/min. The temperature of the autosampler was 8 ℃.

The multi-component target analysis was conducted by QQQ-MS/MS using ESI ion source and MRM positive and negative ion switching method, and MRM ion pairs were shown in Table 1. Ginsenoside Rg1, Rb1, Rd, Re, notoginsenoside R1, and calycosin were detected in negative, and ononin and caylcosin-7-O-β-D- glucopyranoside were detected in positive. IS was 5500 V and 4500 V respectively, and the atomization temperature was 500 ℃. The collision gas (CAD) was nitrogen. The flow rates of curtain gas (CUR), nebulizer gas (GS1) and auxiliary heating gas (GS2) were 35, 50, and 50 L/min, respectively.

***Preparation of relevant solutions***

For preparation of standard, reference substance of Ginsenoside Rg1, Rb1, Rd, Re, notoginsenoside R1, calycosin, ononin and caylcosin-7-O-β-D-glucopyranoside was weighed accurately, prepared as stock reference substance solution (1mg/ml) at constant volume with methanol, and then diluted to 100 ng/mL as working solution, stored at 4 ℃. The mother liquors of mixed standard including the eight reference substances with the concentration of 20 µg/mL were prepared, and used to prepare QC serum samples (200 ng/mL) mixed with blank serum from rats, and stored at -30 ℃. Eight independent standard working solutions and QC serum samples extracts were detected by LC-MS/MS conditions，and the retention times of MRM chromatogram was determined.

For preparation of serum containing ingredients, 150 µL serum sample was mixed with 750 µL acetonitrile and shook for 5 min, centrifuged at 13000 rpm for 15 min. All the supernatant was dried with nitrogen, mixed with 80 % methanol (150 µL), and then centrifuged through 0.2 µM filter membrane, and detected (3 µL injection volume).

The ingredient of SQ was extracted by SPE method. For preparation of standard curve sample, eight ingredients of SQ were weighed accurately, prepared as mother liquor with methanol, and diluted to 50, 10 µg/mL as mixed standard mother liquor. And mixed standard mother liquor (50 µg/mL) was diluted to 100, 80, 50, 30, 10, 3, 1, and 0.3 ng/mL as series standard curve samples.  And mixed standard mother liquor (10 µg/mL) was diluted to 80, 50, 3, and 1 ng/mL as series QC samples. The reference substances puerarin and saikosaponin a was weighed, prepared as mother liquor with methanol, and diluted as double internal standard work solution (puerarin 200 ng/mL, saikosaponin a 2000 ng/mL). Standard curve, QC, and ingredient liquid samples (200 µL) were mixed with 20 µL double internal standard solution, and vibrated for 2 min, centrifuged at 13000 rpm for 10 min, The injection volume of samples was 3 μL.

**Results**

No relative 6aR, 11aR-3-hydroxy-9,10-dimethoxypterocarpan-3-O-β-D- glucopyranoside and isomucronulatol-7-O-β-D-glucopyranoside reference substance were available. The bioavailability of flavonoid compound formononetin was low(Singh et al., 2011), and MRM chromatographic peak of formononetin was also not detected in SQ-treated serum. Thus, caylcosin-7-O-β-D-glucopyranoside, notoginsenoside R1, ginsenoside Re, ginsenoside Rg1, ginsenoside Rb1, calycosin, ginsenoside Rd, and ononin were our main concerns. In this study, the concentration of the above eight ingredients in SQ and the ingredients absorbed by rats were analyzed. The concentration of main active ingredients in SQ was as follows: ginsenoside Rg1: 495.2 µg/mL, ginsenoside Rb1: 215 µg/mL, ginsenoside Re: 80 µg/mL, ginsenoside Rd: 138.73 µg/mL, notoginsenoside R1: 104 µg/mL, calycosin: 29.6 µg/mL, caylcosin-7-O-β-D-glucopyranoside: 109 µg/mL, ononin: 55.8 µg/mL. And MRM chromatogram of ingredients of SQ in SQ-treated rat serum and blank serum containing standards was further confirmed and shown in Fig. 1. It was shown that main active eight ingredients of SQ could be detected in SQ-treated serum. This data clarified the main ingredients of SQ absorbed by rats using sensitive mass method and indicated that those main active ingredients of SQ could be absorbed into rat blood and were potential pharmacological basis for SQ efficacy.

**Reference**

Singh, S.P., Wahajuddin, Tewari, D., Pradhan, T., and Jain, G.K., 2011. PAMPA permeability, plasma protein binding, blood partition, pharmacokinetics and metabolism of formononetin, a methoxylated isoflavone. Food and Chemical Toxicology. 49(5)**,** 1056-1062. doi: 10.1016/j.fct.2011.01.012.

Tian, R.M., Wang, P.C., Huang, L.H., Li, C., Lu, Z.Y., Lu, Z.S., Wu, A.J., Bao, K., Mao, W., Huang, Q.M., and Xu, P., 2020. Sanqi Oral Solution Ameliorates Renal Ischemia/Reperfusion Injury via Reducing Apoptosis and Enhancing Autophagy: Involvement of ERK/mTOR Pathways. Frontiers in Pharmacology. 11**,** 537147. doi: 10.3389/fphar.2020.537147.
